# Persistence Vulnerability of the African Savanna Elephant *Loxodonta africana* to Anthropocene Threats in East Africa

**DOI:** 10.1002/ece3.74389

**Published:** 2026-09-24

**Authors:** Ahmed Seid Ahmed, Daniel Melese, Mulatu Ayenew Aligaz, Anagaw Atickm, Chala Adugna Kufa

**Affiliations:** ^1^ Department of Biology Hawassa University Hawassa Ethiopia; ^2^ Ohio State Global One Health Initiative (GOHi) Addis Ababa Ethiopia; ^3^ Department of Biology Mizan‐Tepi University Tepi Ethiopia; ^4^ Department of Plant Biology and Biodiversity Management Addis Ababa University Addis Ababa Ethiopia; ^5^ Department of Zoological Sciences Addis Ababa University Addis Ababa Ethiopia; ^6^ Department of Biology Debre Markos University Debre Markos Ethiopia; ^7^ Department of Biology Woldia University Woldia Ethiopia

**Keywords:** climatic refugia, East Africa, ensemble modeling, *Loxodonta africana*, protected areas

## Abstract

Anthropogenic climate change caused by human activities reduces suitable habitats for species. This effect is especially pronounced for tropical species, where agricultural expansion and habitat degradation interact with climate‐driven stressors. Spatial conservation prioritization for vulnerable species requires species distribution modeling to evaluate effects of climate change and human pressures. We mapped the current and future extent of suitable habitat for the African Savanna Elephant, *L. africana* , in East Africa using an ensemble species distribution model. We built an ensemble model with 4298 presence records and 10 key environmental predictors, averaging seven algorithms. For each algorithm, 10 model replications and two thresholds were used to map suitable habitat under current and future (2050, 2070) climate scenarios. Suitable habitats' overlap with existing protected areas (PAs) was assessed that may serve as Anthropocene refugia. The results demonstrated high performance for all algorithms with an average AUC value of 0.94, with a significant difference in evaluation metrics (*p* < 0.05) among algorithms. We identified 887,094 and 754,737 km^2^ suitable habitats using the weighted mean and 10% omission‐rate thresholds, respectively. However, these suitable habitats are projected to decline on an average of 51.9% by 2050 and 52.6% by 2070. These projected suitable habitats are severely reduced and fragmented under the worst‐case climate scenario (SSP5‐8.5, 2050 and 2070), with large portions occurring outside PAs and many PAs projected to become unsuitable. Reductions in remaining suitable habitats and climatic refugia within PAs also support this conclusion. Most suitable areas outside PAs highlight the need for conservation and species management action, which is more challenging. We recommend safeguarding currently suitable habitats, even if projected to become climatically unsuitable, and protecting future suitable areas outside PAs, including stable habitats and Anthropocene refugia. This will help ensure long‐term conservation, particularly for populations at risk of local extinction.

## Graphical abstract

Anthropogenic climate change and habitat degradation are rapidly reshaping and reducing tropical habitats, threatening vulnerable species. We aimed to map current and future climatically suitable habitats for the African savanna elephant ( *Loxodonta africana* ) in East Africa using a species distribution modeling framework and to identify Anthropocene refugia within and beyond protected areas (PAs). We built an ensemble model using 4298 presence records and 10 key environmental predictors across seven algorithms. For each algorithm, 10 model replications and two threshold criteria were applied to map potential suitable habitats under current and future climate scenarios (2050 and 2070).

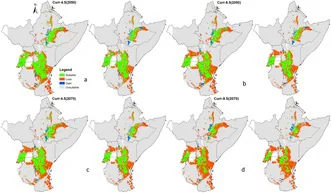

## 1 Introduction

Anthropogenic climate change reduces habitat suitability for tropical species, particularly in regions where agricultural expansion and habitat degradation intensify climate driven stressors (Brawn et al. 2017; Ahmed, Bekele, et al. 2023; Ahmed, Chala, et al. 2023). Anthropogenic pressures have emerged as primary factors limiting species distributions (Monsarrat, Jarvie, et al. 2019; Monsarrat, Novellie, et al. 2019; Head et al. 2022). These pressures act together with climate change to alter species distributions and restrict their suitable habitats (Kufa et al. 2022; Hameed et al. 2025; Lawer et al. 2025). Whenever there are natural and Anthropocene pressures, some areas act as refugia, offering protection and persistence for biodiversity (Monsarrat, Jarvie, et al. 2019; Monsarrat, Novellie, et al. 2019; Kufa et al. 2025). The concept of refugia was previously applied to climatic change and describes stable areas that taxa survive in during climatic fluctuations during glacials (Médail and Diadema 2009). However, recent studies by Monsarrat, Jarvie, et al. (2019), Monsarrat, Novellie, et al. (2019), and Kufa et al. (2025) have broadened this concept, demonstrating that suitable habitats both within and beyond protected areas (PAs) can function as Anthropocene refugia, thereby contributing to biodiversity persistence in an increasingly human‐dominated world. Identifying key areas for species conservation is essential for the long‐term protection of species (Kufa et al. 2025). It supports effective climate change adaptation and reduces uncertainty in conservation planning (Sales and Pires 2023). This is critical for improving conservation efforts worldwide (Lezama‐Ochoa et al. 2025).

Climate and land cover changes pose significant threats to wildlife, particularly African elephants, by reducing the availability of water and forage resources (Dejene et al. 2021). Currently, three global extant elephant species are known: the Asian Elephant ( *Elephas maximus* ), African Savanna Elephant ( *Loxodonta africana* ), and African Forest Elephant ( *Loxodonta cyclotis* ) (Gobush et al. 2022; Kuhner et al. 2025). The latter two species are found in continental Africa (Zuba and Mama 2025) has recently been recognized as separate species (Kuhner et al. 2025). *Loxodonta africana* is a keystone species and emblem of Africa's biodiversity, playing a significant role in maintaining and shaping African ecosystems (Gross and Heinsohn 2023). It occurs currently in 24 countries (Gobush et al. 2021). It is listed as endangered by the International Union of the Conservation of Nature (IUCN) (Gobush et al. 2021; Gross and Heinsohn 2023), due to severe threats and significant population declines (de Sales et al. 2020; Gobush et al. 2021), resulting in the local extirpation of many subpopulations.

Over the last century, there is a significant decline in elephant populations due to increasing anthropogenic pressure (Gross and Heinsohn 2023). Scientists estimate that Africa's Savanna and Forest Elephants together number about 415,428 (±95% CI: 20,111). However, surveys covering nearly 90% of the African Savanna Elephant's range reveal a 30% decline in population size (Chase et al. 2016). Reason for population reduction of mega‐herbivores, *L. africana* , perhaps include habitat loss due to human population expansion, global intensification of agriculture (Montero‐Botey et al. 2024) and poaching (Kuiper et al. 2020). As human populations grow and agriculture expands, their habitats are reduced, fragmented, and encroached, leading to more direct conflict (Sach et al. 2019; Sanare et al. 2022; Montero‐Botey et al. 2024). Farming, charcoal production, and infrastructure development further degrade and fragment elephant ranges (Githiru et al. 2017). Because people and elephants often share the same land and scarce resources, land disputes have become the greatest long‐term threat to elephant survival (Sanare et al. 2022), which leads to crop raiding (Røskaft et al. 2014). Across Africa, increasing competition for space, food, and water continues to intensify human elephant conflict (Lee and Graham 2006; Gross et al. 2022; Leneuiyia et al. 2025).

Human activities like fencing, farming, and dense settlements are cutting elephants off from their natural ranges (Huang et al. 2022). They have been critically endangered from parts of the Horn of Africa, including Somalia. In Eritrea, the elephants' home ranges are increasingly restricted, isolated, and highly vulnerable. In Ethiopia, pressures like intensive hunting, habitat loss, and rapid human population growth have restricted elephants to a limited number of PAs (Dejene 2016; Wale et al. 2017). As a result, elephants are rarely seen, signaling potential local extirpations and alterations in spatial ecology. To cope, elephants often adjust their behavior—moving faster through risky areas to maintain links between fragmented refuges (Graham et al. 2009) and increasing nocturnal movements to access forage and water resources. Yet, expanding agriculture and infrastructure continue to disrupt their movement patterns (Mukomberanwa et al. 2025), squeezing corridors and limiting access to diverse resources, as seen in the Amboseli ecosystem (Gara et al. 2021). However, emerging cross‐border movements of the elephants highlight the need for coordinated transboundary management (Geleta and Girma 2022; Neil and Greengrass 2022). Human footprints shape their paths and create resistance to movement across transboundary regions (Cushman and Huettmann 2010). Ultimately, habitat loss and fragmentation near PAs threaten elephant survival by blocking vital wildlife corridors (Giliba et al. 2023).

Under climate change, species‐specific conservation strategies requires modeling habitat suitability and its distribution patterns (Chala et al. 2019; Dejene et al. 2021; Ahmed, Chala, et al. 2023). Despites their range expansion in some localities such as Kenya and Botswana (Thouless et al. 2016), their distribution is contracting and becoming increasingly fragmented (Dejene et al. 2021). The study predicted 51%–68% loss of suitable habitat by 2050 under Representative Concentration Pathway (RCP) 4.5 and RCP 8.5, and 75%–86% loss by 2070. Mpakairi et al. (2020) predict a likely 40% shrinkage in the potential *L. africana* habitat by 2050, while future climate projections suggest a decline and fragmentation of highly suitable African elephant habitat, especially along the southern and eastern edges of Hwange National Park Boas and Zvidzai (2025), and climate change could be causing a shift in how it uses its landscape (Lowe 2017). Most of the suitable habitat for the species extends beyond PAs and is therefore vulnerable to human pressures (Wall et al. 2021). Studies indicated that habitat fragmentation, land‐use conversion, and poaching are restricted to approximately 17% of its suitable habitat, over half of which falls outside formally PAs (Chase et al. 2016; Edwards et al. 2024). The projected substantial contractions in species range sizes emphasis the necessity of targeted, region‐specific climate impact evaluations (Dejene et al. 2021; Nampindo and Randhir 2024).

Whereas, habitat suitability of *L. africana* are impacted by high temperature, high rainfall, and drought; persistence of extreme temperature (without precipitation) has a net negative impact to *L. cyclotis* (Natarajan et al. 2025). A study conducted by de Knegt et al. (2011) indicate the predictive habitat suitability models for African elephants increases when environmental context is included at characteristic spatial scales. For example, temperature, precipitation‐related variables, distance to water and park boundary, NDVI, slope, and elevation can determine the spatiotemporal distribution of suitable habitats for *L. africana* (Boas and Zvidzai 2025). Likewise, vegetation types and distances from human settlements are biologically meaningful for habitat use modeling (Harris et al. 2008). In northern Kenya, water, terrain features, and human impacts are the primary factors shaping the African savanna elephants' space use and influencing habitat suitability (Gathuku and Wittemyer 2025). Furthermore, in East Africa, the distribution of elephants are best predicted by habitat and other landscape features (Epps et al. 2011). Importantly, its site habitation is also governed by annual rainfall and the number of neighboring water holes (Martin et al. 2010). All of these factors profoundly alter ecosystems and accelerate extinction risks for terrestrial vertebrates (Powers and Jetz 2019; Baisero et al. 2020), thus demanding continued protection for wildlife survival (Van Jaarsveld et al. 1999), including the elephant species.

**FIGURE 1:**
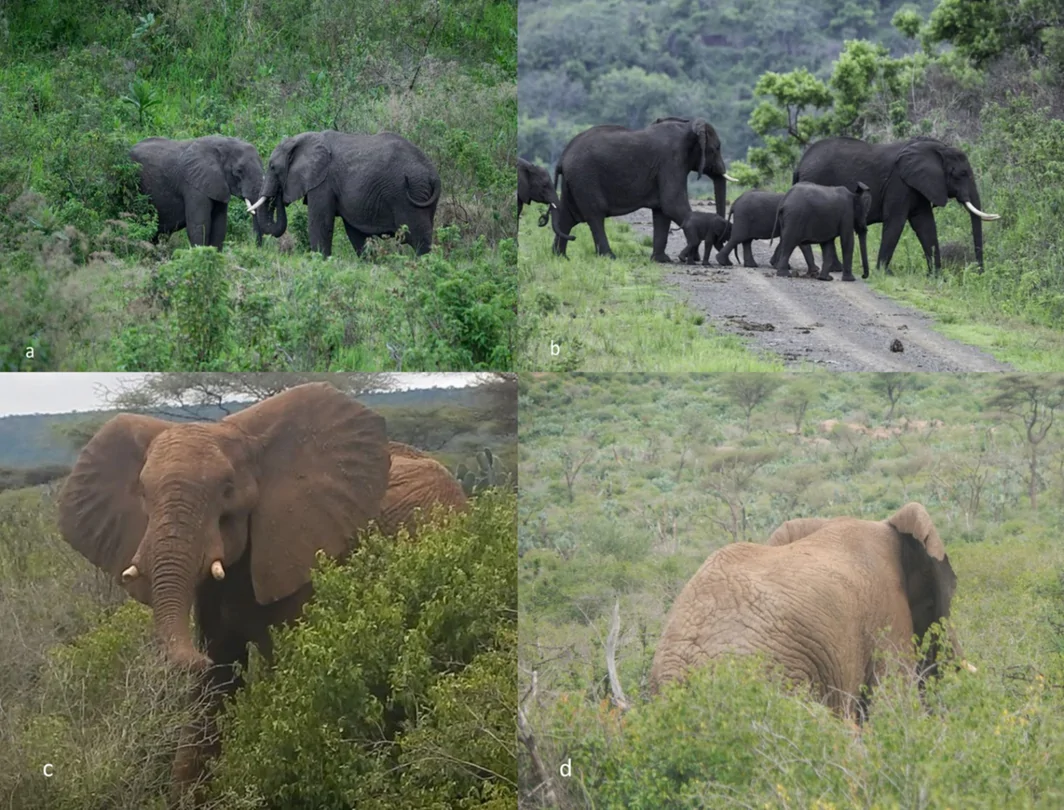
African Savanna Elephant ( *Loxodonta africana* ): (a) a breeding pair; (b) a family herd crossing a road; (c) an adult male; and (d) a herd foraging in an open field. Photographs were taken in Chebera Churchura National Park (a, b) and Babile Elephant Sanctuary, Ethiopia (c, d). Photos credit—Solomon Worku and Teshitu Desalegne.

Climate change is reshaping the future of Africa's savanna elephants (Zacarias and Loyola 2018). Rising temperatures and changing rainfall patterns cause physiological stress and alter migration habits (Hines et al. 2023; Natarajan et al. 2025). Additionally, the degradation of forests and wetlands threatens elephant survival by impacting cooling and shelter availability (Nampindo and Randhir 2024). In desert areas, diminished vegetation and water sources force elephants to change behaviors, heightening risks to their long‐term survival (Shiweda et al. 2023). In this study, we aimed to build habitat suitability modeling to assess future Anthropocene refugia that supports the long‐term persistence of the target species (Figure 1), under changing environmental conditions. Determining suitable refugia for megafuana like *L. africana* is very critical for initiating conservation actions in areas where human pressures are intensifying. We developed an ensemble species distribution model for *L. africana* in Eastern Africa to address the following objectives: (1) identify the key environmental predictors for suitable habitat of *L. africana* ; (2) determine the extent of suitable habitats under current and future (2050 and 2070) climate conditions; (3) assess the spatiotemporal dynamics in the suitable habitats range; and (4) identify Anthropocene refugia (suitable habitats inside of PAs) for *L. africana* . Filling such knowledge gap is critical to develop adaptive, regionally coordinated conservation strategies to address both human pressures and long‐term environmental change.

## 2 Materials and Methods

### 2.1 Species Occurrence Data

**FIGURE 2:**
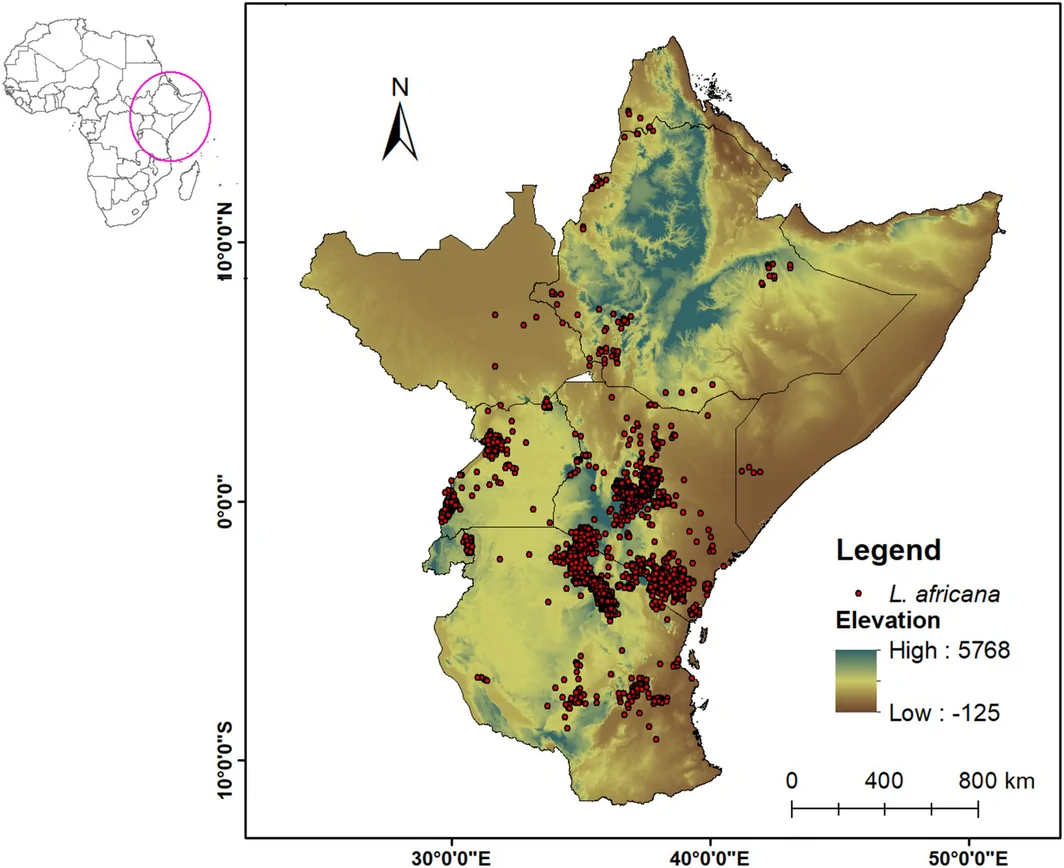
Map showing the occurrences of *L. africana* (red dots) across ranges in the East African countries, namely Eritrea, Ethiopia, South Sudan, Somalia, Kenya, Uganda, United Republic of Tanzania and Rwanda.

We compiled occurrence data for *L. africana* from different sources, including personal field surveys (number = 36) in some of Ethiopia's National Parks (NPs) such as Omo, Mago, Chebera Churchura and Babile Elephant Sanctuary; from published literatures (number = 69; Demeke and Bekele 2000; Ihwagi et al. 2009; Reddy 2014; Eastern Africa 2016 African Elephant Status Report 2016; Lohay et al. 2020; Wale et al. 2021; Jeza and Bekele 2023, 2024; Temesgen and Warkineh 2025); and from Global Biodiversity Information Facility (GBIF.org, 2025; number = 4193) (Figure 2; Table S1). A total of 6805 species occurrence records were compiled from freely available, open‐access sources and used with appropriate acknowledgment. Following preprocessing and the removal of spatially redundant records, 4298 occurrence points were retained for final model development. Field surveys were conducted by the authors between 2021 and 2024 using ground‐based data collection techniques. The species occurrence records with accurate geographic coordinates were retained, while those with uncertain locations were excluded. The species occurrence records used for modeling restricted to those collected or documented since 1990s to accurately represent current distributions due to historical shifts in species ranges (Monsarrat, Jarvie, et al. 2019; Monsarrat, Novellie, et al. 2019).

However, before use of these species occurrence for modeling the suitable habitat of *L. africana* , its reliability was ensured through a multi‐step data cleaning process. First, we rarefied and aligned the species occurrence data to a 1 × 1 km raster grid resolution of the bioclimatic used as environmental variables using SDMtoolbox version 2.5 (Brown 2014) in ArcGIS v10.8. This rarefication process minimize spatial autocorrelation (Guisan et al. 2006). Then, duplicate occurrence points were removed and finally 4298 species occurrence was retained for the final model prediction and projection. In addition, we generated 10,000 random points as pseudo‐absence data within the target study area to balance computational efficiency and model robustness, reduces overfitting and makes predictions more reliable (Kufa et al. 2025). These data have also a substantial influence on predictive performance, particularly in identifying key predictor variables (Barbet‐Massin et al. 2012). Using the presence (represented by 1) and pseudo‐absence (represented by 0) data compiled for modeling, sample‐with‐data (SWD) for each point with its environmental predictors was generated using the USDM package (Naimi 2017) in R software version 4.3.3 (Ahmed, Bekele, et al. 2023; Table S1).

### 2.2 Environmental Predictor Variables

To model the habitat suitability and distribution of *L. africana* in the East Africa, 20 environmental variables, including 19 bioclimatic predictors and the human footprint, were incorporated. These environmental predictors were used to model the habitat suitability and distribution of *L*. *africana* across continental Africa (Dejene et al. 2021; Wall et al. 2021). The 19 bioclimatic variables were obtained from the WorldClim 2.1 database (www.worldclim.org/bioclim) at a spatial resolution of 30 arc seconds (~1 km^2^), based on interpolated climate data from 1970 to 2000 (Fick and Hijmans 2017). To represent anthropogenic disturbance, a Human Footprint Pressure raster (Venter et al. 2016) obtained from SEDAC (https://sedac.ciesin.columbia.edu/data/set/wildareas‐v3‐2009‐human‐footprint) was used. This raster layer reflects the cumulative intensity of human activities such as roads, settlements, agricultural zones, and infrastructure on a scale from 0 to 50 with a spatial resolution of approximately 1 km^2^ (Di Marco et al. 2018; Hending et al. 2023).

These selected 20 environmental variables were stacked and their values extracted by the combined species occurrence and 10,000 randomly generated pseudo‐absence points. These pseudo‐absence points captured the full range of environmental conditions across the targeted study area, enabling more reliable variable selection and helping to reduce model overfitting. To ensure environmental variable independence, we performed a multicollinearity test and retained only those variables with pairwise correlation coefficients below 0.7 and variance inflation factors (VIF) under 5 (Guisan et al. 2017). Among the highly correlated variables, one of the variables was removed iteratively using a stepwise procedure based on VIF, implemented with the “usdm” package in R (Naimi et al. 2014). As a result, 10 environmental predictor variables were retained and used for habitat suitability model development (Table 1).

**TABLE 1:** Selected variables with their variance inflation factor (VIF).

No	Variables	Units	Code	VIF
1	Mean diurnal range (Mean of monthly)	°C	BIO2	1.75
2	Isorthemality (BIO2/BIO7) × 100	°C	BIO3	1.98
3	Mean temperature of wettest quarter	°C	BIO8	1.99
4	Mean temperature of driest quarter	°C	BIO9	1.58
5	Precipitation of warmest month	mm	BIO13	4.36
6	Precipitation of driest month	mm	BIO14	2.33
7	Precipitation seasonality	mm	BIO15	2.01
8	Precipitation of warmest quarter	mm	BIO18	4.36
9	Precipitation of coldest quarter	mm	BIO19	2.23
10	Human footprint	—	Human_Fp	1.18

For future habitat suitability modeling, we used the HadGEM3‐GC global circulation model (GCM) from the Coupled Model Intercomparison Project Phase 6 (CMIP6). This GCM was selected for its demonstrated scientific robustness, computational efficiency, and broad adoption in climate research (Kuhlbrodt et al. 2018). Two shared socioeconomic pathways (SSPs) were selected for projections targeting the years 2050 and 2070. Whereas the first SSP2‐4.5 represents intermediate greenhouse gas emissions with CO_2_ concentrations increasing until 2050 and then declining to net zero by 2100, the second SSP5‐8.5 represents very high greenhouse gas emissions where CO_2_ levels triple by 2100 (Riahi et al. 2017). Consistent with CMIP6 standards (Guarnieri et al. 2024), the selected GCM model effectively produces historical climate variability, incorporates advanced aerosol and climate interactions, and provides high‐resolution atmospheric enabling improved regional representation and reliable projections for the target study area (Roberts et al. 2019; Melese, Aligaz, and Ahmed 2025; Melese, Lemessa, et al. 2025).

### 2.3 Species Distribution Modeling

We applied an ensemble modeling framework that integrates regression and machine learning algorithms to improve predictive robustness (Hijmans and Elith 2021). Among the regression algorithms, we used generalized linear models (GLM), generalized additive models (GAM), and multivariate adaptive regression splines (MARS). From the machine learning algorithms, we employed boosted regression trees (BRT), maximum entropy (MaxEnt), random forests (RF), and support vector machines (SVM) to improve predictive accuracy and model robustness (Santini et al. 2021). The selection of modeling algorithms was driven by their alignment with the data structure and their consistently demonstrated performance in ecological modeling contexts (Peterson et al. 2015; Tredennick et al. 2021). All these algorithms are widely used in research and species conservation (Zhang and Li 2017; Ahmed, Bekele, et al. 2023).

The model performance was optimized using both default and customized parameter settings. The setting included adjustments to background point generation, iteration limits, learning rates, feature classes, and tree numbers. To mitigate sampling bias and prevalence imbalance, case weights were applied to presence and pseudo‐absence data during model calibration (Melese, Aligaz, and Ahmed 2025; Melese, Lemessa, et al. 2025). Algorithm‐specific configuration was implemented to enhance model robustness. The BRT model was run with a learning rate of 0.05, 500 trees, and a bag fraction of 0.75, while the RF model employed 500 trees. The GLM and GAM models were specified with a binomial family (Hastie et al. 2009; Thuiller et al. 2021). The MaxEnt model was run with 1000 iterations and a regularization multiplier of 1 (Elith et al. 2008; Barbet‐Massin et al. 2012; Thuiller et al. 2021; Valavi et al. 2021; Thuiller et al. 2023). The SVM model was implemented using a radial kernel with a cost parameter of 1, and the MARS model was configured with degree 2 and nprune 30 to optimize model performance (Naser et al. 2022).

All species distribution models (SDMs) were implemented using the sdm package in R (Naimi et al. 2014; Ahmed, Bekele, et al. 2023; Ahmed, Chala, et al. 2023). The models were trained on 70% of the occurrence data and validated on the remaining 30% using a 10‐fold subsampling scheme (Wang et al. 2022). We randomly generated 10,000 background points across the study area using the gRandom method within the sdmData function. These points served as pseudo‐absences for algorithms requiring binary inputs and as background for MaxEnt, ensuring consistency across modeling approaches. Finally, an ensemble prediction was obtained by averaging model outputs weighted by their respective true skill statistic (TSS), following established best practices to enhance accuracy and minimize predictive uncertainty (Dormann et al. 2018).

### 2.4 Model Validation and Mapping

**FIGURE 3:**
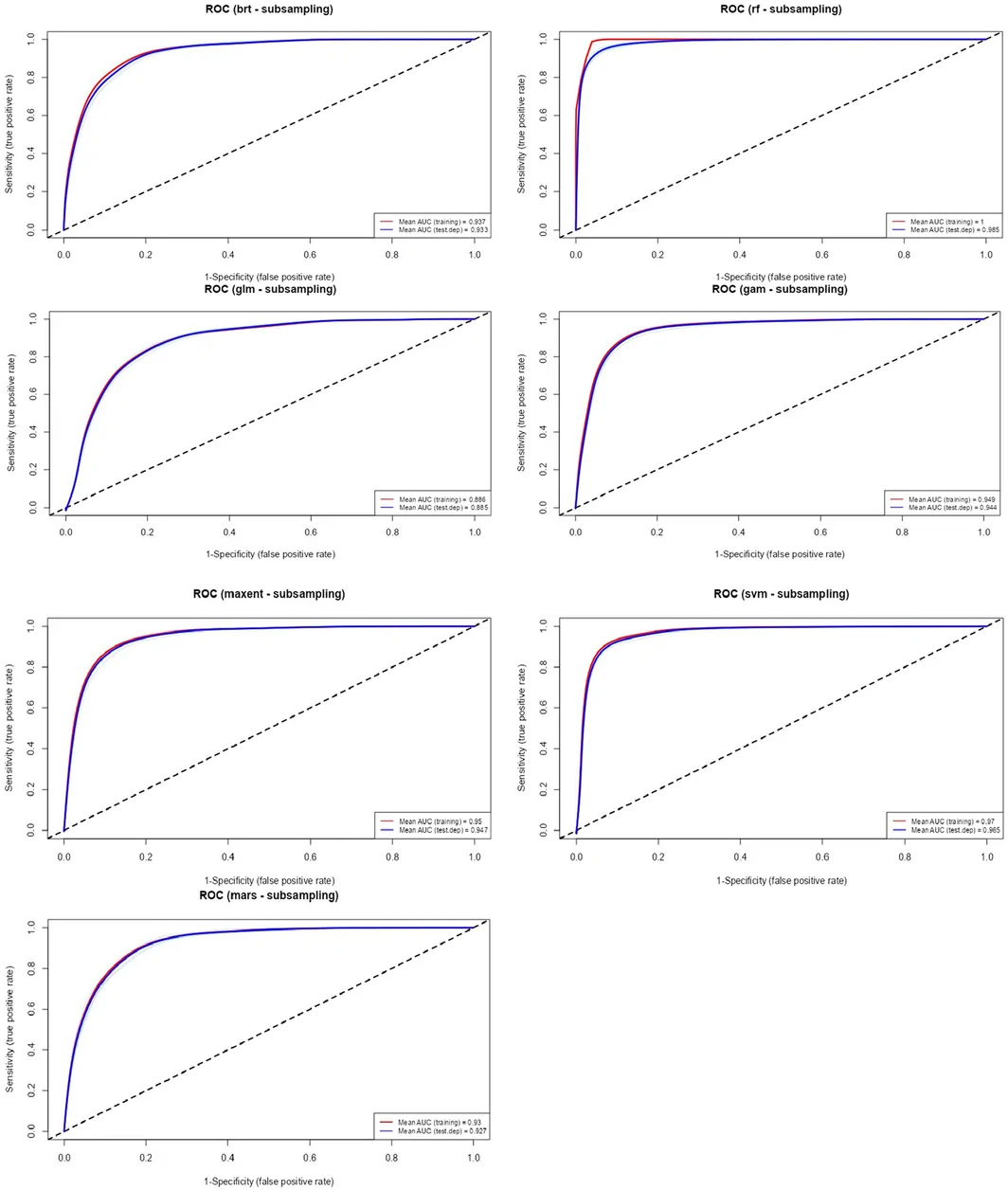
Receiver Operating Characteristic (ROC) curves for *Loxodonta africana* generated using seven modeling algorithms: BRT, RF, GLM, GAM, MaxEnt, SVM, and MARS. The curves illustrate the Area under the receiver operating characteristic curve (AUC) for both training and testing datasets. Higher AUC values indicate greater model performance, while lower values reflect reduced predictive accuracy.

To evaluate the predictive SDMs, we employed complementary metrics such as receiver operating characteristic (ROC), TSS, Kappa statistic, Pearson correlation coefficient (COR), maximum sensitivity plus specificity (max (se + sp)) (Ahmed, Bekele, et al. 2023; Ahmed, Chala, et al. 2023; Kufa et al. 2025). As a threshold‐independent measure of predictive accuracy, we plotted ROC curves (Figure 3) and calculated the area under the curve (AUC) (Allouche et al. 2006; Duan et al. 2014). Its value ranges from 0 to 1, where values < 0.5 indicate performance no better than random, 0.7–0.8 is considered fair, 0.8–0.9 denotes good, and values exceeding 0.9 indicate excellent performance. TSS and max (se + sp) were also evaluated as threshold‐dependent metrics. TSS values range from −1 to +1, where 1 denotes perfect prediction, 0 indicates random performance, and values above 0.6 generally signify good model performance. Likewise, Kappa statistic values > 0.55 indicate good evaluation performances (Ahmed, Bekele, et al. 2023; Ahmed, Chala, et al. 2023). Sensitivity measures correctly predicted presences, and specificity captures correctly classified absences. Both are essential for robust model evaluation (Bulluck et al. 2006). Additionally, the COR is an important metric for evaluating model performance by measuring the relationship between predicted occurrence probabilities and presence and pseudo‐absence test data (Valavi et al. 2021). The strong COR (0.98) shows that it effectively quantifies the relationship between predicted probabilities and test data (Li and Guo 2013). It has useful potential, but it should be applied alongside multiple performance metrics when evaluating SDMs (Mainali et al. 2017).

We used two cutoff thresholds to reduce the uncertainty and class agreement of the suitable habitat predicted. The maximum sensitivity plus specificity (max (se + sp)) was used as a threshold across the seven models to transform the probabilistic occurrence prediction maps into binary presence‐absence maps. This approach aligns with the TSS, a widely applied accuracy metric in ecological modeling (Allouche et al. 2006). To improve the robustness of the max (se + sp), we applied a TSS weighted mean, giving greater weight to higher performing models (Liu et al. 2013; Ahmed, Bekele, et al. 2023; Melese, Aligaz, and Ahmed 2025; Melese, Lemessa, et al. 2025). Additionally, we used a 10‐percentile logistic training threshold by averaging the seven model predictions. The mean threshold values were 0.30 for Wm and 0.34 for the 10% threshold.

$$\mathrm{Wm}=\frac{{\mathrm{Ths}}_{1}1\times {\mathrm{TSS}}_{1}+{\mathrm{Ths}}_{2}\times {\mathrm{TSS}}_{2}\ldots {\mathrm{Ths}}_{n}\times {\mathrm{TSS}}_{n}}{{\mathrm{TSS}}_{1}+{\mathrm{TSS}}_{2}\ldots {\mathrm{TSS}}_{n}}$$

where: Wm = weighted mean, Ths = threshold one, *n* = number of threshold and TSS.

$$10\%=\frac{{\mathrm{Ths}}_{1}1+{\mathrm{Ths}}_{2}\ldots {\mathrm{Ths}}_{n}}{n}$$

where: 10% = 10 percentile omission rates, *n =* number of replications.

Incorporating multiple thresholds and model complexities is crucial for reducing uncertainty in determining suitable habitat predictions (Ahmed, Bekele, et al. 2023). Based on these thresholds, pixels were classified as suitable or unsuitable in the final binary habitat maps. The suitable habitats and distribution patterns for *L. africana* across East Africa were then mapped using ArcGIS 10.8.

We overlaid the current ensembled predicted suitable habitat maps with future projected ensembled predictions (2050 and 2070) using two cutoff thresholds: weighted mean and 10% (10 percentile omission) to detect spatiotemporal changes in suitable habitats and quantify the range size change or impact of climate changes. The current binary output was intersected with projected ensembled prediction for each future climatic scenario (Qin et al. 2017; Zhang et al. 2016). These intersections are essential for evaluating species range size changes. Using ensembled future projections for 2050 and 2070 generated under SSP2‐4.5 and SSP5‐8.5, we quantified the spatial extent of areas classified as unsuitable, stable, lost, and gained in ArcGIS 10.8. These outputs were then used to assess range shifts and potential climate change impacts on species distributions, including patterns of habitat gain, loss, and redistribution (Chala et al. 2016; Gebremedhin et al. 2021; Kufa et al. 2025).

### 2.5 Spatial Overlap of Suitable Habitats With PAs Networks

We overlaid the current ensembled suitable habitats for *L. africana* onto PAs networks of East Africa using ArcGIS 10.8, based on two cutoff thresholds: the weighted mean and the 10% percentile omission rate. The regional PAs network layer was obtained from UNEP‐WCMC and IUCN (2026). We then quantified the spatial extent (or size in square kilometers) of three categories: (1) suitable habitat outside PAs, (2) areas of overlap between suitable habitat and PAs, and (3) PAs that are unsuitable under current climate conditions.

### 2.6 Identification of Anthropocene Refugia

We defined Anthropocene refugia as suitable habitats laid inside of PAs network in East Africa. Such refugia under protection were identified by overlaying predicted suitable habitat maps with East Africa's PAs networks and computing their spatial intersections in square kilometers. The total refugia area within PAs was quantified using merged regional protected area layers obtained from UNEP‐WCMC and IUCN (2026), www.protectedplanet.net; access, 2025.

## 3 Results

### 3.1 Variables That Predict the Distribution of Suitable Habitats *L. africana* Under Current Climate Condition

**FIGURE 4:**
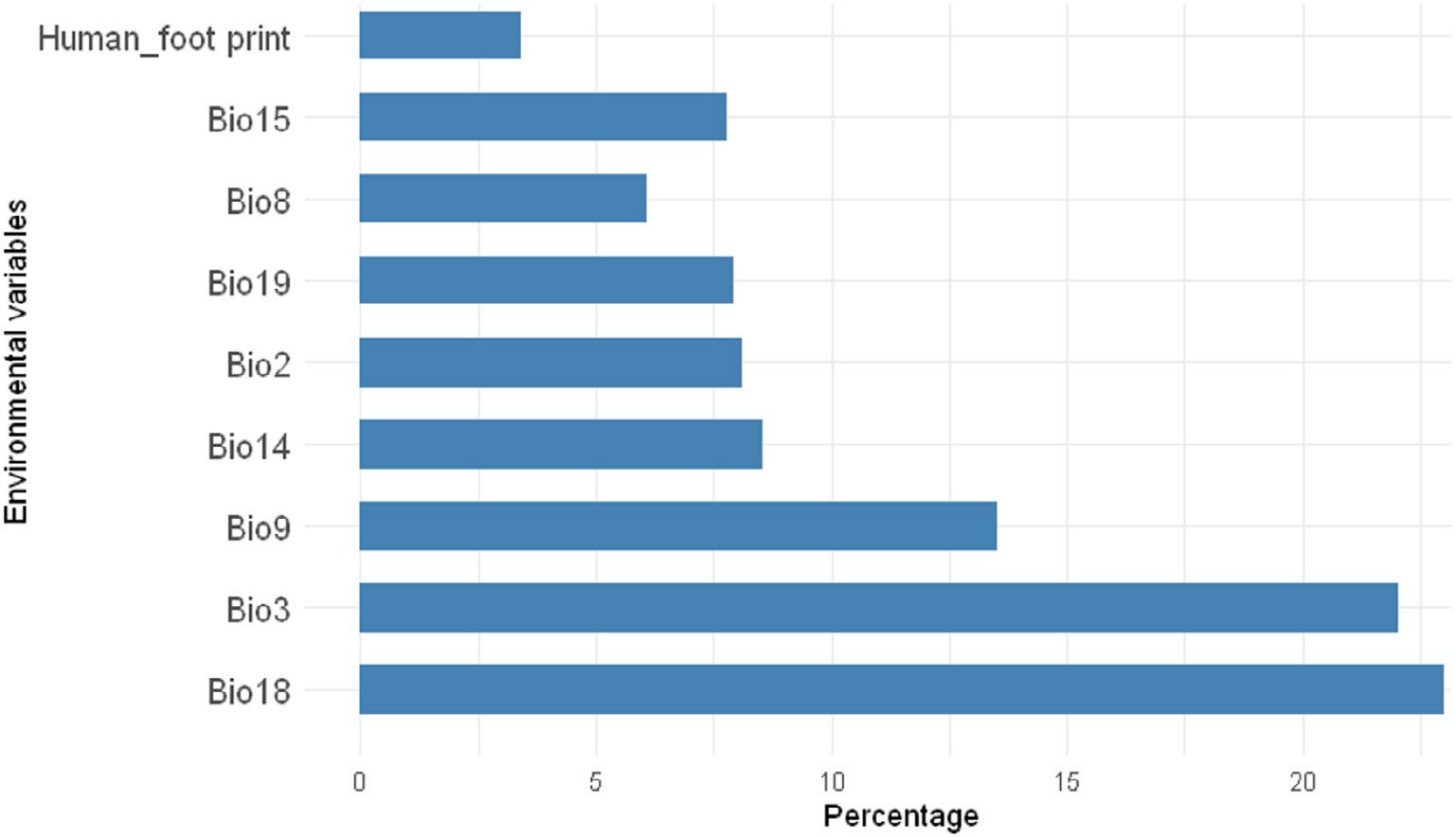
The relative importance of bioclimatic variables for predicting habitat suitability of *L. africana* , based on the final ensemble model. Values represent the averaged relative importance derived from all algorithms used in the ensemble model.

Across all algorithms and settings, the most influential environmental predictors of *L. africana* habitat suitability were precipitation of the warmest quarter (Bio18), Isothermality (Bio3), mean temperature of the driest quarter (Bio9), and precipitation of the driest month (Bio14) (Figure 4). In contrast, human footprints were less influential for predicting the potential habitat suitability of the target species.

### 3.2 Models' Performances and Their Difference Among Algorithms

All algorithms demonstrated high predictive performance (average AUC values of 0.94 ± 0.029) and the ensemble models performed strongly on both the training and test datasets (Table 2). Other evaluation metrics showed sensitivity of 0.91 ± 0.022 and specificity of 0.86 ± 0.056. Among algorithms, RF and SVM exhibited substantially excellent performance across all evaluation metrics. The TSS, KAPPA, and COR metrics also indicated very good model performance. In this study, the machine‐learning algorithms, such as RF and MaxEnt, performed significantly better than the regression‐based models (*p* < 0.05). These high performances of most algorithms are substantially important for reducing uncertainty in determining the potential suitable habitats of *L. africana* . The minimal standard deviations of the AUC and sensitivity values (0.029 and 0.022, respectively) indicate very low variability and high consistency, suggesting sufficient dataset to reliably predict the habitat suitability of *L. africana* (Table 2). The TSS, KAPPA, COR, and specificity values also showed low variability, ranging between 0.056 and 0.097. Low variability in the evaluation metrics (SD: 0.022–0.097) underscores the robustness and consistency of *L. africana* habitat predictions across all algorithms (Table 2).

**TABLE 2:** The overall mean performance of the algorithms evaluated metrics (i.e., AUC, TSS, KAPPA, COR, sensitivity, and specificity) and cutoff thresholds (i.e., weighted‐mean threshold and 10%—10 percentile logistic training threshold).

Algorisms	Evaluation metrics						Thresholds	
	AUC	TSS	KAPPA	COR	Sensitivity	Specificity	WM	10 percentiles
BRT	0.93	0.74	0.65	0.72	0.91	0.83	0.26	0.27
RF	0.98	0.89	0.86	0.86	0.95	0.95	0.32	0.50
GLM	0.88	0.65	0.55	0.65	0.87	0.78	0.25	0.16
GAM	0.94	0.78	0.73	0.78	0.92	0.86	0.29	0.32
Maxent	0.95	0.79	0.71	0.79	0.91	0.87	0.43	0.44
SVM	0.97	0.84	0.8	0.84	0.92	0.93	0.33	0.40
MARS	0.93	0.73	0.63	0.73	0.91	0.82	0.25	0.27
Ensemble	0.94	0.78	0.71	0.77	0.91	0.86	0.30	0.34
SD	0.029	0.072	0.097	0.068	0.022	0.056	0.056	0.11

Abbreviations: AUC, area under curve; COR, correlation coefficient; KAPPA, Kappa statistic; SD, standard deviation; TSS, true skill statistics; Sens, sensitivity; Spec, specificity.

**FIGURE 5:**
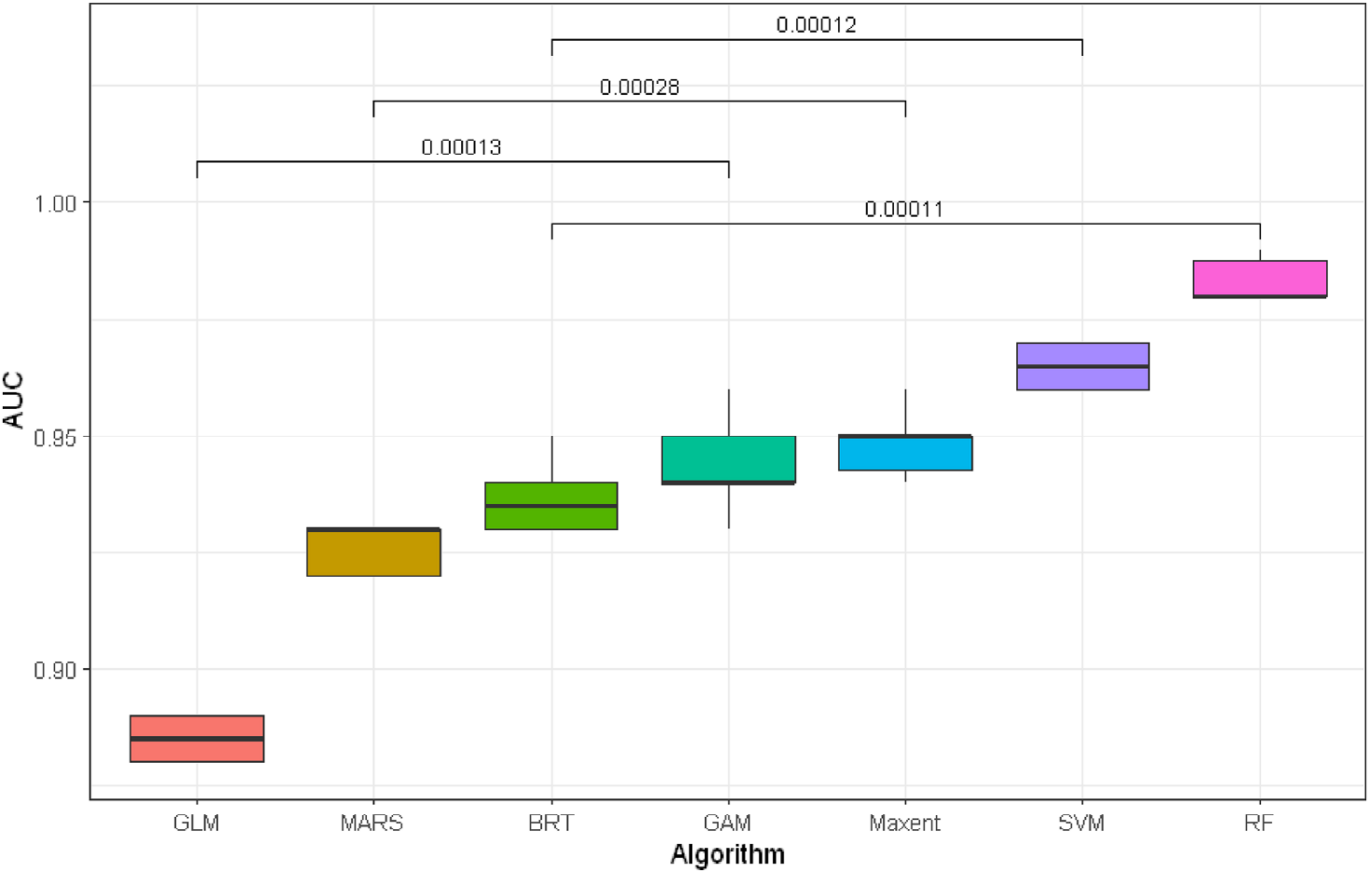
Model performance (AUC) of the algorithms for predicting habitat suitability of the *L. africana* . Boxplots depict the distribution of AUC scores across 10 replicates for each algorithm. Pairwise comparisons were performed using the Wilcoxon rank‐sum test, with statistically significant differences indicated and corresponding *p*‐values shown on the plot.

No substantial differences in several pairwise evaluation metric comparisons were observed among the algorithms (Table 2). However, significant differences were observed in AUC among the algorithm types between GLM, MARS and GAM (*p* < 0.05). There was also a significant difference observed between BRT and MaxEnt (*p* < 0.05) (Figures 3 and 5). Despite variations in algorithmic performance, particularly in AUC and TSS, sensitivity and specificity remained consistent across both training and test datasets. Although significant variations were observed among algorithms and across evaluation metrics, the predictive outputs demonstrated high stability in relation to model complexities, repeated iterations, and datasets.

### 3.3 Current Habitat Suitability Modeling

**FIGURE 6:**
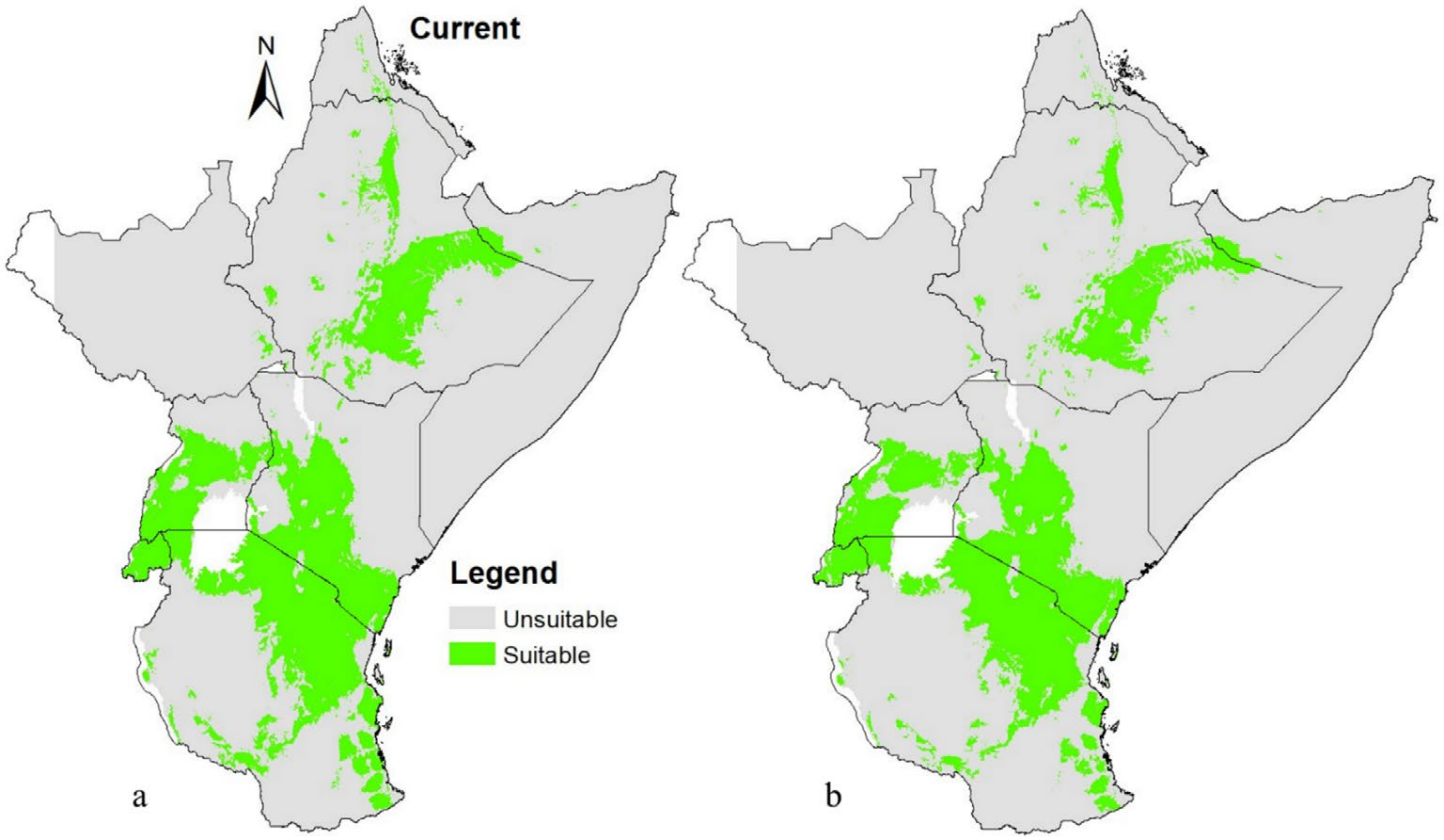
Extent of suitable habitats produced using ensemble models under current climate conditions: (a) the weighted mean and (b) the 10% threshold (10th percentile omission rate).

Under the current climate, the models predicted areas of 887,093.6 and 754,736.9 km^2^ suitable habitats for *L. africana* by the weighted‐mean and the 10‐percentile omission rate (10%) thresholds, respectively (Figure 6; Figure S1; Table 3). Comparable to the suitable habitat predicted by the weighted‐mean, the current IUCN range for *L. africana* across the selected countries for this modeling is 889,149.01 km^2^ (Figure S3; Tables S2 and S3). The predicted suitable habitats concentrated in southern, eastern, and northern Ethiopia, while a more fragmented distribution was exhibited in western Ethiopia. The fragmented suitable areas were observed in southeastern Eritrea and along the border between eastern Ethiopia and Somalia (Figure 6). The predicted suitable habitats indicate further concentration and continuous distribution in central, southern, and western Kenya (Figure 6). The continuous suitable habitat distributions were also observed in northern and eastern Tanzania, extending to Lake Victoria areas, particularly in central, western, and southern Uganda (Figure 6; Figure S1). Rwanda was predicted as suitable habitat for *L. africana* . However, only limited suitable areas were observed in eastern South Sudan. The predicted suitable habitats are highly restricted and fragmented in South Sudan, Eritrea, and Somalia.

**TABLE 3:** The average range size change (i.e., loss, gain, and stable) for *L. africana* under future climate conditions (2050 and 2070) using two cutoff thresholds: The weighted‐mean two thresholds (weighted mean and 10%—10 percentile omission rate).

Scenarios	Threshold	Loss km^2^	Loss %	Remain suitable km^2^	Gain km^2^	Gain %	Range change %	Current range km^2^	future range km^2^
2050	Wm	434,143.3	48.9	452,787.8	37,836.3	4.3	−44.68	886,931.1	490,624.1
	10%	557,075.5	62.8	329,850.4	32,004.9	3.6	−59.2	886,925.9	361,855.3
2070	Wm	377,622.2	50.05	376,957.2	39,118.9	5.2	−44.9	754,579.4	416,076.1
	10%	484,363.2	64.2	270,229.6	29,332.1	3.9	−60.3	754,592.8	299,561.7

### 3.4 Future Projected Habitat Suitability Modeling

**FIGURE 7:**
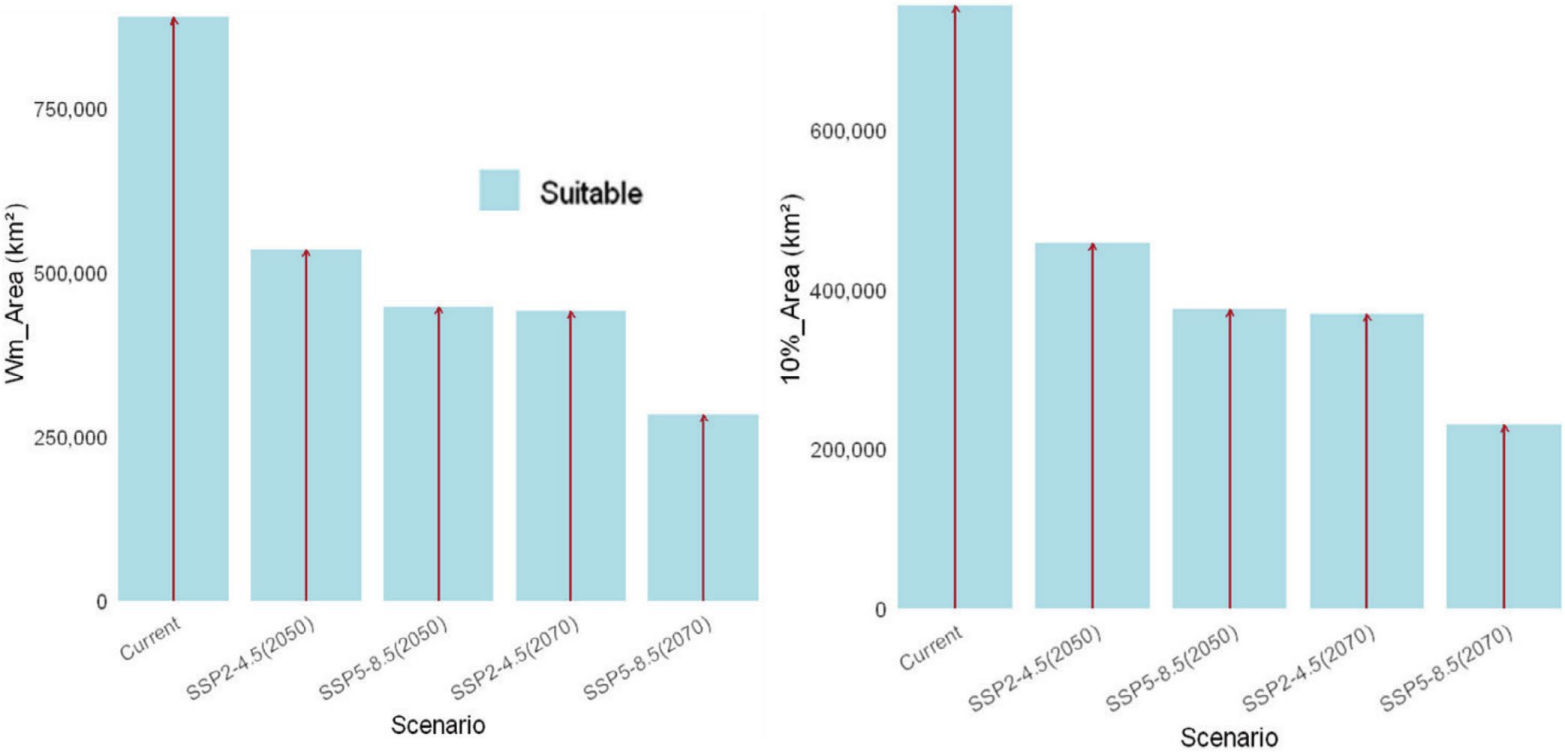
The estimated suitable areas size of *L. africana* under current and future climate emission scenarios (2050s and 2070s). An ensemble model outputs for the weighted mean (right) and the 10% omission rate (left).

**FIGURE 8:**
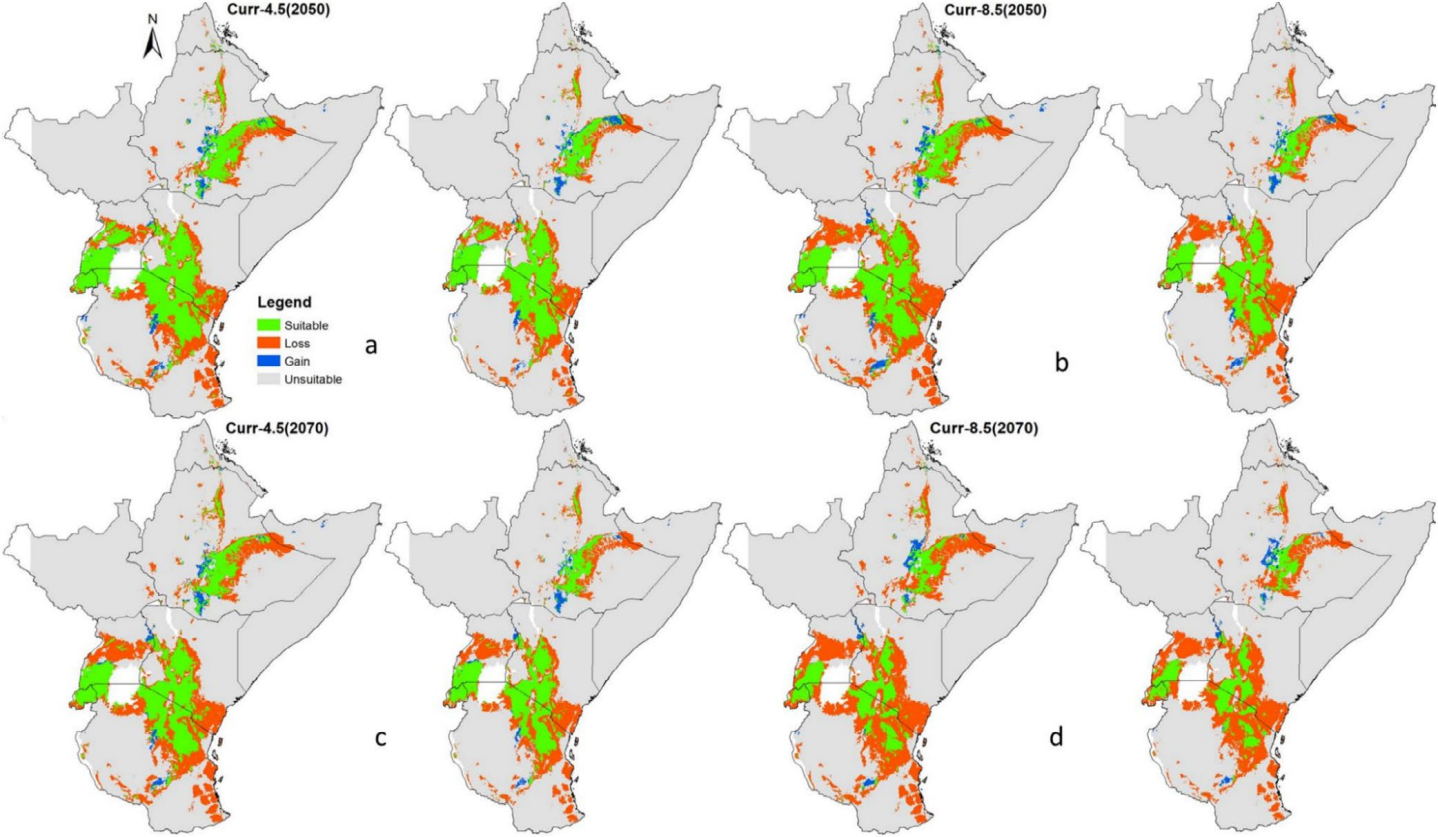
Predicted change in habitat suitability of *L. africana* by 2050 (Curr_2050) and by 2070 (curr_2070). Green, pixels that are predicted to be suitable under both current and future climates; blue, pixels that are not currently predicted to be suitable but forecasted to be suitable in the future; red, currently suitable but not in the future; and gray, unsuitable both under current and future climates. Maps were generated using two thresholds (a–d; weighted mean and 10% applied from left to right, respectively) values to evaluate the area change in each scenario.

Under future climate scenarios, the estimated average suitable areas, were projected to decline markedly from 487,145 km^2^ (54.9%) by the 2050 to 412,997 km^2^ (46.6%; 2070) under SSP2‐4.5; while from 365,334 km^2^ (41.2%; 2050) to 302,641 km^2^ (34.2%) by the 2070 under SSP2‐8.5 (Figures 6, 7, 8; Table 3; Table S2). However, in time wise the mean suitable areas projected for *L. africana* is 426,240 km^2^ (48.6%) by the 2050 and 357,819 km^2^ (40.3%) by the 2070 (Table 3; Table S1; Figure S2). Based on the threshold, the lost suitable habitats projected as high as 71.3% under the weighted‐mean worst‐case scenario for 2050 and 73% under the 10th‐percentile worst‐case scenario for 2070 (Table S2). The mean suitable areas predicted of the species were projected to decline substantially under future scenarios, by 51.9% in 2050 and 52.6% in 2070 (Figures 7 and 8; Figure S2; Table 3; Table S2).

### 3.5 Spatial Overlap of Current Predicted Suitable Habitat With PAs Networks

**FIGURE 9:**
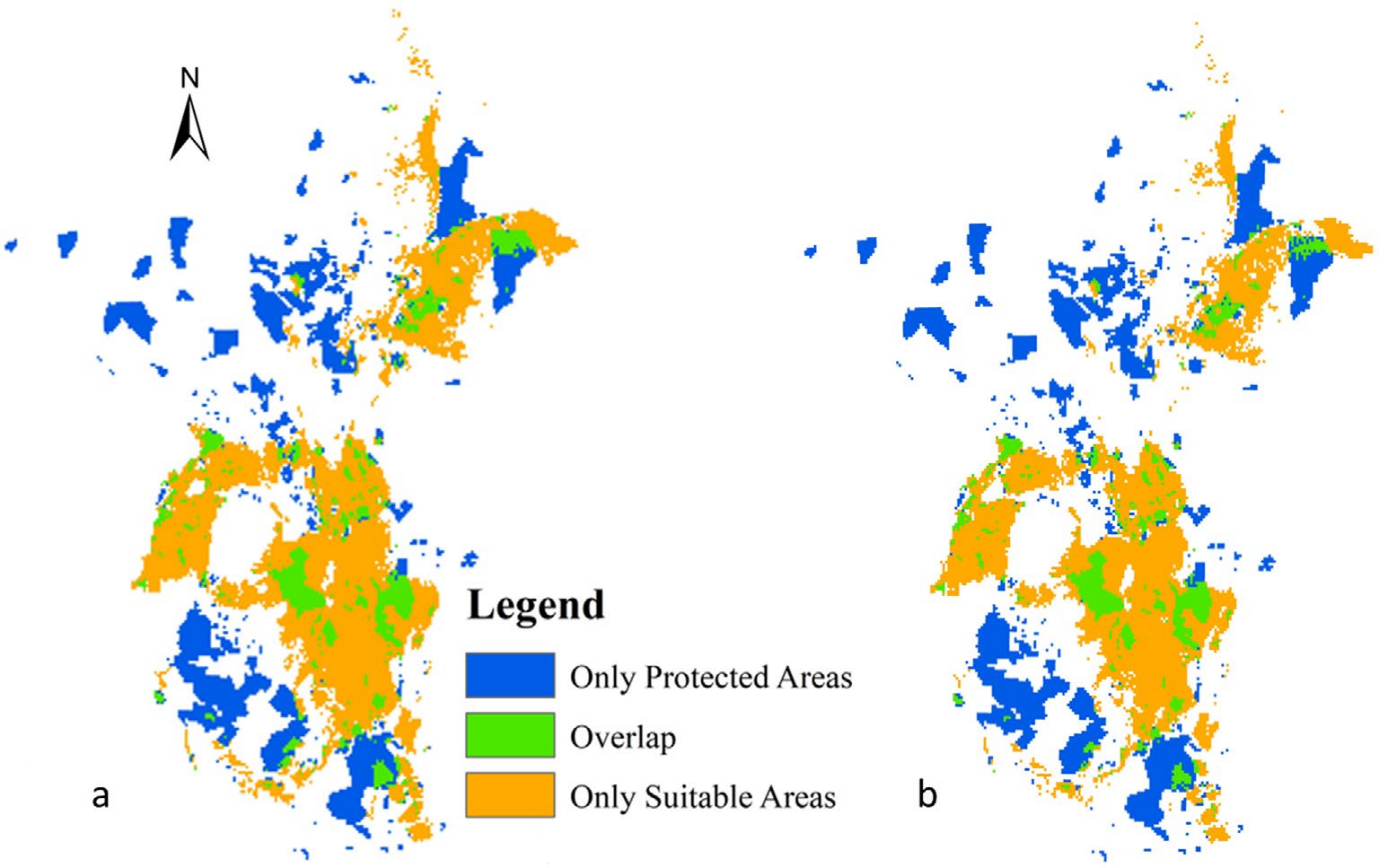
Current suitable habitats for *L. africana* in relation to protected areas networks: Only protected areas (blue), overlapping (suitable habitats in PAs) areas (green), and only suitable areas outside PAs (orange), based on two cutoff thresholds: The weighted mean (a) and the 10% percentile omission rate (b).

The size of the protected area networks in East Africa is about 573,067.4 km^2^. Only small fractions 17.2% and 17.9% of the suitable habitats were found inside PA networks, respectively for weighted mean and 10% omission thresholds (Table 4). Whereas the predicted suitable habitats outside PAs were about 82.5% (734,618.9 km^2^) for the weighted mean and 82.1% (622,252.3 km^2^) for the 10% omission threshold (Figures 8 and 9; Table 4).

**TABLE 4:** Area (km^2^) of predicted suitable habitats for *L. africana* : Only protected areas, overlapping areas, and only suitable areas.

Class	Cutoff‐threshold			%
	Area‐weighted mean	%	10% percentile omission rate	
Current scenario				
Overlap	156,214.8	17.5	135,688.1	17.9
Only suitable areas outside PAs	734,618.9	82.5	622,252.3	82.1

#### 3.5.1 Projected Stable or Remained Suitable Habitats Regarded as Anthropocene Refugia

**FIGURE 10:**
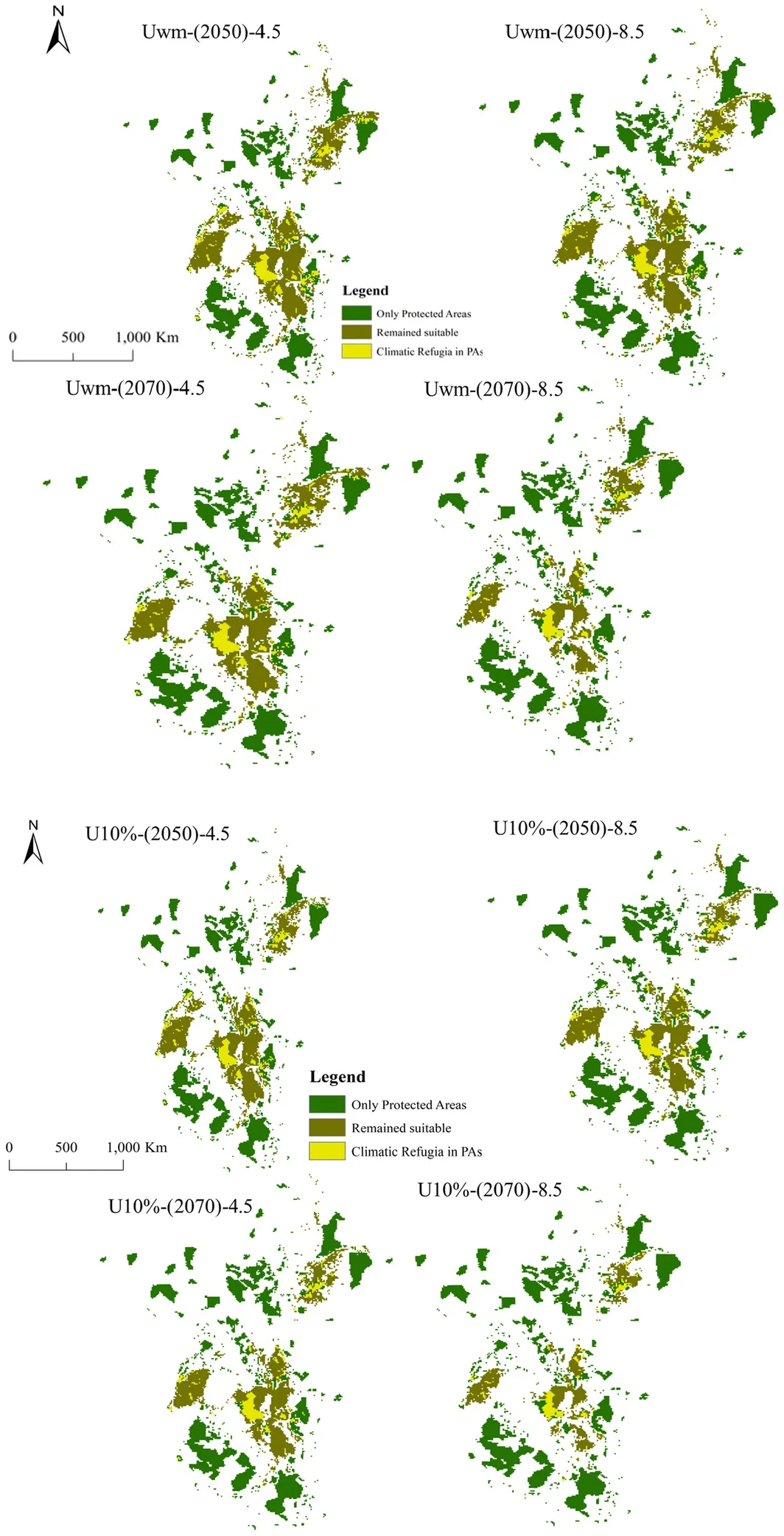
Ensemble maps of climatically suitable habitat for *L. africana* under the weighted mean and 10% percentile omission rate thresholds, showing only protected areas, remaining suitable habitats, and climatic refugia within protected areas (i.e., Anthropocene refugia) across East Africa.

Both the area thresholds of remaining suitable habitat (stable) and climatic refugia within PAs of the climatically suitable habitat of *L. africana* decline under climate change scenarios (Figures 9 and 10; Table 5). Climatic refugia estimated using the weighted mean threshold are projected to decline markedly under the most severe climate scenarios, with reductions of 21,411.5 km^2^ under the 2050–8.5 scenario compared with 2050–4.5, and 23,408.7 km^2^ under the 2070–8.5 scenario compared with 2070–4.5. Similarly, under the 10% percentile omission rate threshold, refugia are predicted to decrease by 16,938.5 km^2^ under the 2050–8.5 scenario relative to 2050–4.5, and by 18,702.9 km^2^ under the 2070–8.5 scenario compared with 2070–4.5 (Figures 9 and 10; Table 5). Notably, the predicted current suitable habitat is markedly smaller than the species' range delineated by the IUCN (Figure S3), indicating a significant reduction in potentially suitable areas (Table S3).

**TABLE 5:** Spatial extent (km^2^) of stable suitable habitats and Anthropocene refugia (remained suitable habitats within protected areas networks) for *L. africana* using two *c*utoff‐threshold area‐weighted mean and 10% percentile omission rate.

Scenarios	Area‐weighted mean			10% percentile omission rate		
	Remained suitable	Anthropocene refugia in PAs	%	Remained suitable	Anthropocene refugia in PAs	%
2050–4.5	411,419.1	88,733.0	21.6	345,778.6	74,275.8	21.5
2050–8.5	342,542.4	67,321.5	19.7	280,375.0	57,337.3	20.5
2070–4.5	338,208.2	69,002.4	20.4	280,156.6	58,434.5	20.9
2070–8.5	210,084.3	45,593.7	21.7	164,860.0	39,731.6	24.1

## 4 Discussion

We predicted the current distribution and suitable habitats of *L. africana* and assessed their overlap with East Africa's protected area network. We then projected future habitat suitability under climate change scenarios and identified climatically stable habitats within PAs that may act as Anthropocene refugia. Our ensemble models demonstrated strong predictive performance across multiple evaluation metrics, exceeding those reported in previous studies (Dejene et al. 2021).

The most influential bioclimatic drivers of *L. africana* habitat suitability were precipitation of the warmest quarter, isothermality, mean temperature of the driest quarter, and precipitation of the driest month. This finding was consistent with previous studies (Dejene et al. 2021; Jeza and Bekele 2023). These variables strongly affect habitat suitability by constraining water availability, foraging productivity or conditions and thermal stress tolerance, which directly influence elephant distribution, migration corridors, and long‐term survival under climate change (Chase et al. 2016; Bastille‐rousseau et al. 2020; Rozen‐rechels et al. 2020; Dejene et al. 2021). Furthermore, the behavioral plasticity of *L. africana* is impacted by variability in environmental conditions (Birkett et al. 2012; Thaker et al. 2019). Its reproduction (Wittemyer et al. 2007) and range change (Roberts et al. 2022) are also affected by seasonal habitat quality and climatic variability. Human footprint was less influential in predicting elephant habitat suitability at broad spatial scales, indicating that climate is the primary driver of elephant distribution. Nevertheless, habitat encroachment, agricultural expansion, roads, and poaching remain important local threats. While climate determines the overall pattern of suitable habitat, human pressures shape elephant movement and habitat use within landscapes. Evidence shows that climate change and human pressures as primary drivers of future habitat loss, with profound global risks to biodiversity, livelihoods, and well‐being (Pörtner et al. 2023; Pfenning‐butterworth et al. 2024). Human footprint and the distribution of PAs strongly influence the range of African elephants across the continent (Dejene et al. 2021; Wall et al. 2021) and Asian elephant (de Silva, Wu, Nyhus, et al. 2023). As a result, these human‐induced climate changes have profoundly impacted the study species, which is now listed as endangered due to escalating pressures (Gobush et al. 2022; Gross and Heinsohn 2023). These changes also severely threatens the Africa's biodiversity (Mosisa et al. 2024).

The current predicted suitable habitat distribution for *L. africana* have determined in compliance to the actual distribution range, except variance among countries, likely reflecting differences in the extent of *L. africana's* ecosystem range (Chase et al. 2016). The predicted suitable habitats are consistent with previous studies (Dejene et al. 2021). Under the current climate, the models predicted the difference of about 132,356.70 km^2^ between the weighted‐mean and the 10‐percentile omission rate (10%) thresholds. However, there is a comparable result obtained to the suitable habitat predicted by the weighted‐mean and the current IUCN range for *L. africana* across the selected countries, which implies habitat suitability modeling as key considering for species geographic range analysis through IUCN (Table S3; Figure S3). The findings highlight that the most concentrated and contiguous suitable habitats occur in Tanzania, Kenya, Ethiopia, and Uganda. Although different threshold methods can substantially alter and reduce the predicted suitable areas in SDMs (Norris 2014; Ahmed, Bekele, et al. 2023; Ahmed, Chala, et al. 2023), our study predicts suitable habitat ranges that extend beyond those delineated by the IUCN (Gobush et al. 2021, Gobush et al. 2022). This underscores the need for conservation measures beyond historical ranges.

For future climate scenarios, the mean suitable habitat area predicted for the species projected substantially to reduce by 51.9% in 2050 and 52.6% in 2070 compared to the current averaged suitable habitat area of 820,915.25 km^2^. The small fraction of suitable habitat, 17.2%–17.9%, lies within the existing East Africa's PAs network, currently qualifies as current Anthropocene refugia are critical for the long‐term conservation of the study species. Moreover, the ongoing expansion of agriculture, urbanization, and infrastructure, including encroachment into PAs, intensifies human‐elephant conflict and accelerates habitat loss as well as reductions in PAs (Chen et al. 2022; de Silva, Wu, Nyhus, et al. 2023). The study discovered that the majority of predicted suitable habitats (82%) fall outside existing PAs require further conservation action to be considered as refugia for the endangered species. Similarly, evidence shows that over 57% of the African elephant's range lies beyond current conservation boundaries (Wall et al. 2021). This suggests that historic PAs were larger, or that all areas were suitable due to the species' extensive home range. The unsuitability of substantial portions of PAs indicates that legal designation alone does not ensure viable habitat conditions, and PAs may ultimately fail to support sustainable elephant populations unless habitat management is improved. This shortfall expose critical conservation gaps and provide no assurance of being better preserved or more effective than unprotected landscapes (Huang et al. 2019; Li et al. 2023; Correa et al. 2024). Therefore, such key findings highlight the need for improved management, funding, and connectivity for effective conservation (Douglas‐Hamilton et al. 2005).

On average, of the projected remained suitable or stable habitats for 2050 and 2070 time periods, nearly 20.825% and 21.775% were found inside the PAs regarded as Anthropocene refugia. Reductions in the Anthropocene refugia signal shrinking safe havens for the study species, increasing their vulnerability to climate change and human‐driven habitat loss. This result is not surprising, as Eastern Africa is threatened by anthropogenic pressures affecting the geographic ranges of its iconic mammals (Tafesse 2022). Reductions in remaining suitable habitats and Anthropocene refugia, including many areas outside PAs, highlight the challenges species face from human‐driven environmental changes such as climate change, habitat loss, and other anthropogenic pressures. This suggests that strategic expansion and reconfiguration of protected area networks, the establishment of corridors, community participatory approaches, and increased awareness of animal welfare, together with strengthened wildlife policies, are essential to ensure the long‐term survival of the endangered *L. africana* in a changing Horn of Africa. Our projected Anthropocene refugia are limited and coincide with the existing PA network, yet much of the network is unsuitable and serves only as PAs. Evidence indicates that many PAs face severe anthropogenic pressures and are increasingly unsuitable for mammals, highlighting the need to consider both environmental conditions and human pressures when defining Anthropocene refugia (Hoffmann et al. 2019; Lawler et al. 2020; Parks et al. 2022).

Besides human‐induced stressors, threats on African elephants are further amplified by climate change (Achille et al. 2020). Pressures on habitat are profound (Neil and Greengrass 2022), directly threatening elephant ranges, consistent with ground evidence from the Horn of Africa, particularly Ethiopia. In the Horn of Africa, anthropogenic pressures, especially agricultural expansion, have likely pushed elephants from savannah forests and corridors toward the limits of their climatic resilience. Worst‐case scenarios predict severe loss and fragmentation of their home range, leaving only small, isolated patches outside their current occurrence areas. If African elephants currently occupy marginal habitats or remain confined to forests to avoid humans, they may function as a refugee species, undermining the topographic and climatic tolerances predicted by our ensemble models. Compounding this, continuous population monitoring is limited, corridor establishment is insufficient, and pressures are exacerbated by ethnic conflicts and the lack of local community stewardship over PAs and African elephants (Breuer et al. 2016). We therefore recommend protecting their current habitats even if forecasted to become climatically unsuitable, especially for populations in Eritrea, Somalia, and South Sudan within the Horn of Africa. Given the escalating impacts of human‐induced climate change on species distributions, modeling its effects on the future distribution of refugia should be a priority for follow‐up studies (Keppel et al. 2015; Kufa et al. 2025). Effective conservation and rehabilitation programs rely on understanding anthropogenic pressures, climate trends, and species distribution ranges. The concept of Anthropocene refugia, which links historical biogeography with current threats, further underscores the need for refined modeling to predict species survival under climate change (Monsarrat, Jarvie, et al. 2019; Monsarrat, Novellie, et al. 2019) and to assess the space available for biodiversity in human‐dominated landscapes.

## 5 Conclusion

Our species distribution modeling demonstrates that the current suitable habitat of *L. africana* is vulnerable to climate change. *Loxodonta africana* is projected to lose substantial portions of its suitable habitat across the Horn of Africa, particularly in South Sudan and Somalia, where suitable areas are likely to become unsuitable entirely. Additionally, identifying climatically suitable habitats and Anthropocene refugia within PAs is a crucial first step and long‐term goal for monitoring *L. africana* distributions and prioritizing sites for conservation under severe anthropogenic pressures. Our models reveal a clear contraction of suitable habitat, including remaining suitable areas and Anthropocene refugia, with substantial historical losses of suitable habitat evident, likely driven by anthropogenic pressures in combination with climate change. Moreover, suitable habitats occur outside PAs, while many existing PAs are unsuitable for the species. These findings support the need to reassess and realign protected‐area boundaries to ensure that future suitable habitats and climate refugia are effectively conserved for this species with limited ranges. In particular, populations currently surviving in unsuitable habitats, despite their documented presence, will be at severe risk. Overall, our findings reveal a critical situation: neither the suitable habitats inside PAs nor those outside them can guarantee the long‐term survival of the species. We recommend urgent ground‐based reassessments, clear demarcation of PAs, strengthened community engagement and stewardship, and the establishment of transboundary landscape corridors to enhance connectivity and improve the long‐term survival prospects of the remaining populations.

## Author Contributions

**Ahmed Seid Ahmed:** conceptualization (equal), data curation (equal), formal analysis (equal), investigation (equal), methodology (equal), project administration (equal), resources (equal), software (equal), supervision (equal), validation (equal), visualization (equal), writing – original draft (equal), writing – review and editing (equal). **Daniel Melese:** conceptualization (equal), formal analysis (equal), investigation (equal), methodology (equal), project administration (equal), software (equal), validation (equal), visualization (equal), writing – review and editing (equal). **Mulatu Ayenew Aligaz:** validation (equal), writing – review and editing (equal). **Anagaw Atickm:** supervision (equal), writing – review and editing (equal). **Chala Adugna Kufa:** conceptualization (equal), formal analysis (equal), investigation (equal), methodology (equal), software (equal), validation (equal), visualization (equal), writing – original draft (equal), writing – review and editing (equal).

## Funding

The authors have nothing to report.

## Conflicts of Interest

The authors declare no conflicts of interest.

## Supporting information


**Table S1:** ece374389‐sup‐0001‐DataS1.docx. *Loxodonta* *Africana* occurrence points and selected environmental variables used for the final model building.
**Table S2:** Projected area (km^2^) of suitable habitat, including gains and losses, for *L. africana* under future climate scenarios (2050 and 2070) using weighted mean and 10% omission rate thresholds.
**Table S3:** Current range of IUCN, suitable area predicted by weighted mean (WM) and 10 percentiles, and its average value.
**Figure S1:** Predicted change in habitat suitability of *L. africana* by 2050s and by 2070s. Green, pixels that are predicted to be suitable under both current and future climates; blue, pixels that are not currently predicted to be suitable but forecasted to be suitable in the future; red, currently suitable but not in the future; and gray, unsuitable both under current and future climates. Maps were generated using two thresholds (weighted mean and 10%—10 percentile omission rate, applied from right to left, respectively) values to evaluate the area change in each scenario.
**Figure S2:** Mean of weighted mean and 10%—10 percentile omission rate threshold suitable areas size of *L. africana* under current and each future climate emission scenarios (2050s and 2070s). An ensemble model outputs for the weighted mean (right) and the 10% omission rate (left).
**Figure S3:** Current IUCN Range of *L. africana* across the east African countries (*Source:* Gobush et al. 2022; the range is summed from Extant (Resident), possibly extant (resident) and extant and reintroduced (resident)).

## Data Availability

All relevant data are provided in the Supporting Information, including the species occurrence records, the raw data used for the final modeling, and the figures and tables referenced in the Supporting Information. Additionally, metadata describing the environmental variables, including their measurement units, abbreviations, and associated raw data, are openly accessible via Zenodo at: https://doi.org/10.5281/zenodo.22286651. The R scripts used for data processing, modeling, and analysis are available from the corresponding author upon reasonable request.
