# Supplementary material for: Persistence Vulnerability of the African Savanna Elephant Loxodonta africana to Anthropocene Threats in East Africa
Source: Ecol Evol. 2026 Sep 24;16(9):e74389. doi: 10.1002/ece3.74389 (PMC13612745; doi:10.1002/ece3.74389)
Supplement: Supplementary file 1 [file ECE3-16-e74389-s001.docx]

**Persistence Vulnerability of the African Savanna Elephant *Loxodonta africana* to Anthropocene Threats in East Africa**

**TABLE S1** L. Africana occurrence points and selected environmental variables used for the final model building

| No | Long | Lat | Source | Bio2 | Bio3 | Bio8 | Bio9 | Bio13 | Bio14 | Bio15 | Bio18 | Bio19 | Footprint |
| --- | --- | --- | --- | --- | --- | --- | --- | --- | --- | --- | --- | --- | --- |
| 1 | 42.25 | 9.14 | GBIF | 13.94 | 74.55 | 22.90 | 21.22 | 87 | 6 | 59.90 | 211 | 31 | 13 |
| 2 | 36.39 | 5.48 | GBIF | 12.89 | 78.13 | 23.57 | 24.07 | 156 | 41 | 38.08 | 179 | 273 | 3 |
| 3 | 36.25 | 7.23 | GBIF | 15.04 | 80.01 | 19.72 | 20.33 | 221 | 43 | 42.36 | 405 | 628 | 10 |
| 4 | 35.76 | 5.86 | f. survey | 13.84 | 79.10 | 23.32 | 24.45 | 186 | 46 | 38.77 | 203 | 424 | 2 |
| 5 | 35.75 | 5.85 | f. survey | 13.88 | 78.84 | 23.37 | 24.52 | 187 | 46 | 39.01 | 203 | 424 | 2 |
| 6 | 36.28 | 5.84 | f. survey | 13.21 | 77.70 | 22.00 | 23.10 | 166 | 43 | 38.23 | 180 | 338 | 13 |
| 7 | 36.42 | 5.67 | f. survey | 12.96 | 77.59 | 22.15 | 23.30 | 153 | 40 | 38.44 | 169 | 284 | 5 |
| 8 | 36.35 | 5.77 | f. survey | 13.41 | 77.96 | 22.22 | 23.35 | 158 | 41 | 38.18 | 173 | 310 | 7 |
| 9 | 36.42 | 5.68 | f. survey | 12.94 | 77.96 | 21.92 | 23.07 | 156 | 41 | 38.22 | 173 | 290 | 6 |
| 10 | 36.45 | 5.78 | f. survey | 12.64 | 77.56 | 20.83 | 22.05 | 179 | 42 | 38.61 | 202 | 330 | 10 |
| 11 | 36.42 | 5.37 | f. survey | 12.72 | 77.54 | 23.45 | 24.02 | 154 | 41 | 37.73 | 183 | 254 | 3 |
| 12 | 35.93 | 5.32 | f. survey | 14.83 | 82.87 | 26.13 | 26.65 | 181 | 48 | 38.77 | 202 | 351 | 6 |
| 13 | 36.43 | 5.32 | f. survey | 12.61 | 77.35 | 23.55 | 24.12 | 152 | 40 | 38.17 | 181 | 241 | 3 |
| 14 | 35.33 | 5.25 | f. survey | 13.88 | 82.59 | 25.97 | 26.32 | 161 | 36 | 39.47 | 180 | 306 | 11 |
| 15 | 36.58 | 6.67 | Literature | 13.84 | 75.64 | 19.62 | 20.47 | 185 | 41 | 42.58 | 191 | 486 | 6 |
| 16 | 35.33 | 5.50 | Literature | 12.62 | 80.88 | 21.83 | 22.18 | 184 | 42 | 37.24 | 228 | 384 | 3 |
| 17 | 36.00 | 6.67 | Literature | 13.71 | 78.33 | 20.98 | 21.82 | 223 | 48 | 40.10 | 235 | 566 | 3 |
| 18 | 36.45 | 6.93 | Literature | 13.83 | 78.15 | 20.88 | 21.28 | 222 | 45 | 42.10 | 421 | 593 | 6 |
| 19 | 36.95 | 7.13 | Literature | 13.69 | 76.92 | 18.70 | 19.95 | 204 | 30 | 52.20 | 314 | 588 | 6 |
| 20 | 35.93 | 5.48 | Literature | 14.14 | 81.27 | 24.77 | 25.92 | 177 | 47 | 38.71 | 196 | 360 | 3 |
| 21 | 35.93 | 6.58 | Literature | 13.70 | 78.29 | 21.13 | 21.97 | 209 | 46 | 40.51 | 217 | 536 | 3 |
| 22 | 42.02 | 8.38 | Literature | 14.19 | 77.55 | 23.77 | 22.27 | 98 | 5 | 60.72 | 229 | 34 | 2 |
| 23 | 43.10 | 9.01 | Literature | 15.24 | 73.99 | 21.62 | 18.72 | 91 | 4 | 68.53 | 229 | 26 | 3 |
| 24 | 42.00 | 8.42 | f. survey | 14.39 | 77.79 | 23.65 | 22.15 | 98 | 5 | 59.88 | 229 | 34 | 2 |
| 25 | 43.12 | 9.13 | f. survey | 15.43 | 73.49 | 20.68 | 17.48 | 98 | 4 | 70.72 | 236 | 26 | 4 |
| 26 | 42.42 | 9.15 | f. survey | 13.00 | 73.45 | 21.33 | 19.50 | 92 | 6 | 61.54 | 228 | 32 | 4 |
| 27 | 42.28 | 9.06 | f. survey | 13.16 | 73.92 | 23.17 | 21.37 | 80 | 6 | 58.94 | 206 | 30 | 12 |
| 28 | 42.27 | 9.02 | f. survey | 13.63 | 74.50 | 23.22 | 21.55 | 80 | 5 | 58.81 | 208 | 29 | 6 |
| 29 | 35.63 | 12.50 | f. survey | 15.18 | 66.30 | 26.62 | 27.37 | 235 | 0 | 121.92 | 75 | 590 | 3 |
| 30 | 35.67 | 12.20 | f. survey | 15.23 | 66.23 | 26.20 | 27.13 | 247 | 0 | 119.44 | 88 | 638 | 2 |
| 31 | 35.73 | 12.38 | f. survey | 15.22 | 66.74 | 26.50 | 27.33 | 244 | 0 | 120.64 | 83 | 618 | 9 |
| 32 | 35.77 | 12.30 | f. survey | 15.03 | 66.81 | 26.35 | 27.17 | 250 | 0 | 119.89 | 87 | 637 | 9 |
| 33 | 35.47 | 12.15 | Literature | 15.49 | 66.77 | 26.30 | 27.25 | 241 | 0 | 119.29 | 85 | 623 | 2 |
| 34 | 35.42 | 12.09 | Literature | 15.59 | 66.35 | 26.33 | 27.22 | 239 | 0 | 119.03 | 85 | 622 | 2 |
| 35 | 37.21 | 14.20 | Literature | 14.09 | 65.85 | 25.02 | 26.05 | 197 | 0 | 136.69 | 46 | 486 | 5 |
| 36 | 36.70 | 14.08 | Literature | 15.56 | 68.24 | 26.22 | 27.13 | 194 | 0 | 134.87 | 45 | 484 | 5 |
| 37 | 37.65 | 14.45 | Literature | 15.23 | 67.40 | 24.80 | 25.33 | 207 | 0 | 136.25 | 131 | 484 | 7 |
| 38 | 34.25 | 8.00 | Literature | 13.47 | 70.18 | 25.25 | 27.13 | 197 | 4 | 80.17 | 89 | 490 | 2 |
| 39 | 36.67 | 6.99 | f. survey | 14.03 | 76.27 | 20.18 | 21.30 | 204 | 41 | 45.97 | 362 | 585 | 5 |
| 40 | 36.77 | 6.99 | f. survey | 14.39 | 74.18 | 19.80 | 21.42 | 194 | 38 | 47.04 | 320 | 541 | 5 |
| 41 | 36.67 | 7.03 | f. survey | 14.25 | 76.20 | 19.90 | 21.33 | 207 | 42 | 45.67 | 364 | 594 | 5 |
| 42 | 36.68 | 7.03 | f. survey | 14.26 | 76.25 | 19.88 | 21.30 | 206 | 41 | 45.93 | 359 | 588 | 5 |
| 43 | 36.70 | 7.01 | f. survey | 14.19 | 75.89 | 19.85 | 21.32 | 203 | 41 | 45.89 | 353 | 578 | 5 |
| 44 | 36.71 | 7.00 | f. survey | 14.24 | 75.75 | 20.27 | 21.35 | 202 | 41 | 45.94 | 354 | 573 | 5 |
| 45 | 36.72 | 7.00 | f. survey | 14.25 | 75.40 | 19.88 | 21.33 | 201 | 40 | 46.29 | 346 | 569 | 5 |
| 46 | 36.67 | 6.96 | f. survey | 13.93 | 76.56 | 19.98 | 20.97 | 203 | 42 | 44.99 | 380 | 579 | 5 |
| 47 | 36.67 | 6.98 | f. survey | 13.98 | 76.83 | 20.08 | 21.05 | 204 | 42 | 45.20 | 377 | 584 | 5 |
| 48 | 36.75 | 6.97 | f. survey | 14.31 | 74.52 | 19.63 | 21.28 | 190 | 38 | 46.74 | 312 | 527 | 5 |
| 49 | 42.50 | 8.60 | f. survey | 14.05 | 75.54 | 22.53 | 20.82 | 99 | 5 | 62.27 | 229 | 32 | 5 |

| 50 | 36.70 | 6.90 | f. survey | 14.08 | 75.31 | 20.28 | 21.53 | 194 | 41 | 44.74 | 360 | 552 | 5 |
| --- | --- | --- | --- | --- | --- | --- | --- | --- | --- | --- | --- | --- | --- |
| 51 | 35.10 | 10.60 | Literature | 13.49 | 66.46 | 24.52 | 26.95 | 232 | 0 | 102.73 | 144 | 639 | 6 |
| 52 | 34.10 | 7.60 | Literature | 13.76 | 68.45 | 25.27 | 26.57 | 184 | 8 | 72.20 | 114 | 490 | 0 |
| 53 | 40.10 | 4.50 | Literature | 11.28 | 72.74 | 22.75 | 20.70 | 137 | 3 | 115.25 | 54 | 10 | 2 |
| 54 | 37.20 | 14.20 | Literature | 14.16 | 66.16 | 25.17 | 26.20 | 195 | 0 | 136.36 | 46 | 481 | 5 |
| 55 | 36.20 | 5.60 | Literature | 13.85 | 79.14 | 23.72 | 24.87 | 162 | 43 | 38.10 | 179 | 314 | 3 |
| 56 | 35.70 | 7.40 | Literature | 13.43 | 82.87 | 16.13 | 16.77 | 223 | 38 | 50.65 | 477 | 618 | 6 |
| 57 | 35.70 | 5.70 | f. survey | 13.65 | 80.29 | 23.03 | 24.22 | 199 | 49 | 38.03 | 225 | 434 | 3 |
| 58 | 36.00 | 12.40 | Literature | 15.08 | 67.64 | 25.97 | 26.85 | 258 | 0 | 119.63 | 92 | 651 | 9 |
| 59 | 42.40 | 8.70 | f. survey | 14.83 | 76.86 | 21.82 | 20.32 | 100 | 5 | 61.14 | 232 | 33 | 8 |
| 60 | 38.90 | 4.20 | Literature | 10.69 | 68.98 | 21.62 | 19.40 | 164 | 7 | 96.98 | 102 | 28 | 4 |
| 61 | 36.80 | 6.90 | Literature | 13.93 | 74.87 | 19.93 | 21.22 | 187 | 38 | 46.31 | 326 | 525 | 6 |
| 62 | 35.10 | 10.50 | Literature | 13.82 | 67.11 | 24.70 | 26.83 | 234 | 1 | 101.11 | 49 | 644 | 10 |
| 63 | 34.00 | 8.00 | Literature | 13.08 | 71.84 | 25.35 | 27.00 | 182 | 4 | 80.65 | 82 | 483 | 3 |
| 64 | 35.80 | 5.80 | f. survey | 14.43 | 79.30 | 23.77 | 24.95 | 186 | 46 | 38.67 | 201 | 410 | 2 |
| 65 | 35.80 | 12.30 | Literature | 14.98 | 67.15 | 26.08 | 26.93 | 256 | 0 | 119.82 | 91 | 653 | 9 |
| 66 | 37.80 | 14.30 | Literature | 14.63 | 66.21 | 24.70 | 25.43 | 202 | 1 | 132.83 | 133 | 470 | 5 |
| 67 | 36.00 | 5.80 | f. survey | 13.42 | 78.46 | 22.93 | 23.98 | 181 | 46 | 37.91 | 198 | 388 | 2 |
| 68 | 33.90 | 8.00 | Literature | 13.22 | 71.49 | 25.43 | 27.20 | 177 | 4 | 80.65 | 80 | 489 | 2 |
| 69 | 42.30 | 8.70 | Literature | 12.90 | 75.44 | 23.67 | 21.77 | 86 | 5 | 58.80 | 207 | 30 | 5 |
| 70 | 36.00 | 5.90 | Literature | 13.42 | 78.00 | 22.65 | 23.65 | 180 | 45 | 38.39 | 195 | 394 | 2 |
| 71 | 34.90 | 7.20 | Literature | 13.59 | 74.68 | 23.33 | 24.12 | 200 | 24 | 55.28 | 213 | 358 | 0 |
| 72 | 36.70 | 7.00 | Literature | 14.18 | 75.40 | 20.18 | 21.30 | 201 | 40 | 46.33 | 345 | 568 | 5 |
| 73 | 42.50 | 8.70 | Literature | 14.18 | 75.44 | 22.37 | 20.55 | 96 | 5 | 61.78 | 225 | 31 | 5 |
| 74 | 33.90 | 8.10 | Literature | 13.22 | 71.44 | 25.47 | 27.42 | 175 | 3 | 82.75 | 75 | 480 | 4 |
| 75 | 36.70 | 14.05 | Literature | 14.88 | 67.31 | 26.03 | 26.98 | 197 | 0 | 134.94 | 46 | 490 | 4 |
| 76 | 37.68 | 14.46 | Literature | 15.18 | 67.44 | 24.77 | 25.23 | 209 | 0 | 135.90 | 135 | 488 | 7 |
| 77 | 37.30 | 14.80 | GBIF | 15.40 | 69.06 | 25.68 | 25.52 | 177 | 0 | 141.75 | 98 | 0 | 9 |
| 78 | 36.86 | 15.07 | Literature | 15.29 | 65.63 | 27.03 | 26.55 | 139 | 0 | 143.10 | 63 | 0 | 7 |
| 79 | 36.85 | 15.06 | Literature | 15.35 | 65.88 | 27.02 | 26.58 | 140 | 0 | 143.11 | 64 | 0 | 7 |
| 80 | 36.89 | 14.94 | Literature | 14.88 | 66.70 | 26.10 | 25.22 | 160 | 0 | 142.83 | 80 | 0 | 7 |
| 81 | 36.70 | 0.40 | Literature | 15.36 | 81.26 | 18.80 | 17.92 | 131 | 19 | 52.36 | 276 | 173 | 6 |
| 82 | 36.70 | 0.60 | Literature | 15.11 | 83.01 | 18.18 | 17.63 | 121 | 17 | 54.38 | 256 | 167 | 6 |
| 83 | 36.60 | 0.40 | Literature | 15.44 | 81.27 | 18.07 | 17.57 | 105 | 19 | 47.88 | 221 | 178 | 6 |
| 84 | 37.30 | 2.50 | Literature | 11.82 | 80.39 | 27.22 | 25.42 | 65 | 1 | 99.50 | 104 | 7 | 4 |
| 85 | 39.90 | 3.30 | Literature | 10.78 | 69.97 | 26.03 | 23.60 | 113 | 1 | 117.93 | 45 | 5 | 13 |
| 86 | 37.30 | 1.00 | Literature | 12.91 | 83.82 | 21.58 | 20.02 | 194 | 6 | 95.67 | 314 | 22 | 14 |
| 87 | 36.80 | 1.10 | Literature | 11.19 | 79.94 | 15.13 | 14.63 | 106 | 20 | 45.84 | 230 | 199 | 4 |
| 88 | 36.90 | 2.40 | Literature | 12.65 | 83.22 | 26.00 | 24.27 | 67 | 2 | 84.92 | 113 | 19 | 1 |
| 89 | 35.60 | 1.30 | Literature | 12.88 | 83.66 | 20.32 | 21.32 | 131 | 18 | 57.15 | 210 | 303 | 14 |
| 90 | 35.90 | 1.10 | Literature | 13.14 | 83.70 | 21.33 | 21.87 | 101 | 16 | 55.89 | 155 | 227 | 5 |
| 91 | 34.90 | 1.20 | Literature | 13.45 | 80.06 | 17.68 | 19.35 | 149 | 19 | 51.63 | 116 | 374 | 18 |
| 92 | 38.00 | 2.30 | Literature | 8.63 | 68.45 | 21.48 | 19.48 | 167 | 5 | 99.79 | 110 | 21 | 7 |
| 93 | 35.50 | 0.50 | Literature | 11.46 | 82.43 | 15.35 | 15.02 | 213 | 40 | 46.47 | 355 | 359 | 8 |
| 94 | 38.70 | 0.20 | Literature | 10.73 | 76.61 | 25.50 | 23.88 | 141 | 0 | 131.26 | 152 | 0 | 3 |
| 95 | 37.60 | 0.10 | Literature | 12.02 | 79.06 | 18.00 | 16.50 | 390 | 19 | 95.69 | 561 | 66 | 15 |
| 96 | 38.40 | 0.10 | Literature | 10.42 | 76.03 | 25.27 | 23.50 | 145 | 0 | 128.32 | 164 | 0 | 2 |
| 97 | 34.60 | 1.00 | Literature | 10.62 | 78.64 | 11.35 | 13.03 | 238 | 41 | 46.12 | 196 | 561 | 0 |
| 98 | 37.40 | 0.20 | Literature | 13.52 | 82.98 | 17.97 | 17.17 | 251 | 18 | 89.36 | 353 | 60 | 9 |
| 99 | 39.40 | 4.30 | Literature | 10.85 | 70.45 | 23.30 | 21.12 | 145 | 5 | 108.37 | 72 | 16 | 2 |

| 100 | 38.50 | 2.90 | Literature | 9.81 | 69.56 | 26.58 | 24.17 | 103 | 1 | 110.74 | 60 | 4 | 2 |
| --- | --- | --- | --- | --- | --- | --- | --- | --- | --- | --- | --- | --- | --- |
| 101 | 37.64 | -3.41 | Literature | 11.60 | 69.46 | 23.82 | 21.03 | 137 | 6 | 81.80 | 176 | 30 | 26 |
| 102 | 38.57 | -2.87 | Literature | 10.55 | 70.33 | 25.68 | 22.67 | 209 | 21 | 76.82 | 360 | 79 | 2 |
| 103 | 37.07 | 0.02 | Literature | 14.67 | 79.71 | 16.92 | 16.10 | 135 | 25 | 52.34 | 225 | 150 | 30 |
| 104 | 37.86 | -2.94 | Literature | 12.49 | 70.18 | 23.75 | 20.70 | 150 | 3 | 94.68 | 150 | 14 | 6 |
| 105 | 37.29 | -2.77 | Literature | 12.88 | 72.33 | 21.97 | 19.32 | 187 | 10 | 82.26 | 196 | 42 | 4 |
| 106 | 37.26 | -2.78 | Literature | 12.53 | 71.62 | 21.60 | 18.97 | 184 | 11 | 80.46 | 200 | 46 | 4 |
| 107 | 37.74 | 1.13 | Literature | 12.10 | 82.31 | 25.02 | 23.47 | 112 | 1 | 110.51 | 179 | 6 | 3 |
| 108 | 37.49 | 0.47 | Literature | 12.69 | 82.95 | 20.92 | 20.57 | 230 | 8 | 97.44 | 337 | 27 | 6 |
| 109 | 36.91 | 0.24 | Literature | 14.66 | 79.66 | 18.52 | 17.57 | 158 | 20 | 58.37 | 328 | 160 | 10 |
| 110 | 37.90 | 3.70 | Literature | 11.30 | 71.52 | 24.12 | 21.80 | 101 | 4 | 87.34 | 88 | 16 | 1 |
| 111 | 37.33 | -0.17 | Literature | 11.40 | 77.55 | 4.15 | 3.17 | 238 | 66 | 45.86 | 478 | 251 | 1 |
| 112 | 36.63 | -0.42 | Literature | 10.50 | 77.21 | 9.20 | 9.10 | 197 | 44 | 33.92 | 385 | 375 | 6 |
| 113 | 40.52 | -2.53 | Literature | 6.63 | 66.25 | 26.97 | 27.80 | 324 | 7 | 99.23 | 168 | 169 | 12 |
| 114 | 39.63 | -0.47 | Literature | 10.01 | 72.00 | 28.85 | 27.07 | 90 | 4 | 99.08 | 117 | 18 | 29 |
| 115 | 37.65 | 0.05 | GBIF | 11.16 | 75.91 | 18.98 | 17.28 | 400 | 14 | 100.29 | 533 | 51 | 31 |
| 116 | 37.44 | 0.48 | GBIF | 14.13 | 84.63 | 22.88 | 22.77 | 156 | 7 | 90.49 | 253 | 248 | 7 |
| 117 | 35.06 | -1.55 | GBIF | 12.68 | 81.30 | 20.63 | 19.18 | 153 | 27 | 44.09 | 292 | 124 | 12 |
| 118 | 35.13 | -1.53 | GBIF | 12.73 | 81.10 | 20.20 | 18.68 | 162 | 27 | 46.39 | 296 | 123 | 2 |
| 119 | 35.10 | -1.48 | GBIF | 12.85 | 81.33 | 20.38 | 18.88 | 156 | 27 | 44.46 | 369 | 128 | 2 |
| 120 | 37.43 | 0.47 | GBIF | 14.15 | 84.73 | 22.88 | 22.73 | 158 | 8 | 90.26 | 253 | 251 | 11 |
| 121 | 36.66 | 0.59 | GBIF | 15.25 | 82.88 | 18.38 | 17.85 | 122 | 17 | 53.17 | 260 | 178 | 5 |
| 122 | 37.95 | -0.05 | GBIF | 11.56 | 76.04 | 23.57 | 21.92 | 185 | 1 | 114.90 | 248 | 6 | 11 |
| 123 | 35.23 | -1.55 | GBIF | 12.70 | 80.89 | 20.15 | 18.58 | 160 | 24 | 49.35 | 367 | 111 | 10 |
| 124 | 36.32 | -0.92 | GBIF | 14.06 | 76.40 | 17.73 | 15.43 | 142 | 28 | 55.85 | 146 | 108 | 8 |
| 125 | 35.23 | -1.52 | GBIF | 12.78 | 81.42 | 20.20 | 18.60 | 159 | 25 | 49.62 | 365 | 109 | 3 |
| 126 | 35.35 | -1.53 | GBIF | 12.25 | 80.59 | 19.48 | 17.77 | 173 | 25 | 52.16 | 387 | 107 | 5 |
| 127 | 35.28 | -1.46 | GBIF | 12.94 | 81.39 | 20.72 | 18.75 | 149 | 24 | 48.20 | 351 | 107 | 3 |
| 128 | 35.12 | -1.47 | GBIF | 12.94 | 81.39 | 20.43 | 18.90 | 155 | 27 | 44.77 | 368 | 126 | 2 |
| 129 | 35.26 | -1.45 | GBIF | 12.96 | 82.01 | 20.65 | 18.70 | 151 | 25 | 47.55 | 355 | 111 | 4 |
| 130 | 35.22 | -1.42 | GBIF | 12.87 | 81.43 | 20.47 | 18.83 | 155 | 26 | 46.02 | 361 | 121 | 11 |
| 131 | 35.32 | -1.30 | GBIF | 12.96 | 82.54 | 19.73 | 17.82 | 162 | 26 | 48.24 | 372 | 118 | 3 |
| 132 | 35.23 | -1.49 | GBIF | 12.89 | 81.59 | 20.20 | 18.62 | 153 | 25 | 47.86 | 359 | 111 | 6 |
| 133 | 35.03 | -1.48 | GBIF | 12.68 | 81.30 | 20.77 | 19.28 | 156 | 29 | 41.76 | 294 | 138 | 5 |
| 134 | 36.91 | 0.17 | GBIF | 15.13 | 78.78 | 18.13 | 17.15 | 140 | 19 | 54.01 | 297 | 164 | 3 |
| 135 | 37.51 | 0.61 | GBIF | 13.14 | 83.18 | 23.13 | 22.72 | 132 | 5 | 96.45 | 197 | 17 | 7 |
| 136 | 35.29 | -1.29 | GBIF | 13.00 | 82.80 | 19.78 | 17.88 | 163 | 27 | 47.19 | 375 | 124 | 3 |
| 137 | 37.52 | 0.73 | GBIF | 12.50 | 83.89 | 23.58 | 22.10 | 136 | 4 | 99.53 | 212 | 15 | 2 |
| 138 | 34.81 | -1.47 | GBIF | 12.51 | 82.29 | 21.23 | 19.88 | 181 | 34 | 41.38 | 304 | 156 | 8 |
| 139 | 35.31 | -1.48 | GBIF | 12.77 | 81.32 | 20.57 | 18.48 | 150 | 22 | 50.71 | 351 | 97 | 2 |
| 140 | 37.55 | 0.61 | GBIF | 13.06 | 83.71 | 23.15 | 22.70 | 132 | 4 | 98.87 | 190 | 15 | 10 |
| 141 | 35.30 | -1.47 | GBIF | 12.87 | 81.43 | 20.73 | 18.78 | 151 | 23 | 50.10 | 351 | 102 | 2 |
| 142 | 35.38 | -1.41 | GBIF | 12.98 | 81.66 | 19.93 | 17.93 | 154 | 23 | 50.93 | 359 | 101 | 3 |
| 143 | 34.82 | -1.56 | GBIF | 12.21 | 80.85 | 21.40 | 19.93 | 177 | 30 | 43.05 | 303 | 144 | 8 |
| 144 | 37.81 | -2.86 | GBIF | 12.18 | 69.62 | 23.13 | 20.25 | 166 | 4 | 94.26 | 167 | 18 | 6 |
| 145 | 35.15 | -1.43 | GBIF | 12.92 | 81.75 | 20.58 | 18.98 | 156 | 28 | 44.98 | 365 | 126 | 2 |
| 146 | 35.01 | -1.28 | GBIF | 12.52 | 81.81 | 20.20 | 18.72 | 176 | 37 | 38.93 | 300 | 173 | 5 |
| 147 | 35.39 | -1.32 | GBIF | 12.71 | 81.99 | 19.38 | 17.40 | 159 | 26 | 50.05 | 365 | 108 | 3 |
| 148 | 35.22 | -1.45 | GBIF | 12.97 | 81.60 | 20.50 | 18.88 | 151 | 25 | 46.49 | 356 | 116 | 11 |
| 149 | 35.36 | -1.60 | GBIF | 12.22 | 80.91 | 18.78 | 16.95 | 178 | 24 | 54.65 | 402 | 99 | 8 |

| 150 | 37.42 | 0.46 | GBIF | 14.20 | 85.03 | 22.83 | 22.72 | 160 | 8 | 89.33 | 255 | 254 | 15 |
| --- | --- | --- | --- | --- | --- | --- | --- | --- | --- | --- | --- | --- | --- |
| 151 | 35.19 | -1.39 | GBIF | 12.88 | 82.01 | 20.27 | 18.68 | 156 | 27 | 44.96 | 366 | 128 | 4 |
| 152 | 37.46 | 0.71 | GBIF | 12.32 | 83.28 | 21.98 | 20.52 | 197 | 6 | 97.68 | 324 | 20 | 3 |
| 153 | 35.10 | -1.54 | GBIF | 12.73 | 81.10 | 20.40 | 18.70 | 158 | 28 | 44.94 | 293 | 123 | 2 |
| 154 | 35.02 | -1.37 | GBIF | 12.78 | 81.94 | 20.45 | 18.95 | 167 | 33 | 40.64 | 297 | 153 | 3 |
| 155 | 37.55 | 0.63 | GBIF | 12.97 | 83.71 | 23.13 | 22.65 | 131 | 4 | 98.98 | 190 | 16 | 10 |
| 156 | 35.33 | -1.50 | GBIF | 12.54 | 80.91 | 20.03 | 18.35 | 168 | 25 | 50.34 | 381 | 110 | 7 |
| 157 | 38.63 | -3.40 | GBIF | 10.70 | 70.39 | 24.53 | 22.07 | 133 | 16 | 66.32 | 267 | 79 | 6 |
| 158 | 37.53 | -2.72 | GBIF | 12.55 | 71.31 | 22.17 | 19.40 | 164 | 8 | 84.09 | 197 | 34 | 8 |
| 159 | 37.26 | -2.70 | GBIF | 13.23 | 73.11 | 22.63 | 19.93 | 153 | 6 | 82.82 | 177 | 26 | 5 |
| 160 | 37.34 | -2.69 | GBIF | 13.03 | 73.22 | 22.60 | 19.87 | 153 | 6 | 83.22 | 182 | 27 | 4 |
| 161 | 34.82 | -1.59 | GBIF | 12.08 | 81.10 | 21.32 | 19.88 | 171 | 30 | 42.49 | 304 | 142 | 6 |
| 162 | 35.03 | -1.53 | GBIF | 12.66 | 81.14 | 20.78 | 19.32 | 157 | 28 | 43.59 | 294 | 129 | 4 |
| 163 | 35.12 | -1.44 | GBIF | 12.91 | 81.70 | 20.48 | 18.93 | 157 | 28 | 44.32 | 370 | 129 | 9 |
| 164 | 37.35 | -2.77 | GBIF | 12.84 | 72.55 | 22.03 | 19.37 | 185 | 10 | 81.57 | 199 | 43 | 5 |
| 165 | 37.34 | -2.62 | GBIF | 12.90 | 73.30 | 22.65 | 19.97 | 152 | 5 | 84.45 | 182 | 24 | 12 |
| 166 | 34.87 | -1.26 | GBIF | 11.43 | 81.03 | 18.78 | 17.27 | 223 | 48 | 39.54 | 341 | 209 | 5 |
| 167 | 38.38 | -2.94 | GBIF | 10.41 | 69.85 | 24.93 | 21.93 | 182 | 16 | 78.94 | 323 | 62 | 6 |
| 168 | 37.21 | -2.61 | GBIF | 12.89 | 73.67 | 22.73 | 20.02 | 138 | 3 | 83.59 | 175 | 18 | 3 |
| 169 | 38.76 | -3.39 | GBIF | 10.62 | 70.31 | 24.98 | 22.52 | 131 | 20 | 63.95 | 263 | 87 | 3 |
| 170 | 37.55 | 0.59 | GBIF | 13.19 | 82.97 | 23.45 | 22.95 | 125 | 4 | 100.05 | 174 | 14 | 20 |
| 171 | 37.02 | 2.76 | GBIF | 10.93 | 77.54 | 25.73 | 24.20 | 65 | 1 | 88.10 | 60 | 16 | 11 |
| 172 | 37.25 | -2.79 | GBIF | 12.21 | 70.98 | 21.33 | 18.73 | 192 | 12 | 79.72 | 203 | 53 | 4 |
| 173 | 37.88 | -2.83 | GBIF | 11.89 | 68.74 | 23.58 | 20.42 | 142 | 3 | 95.17 | 151 | 16 | 6 |
| 174 | 37.94 | -0.06 | GBIF | 11.58 | 75.71 | 23.55 | 21.88 | 186 | 1 | 115.04 | 251 | 6 | 7 |
| 175 | 35.11 | -1.56 | GBIF | 12.71 | 81.46 | 20.47 | 19.00 | 156 | 27 | 45.69 | 293 | 120 | 7 |
| 176 | 37.38 | -2.80 | GBIF | 12.07 | 70.61 | 21.25 | 18.57 | 199 | 13 | 78.63 | 220 | 57 | 7 |
| 177 | 35.20 | -1.47 | GBIF | 12.92 | 81.75 | 20.37 | 18.77 | 152 | 25 | 46.80 | 358 | 116 | 10 |
| 178 | 37.40 | -2.61 | GBIF | 12.85 | 72.60 | 22.42 | 19.70 | 151 | 5 | 83.08 | 188 | 27 | 6 |
| 179 | 37.57 | 0.60 | GBIF | 13.14 | 83.18 | 23.45 | 22.92 | 124 | 3 | 101.76 | 171 | 13 | 11 |
| 180 | 35.10 | -1.51 | GBIF | 12.75 | 81.21 | 20.22 | 18.73 | 158 | 28 | 44.78 | 295 | 127 | 9 |
| 181 | 35.09 | -1.59 | GBIF | 12.72 | 81.52 | 20.63 | 19.25 | 153 | 25 | 46.19 | 291 | 116 | 5 |
| 182 | 37.33 | -2.65 | GBIF | 12.78 | 72.63 | 22.60 | 19.88 | 150 | 5 | 84.31 | 181 | 23 | 4 |
| 183 | 35.28 | -1.39 | GBIF | 12.93 | 81.80 | 20.13 | 18.20 | 154 | 25 | 47.49 | 363 | 115 | 3 |
| 184 | 37.20 | -2.76 | GBIF | 12.40 | 71.26 | 21.85 | 19.17 | 164 | 8 | 80.61 | 191 | 34 | 4 |
| 185 | 36.76 | 0.38 | GBIF | 15.18 | 81.19 | 18.63 | 17.78 | 131 | 18 | 53.29 | 271 | 175 | 5 |
| 186 | 35.37 | -1.50 | GBIF | 12.42 | 80.63 | 19.88 | 18.18 | 170 | 25 | 52.30 | 379 | 105 | 8 |
| 187 | 37.35 | -2.75 | GBIF | 13.12 | 72.87 | 22.23 | 19.52 | 179 | 10 | 81.36 | 197 | 40 | 5 |
| 188 | 38.98 | -2.71 | GBIF | 9.87 | 69.00 | 26.32 | 23.95 | 150 | 23 | 70.17 | 256 | 72 | 2 |
| 189 | 35.21 | -1.51 | GBIF | 12.82 | 81.17 | 20.33 | 18.73 | 153 | 25 | 48.34 | 360 | 110 | 6 |
| 190 | 37.91 | -2.92 | GBIF | 11.42 | 68.78 | 23.62 | 20.53 | 134 | 3 | 95.65 | 134 | 13 | 5 |
| 191 | 35.02 | -1.54 | GBIF | 12.59 | 80.72 | 20.95 | 19.48 | 160 | 28 | 43.83 | 293 | 130 | 6 |
| 192 | 37.62 | 0.43 | GBIF | 13.60 | 80.95 | 23.23 | 22.93 | 164 | 4 | 100.50 | 226 | 257 | 13 |
| 193 | 37.21 | -2.68 | GBIF | 13.27 | 73.70 | 22.73 | 20.02 | 143 | 4 | 82.03 | 176 | 22 | 4 |
| 194 | 35.27 | -1.64 | GBIF | 12.48 | 81.06 | 20.35 | 18.43 | 162 | 22 | 53.34 | 375 | 97 | 6 |
| 195 | 37.53 | 0.56 | GBIF | 13.21 | 83.07 | 23.55 | 23.05 | 125 | 4 | 98.94 | 172 | 16 | 19 |
| 196 | 38.11 | -2.89 | GBIF | 10.84 | 68.19 | 23.28 | 20.23 | 154 | 4 | 97.40 | 152 | 18 | 4 |
| 197 | 35.14 | -1.52 | GBIF | 12.76 | 81.26 | 20.27 | 18.73 | 157 | 26 | 46.09 | 369 | 121 | 2 |
| 198 | 35.29 | -1.50 | GBIF | 12.71 | 80.94 | 20.53 | 18.55 | 160 | 25 | 49.38 | 368 | 109 | 3 |
| 199 | 35.02 | -1.45 | GBIF | 12.61 | 81.34 | 20.50 | 19.02 | 162 | 30 | 41.60 | 299 | 143 | 5 |

| 200 | 37.44 | 0.45 | GBIF | 13.82 | 84.25 | 22.68 | 22.53 | 168 | 8 | 89.82 | 258 | 268 | 15 |
| --- | --- | --- | --- | --- | --- | --- | --- | --- | --- | --- | --- | --- | --- |
| 201 | 35.25 | -1.54 | GBIF | 12.68 | 80.73 | 20.07 | 18.50 | 159 | 24 | 49.81 | 366 | 108 | 3 |
| 202 | 38.37 | -3.46 | GBIF | 10.05 | 67.45 | 22.22 | 19.23 | 172 | 17 | 73.36 | 206 | 65 | 17 |
| 203 | 37.40 | 0.72 | GBIF | 13.01 | 84.47 | 23.38 | 21.92 | 150 | 7 | 94.89 | 249 | 21 | 2 |
| 204 | 36.93 | 0.28 | GBIF | 14.55 | 80.39 | 18.75 | 17.82 | 164 | 20 | 60.82 | 330 | 157 | 11 |
| 205 | 37.61 | 0.62 | GBIF | 13.25 | 83.86 | 23.43 | 22.87 | 126 | 3 | 103.15 | 171 | 13 | 10 |
| 206 | 35.33 | -1.57 | GBIF | 12.12 | 80.78 | 18.63 | 16.98 | 185 | 27 | 52.44 | 410 | 113 | 4 |
| 207 | 37.23 | -2.76 | GBIF | 12.79 | 72.27 | 21.97 | 19.33 | 171 | 8 | 81.11 | 192 | 36 | 5 |
| 208 | 38.50 | -3.33 | GBIF | 10.29 | 69.54 | 24.22 | 21.30 | 147 | 13 | 73.70 | 286 | 53 | 7 |
| 209 | 38.84 | -3.35 | GBIF | 10.80 | 70.13 | 25.10 | 22.67 | 136 | 21 | 63.59 | 267 | 91 | 7 |
| 210 | 34.83 | -1.48 | GBIF | 12.55 | 82.03 | 21.22 | 19.80 | 173 | 34 | 40.66 | 305 | 153 | 8 |
| 211 | 38.11 | -2.79 | GBIF | 10.54 | 68.01 | 23.42 | 20.42 | 152 | 2 | 103.03 | 243 | 10 | 1 |
| 212 | 37.24 | -2.60 | GBIF | 12.68 | 72.89 | 22.73 | 20.03 | 146 | 3 | 84.41 | 176 | 20 | 8 |
| 213 | 37.92 | -2.94 | GBIF | 11.22 | 68.81 | 23.67 | 20.58 | 127 | 3 | 94.99 | 132 | 12 | 4 |
| 214 | 37.63 | 0.66 | GBIF | 13.05 | 83.65 | 23.47 | 22.90 | 127 | 3 | 105.17 | 168 | 12 | 9 |
| 215 | 35.25 | -1.50 | GBIF | 12.80 | 81.53 | 20.50 | 18.55 | 155 | 24 | 48.73 | 361 | 109 | 6 |
| 216 | 38.36 | -3.54 | GBIF | 10.67 | 67.94 | 22.22 | 19.27 | 153 | 15 | 73.07 | 201 | 55 | 7 |
| 217 | 35.06 | -1.45 | GBIF | 12.72 | 81.52 | 20.65 | 19.15 | 161 | 29 | 43.02 | 292 | 137 | 3 |
| 218 | 37.35 | -2.61 | GBIF | 12.77 | 73.00 | 22.55 | 19.88 | 150 | 5 | 82.92 | 184 | 26 | 12 |
| 219 | 37.38 | -2.74 | GBIF | 13.13 | 72.96 | 22.48 | 19.78 | 172 | 9 | 82.19 | 192 | 37 | 7 |
| 220 | 37.23 | -2.63 | GBIF | 12.74 | 73.23 | 22.67 | 19.88 | 140 | 3 | 83.62 | 177 | 20 | 5 |
| 221 | 36.86 | 0.28 | GBIF | 14.81 | 80.48 | 18.07 | 17.08 | 125 | 16 | 52.70 | 261 | 167 | 2 |
| 222 | 38.89 | -2.75 | GBIF | 9.96 | 69.16 | 26.02 | 23.62 | 157 | 22 | 73.21 | 281 | 70 | 2 |
| 223 | 39.98 | -3.31 | GBIF | 7.12 | 65.90 | 24.95 | 26.00 | 212 | 9 | 72.71 | 143 | 234 | 15 |
| 224 | 35.04 | -1.34 | GBIF | 12.68 | 81.25 | 20.15 | 18.63 | 169 | 35 | 39.71 | 298 | 160 | 3 |
| 225 | 37.37 | -2.64 | GBIF | 13.13 | 73.32 | 22.53 | 19.85 | 157 | 5 | 83.97 | 188 | 26 | 4 |
| 226 | 36.92 | 0.06 | GBIF | 14.63 | 78.67 | 17.65 | 16.60 | 134 | 25 | 50.13 | 291 | 176 | 10 |
| 227 | 36.66 | 0.61 | GBIF | 15.23 | 83.20 | 18.30 | 17.77 | 116 | 17 | 52.18 | 247 | 178 | 5 |
| 228 | 35.19 | -1.57 | GBIF | 12.73 | 81.62 | 20.25 | 18.75 | 158 | 25 | 48.28 | 365 | 113 | 7 |
| 229 | 35.17 | -1.25 | GBIF | 12.98 | 82.17 | 19.98 | 18.38 | 179 | 35 | 42.49 | 300 | 159 | 3 |
| 230 | 36.90 | -2.00 | GBIF | 11.30 | 71.97 | 19.98 | 17.55 | 119 | 3 | 81.03 | 221 | 17 | 5 |
| 231 | 39.87 | -3.30 | GBIF | 7.32 | 65.99 | 24.90 | 25.93 | 205 | 13 | 65.74 | 170 | 170 | 3 |
| 232 | 35.10 | -1.21 | GBIF | 13.03 | 82.49 | 19.67 | 18.15 | 186 | 39 | 39.69 | 308 | 182 | 11 |
| 233 | 37.77 | 0.45 | GBIF | 12.78 | 81.89 | 22.83 | 22.35 | 202 | 3 | 107.10 | 275 | 11 | 10 |
| 234 | 37.66 | 0.59 | GBIF | 13.54 | 83.59 | 23.53 | 22.95 | 128 | 3 | 105.46 | 167 | 13 | 8 |
| 235 | 35.31 | -1.51 | GBIF | 12.64 | 81.04 | 20.12 | 18.30 | 168 | 26 | 49.69 | 379 | 112 | 5 |
| 236 | 37.28 | -2.60 | GBIF | 12.63 | 72.98 | 22.70 | 20.03 | 143 | 4 | 84.01 | 177 | 21 | 4 |
| 237 | 36.95 | 0.29 | GBIF | 14.49 | 80.96 | 18.63 | 17.73 | 159 | 19 | 61.01 | 318 | 156 | 5 |
| 238 | 35.17 | -1.49 | GBIF | 12.91 | 81.70 | 20.40 | 18.82 | 150 | 26 | 45.61 | 358 | 119 | 2 |
| 239 | 35.01 | -1.53 | GBIF | 12.57 | 81.13 | 20.75 | 19.32 | 168 | 29 | 44.04 | 298 | 135 | 11 |
| 240 | 35.12 | -1.45 | GBIF | 12.95 | 81.45 | 20.50 | 18.97 | 156 | 27 | 44.69 | 368 | 127 | 9 |
| 241 | 35.16 | -1.53 | GBIF | 12.73 | 81.10 | 20.13 | 18.60 | 162 | 26 | 47.54 | 373 | 118 | 2 |
| 242 | 35.13 | -1.34 | GBIF | 13.05 | 81.56 | 20.52 | 18.95 | 160 | 31 | 42.25 | 288 | 142 | 5 |
| 243 | 35.18 | -1.33 | GBIF | 12.98 | 82.12 | 20.32 | 18.73 | 161 | 30 | 43.30 | 374 | 140 | 3 |
| 244 | 35.06 | -1.21 | GBIF | 12.69 | 82.41 | 19.97 | 18.47 | 174 | 39 | 38.20 | 298 | 179 | 7 |
| 245 | 35.15 | -1.46 | GBIF | 12.97 | 81.55 | 20.48 | 18.90 | 152 | 27 | 45.17 | 361 | 122 | 2 |
| 246 | 35.27 | -1.34 | GBIF | 12.97 | 82.07 | 19.60 | 17.97 | 160 | 27 | 46.66 | 371 | 123 | 5 |
| 247 | 39.00 | -3.86 | GBIF | 9.43 | 67.32 | 25.15 | 22.35 | 102 | 28 | 48.90 | 137 | 98 | 4 |
| 248 | 37.90 | 2.38 | GBIF | 11.01 | 74.89 | 24.68 | 22.45 | 91 | 1 | 104.55 | 69 | 4 | 3 |
| 249 | 38.00 | -0.10 | GBIF | 11.41 | 75.55 | 24.05 | 21.97 | 170 | 1 | 118.46 | 220 | 3 | 14 |

| 250 | 35.02 | -1.35 | GBIF | 12.64 | 81.56 | 20.30 | 18.77 | 172 | 34 | 40.48 | 299 | 158 | 3 |
| --- | --- | --- | --- | --- | --- | --- | --- | --- | --- | --- | --- | --- | --- |
| 251 | 35.17 | -1.40 | GBIF | 12.88 | 82.01 | 20.58 | 18.70 | 160 | 27 | 44.92 | 372 | 130 | 11 |
| 252 | 35.12 | -1.27 | GBIF | 13.08 | 82.29 | 20.15 | 18.63 | 165 | 33 | 40.90 | 294 | 153 | 3 |
| 253 | 37.48 | 0.74 | GBIF | 12.36 | 83.50 | 23.27 | 21.78 | 142 | 5 | 98.48 | 238 | 16 | 2 |
| 254 | 37.62 | 0.55 | GBIF | 13.57 | 82.72 | 23.45 | 22.97 | 134 | 3 | 103.13 | 182 | 13 | 10 |
| 255 | 35.04 | -1.57 | GBIF | 12.71 | 81.46 | 20.78 | 19.38 | 157 | 26 | 44.64 | 292 | 124 | 12 |
| 256 | 37.22 | -2.77 | GBIF | 12.40 | 71.68 | 21.67 | 19.05 | 170 | 10 | 79.29 | 196 | 41 | 4 |
| 257 | 37.74 | 0.45 | GBIF | 12.91 | 81.18 | 23.03 | 22.60 | 183 | 3 | 106.83 | 246 | 11 | 6 |
| 258 | 35.20 | -1.45 | GBIF | 12.91 | 81.70 | 20.48 | 18.85 | 157 | 26 | 46.33 | 363 | 121 | 6 |
| 259 | 37.54 | 0.79 | GBIF | 12.89 | 83.71 | 23.72 | 22.20 | 137 | 4 | 99.84 | 213 | 14 | 7 |
| 260 | 35.17 | -1.03 | GBIF | 12.72 | 81.52 | 19.37 | 17.80 | 178 | 43 | 38.20 | 294 | 191 | 14 |
| 261 | 39.00 | 0.81 | GBIF | 11.37 | 78.94 | 26.97 | 25.62 | 115 | 0 | 136.19 | 151 | 0 | 1 |
| 262 | 35.30 | -1.53 | GBIF | 12.39 | 80.99 | 19.73 | 18.03 | 171 | 26 | 51.08 | 382 | 110 | 7 |
| 263 | 37.60 | 0.79 | GBIF | 12.93 | 83.93 | 23.17 | 22.60 | 130 | 3 | 104.52 | 197 | 12 | 4 |
| 264 | 38.32 | -3.44 | GBIF | 9.75 | 66.33 | 20.73 | 17.73 | 200 | 22 | 72.37 | 257 | 76 | 16 |
| 265 | 37.40 | -2.72 | GBIF | 13.15 | 73.06 | 22.60 | 19.90 | 164 | 8 | 83.01 | 187 | 33 | 15 |
| 266 | 37.31 | -2.58 | GBIF | 12.60 | 72.83 | 22.60 | 19.97 | 144 | 4 | 83.69 | 180 | 22 | 4 |
| 267 | 35.25 | -1.46 | GBIF | 12.99 | 81.71 | 20.53 | 18.88 | 148 | 24 | 47.31 | 350 | 110 | 11 |
| 268 | 37.33 | -2.64 | GBIF | 12.84 | 72.96 | 22.65 | 19.95 | 151 | 5 | 84.19 | 182 | 24 | 5 |
| 269 | 38.97 | -3.30 | GBIF | 10.45 | 70.13 | 25.28 | 22.88 | 133 | 22 | 62.21 | 258 | 94 | 2 |
| 270 | 37.40 | 0.16 | GBIF | 12.88 | 82.59 | 16.87 | 16.02 | 235 | 28 | 81.26 | 338 | 92 | 6 |
| 271 | 37.89 | -0.05 | GBIF | 10.98 | 75.17 | 22.97 | 21.25 | 207 | 2 | 111.93 | 294 | 11 | 14 |
| 272 | 37.28 | -2.78 | GBIF | 12.63 | 71.73 | 21.80 | 19.17 | 186 | 10 | 81.29 | 198 | 43 | 5 |
| 273 | 37.43 | 0.59 | GBIF | 13.54 | 84.64 | 24.43 | 23.13 | 124 | 5 | 93.26 | 197 | 20 | 14 |
| 274 | 37.27 | -2.68 | GBIF | 12.82 | 72.82 | 22.53 | 19.78 | 142 | 4 | 83.04 | 177 | 22 | 8 |
| 275 | 37.38 | -2.65 | GBIF | 13.12 | 73.28 | 22.55 | 19.85 | 153 | 5 | 83.51 | 187 | 26 | 5 |
| 276 | 37.32 | -2.80 | GBIF | 12.36 | 71.02 | 21.52 | 18.83 | 196 | 12 | 80.12 | 208 | 52 | 5 |
| 277 | 37.35 | -2.79 | GBIF | 12.41 | 71.31 | 21.67 | 19.03 | 196 | 12 | 81.02 | 207 | 49 | 5 |
| 278 | 37.29 | -2.79 | GBIF | 12.54 | 71.67 | 21.68 | 19.03 | 184 | 11 | 79.44 | 202 | 48 | 5 |
| 279 | 37.34 | -2.76 | GBIF | 13.12 | 72.87 | 22.20 | 19.52 | 177 | 10 | 81.20 | 196 | 40 | 5 |
| 280 | 37.25 | -2.67 | GBIF | 12.83 | 72.92 | 22.57 | 19.78 | 139 | 4 | 82.78 | 175 | 21 | 8 |
| 281 | 37.42 | 0.79 | GBIF | 12.42 | 82.78 | 20.98 | 19.47 | 221 | 8 | 99.47 | 349 | 24 | 8 |
| 282 | 38.93 | -3.26 | GBIF | 10.42 | 70.38 | 25.33 | 22.95 | 137 | 22 | 63.70 | 261 | 95 | 11 |
| 283 | 35.27 | -1.40 | GBIF | 12.89 | 82.11 | 20.38 | 18.45 | 152 | 25 | 47.46 | 358 | 113 | 3 |
| 284 | 37.39 | -2.69 | GBIF | 13.14 | 73.01 | 22.67 | 19.95 | 159 | 6 | 83.99 | 187 | 27 | 6 |
| 285 | 36.90 | 0.36 | GBIF | 14.67 | 81.48 | 18.77 | 17.83 | 147 | 17 | 59.27 | 299 | 157 | 5 |
| 286 | 37.35 | -2.72 | GBIF | 13.27 | 73.75 | 22.57 | 19.90 | 163 | 7 | 82.53 | 186 | 31 | 13 |
| 287 | 35.35 | -1.52 | GBIF | 12.25 | 81.13 | 19.23 | 17.55 | 179 | 26 | 52.15 | 396 | 111 | 8 |
| 288 | 38.90 | -2.66 | GBIF | 10.03 | 69.14 | 26.35 | 23.93 | 153 | 20 | 73.30 | 266 | 65 | 1 |
| 289 | 37.38 | -2.76 | GBIF | 13.07 | 72.59 | 22.30 | 19.60 | 174 | 10 | 81.13 | 195 | 40 | 6 |
| 290 | 37.21 | -2.71 | GBIF | 13.21 | 73.38 | 22.58 | 19.92 | 153 | 5 | 82.40 | 179 | 25 | 4 |
| 291 | 38.97 | -3.73 | GBIF | 9.68 | 67.66 | 24.58 | 22.20 | 109 | 27 | 52.27 | 154 | 94 | 5 |
| 292 | 37.08 | 0.04 | GBIF | 14.58 | 79.69 | 16.93 | 16.15 | 138 | 23 | 54.15 | 224 | 153 | 10 |
| 293 | 37.57 | 0.43 | GBIF | 13.18 | 81.83 | 23.15 | 22.83 | 158 | 4 | 98.88 | 219 | 246 | 6 |
| 294 | 36.80 | 0.32 | GBIF | 15.14 | 80.54 | 18.30 | 17.30 | 133 | 17 | 54.24 | 276 | 170 | 3 |
| 295 | 35.26 | -1.06 | GBIF | 12.57 | 81.66 | 19.25 | 17.63 | 169 | 39 | 40.42 | 388 | 167 | 13 |
| 296 | 37.01 | -0.11 | GBIF | 14.23 | 79.52 | 17.27 | 16.03 | 133 | 29 | 54.52 | 308 | 231 | 22 |
| 297 | 36.99 | 0.15 | GBIF | 15.06 | 79.67 | 17.78 | 16.85 | 125 | 18 | 52.35 | 271 | 168 | 4 |
| 298 | 37.88 | -2.98 | GBIF | 12.27 | 69.74 | 23.75 | 20.70 | 143 | 3 | 94.18 | 148 | 14 | 5 |
| 299 | 37.32 | -2.71 | GBIF | 13.38 | 73.53 | 22.63 | 19.93 | 166 | 7 | 83.56 | 184 | 31 | 9 |

| 300 | 37.33 | -2.71 | GBIF | 13.36 | 73.40 | 22.62 | 19.93 | 163 | 7 | 82.91 | 183 | 31 | 13 |
| --- | --- | --- | --- | --- | --- | --- | --- | --- | --- | --- | --- | --- | --- |
| 301 | 38.56 | -3.34 | GBIF | 10.60 | 69.74 | 24.65 | 21.72 | 146 | 15 | 69.95 | 286 | 61 | 16 |
| 302 | 38.57 | -3.24 | GBIF | 10.27 | 69.37 | 24.95 | 22.02 | 162 | 17 | 70.82 | 306 | 67 | 1 |
| 303 | 35.13 | -1.22 | GBIF | 13.03 | 82.44 | 19.70 | 18.12 | 184 | 38 | 40.22 | 309 | 175 | 3 |
| 304 | 36.65 | 0.15 | GBIF | 15.37 | 79.62 | 17.80 | 16.80 | 109 | 27 | 41.03 | 223 | 189 | 11 |
| 305 | 35.12 | -1.38 | GBIF | 12.98 | 82.12 | 20.53 | 18.97 | 162 | 29 | 43.33 | 375 | 139 | 11 |
| 306 | 37.39 | -2.43 | GBIF | 11.63 | 70.51 | 22.38 | 19.85 | 134 | 2 | 87.58 | 255 | 16 | 5 |
| 307 | 38.94 | -2.63 | GBIF | 10.17 | 69.63 | 26.40 | 24.02 | 152 | 20 | 72.65 | 260 | 68 | 2 |
| 308 | 37.21 | -2.66 | GBIF | 13.21 | 73.79 | 22.75 | 20.00 | 144 | 4 | 83.33 | 174 | 21 | 4 |
| 309 | 39.21 | -3.01 | GBIF | 10.03 | 70.16 | 26.30 | 24.12 | 130 | 25 | 59.19 | 230 | 105 | 3 |
| 310 | 38.43 | -3.27 | GBIF | 10.36 | 69.99 | 23.80 | 21.38 | 144 | 11 | 78.38 | 281 | 42 | 7 |
| 311 | 37.32 | -2.76 | GBIF | 13.05 | 72.50 | 22.05 | 19.42 | 178 | 10 | 80.63 | 198 | 42 | 5 |
| 312 | 37.26 | -2.71 | GBIF | 13.34 | 73.71 | 22.65 | 19.97 | 156 | 6 | 82.32 | 180 | 28 | 4 |
| 313 | 35.05 | -1.37 | GBIF | 12.75 | 81.73 | 20.35 | 18.82 | 171 | 33 | 41.72 | 390 | 152 | 3 |
| 314 | 38.42 | -3.32 | GBIF | 9.26 | 67.09 | 21.28 | 18.70 | 179 | 14 | 81.57 | 362 | 50 | 7 |
| 315 | 35.06 | -1.60 | GBIF | 12.73 | 81.62 | 20.68 | 19.37 | 159 | 25 | 46.32 | 294 | 119 | 5 |
| 316 | 38.42 | -3.35 | GBIF | 9.13 | 66.67 | 20.53 | 17.88 | 184 | 15 | 79.89 | 245 | 54 | 8 |
| 317 | 36.81 | 0.11 | GBIF | 15.17 | 78.58 | 17.78 | 16.73 | 125 | 23 | 46.24 | 262 | 167 | 18 |
| 318 | 38.85 | -3.79 | GBIF | 9.77 | 67.82 | 24.42 | 22.07 | 111 | 22 | 55.30 | 154 | 84 | 5 |
| 319 | 37.39 | -2.78 | GBIF | 12.55 | 71.71 | 21.82 | 19.12 | 185 | 11 | 79.73 | 208 | 47 | 6 |
| 320 | 38.61 | -3.37 | GBIF | 10.67 | 69.77 | 24.98 | 22.05 | 142 | 18 | 66.98 | 280 | 69 | 11 |
| 321 | 35.03 | -1.31 | GBIF | 12.60 | 81.82 | 20.22 | 18.73 | 172 | 36 | 40.06 | 298 | 162 | 4 |
| 322 | 35.03 | -1.21 | GBIF | 12.12 | 81.87 | 19.00 | 17.53 | 205 | 44 | 39.87 | 321 | 197 | 12 |
| 323 | 37.28 | -2.74 | GBIF | 13.17 | 73.19 | 22.30 | 19.65 | 171 | 8 | 81.92 | 191 | 33 | 4 |
| 324 | 38.67 | -3.22 | GBIF | 10.34 | 69.88 | 25.38 | 22.45 | 164 | 20 | 69.73 | 305 | 72 | 1 |
| 325 | 36.86 | 0.38 | GBIF | 14.72 | 81.31 | 19.07 | 18.15 | 163 | 19 | 61.45 | 334 | 160 | 7 |
| 326 | 37.58 | 1.06 | GBIF | 12.63 | 83.06 | 24.72 | 23.22 | 116 | 2 | 104.40 | 189 | 10 | 4 |
| 327 | 38.74 | -3.28 | GBIF | 10.52 | 70.17 | 25.50 | 22.55 | 146 | 21 | 66.28 | 282 | 75 | 4 |
| 328 | 37.38 | -2.71 | GBIF | 13.18 | 73.19 | 22.63 | 19.90 | 163 | 7 | 83.52 | 185 | 31 | 14 |
| 329 | 36.81 | 0.03 | GBIF | 14.77 | 78.59 | 17.43 | 16.30 | 113 | 28 | 41.16 | 248 | 174 | 3 |
| 330 | 37.36 | -2.76 | GBIF | 13.09 | 72.73 | 22.27 | 19.58 | 178 | 9 | 81.54 | 197 | 39 | 5 |
| 331 | 37.34 | -2.78 | GBIF | 12.62 | 71.69 | 21.83 | 19.17 | 189 | 11 | 80.74 | 203 | 47 | 5 |
| 332 | 38.59 | -3.00 | GBIF | 10.47 | 69.83 | 25.92 | 22.93 | 204 | 25 | 70.99 | 356 | 93 | 6 |
| 333 | 36.88 | 0.20 | GBIF | 14.89 | 78.79 | 18.12 | 17.13 | 141 | 19 | 53.81 | 295 | 167 | 3 |
| 334 | 35.12 | -1.21 | GBIF | 13.02 | 82.44 | 19.60 | 18.10 | 184 | 39 | 40.15 | 307 | 178 | 4 |
| 335 | 38.72 | -3.36 | GBIF | 10.74 | 70.67 | 25.37 | 22.42 | 143 | 20 | 65.43 | 278 | 74 | 4 |
| 336 | 35.13 | -1.33 | GBIF | 13.09 | 81.82 | 20.47 | 18.93 | 162 | 31 | 42.10 | 288 | 145 | 4 |
| 337 | 37.56 | 0.68 | GBIF | 12.69 | 83.50 | 22.92 | 22.42 | 134 | 4 | 100.15 | 195 | 15 | 5 |
| 338 | 38.87 | -3.36 | GBIF | 10.73 | 70.56 | 25.13 | 22.70 | 133 | 21 | 63.23 | 262 | 92 | 7 |
| 339 | 38.99 | -3.42 | GBIF | 10.29 | 69.54 | 25.15 | 22.80 | 123 | 22 | 59.00 | 241 | 96 | 10 |
| 340 | 34.88 | -1.52 | GBIF | 12.57 | 81.66 | 21.18 | 19.62 | 171 | 31 | 42.36 | 303 | 143 | 8 |
| 341 | 34.86 | -1.43 | GBIF | 12.29 | 81.94 | 20.55 | 19.05 | 178 | 37 | 39.40 | 313 | 165 | 6 |
| 342 | 35.03 | -1.56 | GBIF | 12.60 | 81.29 | 20.78 | 19.33 | 156 | 27 | 44.08 | 293 | 126 | 13 |
| 343 | 35.18 | -1.54 | GBIF | 12.78 | 80.85 | 20.17 | 18.60 | 164 | 26 | 48.86 | 375 | 114 | 3 |
| 344 | 35.06 | -1.52 | GBIF | 12.73 | 81.10 | 20.50 | 19.00 | 161 | 28 | 44.30 | 297 | 130 | 9 |
| 345 | 38.55 | -3.03 | GBIF | 10.83 | 70.81 | 25.72 | 22.72 | 216 | 24 | 72.67 | 373 | 91 | 3 |
| 346 | 37.63 | 0.44 | GBIF | 13.53 | 81.48 | 23.22 | 22.90 | 164 | 4 | 101.45 | 224 | 14 | 4 |
| 347 | 34.87 | 1.59 | GBIF | 12.73 | 83.72 | 20.82 | 21.70 | 142 | 22 | 51.39 | 219 | 359 | 6 |
| 348 | 34.94 | -1.49 | GBIF | 12.54 | 81.97 | 20.77 | 19.23 | 171 | 31 | 42.03 | 303 | 145 | 13 |
| 349 | 36.92 | -0.34 | GBIF | 12.77 | 76.91 | 17.38 | 15.72 | 157 | 31 | 56.63 | 358 | 111 | 15 |

| 350 | 38.46 | -3.20 | GBIF | 10.12 | 69.29 | 23.65 | 21.22 | 152 | 12 | 77.36 | 299 | 48 | 5 |
| --- | --- | --- | --- | --- | --- | --- | --- | --- | --- | --- | --- | --- | --- |
| 351 | 34.92 | 1.58 | GBIF | 12.39 | 83.73 | 20.55 | 21.42 | 145 | 21 | 52.71 | 218 | 360 | 11 |
| 352 | 35.03 | -1.60 | GBIF | 12.72 | 81.52 | 20.73 | 19.43 | 159 | 26 | 44.86 | 295 | 124 | 11 |
| 353 | 34.81 | -1.57 | GBIF | 12.16 | 81.06 | 21.50 | 20.02 | 172 | 29 | 43.04 | 300 | 141 | 8 |
| 354 | 38.49 | -3.19 | GBIF | 10.31 | 70.12 | 24.25 | 21.82 | 157 | 14 | 75.89 | 302 | 53 | 10 |
| 355 | 36.98 | -0.36 | GBIF | 12.82 | 76.80 | 18.25 | 16.47 | 157 | 28 | 60.70 | 372 | 102 | 15 |
| 356 | 34.89 | 1.57 | GBIF | 12.48 | 83.72 | 20.58 | 21.47 | 147 | 23 | 51.57 | 241 | 372 | 13 |
| 357 | 38.83 | -3.41 | GBIF | 10.63 | 69.96 | 25.07 | 22.63 | 130 | 22 | 61.70 | 253 | 91 | 4 |
| 358 | 37.00 | -0.24 | GBIF | 14.12 | 78.86 | 17.43 | 15.98 | 149 | 24 | 58.95 | 337 | 91 | 14 |
| 359 | 37.32 | -2.60 | GBIF | 12.63 | 72.98 | 22.68 | 20.03 | 145 | 4 | 84.32 | 177 | 21 | 5 |
| 360 | 36.66 | -0.54 | GBIF | 10.58 | 78.40 | 9.95 | 9.87 | 211 | 43 | 36.62 | 405 | 348 | 7 |
| 361 | 35.19 | -1.54 | GBIF | 12.77 | 81.32 | 20.28 | 18.73 | 159 | 26 | 48.38 | 367 | 115 | 4 |
| 362 | 35.44 | -1.31 | GBIF | 12.83 | 82.26 | 19.05 | 17.03 | 162 | 24 | 51.82 | 367 | 105 | 2 |
| 363 | 38.11 | -2.98 | GBIF | 10.32 | 68.32 | 23.27 | 20.28 | 150 | 5 | 92.71 | 150 | 21 | 9 |
| 364 | 38.44 | -3.02 | GBIF | 10.77 | 70.37 | 25.50 | 22.45 | 209 | 23 | 74.59 | 363 | 84 | 2 |
| 365 | 36.69 | -0.56 | GBIF | 10.60 | 78.52 | 10.82 | 10.20 | 217 | 44 | 38.54 | 414 | 325 | 5 |
| 366 | 38.47 | -3.12 | GBIF | 10.65 | 70.53 | 24.90 | 21.92 | 176 | 16 | 76.64 | 324 | 60 | 3 |
| 367 | 35.17 | -1.44 | GBIF | 12.92 | 81.75 | 20.50 | 18.90 | 156 | 27 | 45.61 | 364 | 123 | 2 |
| 368 | 38.55 | -3.09 | GBIF | 10.61 | 70.25 | 25.38 | 22.45 | 197 | 20 | 72.96 | 352 | 80 | 6 |
| 369 | 38.15 | 0.17 | GBIF | 11.54 | 77.46 | 24.12 | 22.78 | 170 | 1 | 119.77 | 215 | 4 | 0 |
| 370 | 38.95 | -3.22 | GBIF | 10.38 | 70.58 | 25.53 | 23.13 | 138 | 22 | 63.49 | 263 | 96 | 7 |
| 371 | 36.91 | 0.30 | GBIF | 14.59 | 80.62 | 18.85 | 17.92 | 162 | 19 | 60.01 | 327 | 160 | 12 |
| 372 | 37.93 | -2.93 | GBIF | 11.02 | 68.43 | 23.58 | 20.50 | 128 | 3 | 94.93 | 130 | 12 | 4 |
| 373 | 37.22 | -2.63 | GBIF | 12.88 | 73.57 | 22.70 | 19.92 | 137 | 3 | 82.93 | 264 | 20 | 3 |
| 374 | 35.05 | -1.49 | GBIF | 12.73 | 81.10 | 20.62 | 19.12 | 160 | 28 | 43.50 | 296 | 132 | 4 |
| 375 | 38.78 | -3.16 | GBIF | 10.74 | 71.14 | 25.97 | 23.05 | 163 | 22 | 67.95 | 302 | 95 | 2 |
| 376 | 37.08 | -2.71 | GBIF | 12.84 | 73.80 | 22.70 | 20.08 | 139 | 3 | 83.42 | 175 | 17 | 6 |
| 377 | 35.28 | 1.78 | GBIF | 12.22 | 83.16 | 20.35 | 21.03 | 118 | 14 | 54.09 | 180 | 278 | 5 |
| 378 | 35.07 | -1.42 | GBIF | 12.71 | 81.46 | 20.70 | 19.13 | 159 | 29 | 42.37 | 291 | 139 | 3 |
| 379 | 37.40 | -2.75 | GBIF | 13.08 | 72.69 | 22.42 | 19.70 | 172 | 9 | 82.03 | 194 | 37 | 11 |
| 380 | 35.13 | -1.57 | GBIF | 12.68 | 81.25 | 20.32 | 18.83 | 158 | 26 | 46.75 | 369 | 118 | 11 |
| 381 | 35.05 | -1.58 | GBIF | 12.73 | 81.57 | 20.73 | 19.35 | 157 | 26 | 44.75 | 293 | 123 | 11 |
| 382 | 35.39 | -1.69 | GBIF | 12.22 | 80.91 | 19.88 | 17.92 | 154 | 19 | 57.05 | 366 | 79 | 4 |
| 383 | 38.19 | -2.94 | GBIF | 10.47 | 68.41 | 23.17 | 20.20 | 159 | 6 | 93.79 | 162 | 23 | 9 |
| 384 | 37.37 | -2.61 | GBIF | 12.88 | 72.74 | 22.52 | 19.82 | 153 | 5 | 83.56 | 186 | 26 | 8 |
| 385 | 37.74 | 0.33 | GBIF | 12.51 | 80.18 | 22.18 | 21.68 | 251 | 3 | 104.70 | 325 | 11 | 5 |
| 386 | 36.98 | 0.34 | GBIF | 14.64 | 81.80 | 18.98 | 18.08 | 164 | 18 | 65.06 | 246 | 142 | 5 |
| 387 | 36.96 | 0.32 | GBIF | 14.58 | 81.02 | 18.95 | 18.05 | 168 | 19 | 64.14 | 338 | 146 | 5 |
| 388 | 35.07 | -1.44 | GBIF | 12.76 | 81.26 | 20.62 | 19.08 | 159 | 29 | 43.16 | 371 | 134 | 2 |
| 389 | 36.88 | 0.37 | GBIF | 14.69 | 81.62 | 18.77 | 17.83 | 146 | 17 | 59.37 | 301 | 160 | 8 |
| 390 | 38.35 | -2.78 | GBIF | 10.37 | 69.11 | 24.62 | 22.03 | 167 | 9 | 88.57 | 297 | 39 | 1 |
| 391 | 37.41 | 0.54 | GBIF | 13.78 | 84.56 | 24.18 | 22.83 | 137 | 7 | 91.06 | 219 | 23 | 6 |
| 392 | 35.33 | -1.45 | GBIF | 12.88 | 81.54 | 20.45 | 18.47 | 153 | 22 | 51.36 | 356 | 99 | 3 |
| 393 | 36.96 | 0.31 | GBIF | 14.52 | 81.10 | 18.83 | 17.92 | 166 | 19 | 63.19 | 249 | 150 | 5 |
| 394 | 37.05 | 0.54 | GBIF | 13.60 | 83.44 | 19.18 | 18.20 | 166 | 18 | 72.74 | 334 | 107 | 5 |
| 395 | 38.93 | -3.29 | GBIF | 10.56 | 70.39 | 25.35 | 22.93 | 135 | 22 | 63.17 | 262 | 94 | 3 |
| 396 | 36.81 | 0.24 | GBIF | 15.10 | 79.06 | 18.05 | 17.00 | 131 | 18 | 51.63 | 273 | 171 | 3 |
| 397 | 35.17 | -1.57 | GBIF | 12.71 | 80.94 | 20.30 | 18.82 | 160 | 25 | 48.51 | 370 | 113 | 11 |
| 398 | 35.03 | -1.58 | GBIF | 12.66 | 81.14 | 20.72 | 19.35 | 159 | 26 | 44.74 | 296 | 124 | 5 |
| 399 | 35.19 | -1.31 | GBIF | 12.94 | 82.43 | 20.17 | 18.57 | 161 | 30 | 43.88 | 374 | 137 | 3 |

| 400 | 37.56 | 0.72 | GBIF | 12.43 | 83.45 | 22.38 | 21.85 | 152 | 4 | 99.87 | 232 | 14 | 3 |
| --- | --- | --- | --- | --- | --- | --- | --- | --- | --- | --- | --- | --- | --- |
| 401 | 38.66 | -3.06 | GBIF | 10.73 | 70.15 | 26.22 | 23.22 | 191 | 25 | 69.28 | 338 | 88 | 7 |
| 402 | 39.12 | -3.11 | GBIF | 10.52 | 70.58 | 26.05 | 23.82 | 134 | 22 | 61.21 | 243 | 101 | 4 |
| 403 | 37.87 | -0.19 | GBIF | 10.99 | 74.77 | 22.80 | 20.97 | 211 | 1 | 115.04 | 305 | 7 | 8 |
| 404 | 35.19 | -1.40 | GBIF | 12.85 | 81.33 | 20.68 | 18.72 | 158 | 28 | 45.17 | 368 | 129 | 11 |
| 405 | 37.21 | -0.11 | GBIF | 10.38 | 74.64 | 12.92 | 12.03 | 265 | 42 | 58.91 | 441 | 221 | 3 |
| 406 | 38.71 | -3.37 | GBIF | 10.74 | 70.21 | 25.37 | 22.40 | 144 | 20 | 65.71 | 277 | 75 | 10 |
| 407 | 38.77 | -3.57 | GBIF | 10.35 | 69.46 | 24.48 | 22.12 | 122 | 18 | 61.40 | 170 | 83 | 13 |
| 408 | 35.19 | -1.21 | GBIF | 13.02 | 82.91 | 19.68 | 18.13 | 174 | 36 | 41.16 | 396 | 163 | 4 |
| 409 | 38.78 | -3.30 | GBIF | 10.69 | 70.81 | 25.53 | 22.63 | 146 | 21 | 65.99 | 279 | 91 | 7 |
| 410 | 38.87 | -3.38 | GBIF | 10.68 | 70.75 | 25.13 | 22.70 | 132 | 22 | 61.92 | 259 | 94 | 3 |
| 411 | 35.09 | -1.52 | GBIF | 12.73 | 81.57 | 20.33 | 18.87 | 159 | 28 | 44.63 | 372 | 128 | 9 |
| 412 | 37.29 | -2.63 | GBIF | 12.54 | 72.92 | 22.62 | 19.90 | 148 | 4 | 84.25 | 179 | 22 | 12 |
| 413 | 34.94 | -1.45 | GBIF | 12.55 | 82.03 | 20.38 | 18.85 | 176 | 33 | 41.11 | 307 | 156 | 10 |
| 414 | 37.23 | -2.70 | GBIF | 13.22 | 73.43 | 22.67 | 19.97 | 146 | 5 | 81.77 | 175 | 26 | 4 |
| 415 | 38.80 | -3.59 | GBIF | 10.32 | 69.71 | 24.58 | 22.18 | 121 | 18 | 61.18 | 165 | 80 | 13 |
| 416 | 37.26 | -2.79 | GBIF | 12.41 | 71.31 | 21.55 | 18.93 | 185 | 11 | 80.28 | 200 | 47 | 4 |
| 417 | 37.33 | -2.60 | GBIF | 12.69 | 72.94 | 22.65 | 19.98 | 145 | 4 | 84.17 | 179 | 21 | 6 |
| 418 | 35.08 | -1.41 | GBIF | 12.74 | 81.68 | 20.52 | 18.95 | 170 | 31 | 43.23 | 386 | 144 | 3 |
| 419 | 38.87 | -3.53 | GBIF | 10.34 | 70.35 | 24.83 | 22.48 | 122 | 19 | 60.80 | 233 | 83 | 2 |
| 420 | 34.88 | 1.57 | GBIF | 12.55 | 83.11 | 20.63 | 21.53 | 148 | 24 | 51.64 | 249 | 376 | 13 |
| 421 | 39.37 | -3.11 | GBIF | 9.47 | 69.61 | 26.27 | 24.20 | 116 | 22 | 55.03 | 193 | 106 | 6 |
| 422 | 36.90 | 0.10 | GBIF | 15.04 | 78.34 | 18.05 | 17.03 | 140 | 23 | 50.79 | 298 | 159 | 7 |
| 423 | 38.85 | -3.28 | GBIF | 10.70 | 70.39 | 25.18 | 22.78 | 139 | 21 | 64.00 | 271 | 93 | 3 |
| 424 | 37.22 | -2.76 | GBIF | 12.63 | 71.73 | 21.97 | 19.32 | 164 | 8 | 80.39 | 191 | 34 | 4 |
| 425 | 38.70 | -3.59 | GBIF | 10.02 | 68.66 | 24.02 | 21.65 | 125 | 16 | 65.14 | 169 | 74 | 6 |
| 426 | 39.35 | -4.30 | GBIF | 8.11 | 64.35 | 24.85 | 26.73 | 246 | 17 | 67.11 | 100 | 236 | 6 |
| 427 | 39.19 | -3.37 | GBIF | 9.56 | 68.76 | 25.00 | 22.82 | 122 | 25 | 55.89 | 216 | 85 | 2 |
| 428 | 37.28 | -2.65 | GBIF | 12.60 | 72.83 | 22.53 | 19.78 | 143 | 4 | 83.38 | 175 | 23 | 12 |
| 429 | 38.90 | -3.24 | GBIF | 10.43 | 70.50 | 25.20 | 22.83 | 140 | 22 | 63.85 | 267 | 96 | 3 |
| 430 | 37.32 | -2.75 | GBIF | 13.24 | 73.16 | 22.27 | 19.62 | 180 | 9 | 82.25 | 195 | 38 | 5 |
| 431 | 39.16 | -3.23 | GBIF | 10.01 | 69.99 | 25.45 | 23.22 | 130 | 23 | 59.91 | 231 | 98 | 2 |
| 432 | 37.21 | -2.64 | GBIF | 12.97 | 73.67 | 22.73 | 19.97 | 133 | 3 | 83.42 | 259 | 18 | 3 |
| 433 | 37.28 | -2.61 | GBIF | 12.57 | 72.69 | 22.68 | 19.97 | 147 | 4 | 84.51 | 178 | 22 | 6 |
| 434 | 37.72 | -3.31 | GBIF | 11.38 | 68.94 | 23.12 | 20.40 | 207 | 9 | 95.28 | 184 | 36 | 16 |
| 435 | 38.74 | -3.05 | GBIF | 10.63 | 69.90 | 26.43 | 23.45 | 174 | 25 | 67.45 | 314 | 86 | 7 |
| 436 | 36.57 | -0.93 | GBIF | 13.08 | 75.19 | 16.62 | 14.03 | 239 | 39 | 70.61 | 378 | 134 | 12 |
| 437 | 38.19 | -2.86 | GBIF | 10.34 | 67.59 | 23.55 | 20.63 | 157 | 5 | 95.15 | 274 | 22 | 1 |
| 438 | 37.97 | -2.92 | GBIF | 10.87 | 67.92 | 23.85 | 20.45 | 129 | 3 | 95.33 | 131 | 13 | 3 |
| 439 | 35.26 | -1.50 | GBIF | 12.74 | 81.16 | 20.45 | 18.47 | 157 | 23 | 50.20 | 362 | 105 | 10 |
| 440 | 38.26 | -3.53 | GBIF | 11.00 | 68.32 | 22.78 | 19.82 | 133 | 10 | 76.17 | 178 | 37 | 5 |
| 441 | 35.16 | -1.41 | GBIF | 12.85 | 81.85 | 20.53 | 18.93 | 157 | 28 | 44.45 | 366 | 129 | 4 |
| 442 | 35.11 | -1.42 | GBIF | 12.81 | 81.58 | 20.62 | 19.00 | 164 | 29 | 43.76 | 376 | 136 | 2 |
| 443 | 35.11 | -1.59 | GBIF | 12.66 | 81.67 | 20.43 | 19.02 | 154 | 26 | 46.22 | 294 | 118 | 9 |
| 444 | 35.04 | -1.51 | GBIF | 12.73 | 81.05 | 20.60 | 19.12 | 157 | 28 | 43.15 | 297 | 132 | 4 |
| 445 | 37.56 | 0.47 | GBIF | 13.11 | 82.44 | 23.18 | 22.82 | 148 | 4 | 97.78 | 208 | 16 | 10 |
| 446 | 35.10 | -1.50 | GBIF | 12.77 | 80.80 | 20.37 | 18.87 | 159 | 28 | 44.71 | 371 | 129 | 9 |
| 447 | 38.94 | -2.74 | GBIF | 10.07 | 69.01 | 26.20 | 23.77 | 153 | 22 | 70.48 | 268 | 74 | 2 |
| 448 | 36.98 | 0.02 | GBIF | 14.57 | 78.78 | 17.33 | 16.30 | 126 | 26 | 49.79 | 283 | 140 | 12 |
| 449 | 36.92 | 0.19 | GBIF | 14.82 | 79.28 | 18.18 | 17.20 | 146 | 19 | 55.79 | 307 | 162 | 3 |

| 450 | 36.81 | 0.16 | GBIF | 15.32 | 78.95 | 18.07 | 17.08 | 134 | 21 | 49.43 | 279 | 170 | 10 |
| --- | --- | --- | --- | --- | --- | --- | --- | --- | --- | --- | --- | --- | --- |
| 451 | 36.94 | 0.17 | GBIF | 15.06 | 78.84 | 17.97 | 17.00 | 132 | 18 | 52.97 | 281 | 164 | 3 |
| 452 | 36.82 | 0.07 | GBIF | 14.98 | 78.40 | 17.73 | 16.60 | 123 | 25 | 44.54 | 267 | 169 | 3 |
| 453 | 36.93 | 0.02 | GBIF | 14.38 | 78.98 | 17.50 | 16.40 | 126 | 27 | 48.27 | 284 | 184 | 12 |
| 454 | 37.16 | 0.04 | GBIF | 13.71 | 78.78 | 16.35 | 15.48 | 148 | 23 | 57.15 | 229 | 165 | 16 |
| 455 | 37.18 | -2.64 | GBIF | 12.92 | 73.39 | 22.72 | 19.98 | 132 | 3 | 82.71 | 173 | 18 | 3 |
| 456 | 38.82 | -2.67 | GBIF | 9.80 | 68.53 | 26.02 | 23.60 | 160 | 19 | 74.97 | 274 | 64 | 1 |
| 457 | 35.17 | -1.20 | GBIF | 13.05 | 83.12 | 19.72 | 18.20 | 182 | 38 | 40.62 | 303 | 173 | 11 |
| 458 | 37.14 | -2.70 | GBIF | 13.14 | 73.83 | 22.73 | 20.08 | 146 | 4 | 83.78 | 174 | 20 | 5 |
| 459 | 37.10 | -2.64 | GBIF | 13.07 | 74.24 | 22.88 | 20.27 | 129 | 2 | 82.88 | 174 | 15 | 4 |
| 460 | 37.17 | -2.68 | GBIF | 13.18 | 73.65 | 22.75 | 20.05 | 143 | 4 | 81.84 | 176 | 21 | 4 |
| 461 | 37.30 | 0.40 | GBIF | 12.66 | 83.28 | 21.53 | 20.15 | 211 | 14 | 88.19 | 336 | 46 | 9 |
| 462 | 35.33 | -1.13 | GBIF | 12.41 | 82.17 | 18.70 | 17.00 | 173 | 32 | 45.68 | 388 | 141 | 13 |
| 463 | 37.15 | -2.78 | GBIF | 12.10 | 70.76 | 21.60 | 19.02 | 160 | 8 | 79.89 | 188 | 34 | 4 |
| 464 | 37.11 | -2.62 | GBIF | 13.06 | 74.62 | 22.85 | 20.22 | 130 | 2 | 82.71 | 174 | 15 | 4 |
| 465 | 37.19 | -2.79 | GBIF | 12.07 | 70.57 | 21.35 | 18.73 | 175 | 10 | 79.74 | 200 | 42 | 3 |
| 466 | 35.30 | -1.14 | GBIF | 12.35 | 82.89 | 18.80 | 17.17 | 182 | 35 | 44.54 | 403 | 152 | 8 |
| 467 | 35.23 | -1.59 | GBIF | 12.67 | 81.72 | 20.45 | 18.58 | 164 | 24 | 51.20 | 372 | 108 | 9 |
| 468 | 37.06 | -2.62 | GBIF | 12.77 | 73.84 | 22.87 | 20.28 | 126 | 2 | 82.11 | 174 | 15 | 4 |
| 469 | 34.97 | -1.57 | GBIF | 12.49 | 81.11 | 21.08 | 19.55 | 167 | 27 | 45.09 | 294 | 127 | 8 |
| 470 | 37.39 | 0.38 | GBIF | 14.29 | 85.07 | 22.63 | 22.45 | 171 | 10 | 89.53 | 278 | 275 | 6 |
| 471 | 35.05 | -1.25 | GBIF | 12.66 | 82.20 | 20.18 | 18.70 | 179 | 38 | 39.41 | 298 | 175 | 5 |
| 472 | 34.90 | -1.47 | GBIF | 12.57 | 82.19 | 21.00 | 19.48 | 171 | 32 | 41.49 | 302 | 149 | 7 |
| 473 | 37.08 | -2.65 | GBIF | 12.93 | 74.33 | 22.88 | 20.28 | 127 | 2 | 82.32 | 174 | 15 | 4 |
| 474 | 37.78 | -2.49 | GBIF | 10.35 | 67.65 | 21.17 | 18.43 | 192 | 4 | 93.30 | 310 | 21 | 1 |
| 475 | 37.00 | -2.61 | GBIF | 12.27 | 73.07 | 22.73 | 20.20 | 124 | 1 | 82.03 | 179 | 12 | 13 |
| 476 | 35.03 | -1.50 | GBIF | 12.72 | 81.57 | 20.83 | 19.37 | 155 | 28 | 42.38 | 294 | 134 | 4 |
| 477 | 35.19 | -1.48 | GBIF | 12.93 | 81.29 | 20.33 | 18.73 | 152 | 25 | 46.60 | 358 | 116 | 4 |
| 478 | 36.90 | 0.04 | GBIF | 14.70 | 78.61 | 17.75 | 16.63 | 131 | 27 | 47.25 | 288 | 180 | 3 |
| 479 | 38.47 | -3.29 | GBIF | 9.46 | 68.05 | 21.58 | 19.13 | 177 | 13 | 80.14 | 342 | 49 | 10 |
| 480 | 38.48 | -3.32 | GBIF | 9.78 | 68.90 | 23.02 | 20.50 | 143 | 10 | 78.23 | 289 | 43 | 10 |
| 481 | 38.78 | -3.24 | GBIF | 10.56 | 70.39 | 25.67 | 22.72 | 149 | 22 | 65.92 | 287 | 77 | 7 |
| 482 | 38.53 | -3.27 | GBIF | 10.44 | 69.61 | 24.25 | 21.85 | 148 | 14 | 73.19 | 290 | 55 | 20 |
| 483 | 38.60 | -3.26 | GBIF | 10.40 | 69.80 | 25.03 | 22.10 | 157 | 18 | 69.58 | 299 | 69 | 1 |
| 484 | 38.76 | -3.30 | GBIF | 10.67 | 70.64 | 25.50 | 22.60 | 140 | 21 | 65.10 | 275 | 92 | 2 |
| 485 | 35.13 | -1.59 | GBIF | 12.68 | 81.30 | 20.52 | 19.07 | 154 | 24 | 47.61 | 363 | 112 | 12 |
| 486 | 35.08 | -1.55 | GBIF | 12.68 | 81.30 | 20.48 | 18.78 | 154 | 27 | 44.43 | 294 | 123 | 4 |
| 487 | 37.29 | 0.33 | GBIF | 12.18 | 82.32 | 17.58 | 17.12 | 179 | 22 | 76.91 | 262 | 94 | 8 |
| 488 | 37.61 | 0.41 | GBIF | 13.49 | 80.79 | 23.20 | 22.90 | 167 | 4 | 100.24 | 230 | 261 | 10 |
| 489 | 35.27 | -1.72 | GBIF | 12.21 | 80.32 | 19.52 | 17.60 | 162 | 22 | 54.94 | 380 | 90 | 5 |
| 490 | 35.07 | -1.56 | GBIF | 12.70 | 81.41 | 20.58 | 19.15 | 155 | 26 | 44.73 | 293 | 122 | 14 |
| 491 | 35.03 | -1.43 | GBIF | 12.61 | 81.34 | 20.50 | 18.97 | 163 | 30 | 41.50 | 298 | 144 | 4 |
| 492 | 37.73 | 0.52 | GBIF | 13.01 | 81.81 | 23.27 | 22.75 | 158 | 3 | 108.05 | 209 | 11 | 6 |
| 493 | 37.36 | -2.70 | GBIF | 13.14 | 73.42 | 22.63 | 19.90 | 157 | 6 | 83.06 | 184 | 29 | 6 |
| 494 | 38.55 | -3.19 | GBIF | 10.35 | 69.93 | 25.08 | 22.15 | 168 | 16 | 72.20 | 315 | 66 | 5 |
| 495 | 35.10 | -1.47 | GBIF | 12.90 | 81.65 | 20.53 | 19.02 | 153 | 27 | 43.66 | 364 | 128 | 2 |
| 496 | 35.27 | -1.58 | GBIF | 12.50 | 80.65 | 19.82 | 18.22 | 159 | 23 | 51.67 | 369 | 101 | 13 |
| 497 | 36.87 | -0.22 | GBIF | 13.84 | 79.10 | 16.85 | 15.43 | 132 | 28 | 49.18 | 295 | 117 | 13 |
| 498 | 35.02 | -1.49 | GBIF | 12.68 | 81.25 | 20.73 | 19.23 | 159 | 29 | 42.14 | 295 | 137 | 10 |
| 499 | 36.87 | 0.15 | GBIF | 15.27 | 78.33 | 18.17 | 17.22 | 143 | 21 | 52.39 | 299 | 164 | 3 |

| 500 | 35.08 | -1.31 | GBIF | 13.00 | 81.76 | 20.30 | 18.73 | 169 | 33 | 41.34 | 292 | 155 | 5 |
| --- | --- | --- | --- | --- | --- | --- | --- | --- | --- | --- | --- | --- | --- |
| 501 | 38.97 | -2.72 | GBIF | 9.91 | 68.81 | 26.25 | 23.87 | 152 | 22 | 69.98 | 260 | 73 | 2 |
| 502 | 38.68 | -3.28 | GBIF | 10.51 | 70.06 | 25.30 | 22.35 | 151 | 19 | 67.80 | 291 | 74 | 1 |
| 503 | 38.80 | -2.68 | GBIF | 9.76 | 68.72 | 25.87 | 23.47 | 162 | 19 | 76.97 | 281 | 62 | 1 |
| 504 | 37.25 | -2.78 | GBIF | 12.48 | 71.29 | 21.57 | 18.97 | 179 | 11 | 79.91 | 198 | 45 | 4 |
| 505 | 37.35 | -2.73 | GBIF | 13.23 | 73.11 | 22.45 | 19.80 | 171 | 8 | 82.21 | 191 | 36 | 6 |
| 506 | 35.02 | -1.32 | GBIF | 12.50 | 81.70 | 20.22 | 18.68 | 169 | 36 | 39.22 | 299 | 163 | 3 |
| 507 | 34.81 | -1.44 | GBIF | 12.32 | 82.11 | 20.98 | 19.60 | 184 | 37 | 40.24 | 311 | 165 | 9 |
| 508 | 37.69 | 0.53 | GBIF | 13.48 | 81.67 | 23.40 | 22.90 | 147 | 3 | 105.72 | 197 | 12 | 6 |
| 509 | 39.30 | -3.08 | GBIF | 9.76 | 70.20 | 26.42 | 24.28 | 121 | 24 | 55.82 | 204 | 106 | 3 |
| 510 | 38.66 | -3.39 | GBIF | 10.88 | 70.62 | 24.72 | 22.23 | 135 | 17 | 65.93 | 271 | 71 | 5 |
| 511 | 38.79 | -3.38 | GBIF | 10.69 | 70.81 | 25.53 | 22.60 | 136 | 22 | 63.98 | 269 | 89 | 4 |
| 512 | 38.65 | -3.24 | GBIF | 10.42 | 70.44 | 25.27 | 22.37 | 161 | 18 | 70.01 | 301 | 71 | 4 |
| 513 | 38.55 | -3.15 | GBIF | 10.43 | 70.02 | 25.18 | 22.27 | 177 | 18 | 71.82 | 327 | 72 | 1 |
| 514 | 39.52 | -4.20 | GBIF | 8.32 | 66.01 | 25.68 | 27.45 | 265 | 20 | 70.13 | 110 | 234 | 11 |
| 515 | 37.06 | -2.63 | GBIF | 12.92 | 74.23 | 22.88 | 20.33 | 128 | 2 | 82.52 | 175 | 14 | 4 |
| 516 | 38.80 | -2.62 | GBIF | 9.79 | 68.47 | 26.03 | 23.62 | 160 | 18 | 76.93 | 269 | 61 | 2 |
| 517 | 35.28 | -1.48 | GBIF | 12.87 | 81.43 | 20.60 | 18.65 | 152 | 23 | 49.57 | 355 | 105 | 2 |
| 518 | 35.29 | -1.59 | GBIF | 12.46 | 80.90 | 20.00 | 18.03 | 163 | 22 | 53.18 | 373 | 99 | 9 |
| 519 | 38.74 | -3.35 | GBIF | 10.75 | 70.72 | 25.40 | 22.47 | 141 | 20 | 65.91 | 275 | 72 | 3 |
| 520 | 34.89 | -1.28 | GBIF | 11.41 | 80.91 | 18.50 | 16.97 | 227 | 48 | 40.13 | 472 | 207 | 4 |
| 521 | 34.95 | -1.34 | GBIF | 12.36 | 81.30 | 20.17 | 18.60 | 181 | 38 | 39.24 | 310 | 171 | 18 |
| 522 | 34.91 | -1.52 | GBIF | 12.57 | 81.60 | 21.05 | 19.53 | 173 | 31 | 43.23 | 301 | 141 | 8 |
| 523 | 34.86 | -1.47 | GBIF | 12.63 | 81.98 | 21.13 | 19.67 | 173 | 33 | 40.73 | 305 | 154 | 7 |
| 524 | 38.72 | -3.33 | GBIF | 10.69 | 70.34 | 25.40 | 22.45 | 147 | 20 | 66.11 | 284 | 74 | 3 |
| 525 | 35.07 | -1.60 | GBIF | 12.81 | 81.58 | 20.68 | 19.35 | 156 | 25 | 45.56 | 292 | 120 | 5 |
| 526 | 35.18 | -1.40 | GBIF | 12.86 | 81.90 | 20.25 | 18.68 | 159 | 27 | 45.04 | 369 | 129 | 11 |
| 527 | 35.03 | -1.35 | GBIF | 12.66 | 81.67 | 20.20 | 18.67 | 171 | 34 | 40.48 | 299 | 158 | 3 |
| 528 | 38.21 | 0.18 | GBIF | 11.55 | 77.52 | 24.43 | 22.80 | 164 | 1 | 121.51 | 198 | 3 | 2 |
| 529 | 38.80 | -3.28 | GBIF | 10.68 | 70.75 | 25.60 | 22.70 | 146 | 20 | 66.52 | 281 | 91 | 7 |
| 530 | 37.36 | -2.68 | GBIF | 13.05 | 72.91 | 22.65 | 19.90 | 154 | 6 | 83.81 | 183 | 26 | 4 |
| 531 | 37.74 | 0.43 | GBIF | 12.80 | 81.01 | 22.90 | 22.48 | 195 | 3 | 106.84 | 263 | 11 | 10 |
| 532 | 38.84 | -3.37 | GBIF | 10.75 | 70.72 | 25.10 | 22.65 | 133 | 21 | 63.23 | 266 | 90 | 7 |
| 533 | 37.30 | -2.61 | GBIF | 12.59 | 72.78 | 22.70 | 20.05 | 143 | 4 | 84.19 | 176 | 21 | 6 |
| 534 | 36.84 | 0.49 | GBIF | 14.80 | 82.68 | 19.62 | 18.63 | 184 | 21 | 67.13 | 377 | 153 | 6 |
| 535 | 38.96 | -3.24 | GBIF | 10.37 | 70.05 | 25.33 | 22.98 | 137 | 22 | 63.52 | 262 | 96 | 11 |
| 536 | 34.84 | -1.24 | GBIF | 11.64 | 80.84 | 19.22 | 17.75 | 223 | 50 | 37.85 | 341 | 223 | 9 |
| 537 | 38.41 | -3.30 | GBIF | 9.62 | 68.20 | 23.05 | 20.53 | 137 | 8 | 81.06 | 275 | 33 | 14 |
| 538 | 36.65 | 0.52 | GBIF | 15.21 | 82.21 | 18.03 | 17.48 | 116 | 16 | 53.23 | 239 | 173 | 6 |
| 539 | 38.56 | -3.20 | GBIF | 10.26 | 69.78 | 25.03 | 22.10 | 169 | 16 | 72.65 | 316 | 64 | 5 |
| 540 | 37.39 | -2.70 | GBIF | 13.16 | 73.10 | 22.65 | 19.90 | 159 | 7 | 83.46 | 185 | 30 | 7 |
| 541 | 38.72 | -3.56 | GBIF | 10.23 | 69.14 | 24.28 | 21.87 | 126 | 16 | 64.52 | 169 | 77 | 10 |
| 542 | 36.80 | 0.67 | GBIF | 14.76 | 83.85 | 18.82 | 17.78 | 141 | 18 | 59.94 | 298 | 142 | 7 |
| 543 | 38.03 | -3.06 | GBIF | 10.83 | 68.57 | 23.75 | 20.82 | 130 | 4 | 90.08 | 136 | 17 | 2 |
| 544 | 39.93 | -3.26 | GBIF | 7.28 | 66.74 | 24.70 | 25.73 | 215 | 11 | 69.96 | 154 | 214 | 3 |
| 545 | 35.17 | -1.59 | GBIF | 12.71 | 81.99 | 20.43 | 18.95 | 153 | 24 | 48.20 | 361 | 110 | 8 |
| 546 | 38.96 | -3.31 | GBIF | 10.50 | 70.00 | 25.30 | 22.92 | 133 | 23 | 61.63 | 256 | 96 | 2 |
| 547 | 35.08 | -1.46 | GBIF | 12.83 | 81.22 | 20.58 | 19.05 | 156 | 28 | 43.15 | 368 | 133 | 9 |
| 548 | 39.92 | -3.27 | GBIF | 7.32 | 65.92 | 24.78 | 25.82 | 217 | 11 | 70.10 | 157 | 176 | 3 |
| 549 | 37.51 | 0.76 | GBIF | 12.62 | 84.11 | 23.77 | 22.27 | 131 | 4 | 100.39 | 208 | 15 | 2 |

| 550 | 36.17 | -0.32 | GBIF | 14.82 | 81.01 | 17.05 | 17.17 | 123 | 24 | 42.38 | 211 | 221 | 33 |
| --- | --- | --- | --- | --- | --- | --- | --- | --- | --- | --- | --- | --- | --- |
| 551 | 35.36 | -1.58 | GBIF | 12.17 | 80.57 | 18.65 | 16.95 | 184 | 25 | 54.15 | 408 | 105 | 4 |
| 552 | 37.27 | -2.69 | GBIF | 13.09 | 73.14 | 22.57 | 19.85 | 149 | 6 | 82.36 | 178 | 26 | 9 |
| 553 | 37.37 | -2.77 | GBIF | 12.83 | 72.10 | 22.08 | 19.37 | 184 | 10 | 81.37 | 199 | 43 | 6 |
| 554 | 38.72 | -2.86 | GBIF | 10.28 | 69.90 | 25.45 | 23.00 | 185 | 23 | 73.38 | 326 | 80 | 2 |
| 555 | 38.66 | -2.81 | GBIF | 10.40 | 69.80 | 25.42 | 22.97 | 194 | 22 | 76.68 | 337 | 74 | 2 |
| 556 | 38.71 | -2.94 | GBIF | 10.55 | 70.33 | 25.48 | 23.02 | 184 | 24 | 70.77 | 329 | 86 | 3 |
| 557 | 37.26 | -2.68 | GBIF | 12.83 | 72.92 | 22.55 | 19.78 | 140 | 4 | 82.20 | 176 | 23 | 5 |
| 558 | 35.21 | -1.30 | GBIF | 12.89 | 82.11 | 19.97 | 18.38 | 164 | 29 | 44.87 | 379 | 134 | 3 |
| 559 | 37.30 | -2.79 | GBIF | 12.59 | 71.54 | 21.68 | 19.05 | 190 | 11 | 80.32 | 202 | 49 | 5 |
| 560 | 36.83 | 0.31 | GBIF | 15.03 | 80.35 | 18.18 | 17.22 | 131 | 17 | 54.00 | 269 | 168 | 2 |
| 561 | 37.22 | -2.66 | GBIF | 13.05 | 73.31 | 22.72 | 19.93 | 137 | 3 | 82.87 | 175 | 19 | 4 |
| 562 | 37.29 | -2.70 | GBIF | 13.09 | 73.14 | 22.57 | 19.85 | 156 | 6 | 83.58 | 178 | 27 | 13 |
| 563 | 37.27 | -2.79 | GBIF | 12.30 | 71.51 | 21.45 | 18.82 | 188 | 12 | 79.17 | 204 | 51 | 5 |
| 564 | 37.33 | -2.61 | GBIF | 12.76 | 72.90 | 22.68 | 19.98 | 146 | 4 | 84.35 | 179 | 21 | 12 |
| 565 | 37.39 | -2.63 | GBIF | 13.16 | 73.10 | 22.45 | 19.73 | 151 | 6 | 82.63 | 190 | 28 | 5 |
| 566 | 37.26 | -2.36 | GBIF | 11.30 | 70.63 | 22.42 | 19.70 | 125 | 2 | 83.46 | 252 | 17 | 11 |
| 567 | 34.92 | -1.23 | GBIF | 11.29 | 81.24 | 18.92 | 17.32 | 216 | 48 | 38.00 | 337 | 215 | 3 |
| 568 | 36.56 | 0.68 | GBIF | 14.63 | 82.16 | 17.98 | 17.47 | 109 | 19 | 49.52 | 240 | 202 | 11 |
| 569 | 38.25 | -3.78 | GBIF | 11.09 | 68.47 | 23.67 | 20.67 | 112 | 8 | 74.81 | 193 | 34 | 11 |
| 570 | 37.34 | -2.72 | GBIF | 13.30 | 73.48 | 22.53 | 19.87 | 169 | 8 | 82.95 | 187 | 33 | 9 |
| 571 | 37.33 | -2.68 | GBIF | 12.97 | 73.26 | 22.57 | 19.83 | 154 | 6 | 83.36 | 182 | 27 | 4 |
| 572 | 37.49 | -2.73 | GBIF | 12.96 | 72.39 | 22.22 | 19.47 | 174 | 8 | 83.68 | 205 | 36 | 11 |
| 573 | 37.61 | 0.57 | GBIF | 13.52 | 82.92 | 23.53 | 23.02 | 127 | 3 | 104.13 | 173 | 13 | 14 |
| 574 | 37.44 | 0.21 | GBIF | 13.56 | 83.18 | 18.68 | 17.83 | 276 | 13 | 95.31 | 370 | 44 | 6 |
| 575 | 37.99 | -3.23 | GBIF | 11.34 | 68.74 | 22.88 | 19.88 | 169 | 9 | 87.13 | 174 | 31 | 3 |
| 576 | 35.21 | -1.09 | GBIF | 12.72 | 82.58 | 19.62 | 18.03 | 175 | 41 | 39.37 | 398 | 179 | 5 |
| 577 | 35.03 | -1.23 | GBIF | 12.29 | 81.94 | 19.58 | 18.10 | 196 | 41 | 39.38 | 314 | 190 | 11 |
| 578 | 35.20 | -1.57 | GBIF | 12.71 | 81.46 | 20.20 | 18.70 | 159 | 24 | 49.22 | 367 | 112 | 11 |
| 579 | 38.24 | -2.30 | GBIF | 9.58 | 66.55 | 24.12 | 21.53 | 174 | 2 | 111.75 | 229 | 9 | 1 |
| 580 | 36.72 | -0.46 | GBIF | 11.11 | 78.78 | 11.13 | 10.43 | 196 | 39 | 38.49 | 385 | 310 | 4 |
| 581 | 36.94 | 0.01 | GBIF | 14.43 | 78.87 | 17.55 | 16.45 | 126 | 28 | 48.23 | 286 | 186 | 16 |
| 582 | 38.75 | -3.36 | GBIF | 10.70 | 70.39 | 25.40 | 22.47 | 138 | 20 | 64.45 | 273 | 75 | 10 |
| 583 | 38.60 | -3.37 | GBIF | 10.69 | 69.88 | 24.92 | 22.02 | 142 | 19 | 67.02 | 280 | 70 | 11 |
| 584 | 38.83 | -2.98 | GBIF | 10.23 | 69.61 | 25.38 | 22.98 | 166 | 25 | 67.41 | 304 | 103 | 3 |
| 585 | 38.19 | 0.11 | GBIF | 10.32 | 75.36 | 24.23 | 22.73 | 163 | 0 | 122.61 | 196 | 1 | 4 |
| 586 | 38.21 | -3.02 | GBIF | 10.08 | 69.01 | 23.10 | 20.20 | 149 | 6 | 90.08 | 278 | 26 | 1 |
| 587 | 35.11 | -1.46 | GBIF | 12.93 | 81.86 | 20.57 | 19.02 | 156 | 28 | 44.32 | 367 | 127 | 9 |
| 588 | 37.40 | -2.81 | GBIF | 11.85 | 70.12 | 20.85 | 18.15 | 203 | 14 | 76.88 | 237 | 65 | 7 |
| 589 | 36.82 | -1.37 | GBIF | 11.92 | 72.66 | 20.43 | 17.95 | 166 | 14 | 75.97 | 281 | 59 | 10 |
| 590 | 38.23 | -3.54 | GBIF | 11.09 | 68.89 | 22.97 | 20.00 | 130 | 8 | 77.62 | 179 | 32 | 6 |
| 591 | 39.43 | -4.29 | GBIF | 7.77 | 64.19 | 24.77 | 26.60 | 261 | 17 | 68.53 | 104 | 244 | 17 |
| 592 | 38.60 | -3.36 | GBIF | 10.69 | 69.88 | 24.82 | 21.90 | 144 | 18 | 67.66 | 282 | 68 | 15 |
| 593 | 37.75 | -3.55 | GBIF | 10.37 | 68.65 | 24.20 | 21.37 | 100 | 5 | 81.77 | 158 | 18 | 16 |
| 594 | 36.77 | -0.43 | GBIF | 10.88 | 79.96 | 12.22 | 11.48 | 222 | 42 | 47.90 | 424 | 270 | 0 |
| 595 | 38.86 | -3.35 | GBIF | 10.81 | 70.64 | 25.15 | 22.72 | 135 | 20 | 63.27 | 262 | 92 | 11 |
| 596 | 35.18 | -1.50 | GBIF | 12.89 | 81.08 | 20.40 | 18.83 | 151 | 25 | 46.27 | 359 | 117 | 2 |
| 597 | 39.41 | -4.25 | GBIF | 8.05 | 64.92 | 23.88 | 25.65 | 221 | 21 | 59.09 | 116 | 229 | 8 |
| 598 | 35.13 | -1.40 | GBIF | 12.86 | 81.90 | 20.58 | 19.02 | 161 | 28 | 44.10 | 371 | 134 | 3 |
| 599 | 37.43 | 0.68 | GBIF | 12.59 | 83.94 | 23.22 | 21.75 | 150 | 6 | 95.09 | 252 | 20 | 10 |

| 600 | 34.91 | -1.40 | GBIF | 12.41 | 81.63 | 20.30 | 18.78 | 179 | 36 | 39.92 | 312 | 164 | 6 |
| --- | --- | --- | --- | --- | --- | --- | --- | --- | --- | --- | --- | --- | --- |
| 601 | 38.59 | -2.97 | GBIF | 10.40 | 69.80 | 25.37 | 22.33 | 203 | 23 | 72.57 | 358 | 89 | 2 |
| 602 | 35.06 | -1.35 | GBIF | 12.78 | 81.42 | 20.08 | 18.52 | 171 | 33 | 41.34 | 391 | 154 | 3 |
| 603 | 37.24 | 0.08 | GBIF | 13.77 | 78.71 | 16.02 | 15.75 | 134 | 20 | 56.80 | 208 | 162 | 18 |
| 604 | 37.24 | -2.72 | GBIF | 13.28 | 73.39 | 22.58 | 19.93 | 159 | 6 | 82.65 | 180 | 28 | 4 |
| 605 | 35.23 | -1.61 | GBIF | 12.61 | 81.87 | 20.20 | 18.37 | 171 | 24 | 52.08 | 385 | 107 | 14 |
| 606 | 37.49 | 0.70 | GBIF | 12.18 | 83.45 | 22.10 | 21.65 | 154 | 5 | 96.78 | 248 | 16 | 4 |
| 607 | 37.30 | -2.70 | GBIF | 13.04 | 72.86 | 22.53 | 19.83 | 152 | 6 | 82.45 | 179 | 28 | 12 |
| 608 | 37.29 | -2.67 | GBIF | 12.48 | 72.16 | 22.42 | 19.67 | 145 | 5 | 83.25 | 176 | 24 | 10 |
| 609 | 37.40 | -2.67 | GBIF | 13.12 | 73.28 | 22.67 | 19.95 | 153 | 6 | 83.26 | 185 | 27 | 6 |
| 610 | 35.29 | -1.33 | GBIF | 13.01 | 82.33 | 19.72 | 17.82 | 161 | 27 | 47.05 | 373 | 121 | 3 |
| 611 | 34.85 | -1.42 | GBIF | 12.06 | 80.93 | 20.55 | 19.03 | 181 | 37 | 39.26 | 311 | 169 | 6 |
| 612 | 35.15 | -1.51 | GBIF | 12.86 | 80.87 | 20.35 | 18.80 | 159 | 27 | 46.38 | 369 | 122 | 2 |
| 613 | 35.10 | -1.23 | GBIF | 13.02 | 82.38 | 19.85 | 18.35 | 176 | 37 | 40.11 | 301 | 170 | 4 |
| 614 | 37.40 | -2.64 | GBIF | 13.14 | 73.01 | 22.50 | 19.77 | 155 | 6 | 83.39 | 189 | 28 | 5 |
| 615 | 37.37 | -2.72 | GBIF | 13.24 | 73.56 | 22.55 | 19.87 | 168 | 8 | 82.59 | 188 | 34 | 14 |
| 616 | 37.20 | -2.65 | GBIF | 13.11 | 73.64 | 22.75 | 19.98 | 142 | 3 | 83.62 | 175 | 19 | 4 |
| 617 | 37.78 | 0.78 | GBIF | 12.43 | 82.89 | 23.28 | 22.63 | 143 | 2 | 111.06 | 194 | 8 | 7 |
| 618 | 36.94 | 0.06 | GBIF | 14.55 | 78.65 | 17.50 | 16.45 | 129 | 24 | 49.90 | 282 | 148 | 5 |
| 619 | 37.57 | 0.76 | GBIF | 12.38 | 83.67 | 22.95 | 22.37 | 129 | 3 | 102.31 | 195 | 14 | 11 |
| 620 | 37.26 | -2.75 | GBIF | 12.95 | 72.35 | 22.05 | 19.43 | 173 | 9 | 81.47 | 191 | 37 | 5 |
| 621 | 37.88 | -3.21 | GBIF | 11.89 | 69.54 | 23.37 | 20.35 | 186 | 8 | 92.05 | 173 | 29 | 8 |
| 622 | 37.02 | -2.43 | GBIF | 11.18 | 70.34 | 21.77 | 19.17 | 151 | 3 | 79.46 | 302 | 20 | 3 |
| 623 | 36.98 | -0.23 | GBIF | 14.11 | 78.38 | 17.35 | 15.95 | 146 | 24 | 57.67 | 330 | 94 | 11 |
| 624 | 36.88 | -0.24 | GBIF | 13.78 | 78.27 | 17.15 | 15.78 | 134 | 28 | 49.85 | 301 | 113 | 15 |
| 625 | 37.41 | 0.56 | GBIF | 13.68 | 84.41 | 24.12 | 22.78 | 137 | 7 | 91.61 | 223 | 23 | 7 |
| 626 | 37.23 | -2.60 | GBIF | 12.78 | 73.47 | 22.75 | 20.03 | 145 | 3 | 84.25 | 176 | 19 | 6 |
| 627 | 35.31 | -1.59 | GBIF | 12.33 | 81.09 | 19.45 | 17.83 | 171 | 24 | 52.37 | 386 | 105 | 6 |
| 628 | 37.22 | -2.78 | GBIF | 12.20 | 70.93 | 21.35 | 18.75 | 182 | 11 | 80.01 | 202 | 46 | 4 |
| 629 | 36.75 | 0.66 | GBIF | 15.01 | 83.38 | 18.68 | 17.67 | 130 | 18 | 56.23 | 280 | 156 | 6 |
| 630 | 37.23 | 0.02 | GBIF | 12.19 | 77.16 | 14.93 | 13.98 | 166 | 26 | 57.80 | 269 | 160 | 7 |
| 631 | 38.37 | 0.10 | GBIF | 10.38 | 75.79 | 24.97 | 23.25 | 150 | 0 | 126.13 | 165 | 0 | 2 |
| 632 | 35.03 | -1.51 | GBIF | 12.71 | 81.46 | 20.83 | 19.37 | 159 | 28 | 43.41 | 296 | 132 | 4 |
| 633 | 35.07 | -1.46 | GBIF | 12.75 | 81.21 | 20.63 | 19.10 | 159 | 29 | 42.96 | 290 | 136 | 2 |
| 634 | 35.39 | -1.70 | GBIF | 12.06 | 79.86 | 19.17 | 17.17 | 169 | 21 | 57.27 | 388 | 85 | 4 |
| 635 | 34.98 | -1.27 | GBIF | 12.13 | 81.38 | 19.70 | 18.13 | 187 | 41 | 38.40 | 313 | 184 | 11 |
| 636 | 38.20 | -2.85 | GBIF | 10.38 | 68.31 | 23.60 | 20.70 | 155 | 5 | 95.32 | 270 | 22 | 1 |
| 637 | 38.14 | -2.93 | GBIF | 10.83 | 69.00 | 23.60 | 20.53 | 140 | 4 | 94.02 | 146 | 18 | 4 |
| 638 | 37.77 | 0.60 | GBIF | 12.57 | 82.68 | 23.45 | 22.75 | 145 | 2 | 109.52 | 183 | 10 | 4 |
| 639 | 36.96 | -0.26 | GBIF | 13.71 | 77.89 | 17.55 | 16.10 | 143 | 26 | 56.45 | 333 | 97 | 12 |
| 640 | 37.28 | -2.71 | GBIF | 13.43 | 73.76 | 22.65 | 19.95 | 162 | 7 | 82.99 | 182 | 29 | 5 |
| 641 | 37.37 | 0.13 | GBIF | 13.19 | 81.43 | 14.80 | 15.43 | 169 | 23 | 65.31 | 261 | 131 | 16 |
| 642 | 37.47 | 0.41 | GBIF | 13.00 | 83.33 | 21.20 | 20.83 | 222 | 9 | 95.00 | 327 | 30 | 7 |
| 643 | 37.24 | -2.74 | GBIF | 13.11 | 73.23 | 22.33 | 19.67 | 163 | 7 | 82.13 | 185 | 31 | 5 |
| 644 | 35.13 | -1.51 | GBIF | 12.78 | 80.91 | 20.27 | 18.75 | 157 | 27 | 45.70 | 368 | 122 | 2 |
| 645 | 37.55 | 0.76 | GBIF | 12.50 | 83.89 | 22.87 | 22.30 | 133 | 4 | 100.38 | 202 | 15 | 5 |
| 646 | 37.24 | -2.64 | GBIF | 12.81 | 73.61 | 22.65 | 19.88 | 140 | 3 | 83.36 | 175 | 20 | 5 |
| 647 | 37.58 | 0.41 | GBIF | 13.23 | 81.69 | 23.13 | 22.83 | 162 | 4 | 98.95 | 228 | 255 | 14 |
| 648 | 37.30 | -2.64 | GBIF | 12.50 | 72.67 | 22.60 | 19.87 | 147 | 4 | 84.21 | 178 | 22 | 12 |
| 649 | 35.40 | -1.78 | GBIF | 11.88 | 80.24 | 17.70 | 15.77 | 191 | 22 | 60.67 | 426 | 84 | 5 |

| 650 | 34.85 | 1.56 | GBIF | 12.64 | 83.17 | 20.85 | 21.77 | 146 | 23 | 50.63 | 226 | 370 | 13 |
| --- | --- | --- | --- | --- | --- | --- | --- | --- | --- | --- | --- | --- | --- |
| 651 | 35.31 | -1.44 | GBIF | 12.92 | 81.75 | 20.27 | 18.30 | 158 | 24 | 50.48 | 364 | 106 | 3 |
| 652 | 37.28 | -2.63 | GBIF | 12.54 | 72.92 | 22.63 | 19.88 | 147 | 4 | 84.15 | 177 | 22 | 8 |
| 653 | 37.24 | -2.76 | GBIF | 12.88 | 72.33 | 22.02 | 19.38 | 172 | 9 | 81.28 | 191 | 37 | 5 |
| 654 | 37.04 | 0.02 | GBIF | 14.75 | 79.73 | 17.03 | 16.18 | 129 | 25 | 51.55 | 284 | 143 | 16 |
| 655 | 38.19 | -3.23 | GBIF | 10.71 | 69.09 | 23.33 | 20.43 | 150 | 6 | 87.15 | 167 | 24 | 4 |
| 656 | 35.05 | -1.48 | GBIF | 12.71 | 81.46 | 20.60 | 19.10 | 159 | 28 | 42.86 | 294 | 137 | 9 |
| 657 | 37.22 | -2.73 | GBIF | 13.08 | 73.09 | 22.42 | 19.75 | 157 | 6 | 82.01 | 182 | 28 | 4 |
| 658 | 37.57 | 0.69 | GBIF | 12.76 | 83.39 | 23.08 | 22.53 | 130 | 3 | 102.03 | 182 | 13 | 5 |
| 659 | 36.84 | -0.31 | GBIF | 12.82 | 78.68 | 15.85 | 14.18 | 172 | 41 | 49.50 | 385 | 160 | 15 |
| 660 | 38.61 | -3.01 | GBIF | 10.51 | 69.59 | 26.00 | 22.95 | 201 | 25 | 71.07 | 350 | 91 | 6 |
| 661 | 35.26 | -1.63 | GBIF | 12.52 | 81.81 | 20.17 | 18.32 | 159 | 22 | 52.81 | 372 | 97 | 7 |
| 662 | 37.87 | 0.72 | GBIF | 12.79 | 84.16 | 24.20 | 23.40 | 132 | 1 | 115.97 | 173 | 5 | 7 |
| 663 | 37.75 | 0.71 | GBIF | 12.12 | 82.43 | 22.78 | 22.10 | 162 | 3 | 108.19 | 224 | 11 | 4 |
| 664 | 35.39 | -1.08 | GBIF | 12.20 | 81.88 | 17.07 | 15.47 | 205 | 46 | 45.63 | 298 | 175 | 6 |
| 665 | 35.17 | -1.69 | GBIF | 12.34 | 81.20 | 20.35 | 18.58 | 157 | 23 | 51.31 | 370 | 100 | 5 |
| 666 | 37.74 | 0.65 | GBIF | 13.11 | 84.03 | 23.82 | 23.12 | 128 | 2 | 111.18 | 169 | 8 | 9 |
| 667 | 37.37 | -2.62 | GBIF | 13.02 | 73.13 | 22.48 | 19.80 | 154 | 5 | 83.17 | 188 | 26 | 5 |
| 668 | 37.23 | -2.78 | GBIF | 12.38 | 71.58 | 21.55 | 18.95 | 174 | 10 | 79.35 | 197 | 43 | 4 |
| 669 | 37.60 | 0.52 | GBIF | 13.60 | 82.42 | 23.47 | 23.02 | 138 | 4 | 101.23 | 185 | 14 | 6 |
| 670 | 35.39 | -1.56 | GBIF | 12.07 | 80.44 | 18.43 | 16.73 | 185 | 26 | 53.96 | 406 | 107 | 4 |
| 671 | 35.09 | -1.43 | GBIF | 12.77 | 81.37 | 20.62 | 19.03 | 162 | 29 | 43.64 | 374 | 136 | 9 |
| 672 | 35.12 | -1.55 | GBIF | 12.70 | 81.41 | 20.28 | 18.78 | 159 | 26 | 46.23 | 294 | 121 | 2 |
| 673 | 38.42 | -3.39 | GBIF | 9.45 | 67.02 | 22.67 | 19.67 | 158 | 15 | 74.03 | 188 | 59 | 11 |
| 674 | 37.66 | -2.93 | GBIF | 12.03 | 69.96 | 21.32 | 18.72 | 216 | 14 | 82.75 | 272 | 50 | 5 |
| 675 | 35.08 | -1.50 | GBIF | 12.78 | 81.42 | 20.40 | 18.88 | 162 | 28 | 44.55 | 377 | 130 | 3 |
| 676 | 37.37 | -2.78 | GBIF | 12.36 | 71.02 | 21.63 | 18.95 | 193 | 12 | 79.87 | 209 | 51 | 6 |
| 677 | 35.30 | -1.42 | GBIF | 12.88 | 82.01 | 19.93 | 17.95 | 158 | 25 | 48.54 | 367 | 112 | 3 |
| 678 | 37.37 | -2.80 | GBIF | 12.12 | 70.86 | 21.35 | 18.68 | 199 | 13 | 79.32 | 216 | 55 | 6 |
| 679 | 38.98 | -2.80 | GBIF | 10.12 | 69.77 | 26.15 | 23.78 | 151 | 23 | 68.40 | 269 | 77 | 3 |
| 680 | 38.87 | -2.70 | GBIF | 9.95 | 69.58 | 26.15 | 23.78 | 157 | 21 | 73.66 | 273 | 67 | 1 |
| 681 | 37.08 | -2.76 | GBIF | 12.79 | 72.68 | 22.45 | 19.92 | 152 | 4 | 83.62 | 178 | 23 | 5 |
| 682 | 38.68 | -3.31 | GBIF | 10.61 | 70.25 | 25.25 | 22.32 | 147 | 18 | 67.21 | 286 | 71 | 1 |
| 683 | 37.78 | 0.57 | GBIF | 12.19 | 82.38 | 23.17 | 22.50 | 156 | 2 | 110.16 | 202 | 10 | 5 |
| 684 | 35.11 | -1.39 | GBIF | 12.88 | 81.54 | 20.65 | 19.05 | 162 | 29 | 43.44 | 373 | 137 | 4 |
| 685 | 35.19 | -1.26 | GBIF | 12.98 | 82.17 | 19.88 | 18.30 | 164 | 32 | 42.64 | 294 | 147 | 4 |
| 686 | 38.99 | -2.69 | GBIF | 9.88 | 68.63 | 26.35 | 23.98 | 150 | 21 | 70.78 | 253 | 68 | 2 |
| 687 | 35.00 | -1.40 | GBIF | 12.60 | 81.82 | 20.23 | 18.75 | 168 | 33 | 40.48 | 304 | 154 | 8 |
| 688 | 36.86 | -0.25 | GBIF | 13.79 | 79.26 | 16.78 | 15.30 | 138 | 29 | 49.20 | 309 | 122 | 10 |
| 689 | 35.39 | -1.59 | GBIF | 12.17 | 80.57 | 19.08 | 17.10 | 177 | 23 | 55.57 | 397 | 96 | 6 |
| 690 | 37.50 | 0.66 | GBIF | 12.73 | 83.22 | 22.65 | 22.18 | 142 | 5 | 98.11 | 223 | 16 | 5 |
| 691 | 38.05 | -3.14 | GBIF | 10.91 | 69.04 | 23.28 | 20.38 | 162 | 6 | 91.75 | 160 | 22 | 2 |
| 692 | 38.91 | -2.70 | GBIF | 10.02 | 69.14 | 26.25 | 23.87 | 153 | 21 | 72.13 | 262 | 68 | 1 |
| 693 | 36.94 | 0.31 | GBIF | 14.55 | 80.83 | 18.87 | 17.95 | 163 | 19 | 61.51 | 329 | 153 | 4 |
| 694 | 39.85 | -3.26 | GBIF | 7.67 | 67.32 | 24.85 | 25.92 | 195 | 14 | 62.98 | 186 | 164 | 5 |
| 695 | 38.52 | -3.32 | GBIF | 10.49 | 69.94 | 24.45 | 21.53 | 145 | 14 | 72.73 | 285 | 55 | 10 |
| 696 | 35.01 | -1.55 | GBIF | 12.48 | 81.01 | 20.82 | 19.37 | 167 | 28 | 44.59 | 297 | 131 | 14 |
| 697 | 34.79 | 1.09 | GBIF | 12.46 | 79.35 | 16.18 | 18.05 | 160 | 24 | 48.57 | 145 | 416 | 22 |
| 698 | 37.83 | -0.15 | GBIF | 11.22 | 75.79 | 22.72 | 20.98 | 220 | 2 | 111.81 | 320 | 10 | 11 |
| 699 | 37.25 | -2.73 | GBIF | 13.22 | 73.43 | 22.50 | 19.82 | 157 | 7 | 82.04 | 181 | 30 | 5 |

| 700 | 38.11 | 0.86 | GBIF | 11.85 | 81.16 | 24.68 | 23.73 | 128 | 0 | 124.33 | 167 | 0 | 3 |
| --- | --- | --- | --- | --- | --- | --- | --- | --- | --- | --- | --- | --- | --- |
| 701 | 37.57 | 0.77 | GBIF | 12.76 | 84.49 | 23.08 | 22.52 | 129 | 3 | 103.74 | 195 | 13 | 7 |
| 702 | 37.91 | -0.10 | GBIF | 11.32 | 75.44 | 23.27 | 21.55 | 194 | 1 | 113.38 | 269 | 8 | 15 |
| 703 | 37.81 | -0.02 | GBIF | 11.44 | 76.79 | 22.13 | 20.65 | 268 | 6 | 103.96 | 374 | 22 | 11 |
| 704 | 39.95 | -3.22 | GBIF | 7.28 | 66.14 | 24.57 | 25.65 | 208 | 11 | 68.36 | 170 | 204 | 9 |
| 705 | 38.56 | -2.09 | GBIF | 9.32 | 67.51 | 25.33 | 22.87 | 175 | 5 | 106.26 | 221 | 16 | 1 |
| 706 | 38.17 | 0.17 | GBIF | 11.53 | 77.40 | 24.20 | 22.87 | 168 | 1 | 119.44 | 212 | 4 | 0 |
| 707 | 38.17 | 0.35 | GBIF | 11.88 | 79.75 | 23.78 | 22.48 | 183 | 1 | 119.43 | 227 | 3 | 4 |
| 708 | 35.19 | -1.55 | GBIF | 12.75 | 81.21 | 20.23 | 18.67 | 163 | 25 | 49.23 | 370 | 113 | 2 |
| 709 | 35.25 | -1.62 | GBIF | 12.55 | 81.49 | 20.05 | 18.20 | 161 | 22 | 52.85 | 374 | 99 | 10 |
| 710 | 37.43 | 0.65 | GBIF | 12.87 | 84.10 | 23.62 | 22.18 | 141 | 6 | 93.06 | 230 | 20 | 5 |
| 711 | 37.33 | -2.73 | GBIF | 13.26 | 73.25 | 22.38 | 19.72 | 172 | 8 | 82.31 | 192 | 34 | 6 |
| 712 | 37.30 | -2.31 | GBIF | 11.04 | 70.33 | 21.87 | 19.48 | 132 | 2 | 84.79 | 256 | 17 | 2 |
| 713 | 38.51 | -3.38 | GBIF | 10.43 | 69.56 | 24.20 | 21.28 | 138 | 14 | 70.87 | 276 | 57 | 7 |
| 714 | 37.65 | 0.40 | GBIF | 13.30 | 80.61 | 23.08 | 22.75 | 181 | 3 | 101.25 | 244 | 283 | 5 |
| 715 | 35.03 | -1.54 | GBIF | 12.63 | 80.98 | 20.78 | 19.30 | 157 | 27 | 43.91 | 294 | 128 | 7 |
| 716 | 38.34 | -2.73 | GBIF | 10.33 | 68.89 | 24.65 | 22.07 | 162 | 8 | 91.35 | 287 | 35 | 1 |
| 717 | 39.35 | -3.17 | GBIF | 9.48 | 69.73 | 25.98 | 23.92 | 117 | 22 | 55.84 | 193 | 105 | 2 |
| 718 | 36.86 | 0.16 | GBIF | 15.36 | 78.76 | 18.05 | 17.10 | 138 | 20 | 51.64 | 281 | 166 | 3 |
| 719 | 35.18 | -1.47 | GBIF | 12.93 | 81.34 | 20.33 | 18.73 | 154 | 26 | 46.44 | 361 | 118 | 3 |
| 720 | 38.12 | 0.12 | GBIF | 10.55 | 75.90 | 23.75 | 22.30 | 175 | 1 | 117.91 | 221 | 5 | 4 |
| 721 | 37.51 | 0.50 | GBIF | 13.07 | 82.75 | 22.90 | 22.67 | 155 | 5 | 94.98 | 223 | 242 | 6 |
| 722 | 37.51 | 0.54 | GBIF | 13.40 | 83.23 | 23.32 | 22.95 | 137 | 5 | 97.07 | 196 | 17 | 7 |
| 723 | 37.57 | 0.46 | GBIF | 13.23 | 82.14 | 23.23 | 22.87 | 148 | 4 | 98.70 | 207 | 15 | 5 |
| 724 | 35.44 | -1.49 | GBIF | 12.62 | 81.40 | 19.93 | 17.72 | 155 | 22 | 53.85 | 359 | 90 | 4 |
| 725 | 37.54 | 0.42 | GBIF | 12.59 | 80.72 | 22.82 | 22.53 | 165 | 5 | 95.74 | 230 | 260 | 11 |
| 726 | 37.60 | 0.43 | GBIF | 13.42 | 81.31 | 23.20 | 22.88 | 160 | 4 | 99.62 | 225 | 14 | 15 |
| 727 | 38.84 | -3.02 | GBIF | 10.12 | 69.29 | 26.05 | 23.13 | 166 | 27 | 65.89 | 300 | 90 | 2 |
| 728 | 37.56 | 0.44 | GBIF | 12.86 | 81.90 | 23.02 | 22.70 | 155 | 5 | 97.20 | 216 | 17 | 10 |
| 729 | 35.24 | -1.44 | GBIF | 12.92 | 81.75 | 20.40 | 18.80 | 152 | 26 | 46.70 | 357 | 115 | 7 |
| 730 | 37.57 | 0.56 | GBIF | 13.43 | 82.92 | 23.62 | 23.28 | 124 | 3 | 101.84 | 167 | 14 | 11 |
| 731 | 37.44 | 0.53 | GBIF | 13.99 | 84.29 | 23.15 | 22.88 | 141 | 7 | 91.26 | 223 | 222 | 6 |
| 732 | 37.49 | 0.50 | GBIF | 13.27 | 82.92 | 22.85 | 22.60 | 157 | 6 | 93.17 | 233 | 248 | 6 |
| 733 | 37.44 | 0.60 | GBIF | 13.46 | 84.11 | 23.28 | 22.98 | 129 | 5 | 93.97 | 200 | 20 | 10 |
| 734 | 37.23 | -2.69 | GBIF | 13.16 | 73.51 | 22.68 | 19.97 | 145 | 5 | 82.35 | 174 | 24 | 4 |
| 735 | 38.98 | -2.73 | GBIF | 9.93 | 68.98 | 26.28 | 23.90 | 151 | 23 | 70.34 | 261 | 73 | 2 |
| 736 | 37.20 | -2.61 | GBIF | 12.89 | 73.67 | 22.73 | 20.03 | 137 | 2 | 83.96 | 174 | 17 | 3 |
| 737 | 35.14 | -1.56 | GBIF | 12.69 | 81.36 | 20.18 | 18.70 | 161 | 25 | 47.33 | 373 | 117 | 4 |
| 738 | 38.11 | 0.28 | GBIF | 11.72 | 78.64 | 23.48 | 22.38 | 192 | 2 | 115.60 | 255 | 8 | 14 |
| 739 | 37.48 | 0.46 | GBIF | 12.90 | 83.23 | 22.43 | 22.20 | 172 | 7 | 92.92 | 262 | 273 | 11 |
| 740 | 37.92 | 0.64 | GBIF | 13.07 | 84.35 | 23.98 | 23.22 | 149 | 1 | 116.60 | 188 | 5 | 4 |
| 741 | 39.99 | -3.23 | GBIF | 7.20 | 66.06 | 24.60 | 25.65 | 230 | 10 | 72.56 | 191 | 230 | 10 |
| 742 | 37.05 | 0.05 | GBIF | 14.68 | 79.32 | 17.07 | 16.20 | 127 | 23 | 51.90 | 285 | 151 | 24 |
| 743 | 38.92 | -2.78 | GBIF | 10.13 | 69.41 | 26.05 | 23.68 | 156 | 22 | 70.91 | 281 | 74 | 3 |
| 744 | 38.91 | -2.74 | GBIF | 10.07 | 69.48 | 26.13 | 23.77 | 155 | 21 | 72.33 | 274 | 70 | 2 |
| 745 | 38.20 | 0.37 | GBIF | 11.70 | 79.59 | 23.90 | 22.60 | 176 | 1 | 120.32 | 213 | 3 | 12 |
| 746 | 38.97 | -2.65 | GBIF | 9.95 | 69.10 | 26.38 | 24.00 | 150 | 21 | 71.58 | 258 | 68 | 2 |
| 747 | 37.20 | -2.63 | GBIF | 12.91 | 73.76 | 22.70 | 19.97 | 132 | 3 | 82.90 | 259 | 18 | 3 |
| 748 | 35.21 | 1.74 | GBIF | 12.35 | 84.01 | 21.20 | 21.90 | 112 | 15 | 53.55 | 177 | 266 | 5 |
| 749 | 37.49 | 0.49 | GBIF | 13.09 | 82.86 | 22.63 | 22.42 | 165 | 7 | 93.07 | 250 | 261 | 10 |

| 750 | 38.03 | -2.85 | GBIF | 10.21 | 67.16 | 23.37 | 20.08 | 139 | 2 | 98.99 | 143 | 12 | 1 |
| --- | --- | --- | --- | --- | --- | --- | --- | --- | --- | --- | --- | --- | --- |
| 751 | 38.90 | -2.67 | GBIF | 10.02 | 69.56 | 26.33 | 23.92 | 152 | 21 | 73.44 | 268 | 67 | 1 |
| 752 | 37.41 | 0.41 | GBIF | 14.23 | 84.67 | 22.78 | 22.65 | 165 | 9 | 90.01 | 271 | 264 | 6 |
| 753 | 38.88 | -2.69 | GBIF | 10.05 | 69.31 | 26.27 | 23.87 | 155 | 21 | 73.26 | 268 | 68 | 1 |
| 754 | 37.32 | -0.18 | GBIF | 11.27 | 77.76 | 4.65 | 3.55 | 242 | 65 | 44.37 | 489 | 271 | 1 |
| 755 | 38.41 | -3.33 | GBIF | 9.30 | 67.39 | 21.33 | 18.77 | 182 | 15 | 80.38 | 233 | 54 | 10 |
| 756 | 36.62 | -0.22 | GBIF | 10.73 | 79.44 | 12.02 | 10.95 | 167 | 35 | 36.36 | 432 | 330 | 3 |
| 757 | 37.80 | 0.54 | GBIF | 12.09 | 81.70 | 23.03 | 22.35 | 170 | 2 | 109.73 | 218 | 10 | 6 |
| 758 | 35.02 | -1.26 | GBIF | 12.46 | 81.96 | 20.18 | 18.70 | 180 | 38 | 39.06 | 299 | 177 | 12 |
| 759 | 35.21 | -1.53 | GBIF | 12.75 | 81.21 | 20.18 | 18.62 | 160 | 25 | 48.84 | 368 | 113 | 10 |
| 760 | 36.98 | -0.33 | GBIF | 13.04 | 77.17 | 18.03 | 16.35 | 155 | 28 | 59.20 | 359 | 101 | 9 |
| 761 | 35.01 | -1.34 | GBIF | 12.50 | 81.70 | 20.28 | 18.77 | 173 | 35 | 40.26 | 298 | 161 | 3 |
| 762 | 38.21 | -3.16 | GBIF | 10.81 | 70.18 | 24.00 | 21.13 | 148 | 6 | 85.66 | 157 | 26 | 2 |
| 763 | 38.34 | -3.08 | GBIF | 10.38 | 69.63 | 24.73 | 21.78 | 177 | 15 | 78.53 | 309 | 56 | 5 |
| 764 | 37.26 | -2.65 | GBIF | 12.67 | 73.22 | 22.57 | 19.83 | 142 | 4 | 83.70 | 175 | 21 | 12 |
| 765 | 37.30 | -2.71 | GBIF | 13.47 | 73.59 | 22.63 | 19.97 | 164 | 7 | 82.60 | 184 | 31 | 8 |
| 766 | 38.25 | -3.02 | GBIF | 10.43 | 69.50 | 24.18 | 21.23 | 148 | 8 | 84.88 | 273 | 31 | 1 |
| 767 | 38.79 | -3.41 | GBIF | 10.59 | 70.14 | 24.98 | 22.55 | 131 | 20 | 63.99 | 261 | 84 | 3 |
| 768 | 37.69 | 0.48 | GBIF | 13.34 | 80.86 | 23.25 | 22.85 | 162 | 3 | 104.92 | 218 | 11 | 6 |
| 769 | 37.36 | -2.65 | GBIF | 13.05 | 73.31 | 22.65 | 19.95 | 158 | 5 | 84.88 | 181 | 25 | 4 |
| 770 | 35.33 | -1.40 | GBIF | 13.03 | 81.97 | 20.02 | 18.05 | 159 | 23 | 50.41 | 364 | 106 | 3 |
| 771 | 35.11 | -1.49 | GBIF | 12.80 | 81.01 | 20.38 | 18.88 | 156 | 28 | 44.56 | 369 | 127 | 9 |
| 772 | 36.79 | 0.72 | GBIF | 14.91 | 84.23 | 18.72 | 17.63 | 134 | 18 | 57.91 | 278 | 144 | 6 |
| 773 | 36.42 | 0.80 | GBIF | 13.42 | 82.31 | 18.43 | 18.05 | 122 | 21 | 51.12 | 280 | 240 | 8 |
| 774 | 36.78 | 0.65 | GBIF | 14.82 | 83.71 | 18.72 | 17.68 | 138 | 18 | 58.75 | 292 | 150 | 6 |
| 775 | 37.54 | 0.68 | GBIF | 12.68 | 83.39 | 22.87 | 22.37 | 134 | 4 | 99.46 | 197 | 14 | 5 |
| 776 | 34.97 | -1.33 | GBIF | 12.53 | 81.92 | 20.27 | 18.68 | 182 | 38 | 39.87 | 308 | 169 | 11 |
| 777 | 35.09 | -1.56 | GBIF | 12.70 | 81.41 | 20.50 | 19.05 | 156 | 27 | 45.56 | 292 | 120 | 7 |
| 778 | 36.95 | 0.31 | GBIF | 14.52 | 81.10 | 18.88 | 18.02 | 168 | 20 | 63.16 | 336 | 149 | 4 |
| 779 | 34.81 | -1.21 | GBIF | 11.71 | 81.88 | 19.98 | 18.55 | 209 | 51 | 36.60 | 327 | 223 | 9 |
| 780 | 36.96 | 0.21 | GBIF | 14.55 | 79.08 | 18.02 | 17.05 | 140 | 18 | 56.12 | 294 | 162 | 4 |
| 781 | 36.86 | 0.27 | GBIF | 14.86 | 80.32 | 18.02 | 17.02 | 129 | 16 | 53.67 | 266 | 165 | 2 |
| 782 | 36.73 | 0.62 | GBIF | 15.04 | 83.56 | 18.33 | 17.77 | 130 | 18 | 55.98 | 279 | 158 | 6 |
| 783 | 37.48 | 0.27 | GBIF | 13.19 | 82.45 | 19.65 | 19.00 | 273 | 10 | 97.33 | 367 | 36 | 6 |
| 784 | 39.83 | -3.39 | GBIF | 7.56 | 66.89 | 24.62 | 25.75 | 213 | 14 | 65.84 | 182 | 173 | 4 |
| 785 | 37.47 | 0.22 | GBIF | 13.43 | 82.87 | 18.60 | 17.78 | 282 | 13 | 95.58 | 381 | 45 | 5 |
| 786 | 36.72 | -0.58 | GBIF | 10.58 | 77.82 | 10.92 | 10.35 | 223 | 46 | 40.70 | 424 | 303 | 2 |
| 787 | 37.56 | 0.33 | GBIF | 13.24 | 81.74 | 22.48 | 22.23 | 204 | 5 | 97.09 | 277 | 320 | 17 |
| 788 | 37.72 | 0.54 | GBIF | 13.09 | 81.82 | 23.30 | 22.77 | 153 | 3 | 107.45 | 199 | 11 | 6 |
| 789 | 36.96 | 0.30 | GBIF | 14.48 | 80.91 | 18.83 | 17.92 | 169 | 19 | 63.67 | 253 | 149 | 5 |
| 790 | 36.81 | 0.21 | GBIF | 15.13 | 78.37 | 17.88 | 16.88 | 128 | 18 | 51.25 | 263 | 168 | 10 |
| 791 | 34.82 | -1.29 | GBIF | 11.72 | 80.80 | 20.02 | 18.52 | 207 | 45 | 38.71 | 327 | 202 | 10 |
| 792 | 34.83 | -1.24 | GBIF | 11.77 | 81.21 | 19.73 | 18.27 | 217 | 49 | 37.83 | 334 | 218 | 9 |
| 793 | 36.74 | 0.55 | GBIF | 14.98 | 83.24 | 18.37 | 17.42 | 118 | 17 | 53.83 | 247 | 162 | 9 |
| 794 | 37.19 | 0.40 | GBIF | 12.58 | 82.24 | 18.10 | 17.18 | 141 | 17 | 67.28 | 288 | 119 | 19 |
| 795 | 36.88 | 0.40 | GBIF | 14.72 | 81.76 | 19.15 | 18.18 | 172 | 19 | 64.45 | 341 | 155 | 8 |
| 796 | 39.33 | -4.32 | GBIF | 8.23 | 64.83 | 25.05 | 26.97 | 238 | 18 | 65.90 | 100 | 233 | 13 |
| 797 | 37.19 | -2.74 | GBIF | 12.82 | 72.46 | 22.30 | 19.63 | 154 | 6 | 81.54 | 183 | 27 | 4 |
| 798 | 36.88 | -0.37 | GBIF | 12.41 | 77.55 | 16.58 | 14.83 | 189 | 39 | 56.78 | 429 | 150 | 8 |
| 799 | 37.50 | 0.59 | GBIF | 13.31 | 83.70 | 23.40 | 23.08 | 126 | 4 | 96.79 | 185 | 17 | 11 |

| 800 | 36.91 | 0.10 | GBIF | 15.06 | 78.43 | 17.98 | 16.98 | 137 | 22 | 51.18 | 289 | 157 | 5 |
| --- | --- | --- | --- | --- | --- | --- | --- | --- | --- | --- | --- | --- | --- |
| 801 | 37.33 | 0.26 | GBIF | 13.13 | 83.07 | 17.03 | 16.52 | 182 | 22 | 75.44 | 254 | 98 | 11 |
| 802 | 37.35 | -2.66 | GBIF | 13.02 | 73.17 | 22.65 | 19.93 | 157 | 5 | 85.03 | 181 | 24 | 4 |
| 803 | 36.61 | 0.72 | GBIF | 14.78 | 82.59 | 18.17 | 17.57 | 116 | 18 | 51.00 | 260 | 193 | 10 |
| 804 | 35.01 | -1.60 | GBIF | 12.67 | 81.20 | 20.82 | 19.27 | 165 | 26 | 45.85 | 295 | 123 | 5 |
| 805 | 37.98 | -2.99 | GBIF | 11.35 | 68.79 | 23.80 | 20.75 | 123 | 3 | 92.39 | 131 | 14 | 6 |
| 806 | 35.33 | -1.46 | GBIF | 12.82 | 81.17 | 20.67 | 18.72 | 152 | 22 | 51.21 | 351 | 98 | 3 |
| 807 | 37.49 | 0.68 | GBIF | 12.47 | 83.67 | 23.35 | 21.92 | 148 | 5 | 97.34 | 237 | 16 | 10 |
| 808 | 37.64 | 0.46 | GBIF | 13.58 | 81.34 | 23.30 | 22.92 | 158 | 3 | 102.56 | 216 | 12 | 6 |
| 809 | 35.29 | -1.54 | GBIF | 12.29 | 80.87 | 19.45 | 17.80 | 178 | 26 | 51.13 | 394 | 114 | 11 |
| 810 | 38.08 | 0.20 | GBIF | 11.08 | 77.45 | 23.50 | 22.07 | 187 | 2 | 115.00 | 252 | 9 | 5 |
| 811 | 35.12 | -1.43 | GBIF | 12.83 | 81.74 | 20.52 | 18.93 | 163 | 28 | 45.01 | 375 | 130 | 9 |
| 812 | 35.07 | -1.53 | GBIF | 12.70 | 81.41 | 20.55 | 19.08 | 157 | 27 | 44.30 | 295 | 126 | 9 |
| 813 | 38.34 | -3.53 | GBIF | 10.63 | 67.68 | 22.50 | 19.55 | 141 | 12 | 74.47 | 184 | 46 | 6 |
| 814 | 36.81 | 0.33 | GBIF | 15.12 | 80.41 | 18.28 | 17.32 | 129 | 17 | 53.79 | 269 | 169 | 3 |
| 815 | 35.18 | -1.27 | GBIF | 12.98 | 82.17 | 20.00 | 18.45 | 164 | 31 | 42.70 | 380 | 145 | 3 |
| 816 | 38.79 | -3.61 | GBIF | 10.22 | 69.03 | 24.45 | 22.05 | 119 | 18 | 60.26 | 166 | 80 | 7 |
| 817 | 35.30 | -1.55 | GBIF | 12.22 | 80.91 | 19.30 | 17.65 | 177 | 26 | 50.94 | 395 | 114 | 15 |
| 818 | 35.15 | -1.23 | GBIF | 13.01 | 82.33 | 19.88 | 18.28 | 190 | 38 | 42.08 | 415 | 170 | 3 |
| 819 | 37.38 | -2.77 | GBIF | 12.81 | 72.36 | 22.05 | 19.35 | 180 | 11 | 80.13 | 201 | 44 | 6 |
| 820 | 37.34 | -2.74 | GBIF | 13.19 | 72.88 | 22.30 | 19.63 | 174 | 9 | 81.09 | 195 | 39 | 5 |
| 821 | 35.40 | -1.29 | GBIF | 12.51 | 82.29 | 18.50 | 16.77 | 174 | 27 | 50.25 | 385 | 118 | 3 |
| 822 | 37.60 | 0.66 | GBIF | 12.99 | 83.82 | 23.28 | 22.77 | 129 | 3 | 103.38 | 176 | 13 | 7 |
| 823 | 37.66 | 0.49 | GBIF | 13.63 | 81.64 | 23.35 | 22.90 | 155 | 3 | 104.13 | 208 | 11 | 6 |
| 824 | 37.67 | 0.41 | GBIF | 13.22 | 80.59 | 23.03 | 22.67 | 186 | 3 | 101.81 | 246 | 13 | 6 |
| 825 | 36.57 | 0.63 | GBIF | 15.02 | 82.55 | 18.42 | 17.92 | 120 | 19 | 51.57 | 258 | 195 | 7 |
| 826 | 37.76 | 0.52 | GBIF | 12.59 | 82.30 | 23.15 | 22.60 | 166 | 3 | 108.23 | 213 | 11 | 7 |
| 827 | 35.19 | -1.43 | GBIF | 12.88 | 82.01 | 20.58 | 18.98 | 152 | 27 | 45.09 | 358 | 123 | 3 |
| 828 | 36.89 | -0.28 | GBIF | 13.55 | 78.32 | 17.18 | 15.70 | 140 | 28 | 52.16 | 324 | 112 | 21 |
| 829 | 35.17 | -1.51 | GBIF | 12.85 | 80.82 | 20.33 | 18.77 | 154 | 26 | 46.64 | 363 | 117 | 2 |
| 830 | 36.82 | 0.38 | GBIF | 14.99 | 81.48 | 18.52 | 17.58 | 133 | 16 | 56.48 | 273 | 166 | 3 |
| 831 | 36.93 | 0.38 | GBIF | 14.67 | 81.94 | 19.47 | 18.52 | 183 | 21 | 66.80 | 367 | 144 | 5 |
| 832 | 35.31 | -1.42 | GBIF | 12.96 | 82.01 | 19.97 | 18.00 | 156 | 24 | 49.23 | 364 | 108 | 3 |
| 833 | 35.27 | -1.50 | GBIF | 12.78 | 81.37 | 20.48 | 18.53 | 153 | 23 | 49.42 | 358 | 105 | 6 |
| 834 | 36.91 | 0.04 | GBIF | 14.59 | 78.45 | 17.72 | 16.60 | 131 | 26 | 48.59 | 291 | 181 | 3 |
| 835 | 35.27 | -1.44 | GBIF | 12.93 | 81.80 | 20.50 | 18.57 | 152 | 25 | 47.71 | 357 | 111 | 3 |
| 836 | 35.03 | -1.37 | GBIF | 12.77 | 82.37 | 20.38 | 18.87 | 167 | 33 | 40.95 | 298 | 152 | 3 |
| 837 | 36.83 | 0.25 | GBIF | 15.01 | 78.99 | 18.10 | 17.07 | 134 | 18 | 52.96 | 281 | 171 | 3 |
| 838 | 37.40 | -2.30 | GBIF | 11.11 | 69.86 | 21.75 | 19.48 | 147 | 1 | 87.44 | 261 | 15 | 3 |
| 839 | 36.94 | -0.24 | GBIF | 13.89 | 78.48 | 17.30 | 15.92 | 135 | 27 | 53.39 | 314 | 102 | 18 |
| 840 | 35.15 | -1.31 | GBIF | 13.03 | 81.97 | 20.32 | 18.78 | 172 | 32 | 41.92 | 392 | 154 | 3 |
| 841 | 38.16 | -2.80 | GBIF | 10.57 | 67.79 | 23.60 | 20.62 | 148 | 3 | 99.30 | 145 | 15 | 1 |
| 842 | 35.37 | -1.49 | GBIF | 12.49 | 81.11 | 20.28 | 18.27 | 161 | 22 | 52.99 | 364 | 98 | 7 |
| 843 | 38.13 | -2.86 | GBIF | 10.59 | 67.90 | 23.40 | 20.38 | 148 | 3 | 99.46 | 260 | 15 | 8 |
| 844 | 38.19 | -2.88 | GBIF | 10.38 | 68.26 | 23.28 | 20.35 | 164 | 5 | 95.12 | 286 | 22 | 8 |
| 845 | 38.01 | -2.88 | GBIF | 10.45 | 67.42 | 23.48 | 20.30 | 137 | 3 | 95.35 | 142 | 16 | 1 |
| 846 | 38.91 | -2.62 | GBIF | 10.09 | 69.12 | 26.38 | 23.98 | 153 | 20 | 74.30 | 267 | 65 | 2 |
| 847 | 38.88 | -2.63 | GBIF | 9.89 | 68.69 | 26.28 | 23.88 | 155 | 20 | 75.23 | 266 | 64 | 2 |
| 848 | 35.09 | -1.32 | GBIF | 13.07 | 82.23 | 20.27 | 18.72 | 169 | 32 | 41.75 | 294 | 152 | 7 |
| 849 | 37.36 | -2.69 | GBIF | 13.08 | 73.09 | 22.63 | 19.90 | 156 | 6 | 83.58 | 184 | 27 | 5 |

| 850 | 38.84 | -2.79 | GBIF | 9.89 | 69.17 | 25.72 | 23.35 | 161 | 21 | 73.37 | 289 | 70 | 3 |
| --- | --- | --- | --- | --- | --- | --- | --- | --- | --- | --- | --- | --- | --- |
| 851 | 38.90 | -2.78 | GBIF | 10.02 | 69.08 | 26.00 | 23.63 | 159 | 23 | 71.46 | 284 | 74 | 3 |
| 852 | 38.81 | -2.68 | GBIF | 9.80 | 69.01 | 25.95 | 23.55 | 161 | 19 | 75.70 | 277 | 63 | 1 |
| 853 | 38.81 | -2.75 | GBIF | 9.88 | 69.06 | 25.70 | 23.28 | 165 | 21 | 74.89 | 293 | 69 | 2 |
| 854 | 37.86 | -2.92 | GBIF | 12.28 | 69.79 | 23.75 | 20.68 | 143 | 3 | 93.95 | 145 | 14 | 6 |
| 855 | 38.78 | -2.87 | GBIF | 10.13 | 69.41 | 25.55 | 23.18 | 174 | 23 | 71.41 | 312 | 79 | 2 |
| 856 | 38.51 | -3.35 | GBIF | 10.49 | 69.94 | 24.32 | 21.43 | 140 | 14 | 71.39 | 278 | 56 | 15 |
| 857 | 38.64 | -3.72 | GBIF | 10.20 | 67.55 | 23.40 | 20.88 | 128 | 15 | 64.81 | 177 | 73 | 6 |
| 858 | 38.28 | -3.56 | GBIF | 10.99 | 68.70 | 22.37 | 19.43 | 134 | 11 | 75.19 | 193 | 42 | 4 |
| 859 | 38.08 | -3.56 | GBIF | 11.23 | 68.87 | 22.88 | 19.88 | 123 | 7 | 78.90 | 182 | 27 | 2 |
| 860 | 38.62 | -3.39 | GBIF | 10.70 | 69.93 | 25.00 | 22.05 | 136 | 16 | 67.36 | 277 | 66 | 6 |
| 861 | 38.75 | -3.29 | GBIF | 10.57 | 70.50 | 25.50 | 22.55 | 144 | 21 | 65.57 | 279 | 76 | 2 |
| 862 | 37.58 | -2.40 | GBIF | 12.27 | 70.50 | 22.53 | 19.98 | 158 | 1 | 97.48 | 250 | 8 | 4 |
| 863 | 38.06 | -3.43 | GBIF | 11.07 | 68.36 | 22.23 | 19.23 | 123 | 8 | 80.51 | 172 | 29 | 3 |
| 864 | 37.91 | -2.98 | GBIF | 12.04 | 70.01 | 23.92 | 20.88 | 135 | 3 | 94.46 | 138 | 12 | 4 |
| 865 | 37.45 | 0.77 | GBIF | 12.36 | 83.50 | 22.07 | 20.55 | 187 | 6 | 97.00 | 310 | 19 | 2 |
| 866 | 36.90 | 0.33 | GBIF | 14.67 | 81.03 | 19.15 | 18.25 | 173 | 20 | 62.42 | 350 | 156 | 6 |
| 867 | 37.94 | -3.44 | GBIF | 11.13 | 67.84 | 22.57 | 19.73 | 163 | 11 | 81.33 | 191 | 39 | 3 |
| 868 | 36.99 | 0.39 | GBIF | 14.74 | 82.36 | 19.17 | 18.30 | 167 | 19 | 67.05 | 251 | 133 | 6 |
| 869 | 38.84 | -3.55 | GBIF | 10.43 | 70.44 | 24.78 | 22.40 | 120 | 19 | 61.20 | 238 | 82 | 2 |
| 870 | 38.92 | -3.02 | GBIF | 10.43 | 70.02 | 26.15 | 23.78 | 150 | 26 | 63.44 | 277 | 88 | 4 |
| 871 | 36.03 | -1.93 | GBIF | 11.00 | 73.33 | 23.60 | 21.38 | 97 | 5 | 75.19 | 167 | 19 | 5 |
| 872 | 36.82 | 0.31 | GBIF | 15.05 | 80.05 | 18.20 | 17.22 | 129 | 17 | 53.01 | 267 | 168 | 2 |
| 873 | 37.40 | -2.56 | GBIF | 12.17 | 71.15 | 22.48 | 19.75 | 144 | 5 | 84.29 | 182 | 24 | 7 |
| 874 | 37.80 | -3.23 | GBIF | 11.80 | 69.01 | 23.23 | 20.47 | 203 | 8 | 95.02 | 181 | 31 | 11 |
| 875 | 37.89 | -3.23 | GBIF | 11.70 | 69.23 | 23.18 | 20.17 | 189 | 9 | 91.22 | 177 | 31 | 4 |
| 876 | 35.27 | -1.03 | GBIF | 12.75 | 81.21 | 19.32 | 17.73 | 167 | 37 | 41.07 | 286 | 165 | 11 |
| 877 | 38.76 | -3.58 | GBIF | 10.32 | 69.24 | 24.35 | 21.93 | 122 | 17 | 62.06 | 170 | 82 | 10 |
| 878 | 36.94 | 0.60 | GBIF | 14.04 | 83.58 | 19.58 | 18.60 | 188 | 21 | 73.71 | 378 | 119 | 6 |
| 879 | 38.45 | -3.30 | GBIF | 9.40 | 68.12 | 22.17 | 19.67 | 159 | 11 | 81.48 | 314 | 41 | 7 |
| 880 | 35.15 | -1.33 | GBIF | 13.10 | 82.39 | 20.45 | 18.93 | 163 | 31 | 42.27 | 291 | 145 | 3 |
| 881 | 38.58 | -3.39 | GBIF | 10.54 | 69.35 | 24.75 | 21.85 | 145 | 18 | 66.84 | 283 | 70 | 27 |
| 882 | 38.74 | -3.30 | GBIF | 10.60 | 70.20 | 25.47 | 22.53 | 143 | 21 | 65.91 | 280 | 74 | 3 |
| 883 | 37.25 | -2.61 | GBIF | 12.64 | 73.07 | 22.70 | 19.95 | 148 | 3 | 84.77 | 177 | 20 | 12 |
| 884 | 38.54 | -3.20 | GBIF | 10.38 | 69.63 | 25.02 | 22.07 | 164 | 15 | 73.07 | 311 | 60 | 5 |
| 885 | 37.47 | 0.78 | GBIF | 12.57 | 83.78 | 23.28 | 21.75 | 140 | 5 | 97.07 | 233 | 17 | 8 |
| 886 | 37.22 | -2.71 | GBIF | 13.28 | 73.80 | 22.63 | 19.95 | 155 | 5 | 82.62 | 180 | 26 | 4 |
| 887 | 37.59 | 0.61 | GBIF | 13.24 | 83.28 | 23.55 | 22.97 | 122 | 3 | 103.14 | 165 | 13 | 18 |
| 888 | 35.10 | -1.43 | GBIF | 12.80 | 82.05 | 20.53 | 19.00 | 160 | 29 | 43.36 | 371 | 136 | 9 |
| 889 | 37.86 | 2.26 | GBIF | 10.05 | 72.30 | 23.20 | 21.05 | 106 | 1 | 105.03 | 171 | 5 | 3 |
| 890 | 38.34 | -2.97 | GBIF | 10.25 | 68.79 | 24.70 | 21.68 | 173 | 13 | 81.18 | 308 | 54 | 3 |
| 891 | 38.09 | -2.83 | GBIF | 10.42 | 67.64 | 23.27 | 20.20 | 151 | 2 | 101.99 | 148 | 11 | 8 |
| 892 | 35.21 | -1.42 | GBIF | 12.85 | 81.85 | 20.78 | 18.87 | 157 | 26 | 46.25 | 363 | 122 | 11 |
| 893 | 38.82 | -3.31 | GBIF | 10.78 | 70.48 | 25.62 | 22.70 | 139 | 20 | 63.98 | 276 | 92 | 4 |
| 894 | 38.29 | -2.91 | GBIF | 10.21 | 68.51 | 23.87 | 21.23 | 158 | 8 | 87.84 | 285 | 35 | 2 |
| 895 | 38.48 | -3.17 | GBIF | 10.36 | 69.99 | 24.72 | 21.77 | 159 | 14 | 75.92 | 305 | 54 | 7 |
| 896 | 37.91 | -3.04 | GBIF | 12.18 | 70.02 | 24.00 | 20.98 | 139 | 3 | 95.31 | 136 | 12 | 11 |
| 897 | 38.39 | -2.80 | GBIF | 10.45 | 69.21 | 24.88 | 22.33 | 178 | 14 | 83.74 | 312 | 53 | 1 |
| 898 | 37.47 | 0.62 | GBIF | 13.27 | 83.49 | 23.23 | 22.93 | 130 | 5 | 94.88 | 194 | 19 | 6 |
| 899 | 38.09 | -2.82 | GBIF | 10.34 | 67.59 | 23.25 | 20.15 | 150 | 2 | 103.94 | 145 | 9 | 1 |

| 900 | 36.92 | 0.02 | GBIF | 14.48 | 78.71 | 17.65 | 16.53 | 130 | 27 | 49.29 | 293 | 185 | 12 |
| --- | --- | --- | --- | --- | --- | --- | --- | --- | --- | --- | --- | --- | --- |
| 901 | 38.76 | -3.25 | GBIF | 10.53 | 70.17 | 25.62 | 22.68 | 154 | 21 | 66.91 | 290 | 93 | 10 |
| 902 | 35.02 | -1.42 | GBIF | 12.55 | 82.03 | 20.27 | 18.78 | 166 | 31 | 40.68 | 304 | 151 | 5 |
| 903 | 37.55 | 0.40 | GBIF | 12.81 | 81.58 | 22.93 | 22.65 | 164 | 5 | 96.41 | 232 | 261 | 7 |
| 904 | 36.85 | 0.26 | GBIF | 14.93 | 79.81 | 18.02 | 17.00 | 131 | 17 | 53.33 | 273 | 169 | 2 |
| 905 | 38.88 | -3.22 | GBIF | 10.39 | 70.21 | 25.15 | 22.80 | 143 | 22 | 64.94 | 273 | 96 | 3 |
| 906 | 39.17 | -3.14 | GBIF | 10.37 | 70.52 | 26.03 | 23.82 | 130 | 23 | 60.06 | 236 | 101 | 3 |
| 907 | 37.90 | -0.11 | GBIF | 11.23 | 75.84 | 23.10 | 21.37 | 200 | 1 | 112.91 | 285 | 8 | 8 |
| 908 | 38.94 | -3.38 | GBIF | 10.57 | 69.98 | 25.15 | 22.77 | 130 | 24 | 60.94 | 258 | 96 | 6 |
| 909 | 38.63 | -3.05 | GBIF | 10.73 | 70.56 | 26.02 | 23.05 | 196 | 24 | 70.39 | 344 | 89 | 11 |
| 910 | 38.89 | -3.09 | GBIF | 10.50 | 70.00 | 25.88 | 23.52 | 149 | 24 | 64.42 | 279 | 83 | 3 |
| 911 | 38.94 | -3.25 | GBIF | 10.42 | 70.38 | 25.33 | 22.95 | 136 | 22 | 62.73 | 262 | 98 | 7 |
| 912 | 36.95 | 0.08 | GBIF | 14.75 | 78.88 | 17.57 | 16.55 | 128 | 22 | 49.92 | 278 | 155 | 19 |
| 913 | 38.92 | -3.20 | GBIF | 10.38 | 70.63 | 25.47 | 23.13 | 140 | 21 | 64.77 | 267 | 95 | 3 |
| 914 | 37.44 | 0.56 | GBIF | 13.79 | 84.61 | 23.12 | 22.93 | 140 | 6 | 92.13 | 218 | 23 | 8 |
| 915 | 36.84 | 0.06 | GBIF | 14.82 | 78.44 | 17.60 | 16.47 | 121 | 26 | 44.34 | 261 | 171 | 3 |
| 916 | 35.33 | -1.55 | GBIF | 12.05 | 80.87 | 18.38 | 16.72 | 195 | 28 | 52.22 | 422 | 121 | 4 |
| 917 | 35.22 | -1.56 | GBIF | 12.73 | 81.10 | 20.15 | 18.63 | 161 | 24 | 49.49 | 368 | 111 | 4 |
| 918 | 35.59 | -1.26 | GBIF | 13.45 | 81.52 | 18.58 | 16.78 | 149 | 22 | 54.10 | 339 | 89 | 3 |
| 919 | 35.46 | -1.22 | GBIF | 13.24 | 82.25 | 18.27 | 16.57 | 161 | 26 | 49.12 | 365 | 114 | 3 |
| 920 | 38.49 | -3.33 | GBIF | 10.03 | 69.20 | 23.93 | 21.02 | 148 | 12 | 74.23 | 288 | 52 | 10 |
| 921 | 35.52 | -1.28 | GBIF | 13.27 | 81.89 | 18.72 | 16.97 | 154 | 23 | 52.69 | 351 | 96 | 3 |
| 922 | 37.35 | -2.68 | GBIF | 13.02 | 73.13 | 22.62 | 19.88 | 158 | 5 | 85.23 | 181 | 25 | 4 |
| 923 | 37.22 | -2.37 | GBIF | 11.88 | 71.97 | 22.65 | 20.00 | 121 | 1 | 83.11 | 249 | 15 | 11 |
| 924 | 37.26 | -2.29 | GBIF | 11.09 | 70.20 | 22.18 | 19.47 | 127 | 2 | 81.72 | 249 | 17 | 3 |
| 925 | 37.40 | -2.24 | GBIF | 10.96 | 69.36 | 21.52 | 19.37 | 152 | 1 | 87.59 | 258 | 16 | 3 |
| 926 | 35.59 | -1.23 | GBIF | 13.53 | 81.53 | 18.48 | 16.72 | 153 | 23 | 54.00 | 343 | 93 | 3 |
| 927 | 35.54 | -1.29 | GBIF | 13.27 | 81.94 | 18.52 | 16.77 | 151 | 23 | 52.92 | 346 | 93 | 7 |
| 928 | 38.11 | -3.48 | GBIF | 11.32 | 68.22 | 22.82 | 19.77 | 126 | 8 | 79.18 | 171 | 29 | 3 |
| 929 | 36.72 | -0.42 | GBIF | 10.99 | 79.08 | 10.55 | 9.82 | 188 | 37 | 36.33 | 374 | 320 | 4 |
| 930 | 36.77 | 0.56 | GBIF | 14.74 | 82.82 | 18.50 | 17.58 | 126 | 16 | 55.94 | 267 | 155 | 13 |
| 931 | 37.92 | -2.87 | GBIF | 11.77 | 68.46 | 23.73 | 20.32 | 150 | 3 | 96.62 | 147 | 14 | 9 |
| 932 | 36.73 | 0.57 | GBIF | 14.98 | 83.19 | 18.00 | 17.42 | 117 | 16 | 54.14 | 248 | 162 | 6 |
| 933 | 37.23 | -2.21 | GBIF | 11.70 | 71.78 | 21.75 | 19.62 | 126 | 1 | 82.42 | 244 | 16 | 7 |
| 934 | 35.48 | -1.33 | GBIF | 13.09 | 81.82 | 19.48 | 17.47 | 145 | 23 | 51.89 | 343 | 94 | 3 |
| 935 | 38.54 | -3.33 | GBIF | 10.54 | 69.81 | 24.53 | 21.62 | 144 | 14 | 71.79 | 283 | 57 | 20 |
| 936 | 38.19 | 0.12 | GBIF | 10.56 | 75.96 | 24.27 | 22.48 | 164 | 1 | 121.30 | 199 | 3 | 4 |
| 937 | 35.04 | -1.47 | GBIF | 12.69 | 81.36 | 20.72 | 19.18 | 159 | 29 | 42.21 | 291 | 140 | 5 |
| 938 | 37.41 | 0.65 | GBIF | 12.96 | 84.15 | 22.88 | 21.43 | 170 | 7 | 93.61 | 286 | 23 | 5 |
| 939 | 35.05 | -1.44 | GBIF | 12.70 | 81.41 | 20.65 | 19.13 | 162 | 29 | 42.74 | 377 | 139 | 3 |
| 940 | 35.22 | -1.38 | GBIF | 12.89 | 82.11 | 20.05 | 18.47 | 155 | 27 | 45.24 | 364 | 126 | 7 |
| 941 | 39.81 | -3.25 | GBIF | 8.22 | 67.91 | 25.15 | 26.32 | 163 | 14 | 55.93 | 166 | 164 | 13 |
| 942 | 39.96 | -3.26 | GBIF | 7.10 | 66.36 | 24.70 | 25.73 | 223 | 10 | 73.21 | 151 | 227 | 5 |
| 943 | 35.11 | -1.31 | GBIF | 13.12 | 81.98 | 20.37 | 18.85 | 166 | 32 | 41.76 | 290 | 150 | 7 |
| 944 | 35.21 | -1.04 | GBIF | 12.71 | 81.46 | 19.25 | 17.70 | 167 | 41 | 38.29 | 292 | 179 | 21 |
| 945 | 35.06 | -1.09 | GBIF | 12.32 | 82.66 | 18.88 | 17.37 | 206 | 52 | 36.74 | 324 | 226 | 15 |
| 946 | 35.00 | -1.01 | GBIF | 12.33 | 82.22 | 19.25 | 17.82 | 214 | 60 | 35.18 | 323 | 262 | 14 |
| 947 | 36.85 | 0.07 | GBIF | 14.92 | 78.51 | 17.82 | 16.70 | 132 | 25 | 47.06 | 280 | 170 | 3 |
| 948 | 37.77 | 0.77 | GBIF | 12.18 | 82.82 | 22.80 | 22.20 | 157 | 2 | 108.27 | 217 | 10 | 8 |
| 949 | 37.76 | 0.66 | GBIF | 12.92 | 83.87 | 23.87 | 23.13 | 128 | 2 | 110.95 | 169 | 8 | 6 |

| 950 | 37.26 | -2.63 | GBIF | 12.57 | 73.06 | 22.60 | 19.87 | 143 | 3 | 84.52 | 176 | 19 | 8 |
| --- | --- | --- | --- | --- | --- | --- | --- | --- | --- | --- | --- | --- | --- |
| 951 | 37.58 | 0.73 | GBIF | 12.26 | 83.39 | 22.90 | 22.30 | 132 | 3 | 102.64 | 190 | 12 | 12 |
| 952 | 37.86 | -2.69 | GBIF | 10.55 | 67.63 | 21.13 | 18.13 | 202 | 7 | 92.40 | 223 | 27 | 5 |
| 953 | 34.97 | -1.52 | GBIF | 12.47 | 81.54 | 20.57 | 19.12 | 171 | 30 | 43.00 | 305 | 140 | 17 |
| 954 | 38.96 | -0.43 | GBIF | 10.53 | 73.66 | 26.55 | 24.58 | 145 | 0 | 129.18 | 134 | 2 | 11 |
| 955 | 35.00 | -1.43 | GBIF | 12.58 | 81.71 | 20.45 | 18.93 | 163 | 32 | 40.46 | 302 | 150 | 4 |
| 956 | 35.13 | -1.42 | GBIF | 12.84 | 81.79 | 20.67 | 19.07 | 159 | 28 | 44.26 | 369 | 131 | 2 |
| 957 | 36.83 | 0.22 | GBIF | 15.04 | 79.17 | 17.97 | 16.95 | 130 | 18 | 51.72 | 274 | 171 | 3 |
| 958 | 38.31 | -3.56 | GBIF | 10.80 | 67.92 | 22.12 | 19.17 | 143 | 12 | 74.19 | 199 | 49 | 6 |
| 959 | 37.70 | -2.32 | GBIF | 11.44 | 69.34 | 22.68 | 20.13 | 165 | 2 | 98.89 | 234 | 11 | 4 |
| 960 | 37.31 | -2.74 | GBIF | 13.37 | 73.44 | 22.42 | 19.77 | 175 | 8 | 82.98 | 192 | 33 | 4 |
| 961 | 35.25 | -1.60 | GBIF | 12.57 | 81.13 | 20.25 | 18.38 | 162 | 23 | 52.22 | 371 | 102 | 7 |
| 962 | 35.22 | 1.78 | GBIF | 12.71 | 84.16 | 21.83 | 22.57 | 103 | 14 | 52.84 | 162 | 244 | 5 |
| 963 | 36.87 | 0.03 | GBIF | 14.68 | 78.48 | 17.60 | 16.47 | 129 | 28 | 45.93 | 280 | 180 | 10 |
| 964 | 36.86 | 0.31 | GBIF | 14.91 | 80.59 | 18.17 | 17.20 | 125 | 16 | 53.40 | 259 | 165 | 4 |
| 965 | 36.95 | -0.27 | GBIF | 13.52 | 77.73 | 17.55 | 16.10 | 141 | 26 | 55.99 | 328 | 98 | 6 |
| 966 | 36.98 | 0.29 | GBIF | 14.43 | 80.63 | 18.58 | 17.70 | 157 | 18 | 61.66 | 237 | 152 | 5 |
| 967 | 36.82 | 0.05 | GBIF | 14.88 | 78.29 | 17.63 | 16.50 | 120 | 27 | 43.38 | 261 | 171 | 3 |
| 968 | 36.93 | -0.30 | GBIF | 13.23 | 77.34 | 17.45 | 15.92 | 143 | 28 | 54.82 | 334 | 104 | 10 |
| 969 | 35.09 | -1.25 | GBIF | 12.99 | 82.23 | 20.07 | 18.55 | 177 | 38 | 39.17 | 304 | 175 | 3 |
| 970 | 35.19 | -1.51 | GBIF | 12.85 | 80.82 | 20.35 | 18.73 | 156 | 25 | 48.19 | 364 | 113 | 6 |
| 971 | 36.86 | -0.28 | GBIF | 13.50 | 78.95 | 16.40 | 14.77 | 152 | 34 | 49.77 | 338 | 135 | 14 |
| 972 | 36.85 | 0.11 | GBIF | 15.11 | 78.28 | 18.03 | 16.98 | 136 | 22 | 49.30 | 288 | 163 | 11 |
| 973 | 35.05 | -1.21 | GBIF | 12.49 | 82.18 | 20.03 | 18.50 | 177 | 40 | 38.15 | 300 | 182 | 8 |
| 974 | 36.84 | 0.58 | GBIF | 14.49 | 83.29 | 19.52 | 18.50 | 174 | 21 | 66.31 | 365 | 147 | 5 |
| 975 | 35.30 | -1.54 | GBIF | 12.32 | 80.50 | 19.38 | 17.73 | 171 | 25 | 51.60 | 385 | 108 | 11 |
| 976 | 35.10 | -1.37 | GBIF | 12.95 | 81.45 | 20.17 | 18.60 | 170 | 31 | 42.87 | 387 | 147 | 4 |
| 977 | 35.00 | -1.50 | GBIF | 12.63 | 81.51 | 20.78 | 19.28 | 164 | 29 | 42.41 | 298 | 140 | 12 |
| 978 | 37.20 | -2.78 | GBIF | 12.19 | 70.88 | 21.52 | 18.88 | 171 | 10 | 79.54 | 198 | 41 | 4 |
| 979 | 36.98 | 0.23 | GBIF | 14.37 | 79.81 | 18.23 | 17.33 | 150 | 19 | 59.09 | 230 | 155 | 19 |
| 980 | 38.36 | -2.80 | GBIF | 10.39 | 69.28 | 24.68 | 22.13 | 172 | 11 | 86.45 | 304 | 44 | 1 |
| 981 | 38.32 | -2.61 | GBIF | 10.23 | 69.09 | 24.70 | 22.15 | 161 | 5 | 98.91 | 263 | 23 | 5 |
| 982 | 39.00 | -3.22 | GBIF | 10.32 | 70.18 | 25.40 | 23.07 | 137 | 24 | 61.70 | 253 | 101 | 2 |
| 983 | 34.89 | -1.21 | GBIF | 11.41 | 81.49 | 19.28 | 17.77 | 217 | 49 | 37.59 | 330 | 223 | 3 |
| 984 | 38.75 | -3.25 | GBIF | 10.46 | 69.72 | 25.58 | 22.62 | 157 | 21 | 67.34 | 295 | 76 | 10 |
| 985 | 34.91 | -1.43 | GBIF | 12.58 | 81.71 | 20.58 | 19.03 | 173 | 34 | 40.19 | 305 | 157 | 5 |
| 986 | 37.52 | 0.45 | GBIF | 12.28 | 81.89 | 20.57 | 20.15 | 247 | 8 | 98.54 | 348 | 27 | 10 |
| 987 | 38.63 | -3.27 | GBIF | 10.44 | 70.08 | 25.13 | 22.20 | 158 | 17 | 70.02 | 301 | 68 | 8 |
| 988 | 37.41 | 0.53 | GBIF | 13.89 | 84.71 | 24.12 | 22.80 | 141 | 7 | 90.47 | 229 | 24 | 6 |
| 989 | 38.65 | -3.31 | GBIF | 10.63 | 70.83 | 25.13 | 22.23 | 149 | 18 | 68.35 | 288 | 69 | 0 |
| 990 | 36.84 | -0.22 | GBIF | 13.85 | 79.14 | 16.68 | 15.23 | 131 | 29 | 47.89 | 297 | 123 | 9 |
| 991 | 37.57 | 0.80 | GBIF | 12.83 | 83.88 | 22.98 | 22.42 | 131 | 3 | 102.30 | 195 | 12 | 4 |
| 992 | 35.21 | -1.48 | GBIF | 12.93 | 81.34 | 20.33 | 18.73 | 153 | 25 | 47.34 | 358 | 114 | 6 |
| 993 | 39.93 | -3.27 | GBIF | 7.26 | 66.59 | 24.80 | 25.83 | 216 | 11 | 70.05 | 154 | 176 | 3 |
| 994 | 38.74 | -3.24 | GBIF | 10.44 | 70.08 | 25.57 | 22.62 | 157 | 21 | 66.86 | 296 | 77 | 3 |
| 995 | 37.59 | 0.48 | GBIF | 13.45 | 82.01 | 23.33 | 22.93 | 147 | 4 | 100.76 | 203 | 14 | 10 |
| 996 | 38.38 | -3.42 | GBIF | 9.72 | 67.01 | 21.30 | 18.38 | 185 | 21 | 73.22 | 237 | 75 | 12 |
| 997 | 38.60 | -3.35 | GBIF | 10.77 | 69.97 | 24.85 | 21.90 | 146 | 17 | 68.71 | 287 | 66 | 10 |
| 998 | 34.91 | 1.60 | GBIF | 12.48 | 83.72 | 20.62 | 21.47 | 143 | 21 | 52.01 | 217 | 356 | 7 |
| 999 | 39.97 | -3.24 | GBIF | 7.16 | 65.67 | 24.50 | 25.55 | 209 | 10 | 68.24 | 168 | 218 | 7 |

| 1000 | 35.39 | -1.41 | GBIF | 12.99 | 81.71 | 20.08 | 18.07 | 151 | 22 | 51.88 | 353 | 96 | 3 |
| --- | --- | --- | --- | --- | --- | --- | --- | --- | --- | --- | --- | --- | --- |
| 1001 | 39.84 | -3.30 | GBIF | 7.70 | 67.54 | 24.72 | 25.85 | 196 | 14 | 62.33 | 187 | 165 | 5 |
| 1002 | 38.61 | -3.54 | GBIF | 9.41 | 67.20 | 22.65 | 20.12 | 138 | 16 | 68.67 | 188 | 64 | 13 |
| 1003 | 35.01 | -1.29 | GBIF | 12.54 | 81.97 | 20.23 | 18.77 | 175 | 37 | 38.91 | 300 | 172 | 4 |
| 1004 | 35.11 | -1.57 | GBIF | 12.71 | 81.46 | 20.47 | 19.03 | 156 | 26 | 45.77 | 293 | 119 | 12 |
| 1005 | 35.08 | -1.35 | GBIF | 12.93 | 81.80 | 20.08 | 18.55 | 172 | 32 | 42.46 | 298 | 150 | 3 |
| 1006 | 35.07 | -1.59 | GBIF | 12.76 | 81.78 | 20.73 | 19.10 | 154 | 26 | 45.19 | 291 | 119 | 6 |
| 1007 | 35.12 | -1.56 | GBIF | 12.71 | 81.46 | 20.32 | 18.85 | 160 | 25 | 46.62 | 294 | 119 | 4 |
| 1008 | 37.23 | -2.74 | GBIF | 12.87 | 72.28 | 22.12 | 19.48 | 165 | 7 | 81.40 | 188 | 33 | 5 |
| 1009 | 35.17 | -1.56 | GBIF | 12.73 | 81.05 | 20.22 | 18.68 | 162 | 25 | 48.62 | 371 | 113 | 3 |
| 1010 | 35.07 | -1.29 | GBIF | 12.92 | 82.27 | 20.28 | 18.75 | 171 | 35 | 40.05 | 296 | 163 | 7 |
| 1011 | 37.38 | -2.73 | GBIF | 13.18 | 73.24 | 22.52 | 19.82 | 166 | 8 | 82.11 | 190 | 35 | 10 |
| 1012 | 36.84 | 0.36 | GBIF | 14.93 | 81.11 | 18.38 | 17.45 | 128 | 16 | 54.95 | 268 | 164 | 3 |
| 1013 | 36.85 | 0.18 | GBIF | 15.17 | 78.99 | 18.12 | 17.08 | 138 | 20 | 51.88 | 291 | 165 | 3 |
| 1014 | 36.90 | 0.28 | GBIF | 14.67 | 80.59 | 18.60 | 17.68 | 154 | 19 | 57.98 | 314 | 162 | 4 |
| 1015 | 38.16 | 2.26 | GBIF | 10.40 | 72.22 | 25.55 | 23.65 | 116 | 1 | 112.85 | 69 | 5 | 6 |
| 1016 | 36.91 | 0.20 | GBIF | 14.85 | 78.99 | 18.28 | 17.27 | 149 | 20 | 55.59 | 316 | 165 | 3 |
| 1017 | 37.36 | -2.74 | GBIF | 13.18 | 73.19 | 22.42 | 19.75 | 171 | 9 | 81.31 | 193 | 37 | 5 |
| 1018 | 37.38 | -2.69 | GBIF | 13.17 | 73.15 | 22.63 | 19.93 | 161 | 6 | 84.08 | 185 | 28 | 7 |
| 1019 | 37.48 | 0.79 | GBIF | 12.69 | 84.05 | 23.55 | 22.18 | 134 | 4 | 97.77 | 218 | 16 | 12 |
| 1020 | 36.93 | -0.22 | GBIF | 13.87 | 78.34 | 17.28 | 15.93 | 132 | 26 | 52.75 | 302 | 248 | 14 |
| 1021 | 37.46 | 0.55 | GBIF | 13.77 | 83.94 | 23.15 | 22.87 | 142 | 6 | 93.70 | 217 | 21 | 7 |
| 1022 | 37.30 | -2.76 | GBIF | 13.17 | 72.74 | 22.22 | 19.55 | 175 | 9 | 81.24 | 194 | 38 | 4 |
| 1023 | 38.37 | -3.51 | GBIF | 10.60 | 67.95 | 22.90 | 19.90 | 134 | 10 | 75.82 | 174 | 41 | 25 |
| 1024 | 37.00 | -0.27 | GBIF | 13.61 | 78.21 | 17.35 | 15.82 | 155 | 25 | 59.80 | 357 | 94 | 31 |
| 1025 | 35.14 | -1.54 | GBIF | 12.73 | 81.10 | 20.13 | 18.63 | 163 | 26 | 47.03 | 376 | 120 | 2 |
| 1026 | 38.16 | -2.60 | GBIF | 11.13 | 70.02 | 24.32 | 21.63 | 155 | 2 | 105.97 | 249 | 13 | 6 |
| 1027 | 38.03 | -2.90 | GBIF | 10.56 | 68.12 | 23.80 | 20.50 | 127 | 3 | 96.41 | 133 | 13 | 9 |
| 1028 | 37.41 | 0.64 | GBIF | 13.09 | 84.46 | 23.63 | 22.17 | 145 | 7 | 93.86 | 240 | 21 | 6 |
| 1029 | 37.29 | -2.64 | GBIF | 12.52 | 72.82 | 22.57 | 19.83 | 144 | 4 | 83.93 | 176 | 22 | 12 |
| 1030 | 35.11 | -1.22 | GBIF | 13.03 | 82.49 | 19.78 | 18.27 | 180 | 38 | 39.59 | 305 | 176 | 4 |
| 1031 | 38.99 | -2.70 | GBIF | 9.88 | 68.63 | 26.33 | 23.97 | 149 | 23 | 69.64 | 252 | 72 | 2 |
| 1032 | 34.85 | -1.26 | GBIF | 11.55 | 81.34 | 19.05 | 17.57 | 217 | 48 | 38.40 | 338 | 212 | 5 |
| 1033 | 35.35 | -1.44 | GBIF | 12.88 | 81.49 | 20.55 | 18.55 | 150 | 22 | 51.20 | 350 | 98 | 3 |
| 1034 | 37.46 | 0.46 | GBIF | 13.40 | 83.75 | 22.75 | 22.55 | 162 | 7 | 91.56 | 251 | 257 | 9 |
| 1035 | 37.77 | -3.56 | GBIF | 10.52 | 68.74 | 24.25 | 21.45 | 101 | 4 | 82.08 | 160 | 17 | 11 |
| 1036 | 35.05 | -1.59 | GBIF | 12.78 | 81.42 | 20.67 | 19.35 | 156 | 26 | 44.78 | 294 | 122 | 11 |
| 1037 | 35.19 | -1.32 | GBIF | 12.95 | 81.96 | 20.32 | 18.72 | 156 | 29 | 43.32 | 367 | 135 | 3 |
| 1038 | 36.67 | -0.55 | GBIF | 10.35 | 78.41 | 9.92 | 9.25 | 223 | 47 | 37.28 | 423 | 345 | 6 |
| 1039 | 37.89 | -0.18 | GBIF | 10.83 | 74.20 | 23.23 | 20.97 | 191 | 0 | 117.13 | 274 | 4 | 6 |
| 1040 | 35.20 | -1.25 | GBIF | 12.94 | 82.43 | 19.60 | 18.05 | 169 | 33 | 43.07 | 296 | 148 | 4 |
| 1041 | 38.82 | -2.72 | GBIF | 9.82 | 68.71 | 25.83 | 23.45 | 162 | 20 | 74.99 | 281 | 67 | 2 |
| 1042 | 35.11 | -1.28 | GBIF | 13.11 | 81.93 | 20.20 | 18.68 | 166 | 33 | 40.72 | 292 | 155 | 4 |
| 1043 | 37.69 | -3.41 | GBIF | 11.29 | 69.27 | 24.12 | 21.27 | 146 | 7 | 85.72 | 166 | 30 | 14 |
| 1044 | 38.24 | 0.18 | GBIF | 11.52 | 77.82 | 24.60 | 23.30 | 160 | 0 | 124.61 | 189 | 1 | 0 |
| 1045 | 37.41 | 0.51 | GBIF | 14.05 | 84.64 | 24.13 | 22.83 | 146 | 8 | 90.11 | 238 | 231 | 7 |
| 1046 | 38.70 | -3.73 | GBIF | 10.34 | 68.49 | 24.17 | 21.68 | 120 | 17 | 60.51 | 166 | 77 | 15 |
| 1047 | 37.21 | -2.80 | GBIF | 12.04 | 70.83 | 21.12 | 18.53 | 186 | 12 | 79.30 | 206 | 50 | 3 |
| 1048 | 37.43 | 0.52 | GBIF | 14.01 | 84.39 | 23.13 | 22.87 | 143 | 7 | 91.28 | 232 | 226 | 6 |
| 1049 | 35.13 | -1.24 | GBIF | 13.00 | 82.28 | 19.88 | 18.30 | 187 | 38 | 40.56 | 309 | 176 | 3 |

| 1050 | 35.38 | -1.47 | GBIF | 12.69 | 81.36 | 20.38 | 18.37 | 155 | 22 | 53.64 | 353 | 93 | 8 |
| --- | --- | --- | --- | --- | --- | --- | --- | --- | --- | --- | --- | --- | --- |
| 1051 | 37.10 | -2.57 | GBIF | 12.11 | 71.65 | 22.68 | 20.05 | 123 | 2 | 81.88 | 175 | 15 | 4 |
| 1052 | 36.90 | 0.26 | GBIF | 14.67 | 80.15 | 18.33 | 17.37 | 148 | 18 | 57.15 | 298 | 163 | 3 |
| 1053 | 35.18 | -1.28 | GBIF | 12.96 | 82.54 | 20.02 | 18.48 | 164 | 31 | 42.50 | 294 | 145 | 3 |
| 1054 | 37.03 | -2.59 | GBIF | 12.18 | 72.09 | 22.68 | 20.15 | 120 | 1 | 81.98 | 177 | 13 | 4 |
| 1055 | 37.12 | -2.54 | GBIF | 12.06 | 71.35 | 22.68 | 20.03 | 126 | 2 | 83.02 | 178 | 13 | 13 |
| 1056 | 35.08 | -1.52 | GBIF | 12.73 | 81.57 | 20.33 | 18.87 | 160 | 28 | 44.26 | 374 | 130 | 9 |
| 1057 | 37.17 | -2.54 | GBIF | 12.48 | 72.16 | 22.77 | 20.18 | 132 | 2 | 83.77 | 178 | 14 | 13 |
| 1058 | 35.16 | -1.34 | GBIF | 13.01 | 81.81 | 20.47 | 18.90 | 158 | 30 | 43.35 | 287 | 137 | 3 |
| 1059 | 37.15 | -2.45 | GBIF | 12.05 | 71.73 | 22.53 | 19.93 | 122 | 2 | 81.19 | 250 | 17 | 3 |
| 1060 | 37.06 | -2.56 | GBIF | 11.75 | 71.21 | 22.57 | 19.95 | 126 | 2 | 82.12 | 177 | 15 | 5 |
| 1061 | 37.03 | -2.48 | GBIF | 10.95 | 69.75 | 21.65 | 19.03 | 134 | 2 | 80.05 | 194 | 17 | 4 |
| 1062 | 37.05 | -2.54 | GBIF | 11.60 | 70.73 | 22.52 | 19.92 | 126 | 2 | 82.02 | 177 | 15 | 9 |
| 1063 | 36.80 | 0.26 | GBIF | 15.17 | 78.99 | 18.08 | 17.05 | 133 | 18 | 52.54 | 275 | 173 | 3 |
| 1064 | 36.95 | 0.21 | GBIF | 14.63 | 79.53 | 18.03 | 17.05 | 136 | 18 | 54.90 | 286 | 163 | 3 |
| 1065 | 37.16 | -2.49 | GBIF | 12.21 | 72.24 | 22.57 | 19.93 | 129 | 2 | 82.19 | 259 | 17 | 4 |
| 1066 | 36.80 | -2.50 | GBIF | 9.91 | 68.81 | 19.78 | 17.23 | 163 | 2 | 87.13 | 321 | 17 | 6 |
| 1067 | 36.97 | -2.43 | GBIF | 10.96 | 69.80 | 21.75 | 19.17 | 131 | 1 | 82.38 | 267 | 13 | 3 |
| 1068 | 37.04 | -2.56 | GBIF | 11.81 | 71.57 | 22.58 | 19.98 | 124 | 1 | 82.33 | 178 | 13 | 5 |
| 1069 | 37.02 | -2.44 | GBIF | 11.03 | 69.83 | 21.58 | 18.95 | 157 | 3 | 80.73 | 205 | 20 | 3 |
| 1070 | 37.58 | 0.33 | GBIF | 13.31 | 81.15 | 22.57 | 22.33 | 206 | 5 | 97.90 | 275 | 324 | 17 |
| 1071 | 37.17 | -2.46 | GBIF | 12.35 | 72.65 | 22.67 | 20.03 | 127 | 2 | 82.13 | 256 | 16 | 4 |
| 1072 | 37.87 | -3.28 | GBIF | 11.44 | 68.51 | 22.92 | 19.90 | 191 | 10 | 88.79 | 189 | 36 | 11 |
| 1073 | 37.14 | -2.57 | GBIF | 12.43 | 72.66 | 22.78 | 20.12 | 125 | 2 | 82.99 | 174 | 14 | 4 |
| 1074 | 37.07 | -2.46 | GBIF | 11.45 | 70.68 | 22.17 | 19.55 | 123 | 2 | 80.75 | 180 | 15 | 3 |
| 1075 | 34.94 | -1.42 | GBIF | 12.63 | 81.98 | 20.22 | 18.67 | 175 | 35 | 40.20 | 311 | 158 | 4 |
| 1076 | 36.95 | 0.24 | GBIF | 14.52 | 79.37 | 18.18 | 17.22 | 146 | 18 | 57.56 | 298 | 159 | 14 |
| 1077 | 37.01 | -2.59 | GBIF | 12.09 | 72.41 | 22.67 | 20.15 | 122 | 1 | 82.32 | 178 | 12 | 4 |
| 1078 | 37.04 | -2.55 | GBIF | 11.63 | 70.93 | 22.53 | 19.95 | 126 | 2 | 82.38 | 177 | 14 | 6 |
| 1079 | 36.35 | -0.55 | GBIF | 15.43 | 79.51 | 17.75 | 17.38 | 102 | 25 | 39.19 | 185 | 143 | 19 |
| 1080 | 37.52 | 0.67 | GBIF | 12.65 | 83.22 | 22.72 | 22.23 | 138 | 4 | 98.14 | 209 | 15 | 10 |
| 1081 | 37.66 | -2.95 | GBIF | 12.00 | 70.18 | 21.35 | 18.73 | 225 | 16 | 82.19 | 275 | 54 | 7 |
| 1082 | 36.62 | -0.73 | GBIF | 12.62 | 77.88 | 13.53 | 11.20 | 235 | 52 | 53.85 | 422 | 190 | 22 |
| 1083 | 36.87 | 0.11 | GBIF | 15.13 | 78.37 | 18.03 | 17.00 | 137 | 22 | 49.67 | 289 | 162 | 11 |
| 1084 | 36.81 | 0.09 | GBIF | 15.06 | 78.43 | 17.75 | 16.65 | 120 | 24 | 44.17 | 260 | 164 | 12 |
| 1085 | 36.83 | 0.35 | GBIF | 15.02 | 80.78 | 18.35 | 17.37 | 128 | 16 | 54.61 | 268 | 166 | 3 |
| 1086 | 36.92 | 0.40 | GBIF | 14.69 | 81.62 | 19.47 | 18.55 | 184 | 21 | 67.00 | 275 | 145 | 5 |
| 1087 | 35.15 | -1.29 | GBIF | 13.05 | 82.08 | 20.22 | 18.67 | 168 | 33 | 40.63 | 301 | 156 | 3 |
| 1088 | 34.85 | -1.30 | GBIF | 11.50 | 80.42 | 18.90 | 17.38 | 223 | 48 | 38.55 | 350 | 213 | 13 |
| 1089 | 36.86 | 0.39 | GBIF | 14.69 | 81.62 | 19.15 | 18.20 | 167 | 19 | 62.36 | 339 | 158 | 12 |
| 1090 | 36.98 | 0.25 | GBIF | 14.34 | 80.12 | 18.38 | 17.48 | 155 | 18 | 60.22 | 236 | 156 | 6 |
| 1091 | 36.93 | 0.22 | GBIF | 14.68 | 79.37 | 18.25 | 17.30 | 152 | 19 | 57.73 | 309 | 160 | 3 |
| 1092 | 35.10 | -1.45 | GBIF | 12.85 | 81.85 | 20.50 | 18.97 | 157 | 28 | 43.42 | 369 | 133 | 9 |
| 1093 | 36.80 | 0.37 | GBIF | 15.05 | 81.35 | 18.48 | 17.53 | 130 | 17 | 54.88 | 271 | 167 | 4 |
| 1094 | 35.01 | -1.50 | GBIF | 12.68 | 81.25 | 20.73 | 19.25 | 162 | 29 | 42.48 | 298 | 139 | 11 |
| 1095 | 36.95 | 0.27 | GBIF | 14.52 | 80.25 | 18.57 | 17.65 | 160 | 20 | 60.33 | 323 | 154 | 11 |
| 1096 | 36.98 | 0.35 | GBIF | 14.68 | 82.03 | 18.90 | 18.00 | 157 | 18 | 63.78 | 318 | 143 | 5 |
| 1097 | 38.20 | -3.32 | GBIF | 10.76 | 68.96 | 22.85 | 19.90 | 138 | 7 | 85.91 | 175 | 24 | 3 |
| 1098 | 37.94 | -0.14 | GBIF | 11.23 | 74.83 | 24.00 | 22.28 | 167 | 0 | 118.48 | 228 | 3 | 8 |
| 1099 | 37.26 | -2.32 | GBIF | 10.98 | 69.90 | 22.20 | 19.47 | 127 | 2 | 83.32 | 254 | 17 | 3 |

| 1100 | 37.06 | -2.47 | GBIF | 11.41 | 70.42 | 22.20 | 19.57 | 123 | 2 | 81.14 | 180 | 14 | 4 |
| --- | --- | --- | --- | --- | --- | --- | --- | --- | --- | --- | --- | --- | --- |
| 1101 | 36.81 | 0.39 | GBIF | 14.93 | 81.60 | 18.53 | 17.62 | 132 | 16 | 56.00 | 273 | 169 | 4 |
| 1102 | 37.51 | 0.69 | GBIF | 12.49 | 83.28 | 22.62 | 22.12 | 140 | 4 | 98.28 | 213 | 15 | 4 |
| 1103 | 38.71 | -3.77 | GBIF | 9.92 | 67.92 | 24.08 | 21.62 | 118 | 17 | 60.54 | 164 | 76 | 5 |
| 1104 | 36.86 | 0.36 | GBIF | 14.81 | 81.36 | 18.57 | 17.65 | 136 | 16 | 56.74 | 281 | 163 | 4 |
| 1105 | 38.33 | -3.60 | GBIF | 10.85 | 67.81 | 22.87 | 19.88 | 123 | 9 | 74.97 | 178 | 38 | 6 |
| 1106 | 37.00 | 0.32 | GBIF | 14.51 | 81.51 | 18.62 | 17.73 | 151 | 17 | 62.39 | 226 | 146 | 5 |
| 1107 | 38.67 | -3.43 | GBIF | 10.55 | 70.33 | 24.62 | 22.13 | 131 | 17 | 65.50 | 259 | 81 | 5 |
| 1108 | 35.08 | -1.39 | GBIF | 12.77 | 81.89 | 20.43 | 18.88 | 169 | 30 | 42.64 | 387 | 146 | 3 |
| 1109 | 37.82 | -3.00 | GBIF | 11.93 | 68.98 | 23.38 | 20.37 | 158 | 5 | 93.83 | 159 | 17 | 5 |
| 1110 | 35.08 | -1.51 | GBIF | 12.74 | 81.16 | 20.32 | 18.83 | 163 | 28 | 44.98 | 298 | 128 | 9 |
| 1111 | 37.81 | -2.96 | GBIF | 12.29 | 69.44 | 23.33 | 20.32 | 160 | 5 | 93.06 | 165 | 18 | 5 |
| 1112 | 35.28 | -1.74 | GBIF | 12.12 | 80.24 | 19.20 | 17.28 | 163 | 21 | 56.26 | 382 | 86 | 5 |
| 1113 | 35.37 | -1.68 | GBIF | 12.31 | 80.98 | 19.83 | 17.88 | 151 | 19 | 57.98 | 358 | 75 | 4 |
| 1114 | 37.86 | -3.22 | GBIF | 11.78 | 69.26 | 23.23 | 20.23 | 195 | 9 | 92.25 | 178 | 32 | 7 |
| 1115 | 35.26 | -1.53 | GBIF | 12.61 | 81.34 | 19.95 | 18.37 | 160 | 24 | 50.55 | 368 | 105 | 6 |
| 1116 | 37.09 | -0.38 | GBIF | 12.25 | 76.09 | 17.67 | 15.75 | 222 | 38 | 68.01 | 354 | 128 | 16 |
| 1117 | 38.85 | -2.68 | GBIF | 9.94 | 69.04 | 26.12 | 23.72 | 160 | 20 | 75.09 | 285 | 66 | 1 |
| 1118 | 37.42 | 0.60 | GBIF | 13.45 | 84.06 | 24.22 | 22.92 | 131 | 6 | 92.42 | 209 | 22 | 13 |
| 1119 | 35.36 | -1.70 | GBIF | 12.19 | 80.74 | 19.17 | 17.23 | 167 | 21 | 57.14 | 385 | 85 | 4 |
| 1120 | 37.45 | 0.45 | GBIF | 13.61 | 84.00 | 22.75 | 22.60 | 163 | 7 | 90.75 | 248 | 259 | 14 |
| 1121 | 37.50 | 0.53 | GBIF | 13.60 | 83.44 | 23.33 | 22.97 | 137 | 5 | 95.39 | 204 | 19 | 6 |
| 1122 | 34.82 | -1.35 | GBIF | 11.67 | 80.46 | 19.37 | 17.88 | 214 | 46 | 38.43 | 346 | 200 | 8 |
| 1123 | 37.87 | -0.17 | GBIF | 11.07 | 74.77 | 23.13 | 21.32 | 192 | 1 | 115.10 | 279 | 7 | 6 |
| 1124 | 35.31 | 1.78 | GBIF | 12.23 | 83.73 | 22.02 | 22.17 | 106 | 15 | 54.38 | 178 | 249 | 5 |
| 1125 | 35.17 | -1.41 | GBIF | 12.82 | 81.69 | 20.42 | 18.80 | 160 | 27 | 45.35 | 369 | 128 | 7 |
| 1126 | 38.91 | -2.67 | GBIF | 9.98 | 69.33 | 26.32 | 23.93 | 152 | 22 | 72.74 | 265 | 68 | 1 |
| 1127 | 35.13 | -1.54 | GBIF | 12.68 | 81.30 | 20.17 | 18.65 | 162 | 26 | 46.73 | 376 | 121 | 2 |
| 1128 | 38.86 | -2.60 | GBIF | 9.81 | 68.59 | 26.25 | 23.83 | 156 | 19 | 76.44 | 265 | 61 | 2 |
| 1129 | 37.27 | -2.76 | GBIF | 12.92 | 72.57 | 22.05 | 19.42 | 182 | 9 | 82.68 | 193 | 38 | 4 |
| 1130 | 37.98 | -3.33 | GBIF | 11.06 | 68.26 | 22.67 | 19.70 | 157 | 9 | 83.80 | 178 | 33 | 3 |
| 1131 | 38.99 | -2.73 | GBIF | 9.93 | 69.41 | 26.32 | 23.95 | 150 | 23 | 69.71 | 257 | 74 | 2 |
| 1132 | 38.99 | -2.67 | GBIF | 9.93 | 68.92 | 26.37 | 24.02 | 150 | 21 | 70.50 | 252 | 70 | 2 |
| 1133 | 34.97 | -1.40 | GBIF | 12.65 | 81.61 | 20.30 | 18.70 | 168 | 34 | 39.90 | 307 | 155 | 12 |
| 1134 | 35.10 | -1.36 | GBIF | 12.98 | 81.60 | 20.18 | 18.63 | 170 | 31 | 42.80 | 388 | 148 | 4 |
| 1135 | 36.87 | -0.36 | GBIF | 12.43 | 78.20 | 16.35 | 14.58 | 190 | 40 | 55.14 | 427 | 158 | 11 |
| 1136 | 34.93 | -1.34 | GBIF | 12.17 | 81.71 | 20.13 | 18.60 | 185 | 39 | 39.55 | 312 | 172 | 11 |
| 1137 | 35.05 | -1.35 | GBIF | 12.74 | 81.68 | 20.10 | 18.53 | 172 | 33 | 40.88 | 300 | 157 | 3 |
| 1138 | 35.19 | -1.34 | GBIF | 12.93 | 81.80 | 20.28 | 18.70 | 158 | 29 | 43.77 | 369 | 134 | 3 |
| 1139 | 38.95 | -3.45 | GBIF | 10.31 | 69.65 | 25.08 | 22.70 | 123 | 21 | 60.01 | 240 | 91 | 2 |
| 1140 | 35.02 | -1.10 | GBIF | 11.86 | 81.78 | 18.78 | 17.28 | 211 | 53 | 36.77 | 323 | 231 | 9 |
| 1141 | 35.13 | -1.10 | GBIF | 12.70 | 82.47 | 19.47 | 17.95 | 191 | 45 | 38.42 | 306 | 198 | 9 |
| 1142 | 39.24 | -4.25 | GBIF | 9.10 | 65.94 | 24.52 | 26.52 | 178 | 22 | 56.62 | 108 | 189 | 7 |
| 1143 | 39.28 | -4.36 | GBIF | 8.71 | 65.48 | 25.20 | 26.93 | 232 | 18 | 66.43 | 102 | 219 | 22 |
| 1144 | 35.03 | -1.15 | GBIF | 11.62 | 81.24 | 18.77 | 17.23 | 207 | 49 | 37.32 | 325 | 219 | 4 |
| 1145 | 39.33 | -4.39 | GBIF | 8.54 | 65.20 | 25.77 | 27.55 | 275 | 19 | 72.20 | 115 | 244 | 9 |
| 1146 | 35.05 | -1.11 | GBIF | 12.18 | 82.26 | 18.93 | 17.45 | 208 | 51 | 37.60 | 320 | 220 | 8 |
| 1147 | 38.75 | -3.28 | GBIF | 10.56 | 69.92 | 25.53 | 22.60 | 149 | 21 | 65.83 | 285 | 76 | 3 |
| 1148 | 39.37 | -4.24 | GBIF | 8.69 | 65.35 | 24.77 | 26.65 | 216 | 22 | 61.87 | 117 | 215 | 10 |
| 1149 | 38.62 | -3.25 | GBIF | 10.41 | 69.85 | 25.12 | 22.17 | 160 | 18 | 70.42 | 302 | 68 | 8 |

| 1150 | 37.33 | -2.67 | GBIF | 12.76 | 72.90 | 22.57 | 19.85 | 148 | 5 | 83.56 | 182 | 24 | 4 |
| --- | --- | --- | --- | --- | --- | --- | --- | --- | --- | --- | --- | --- | --- |
| 1151 | 37.42 | 0.66 | GBIF | 12.79 | 84.16 | 23.37 | 21.90 | 147 | 7 | 93.81 | 247 | 21 | 5 |
| 1152 | 38.99 | -3.29 | GBIF | 10.37 | 70.05 | 25.27 | 22.90 | 133 | 23 | 61.65 | 256 | 97 | 2 |
| 1153 | 34.92 | -1.35 | GBIF | 12.02 | 81.25 | 20.08 | 18.52 | 188 | 41 | 39.56 | 315 | 175 | 11 |
| 1154 | 37.71 | 0.59 | GBIF | 13.09 | 83.39 | 23.48 | 22.85 | 138 | 3 | 107.25 | 183 | 12 | 6 |
| 1155 | 34.80 | -1.25 | GBIF | 11.77 | 81.21 | 20.20 | 18.73 | 202 | 47 | 37.92 | 324 | 205 | 13 |
| 1156 | 36.82 | 0.32 | GBIF | 15.07 | 80.57 | 18.25 | 17.25 | 130 | 16 | 53.93 | 268 | 169 | 2 |
| 1157 | 38.18 | -2.97 | GBIF | 10.23 | 68.68 | 23.17 | 20.22 | 148 | 6 | 91.06 | 271 | 26 | 9 |
| 1158 | 38.52 | -3.23 | GBIF | 10.46 | 69.72 | 24.88 | 21.95 | 160 | 14 | 73.73 | 303 | 58 | 16 |
| 1159 | 38.93 | -3.21 | GBIF | 10.35 | 70.41 | 25.40 | 23.05 | 139 | 22 | 64.25 | 264 | 94 | 3 |
| 1160 | 38.69 | -3.26 | GBIF | 10.45 | 70.13 | 25.35 | 22.40 | 159 | 19 | 68.71 | 298 | 74 | 1 |
| 1161 | 38.26 | -2.27 | GBIF | 9.73 | 67.13 | 23.50 | 20.97 | 185 | 2 | 112.98 | 232 | 8 | 0 |
| 1162 | 38.28 | -3.44 | GBIF | 10.32 | 67.05 | 21.47 | 18.53 | 183 | 18 | 75.04 | 221 | 65 | 15 |
| 1163 | 37.59 | 0.49 | GBIF | 13.49 | 81.77 | 23.35 | 22.95 | 143 | 4 | 100.42 | 194 | 14 | 6 |
| 1164 | 37.44 | 0.57 | GBIF | 13.71 | 84.62 | 23.32 | 22.97 | 131 | 6 | 93.06 | 204 | 20 | 11 |
| 1165 | 34.85 | -1.58 | GBIF | 12.24 | 81.07 | 21.30 | 19.82 | 169 | 29 | 43.01 | 300 | 138 | 7 |
| 1166 | 34.97 | -1.49 | GBIF | 12.49 | 81.64 | 20.37 | 18.78 | 171 | 31 | 42.20 | 305 | 144 | 16 |
| 1167 | 37.85 | -0.14 | GBIF | 11.20 | 75.68 | 23.15 | 21.38 | 195 | 1 | 112.82 | 283 | 8 | 7 |
| 1168 | 34.89 | -1.44 | GBIF | 12.52 | 81.81 | 20.58 | 19.07 | 177 | 35 | 40.09 | 309 | 161 | 6 |
| 1169 | 34.89 | 1.54 | GBIF | 12.41 | 83.28 | 20.97 | 21.33 | 155 | 24 | 51.95 | 261 | 389 | 12 |
| 1170 | 36.85 | 0.35 | GBIF | 14.96 | 80.86 | 18.45 | 17.48 | 135 | 16 | 56.07 | 280 | 166 | 3 |
| 1171 | 37.53 | 0.49 | GBIF | 12.99 | 82.75 | 22.97 | 22.68 | 152 | 5 | 96.26 | 215 | 17 | 10 |
| 1172 | 38.04 | 0.16 | GBIF | 10.99 | 77.41 | 23.22 | 21.82 | 199 | 3 | 112.23 | 260 | 10 | 9 |
| 1173 | 38.00 | 0.08 | GBIF | 11.39 | 76.97 | 23.42 | 21.92 | 193 | 3 | 112.53 | 262 | 10 | 21 |
| 1174 | 35.10 | 1.77 | GBIF | 12.71 | 84.72 | 21.60 | 22.42 | 111 | 15 | 52.90 | 171 | 268 | 5 |
| 1175 | 37.56 | 0.58 | GBIF | 13.23 | 83.23 | 23.43 | 22.95 | 127 | 4 | 100.14 | 177 | 15 | 11 |
| 1176 | 36.90 | 0.09 | GBIF | 14.91 | 78.46 | 17.92 | 16.88 | 140 | 23 | 50.88 | 296 | 156 | 11 |
| 1177 | 37.25 | -2.71 | GBIF | 13.33 | 73.66 | 22.68 | 19.98 | 158 | 6 | 82.72 | 180 | 28 | 4 |
| 1178 | 37.53 | 0.51 | GBIF | 13.33 | 82.82 | 23.27 | 22.88 | 141 | 4 | 97.61 | 200 | 16 | 6 |
| 1179 | 35.26 | -1.47 | GBIF | 12.92 | 81.80 | 20.75 | 18.82 | 148 | 24 | 47.79 | 351 | 109 | 3 |
| 1180 | 35.11 | -1.53 | GBIF | 12.75 | 81.21 | 20.30 | 18.82 | 157 | 27 | 44.60 | 295 | 125 | 9 |
| 1181 | 37.30 | -2.69 | GBIF | 12.82 | 72.87 | 22.52 | 19.80 | 149 | 6 | 82.19 | 179 | 27 | 8 |
| 1182 | 39.85 | -3.27 | GBIF | 7.64 | 67.03 | 24.83 | 25.90 | 194 | 13 | 62.71 | 181 | 166 | 3 |
| 1183 | 37.87 | -0.14 | GBIF | 11.17 | 74.94 | 23.28 | 21.52 | 187 | 1 | 113.66 | 271 | 7 | 7 |
| 1184 | 34.94 | -1.38 | GBIF | 12.57 | 81.60 | 20.32 | 18.77 | 177 | 35 | 40.22 | 307 | 162 | 14 |
| 1185 | 36.90 | 0.55 | GBIF | 14.71 | 83.57 | 19.83 | 18.85 | 191 | 21 | 71.75 | 390 | 131 | 9 |
| 1186 | 39.96 | -3.31 | GBIF | 7.17 | 65.75 | 25.07 | 26.12 | 218 | 9 | 74.38 | 145 | 175 | 10 |
| 1187 | 39.90 | -3.39 | GBIF | 7.18 | 65.30 | 25.22 | 26.30 | 229 | 10 | 74.18 | 157 | 184 | 17 |
| 1188 | 37.55 | 0.54 | GBIF | 13.39 | 82.66 | 23.38 | 22.95 | 136 | 4 | 98.76 | 188 | 16 | 10 |
| 1189 | 36.85 | 1.43 | GBIF | 13.45 | 83.02 | 22.60 | 20.77 | 126 | 10 | 78.02 | 264 | 44 | 14 |
| 1190 | 38.95 | -3.38 | GBIF | 10.56 | 69.92 | 25.13 | 22.77 | 130 | 23 | 61.19 | 256 | 94 | 6 |
| 1191 | 34.81 | -1.30 | GBIF | 11.68 | 80.57 | 19.70 | 18.22 | 211 | 46 | 38.27 | 334 | 204 | 11 |
| 1192 | 34.97 | -1.23 | GBIF | 11.57 | 81.46 | 18.98 | 17.43 | 204 | 45 | 37.93 | 329 | 204 | 4 |
| 1193 | 35.38 | -1.11 | GBIF | 12.52 | 81.86 | 18.13 | 16.43 | 193 | 35 | 47.85 | 410 | 146 | 13 |
| 1194 | 35.33 | -1.52 | GBIF | 12.34 | 80.66 | 19.70 | 18.00 | 175 | 26 | 51.59 | 390 | 111 | 12 |
| 1195 | 38.30 | -3.58 | GBIF | 10.85 | 68.24 | 22.52 | 19.55 | 128 | 11 | 74.67 | 189 | 42 | 6 |
| 1196 | 35.07 | -1.34 | GBIF | 12.84 | 81.28 | 20.07 | 18.52 | 171 | 34 | 41.16 | 391 | 155 | 3 |
| 1197 | 35.21 | -1.46 | GBIF | 12.92 | 81.80 | 20.45 | 18.85 | 152 | 26 | 45.96 | 358 | 119 | 10 |
| 1198 | 35.00 | -1.47 | GBIF | 12.59 | 81.76 | 20.53 | 19.07 | 165 | 31 | 41.69 | 300 | 144 | 10 |
| 1199 | 36.66 | -0.60 | GBIF | 10.60 | 77.94 | 11.43 | 10.78 | 207 | 46 | 38.04 | 401 | 326 | 6 |

| 1200 | 38.19 | -2.91 | GBIF | 10.38 | 68.31 | 22.98 | 20.08 | 165 | 6 | 94.88 | 294 | 24 | 1 |
| --- | --- | --- | --- | --- | --- | --- | --- | --- | --- | --- | --- | --- | --- |
| 1201 | 36.93 | 0.43 | GBIF | 14.81 | 82.73 | 19.35 | 18.42 | 175 | 19 | 67.97 | 351 | 137 | 7 |
| 1202 | 38.64 | -3.18 | GBIF | 10.47 | 69.83 | 25.43 | 22.50 | 171 | 19 | 70.33 | 316 | 73 | 9 |
| 1203 | 38.73 | -3.38 | GBIF | 10.68 | 70.29 | 25.38 | 22.43 | 137 | 21 | 64.27 | 271 | 75 | 7 |
| 1204 | 35.12 | -1.26 | GBIF | 13.06 | 82.13 | 20.10 | 18.58 | 174 | 35 | 41.03 | 298 | 161 | 3 |
| 1205 | 36.91 | 0.54 | GBIF | 14.75 | 83.81 | 19.70 | 18.72 | 185 | 20 | 70.81 | 374 | 132 | 8 |
| 1206 | 38.60 | -3.30 | GBIF | 10.59 | 70.14 | 24.92 | 21.98 | 150 | 18 | 68.72 | 290 | 70 | 1 |
| 1207 | 38.12 | -2.80 | GBIF | 10.48 | 67.63 | 23.43 | 20.43 | 150 | 2 | 101.93 | 254 | 12 | 8 |
| 1208 | 38.06 | -2.94 | GBIF | 10.97 | 68.97 | 23.73 | 20.48 | 135 | 4 | 93.62 | 141 | 16 | 5 |
| 1209 | 35.25 | -1.31 | GBIF | 12.88 | 82.59 | 19.90 | 18.02 | 162 | 28 | 45.62 | 375 | 128 | 7 |
| 1210 | 38.14 | -2.95 | GBIF | 10.77 | 69.02 | 23.57 | 20.48 | 147 | 4 | 93.35 | 150 | 19 | 9 |
| 1211 | 38.74 | -3.39 | GBIF | 10.62 | 70.31 | 25.43 | 22.48 | 132 | 20 | 63.84 | 263 | 74 | 3 |
| 1212 | 38.69 | -2.86 | GBIF | 10.35 | 69.93 | 25.37 | 22.93 | 184 | 23 | 73.23 | 329 | 80 | 2 |
| 1213 | 37.07 | -0.38 | GBIF | 12.45 | 75.45 | 18.35 | 16.40 | 198 | 34 | 67.68 | 320 | 114 | 23 |
| 1214 | 37.80 | -2.13 | GBIF | 11.41 | 69.99 | 23.52 | 21.03 | 164 | 1 | 109.25 | 218 | 5 | 6 |
| 1215 | 37.89 | -0.08 | GBIF | 11.09 | 75.45 | 23.12 | 21.43 | 197 | 2 | 111.80 | 277 | 9 | 9 |
| 1216 | 37.93 | 2.87 | GBIF | 10.37 | 73.52 | 27.35 | 25.03 | 70 | 0 | 106.73 | 53 | 2 | 1 |
| 1217 | 37.86 | 2.59 | GBIF | 11.48 | 77.01 | 26.90 | 24.78 | 79 | 0 | 105.80 | 56 | 4 | 4 |
| 1218 | 37.93 | -2.73 | GBIF | 10.40 | 68.42 | 20.18 | 17.00 | 226 | 6 | 92.62 | 260 | 25 | 4 |
| 1219 | 37.85 | -2.59 | GBIF | 10.55 | 69.41 | 18.87 | 16.00 | 241 | 4 | 94.92 | 271 | 19 | 10 |
| 1220 | 37.70 | -2.42 | GBIF | 11.65 | 68.93 | 22.27 | 19.60 | 174 | 2 | 98.21 | 267 | 11 | 2 |
| 1221 | 37.71 | -2.39 | GBIF | 11.20 | 68.71 | 22.05 | 19.18 | 175 | 2 | 97.39 | 264 | 12 | 1 |
| 1222 | 39.34 | -2.39 | GBIF | 9.46 | 69.04 | 26.72 | 24.48 | 133 | 18 | 66.98 | 200 | 71 | 2 |
| 1223 | 37.90 | -2.52 | GBIF | 10.28 | 68.10 | 22.38 | 19.77 | 173 | 2 | 103.62 | 234 | 10 | 7 |
| 1224 | 37.93 | -2.76 | GBIF | 10.38 | 67.81 | 20.03 | 16.72 | 228 | 9 | 89.84 | 269 | 33 | 7 |
| 1225 | 37.74 | -2.37 | GBIF | 10.79 | 68.30 | 21.72 | 19.17 | 184 | 3 | 94.71 | 266 | 17 | 0 |
| 1226 | 37.77 | -2.35 | GBIF | 10.96 | 69.36 | 22.33 | 19.92 | 172 | 2 | 100.43 | 221 | 11 | 2 |
| 1227 | 36.90 | 0.29 | GBIF | 14.64 | 80.89 | 18.83 | 17.93 | 161 | 20 | 59.20 | 328 | 161 | 17 |
| 1228 | 39.39 | -4.26 | GBIF | 8.26 | 65.54 | 23.63 | 25.47 | 215 | 22 | 59.42 | 118 | 214 | 13 |
| 1229 | 34.86 | -1.40 | GBIF | 12.05 | 81.42 | 20.47 | 18.93 | 186 | 38 | 39.76 | 313 | 169 | 6 |
| 1230 | 37.54 | -0.62 | GBIF | 11.54 | 77.46 | 22.08 | 19.78 | 233 | 5 | 99.47 | 363 | 26 | 21 |
| 1231 | 35.13 | -1.15 | GBIF | 12.90 | 83.23 | 19.77 | 18.22 | 187 | 43 | 38.64 | 306 | 191 | 7 |
| 1232 | 35.31 | 1.38 | GBIF | 12.94 | 82.43 | 19.15 | 20.27 | 142 | 17 | 56.00 | 224 | 360 | 9 |
| 1233 | 35.26 | -1.09 | GBIF | 12.39 | 82.06 | 18.98 | 17.37 | 176 | 38 | 41.88 | 295 | 164 | 9 |
| 1234 | 35.44 | -1.29 | GBIF | 12.82 | 82.21 | 19.05 | 17.03 | 159 | 24 | 50.82 | 362 | 107 | 3 |
| 1235 | 35.43 | -1.38 | GBIF | 13.00 | 81.76 | 19.63 | 17.65 | 158 | 23 | 52.68 | 359 | 99 | 2 |
| 1236 | 35.47 | -1.74 | GBIF | 11.66 | 79.31 | 18.07 | 16.15 | 177 | 19 | 62.25 | 400 | 72 | 4 |
| 1237 | 38.21 | -3.57 | GBIF | 11.16 | 68.88 | 23.20 | 20.23 | 119 | 6 | 78.32 | 174 | 28 | 5 |
| 1238 | 35.02 | -1.51 | GBIF | 12.67 | 81.20 | 20.90 | 19.43 | 162 | 29 | 42.89 | 296 | 136 | 7 |
| 1239 | 35.32 | -1.23 | GBIF | 12.84 | 82.85 | 19.50 | 17.58 | 162 | 28 | 46.48 | 374 | 128 | 4 |
| 1240 | 35.14 | -1.38 | GBIF | 12.95 | 81.96 | 20.57 | 19.02 | 158 | 28 | 43.49 | 286 | 136 | 7 |
| 1241 | 36.91 | 0.22 | GBIF | 14.75 | 79.73 | 18.40 | 17.43 | 155 | 20 | 57.11 | 321 | 164 | 10 |
| 1242 | 37.32 | -2.74 | GBIF | 13.30 | 73.48 | 22.38 | 19.72 | 175 | 9 | 82.31 | 192 | 36 | 5 |
| 1243 | 37.09 | -2.63 | GBIF | 12.97 | 74.10 | 22.90 | 20.28 | 124 | 2 | 81.62 | 174 | 15 | 4 |
| 1244 | 37.48 | 0.44 | GBIF | 12.82 | 82.69 | 22.13 | 21.88 | 185 | 8 | 93.10 | 274 | 291 | 12 |
| 1245 | 37.51 | 0.41 | GBIF | 12.27 | 81.83 | 20.62 | 20.18 | 245 | 8 | 97.50 | 343 | 28 | 11 |
| 1246 | 35.20 | -1.53 | GBIF | 12.81 | 81.58 | 20.32 | 18.75 | 156 | 25 | 48.12 | 363 | 113 | 6 |
| 1247 | 37.46 | 0.45 | GBIF | 13.34 | 83.91 | 22.53 | 22.30 | 172 | 8 | 91.00 | 260 | 274 | 10 |
| 1248 | 37.48 | 0.56 | GBIF | 13.65 | 83.74 | 23.33 | 22.97 | 135 | 5 | 95.41 | 204 | 19 | 7 |
| 1249 | 35.24 | -1.42 | GBIF | 12.91 | 81.70 | 20.75 | 18.80 | 153 | 26 | 46.40 | 357 | 118 | 5 |

| 1250 | 37.37 | -2.65 | GBIF | 13.07 | 73.46 | 22.65 | 19.93 | 158 | 5 | 84.49 | 184 | 26 | 5 |
| --- | --- | --- | --- | --- | --- | --- | --- | --- | --- | --- | --- | --- | --- |
| 1251 | 36.97 | -0.36 | GBIF | 12.74 | 76.76 | 18.12 | 16.30 | 157 | 28 | 60.61 | 369 | 100 | 18 |
| 1252 | 37.29 | -2.74 | GBIF | 13.36 | 73.40 | 22.42 | 19.75 | 178 | 8 | 83.30 | 191 | 33 | 4 |
| 1253 | 37.32 | -2.77 | GBIF | 12.88 | 71.97 | 21.92 | 19.25 | 182 | 10 | 80.57 | 200 | 44 | 5 |
| 1254 | 37.38 | 0.63 | GBIF | 13.43 | 85.02 | 24.03 | 22.72 | 134 | 7 | 91.21 | 228 | 24 | 8 |
| 1255 | 38.63 | -3.13 | GBIF | 10.73 | 70.10 | 25.68 | 22.77 | 186 | 22 | 70.29 | 333 | 81 | 3 |
| 1256 | 36.99 | 0.05 | GBIF | 14.56 | 78.69 | 17.30 | 16.32 | 124 | 23 | 49.70 | 273 | 148 | 12 |
| 1257 | 37.57 | 0.50 | GBIF | 13.48 | 82.16 | 23.32 | 22.95 | 143 | 4 | 99.99 | 197 | 14 | 6 |
| 1258 | 36.96 | 0.17 | GBIF | 15.00 | 78.95 | 18.08 | 17.13 | 140 | 19 | 55.11 | 299 | 162 | 3 |
| 1259 | 36.94 | 0.02 | GBIF | 14.36 | 78.46 | 17.42 | 16.35 | 125 | 27 | 48.37 | 280 | 183 | 12 |
| 1260 | 35.29 | -1.26 | GBIF | 12.95 | 82.48 | 19.70 | 17.78 | 164 | 28 | 46.45 | 378 | 128 | 3 |
| 1261 | 35.34 | -1.59 | GBIF | 12.25 | 80.59 | 19.25 | 17.42 | 178 | 24 | 53.46 | 400 | 105 | 6 |
| 1262 | 36.50 | -1.39 | GBIF | 12.09 | 75.10 | 22.28 | 19.97 | 162 | 11 | 80.59 | 273 | 48 | 6 |
| 1263 | 35.36 | -1.45 | GBIF | 12.87 | 81.43 | 20.45 | 18.43 | 150 | 22 | 51.33 | 350 | 97 | 5 |
| 1264 | 38.28 | -2.55 | GBIF | 10.44 | 69.15 | 24.67 | 22.10 | 162 | 3 | 103.85 | 259 | 17 | 6 |
| 1265 | 35.25 | -1.43 | GBIF | 12.93 | 81.29 | 20.30 | 18.67 | 150 | 26 | 46.27 | 355 | 115 | 5 |
| 1266 | 39.87 | -3.38 | GBIF | 7.16 | 65.67 | 24.92 | 26.00 | 222 | 12 | 69.34 | 164 | 183 | 12 |
| 1267 | 39.46 | -4.02 | GBIF | 8.82 | 65.86 | 25.10 | 26.95 | 190 | 18 | 58.50 | 101 | 189 | 15 |
| 1268 | 39.59 | -4.20 | GBIF | 8.14 | 64.62 | 26.05 | 27.75 | 264 | 17 | 74.88 | 270 | 203 | 16 |
| 1269 | 37.26 | -2.73 | GBIF | 13.21 | 73.38 | 22.38 | 19.73 | 166 | 7 | 82.39 | 185 | 32 | 4 |
| 1270 | 37.52 | 0.52 | GBIF | 13.38 | 82.56 | 23.23 | 22.90 | 142 | 5 | 96.61 | 203 | 17 | 6 |
| 1271 | 35.96 | -1.25 | GBIF | 12.58 | 78.16 | 19.33 | 17.30 | 153 | 22 | 60.15 | 304 | 88 | 3 |
| 1272 | 38.03 | -2.82 | GBIF | 9.92 | 67.00 | 22.82 | 19.58 | 157 | 2 | 100.57 | 159 | 11 | 1 |
| 1273 | 37.52 | 0.46 | GBIF | 12.50 | 81.70 | 22.38 | 22.15 | 176 | 6 | 95.32 | 257 | 277 | 10 |
| 1274 | 35.23 | -1.56 | GBIF | 12.70 | 81.41 | 20.05 | 18.53 | 161 | 24 | 49.66 | 370 | 111 | 6 |
| 1275 | 37.55 | 0.55 | GBIF | 13.33 | 82.82 | 23.47 | 23.00 | 129 | 4 | 99.29 | 177 | 15 | 20 |
| 1276 | 37.34 | -2.64 | GBIF | 12.95 | 73.58 | 22.63 | 19.95 | 156 | 5 | 84.29 | 184 | 25 | 4 |
| 1277 | 38.14 | 0.14 | GBIF | 10.98 | 76.81 | 24.02 | 22.62 | 169 | 1 | 119.22 | 212 | 4 | 8 |
| 1278 | 37.20 | 0.18 | GBIF | 14.18 | 80.54 | 17.23 | 16.48 | 135 | 18 | 59.13 | 208 | 153 | 5 |
| 1279 | 38.17 | 0.00 | GBIF | 11.23 | 75.84 | 24.65 | 23.13 | 159 | 0 | 121.98 | 187 | 1 | 4 |
| 1280 | 37.31 | -2.73 | GBIF | 13.42 | 73.32 | 22.52 | 19.85 | 177 | 8 | 83.65 | 188 | 34 | 5 |
| 1281 | 38.08 | 0.11 | GBIF | 10.41 | 76.53 | 23.65 | 22.15 | 177 | 1 | 117.44 | 227 | 6 | 0 |
| 1282 | 38.06 | 0.04 | GBIF | 11.02 | 76.03 | 23.75 | 22.22 | 179 | 1 | 117.84 | 226 | 4 | 13 |
| 1283 | 38.09 | 0.16 | GBIF | 11.09 | 77.03 | 23.68 | 22.25 | 181 | 1 | 116.92 | 233 | 7 | 2 |
| 1284 | 37.57 | 0.52 | GBIF | 13.63 | 82.08 | 23.42 | 23.02 | 138 | 4 | 100.41 | 189 | 14 | 6 |
| 1285 | 35.26 | -1.41 | GBIF | 12.88 | 82.06 | 20.58 | 18.65 | 149 | 25 | 46.65 | 354 | 114 | 3 |
| 1286 | 37.21 | -2.77 | GBIF | 12.32 | 71.24 | 21.68 | 19.05 | 168 | 9 | 79.62 | 196 | 38 | 4 |
| 1287 | 37.41 | 0.75 | GBIF | 12.99 | 84.91 | 23.32 | 22.00 | 143 | 6 | 94.38 | 241 | 21 | 2 |
| 1288 | 37.58 | 0.67 | GBIF | 12.85 | 83.44 | 23.12 | 22.63 | 130 | 3 | 101.59 | 182 | 13 | 6 |
| 1289 | 37.30 | -2.73 | GBIF | 13.43 | 73.41 | 22.50 | 19.85 | 172 | 8 | 82.90 | 188 | 33 | 4 |
| 1290 | 37.30 | -2.65 | GBIF | 12.50 | 72.67 | 22.55 | 19.83 | 144 | 4 | 84.31 | 177 | 22 | 6 |
| 1291 | 36.69 | -0.51 | GBIF | 10.98 | 79.02 | 10.93 | 10.25 | 202 | 41 | 36.97 | 393 | 332 | 5 |
| 1292 | 36.93 | 0.00 | GBIF | 14.47 | 78.62 | 17.65 | 16.48 | 129 | 28 | 48.90 | 290 | 187 | 13 |
| 1293 | 34.63 | -1.21 | GBIF | 11.70 | 80.69 | 20.95 | 19.60 | 194 | 49 | 38.93 | 312 | 198 | 12 |
| 1294 | 35.58 | -1.70 | GBIF | 11.88 | 79.17 | 19.08 | 17.00 | 157 | 15 | 64.72 | 361 | 59 | 8 |
| 1295 | 38.67 | -3.34 | GBIF | 10.79 | 70.53 | 25.20 | 22.27 | 144 | 18 | 67.00 | 283 | 72 | 1 |
| 1296 | 35.52 | -1.62 | GBIF | 11.93 | 79.56 | 18.00 | 16.23 | 179 | 23 | 58.30 | 396 | 87 | 6 |
| 1297 | 38.77 | -2.34 | GBIF | 10.08 | 69.06 | 26.17 | 23.72 | 163 | 13 | 88.47 | 239 | 39 | 0 |
| 1298 | 37.37 | -2.67 | GBIF | 13.02 | 73.17 | 22.65 | 19.93 | 156 | 6 | 83.64 | 184 | 27 | 5 |
| 1299 | 38.05 | -2.99 | GBIF | 10.69 | 68.54 | 24.10 | 20.75 | 115 | 4 | 91.00 | 126 | 16 | 2 |

| 1300 | 38.77 | -3.23 | GBIF | 10.55 | 70.33 | 25.68 | 22.70 | 156 | 22 | 66.56 | 295 | 78 | 10 |
| --- | --- | --- | --- | --- | --- | --- | --- | --- | --- | --- | --- | --- | --- |
| 1301 | 38.65 | -3.23 | GBIF | 10.34 | 69.88 | 25.28 | 22.35 | 163 | 19 | 70.45 | 306 | 87 | 5 |
| 1302 | 37.32 | -2.69 | GBIF | 13.09 | 73.14 | 22.57 | 19.87 | 152 | 6 | 82.57 | 180 | 28 | 9 |
| 1303 | 37.66 | 0.54 | GBIF | 13.58 | 81.83 | 23.40 | 22.92 | 143 | 3 | 104.92 | 193 | 13 | 14 |
| 1304 | 37.66 | 0.46 | GBIF | 13.53 | 81.04 | 23.27 | 22.85 | 162 | 3 | 103.30 | 218 | 12 | 5 |
| 1305 | 35.04 | -1.41 | GBIF | 12.65 | 81.61 | 20.70 | 19.13 | 161 | 31 | 41.42 | 376 | 146 | 5 |
| 1306 | 35.26 | -1.73 | GBIF | 12.03 | 80.22 | 19.00 | 17.10 | 173 | 23 | 54.90 | 399 | 95 | 5 |
| 1307 | 37.57 | 0.53 | GBIF | 13.54 | 82.57 | 23.40 | 22.98 | 136 | 4 | 99.53 | 188 | 16 | 6 |
| 1308 | 36.81 | 0.07 | GBIF | 14.98 | 78.40 | 17.63 | 16.52 | 115 | 26 | 41.93 | 252 | 166 | 3 |
| 1309 | 36.80 | 0.01 | GBIF | 14.73 | 78.79 | 17.23 | 16.07 | 108 | 30 | 39.78 | 239 | 180 | 4 |
| 1310 | 36.82 | -1.24 | GBIF | 11.25 | 73.05 | 20.25 | 17.72 | 206 | 19 | 77.34 | 336 | 76 | 38 |
| 1311 | 35.16 | -1.38 | GBIF | 12.92 | 81.75 | 20.43 | 18.88 | 160 | 28 | 44.28 | 287 | 133 | 11 |
| 1312 | 37.62 | 0.79 | GBIF | 13.04 | 84.14 | 23.32 | 22.75 | 131 | 3 | 104.98 | 191 | 12 | 4 |
| 1313 | 36.84 | 0.15 | GBIF | 15.37 | 78.40 | 18.05 | 17.08 | 140 | 21 | 51.40 | 283 | 168 | 3 |
| 1314 | 36.86 | 0.20 | GBIF | 15.02 | 78.66 | 18.00 | 16.95 | 133 | 19 | 51.67 | 282 | 166 | 3 |
| 1315 | 36.97 | 0.20 | GBIF | 14.61 | 79.39 | 17.87 | 16.92 | 135 | 17 | 55.20 | 283 | 164 | 3 |
| 1316 | 39.42 | -4.24 | GBIF | 8.10 | 65.32 | 23.47 | 25.23 | 213 | 23 | 57.04 | 126 | 218 | 12 |
| 1317 | 36.79 | -0.29 | GBIF | 13.09 | 80.32 | 15.33 | 13.68 | 189 | 45 | 50.87 | 420 | 171 | 18 |
| 1318 | 35.04 | -1.31 | GBIF | 12.69 | 81.36 | 20.28 | 18.75 | 170 | 35 | 40.07 | 296 | 159 | 5 |
| 1319 | 36.73 | -0.24 | GBIF | 12.80 | 81.01 | 14.08 | 13.03 | 160 | 38 | 46.64 | 414 | 180 | 17 |
| 1320 | 36.94 | 0.35 | GBIF | 14.63 | 81.30 | 19.17 | 18.27 | 170 | 20 | 64.19 | 257 | 147 | 4 |
| 1321 | 38.71 | -3.02 | GBIF | 10.49 | 69.94 | 26.00 | 23.02 | 177 | 26 | 67.81 | 319 | 90 | 5 |
| 1322 | 36.81 | 0.36 | GBIF | 15.08 | 81.09 | 18.43 | 17.50 | 131 | 16 | 55.26 | 272 | 168 | 3 |
| 1323 | 37.30 | 2.22 | GBIF | 12.42 | 82.83 | 26.65 | 24.82 | 80 | 1 | 98.34 | 127 | 10 | 1 |
| 1324 | 38.12 | -2.91 | GBIF | 10.83 | 68.57 | 23.13 | 20.08 | 154 | 4 | 95.28 | 162 | 19 | 4 |
| 1325 | 37.00 | 0.37 | GBIF | 14.68 | 82.03 | 19.05 | 18.18 | 162 | 19 | 65.00 | 245 | 138 | 5 |
| 1326 | 35.14 | -1.20 | GBIF | 13.08 | 82.81 | 19.70 | 18.17 | 186 | 40 | 39.74 | 308 | 181 | 14 |
| 1327 | 38.25 | -2.29 | GBIF | 9.66 | 67.07 | 23.48 | 20.95 | 185 | 2 | 113.47 | 230 | 8 | 1 |
| 1328 | 36.87 | 0.27 | GBIF | 14.78 | 80.34 | 18.05 | 17.05 | 129 | 16 | 53.51 | 268 | 167 | 2 |
| 1329 | 36.95 | 0.33 | GBIF | 14.56 | 81.33 | 18.95 | 18.02 | 162 | 19 | 63.12 | 331 | 147 | 5 |
| 1330 | 36.80 | 0.30 | GBIF | 15.14 | 80.11 | 18.25 | 17.25 | 133 | 17 | 53.88 | 277 | 170 | 3 |
| 1331 | 36.87 | 0.23 | GBIF | 14.88 | 79.55 | 18.17 | 17.18 | 143 | 18 | 54.92 | 297 | 168 | 3 |
| 1332 | 34.96 | -1.37 | GBIF | 12.62 | 81.93 | 20.35 | 18.87 | 178 | 35 | 41.06 | 304 | 160 | 11 |
| 1333 | 37.35 | -2.63 | GBIF | 13.13 | 73.32 | 22.63 | 19.95 | 153 | 5 | 83.74 | 186 | 25 | 5 |
| 1334 | 36.86 | 0.21 | GBIF | 14.92 | 78.92 | 18.15 | 17.15 | 142 | 19 | 54.25 | 301 | 167 | 3 |
| 1335 | 36.88 | 0.21 | GBIF | 14.87 | 79.50 | 18.25 | 17.23 | 147 | 19 | 55.23 | 308 | 167 | 10 |
| 1336 | 36.85 | 0.41 | GBIF | 14.62 | 81.66 | 19.18 | 18.23 | 172 | 20 | 63.71 | 346 | 157 | 12 |
| 1337 | 34.88 | -1.32 | GBIF | 11.35 | 81.07 | 18.33 | 16.78 | 226 | 47 | 39.65 | 353 | 207 | 12 |
| 1338 | 34.83 | -1.28 | GBIF | 11.67 | 81.02 | 19.72 | 18.25 | 213 | 46 | 38.22 | 337 | 208 | 14 |
| 1339 | 35.02 | -1.47 | GBIF | 12.62 | 81.40 | 20.67 | 19.18 | 159 | 30 | 41.56 | 296 | 141 | 10 |
| 1340 | 38.20 | -2.75 | GBIF | 10.39 | 67.48 | 23.97 | 21.05 | 150 | 4 | 99.00 | 261 | 19 | 1 |
| 1341 | 37.40 | 0.58 | GBIF | 13.55 | 84.69 | 24.40 | 23.10 | 125 | 6 | 91.55 | 199 | 23 | 10 |
| 1342 | 37.27 | -2.61 | GBIF | 12.63 | 73.03 | 22.70 | 20.02 | 148 | 3 | 84.87 | 178 | 20 | 8 |
| 1343 | 38.16 | -2.79 | GBIF | 10.57 | 67.74 | 23.70 | 20.72 | 149 | 3 | 99.37 | 261 | 16 | 1 |
| 1344 | 37.77 | 0.75 | GBIF | 12.22 | 83.11 | 23.10 | 22.43 | 148 | 2 | 109.88 | 198 | 9 | 8 |
| 1345 | 35.30 | -1.57 | GBIF | 12.33 | 81.09 | 19.47 | 17.83 | 176 | 25 | 51.46 | 393 | 111 | 6 |
| 1346 | 37.39 | -2.74 | GBIF | 13.10 | 72.78 | 22.38 | 19.70 | 175 | 9 | 82.56 | 194 | 37 | 9 |
| 1347 | 34.82 | -1.45 | GBIF | 12.28 | 81.89 | 20.80 | 19.40 | 183 | 36 | 40.19 | 313 | 165 | 7 |
| 1348 | 37.94 | -3.46 | GBIF | 11.13 | 68.25 | 22.57 | 19.57 | 153 | 11 | 79.87 | 195 | 37 | 2 |
| 1349 | 38.23 | -3.42 | GBIF | 10.77 | 68.14 | 21.85 | 18.93 | 152 | 11 | 79.66 | 200 | 41 | 6 |

| 1350 | 37.47 | 0.42 | GBIF | 13.27 | 83.44 | 22.18 | 21.93 | 187 | 8 | 92.19 | 283 | 294 | 8 |
| --- | --- | --- | --- | --- | --- | --- | --- | --- | --- | --- | --- | --- | --- |
| 1351 | 37.54 | 0.57 | GBIF | 13.25 | 83.33 | 23.58 | 23.05 | 124 | 4 | 99.80 | 171 | 15 | 19 |
| 1352 | 39.87 | -3.32 | GBIF | 7.33 | 66.59 | 24.85 | 25.88 | 203 | 13 | 65.15 | 174 | 169 | 3 |
| 1353 | 37.51 | 0.46 | GBIF | 12.60 | 82.35 | 21.65 | 21.35 | 206 | 7 | 95.81 | 299 | 23 | 6 |
| 1354 | 36.62 | -0.43 | GBIF | 10.77 | 76.90 | 10.60 | 10.45 | 193 | 41 | 35.11 | 371 | 388 | 8 |
| 1355 | 39.93 | -3.23 | GBIF | 7.37 | 66.37 | 24.65 | 25.73 | 202 | 11 | 67.05 | 162 | 200 | 5 |
| 1356 | 35.32 | -1.15 | GBIF | 12.49 | 82.18 | 18.88 | 17.18 | 178 | 33 | 45.31 | 394 | 145 | 5 |
| 1357 | 36.96 | 0.36 | GBIF | 14.63 | 81.75 | 19.13 | 18.22 | 168 | 20 | 64.77 | 255 | 143 | 5 |
| 1358 | 38.54 | -3.30 | GBIF | 10.48 | 69.83 | 23.98 | 21.55 | 149 | 13 | 74.60 | 291 | 51 | 9 |
| 1359 | 39.57 | -4.31 | GBIF | 7.90 | 64.23 | 26.02 | 27.82 | 291 | 17 | 79.74 | 100 | 214 | 20 |
| 1360 | 36.93 | 0.32 | GBIF | 14.58 | 81.02 | 19.07 | 18.17 | 173 | 20 | 63.48 | 262 | 151 | 4 |
| 1361 | 38.43 | -3.25 | GBIF | 10.34 | 70.35 | 23.83 | 21.42 | 144 | 11 | 78.00 | 283 | 43 | 13 |
| 1362 | 36.99 | 0.29 | GBIF | 14.45 | 81.18 | 18.57 | 17.65 | 154 | 18 | 61.02 | 311 | 151 | 5 |
| 1363 | 36.95 | 0.44 | GBIF | 14.81 | 82.73 | 19.35 | 18.42 | 176 | 19 | 68.77 | 353 | 135 | 7 |
| 1364 | 38.42 | -3.23 | GBIF | 10.28 | 69.95 | 23.57 | 21.12 | 149 | 10 | 80.24 | 289 | 40 | 8 |
| 1365 | 36.90 | 0.27 | GBIF | 14.65 | 80.05 | 18.55 | 17.63 | 158 | 19 | 58.99 | 321 | 162 | 3 |
| 1366 | 38.47 | -3.25 | GBIF | 9.96 | 69.16 | 23.82 | 21.38 | 143 | 12 | 75.45 | 283 | 48 | 13 |
| 1367 | 35.06 | -1.58 | GBIF | 12.80 | 81.53 | 20.78 | 19.17 | 154 | 26 | 44.67 | 293 | 121 | 12 |
| 1368 | 38.37 | -2.90 | GBIF | 10.33 | 69.35 | 24.98 | 21.95 | 180 | 15 | 81.63 | 319 | 55 | 14 |
| 1369 | 36.99 | -0.59 | GBIF | 11.67 | 74.31 | 18.27 | 16.15 | 324 | 39 | 84.52 | 451 | 145 | 13 |
| 1370 | 38.66 | -3.26 | GBIF | 10.44 | 70.08 | 25.27 | 22.33 | 161 | 18 | 69.67 | 301 | 72 | 1 |
| 1371 | 36.89 | 0.27 | GBIF | 14.72 | 79.98 | 18.38 | 17.43 | 145 | 18 | 56.35 | 296 | 164 | 2 |
| 1372 | 37.31 | -2.66 | GBIF | 12.57 | 72.64 | 22.48 | 19.75 | 150 | 5 | 84.59 | 179 | 23 | 4 |
| 1373 | 37.95 | -2.59 | GBIF | 10.87 | 69.66 | 22.60 | 20.08 | 168 | 1 | 105.31 | 242 | 7 | 11 |
| 1374 | 36.79 | 0.89 | GBIF | 14.76 | 83.38 | 18.88 | 17.73 | 128 | 19 | 57.17 | 273 | 128 | 20 |
| 1375 | 38.82 | -3.60 | GBIF | 10.22 | 69.50 | 24.63 | 22.27 | 119 | 19 | 60.61 | 233 | 80 | 11 |
| 1376 | 38.91 | -3.73 | GBIF | 9.78 | 67.94 | 24.60 | 22.23 | 110 | 24 | 54.61 | 155 | 88 | 10 |
| 1377 | 38.79 | -3.53 | GBIF | 10.53 | 70.17 | 24.72 | 22.28 | 123 | 19 | 61.29 | 241 | 81 | 3 |
| 1378 | 37.23 | -2.67 | GBIF | 13.02 | 73.54 | 22.63 | 19.88 | 141 | 4 | 83.08 | 174 | 21 | 4 |
| 1379 | 38.04 | -2.79 | GBIF | 10.10 | 67.79 | 23.08 | 19.98 | 151 | 1 | 104.69 | 145 | 8 | 1 |
| 1380 | 36.94 | 0.08 | GBIF | 14.79 | 78.68 | 17.63 | 16.62 | 130 | 23 | 49.85 | 281 | 153 | 18 |
| 1381 | 37.39 | -2.80 | GBIF | 12.09 | 70.71 | 21.18 | 18.52 | 202 | 12 | 78.64 | 225 | 60 | 7 |
| 1382 | 37.32 | -2.67 | GBIF | 12.64 | 72.24 | 22.50 | 19.75 | 150 | 5 | 83.60 | 180 | 25 | 4 |
| 1383 | 38.73 | -3.49 | GBIF | 10.51 | 69.59 | 24.58 | 22.15 | 127 | 18 | 64.03 | 171 | 82 | 3 |
| 1384 | 34.87 | -1.45 | GBIF | 12.52 | 81.86 | 20.78 | 19.28 | 175 | 35 | 40.11 | 307 | 159 | 7 |
| 1385 | 34.90 | -1.20 | GBIF | 11.25 | 80.94 | 19.12 | 17.60 | 218 | 49 | 37.50 | 332 | 225 | 3 |
| 1386 | 35.01 | -1.30 | GBIF | 12.45 | 81.37 | 20.28 | 18.77 | 174 | 37 | 39.09 | 396 | 168 | 12 |
| 1387 | 35.07 | -1.24 | GBIF | 12.87 | 82.48 | 20.08 | 18.62 | 183 | 39 | 39.58 | 303 | 180 | 7 |
| 1388 | 38.17 | -2.71 | GBIF | 10.58 | 68.23 | 23.98 | 21.10 | 150 | 3 | 102.01 | 254 | 16 | 8 |
| 1389 | 36.53 | -0.64 | GBIF | 13.26 | 77.53 | 13.97 | 11.77 | 160 | 45 | 45.16 | 295 | 157 | 22 |
| 1390 | 38.16 | -2.68 | GBIF | 10.81 | 68.41 | 24.18 | 21.25 | 150 | 2 | 102.82 | 252 | 15 | 9 |
| 1391 | 38.93 | -3.24 | GBIF | 10.40 | 70.27 | 25.33 | 22.95 | 137 | 22 | 63.00 | 266 | 98 | 7 |
| 1392 | 38.98 | -3.64 | GBIF | 10.01 | 68.55 | 24.63 | 22.23 | 114 | 23 | 55.13 | 217 | 88 | 4 |
| 1393 | 38.97 | -3.56 | GBIF | 10.09 | 69.60 | 24.93 | 22.57 | 115 | 22 | 57.55 | 216 | 86 | 2 |
| 1394 | 36.47 | -0.75 | GBIF | 14.08 | 78.24 | 17.00 | 14.78 | 129 | 36 | 48.73 | 229 | 123 | 20 |
| 1395 | 38.70 | -3.41 | GBIF | 10.60 | 70.20 | 25.22 | 22.27 | 134 | 19 | 65.04 | 269 | 86 | 2 |
| 1396 | 38.08 | -2.79 | GBIF | 10.31 | 67.82 | 23.25 | 20.17 | 151 | 1 | 104.25 | 145 | 8 | 0 |
| 1397 | 38.80 | -3.50 | GBIF | 10.50 | 70.00 | 24.80 | 22.40 | 125 | 21 | 61.38 | 245 | 86 | 2 |
| 1398 | 38.13 | -2.94 | GBIF | 10.78 | 68.63 | 23.55 | 20.48 | 146 | 4 | 93.64 | 147 | 19 | 5 |
| 1399 | 36.85 | 0.72 | GBIF | 14.77 | 84.38 | 18.80 | 17.75 | 140 | 18 | 61.10 | 289 | 129 | 5 |

| 1400 | 38.81 | -3.34 | GBIF | 10.80 | 70.59 | 25.58 | 22.63 | 138 | 21 | 63.68 | 275 | 92 | 11 |
| --- | --- | --- | --- | --- | --- | --- | --- | --- | --- | --- | --- | --- | --- |
| 1401 | 37.24 | -2.61 | GBIF | 12.72 | 73.13 | 22.68 | 19.98 | 145 | 3 | 83.93 | 177 | 20 | 4 |
| 1402 | 38.70 | -3.29 | GBIF | 10.54 | 69.81 | 25.40 | 22.43 | 155 | 19 | 68.06 | 293 | 73 | 5 |
| 1403 | 37.07 | -2.66 | GBIF | 12.91 | 74.19 | 22.85 | 20.28 | 128 | 2 | 82.93 | 173 | 14 | 4 |
| 1404 | 34.96 | -1.27 | GBIF | 11.87 | 81.84 | 18.78 | 17.27 | 210 | 44 | 39.47 | 331 | 197 | 4 |
| 1405 | 34.95 | -1.50 | GBIF | 12.54 | 81.44 | 20.53 | 18.97 | 176 | 30 | 43.12 | 306 | 143 | 13 |
| 1406 | 34.95 | -1.35 | GBIF | 12.39 | 82.06 | 20.23 | 18.68 | 181 | 38 | 39.68 | 309 | 169 | 14 |
| 1407 | 38.73 | -3.68 | GBIF | 10.42 | 68.98 | 23.98 | 21.48 | 124 | 18 | 61.35 | 168 | 82 | 16 |
| 1408 | 35.22 | -1.43 | GBIF | 12.90 | 81.65 | 20.50 | 18.90 | 152 | 26 | 45.82 | 357 | 119 | 11 |
| 1409 | 38.07 | -2.82 | GBIF | 10.27 | 67.60 | 23.35 | 20.18 | 145 | 2 | 102.87 | 142 | 9 | 0 |
| 1410 | 35.17 | -1.23 | GBIF | 13.00 | 82.80 | 19.72 | 18.17 | 185 | 36 | 41.74 | 410 | 167 | 3 |
| 1411 | 37.51 | 0.57 | GBIF | 13.42 | 83.33 | 23.55 | 23.23 | 124 | 4 | 97.23 | 177 | 17 | 19 |
| 1412 | 38.67 | -3.14 | GBIF | 10.86 | 70.51 | 25.90 | 22.95 | 178 | 22 | 68.74 | 323 | 81 | 3 |
| 1413 | 38.64 | -3.50 | GBIF | 10.15 | 69.05 | 24.15 | 21.73 | 127 | 16 | 66.02 | 254 | 63 | 5 |
| 1414 | 37.65 | 0.50 | GBIF | 13.63 | 81.59 | 23.38 | 22.95 | 148 | 3 | 103.48 | 203 | 12 | 9 |
| 1415 | 36.89 | -0.33 | GBIF | 12.83 | 77.31 | 16.93 | 15.23 | 161 | 34 | 54.19 | 359 | 123 | 15 |
| 1416 | 40.02 | -1.95 | GBIF | 8.91 | 67.49 | 28.40 | 28.95 | 94 | 11 | 49.71 | 156 | 115 | 4 |
| 1417 | 35.16 | -1.56 | GBIF | 12.69 | 80.84 | 20.20 | 18.72 | 160 | 25 | 48.13 | 370 | 114 | 3 |
| 1418 | 38.19 | -2.87 | GBIF | 10.43 | 68.14 | 23.48 | 20.53 | 159 | 5 | 95.27 | 276 | 21 | 4 |
| 1419 | 39.30 | -4.38 | GBIF | 8.79 | 65.61 | 25.35 | 27.13 | 243 | 20 | 67.03 | 120 | 227 | 22 |
| 1420 | 37.89 | 0.72 | GBIF | 12.79 | 84.16 | 24.20 | 23.40 | 133 | 1 | 116.37 | 175 | 5 | 7 |
| 1421 | 36.90 | 0.31 | GBIF | 14.66 | 80.99 | 18.52 | 17.60 | 145 | 17 | 57.46 | 291 | 164 | 14 |
| 1422 | 38.22 | -2.28 | GBIF | 9.93 | 67.98 | 24.07 | 21.45 | 175 | 2 | 113.41 | 225 | 8 | 2 |
| 1423 | 38.23 | -2.33 | GBIF | 9.76 | 67.30 | 24.13 | 21.53 | 172 | 2 | 111.69 | 229 | 10 | 2 |
| 1424 | 35.61 | -1.33 | GBIF | 13.23 | 82.14 | 19.00 | 16.98 | 142 | 20 | 55.45 | 331 | 79 | 11 |
| 1425 | 37.47 | 0.21 | GBIF | 13.41 | 82.77 | 18.73 | 17.90 | 290 | 13 | 95.85 | 383 | 44 | 15 |
| 1426 | 37.41 | 0.47 | GBIF | 14.24 | 84.77 | 24.02 | 22.77 | 154 | 8 | 89.50 | 258 | 245 | 15 |
| 1427 | 39.84 | -3.39 | GBIF | 7.44 | 66.44 | 24.57 | 25.68 | 215 | 14 | 66.15 | 182 | 174 | 4 |
| 1428 | 37.25 | -2.63 | GBIF | 12.68 | 73.31 | 22.65 | 19.88 | 139 | 3 | 83.14 | 268 | 20 | 8 |
| 1429 | 35.39 | -1.50 | GBIF | 12.42 | 81.15 | 20.00 | 18.00 | 166 | 24 | 52.94 | 374 | 101 | 4 |
| 1430 | 36.80 | 0.16 | GBIF | 15.29 | 78.42 | 17.97 | 16.97 | 129 | 20 | 49.21 | 266 | 169 | 3 |
| 1431 | 36.93 | -0.17 | GBIF | 13.97 | 78.46 | 17.47 | 16.18 | 127 | 25 | 52.55 | 292 | 234 | 11 |
| 1432 | 37.38 | -2.61 | GBIF | 12.86 | 73.06 | 22.45 | 19.77 | 154 | 5 | 83.82 | 186 | 26 | 6 |
| 1433 | 38.37 | -2.54 | GBIF | 9.78 | 67.94 | 23.95 | 21.48 | 178 | 3 | 105.01 | 265 | 16 | 5 |
| 1434 | 35.10 | -1.59 | GBIF | 12.69 | 81.88 | 20.53 | 19.13 | 156 | 25 | 47.24 | 292 | 114 | 9 |
| 1435 | 37.28 | -2.72 | GBIF | 13.47 | 73.59 | 22.62 | 19.93 | 167 | 7 | 83.26 | 183 | 30 | 4 |
| 1436 | 36.85 | -0.06 | GBIF | 14.43 | 78.83 | 17.47 | 16.23 | 123 | 34 | 44.28 | 278 | 197 | 11 |
| 1437 | 37.78 | 0.58 | GBIF | 12.26 | 82.27 | 23.23 | 22.53 | 153 | 2 | 110.28 | 197 | 10 | 10 |
| 1438 | 37.79 | 0.54 | GBIF | 12.23 | 82.05 | 23.05 | 22.42 | 168 | 3 | 109.22 | 215 | 11 | 6 |
| 1439 | 39.82 | -3.24 | GBIF | 8.23 | 68.04 | 25.22 | 26.40 | 167 | 14 | 56.91 | 168 | 168 | 13 |
| 1440 | 37.70 | 0.55 | GBIF | 13.23 | 82.19 | 23.35 | 22.82 | 148 | 3 | 107.15 | 192 | 11 | 6 |
| 1441 | 37.16 | 1.39 | GBIF | 11.81 | 81.44 | 22.08 | 20.37 | 141 | 6 | 90.51 | 231 | 21 | 1 |
| 1442 | 38.82 | -3.73 | GBIF | 9.85 | 67.93 | 24.38 | 22.02 | 113 | 20 | 56.93 | 159 | 82 | 3 |
| 1443 | 38.76 | -3.20 | GBIF | 10.54 | 70.75 | 25.63 | 22.70 | 160 | 22 | 66.96 | 301 | 98 | 2 |
| 1444 | 38.85 | -3.66 | GBIF | 9.98 | 68.79 | 24.52 | 22.18 | 115 | 21 | 56.99 | 160 | 87 | 3 |
| 1445 | 34.91 | -1.36 | GBIF | 12.19 | 81.28 | 20.20 | 18.67 | 186 | 39 | 39.55 | 313 | 173 | 10 |
| 1446 | 38.91 | -3.52 | GBIF | 10.18 | 69.27 | 24.80 | 22.42 | 122 | 22 | 60.08 | 236 | 87 | 7 |
| 1447 | 38.87 | -3.49 | GBIF | 10.40 | 69.80 | 24.95 | 22.55 | 124 | 20 | 61.37 | 240 | 86 | 3 |
| 1448 | 37.35 | -2.46 | GBIF | 11.98 | 71.33 | 22.48 | 19.92 | 130 | 2 | 84.63 | 253 | 18 | 5 |
| 1449 | 38.93 | -3.80 | GBIF | 9.63 | 67.84 | 24.58 | 22.23 | 107 | 26 | 51.33 | 151 | 92 | 9 |

| 1450 | 37.32 | -2.62 | GBIF | 12.70 | 72.99 | 22.70 | 20.00 | 146 | 5 | 83.47 | 179 | 23 | 8 |
| --- | --- | --- | --- | --- | --- | --- | --- | --- | --- | --- | --- | --- | --- |
| 1451 | 37.38 | 1.85 | GBIF | 12.43 | 82.28 | 26.03 | 24.25 | 100 | 2 | 101.37 | 156 | 11 | 2 |
| 1452 | 35.09 | -1.48 | GBIF | 12.84 | 81.28 | 20.48 | 18.97 | 156 | 28 | 43.86 | 368 | 130 | 9 |
| 1453 | 38.70 | -3.33 | GBIF | 10.69 | 70.34 | 25.32 | 22.37 | 150 | 19 | 67.34 | 289 | 73 | 5 |
| 1454 | 38.94 | -3.57 | GBIF | 10.11 | 69.24 | 24.90 | 22.52 | 117 | 22 | 58.33 | 223 | 86 | 4 |
| 1455 | 38.96 | -3.48 | GBIF | 10.23 | 69.56 | 24.98 | 22.63 | 123 | 21 | 60.04 | 233 | 88 | 2 |
| 1456 | 37.41 | 0.43 | GBIF | 14.25 | 84.82 | 22.77 | 22.63 | 165 | 9 | 89.68 | 270 | 263 | 6 |
| 1457 | 36.91 | -0.27 | GBIF | 13.59 | 78.11 | 17.32 | 15.85 | 141 | 25 | 53.71 | 319 | 104 | 15 |
| 1458 | 36.83 | -0.38 | GBIF | 11.98 | 79.36 | 15.35 | 13.50 | 239 | 54 | 56.15 | 540 | 204 | 10 |
| 1459 | 37.00 | -0.31 | GBIF | 13.12 | 77.16 | 17.73 | 16.12 | 158 | 26 | 60.14 | 365 | 98 | 16 |
| 1460 | 36.95 | -0.32 | GBIF | 12.94 | 77.03 | 17.60 | 15.95 | 153 | 28 | 57.13 | 348 | 103 | 9 |
| 1461 | 35.16 | -1.15 | GBIF | 13.01 | 83.39 | 19.62 | 18.07 | 184 | 42 | 39.55 | 305 | 183 | 4 |
| 1462 | 38.76 | -3.14 | GBIF | 10.83 | 70.81 | 26.07 | 23.12 | 169 | 23 | 68.15 | 309 | 80 | 1 |
| 1463 | 35.01 | -1.56 | GBIF | 12.56 | 81.02 | 20.97 | 19.57 | 162 | 27 | 45.00 | 293 | 126 | 7 |
| 1464 | 38.96 | -3.33 | GBIF | 10.54 | 69.81 | 25.27 | 22.87 | 135 | 21 | 62.66 | 260 | 96 | 2 |
| 1465 | 38.08 | 0.18 | GBIF | 10.95 | 77.11 | 23.53 | 22.10 | 184 | 2 | 115.12 | 244 | 9 | 3 |
| 1466 | 35.63 | -1.21 | GBIF | 13.63 | 82.08 | 18.53 | 16.72 | 147 | 23 | 53.63 | 332 | 92 | 11 |
| 1467 | 37.65 | 3.72 | GBIF | 10.93 | 73.38 | 25.83 | 23.37 | 71 | 2 | 85.88 | 73 | 10 | 1 |
| 1468 | 35.00 | -1.26 | GBIF | 12.32 | 82.17 | 20.03 | 18.52 | 188 | 41 | 38.55 | 312 | 185 | 11 |
| 1469 | 35.05 | -1.27 | GBIF | 12.76 | 81.78 | 20.33 | 18.83 | 170 | 36 | 38.88 | 296 | 169 | 7 |
| 1470 | 39.44 | -4.24 | GBIF | 7.88 | 64.62 | 23.67 | 25.42 | 223 | 22 | 59.01 | 123 | 222 | 7 |
| 1471 | 36.08 | -1.83 | GBIF | 11.60 | 73.89 | 25.83 | 22.78 | 87 | 4 | 76.60 | 199 | 14 | 13 |
| 1472 | 37.76 | 1.01 | GBIF | 12.32 | 82.66 | 24.72 | 23.05 | 121 | 2 | 110.51 | 187 | 7 | 2 |
| 1473 | 37.62 | 1.14 | GBIF | 12.38 | 82.56 | 24.92 | 23.28 | 112 | 2 | 106.48 | 181 | 7 | 4 |
| 1474 | 37.79 | 1.14 | GBIF | 12.24 | 82.16 | 25.08 | 23.43 | 115 | 1 | 111.88 | 180 | 5 | 3 |
| 1475 | 37.68 | 1.10 | GBIF | 12.19 | 81.82 | 24.80 | 23.30 | 115 | 1 | 109.58 | 184 | 6 | 6 |
| 1476 | 37.78 | 1.10 | GBIF | 12.03 | 81.86 | 24.95 | 23.23 | 115 | 1 | 112.13 | 183 | 5 | 2 |
| 1477 | 38.05 | 0.21 | GBIF | 11.01 | 77.52 | 23.07 | 21.33 | 207 | 3 | 113.46 | 272 | 9 | 7 |
| 1478 | 37.00 | 0.34 | GBIF | 14.63 | 81.70 | 18.78 | 17.88 | 153 | 17 | 63.41 | 230 | 142 | 5 |
| 1479 | 37.57 | 0.72 | GBIF | 12.34 | 82.83 | 21.63 | 21.12 | 188 | 4 | 100.93 | 293 | 15 | 4 |
| 1480 | 36.18 | -2.18 | GBIF | 12.49 | 75.25 | 26.08 | 23.00 | 80 | 0 | 87.01 | 154 | 2 | 5 |
| 1481 | 37.21 | -2.69 | GBIF | 13.23 | 73.52 | 22.68 | 19.97 | 147 | 5 | 82.00 | 175 | 24 | 4 |
| 1482 | 35.00 | -1.34 | GBIF | 12.56 | 81.55 | 20.33 | 18.82 | 177 | 35 | 40.84 | 301 | 161 | 4 |
| 1483 | 37.10 | 0.15 | GBIF | 14.33 | 80.52 | 17.32 | 16.53 | 132 | 18 | 56.24 | 205 | 155 | 12 |
| 1484 | 38.58 | -3.28 | GBIF | 10.52 | 70.11 | 24.88 | 21.95 | 157 | 16 | 71.50 | 297 | 64 | 1 |
| 1485 | 37.93 | 1.78 | GBIF | 12.10 | 80.13 | 26.87 | 25.10 | 99 | 0 | 115.85 | 149 | 3 | 3 |
| 1486 | 37.54 | -2.75 | GBIF | 12.71 | 71.80 | 22.17 | 19.42 | 171 | 9 | 83.30 | 204 | 37 | 8 |
| 1487 | 36.83 | 2.16 | GBIF | 10.11 | 77.76 | 14.47 | 13.95 | 148 | 27 | 46.03 | 257 | 213 | 5 |
| 1488 | 38.33 | -3.42 | GBIF | 9.52 | 65.69 | 18.88 | 16.08 | 213 | 18 | 76.88 | 308 | 63 | 17 |
| 1489 | 37.83 | -2.84 | GBIF | 12.04 | 69.20 | 23.20 | 20.30 | 157 | 4 | 94.01 | 161 | 18 | 6 |
| 1490 | 34.88 | -1.42 | GBIF | 12.42 | 81.69 | 20.30 | 18.85 | 181 | 36 | 39.88 | 314 | 164 | 5 |
| 1491 | 38.76 | -3.33 | GBIF | 10.72 | 70.97 | 24.97 | 22.55 | 138 | 22 | 64.53 | 273 | 76 | 2 |
| 1492 | 37.20 | -2.70 | GBIF | 13.22 | 73.47 | 22.67 | 19.97 | 152 | 5 | 82.30 | 178 | 25 | 4 |
| 1493 | 39.11 | -3.10 | GBIF | 10.51 | 71.00 | 26.05 | 23.80 | 134 | 22 | 61.31 | 245 | 101 | 3 |
| 1494 | 38.08 | -2.96 | GBIF | 10.80 | 68.79 | 23.50 | 20.32 | 149 | 4 | 94.71 | 149 | 17 | 1 |
| 1495 | 37.39 | -2.67 | GBIF | 13.10 | 73.60 | 22.67 | 19.95 | 154 | 6 | 83.53 | 184 | 27 | 6 |
| 1496 | 37.91 | -2.80 | GBIF | 11.18 | 67.78 | 22.67 | 19.43 | 166 | 6 | 91.87 | 180 | 26 | 6 |
| 1497 | 35.31 | -1.16 | GBIF | 12.48 | 82.62 | 18.90 | 17.23 | 187 | 34 | 45.29 | 408 | 153 | 4 |
| 1498 | 35.36 | -1.56 | GBIF | 12.14 | 80.41 | 19.03 | 17.30 | 182 | 25 | 53.19 | 402 | 109 | 4 |
| 1499 | 37.42 | 0.49 | GBIF | 14.17 | 85.34 | 23.02 | 22.83 | 148 | 8 | 89.44 | 242 | 235 | 8 |

| 1500 | 35.29 | -1.61 | GBIF | 12.43 | 80.74 | 20.00 | 18.05 | 160 | 22 | 53.31 | 372 | 97 | 14 |
| --- | --- | --- | --- | --- | --- | --- | --- | --- | --- | --- | --- | --- | --- |
| 1501 | 34.89 | -1.36 | GBIF | 12.03 | 81.31 | 20.15 | 18.67 | 192 | 39 | 40.25 | 317 | 173 | 11 |
| 1502 | 35.04 | -1.45 | GBIF | 12.70 | 81.41 | 20.67 | 19.18 | 161 | 29 | 42.68 | 292 | 138 | 4 |
| 1503 | 35.07 | -1.43 | GBIF | 12.75 | 81.73 | 20.68 | 19.12 | 159 | 29 | 42.30 | 292 | 139 | 2 |
| 1504 | 36.83 | -0.74 | GBIF | 11.98 | 76.82 | 15.38 | 12.93 | 395 | 61 | 73.81 | 598 | 239 | 15 |
| 1505 | 35.08 | -1.56 | GBIF | 12.70 | 81.41 | 20.58 | 19.13 | 153 | 26 | 44.50 | 291 | 122 | 8 |
| 1506 | 34.93 | -1.38 | GBIF | 12.51 | 81.75 | 20.27 | 18.72 | 179 | 36 | 40.03 | 309 | 165 | 11 |
| 1507 | 35.21 | 1.79 | GBIF | 12.75 | 85.00 | 21.95 | 22.67 | 103 | 14 | 53.54 | 160 | 242 | 5 |
| 1508 | 36.89 | 0.06 | GBIF | 14.77 | 78.55 | 17.65 | 16.53 | 131 | 24 | 47.95 | 277 | 169 | 3 |
| 1509 | 35.21 | -1.07 | GBIF | 12.73 | 81.57 | 19.47 | 17.88 | 169 | 39 | 39.92 | 288 | 171 | 14 |
| 1510 | 35.25 | -1.07 | GBIF | 12.55 | 81.49 | 19.28 | 17.70 | 162 | 37 | 40.67 | 288 | 162 | 13 |
| 1511 | 37.53 | 0.65 | GBIF | 12.89 | 83.17 | 22.95 | 22.52 | 136 | 4 | 100.01 | 200 | 15 | 10 |
| 1512 | 34.97 | -1.47 | GBIF | 12.49 | 81.64 | 20.30 | 18.87 | 175 | 32 | 41.96 | 308 | 149 | 18 |
| 1513 | 34.90 | -1.33 | GBIF | 11.56 | 80.83 | 18.33 | 16.82 | 220 | 45 | 40.75 | 343 | 195 | 6 |
| 1514 | 34.87 | -1.23 | GBIF | 11.44 | 81.73 | 18.83 | 17.33 | 225 | 50 | 38.33 | 343 | 222 | 5 |
| 1515 | 36.75 | -0.45 | GBIF | 11.26 | 79.28 | 12.90 | 12.13 | 226 | 45 | 53.70 | 430 | 216 | 2 |
| 1516 | 38.50 | -3.31 | GBIF | 9.97 | 68.79 | 23.53 | 21.02 | 141 | 11 | 76.60 | 283 | 44 | 10 |
| 1517 | 37.26 | -2.69 | GBIF | 13.13 | 73.32 | 22.63 | 19.90 | 145 | 5 | 82.15 | 175 | 25 | 4 |
| 1518 | 36.96 | -0.35 | GBIF | 12.79 | 76.60 | 17.92 | 16.18 | 160 | 28 | 59.70 | 365 | 101 | 17 |
| 1519 | 38.63 | -3.00 | GBIF | 10.48 | 69.83 | 25.68 | 22.67 | 190 | 24 | 70.23 | 340 | 89 | 3 |
| 1520 | 37.27 | -2.62 | GBIF | 12.57 | 73.11 | 22.65 | 19.92 | 145 | 3 | 84.66 | 177 | 20 | 8 |
| 1521 | 35.02 | -1.40 | GBIF | 12.61 | 81.87 | 20.28 | 18.77 | 167 | 32 | 40.66 | 301 | 152 | 12 |
| 1522 | 37.51 | 0.45 | GBIF | 12.46 | 82.51 | 20.87 | 20.50 | 234 | 8 | 97.24 | 334 | 26 | 10 |
| 1523 | 37.95 | 0.69 | GBIF | 13.12 | 84.62 | 24.27 | 23.47 | 142 | 1 | 117.81 | 185 | 5 | 2 |
| 1524 | 38.86 | -3.08 | GBIF | 10.48 | 69.89 | 26.52 | 23.58 | 157 | 25 | 64.47 | 291 | 86 | 6 |
| 1525 | 38.91 | -3.29 | GBIF | 10.61 | 70.25 | 25.78 | 22.85 | 142 | 22 | 64.06 | 272 | 97 | 5 |
| 1526 | 38.96 | -3.26 | GBIF | 10.38 | 70.16 | 25.27 | 22.92 | 137 | 22 | 62.78 | 260 | 97 | 3 |
| 1527 | 38.89 | -3.36 | GBIF | 10.73 | 70.56 | 25.17 | 22.77 | 134 | 22 | 62.75 | 261 | 93 | 6 |
| 1528 | 37.35 | -2.62 | GBIF | 13.00 | 73.45 | 22.65 | 19.97 | 153 | 5 | 84.04 | 184 | 25 | 8 |
| 1529 | 37.67 | -0.89 | GBIF | 11.68 | 75.87 | 22.25 | 20.20 | 209 | 2 | 107.87 | 292 | 11 | 15 |
| 1530 | 35.02 | -1.57 | GBIF | 12.63 | 80.98 | 20.78 | 19.42 | 161 | 27 | 44.56 | 294 | 127 | 6 |
| 1531 | 38.31 | -2.35 | GBIF | 9.56 | 67.31 | 24.78 | 22.18 | 165 | 3 | 104.14 | 234 | 15 | 0 |
| 1532 | 37.68 | -2.41 | GBIF | 11.92 | 69.69 | 22.43 | 19.57 | 168 | 1 | 99.36 | 258 | 8 | 2 |
| 1533 | 37.41 | 0.26 | GBIF | 13.57 | 83.74 | 19.32 | 18.65 | 251 | 11 | 94.97 | 356 | 41 | 6 |
| 1534 | 37.00 | -0.21 | GBIF | 14.12 | 78.86 | 17.17 | 15.78 | 143 | 23 | 58.36 | 327 | 89 | 13 |
| 1535 | 35.28 | -1.02 | GBIF | 12.81 | 81.58 | 19.32 | 17.72 | 165 | 38 | 40.58 | 285 | 164 | 15 |
| 1536 | 37.52 | 0.55 | GBIF | 13.31 | 83.18 | 23.47 | 23.02 | 129 | 4 | 98.38 | 182 | 16 | 12 |
| 1537 | 37.45 | 0.57 | GBIF | 13.67 | 84.36 | 23.25 | 22.92 | 134 | 6 | 93.49 | 204 | 20 | 11 |
| 1538 | 38.06 | -2.98 | GBIF | 10.59 | 68.78 | 23.35 | 20.33 | 146 | 5 | 94.00 | 145 | 19 | 1 |
| 1539 | 35.09 | -1.34 | GBIF | 12.95 | 81.96 | 20.22 | 18.67 | 170 | 32 | 42.19 | 295 | 151 | 3 |
| 1540 | 38.32 | -2.22 | GBIF | 10.04 | 67.85 | 24.68 | 22.15 | 175 | 2 | 112.71 | 230 | 9 | 4 |
| 1541 | 37.60 | 0.08 | GBIF | 11.85 | 79.00 | 17.90 | 16.32 | 390 | 19 | 95.66 | 554 | 65 | 19 |
| 1542 | 38.18 | -2.73 | GBIF | 10.44 | 67.80 | 23.88 | 20.95 | 151 | 3 | 101.02 | 254 | 16 | 1 |
| 1543 | 38.17 | -3.00 | GBIF | 9.92 | 67.92 | 22.73 | 19.75 | 159 | 6 | 90.63 | 177 | 25 | 5 |
| 1544 | 34.83 | -1.23 | GBIF | 11.79 | 81.89 | 19.87 | 18.40 | 214 | 50 | 37.33 | 332 | 220 | 9 |
| 1545 | 38.68 | -3.32 | GBIF | 10.67 | 70.18 | 25.27 | 22.33 | 144 | 18 | 67.02 | 283 | 70 | 1 |
| 1546 | 36.98 | 0.37 | GBIF | 14.72 | 82.22 | 19.02 | 18.10 | 159 | 18 | 64.70 | 240 | 139 | 5 |
| 1547 | 35.01 | -1.38 | GBIF | 12.71 | 81.99 | 20.48 | 18.98 | 168 | 33 | 40.58 | 387 | 154 | 4 |
| 1548 | 36.82 | 0.18 | GBIF | 15.24 | 78.97 | 17.97 | 16.93 | 135 | 19 | 51.89 | 276 | 167 | 10 |
| 1549 | 39.90 | -3.20 | GBIF | 7.76 | 66.88 | 24.98 | 26.10 | 207 | 13 | 66.85 | 195 | 189 | 16 |

| 1550 | 37.58 | 1.08 | GBIF | 12.59 | 83.39 | 24.70 | 23.18 | 115 | 2 | 104.84 | 188 | 10 | 7 |
| --- | --- | --- | --- | --- | --- | --- | --- | --- | --- | --- | --- | --- | --- |
| 1551 | 37.21 | -2.60 | GBIF | 12.88 | 73.62 | 22.73 | 20.07 | 137 | 3 | 83.39 | 175 | 18 | 3 |
| 1552 | 34.95 | -1.29 | GBIF | 11.84 | 81.67 | 18.48 | 17.00 | 212 | 45 | 39.38 | 337 | 199 | 4 |
| 1553 | 37.15 | -0.30 | GBIF | 11.49 | 76.61 | 15.78 | 13.92 | 231 | 29 | 67.85 | 402 | 97 | 14 |
| 1554 | 35.07 | -1.48 | GBIF | 12.77 | 81.37 | 20.50 | 18.98 | 161 | 28 | 44.08 | 376 | 131 | 9 |
| 1555 | 37.63 | -2.99 | GBIF | 11.94 | 70.25 | 21.07 | 18.50 | 240 | 19 | 78.34 | 306 | 70 | 11 |
| 1556 | 38.97 | -3.50 | GBIF | 10.20 | 69.86 | 24.97 | 22.62 | 121 | 23 | 57.69 | 228 | 92 | 2 |
| 1557 | 34.99 | -1.38 | GBIF | 12.68 | 81.83 | 20.43 | 18.92 | 172 | 34 | 40.99 | 300 | 156 | 5 |
| 1558 | 34.83 | -1.47 | GBIF | 12.55 | 82.03 | 21.15 | 19.78 | 174 | 34 | 40.17 | 306 | 156 | 8 |
| 1559 | 38.07 | -2.96 | GBIF | 10.73 | 68.75 | 23.13 | 20.12 | 156 | 5 | 93.91 | 159 | 19 | 2 |
| 1560 | 38.80 | -3.56 | GBIF | 10.43 | 70.02 | 24.63 | 22.23 | 122 | 18 | 61.74 | 167 | 81 | 4 |
| 1561 | 34.98 | -1.53 | GBIF | 12.48 | 81.59 | 20.77 | 19.37 | 167 | 29 | 43.57 | 299 | 136 | 11 |
| 1562 | 35.00 | -1.44 | GBIF | 12.58 | 81.71 | 20.47 | 18.98 | 163 | 32 | 40.65 | 300 | 148 | 4 |
| 1563 | 37.58 | 0.58 | GBIF | 13.31 | 83.18 | 23.60 | 23.05 | 121 | 3 | 102.56 | 170 | 13 | 14 |
| 1564 | 35.40 | -1.36 | GBIF | 13.02 | 81.87 | 19.73 | 17.75 | 154 | 23 | 51.83 | 357 | 99 | 3 |
| 1565 | 37.49 | 0.54 | GBIF | 13.65 | 83.74 | 23.35 | 23.12 | 136 | 5 | 95.29 | 205 | 20 | 7 |
| 1566 | 37.53 | 0.53 | GBIF | 13.38 | 82.56 | 23.33 | 22.95 | 137 | 4 | 97.48 | 193 | 16 | 7 |
| 1567 | 37.53 | 0.60 | GBIF | 13.15 | 83.23 | 23.08 | 22.65 | 139 | 4 | 97.94 | 205 | 16 | 16 |
| 1568 | 38.74 | -3.42 | GBIF | 10.51 | 69.59 | 25.32 | 22.37 | 135 | 19 | 64.91 | 268 | 85 | 3 |
| 1569 | 38.61 | -3.09 | GBIF | 10.74 | 70.21 | 25.80 | 22.83 | 197 | 23 | 71.19 | 347 | 83 | 10 |
| 1570 | 35.59 | -1.37 | GBIF | 13.13 | 82.03 | 19.23 | 17.22 | 153 | 20 | 57.19 | 344 | 80 | 4 |
| 1571 | 34.93 | 1.58 | GBIF | 12.44 | 84.07 | 20.67 | 21.48 | 143 | 21 | 52.20 | 215 | 354 | 11 |
| 1572 | 38.75 | -3.31 | GBIF | 10.63 | 70.42 | 25.50 | 22.57 | 144 | 20 | 65.63 | 279 | 74 | 2 |
| 1573 | 37.49 | 0.44 | GBIF | 12.51 | 82.29 | 21.23 | 20.88 | 221 | 8 | 95.77 | 317 | 26 | 11 |
| 1574 | 34.98 | -1.37 | GBIF | 12.71 | 81.99 | 20.43 | 18.92 | 170 | 34 | 40.30 | 300 | 156 | 4 |
| 1575 | 37.44 | 0.50 | GBIF | 14.07 | 84.23 | 23.05 | 22.83 | 149 | 7 | 90.95 | 236 | 235 | 6 |
| 1576 | 36.80 | 0.04 | GBIF | 14.86 | 78.62 | 17.48 | 16.32 | 114 | 28 | 41.08 | 248 | 174 | 3 |
| 1577 | 35.39 | -1.53 | GBIF | 12.07 | 80.44 | 18.20 | 16.50 | 190 | 27 | 54.30 | 414 | 109 | 11 |
| 1578 | 36.81 | -0.26 | GBIF | 13.65 | 80.29 | 15.90 | 14.27 | 163 | 38 | 48.91 | 360 | 151 | 15 |
| 1579 | 34.97 | 1.59 | GBIF | 12.56 | 83.72 | 20.90 | 21.70 | 137 | 20 | 52.04 | 207 | 342 | 9 |
| 1580 | 35.53 | -0.71 | GBIF | 11.04 | 81.19 | 15.62 | 15.52 | 201 | 66 | 36.47 | 381 | 304 | 3 |
| 1581 | 35.10 | -1.15 | GBIF | 12.74 | 82.74 | 19.85 | 18.32 | 183 | 43 | 38.01 | 302 | 192 | 7 |
| 1582 | 38.16 | -2.73 | GBIF | 10.47 | 67.58 | 23.83 | 20.90 | 150 | 2 | 101.83 | 249 | 15 | 10 |
| 1583 | 36.74 | -0.44 | GBIF | 10.88 | 78.86 | 11.08 | 10.43 | 198 | 39 | 39.25 | 389 | 306 | 1 |
| 1584 | 37.60 | 0.57 | GBIF | 13.47 | 83.13 | 23.52 | 23.00 | 126 | 3 | 103.62 | 177 | 13 | 10 |
| 1585 | 35.08 | -1.54 | GBIF | 12.70 | 81.41 | 20.52 | 18.87 | 154 | 28 | 44.21 | 292 | 124 | 3 |
| 1586 | 37.38 | -0.54 | GBIF | 11.56 | 76.55 | 20.58 | 18.42 | 282 | 19 | 91.32 | 409 | 69 | 16 |
| 1587 | 37.05 | -0.29 | GBIF | 13.09 | 77.93 | 17.48 | 15.83 | 176 | 25 | 64.11 | 403 | 95 | 14 |
| 1588 | 38.72 | -3.53 | GBIF | 10.46 | 69.26 | 24.43 | 22.05 | 127 | 17 | 63.52 | 173 | 82 | 17 |
| 1589 | 38.68 | -3.40 | GBIF | 10.77 | 70.42 | 24.73 | 22.25 | 136 | 18 | 65.19 | 275 | 73 | 3 |
| 1590 | 35.50 | -1.21 | GBIF | 13.42 | 82.31 | 18.23 | 16.57 | 159 | 25 | 50.60 | 358 | 108 | 4 |
| 1591 | 38.64 | -3.49 | GBIF | 10.06 | 68.89 | 24.00 | 21.58 | 128 | 14 | 67.61 | 255 | 59 | 9 |
| 1592 | 36.93 | 0.26 | GBIF | 14.56 | 80.43 | 18.67 | 17.73 | 165 | 20 | 60.83 | 334 | 157 | 18 |
| 1593 | 38.62 | -3.21 | GBIF | 10.17 | 69.63 | 25.18 | 22.25 | 164 | 18 | 70.19 | 307 | 71 | 5 |
| 1594 | 34.90 | -1.38 | GBIF | 12.26 | 81.18 | 20.25 | 18.72 | 183 | 37 | 39.58 | 315 | 169 | 6 |
| 1595 | 40.06 | -1.82 | GBIF | 8.65 | 67.05 | 27.40 | 28.93 | 88 | 10 | 49.35 | 138 | 108 | 14 |
| 1596 | 34.98 | -1.44 | GBIF | 12.54 | 81.97 | 20.30 | 18.85 | 170 | 32 | 41.13 | 306 | 150 | 5 |
| 1597 | 37.87 | -3.00 | GBIF | 12.28 | 69.79 | 23.68 | 20.67 | 150 | 4 | 93.81 | 151 | 15 | 6 |
| 1598 | 37.62 | 0.49 | GBIF | 13.57 | 81.73 | 23.33 | 22.97 | 148 | 3 | 101.22 | 201 | 13 | 10 |
| 1599 | 34.95 | -1.52 | GBIF | 12.55 | 81.49 | 20.82 | 19.25 | 171 | 31 | 42.68 | 302 | 142 | 14 |

| 1600 | 37.54 | 0.21 | GBIF | 13.46 | 82.06 | 19.42 | 18.58 | 317 | 7 | 101.96 | 400 | 28 | 11 |
| --- | --- | --- | --- | --- | --- | --- | --- | --- | --- | --- | --- | --- | --- |
| 1601 | 37.02 | 0.33 | GBIF | 14.57 | 81.84 | 18.85 | 17.95 | 152 | 18 | 63.16 | 230 | 141 | 5 |
| 1602 | 37.69 | 0.25 | GBIF | 12.88 | 82.06 | 21.63 | 20.95 | 283 | 4 | 104.24 | 344 | 14 | 8 |
| 1603 | 35.07 | -1.38 | GBIF | 12.78 | 81.94 | 20.23 | 18.67 | 167 | 32 | 41.83 | 385 | 148 | 3 |
| 1604 | 37.63 | 0.27 | GBIF | 13.23 | 81.64 | 21.78 | 21.23 | 257 | 5 | 101.86 | 324 | 15 | 7 |
| 1605 | 36.81 | 0.43 | GBIF | 14.46 | 81.23 | 19.12 | 18.17 | 160 | 20 | 60.26 | 336 | 166 | 3 |
| 1606 | 34.81 | -1.32 | GBIF | 11.65 | 80.34 | 19.48 | 18.02 | 222 | 46 | 38.97 | 344 | 208 | 12 |
| 1607 | 37.52 | 0.47 | GBIF | 12.53 | 81.92 | 22.18 | 21.93 | 185 | 6 | 95.96 | 272 | 289 | 10 |
| 1608 | 37.90 | -3.43 | GBIF | 11.25 | 68.60 | 22.80 | 20.00 | 165 | 10 | 81.21 | 195 | 40 | 3 |
| 1609 | 37.93 | -3.49 | GBIF | 11.07 | 68.36 | 22.65 | 19.65 | 153 | 10 | 79.93 | 197 | 37 | 2 |
| 1610 | 36.88 | -0.16 | GBIF | 14.00 | 78.21 | 17.25 | 16.00 | 122 | 27 | 47.92 | 279 | 225 | 15 |
| 1611 | 35.70 | -1.38 | GBIF | 12.59 | 80.20 | 18.05 | 16.27 | 170 | 25 | 58.11 | 356 | 92 | 3 |
| 1612 | 35.19 | -1.65 | GBIF | 12.68 | 81.77 | 20.72 | 18.95 | 149 | 23 | 50.29 | 358 | 99 | 6 |
| 1613 | 37.60 | 0.25 | GBIF | 13.24 | 82.25 | 21.07 | 20.47 | 276 | 5 | 102.57 | 341 | 17 | 14 |
| 1614 | 38.55 | -2.11 | GBIF | 9.41 | 67.69 | 25.42 | 22.92 | 174 | 5 | 105.98 | 225 | 16 | 0 |
| 1615 | 38.49 | -2.09 | GBIF | 9.43 | 67.38 | 25.18 | 22.65 | 174 | 4 | 107.74 | 226 | 14 | 0 |
| 1616 | 38.53 | -2.03 | GBIF | 9.31 | 67.45 | 25.27 | 22.77 | 176 | 4 | 109.53 | 220 | 13 | 0 |
| 1617 | 38.42 | -3.37 | GBIF | 9.29 | 66.37 | 21.58 | 18.57 | 194 | 21 | 73.31 | 236 | 78 | 9 |
| 1618 | 34.81 | -1.53 | GBIF | 12.37 | 81.90 | 21.33 | 19.92 | 177 | 32 | 42.12 | 304 | 149 | 14 |
| 1619 | 37.43 | 0.41 | GBIF | 14.12 | 84.53 | 22.72 | 22.58 | 169 | 8 | 89.61 | 266 | 272 | 10 |
| 1620 | 37.21 | 0.40 | GBIF | 12.16 | 82.15 | 17.57 | 17.20 | 155 | 20 | 72.14 | 242 | 106 | 12 |
| 1621 | 38.63 | -2.65 | GBIF | 9.78 | 68.41 | 25.40 | 22.93 | 172 | 14 | 83.95 | 296 | 51 | 1 |
| 1622 | 38.51 | -2.06 | GBIF | 9.29 | 67.33 | 25.17 | 22.67 | 175 | 4 | 109.50 | 218 | 13 | 0 |
| 1623 | 35.18 | -1.35 | GBIF | 12.97 | 81.55 | 20.38 | 18.78 | 158 | 29 | 43.92 | 369 | 134 | 3 |
| 1624 | 36.88 | -1.32 | GBIF | 12.18 | 74.74 | 20.88 | 18.52 | 163 | 13 | 74.48 | 281 | 55 | 42 |
| 1625 | 38.49 | -2.02 | GBIF | 9.44 | 67.44 | 25.08 | 22.58 | 177 | 3 | 111.26 | 218 | 10 | 3 |
| 1626 | 34.96 | -1.26 | GBIF | 11.77 | 81.77 | 18.75 | 17.25 | 219 | 45 | 39.98 | 336 | 203 | 7 |
| 1627 | 36.89 | 0.25 | GBIF | 14.73 | 79.64 | 18.23 | 17.27 | 143 | 18 | 55.67 | 293 | 163 | 2 |
| 1628 | 36.82 | 0.22 | GBIF | 15.08 | 78.97 | 17.92 | 16.90 | 128 | 17 | 51.70 | 269 | 171 | 3 |
| 1629 | 37.57 | 0.37 | GBIF | 13.11 | 80.92 | 23.00 | 22.85 | 173 | 4 | 96.84 | 246 | 276 | 14 |
| 1630 | 35.15 | -1.25 | GBIF | 13.02 | 82.38 | 20.02 | 18.42 | 181 | 35 | 41.86 | 404 | 161 | 3 |
| 1631 | 35.31 | -1.09 | GBIF | 11.99 | 81.58 | 17.43 | 15.85 | 203 | 46 | 42.71 | 425 | 183 | 5 |
| 1632 | 38.31 | -3.50 | GBIF | 10.42 | 67.64 | 22.50 | 19.48 | 147 | 13 | 74.08 | 182 | 50 | 21 |
| 1633 | 36.86 | 0.34 | GBIF | 14.96 | 80.86 | 18.38 | 17.43 | 135 | 16 | 56.32 | 277 | 164 | 4 |
| 1634 | 38.83 | -3.24 | GBIF | 10.48 | 70.36 | 25.17 | 22.75 | 146 | 22 | 65.05 | 281 | 97 | 3 |
| 1635 | 39.48 | -1.57 | GBIF | 9.50 | 69.34 | 27.33 | 24.90 | 109 | 5 | 76.78 | 156 | 57 | 10 |
| 1636 | 40.09 | -1.73 | GBIF | 8.11 | 67.01 | 27.28 | 28.75 | 85 | 10 | 50.43 | 124 | 98 | 13 |
| 1637 | 34.95 | -1.54 | GBIF | 12.45 | 81.37 | 20.87 | 19.28 | 172 | 29 | 44.02 | 302 | 136 | 13 |
| 1638 | 35.27 | -1.14 | GBIF | 12.35 | 82.33 | 18.47 | 16.87 | 196 | 38 | 44.28 | 311 | 163 | 8 |
| 1639 | 37.04 | -0.35 | GBIF | 12.73 | 76.71 | 18.13 | 16.33 | 184 | 33 | 65.18 | 423 | 109 | 15 |
| 1640 | 35.15 | -1.40 | GBIF | 12.89 | 82.11 | 20.53 | 18.97 | 161 | 28 | 44.87 | 371 | 132 | 7 |
| 1641 | 34.94 | -1.23 | GBIF | 11.39 | 81.37 | 18.77 | 17.23 | 212 | 47 | 37.68 | 336 | 213 | 4 |
| 1642 | 37.37 | -0.05 | GBIF | 12.49 | 77.11 | 7.95 | 7.02 | 230 | 46 | 53.25 | 435 | 209 | 1 |
| 1643 | 35.03 | -1.28 | GBIF | 12.68 | 81.77 | 20.28 | 18.80 | 174 | 37 | 39.13 | 298 | 169 | 11 |
| 1644 | 37.00 | 0.17 | GBIF | 14.85 | 79.84 | 17.82 | 16.90 | 129 | 17 | 54.46 | 276 | 167 | 6 |
| 1645 | 35.21 | -1.47 | GBIF | 12.97 | 81.55 | 20.33 | 18.68 | 153 | 26 | 47.32 | 359 | 113 | 10 |
| 1646 | 35.24 | -1.40 | GBIF | 12.88 | 81.54 | 20.65 | 18.70 | 151 | 26 | 46.11 | 357 | 118 | 4 |
| 1647 | 38.07 | -3.40 | GBIF | 11.12 | 68.20 | 22.13 | 19.17 | 119 | 7 | 80.99 | 168 | 28 | 6 |
| 1648 | 38.17 | -3.57 | GBIF | 11.25 | 69.02 | 23.12 | 20.13 | 117 | 7 | 77.93 | 175 | 28 | 4 |
| 1649 | 37.21 | 2.91 | GBIF | 12.23 | 79.44 | 28.02 | 26.27 | 58 | 0 | 95.27 | 49 | 9 | 0 |

| 1650 | 35.17 | -1.19 | GBIF | 13.03 | 83.01 | 19.60 | 18.03 | 187 | 39 | 40.94 | 413 | 178 | 11 |
| --- | --- | --- | --- | --- | --- | --- | --- | --- | --- | --- | --- | --- | --- |
| 1651 | 35.16 | -1.52 | GBIF | 12.80 | 81.53 | 20.22 | 18.70 | 157 | 26 | 46.86 | 367 | 117 | 2 |
| 1652 | 38.08 | -3.42 | GBIF | 11.08 | 68.00 | 22.27 | 19.30 | 118 | 8 | 80.29 | 165 | 28 | 6 |
| 1653 | 38.08 | -3.52 | GBIF | 11.31 | 68.54 | 22.82 | 19.78 | 129 | 8 | 79.17 | 177 | 29 | 2 |
| 1654 | 38.14 | -3.47 | GBIF | 11.28 | 68.38 | 22.67 | 19.68 | 123 | 7 | 79.02 | 169 | 29 | 4 |
| 1655 | 38.02 | -3.52 | GBIF | 11.13 | 68.25 | 22.88 | 19.88 | 138 | 8 | 80.51 | 183 | 30 | 3 |
| 1656 | 38.11 | -3.43 | GBIF | 11.14 | 68.35 | 22.37 | 19.35 | 122 | 9 | 80.72 | 165 | 29 | 3 |
| 1657 | 34.93 | 1.60 | GBIF | 12.45 | 84.12 | 20.68 | 21.48 | 141 | 21 | 52.01 | 214 | 351 | 7 |
| 1658 | 37.47 | 0.54 | GBIF | 13.77 | 83.99 | 23.27 | 22.97 | 139 | 6 | 93.85 | 210 | 20 | 6 |
| 1659 | 38.17 | -3.52 | GBIF | 11.25 | 68.60 | 22.93 | 19.92 | 126 | 7 | 79.29 | 172 | 28 | 4 |
| 1660 | 38.10 | -3.47 | GBIF | 11.29 | 68.43 | 22.70 | 19.68 | 126 | 8 | 80.00 | 168 | 28 | 2 |
| 1661 | 38.16 | -2.87 | GBIF | 10.68 | 68.48 | 23.33 | 20.37 | 153 | 4 | 97.85 | 153 | 17 | 1 |
| 1662 | 39.17 | -3.19 | GBIF | 10.16 | 70.54 | 25.78 | 23.55 | 128 | 24 | 58.81 | 234 | 103 | 2 |
| 1663 | 39.11 | -3.01 | GBIF | 10.49 | 70.89 | 26.43 | 24.13 | 137 | 25 | 61.19 | 257 | 105 | 3 |
| 1664 | 38.92 | -3.27 | GBIF | 10.50 | 70.00 | 25.35 | 22.95 | 137 | 21 | 63.69 | 265 | 95 | 11 |
| 1665 | 35.21 | -1.05 | GBIF | 12.70 | 81.41 | 19.32 | 17.77 | 168 | 41 | 38.84 | 290 | 178 | 15 |
| 1666 | 37.20 | -2.67 | GBIF | 13.25 | 73.61 | 22.75 | 20.02 | 143 | 4 | 82.79 | 175 | 21 | 4 |
| 1667 | 35.31 | -1.07 | GBIF | 12.36 | 81.84 | 18.82 | 17.18 | 173 | 35 | 43.10 | 391 | 155 | 5 |
| 1668 | 35.17 | -1.60 | GBIF | 12.70 | 81.41 | 20.43 | 19.00 | 155 | 24 | 48.36 | 363 | 110 | 10 |
| 1669 | 37.53 | 0.35 | GBIF | 12.92 | 81.75 | 22.20 | 21.93 | 200 | 6 | 95.27 | 281 | 313 | 16 |
| 1670 | 36.86 | 0.09 | GBIF | 14.97 | 78.36 | 17.92 | 16.83 | 134 | 23 | 48.37 | 284 | 167 | 4 |
| 1671 | 38.71 | -3.40 | GBIF | 10.63 | 69.96 | 25.28 | 22.30 | 136 | 19 | 65.11 | 270 | 73 | 3 |
| 1672 | 36.78 | 0.04 | GBIF | 14.94 | 78.64 | 17.30 | 16.20 | 110 | 29 | 40.16 | 238 | 175 | 4 |
| 1673 | 36.63 | 0.13 | GBIF | 15.35 | 79.95 | 17.80 | 16.87 | 107 | 28 | 39.41 | 224 | 194 | 18 |
| 1674 | 38.68 | -3.04 | GBIF | 10.52 | 69.70 | 26.27 | 23.28 | 181 | 25 | 68.70 | 322 | 87 | 11 |
| 1675 | 38.64 | -3.20 | GBIF | 10.40 | 69.80 | 25.37 | 22.42 | 170 | 19 | 70.67 | 314 | 72 | 9 |
| 1676 | 37.70 | 0.60 | GBIF | 13.15 | 83.23 | 23.47 | 22.82 | 138 | 2 | 107.07 | 177 | 11 | 6 |
| 1677 | 36.87 | 0.09 | GBIF | 15.02 | 78.66 | 17.90 | 16.87 | 135 | 22 | 49.29 | 285 | 160 | 11 |
| 1678 | 38.03 | 0.35 | GBIF | 10.48 | 78.82 | 21.05 | 19.82 | 350 | 9 | 107.52 | 499 | 30 | 7 |
| 1679 | 37.41 | 0.45 | GBIF | 14.23 | 84.67 | 22.78 | 22.65 | 162 | 9 | 89.44 | 262 | 258 | 7 |
| 1680 | 36.69 | 0.59 | GBIF | 15.13 | 82.70 | 18.23 | 17.67 | 125 | 17 | 55.39 | 263 | 170 | 5 |
| 1681 | 36.97 | 0.03 | GBIF | 14.48 | 78.71 | 17.40 | 16.37 | 126 | 26 | 48.82 | 279 | 181 | 11 |
| 1682 | 36.90 | 0.47 | GBIF | 14.92 | 82.41 | 19.67 | 18.68 | 190 | 21 | 69.93 | 379 | 139 | 7 |
| 1683 | 36.86 | 0.45 | GBIF | 14.72 | 81.81 | 19.25 | 18.32 | 174 | 20 | 65.18 | 346 | 153 | 6 |
| 1684 | 38.25 | -2.75 | GBIF | 10.12 | 67.90 | 23.95 | 21.47 | 154 | 5 | 95.80 | 272 | 26 | 8 |
| 1685 | 35.20 | -1.24 | GBIF | 12.96 | 82.54 | 19.57 | 18.00 | 170 | 33 | 42.95 | 297 | 149 | 4 |
| 1686 | 38.44 | -2.11 | GBIF | 9.48 | 67.26 | 25.08 | 22.57 | 174 | 3 | 110.28 | 228 | 12 | 0 |
| 1687 | 38.72 | -3.14 | GBIF | 10.86 | 70.51 | 26.00 | 23.02 | 174 | 22 | 68.60 | 317 | 81 | 1 |
| 1688 | 38.53 | -2.13 | GBIF | 9.44 | 67.93 | 25.35 | 22.82 | 173 | 5 | 106.08 | 228 | 16 | 0 |
| 1689 | 38.41 | -2.13 | GBIF | 9.53 | 67.14 | 24.78 | 22.30 | 179 | 3 | 111.33 | 229 | 12 | 1 |
| 1690 | 37.72 | 0.68 | GBIF | 12.72 | 83.66 | 23.62 | 22.93 | 132 | 2 | 110.83 | 174 | 9 | 5 |
| 1691 | 36.92 | 0.33 | GBIF | 14.60 | 81.11 | 19.07 | 18.17 | 175 | 20 | 63.87 | 263 | 153 | 4 |
| 1692 | 37.72 | 3.75 | GBIF | 10.88 | 73.04 | 25.57 | 23.15 | 74 | 2 | 86.32 | 75 | 10 | 1 |
| 1693 | 37.61 | -0.30 | GBIF | 10.19 | 77.80 | 19.37 | 17.23 | 379 | 22 | 93.41 | 557 | 99 | 12 |
| 1694 | 38.21 | -0.09 | GBIF | 11.53 | 75.88 | 25.57 | 23.57 | 146 | 0 | 124.72 | 167 | 0 | 5 |
| 1695 | 36.65 | -0.43 | GBIF | 10.47 | 77.53 | 9.47 | 8.75 | 204 | 45 | 34.46 | 397 | 362 | 5 |
| 1696 | 40.53 | -2.53 | GBIF | 6.68 | 66.17 | 27.00 | 27.85 | 336 | 7 | 100.62 | 173 | 171 | 18 |
| 1697 | 37.59 | -0.30 | GBIF | 10.02 | 77.05 | 18.58 | 16.42 | 410 | 31 | 88.56 | 603 | 136 | 8 |
| 1698 | 38.02 | -0.06 | GBIF | 11.42 | 75.61 | 23.98 | 21.97 | 173 | 1 | 118.19 | 223 | 3 | 10 |
| 1699 | 38.19 | -0.09 | GBIF | 11.45 | 75.33 | 25.35 | 23.37 | 149 | 0 | 123.56 | 171 | 0 | 4 |

| 1700 | 38.02 | 0.33 | GBIF | 10.20 | 78.46 | 21.15 | 19.78 | 339 | 9 | 106.85 | 487 | 30 | 8 |
| --- | --- | --- | --- | --- | --- | --- | --- | --- | --- | --- | --- | --- | --- |
| 1701 | 37.96 | -0.23 | GBIF | 10.73 | 73.97 | 24.32 | 22.10 | 160 | 0 | 119.08 | 213 | 2 | 10 |
| 1702 | 38.62 | -0.17 | GBIF | 10.57 | 74.47 | 25.83 | 23.95 | 148 | 0 | 131.38 | 140 | 0 | 2 |
| 1703 | 38.16 | -0.12 | GBIF | 11.13 | 74.66 | 25.03 | 22.97 | 156 | 0 | 125.18 | 177 | 0 | 13 |
| 1704 | 38.39 | -0.10 | GBIF | 10.41 | 74.88 | 25.88 | 23.98 | 140 | 0 | 130.58 | 150 | 0 | 7 |
| 1705 | 38.59 | -0.17 | GBIF | 10.69 | 74.77 | 26.03 | 24.15 | 145 | 0 | 131.70 | 138 | 0 | 2 |
| 1706 | 40.14 | -1.93 | GBIF | 8.07 | 67.29 | 27.30 | 28.62 | 86 | 10 | 48.56 | 137 | 118 | 1 |
| 1707 | 40.03 | -1.40 | GBIF | 8.58 | 66.03 | 27.63 | 25.65 | 86 | 9 | 67.01 | 116 | 55 | 7 |
| 1708 | 38.17 | -0.11 | GBIF | 11.21 | 75.22 | 25.13 | 23.12 | 154 | 0 | 126.18 | 178 | 0 | 8 |
| 1709 | 36.65 | 0.22 | GBIF | 15.65 | 79.85 | 18.03 | 17.12 | 111 | 23 | 44.36 | 228 | 185 | 5 |
| 1710 | 36.67 | 0.28 | GBIF | 15.73 | 80.27 | 18.37 | 17.47 | 116 | 21 | 46.41 | 241 | 180 | 6 |
| 1711 | 36.71 | 0.25 | GBIF | 15.71 | 79.74 | 18.25 | 17.38 | 119 | 21 | 47.31 | 244 | 178 | 4 |
| 1712 | 36.73 | 0.28 | GBIF | 15.55 | 79.74 | 18.30 | 17.30 | 127 | 20 | 49.67 | 263 | 176 | 4 |
| 1713 | 37.59 | 0.73 | GBIF | 12.56 | 83.72 | 23.13 | 22.55 | 129 | 3 | 103.92 | 186 | 12 | 12 |
| 1714 | 35.15 | -1.26 | GBIF | 13.02 | 81.92 | 20.12 | 18.52 | 169 | 34 | 41.05 | 390 | 155 | 3 |
| 1715 | 35.02 | -1.27 | GBIF | 12.47 | 82.02 | 20.18 | 18.72 | 179 | 38 | 38.88 | 302 | 177 | 8 |
| 1716 | 37.11 | -0.01 | GBIF | 14.18 | 79.19 | 16.58 | 15.73 | 152 | 28 | 56.05 | 243 | 153 | 25 |
| 1717 | 36.95 | 0.35 | GBIF | 14.60 | 81.56 | 19.15 | 18.22 | 169 | 19 | 64.49 | 340 | 146 | 5 |
| 1718 | 38.59 | -2.02 | GBIF | 9.47 | 68.11 | 25.50 | 23.03 | 175 | 4 | 108.32 | 219 | 14 | 1 |
| 1719 | 38.42 | -2.19 | GBIF | 9.57 | 67.37 | 25.10 | 22.57 | 172 | 4 | 107.86 | 230 | 14 | 2 |
| 1720 | 35.18 | -1.45 | GBIF | 12.92 | 81.29 | 20.43 | 18.83 | 153 | 26 | 46.10 | 360 | 119 | 3 |
| 1721 | 37.99 | 2.06 | GBIF | 10.80 | 75.52 | 26.10 | 24.32 | 105 | 1 | 113.01 | 158 | 3 | 3 |
| 1722 | 34.85 | -1.60 | GBIF | 12.23 | 80.96 | 21.27 | 19.73 | 168 | 29 | 43.13 | 301 | 137 | 5 |
| 1723 | 37.76 | -0.92 | GBIF | 11.48 | 75.05 | 22.38 | 20.25 | 215 | 1 | 113.01 | 287 | 6 | 10 |
| 1724 | 37.63 | 0.58 | GBIF | 13.48 | 83.23 | 23.57 | 22.98 | 125 | 3 | 104.24 | 161 | 13 | 9 |
| 1725 | 37.54 | 0.23 | GBIF | 13.31 | 82.15 | 19.93 | 19.20 | 297 | 7 | 101.77 | 369 | 26 | 11 |
| 1726 | 37.63 | 0.63 | GBIF | 13.34 | 83.91 | 23.57 | 22.98 | 123 | 3 | 105.07 | 165 | 13 | 10 |
| 1727 | 38.63 | -3.38 | GBIF | 10.77 | 70.37 | 25.07 | 22.13 | 135 | 17 | 66.79 | 273 | 66 | 6 |
| 1728 | 39.88 | -3.38 | GBIF | 7.17 | 65.75 | 25.12 | 26.18 | 226 | 12 | 71.20 | 157 | 186 | 13 |
| 1729 | 36.88 | -1.38 | GBIF | 12.07 | 73.13 | 20.63 | 18.20 | 151 | 12 | 74.74 | 262 | 50 | 14 |
| 1730 | 37.63 | -0.63 | GBIF | 10.77 | 75.88 | 21.33 | 19.45 | 234 | 5 | 101.65 | 353 | 20 | 22 |
| 1731 | 34.88 | -1.38 | GBIF | 12.04 | 81.36 | 20.35 | 18.82 | 187 | 38 | 39.93 | 315 | 170 | 7 |
| 1732 | 37.13 | 0.13 | GBIF | 14.51 | 80.16 | 17.33 | 16.57 | 138 | 20 | 58.20 | 217 | 146 | 9 |
| 1733 | 38.13 | -3.13 | GBIF | 10.77 | 69.07 | 23.50 | 20.60 | 156 | 5 | 91.99 | 153 | 20 | 9 |
| 1734 | 39.38 | -4.13 | GBIF | 8.83 | 65.92 | 24.73 | 26.63 | 180 | 24 | 56.40 | 115 | 189 | 8 |
| 1735 | 37.38 | 0.13 | GBIF | 13.34 | 80.37 | 14.30 | 13.97 | 177 | 25 | 64.48 | 284 | 138 | 20 |
| 1736 | 38.13 | -3.63 | GBIF | 11.26 | 69.07 | 23.08 | 20.10 | 115 | 6 | 78.70 | 186 | 25 | 3 |
| 1737 | 38.13 | -3.38 | GBIF | 11.11 | 68.57 | 21.75 | 18.82 | 140 | 9 | 82.67 | 202 | 31 | 11 |
| 1738 | 38.17 | -2.81 | GBIF | 10.52 | 67.85 | 23.63 | 20.67 | 149 | 4 | 98.12 | 262 | 18 | 4 |
| 1739 | 38.86 | -3.38 | GBIF | 10.69 | 70.81 | 25.13 | 22.70 | 132 | 22 | 62.02 | 262 | 93 | 4 |
| 1740 | 34.84 | -1.58 | GBIF | 12.24 | 80.54 | 21.38 | 19.90 | 169 | 29 | 42.88 | 299 | 139 | 7 |
| 1741 | 35.35 | -1.46 | GBIF | 12.82 | 81.12 | 20.57 | 18.55 | 151 | 21 | 51.86 | 352 | 96 | 4 |
| 1742 | 34.89 | -1.25 | GBIF | 11.38 | 81.25 | 18.55 | 17.05 | 227 | 50 | 38.91 | 347 | 217 | 11 |
| 1743 | 37.31 | -2.63 | GBIF | 12.63 | 73.03 | 22.65 | 19.95 | 148 | 4 | 84.37 | 178 | 22 | 8 |
| 1744 | 38.95 | -2.93 | GBIF | 10.23 | 69.61 | 25.98 | 23.60 | 150 | 25 | 65.10 | 273 | 86 | 2 |
| 1745 | 37.23 | -2.80 | GBIF | 12.18 | 71.20 | 21.22 | 18.63 | 190 | 12 | 79.60 | 206 | 51 | 4 |
| 1746 | 35.27 | -1.57 | GBIF | 12.46 | 80.90 | 20.08 | 18.18 | 165 | 24 | 51.53 | 377 | 107 | 9 |
| 1747 | 35.35 | -1.42 | GBIF | 12.98 | 81.66 | 20.23 | 18.23 | 156 | 23 | 50.88 | 359 | 103 | 3 |
| 1748 | 34.96 | -1.24 | GBIF | 11.57 | 81.46 | 18.87 | 17.32 | 207 | 46 | 37.64 | 332 | 208 | 4 |
| 1749 | 34.84 | -1.37 | GBIF | 11.61 | 80.06 | 18.92 | 17.42 | 221 | 44 | 40.28 | 349 | 195 | 9 |

| 1750 | 37.31 | -2.77 | GBIF | 12.91 | 72.11 | 21.93 | 19.30 | 184 | 10 | 81.28 | 198 | 42 | 4 |
| --- | --- | --- | --- | --- | --- | --- | --- | --- | --- | --- | --- | --- | --- |
| 1751 | 35.14 | -1.43 | GBIF | 12.89 | 81.59 | 20.58 | 19.00 | 159 | 28 | 45.34 | 369 | 127 | 2 |
| 1752 | 35.18 | -1.55 | GBIF | 12.75 | 81.21 | 20.22 | 18.70 | 160 | 25 | 48.73 | 369 | 113 | 3 |
| 1753 | 35.00 | -1.58 | GBIF | 12.57 | 81.60 | 20.90 | 19.33 | 167 | 27 | 45.65 | 294 | 125 | 5 |
| 1754 | 35.00 | -1.41 | GBIF | 12.60 | 81.82 | 20.27 | 18.75 | 168 | 33 | 40.40 | 305 | 154 | 14 |
| 1755 | 34.90 | 1.60 | GBIF | 12.57 | 83.78 | 20.68 | 21.52 | 142 | 21 | 52.03 | 221 | 358 | 6 |
| 1756 | 34.84 | -1.21 | GBIF | 11.64 | 81.41 | 19.38 | 17.92 | 222 | 51 | 37.54 | 338 | 226 | 5 |
| 1757 | 34.94 | -1.24 | GBIF | 11.40 | 81.43 | 18.73 | 17.20 | 218 | 48 | 38.27 | 340 | 214 | 5 |
| 1758 | 34.83 | -1.39 | GBIF | 11.78 | 80.65 | 20.12 | 18.62 | 192 | 40 | 39.13 | 322 | 176 | 7 |
| 1759 | 37.46 | -0.01 | GBIF | 11.85 | 78.48 | 11.52 | 10.60 | 232 | 38 | 62.18 | 411 | 171 | 1 |
| 1760 | 38.11 | -2.99 | GBIF | 10.19 | 67.94 | 23.67 | 20.68 | 138 | 5 | 92.37 | 136 | 19 | 9 |
| 1761 | 36.91 | 0.11 | GBIF | 15.13 | 78.37 | 18.00 | 17.03 | 134 | 22 | 50.29 | 286 | 160 | 4 |
| 1762 | 37.29 | -2.66 | GBIF | 12.53 | 72.45 | 22.53 | 19.77 | 145 | 4 | 83.86 | 175 | 23 | 5 |
| 1763 | 35.31 | -1.71 | GBIF | 12.33 | 80.61 | 19.67 | 17.75 | 155 | 21 | 55.44 | 370 | 86 | 5 |
| 1764 | 39.00 | -3.33 | GBIF | 10.42 | 69.91 | 25.28 | 22.88 | 131 | 24 | 60.34 | 255 | 99 | 2 |
| 1765 | 34.81 | -1.52 | GBIF | 12.41 | 81.63 | 21.30 | 19.87 | 177 | 32 | 41.88 | 304 | 151 | 8 |
| 1766 | 34.85 | 1.53 | GBIF | 12.57 | 83.28 | 20.85 | 21.80 | 148 | 23 | 51.22 | 237 | 377 | 5 |
| 1767 | 35.25 | -1.64 | GBIF | 12.50 | 81.17 | 20.20 | 18.32 | 161 | 22 | 53.12 | 374 | 96 | 8 |
| 1768 | 35.37 | -1.64 | GBIF | 12.32 | 80.50 | 19.78 | 17.77 | 156 | 19 | 57.10 | 366 | 80 | 5 |
| 1769 | 37.49 | 0.77 | GBIF | 12.66 | 83.83 | 23.60 | 22.08 | 132 | 4 | 98.34 | 215 | 16 | 9 |
| 1770 | 37.59 | 0.35 | GBIF | 13.26 | 80.84 | 22.98 | 22.80 | 186 | 5 | 95.51 | 273 | 298 | 19 |
| 1771 | 37.41 | 0.35 | GBIF | 14.00 | 84.34 | 22.40 | 22.18 | 181 | 9 | 90.59 | 290 | 290 | 6 |
| 1772 | 35.39 | -1.27 | GBIF | 12.49 | 82.18 | 17.87 | 16.20 | 194 | 32 | 49.96 | 412 | 134 | 3 |
| 1773 | 37.11 | -2.78 | GBIF | 12.24 | 71.17 | 21.72 | 19.12 | 158 | 6 | 80.52 | 186 | 31 | 5 |
| 1774 | 36.42 | 0.19 | GBIF | 14.76 | 81.54 | 15.47 | 16.52 | 124 | 29 | 45.08 | 260 | 273 | 11 |
| 1775 | 36.60 | 0.21 | GBIF | 15.72 | 80.19 | 18.25 | 17.38 | 119 | 26 | 43.09 | 243 | 200 | 7 |
| 1776 | 36.56 | 0.19 | GBIF | 15.41 | 80.67 | 17.95 | 17.05 | 108 | 28 | 39.98 | 226 | 208 | 6 |
| 1777 | 36.67 | 0.12 | GBIF | 15.28 | 79.60 | 17.70 | 16.70 | 106 | 28 | 40.01 | 222 | 185 | 12 |
| 1778 | 36.42 | 0.57 | GBIF | 13.71 | 82.58 | 17.78 | 17.37 | 117 | 22 | 48.95 | 254 | 233 | 7 |
| 1779 | 36.54 | 0.19 | GBIF | 15.25 | 81.12 | 18.05 | 17.18 | 115 | 28 | 41.04 | 240 | 217 | 6 |
| 1780 | 36.40 | 0.69 | GBIF | 13.43 | 82.41 | 18.07 | 17.68 | 117 | 22 | 50.55 | 270 | 243 | 10 |
| 1781 | 36.41 | 0.64 | GBIF | 13.53 | 83.03 | 18.25 | 17.83 | 124 | 21 | 51.60 | 282 | 243 | 10 |
| 1782 | 36.44 | 0.12 | GBIF | 14.52 | 81.55 | 15.12 | 16.22 | 118 | 30 | 43.80 | 252 | 282 | 7 |
| 1783 | 36.39 | 0.71 | GBIF | 13.42 | 82.31 | 18.37 | 18.00 | 117 | 22 | 51.01 | 274 | 244 | 11 |
| 1784 | 36.42 | 0.60 | GBIF | 13.54 | 82.57 | 17.88 | 17.47 | 122 | 22 | 50.88 | 268 | 236 | 11 |
| 1785 | 36.42 | 0.66 | GBIF | 13.55 | 82.62 | 18.08 | 17.72 | 124 | 22 | 51.42 | 277 | 241 | 10 |
| 1786 | 36.75 | 0.08 | GBIF | 15.13 | 78.78 | 17.57 | 16.47 | 111 | 27 | 40.31 | 238 | 174 | 15 |
| 1787 | 36.82 | 0.00 | GBIF | 14.58 | 78.83 | 17.15 | 16.02 | 111 | 30 | 40.79 | 244 | 183 | 3 |
| 1788 | 36.36 | 0.48 | GBIF | 13.98 | 82.25 | 17.60 | 17.20 | 124 | 24 | 49.34 | 272 | 248 | 13 |
| 1789 | 36.67 | 0.15 | GBIF | 15.35 | 79.12 | 17.83 | 16.83 | 110 | 26 | 41.12 | 226 | 190 | 10 |
| 1790 | 36.38 | 0.59 | GBIF | 13.41 | 82.77 | 18.45 | 18.03 | 128 | 22 | 52.17 | 296 | 249 | 6 |
| 1791 | 36.38 | 0.51 | GBIF | 13.69 | 82.48 | 17.90 | 17.52 | 133 | 23 | 51.80 | 287 | 252 | 12 |
| 1792 | 36.38 | 0.53 | GBIF | 13.60 | 82.42 | 17.98 | 17.58 | 130 | 23 | 50.93 | 282 | 250 | 7 |
| 1793 | 36.68 | 0.17 | GBIF | 15.42 | 79.06 | 17.88 | 16.88 | 114 | 25 | 42.85 | 230 | 186 | 3 |
| 1794 | 36.64 | 0.15 | GBIF | 15.42 | 79.47 | 17.80 | 16.83 | 108 | 28 | 40.68 | 221 | 190 | 14 |
| 1795 | 36.85 | 0.03 | GBIF | 14.74 | 78.41 | 17.53 | 16.38 | 121 | 27 | 44.01 | 264 | 174 | 10 |
| 1796 | 36.41 | 0.69 | GBIF | 13.49 | 82.77 | 18.18 | 17.78 | 119 | 22 | 50.91 | 273 | 242 | 10 |
| 1797 | 36.84 | 0.19 | GBIF | 15.13 | 78.41 | 17.98 | 16.97 | 137 | 19 | 52.45 | 279 | 167 | 3 |
| 1798 | 36.35 | 0.53 | GBIF | 13.39 | 82.66 | 17.73 | 17.33 | 130 | 24 | 50.26 | 286 | 255 | 6 |
| 1799 | 36.70 | 0.12 | GBIF | 15.30 | 79.27 | 17.67 | 16.65 | 108 | 27 | 40.04 | 225 | 183 | 3 |

| 1800 | 36.42 | 0.68 | GBIF | 13.52 | 82.98 | 18.08 | 17.73 | 119 | 22 | 50.63 | 274 | 242 | 6 |
| --- | --- | --- | --- | --- | --- | --- | --- | --- | --- | --- | --- | --- | --- |
| 1801 | 36.42 | 0.58 | GBIF | 13.63 | 82.63 | 17.87 | 17.48 | 117 | 22 | 50.07 | 260 | 234 | 11 |
| 1802 | 36.73 | 0.08 | GBIF | 15.15 | 78.91 | 17.62 | 16.53 | 113 | 27 | 40.52 | 241 | 175 | 19 |
| 1803 | 36.31 | 0.50 | GBIF | 13.69 | 82.48 | 17.77 | 17.40 | 134 | 25 | 50.96 | 293 | 266 | 7 |
| 1804 | 36.60 | 0.18 | GBIF | 15.61 | 80.04 | 18.10 | 17.15 | 115 | 27 | 41.38 | 234 | 204 | 9 |
| 1805 | 36.72 | 0.15 | GBIF | 15.37 | 79.21 | 17.90 | 16.87 | 118 | 24 | 43.33 | 244 | 182 | 10 |
| 1806 | 37.85 | 0.88 | GBIF | 12.59 | 83.39 | 23.97 | 23.22 | 129 | 1 | 113.68 | 183 | 5 | 3 |
| 1807 | 37.84 | 0.73 | GBIF | 12.73 | 83.72 | 24.08 | 23.35 | 132 | 1 | 115.57 | 170 | 5 | 2 |
| 1808 | 36.94 | 0.05 | GBIF | 14.53 | 78.51 | 17.50 | 16.45 | 128 | 25 | 49.14 | 280 | 145 | 11 |
| 1809 | 36.44 | 0.64 | GBIF | 13.59 | 82.37 | 17.90 | 17.50 | 117 | 21 | 50.85 | 267 | 237 | 10 |
| 1810 | 36.40 | 0.62 | GBIF | 13.43 | 82.87 | 18.33 | 17.93 | 128 | 22 | 52.25 | 287 | 245 | 6 |
| 1811 | 36.34 | 0.11 | GBIF | 13.46 | 82.06 | 13.25 | 14.48 | 130 | 33 | 42.23 | 262 | 306 | 4 |
| 1812 | 36.82 | -0.05 | GBIF | 14.43 | 79.26 | 17.47 | 16.22 | 119 | 35 | 41.95 | 267 | 198 | 7 |
| 1813 | 37.77 | 0.67 | GBIF | 12.73 | 83.77 | 23.90 | 23.15 | 128 | 2 | 111.81 | 167 | 8 | 8 |
| 1814 | 36.30 | 0.53 | GBIF | 13.52 | 82.92 | 18.13 | 17.77 | 137 | 25 | 51.33 | 305 | 264 | 6 |
| 1815 | 36.87 | 0.00 | GBIF | 14.55 | 78.23 | 17.60 | 16.42 | 127 | 29 | 45.97 | 282 | 184 | 4 |
| 1816 | 36.96 | 0.01 | GBIF | 14.48 | 78.71 | 17.47 | 16.37 | 126 | 27 | 48.66 | 283 | 185 | 14 |
| 1817 | 36.41 | 0.67 | GBIF | 13.53 | 83.03 | 18.18 | 17.83 | 122 | 21 | 51.80 | 280 | 242 | 10 |
| 1818 | 36.34 | 0.53 | GBIF | 13.39 | 82.66 | 17.75 | 17.33 | 131 | 25 | 50.24 | 287 | 258 | 6 |
| 1819 | 36.86 | 0.00 | GBIF | 14.51 | 78.42 | 17.55 | 16.35 | 126 | 30 | 45.01 | 277 | 185 | 3 |
| 1820 | 36.43 | 0.63 | GBIF | 13.60 | 82.42 | 18.08 | 17.68 | 121 | 21 | 51.51 | 274 | 236 | 10 |
| 1821 | 37.80 | 0.97 | GBIF | 12.32 | 82.66 | 23.75 | 23.05 | 124 | 2 | 111.01 | 186 | 6 | 3 |
| 1822 | 36.82 | -0.01 | GBIF | 14.52 | 78.89 | 17.28 | 16.10 | 116 | 31 | 41.68 | 255 | 186 | 3 |
| 1823 | 37.66 | 0.98 | GBIF | 12.71 | 82.52 | 24.27 | 22.62 | 127 | 2 | 106.21 | 202 | 9 | 4 |
| 1824 | 36.86 | -0.03 | GBIF | 14.38 | 78.60 | 17.47 | 16.23 | 122 | 32 | 44.00 | 273 | 191 | 3 |
| 1825 | 36.86 | 0.07 | GBIF | 14.82 | 78.44 | 17.67 | 16.55 | 128 | 24 | 46.69 | 272 | 169 | 10 |
| 1826 | 37.77 | 0.70 | GBIF | 12.16 | 82.71 | 22.33 | 21.70 | 197 | 3 | 107.11 | 280 | 12 | 4 |
| 1827 | 36.77 | 0.08 | GBIF | 15.06 | 78.43 | 17.60 | 16.52 | 111 | 26 | 40.71 | 242 | 170 | 19 |
| 1828 | 36.86 | -0.01 | GBIF | 14.45 | 78.53 | 17.52 | 16.30 | 127 | 30 | 45.56 | 279 | 186 | 10 |
| 1829 | 36.83 | 0.03 | GBIF | 14.79 | 78.26 | 17.52 | 16.37 | 116 | 28 | 42.10 | 255 | 175 | 3 |
| 1830 | 36.37 | 0.55 | GBIF | 13.47 | 82.62 | 18.00 | 17.58 | 131 | 23 | 51.95 | 291 | 251 | 6 |
| 1831 | 37.83 | 0.89 | GBIF | 12.46 | 83.06 | 23.80 | 23.07 | 131 | 1 | 113.53 | 184 | 5 | 3 |
| 1832 | 37.46 | 0.59 | GBIF | 13.50 | 83.85 | 23.52 | 23.05 | 125 | 5 | 94.79 | 183 | 19 | 17 |
| 1833 | 37.88 | 0.89 | GBIF | 12.65 | 83.22 | 23.98 | 23.27 | 131 | 1 | 115.29 | 183 | 5 | 3 |
| 1834 | 37.86 | 0.89 | GBIF | 12.59 | 83.39 | 23.95 | 23.20 | 130 | 1 | 114.80 | 187 | 5 | 3 |
| 1835 | 36.89 | -0.05 | GBIF | 14.41 | 78.73 | 17.57 | 16.30 | 125 | 33 | 46.40 | 285 | 196 | 12 |
| 1836 | 37.65 | 0.90 | GBIF | 12.73 | 83.22 | 24.37 | 22.97 | 125 | 2 | 106.54 | 196 | 10 | 3 |
| 1837 | 37.86 | 0.86 | GBIF | 12.57 | 83.22 | 23.97 | 23.22 | 129 | 1 | 114.39 | 183 | 5 | 3 |
| 1838 | 37.94 | 0.83 | GBIF | 12.55 | 83.67 | 24.33 | 23.58 | 129 | 1 | 117.71 | 178 | 4 | 10 |
| 1839 | 37.84 | 0.88 | GBIF | 12.58 | 83.33 | 23.90 | 23.22 | 130 | 1 | 114.87 | 185 | 5 | 3 |
| 1840 | 37.49 | 0.58 | GBIF | 13.48 | 83.70 | 23.52 | 23.05 | 126 | 5 | 96.41 | 184 | 17 | 15 |
| 1841 | 36.38 | 0.50 | GBIF | 14.01 | 82.40 | 17.92 | 17.50 | 136 | 23 | 51.97 | 286 | 249 | 13 |
| 1842 | 36.83 | -0.03 | GBIF | 14.44 | 78.92 | 17.42 | 16.20 | 119 | 33 | 41.98 | 265 | 190 | 4 |
| 1843 | 37.65 | 0.85 | GBIF | 12.76 | 83.39 | 23.25 | 22.67 | 132 | 3 | 107.04 | 195 | 10 | 4 |
| 1844 | 36.34 | 0.48 | GBIF | 14.02 | 82.02 | 17.68 | 17.33 | 129 | 25 | 49.49 | 283 | 257 | 10 |
| 1845 | 37.89 | 0.79 | GBIF | 11.84 | 82.23 | 23.77 | 22.97 | 136 | 1 | 116.07 | 186 | 5 | 7 |
| 1846 | 37.56 | 0.55 | GBIF | 13.41 | 82.77 | 23.57 | 23.08 | 126 | 4 | 101.07 | 171 | 14 | 19 |
| 1847 | 37.97 | 0.87 | GBIF | 12.39 | 82.61 | 24.32 | 23.50 | 130 | 1 | 117.16 | 176 | 3 | 2 |
| 1848 | 37.65 | 0.92 | GBIF | 12.81 | 83.17 | 24.42 | 22.82 | 123 | 3 | 105.30 | 193 | 10 | 3 |
| 1849 | 37.78 | 0.81 | GBIF | 12.52 | 83.50 | 23.55 | 22.87 | 136 | 2 | 111.85 | 191 | 8 | 3 |

| 1850 | 37.49 | 0.59 | GBIF | 13.34 | 83.91 | 23.45 | 23.13 | 125 | 4 | 97.40 | 183 | 17 | 15 |
| --- | --- | --- | --- | --- | --- | --- | --- | --- | --- | --- | --- | --- | --- |
| 1851 | 37.66 | 0.97 | GBIF | 12.74 | 82.74 | 24.20 | 22.57 | 129 | 2 | 106.84 | 202 | 8 | 4 |
| 1852 | 37.72 | 0.78 | GBIF | 12.74 | 83.28 | 23.20 | 22.62 | 141 | 2 | 108.08 | 204 | 10 | 4 |
| 1853 | 37.58 | 1.26 | GBIF | 12.23 | 82.66 | 24.68 | 23.22 | 118 | 2 | 104.91 | 189 | 9 | 3 |
| 1854 | 36.84 | -0.03 | GBIF | 14.41 | 78.73 | 17.45 | 16.23 | 121 | 32 | 43.39 | 270 | 191 | 3 |
| 1855 | 37.73 | 0.82 | GBIF | 12.62 | 83.00 | 23.15 | 22.53 | 141 | 3 | 107.98 | 201 | 10 | 4 |
| 1856 | 37.51 | 0.62 | GBIF | 13.06 | 83.71 | 23.03 | 22.60 | 134 | 5 | 97.43 | 201 | 17 | 6 |
| 1857 | 37.65 | 0.97 | GBIF | 12.79 | 83.06 | 24.30 | 22.67 | 126 | 3 | 104.96 | 199 | 10 | 3 |
| 1858 | 37.92 | 0.91 | GBIF | 12.48 | 82.67 | 24.17 | 23.40 | 128 | 1 | 115.48 | 182 | 4 | 3 |
| 1859 | 37.95 | 0.73 | GBIF | 12.66 | 83.83 | 24.38 | 23.53 | 132 | 1 | 119.18 | 170 | 3 | 11 |
| 1860 | 37.44 | 1.04 | GBIF | 12.16 | 82.71 | 23.92 | 22.48 | 116 | 4 | 96.16 | 196 | 15 | 12 |
| 1861 | 37.76 | 0.74 | GBIF | 12.02 | 82.31 | 22.37 | 21.78 | 179 | 3 | 107.30 | 254 | 12 | 8 |
| 1862 | 37.52 | 0.60 | GBIF | 13.18 | 83.39 | 23.17 | 22.75 | 135 | 5 | 97.01 | 196 | 17 | 7 |
| 1863 | 37.38 | 0.77 | GBIF | 13.30 | 84.71 | 23.22 | 21.83 | 160 | 7 | 93.95 | 265 | 22 | 3 |
| 1864 | 37.55 | 0.56 | GBIF | 13.34 | 82.87 | 23.57 | 23.08 | 125 | 4 | 100.21 | 173 | 15 | 16 |
| 1865 | 37.37 | 0.75 | GBIF | 13.58 | 85.43 | 23.48 | 22.10 | 154 | 7 | 93.28 | 257 | 23 | 10 |
| 1866 | 37.64 | 0.91 | GBIF | 12.80 | 83.12 | 24.32 | 22.73 | 125 | 3 | 105.94 | 197 | 10 | 3 |
| 1867 | 37.37 | 0.76 | GBIF | 13.48 | 84.80 | 23.40 | 22.02 | 158 | 7 | 93.94 | 261 | 23 | 10 |
| 1868 | 37.43 | 1.08 | GBIF | 11.93 | 82.30 | 23.83 | 22.38 | 117 | 3 | 96.96 | 197 | 15 | 10 |
| 1869 | 37.53 | 0.62 | GBIF | 13.02 | 83.44 | 22.97 | 22.53 | 139 | 4 | 98.22 | 207 | 16 | 10 |
| 1870 | 37.61 | 1.17 | GBIF | 12.29 | 82.49 | 24.77 | 23.18 | 113 | 2 | 106.35 | 182 | 7 | 10 |
| 1871 | 37.36 | 0.77 | GBIF | 13.57 | 84.84 | 23.38 | 21.98 | 157 | 7 | 92.86 | 262 | 23 | 14 |
| 1872 | 37.56 | 0.98 | GBIF | 12.52 | 82.95 | 24.10 | 22.70 | 127 | 3 | 102.82 | 207 | 13 | 6 |
| 1873 | 37.58 | 1.18 | GBIF | 12.33 | 82.77 | 24.63 | 23.13 | 114 | 2 | 103.58 | 186 | 9 | 10 |
| 1874 | 37.57 | 1.27 | GBIF | 12.19 | 82.38 | 24.67 | 23.17 | 117 | 2 | 104.59 | 188 | 9 | 3 |
| 1875 | 37.49 | 0.61 | GBIF | 13.26 | 83.39 | 23.40 | 23.08 | 126 | 4 | 96.64 | 185 | 17 | 10 |
| 1876 | 37.60 | 0.91 | GBIF | 12.68 | 82.84 | 24.07 | 22.48 | 125 | 3 | 103.98 | 201 | 11 | 3 |
| 1877 | 37.59 | 1.17 | GBIF | 12.35 | 82.33 | 24.68 | 23.07 | 115 | 2 | 104.90 | 187 | 8 | 10 |
| 1878 | 37.58 | 1.21 | GBIF | 12.37 | 82.44 | 24.70 | 23.23 | 117 | 2 | 105.33 | 188 | 9 | 2 |
| 1879 | 37.48 | 0.59 | GBIF | 13.45 | 84.06 | 23.52 | 23.20 | 125 | 4 | 97.10 | 184 | 17 | 17 |
| 1880 | 37.42 | 0.63 | GBIF | 13.23 | 84.29 | 23.62 | 22.20 | 147 | 7 | 92.81 | 244 | 22 | 9 |
| 1881 | 37.54 | 1.19 | GBIF | 12.39 | 82.61 | 24.47 | 22.98 | 116 | 2 | 103.43 | 190 | 9 | 7 |
| 1882 | 37.58 | 0.99 | GBIF | 12.59 | 83.39 | 24.22 | 22.65 | 125 | 3 | 104.56 | 204 | 10 | 8 |
| 1883 | 37.67 | 0.99 | GBIF | 12.64 | 82.63 | 24.25 | 22.63 | 126 | 2 | 106.46 | 202 | 9 | 7 |
| 1884 | 37.39 | 0.75 | GBIF | 13.42 | 84.92 | 23.42 | 22.00 | 149 | 7 | 93.93 | 249 | 22 | 8 |
| 1885 | 37.62 | 1.26 | GBIF | 11.92 | 81.62 | 24.90 | 23.38 | 108 | 1 | 106.33 | 175 | 6 | 3 |
| 1886 | 37.58 | 1.05 | GBIF | 12.68 | 83.44 | 24.67 | 23.15 | 121 | 2 | 104.50 | 195 | 11 | 4 |
| 1887 | 37.59 | 1.03 | GBIF | 12.59 | 82.84 | 24.53 | 23.07 | 119 | 2 | 105.15 | 193 | 10 | 3 |
| 1888 | 37.55 | 0.93 | GBIF | 12.32 | 83.22 | 23.83 | 22.38 | 126 | 3 | 101.50 | 209 | 13 | 4 |
| 1889 | 37.55 | 0.99 | GBIF | 12.33 | 83.33 | 24.08 | 22.50 | 123 | 3 | 102.50 | 202 | 12 | 5 |
| 1890 | 37.45 | 0.99 | GBIF | 12.02 | 82.87 | 23.45 | 22.07 | 136 | 4 | 97.62 | 225 | 16 | 18 |
| 1891 | 37.46 | 0.89 | GBIF | 11.81 | 82.58 | 22.77 | 21.30 | 155 | 5 | 98.32 | 256 | 18 | 10 |
| 1892 | 37.59 | 0.92 | GBIF | 12.77 | 83.44 | 24.10 | 22.52 | 125 | 3 | 103.64 | 203 | 10 | 2 |
| 1893 | 37.45 | 0.93 | GBIF | 11.39 | 81.95 | 22.75 | 21.27 | 146 | 5 | 96.99 | 243 | 18 | 17 |
| 1894 | 37.55 | 0.89 | GBIF | 12.12 | 82.43 | 23.33 | 21.77 | 139 | 4 | 100.68 | 228 | 13 | 13 |
| 1895 | 37.63 | 0.94 | GBIF | 12.82 | 82.69 | 24.32 | 22.70 | 125 | 3 | 106.04 | 200 | 10 | 3 |
| 1896 | 37.56 | 0.91 | GBIF | 12.32 | 83.22 | 23.73 | 22.32 | 128 | 3 | 101.59 | 210 | 13 | 7 |
| 1897 | 37.48 | 1.03 | GBIF | 12.12 | 82.43 | 23.82 | 22.37 | 124 | 3 | 99.18 | 208 | 14 | 3 |
| 1898 | 37.37 | 1.62 | GBIF | 11.45 | 81.21 | 25.22 | 23.62 | 107 | 2 | 102.65 | 169 | 11 | 8 |
| 1899 | 37.48 | 0.93 | GBIF | 11.80 | 82.52 | 23.27 | 21.85 | 136 | 4 | 97.97 | 227 | 17 | 17 |

| 1900 | 37.55 | 1.21 | GBIF | 12.37 | 82.44 | 24.53 | 23.07 | 114 | 2 | 102.87 | 188 | 9 | 7 |
| --- | --- | --- | --- | --- | --- | --- | --- | --- | --- | --- | --- | --- | --- |
| 1901 | 37.62 | 1.25 | GBIF | 12.02 | 81.75 | 24.83 | 23.33 | 110 | 1 | 106.59 | 178 | 7 | 3 |
| 1902 | 37.60 | 1.21 | GBIF | 12.30 | 82.00 | 24.73 | 23.23 | 117 | 2 | 105.97 | 187 | 9 | 4 |
| 1903 | 37.57 | 1.06 | GBIF | 12.65 | 83.22 | 24.58 | 23.10 | 118 | 2 | 102.87 | 193 | 11 | 7 |
| 1904 | 37.72 | 1.19 | GBIF | 12.02 | 81.80 | 25.27 | 23.62 | 112 | 1 | 111.46 | 176 | 5 | 8 |
| 1905 | 37.46 | 0.87 | GBIF | 11.95 | 82.99 | 22.78 | 21.35 | 152 | 5 | 98.41 | 254 | 17 | 13 |
| 1906 | 37.59 | 0.93 | GBIF | 12.76 | 83.39 | 24.12 | 22.53 | 126 | 3 | 103.76 | 200 | 10 | 4 |
| 1907 | 37.56 | 0.96 | GBIF | 12.57 | 83.22 | 23.98 | 22.57 | 125 | 3 | 102.47 | 205 | 13 | 11 |
| 1908 | 37.58 | 0.95 | GBIF | 12.71 | 83.06 | 23.98 | 22.57 | 127 | 3 | 102.76 | 206 | 12 | 6 |
| 1909 | 37.12 | 1.08 | GBIF | 13.74 | 84.30 | 22.43 | 20.67 | 185 | 10 | 91.46 | 357 | 31 | 8 |
| 1910 | 37.63 | 0.97 | GBIF | 12.78 | 83.01 | 24.28 | 22.67 | 125 | 3 | 104.95 | 203 | 10 | 3 |
| 1911 | 37.40 | 1.03 | GBIF | 12.14 | 83.16 | 23.57 | 22.18 | 132 | 4 | 96.47 | 218 | 17 | 11 |
| 1912 | 37.74 | 1.21 | GBIF | 11.96 | 81.91 | 25.30 | 23.60 | 109 | 1 | 111.97 | 173 | 4 | 3 |
| 1913 | 37.31 | 0.22 | GBIF | 13.39 | 83.18 | 16.52 | 16.18 | 176 | 23 | 69.43 | 246 | 118 | 15 |
| 1914 | 37.63 | 0.88 | GBIF | 12.61 | 83.50 | 23.17 | 22.57 | 129 | 3 | 104.59 | 194 | 10 | 3 |
| 1915 | 37.65 | 1.14 | GBIF | 12.24 | 82.16 | 25.00 | 23.52 | 110 | 1 | 108.57 | 177 | 6 | 8 |
| 1916 | 37.59 | 0.98 | GBIF | 12.71 | 83.06 | 24.20 | 22.62 | 125 | 3 | 104.67 | 204 | 10 | 4 |
| 1917 | 37.65 | 1.05 | GBIF | 12.28 | 81.89 | 24.35 | 22.72 | 122 | 2 | 107.43 | 196 | 7 | 3 |
| 1918 | 37.46 | 0.20 | GBIF | 13.48 | 82.72 | 18.67 | 17.80 | 282 | 13 | 94.89 | 378 | 45 | 19 |
| 1919 | 37.43 | 1.05 | GBIF | 12.17 | 82.77 | 23.83 | 22.42 | 121 | 4 | 96.53 | 202 | 15 | 11 |
| 1920 | 37.34 | 1.31 | GBIF | 11.02 | 80.41 | 23.23 | 21.68 | 126 | 4 | 93.28 | 207 | 21 | 9 |
| 1921 | 37.32 | 1.57 | GBIF | 11.65 | 81.47 | 25.00 | 23.43 | 99 | 2 | 97.97 | 160 | 13 | 8 |
| 1922 | 37.45 | 0.98 | GBIF | 11.63 | 83.04 | 23.07 | 21.68 | 145 | 5 | 97.46 | 239 | 17 | 14 |
| 1923 | 37.32 | 1.50 | GBIF | 11.44 | 81.15 | 24.40 | 22.78 | 109 | 3 | 96.45 | 177 | 15 | 8 |
| 1924 | 37.61 | 0.97 | GBIF | 12.80 | 83.12 | 24.37 | 22.75 | 121 | 3 | 104.54 | 198 | 10 | 3 |
| 1925 | 37.57 | 0.88 | GBIF | 12.22 | 83.11 | 23.52 | 21.98 | 135 | 4 | 101.50 | 213 | 12 | 6 |
| 1926 | 37.16 | 0.31 | GBIF | 14.23 | 83.19 | 18.52 | 17.67 | 145 | 18 | 66.77 | 224 | 123 | 9 |
| 1927 | 37.38 | 0.22 | GBIF | 13.64 | 83.69 | 18.28 | 17.55 | 241 | 16 | 90.04 | 341 | 56 | 5 |
| 1928 | 37.49 | 0.27 | GBIF | 13.17 | 82.29 | 19.27 | 18.63 | 285 | 11 | 97.34 | 384 | 41 | 11 |
| 1929 | 37.46 | 0.91 | GBIF | 11.58 | 82.74 | 22.62 | 21.15 | 151 | 5 | 97.62 | 253 | 17 | 10 |
| 1930 | 37.58 | 0.92 | GBIF | 12.67 | 83.89 | 23.97 | 22.40 | 127 | 3 | 103.05 | 206 | 12 | 6 |
| 1931 | 37.17 | 0.24 | GBIF | 13.99 | 80.88 | 17.70 | 16.87 | 134 | 17 | 61.31 | 209 | 139 | 6 |
| 1932 | 37.52 | 0.94 | GBIF | 12.02 | 82.87 | 23.60 | 22.15 | 132 | 4 | 100.19 | 217 | 15 | 10 |
| 1933 | 37.31 | 0.24 | GBIF | 13.53 | 83.54 | 17.07 | 16.63 | 183 | 24 | 72.36 | 256 | 109 | 16 |
| 1934 | 37.52 | 0.25 | GBIF | 13.21 | 82.55 | 19.92 | 19.23 | 287 | 7 | 100.15 | 365 | 28 | 16 |
| 1935 | 37.59 | 0.87 | GBIF | 12.40 | 83.22 | 23.82 | 22.27 | 132 | 3 | 104.08 | 204 | 11 | 2 |
| 1936 | 37.16 | 0.46 | GBIF | 12.86 | 82.96 | 19.08 | 17.78 | 171 | 19 | 77.02 | 259 | 93 | 7 |
| 1937 | 37.16 | 0.24 | GBIF | 13.88 | 80.67 | 17.45 | 16.63 | 128 | 17 | 58.85 | 200 | 146 | 6 |
| 1938 | 37.71 | 1.23 | GBIF | 11.77 | 81.15 | 25.03 | 23.35 | 110 | 1 | 109.45 | 176 | 5 | 3 |
| 1939 | 37.17 | 0.27 | GBIF | 14.30 | 82.18 | 18.23 | 17.35 | 140 | 18 | 63.89 | 217 | 132 | 6 |
| 1940 | 36.88 | 0.49 | GBIF | 14.88 | 82.69 | 19.87 | 18.90 | 198 | 22 | 71.16 | 400 | 140 | 7 |
| 1941 | 36.84 | 0.63 | GBIF | 14.36 | 83.48 | 19.53 | 18.48 | 178 | 21 | 69.04 | 373 | 139 | 5 |
| 1942 | 37.73 | 1.19 | GBIF | 12.04 | 81.92 | 25.30 | 23.63 | 111 | 1 | 111.12 | 175 | 5 | 3 |
| 1943 | 37.35 | 0.25 | GBIF | 13.49 | 83.80 | 18.67 | 17.98 | 227 | 17 | 89.00 | 328 | 57 | 6 |
| 1944 | 37.66 | 1.14 | GBIF | 12.22 | 81.99 | 24.90 | 23.42 | 113 | 1 | 108.39 | 181 | 7 | 2 |
| 1945 | 37.43 | 0.20 | GBIF | 13.57 | 82.72 | 18.48 | 17.67 | 269 | 14 | 93.99 | 373 | 49 | 12 |
| 1946 | 37.60 | 0.98 | GBIF | 12.73 | 83.22 | 24.38 | 22.78 | 122 | 3 | 104.20 | 199 | 10 | 3 |
| 1947 | 36.82 | 0.58 | GBIF | 14.48 | 83.67 | 19.13 | 18.17 | 157 | 20 | 62.83 | 330 | 140 | 5 |
| 1948 | 36.80 | 0.56 | GBIF | 14.59 | 83.38 | 18.77 | 17.80 | 139 | 18 | 58.30 | 293 | 151 | 5 |
| 1949 | 36.84 | 0.60 | GBIF | 14.43 | 83.91 | 19.78 | 18.75 | 189 | 23 | 69.83 | 394 | 127 | 5 |

| 1950 | 36.85 | 0.59 | GBIF | 14.43 | 83.43 | 19.70 | 18.68 | 184 | 22 | 69.66 | 383 | 125 | 6 |
| --- | --- | --- | --- | --- | --- | --- | --- | --- | --- | --- | --- | --- | --- |
| 1951 | 37.08 | 0.55 | GBIF | 13.13 | 83.65 | 19.83 | 18.45 | 203 | 23 | 80.34 | 400 | 91 | 5 |
| 1952 | 37.14 | 0.32 | GBIF | 14.12 | 83.04 | 18.63 | 17.77 | 148 | 18 | 66.95 | 229 | 122 | 9 |
| 1953 | 37.17 | 0.30 | GBIF | 14.37 | 83.53 | 18.52 | 17.63 | 144 | 18 | 65.85 | 224 | 125 | 7 |
| 1954 | 38.52 | 2.46 | GBIF | 9.77 | 70.83 | 27.12 | 24.93 | 97 | 0 | 118.25 | 52 | 2 | 2 |
| 1955 | 36.94 | 0.48 | GBIF | 14.88 | 83.10 | 19.53 | 18.60 | 180 | 20 | 69.82 | 367 | 124 | 11 |
| 1956 | 37.18 | 0.28 | GBIF | 14.27 | 82.95 | 18.38 | 17.50 | 144 | 18 | 65.57 | 224 | 128 | 7 |
| 1957 | 37.13 | 0.31 | GBIF | 14.02 | 82.50 | 18.53 | 17.67 | 145 | 17 | 65.35 | 224 | 129 | 7 |
| 1958 | 37.15 | 0.30 | GBIF | 14.23 | 83.24 | 18.40 | 17.48 | 141 | 17 | 64.35 | 296 | 131 | 6 |
| 1959 | 37.15 | 0.32 | GBIF | 14.12 | 83.04 | 18.63 | 17.75 | 151 | 18 | 67.41 | 232 | 122 | 8 |
| 1960 | 36.65 | 0.48 | GBIF | 15.04 | 81.75 | 18.17 | 17.70 | 121 | 17 | 52.87 | 256 | 175 | 9 |
| 1961 | 38.59 | 2.55 | GBIF | 9.46 | 70.06 | 27.02 | 24.80 | 99 | 0 | 117.48 | 53 | 2 | 2 |
| 1962 | 36.83 | 0.51 | GBIF | 14.79 | 83.10 | 19.65 | 18.67 | 181 | 22 | 66.44 | 375 | 151 | 5 |
| 1963 | 36.84 | 0.66 | GBIF | 14.48 | 83.67 | 19.12 | 18.08 | 157 | 19 | 64.60 | 328 | 129 | 9 |
| 1964 | 37.32 | 0.23 | GBIF | 13.47 | 83.18 | 17.13 | 16.50 | 191 | 25 | 75.24 | 268 | 99 | 11 |
| 1965 | 37.07 | 0.30 | GBIF | 13.93 | 81.48 | 18.00 | 17.15 | 129 | 15 | 58.84 | 198 | 149 | 7 |
| 1966 | 36.78 | 0.43 | GBIF | 14.46 | 81.23 | 18.95 | 18.02 | 155 | 20 | 58.57 | 322 | 173 | 10 |
| 1967 | 37.35 | 0.28 | GBIF | 12.86 | 83.50 | 18.62 | 17.95 | 220 | 18 | 88.26 | 323 | 61 | 11 |
| 1968 | 37.10 | 0.38 | GBIF | 14.19 | 82.99 | 18.95 | 18.03 | 154 | 18 | 68.27 | 314 | 120 | 11 |
| 1969 | 36.82 | 0.44 | GBIF | 14.59 | 81.52 | 19.32 | 18.37 | 173 | 21 | 62.75 | 352 | 162 | 3 |
| 1970 | 37.89 | 1.07 | GBIF | 12.12 | 81.87 | 24.20 | 23.45 | 117 | 1 | 114.79 | 179 | 3 | 2 |
| 1971 | 37.87 | 1.13 | GBIF | 12.17 | 82.26 | 25.10 | 23.42 | 119 | 1 | 114.03 | 185 | 4 | 2 |
| 1972 | 36.86 | 0.60 | GBIF | 14.35 | 83.43 | 19.88 | 18.82 | 194 | 23 | 71.87 | 404 | 121 | 14 |
| 1973 | 36.84 | 0.62 | GBIF | 14.39 | 83.67 | 19.60 | 18.57 | 183 | 22 | 69.85 | 379 | 125 | 5 |
| 1974 | 37.87 | 1.17 | GBIF | 12.32 | 82.11 | 25.17 | 23.50 | 119 | 1 | 116.04 | 183 | 3 | 3 |
| 1975 | 36.82 | 0.46 | GBIF | 14.71 | 82.17 | 19.35 | 18.40 | 172 | 21 | 63.24 | 351 | 159 | 9 |
| 1976 | 37.85 | 1.08 | GBIF | 12.13 | 81.98 | 24.00 | 23.28 | 119 | 1 | 113.38 | 183 | 4 | 2 |
| 1977 | 36.86 | 0.66 | GBIF | 14.25 | 83.82 | 18.88 | 17.87 | 146 | 18 | 63.49 | 303 | 126 | 13 |
| 1978 | 37.46 | 0.25 | GBIF | 13.42 | 82.82 | 19.18 | 18.40 | 269 | 10 | 96.49 | 368 | 39 | 6 |
| 1979 | 36.82 | 0.41 | GBIF | 14.60 | 81.11 | 18.62 | 17.68 | 137 | 17 | 56.76 | 283 | 167 | 3 |
| 1980 | 36.83 | 0.54 | GBIF | 14.66 | 83.29 | 19.10 | 18.13 | 151 | 19 | 61.68 | 318 | 152 | 6 |
| 1981 | 36.89 | 0.47 | GBIF | 14.90 | 82.78 | 19.63 | 18.67 | 190 | 21 | 68.95 | 378 | 144 | 6 |
| 1982 | 37.88 | 1.14 | GBIF | 12.14 | 82.04 | 25.18 | 23.50 | 114 | 1 | 114.56 | 177 | 3 | 3 |
| 1983 | 36.86 | 0.65 | GBIF | 14.21 | 83.58 | 19.32 | 18.33 | 170 | 20 | 68.00 | 351 | 122 | 13 |
| 1984 | 36.82 | 0.50 | GBIF | 14.77 | 82.50 | 19.53 | 18.57 | 175 | 21 | 64.89 | 366 | 155 | 5 |
| 1985 | 37.76 | 1.15 | GBIF | 12.11 | 82.37 | 25.17 | 23.48 | 112 | 1 | 111.82 | 177 | 5 | 3 |
| 1986 | 37.93 | 1.11 | GBIF | 11.98 | 82.02 | 24.35 | 23.58 | 116 | 1 | 116.09 | 173 | 3 | 3 |
| 1987 | 36.83 | 0.68 | GBIF | 14.64 | 84.15 | 19.08 | 18.05 | 154 | 19 | 63.67 | 319 | 130 | 9 |
| 1988 | 36.79 | 0.54 | GBIF | 14.72 | 82.68 | 18.80 | 17.83 | 138 | 18 | 58.17 | 289 | 151 | 5 |
| 1989 | 36.84 | 0.56 | GBIF | 14.51 | 83.38 | 19.70 | 18.65 | 184 | 22 | 68.43 | 384 | 145 | 6 |
| 1990 | 36.85 | 0.54 | GBIF | 14.68 | 83.38 | 19.43 | 18.43 | 171 | 20 | 66.18 | 350 | 134 | 6 |
| 1991 | 37.85 | 1.11 | GBIF | 12.17 | 82.21 | 25.00 | 23.33 | 123 | 1 | 114.85 | 190 | 4 | 2 |
| 1992 | 36.62 | 0.74 | GBIF | 14.76 | 82.45 | 18.17 | 17.52 | 117 | 18 | 51.22 | 260 | 188 | 10 |
| 1993 | 36.60 | 0.75 | GBIF | 14.59 | 82.44 | 18.07 | 17.43 | 114 | 19 | 50.94 | 256 | 193 | 8 |
| 1994 | 36.64 | 0.72 | GBIF | 14.89 | 82.73 | 18.25 | 17.57 | 119 | 18 | 51.67 | 265 | 183 | 6 |
| 1995 | 37.85 | 1.13 | GBIF | 12.21 | 81.94 | 25.07 | 23.40 | 119 | 1 | 114.22 | 185 | 4 | 2 |
| 1996 | 37.77 | 1.21 | GBIF | 12.04 | 81.92 | 25.05 | 23.33 | 111 | 1 | 111.49 | 176 | 5 | 3 |
| 1997 | 37.89 | 1.06 | GBIF | 12.14 | 82.04 | 24.18 | 23.42 | 119 | 1 | 114.97 | 182 | 5 | 1 |
| 1998 | 37.78 | 1.19 | GBIF | 12.30 | 82.55 | 25.42 | 23.73 | 107 | 1 | 112.28 | 168 | 5 | 3 |
| 1999 | 37.86 | 1.10 | GBIF | 12.13 | 82.54 | 25.00 | 23.30 | 121 | 1 | 114.95 | 187 | 4 | 2 |

| 2000 | 37.98 | 1.14 | GBIF | 11.67 | 81.02 | 24.55 | 23.73 | 113 | 0 | 118.16 | 170 | 2 | 3 |
| --- | --- | --- | --- | --- | --- | --- | --- | --- | --- | --- | --- | --- | --- |
| 2001 | 36.83 | 0.57 | GBIF | 14.48 | 83.24 | 19.38 | 18.38 | 169 | 20 | 65.46 | 353 | 137 | 10 |
| 2002 | 38.58 | 2.56 | GBIF | 9.45 | 69.49 | 26.93 | 24.72 | 102 | 0 | 117.99 | 54 | 2 | 2 |
| 2003 | 37.93 | 1.18 | GBIF | 11.93 | 81.68 | 25.37 | 23.70 | 113 | 1 | 117.13 | 174 | 3 | 3 |
| 2004 | 37.81 | 1.06 | GBIF | 12.07 | 81.59 | 24.63 | 22.93 | 122 | 1 | 112.20 | 191 | 5 | 3 |
| 2005 | 36.64 | 0.70 | GBIF | 14.99 | 82.83 | 18.30 | 17.63 | 119 | 18 | 51.85 | 264 | 183 | 6 |
| 2006 | 37.82 | 1.17 | GBIF | 12.36 | 82.39 | 24.77 | 23.10 | 123 | 1 | 112.42 | 192 | 5 | 3 |
| 2007 | 37.75 | 1.07 | GBIF | 12.12 | 81.87 | 24.73 | 23.05 | 116 | 1 | 110.15 | 183 | 5 | 3 |
| 2008 | 36.63 | 0.23 | GBIF | 15.77 | 80.08 | 18.13 | 17.23 | 109 | 24 | 43.40 | 219 | 188 | 5 |
| 2009 | 36.62 | 0.71 | GBIF | 14.88 | 82.69 | 18.23 | 17.60 | 117 | 18 | 51.50 | 263 | 187 | 8 |
| 2010 | 36.79 | 0.33 | GBIF | 15.24 | 80.22 | 18.37 | 17.43 | 135 | 18 | 53.50 | 280 | 175 | 3 |
| 2011 | 37.81 | 1.16 | GBIF | 12.32 | 82.11 | 24.78 | 23.12 | 121 | 1 | 112.36 | 190 | 5 | 3 |
| 2012 | 36.74 | 0.32 | GBIF | 15.51 | 80.35 | 18.37 | 17.38 | 129 | 18 | 52.34 | 263 | 172 | 5 |
| 2013 | 37.83 | 1.17 | GBIF | 12.38 | 81.95 | 24.93 | 23.28 | 122 | 1 | 113.94 | 189 | 4 | 3 |
| 2014 | 36.58 | 0.74 | GBIF | 14.63 | 82.16 | 18.07 | 17.47 | 113 | 18 | 50.54 | 254 | 197 | 6 |
| 2015 | 38.66 | 2.55 | GBIF | 9.47 | 69.61 | 27.03 | 24.82 | 103 | 0 | 118.20 | 53 | 2 | 2 |
| 2016 | 37.77 | 1.23 | GBIF | 11.89 | 80.90 | 24.83 | 23.22 | 117 | 1 | 111.35 | 185 | 5 | 3 |
| 2017 | 36.60 | 0.74 | GBIF | 14.68 | 82.44 | 18.12 | 17.48 | 115 | 19 | 50.78 | 255 | 192 | 14 |
| 2018 | 37.83 | 1.10 | GBIF | 12.09 | 82.26 | 24.80 | 23.10 | 121 | 1 | 112.88 | 190 | 5 | 2 |
| 2019 | 37.91 | 1.08 | GBIF | 12.09 | 81.70 | 24.32 | 23.53 | 116 | 1 | 115.59 | 177 | 3 | 1 |
| 2020 | 36.65 | 0.40 | GBIF | 15.47 | 80.98 | 18.42 | 17.93 | 118 | 19 | 48.52 | 253 | 179 | 13 |
| 2021 | 36.84 | 0.67 | GBIF | 14.48 | 84.16 | 19.02 | 17.97 | 150 | 19 | 63.25 | 313 | 130 | 13 |
| 2022 | 37.89 | 1.14 | GBIF | 12.11 | 82.37 | 25.23 | 23.58 | 114 | 1 | 114.51 | 176 | 3 | 3 |
| 2023 | 36.62 | 0.69 | GBIF | 15.00 | 82.42 | 18.32 | 17.72 | 122 | 18 | 52.16 | 267 | 188 | 7 |
| 2024 | 36.70 | 0.29 | GBIF | 15.63 | 80.54 | 18.30 | 17.37 | 118 | 20 | 47.87 | 243 | 177 | 6 |
| 2025 | 36.64 | 0.68 | GBIF | 15.03 | 82.60 | 18.25 | 17.62 | 118 | 18 | 51.56 | 258 | 184 | 6 |
| 2026 | 37.87 | 1.06 | GBIF | 12.15 | 82.09 | 24.07 | 23.33 | 121 | 1 | 114.68 | 184 | 5 | 1 |
| 2027 | 37.81 | 1.08 | GBIF | 12.09 | 82.26 | 24.77 | 23.10 | 120 | 1 | 112.70 | 189 | 5 | 2 |
| 2028 | 38.03 | 1.14 | GBIF | 11.53 | 80.65 | 24.68 | 23.83 | 112 | 0 | 119.02 | 166 | 2 | 2 |
| 2029 | 37.76 | 1.18 | GBIF | 12.13 | 81.98 | 25.40 | 23.85 | 112 | 1 | 111.04 | 175 | 6 | 3 |
| 2030 | 36.55 | 0.71 | GBIF | 14.39 | 81.77 | 17.85 | 17.37 | 110 | 19 | 49.69 | 245 | 207 | 14 |
| 2031 | 36.85 | 0.23 | GBIF | 14.96 | 79.14 | 18.07 | 17.07 | 138 | 18 | 53.55 | 287 | 169 | 3 |
| 2032 | 37.88 | 1.11 | GBIF | 12.11 | 82.37 | 24.10 | 23.38 | 118 | 1 | 115.08 | 182 | 3 | 2 |
| 2033 | 36.85 | 0.47 | GBIF | 14.83 | 82.36 | 19.55 | 18.60 | 182 | 21 | 66.27 | 372 | 152 | 6 |
| 2034 | 36.61 | 0.73 | GBIF | 14.73 | 82.31 | 18.15 | 17.52 | 116 | 18 | 51.44 | 262 | 189 | 10 |
| 2035 | 36.86 | 0.47 | GBIF | 14.88 | 82.64 | 19.45 | 18.48 | 175 | 20 | 65.79 | 355 | 151 | 6 |
| 2036 | 37.89 | 1.11 | GBIF | 12.07 | 82.09 | 24.20 | 23.45 | 117 | 1 | 114.79 | 179 | 3 | 2 |
| 2037 | 37.99 | 1.14 | GBIF | 11.66 | 80.96 | 24.58 | 23.75 | 112 | 0 | 118.39 | 169 | 2 | 2 |
| 2038 | 36.89 | 0.63 | GBIF | 14.12 | 83.53 | 19.87 | 18.85 | 200 | 23 | 74.36 | 411 | 113 | 7 |
| 2039 | 36.95 | 0.36 | GBIF | 14.65 | 81.84 | 19.18 | 18.30 | 169 | 20 | 64.61 | 256 | 143 | 6 |
| 2040 | 36.59 | 0.70 | GBIF | 14.86 | 82.09 | 18.20 | 17.60 | 117 | 19 | 51.18 | 258 | 197 | 14 |
| 2041 | 36.93 | 0.42 | GBIF | 14.81 | 82.27 | 19.37 | 18.43 | 174 | 19 | 67.61 | 351 | 137 | 11 |
| 2042 | 36.86 | 0.23 | GBIF | 14.90 | 79.26 | 18.10 | 17.12 | 141 | 18 | 54.66 | 292 | 167 | 3 |
| 2043 | 36.96 | 0.29 | GBIF | 14.48 | 80.91 | 18.73 | 17.82 | 167 | 19 | 63.02 | 249 | 152 | 5 |
| 2044 | 36.84 | 0.42 | GBIF | 14.56 | 81.33 | 19.17 | 18.25 | 167 | 20 | 61.96 | 343 | 160 | 5 |
| 2045 | 37.77 | 1.18 | GBIF | 12.25 | 82.21 | 25.35 | 23.65 | 112 | 1 | 112.59 | 174 | 5 | 3 |
| 2046 | 36.77 | 0.34 | GBIF | 15.30 | 80.53 | 18.43 | 17.43 | 135 | 18 | 53.74 | 280 | 176 | 4 |
| 2047 | 36.64 | 0.19 | GBIF | 15.58 | 79.51 | 17.90 | 16.97 | 107 | 25 | 42.05 | 219 | 187 | 4 |
| 2048 | 36.74 | 0.34 | GBIF | 15.55 | 80.57 | 18.42 | 17.50 | 128 | 18 | 52.57 | 262 | 170 | 5 |
| 2049 | 36.92 | 0.23 | GBIF | 14.66 | 79.66 | 18.45 | 17.48 | 155 | 20 | 57.80 | 320 | 161 | 3 |

| 2050 | 36.92 | 0.26 | GBIF | 14.58 | 80.13 | 18.52 | 17.58 | 159 | 19 | 59.44 | 320 | 162 | 14 |
| --- | --- | --- | --- | --- | --- | --- | --- | --- | --- | --- | --- | --- | --- |
| 2051 | 36.57 | 0.74 | GBIF | 14.41 | 82.33 | 17.92 | 17.37 | 110 | 19 | 49.82 | 248 | 200 | 6 |
| 2052 | 37.97 | 2.14 | GBIF | 10.57 | 73.89 | 24.77 | 23.10 | 118 | 1 | 108.93 | 179 | 7 | 3 |
| 2053 | 36.92 | 0.37 | GBIF | 14.67 | 81.48 | 19.28 | 18.37 | 176 | 20 | 64.95 | 355 | 148 | 5 |
| 2054 | 36.60 | 0.70 | GBIF | 14.86 | 82.09 | 18.25 | 17.65 | 118 | 19 | 50.98 | 259 | 193 | 13 |
| 2055 | 36.67 | 0.37 | GBIF | 15.74 | 80.73 | 18.82 | 17.95 | 125 | 20 | 49.83 | 262 | 180 | 8 |
| 2056 | 36.77 | 0.30 | GBIF | 15.31 | 79.73 | 18.25 | 17.22 | 134 | 18 | 52.87 | 278 | 173 | 4 |
| 2057 | 36.99 | 0.36 | GBIF | 14.70 | 82.12 | 18.98 | 18.10 | 158 | 18 | 64.14 | 239 | 141 | 5 |
| 2058 | 38.06 | 1.17 | GBIF | 11.53 | 80.65 | 24.93 | 24.03 | 109 | 0 | 121.58 | 164 | 0 | 2 |
| 2059 | 36.69 | 0.23 | GBIF | 15.63 | 79.72 | 18.15 | 17.25 | 119 | 22 | 46.32 | 244 | 184 | 5 |
| 2060 | 36.82 | 0.49 | GBIF | 14.76 | 82.45 | 19.58 | 18.63 | 185 | 22 | 66.56 | 381 | 157 | 6 |
| 2061 | 37.85 | 0.62 | GBIF | 12.90 | 83.77 | 23.88 | 23.15 | 143 | 1 | 113.70 | 176 | 7 | 3 |
| 2062 | 37.94 | 2.15 | GBIF | 10.53 | 73.66 | 24.17 | 22.30 | 118 | 2 | 106.19 | 183 | 7 | 4 |
| 2063 | 37.45 | 0.58 | GBIF | 13.59 | 84.42 | 23.48 | 23.20 | 126 | 5 | 94.45 | 186 | 20 | 17 |
| 2064 | 37.90 | 0.70 | GBIF | 12.97 | 84.75 | 24.20 | 23.47 | 135 | 1 | 116.89 | 175 | 5 | 5 |
| 2065 | 37.92 | 2.16 | GBIF | 10.47 | 73.19 | 23.75 | 21.88 | 118 | 2 | 105.46 | 184 | 8 | 4 |
| 2066 | 36.84 | 0.52 | GBIF | 14.77 | 82.96 | 19.67 | 18.68 | 185 | 21 | 69.10 | 382 | 144 | 6 |
| 2067 | 36.70 | 0.25 | GBIF | 15.73 | 79.86 | 18.35 | 17.47 | 126 | 21 | 48.33 | 254 | 181 | 10 |
| 2068 | 36.87 | 0.46 | GBIF | 14.85 | 82.50 | 19.37 | 18.40 | 180 | 20 | 66.84 | 356 | 150 | 6 |
| 2069 | 36.94 | 0.33 | GBIF | 14.57 | 81.38 | 19.10 | 18.22 | 174 | 20 | 64.13 | 263 | 149 | 4 |
| 2070 | 36.85 | 0.33 | GBIF | 14.93 | 80.72 | 18.27 | 17.30 | 129 | 16 | 54.69 | 266 | 166 | 4 |
| 2071 | 37.92 | 0.66 | GBIF | 13.11 | 84.57 | 24.15 | 23.37 | 142 | 1 | 116.85 | 181 | 5 | 6 |
| 2072 | 37.95 | 2.15 | GBIF | 10.48 | 73.31 | 24.15 | 22.30 | 123 | 2 | 106.71 | 189 | 8 | 4 |
| 2073 | 37.01 | 0.29 | GBIF | 14.30 | 81.25 | 18.33 | 17.45 | 145 | 16 | 60.78 | 218 | 150 | 5 |
| 2074 | 37.01 | 0.39 | GBIF | 14.68 | 82.49 | 19.05 | 18.15 | 160 | 18 | 65.61 | 241 | 135 | 6 |
| 2075 | 36.95 | 0.32 | GBIF | 14.55 | 81.28 | 19.03 | 18.12 | 169 | 19 | 64.18 | 341 | 145 | 5 |
| 2076 | 37.87 | 0.68 | GBIF | 12.99 | 84.36 | 24.12 | 23.38 | 136 | 1 | 115.48 | 169 | 5 | 6 |
| 2077 | 37.42 | 0.54 | GBIF | 13.80 | 84.66 | 23.03 | 22.72 | 143 | 7 | 91.35 | 229 | 23 | 6 |
| 2078 | 36.93 | 0.40 | GBIF | 14.76 | 81.99 | 19.40 | 18.47 | 179 | 20 | 67.52 | 358 | 140 | 6 |
| 2079 | 37.92 | 2.12 | GBIF | 10.67 | 74.07 | 24.52 | 22.65 | 113 | 1 | 108.14 | 175 | 5 | 3 |
| 2080 | 37.87 | 0.70 | GBIF | 12.94 | 84.04 | 24.13 | 23.37 | 135 | 1 | 116.02 | 175 | 5 | 4 |
| 2081 | 37.55 | 0.52 | GBIF | 13.43 | 82.87 | 23.32 | 22.93 | 140 | 4 | 97.82 | 195 | 16 | 6 |
| 2082 | 37.95 | 2.21 | GBIF | 9.50 | 71.43 | 23.10 | 21.07 | 138 | 3 | 104.64 | 211 | 11 | 7 |
| 2083 | 37.90 | 0.69 | GBIF | 13.07 | 84.30 | 24.25 | 23.45 | 136 | 1 | 117.24 | 172 | 5 | 4 |
| 2084 | 37.01 | 0.37 | GBIF | 14.62 | 82.58 | 18.98 | 18.08 | 157 | 18 | 65.01 | 238 | 135 | 6 |
| 2085 | 37.46 | 0.51 | GBIF | 13.77 | 83.99 | 23.12 | 22.88 | 146 | 7 | 91.86 | 224 | 230 | 6 |
| 2086 | 36.96 | 0.35 | GBIF | 14.62 | 81.66 | 19.08 | 18.20 | 170 | 19 | 65.27 | 255 | 144 | 5 |
| 2087 | 37.95 | 2.13 | GBIF | 10.63 | 74.30 | 24.47 | 22.82 | 120 | 1 | 108.61 | 183 | 7 | 3 |
| 2088 | 37.91 | 0.66 | GBIF | 13.07 | 84.30 | 24.15 | 23.37 | 140 | 1 | 116.19 | 177 | 5 | 6 |
| 2089 | 37.45 | 0.37 | GBIF | 13.71 | 83.59 | 22.17 | 21.95 | 193 | 8 | 90.94 | 287 | 305 | 10 |
| 2090 | 37.55 | 0.49 | GBIF | 13.03 | 82.49 | 23.13 | 22.78 | 146 | 4 | 97.41 | 206 | 16 | 10 |
| 2091 | 37.93 | 0.72 | GBIF | 12.72 | 84.22 | 24.28 | 23.47 | 135 | 1 | 117.87 | 173 | 4 | 10 |
| 2092 | 37.41 | 0.38 | GBIF | 14.20 | 84.52 | 22.50 | 22.35 | 178 | 10 | 90.11 | 284 | 285 | 6 |
| 2093 | 37.95 | 2.17 | GBIF | 10.37 | 73.00 | 23.90 | 22.05 | 126 | 2 | 105.76 | 194 | 8 | 3 |
| 2094 | 37.91 | 2.14 | GBIF | 10.53 | 73.66 | 24.00 | 22.32 | 114 | 1 | 105.77 | 179 | 7 | 5 |
| 2095 | 37.95 | 2.19 | GBIF | 10.04 | 72.24 | 23.48 | 21.57 | 129 | 2 | 104.73 | 199 | 9 | 4 |
| 2096 | 37.40 | 0.32 | GBIF | 13.67 | 84.36 | 22.18 | 21.88 | 184 | 10 | 89.70 | 296 | 295 | 6 |
| 2097 | 37.43 | 0.43 | GBIF | 14.14 | 84.68 | 22.73 | 22.62 | 167 | 8 | 89.80 | 265 | 266 | 10 |
| 2098 | 37.88 | 0.70 | GBIF | 12.95 | 84.09 | 24.18 | 23.40 | 133 | 1 | 115.89 | 176 | 5 | 3 |
| 2099 | 37.56 | 0.57 | GBIF | 13.31 | 82.66 | 23.60 | 23.25 | 123 | 3 | 101.15 | 168 | 14 | 23 |

| 2100 | 37.38 | 0.36 | GBIF | 13.89 | 84.71 | 22.58 | 22.32 | 168 | 10 | 88.65 | 276 | 271 | 5 |
| --- | --- | --- | --- | --- | --- | --- | --- | --- | --- | --- | --- | --- | --- |
| 2101 | 37.87 | 0.64 | GBIF | 13.00 | 84.42 | 24.02 | 23.28 | 142 | 1 | 115.47 | 177 | 5 | 4 |
| 2102 | 37.85 | 0.64 | GBIF | 13.00 | 83.87 | 24.02 | 23.28 | 139 | 1 | 115.31 | 172 | 6 | 4 |
| 2103 | 37.37 | 0.34 | GBIF | 13.57 | 84.32 | 22.13 | 21.83 | 178 | 10 | 89.13 | 296 | 286 | 5 |
| 2104 | 37.38 | 0.35 | GBIF | 13.77 | 84.46 | 22.33 | 22.03 | 175 | 10 | 89.45 | 290 | 282 | 6 |
| 2105 | 37.46 | 0.30 | GBIF | 13.28 | 83.02 | 20.45 | 19.95 | 251 | 9 | 96.43 | 350 | 33 | 6 |
| 2106 | 37.33 | 0.49 | GBIF | 13.61 | 84.52 | 23.75 | 22.53 | 153 | 10 | 87.83 | 260 | 31 | 5 |
| 2107 | 37.60 | 0.55 | GBIF | 13.54 | 82.57 | 23.52 | 23.03 | 130 | 3 | 102.00 | 178 | 13 | 8 |
| 2108 | 37.38 | 0.37 | GBIF | 14.16 | 84.78 | 22.58 | 22.42 | 170 | 10 | 88.71 | 277 | 274 | 6 |
| 2109 | 37.58 | 0.54 | GBIF | 13.57 | 82.72 | 23.47 | 23.02 | 134 | 4 | 100.75 | 178 | 14 | 8 |
| 2110 | 37.33 | 0.47 | GBIF | 13.63 | 84.10 | 23.68 | 22.33 | 155 | 10 | 87.54 | 263 | 240 | 4 |
| 2111 | 37.31 | 0.46 | GBIF | 13.46 | 84.11 | 23.40 | 22.03 | 169 | 11 | 88.08 | 280 | 34 | 3 |
| 2112 | 37.35 | 0.41 | GBIF | 13.88 | 84.60 | 23.85 | 22.53 | 157 | 10 | 88.50 | 267 | 249 | 5 |
| 2113 | 37.38 | 0.32 | GBIF | 13.41 | 84.33 | 21.87 | 21.53 | 187 | 10 | 89.64 | 303 | 32 | 6 |
| 2114 | 37.37 | 0.41 | GBIF | 13.92 | 84.34 | 23.82 | 22.50 | 157 | 10 | 88.14 | 266 | 252 | 5 |
| 2115 | 37.35 | 0.43 | GBIF | 14.04 | 84.59 | 23.93 | 22.62 | 154 | 10 | 88.32 | 261 | 241 | 5 |
| 2116 | 37.32 | 0.46 | GBIF | 13.57 | 84.27 | 23.55 | 22.20 | 164 | 10 | 87.98 | 274 | 33 | 4 |
| 2117 | 37.35 | 0.33 | GBIF | 12.94 | 83.49 | 21.22 | 20.77 | 197 | 12 | 89.53 | 322 | 38 | 10 |
| 2118 | 37.37 | 0.39 | GBIF | 13.99 | 84.80 | 22.70 | 22.48 | 160 | 10 | 88.37 | 269 | 258 | 5 |
| 2119 | 37.43 | 0.39 | GBIF | 14.13 | 84.08 | 22.67 | 22.52 | 172 | 9 | 89.61 | 271 | 276 | 8 |
| 2120 | 37.39 | 0.34 | GBIF | 13.90 | 84.24 | 22.35 | 22.05 | 177 | 10 | 89.67 | 290 | 285 | 6 |
| 2121 | 37.32 | 0.42 | GBIF | 13.48 | 83.70 | 23.32 | 21.98 | 168 | 11 | 87.33 | 282 | 257 | 9 |
| 2122 | 37.38 | 0.39 | GBIF | 14.08 | 84.84 | 22.63 | 22.45 | 165 | 10 | 88.59 | 277 | 266 | 6 |
| 2123 | 37.87 | -1.58 | GBIF | 10.97 | 71.21 | 23.18 | 20.83 | 212 | 1 | 110.81 | 250 | 11 | 14 |
| 2124 | 35.03 | -1.33 | GBIF | 12.60 | 81.29 | 20.23 | 18.70 | 168 | 35 | 39.71 | 297 | 159 | 3 |
| 2125 | 36.96 | 0.14 | GBIF | 15.17 | 78.99 | 17.83 | 16.88 | 127 | 19 | 51.63 | 273 | 164 | 3 |
| 2126 | 37.00 | 0.02 | GBIF | 14.67 | 78.85 | 17.28 | 16.33 | 126 | 26 | 50.56 | 283 | 137 | 12 |
| 2127 | 36.93 | 0.20 | GBIF | 14.82 | 79.23 | 18.18 | 17.20 | 145 | 19 | 55.57 | 307 | 163 | 3 |
| 2128 | 36.84 | 0.09 | GBIF | 15.05 | 78.39 | 17.85 | 16.75 | 128 | 23 | 47.04 | 273 | 161 | 12 |
| 2129 | 36.98 | 0.18 | GBIF | 14.68 | 79.37 | 17.90 | 16.97 | 137 | 18 | 55.37 | 287 | 165 | 3 |
| 2130 | 37.00 | 0.08 | GBIF | 14.66 | 78.81 | 17.37 | 16.42 | 124 | 21 | 50.25 | 271 | 156 | 13 |
| 2131 | 34.83 | -1.21 | GBIF | 11.68 | 81.64 | 19.55 | 18.10 | 218 | 51 | 37.14 | 335 | 225 | 13 |
| 2132 | 35.21 | -1.70 | GBIF | 12.43 | 81.26 | 20.30 | 18.50 | 153 | 21 | 52.61 | 366 | 93 | 11 |
| 2133 | 35.33 | -1.65 | GBIF | 12.38 | 81.41 | 19.75 | 17.62 | 168 | 22 | 54.97 | 388 | 91 | 5 |
| 2134 | 35.36 | -1.61 | GBIF | 12.27 | 80.70 | 19.45 | 17.23 | 172 | 23 | 54.77 | 393 | 95 | 8 |
| 2135 | 35.30 | -1.64 | GBIF | 12.42 | 81.15 | 20.03 | 18.10 | 172 | 23 | 54.47 | 390 | 96 | 6 |
| 2136 | 35.35 | -1.76 | GBIF | 11.86 | 80.12 | 17.80 | 15.68 | 192 | 23 | 59.02 | 428 | 90 | 5 |
| 2137 | 35.25 | -1.72 | GBIF | 11.90 | 80.41 | 17.78 | 15.77 | 196 | 26 | 55.47 | 433 | 103 | 5 |
| 2138 | 38.43 | -3.29 | GBIF | 9.52 | 67.98 | 22.95 | 20.43 | 140 | 8 | 80.82 | 279 | 34 | 14 |
| 2139 | 35.36 | -1.27 | GBIF | 12.59 | 82.84 | 18.75 | 17.05 | 174 | 28 | 48.48 | 389 | 124 | 3 |
| 2140 | 35.28 | -1.29 | GBIF | 13.01 | 82.33 | 19.80 | 17.88 | 163 | 28 | 46.64 | 376 | 126 | 4 |
| 2141 | 36.92 | -0.41 | GBIF | 12.41 | 76.59 | 17.78 | 15.85 | 171 | 33 | 62.21 | 391 | 109 | 14 |
| 2142 | 37.78 | 1.61 | GBIF | 12.15 | 80.46 | 25.87 | 24.05 | 113 | 1 | 111.26 | 174 | 4 | 4 |
| 2143 | 36.87 | 0.29 | GBIF | 14.77 | 80.25 | 18.10 | 17.13 | 129 | 16 | 53.96 | 263 | 168 | 4 |
| 2144 | 36.92 | 0.42 | GBIF | 14.79 | 82.18 | 19.55 | 18.57 | 186 | 21 | 68.56 | 373 | 141 | 6 |
| 2145 | 36.83 | 0.23 | GBIF | 15.02 | 79.04 | 17.98 | 16.95 | 127 | 17 | 51.51 | 270 | 172 | 3 |
| 2146 | 36.94 | 0.39 | GBIF | 14.73 | 81.81 | 19.07 | 18.17 | 159 | 18 | 64.04 | 239 | 143 | 11 |
| 2147 | 37.00 | 0.23 | GBIF | 14.33 | 79.63 | 18.12 | 17.25 | 145 | 18 | 58.32 | 222 | 157 | 6 |
| 2148 | 37.85 | 0.71 | GBIF | 12.77 | 83.99 | 24.15 | 23.38 | 131 | 1 | 116.40 | 173 | 5 | 6 |
| 2149 | 36.93 | -0.07 | GBIF | 14.36 | 78.89 | 17.55 | 16.38 | 126 | 30 | 49.35 | 286 | 205 | 17 |

| 2150 | 36.96 | -0.06 | GBIF | 14.44 | 78.92 | 17.50 | 16.28 | 126 | 31 | 49.78 | 287 | 202 | 22 |
| --- | --- | --- | --- | --- | --- | --- | --- | --- | --- | --- | --- | --- | --- |
| 2151 | 36.89 | -0.08 | GBIF | 14.38 | 79.42 | 17.48 | 16.30 | 123 | 31 | 46.58 | 280 | 204 | 4 |
| 2152 | 36.82 | 0.13 | GBIF | 15.22 | 78.44 | 17.95 | 16.93 | 130 | 22 | 48.12 | 273 | 166 | 4 |
| 2153 | 37.19 | -2.70 | GBIF | 13.18 | 73.65 | 22.68 | 20.00 | 146 | 5 | 81.39 | 177 | 24 | 5 |
| 2154 | 37.01 | 0.19 | GBIF | 14.52 | 79.76 | 17.93 | 17.02 | 135 | 17 | 56.12 | 283 | 161 | 8 |
| 2155 | 34.97 | -1.32 | GBIF | 12.45 | 81.91 | 20.18 | 18.60 | 186 | 39 | 39.05 | 310 | 177 | 18 |
| 2156 | 34.87 | -1.46 | GBIF | 12.60 | 82.35 | 20.92 | 19.43 | 175 | 34 | 40.76 | 306 | 156 | 7 |
| 2157 | 36.70 | 1.08 | GBIF | 13.79 | 80.18 | 17.00 | 16.42 | 97 | 18 | 45.57 | 213 | 176 | 22 |
| 2158 | 37.71 | -3.23 | GBIF | 11.96 | 69.53 | 22.83 | 20.15 | 204 | 10 | 90.72 | 202 | 35 | 13 |
| 2159 | 38.66 | -3.80 | GBIF | 9.56 | 67.31 | 23.18 | 20.67 | 127 | 17 | 63.12 | 178 | 76 | 5 |
| 2160 | 38.75 | -3.72 | GBIF | 10.17 | 68.23 | 24.23 | 21.75 | 117 | 18 | 59.53 | 165 | 80 | 7 |
| 2161 | 38.64 | -3.76 | GBIF | 9.95 | 67.23 | 23.43 | 20.98 | 125 | 15 | 64.47 | 175 | 72 | 8 |
| 2162 | 38.72 | -3.67 | GBIF | 10.46 | 68.80 | 23.33 | 20.88 | 131 | 18 | 63.24 | 180 | 81 | 10 |
| 2163 | 38.62 | -3.70 | GBIF | 9.94 | 66.72 | 23.55 | 21.07 | 124 | 13 | 67.02 | 169 | 65 | 7 |
| 2164 | 38.70 | -3.70 | GBIF | 10.77 | 69.07 | 24.18 | 21.63 | 121 | 17 | 61.34 | 170 | 77 | 11 |
| 2165 | 38.61 | -3.62 | GBIF | 9.95 | 67.69 | 23.82 | 21.33 | 123 | 13 | 66.79 | 168 | 59 | 7 |
| 2166 | 38.64 | -3.60 | GBIF | 9.86 | 67.99 | 23.88 | 21.43 | 125 | 16 | 65.52 | 169 | 73 | 7 |
| 2167 | 36.76 | -0.37 | GBIF | 11.13 | 80.04 | 12.83 | 11.93 | 207 | 40 | 50.72 | 535 | 218 | 4 |
| 2168 | 36.83 | -0.34 | GBIF | 12.14 | 78.84 | 15.50 | 13.72 | 204 | 47 | 52.79 | 451 | 179 | 14 |
| 2169 | 35.34 | -1.49 | GBIF | 12.63 | 80.93 | 20.40 | 18.42 | 156 | 24 | 50.21 | 361 | 104 | 5 |
| 2170 | 36.38 | -0.75 | GBIF | 12.31 | 77.90 | 17.85 | 15.70 | 124 | 29 | 51.68 | 217 | 105 | 6 |
| 2171 | 36.10 | -0.42 | GBIF | 14.52 | 81.15 | 18.37 | 17.87 | 125 | 26 | 41.62 | 217 | 194 | 6 |
| 2172 | 38.33 | -3.05 | GBIF | 10.44 | 69.61 | 25.02 | 22.05 | 177 | 15 | 77.52 | 309 | 59 | 1 |
| 2173 | 36.86 | 0.24 | GBIF | 14.88 | 79.55 | 18.00 | 17.00 | 134 | 17 | 53.57 | 277 | 168 | 2 |
| 2174 | 36.19 | -0.32 | GBIF | 14.88 | 80.84 | 17.05 | 17.20 | 124 | 24 | 42.13 | 212 | 217 | 27 |
| 2175 | 38.61 | -3.91 | GBIF | 10.28 | 68.10 | 23.98 | 21.50 | 117 | 14 | 62.97 | 169 | 67 | 5 |
| 2176 | 34.94 | -1.50 | GBIF | 12.57 | 81.60 | 20.72 | 19.15 | 172 | 30 | 42.79 | 304 | 142 | 13 |
| 2177 | 34.80 | -1.41 | GBIF | 11.96 | 80.80 | 20.70 | 19.17 | 186 | 38 | 39.56 | 314 | 170 | 8 |
| 2178 | 36.98 | 0.03 | GBIF | 14.59 | 78.87 | 17.35 | 16.33 | 126 | 25 | 49.77 | 280 | 144 | 12 |
| 2179 | 37.31 | -2.62 | GBIF | 12.62 | 72.93 | 22.70 | 20.03 | 144 | 4 | 84.03 | 177 | 21 | 12 |
| 2180 | 37.88 | -3.26 | GBIF | 11.52 | 68.60 | 22.88 | 20.05 | 188 | 10 | 87.53 | 189 | 37 | 5 |
| 2181 | 38.38 | 0.13 | GBIF | 10.45 | 76.28 | 25.05 | 23.42 | 148 | 0 | 127.03 | 162 | 0 | 2 |
| 2182 | 36.98 | 0.07 | GBIF | 14.57 | 78.36 | 17.37 | 16.40 | 121 | 22 | 49.13 | 268 | 153 | 13 |
| 2183 | 37.49 | 0.63 | GBIF | 13.05 | 83.65 | 23.10 | 22.60 | 132 | 5 | 96.96 | 202 | 18 | 6 |
| 2184 | 37.80 | -3.49 | GBIF | 11.33 | 69.05 | 23.87 | 21.17 | 148 | 6 | 84.54 | 178 | 27 | 4 |
| 2185 | 38.45 | -2.97 | GBIF | 10.69 | 70.34 | 25.53 | 22.50 | 214 | 22 | 75.04 | 368 | 85 | 18 |
| 2186 | 37.51 | 0.60 | GBIF | 13.23 | 83.18 | 23.23 | 22.80 | 132 | 5 | 97.07 | 191 | 17 | 8 |
| 2187 | 38.60 | -2.82 | GBIF | 10.34 | 69.41 | 25.17 | 22.70 | 194 | 21 | 77.15 | 339 | 74 | 4 |
| 2188 | 36.39 | 0.43 | GBIF | 14.92 | 81.51 | 17.48 | 17.10 | 115 | 25 | 45.90 | 250 | 234 | 18 |
| 2189 | 37.50 | 0.73 | GBIF | 12.41 | 83.84 | 23.55 | 22.07 | 135 | 4 | 99.02 | 215 | 15 | 2 |
| 2190 | 39.22 | -1.18 | GBIF | 9.83 | 70.74 | 27.22 | 24.90 | 149 | 3 | 105.30 | 158 | 20 | 1 |
| 2191 | 36.30 | -0.89 | GBIF | 13.82 | 76.38 | 17.00 | 14.72 | 143 | 32 | 53.09 | 250 | 116 | 8 |
| 2192 | 36.89 | 0.31 | GBIF | 14.70 | 80.77 | 18.48 | 17.52 | 141 | 17 | 56.37 | 287 | 164 | 11 |
| 2193 | 36.84 | 0.76 | GBIF | 14.92 | 84.27 | 18.85 | 17.75 | 141 | 18 | 61.07 | 292 | 128 | 6 |
| 2194 | 36.84 | 0.14 | GBIF | 15.27 | 78.29 | 18.00 | 16.98 | 134 | 21 | 49.75 | 280 | 167 | 4 |
| 2195 | 36.90 | 0.23 | GBIF | 14.78 | 79.48 | 18.37 | 17.38 | 150 | 19 | 56.08 | 312 | 165 | 10 |
| 2196 | 36.88 | 0.08 | GBIF | 14.86 | 78.20 | 17.68 | 16.62 | 128 | 23 | 47.54 | 272 | 165 | 4 |
| 2197 | 36.91 | 0.28 | GBIF | 14.59 | 80.62 | 18.85 | 17.92 | 161 | 20 | 59.31 | 326 | 158 | 12 |
| 2198 | 36.82 | 0.37 | GBIF | 14.96 | 81.30 | 18.45 | 17.52 | 129 | 16 | 55.30 | 268 | 166 | 3 |
| 2199 | 36.97 | 0.23 | GBIF | 14.43 | 79.74 | 18.12 | 17.20 | 145 | 18 | 57.34 | 297 | 161 | 7 |

| 2200 | 36.98 | 0.39 | GBIF | 14.74 | 82.36 | 19.27 | 18.40 | 170 | 19 | 66.83 | 258 | 135 | 6 |
| --- | --- | --- | --- | --- | --- | --- | --- | --- | --- | --- | --- | --- | --- |
| 2201 | 36.88 | 0.33 | GBIF | 14.77 | 80.74 | 18.43 | 17.47 | 134 | 16 | 55.41 | 277 | 165 | 7 |
| 2202 | 36.80 | 0.35 | GBIF | 15.14 | 80.54 | 18.35 | 17.40 | 128 | 17 | 53.44 | 269 | 171 | 3 |
| 2203 | 36.99 | 0.32 | GBIF | 14.56 | 81.79 | 18.65 | 17.75 | 153 | 17 | 62.05 | 305 | 148 | 5 |
| 2204 | 35.03 | -1.24 | GBIF | 12.47 | 82.02 | 20.25 | 18.77 | 185 | 39 | 39.64 | 300 | 179 | 11 |
| 2205 | 37.42 | 0.73 | GBIF | 12.78 | 84.66 | 23.32 | 21.90 | 144 | 6 | 95.75 | 242 | 19 | 2 |
| 2206 | 37.83 | 2.38 | GBIF | 11.52 | 76.27 | 25.43 | 23.30 | 87 | 1 | 104.81 | 65 | 5 | 4 |
| 2207 | 38.33 | -3.14 | GBIF | 10.36 | 69.52 | 24.08 | 21.20 | 159 | 12 | 79.54 | 294 | 45 | 1 |
| 2208 | 37.00 | -2.50 | GBIF | 11.13 | 70.02 | 22.30 | 19.70 | 122 | 1 | 81.61 | 180 | 13 | 12 |
| 2209 | 34.85 | -1.41 | GBIF | 11.96 | 80.80 | 20.53 | 19.00 | 185 | 39 | 39.68 | 314 | 169 | 7 |
| 2210 | 35.24 | -1.58 | GBIF | 12.64 | 81.04 | 20.37 | 18.48 | 161 | 24 | 50.76 | 370 | 107 | 12 |
| 2211 | 37.28 | -2.68 | GBIF | 12.86 | 73.06 | 22.53 | 19.82 | 147 | 5 | 82.91 | 178 | 24 | 8 |
| 2212 | 34.97 | -1.45 | GBIF | 12.52 | 81.81 | 20.17 | 18.72 | 172 | 33 | 40.85 | 311 | 152 | 14 |
| 2213 | 38.38 | -0.05 | GBIF | 10.40 | 74.82 | 25.62 | 23.73 | 141 | 0 | 128.57 | 150 | 0 | 3 |
| 2214 | 39.95 | -1.10 | GBIF | 8.87 | 67.68 | 27.95 | 25.58 | 100 | 6 | 94.85 | 154 | 28 | 15 |
| 2215 | 35.13 | -1.39 | GBIF | 12.93 | 81.86 | 20.60 | 19.05 | 158 | 28 | 43.29 | 368 | 136 | 7 |
| 2216 | 36.95 | -0.29 | GBIF | 13.09 | 77.01 | 17.70 | 16.15 | 145 | 26 | 57.03 | 337 | 97 | 8 |
| 2217 | 37.25 | -2.68 | GBIF | 12.99 | 73.40 | 22.62 | 19.88 | 142 | 4 | 83.04 | 174 | 22 | 4 |
| 2218 | 37.27 | -2.66 | GBIF | 12.66 | 72.75 | 22.53 | 19.77 | 141 | 4 | 83.75 | 175 | 21 | 12 |
| 2219 | 35.48 | -1.48 | GBIF | 12.74 | 81.16 | 19.82 | 17.72 | 152 | 21 | 55.05 | 350 | 85 | 4 |
| 2220 | 35.32 | -1.51 | GBIF | 12.51 | 81.22 | 19.95 | 18.28 | 171 | 26 | 49.91 | 384 | 114 | 7 |
| 2221 | 30.80 | 0.20 | GBIF | 10.96 | 83.65 | 21.32 | 21.78 | 127 | 35 | 42.84 | 167 | 161 | 6 |
| 2222 | 30.40 | 0.50 | GBIF | 10.41 | 86.74 | 19.72 | 20.05 | 199 | 48 | 46.71 | 329 | 216 | 6 |
| 2223 | 33.80 | 3.60 | GBIF | 12.95 | 75.73 | 21.10 | 23.85 | 132 | 8 | 60.10 | 86 | 363 | 3 |
| 2224 | 31.50 | 2.70 | GBIF | 11.18 | 58.82 | 22.87 | 25.92 | 141 | 15 | 49.69 | 103 | 390 | 0 |
| 2225 | 31.80 | 2.20 | GBIF | 11.13 | 73.68 | 23.30 | 25.22 | 148 | 23 | 44.76 | 145 | 420 | 0 |
| 2226 | 31.90 | 3.70 | GBIF | 11.89 | 71.21 | 22.95 | 26.53 | 170 | 9 | 59.96 | 84 | 477 | 13 |
| 2227 | 29.90 | 0.30 | GBIF | 9.73 | 81.11 | 6.23 | 5.43 | 231 | 96 | 28.69 | 416 | 356 | 5 |
| 2228 | 30.00 | 0.40 | GBIF | 9.81 | 84.55 | 12.43 | 12.28 | 202 | 80 | 30.60 | 444 | 335 | 6 |
| 2229 | 31.70 | 0.90 | GBIF | 10.18 | 80.75 | 21.47 | 22.37 | 161 | 32 | 43.71 | 175 | 242 | 8 |
| 2230 | 30.00 | 0.80 | GBIF | 10.05 | 88.16 | 22.40 | 22.87 | 136 | 45 | 29.34 | 298 | 316 | 7 |
| 2231 | 30.40 | 1.00 | GBIF | 11.03 | 83.59 | 24.82 | 24.80 | 147 | 31 | 37.50 | 169 | 305 | 5 |
| 2232 | 30.14 | -0.02 | GBIF | 11.14 | 88.43 | 23.22 | 23.23 | 120 | 27 | 45.10 | 243 | 277 |  |
| 2233 | 30.05 | -0.10 | GBIF | 11.46 | 87.47 | 23.07 | 23.10 | 119 | 24 | 45.48 | 244 | 284 | 9 |
| 2234 | 31.49 | 2.55 | GBIF | 11.01 | 60.15 | 22.65 | 25.58 | 134 | 16 | 48.61 | 105 | 380 | 4 |
| 2235 | 31.64 | 2.30 | GBIF | 10.98 | 70.35 | 23.97 | 26.10 | 144 | 19 | 45.58 | 135 | 392 | 0 |
| 2236 | 29.81 | -0.32 | GBIF | 9.04 | 92.26 | 22.98 | 22.85 | 106 | 26 | 42.53 | 159 | 147 |  |
| 2237 | 32.45 | 1.11 | GBIF | 11.24 | 82.06 | 22.80 | 22.97 | 171 | 31 | 42.22 | 185 | 264 | 18 |
| 2238 | 29.84 | -0.33 | GBIF | 9.68 | 91.27 | 22.88 | 22.73 | 108 | 25 | 43.18 | 159 | 150 |  |
| 2239 | 31.82 | 2.25 | GBIF | 11.45 | 72.01 | 23.33 | 25.38 | 149 | 23 | 44.96 | 144 | 426 | 7 |
| 2240 | 29.93 | -0.31 | GBIF | 10.46 | 89.39 | 22.50 | 22.33 | 118 | 24 | 44.98 | 164 | 157 | 4 |
| 2241 | 31.71 | 2.22 | GBIF | 10.77 | 72.75 | 23.42 | 25.38 | 146 | 23 | 44.58 | 143 | 410 | 2 |
| 2242 | 29.65 | -0.79 | GBIF | 11.38 | 91.07 | 21.85 | 21.75 | 121 | 33 | 34.85 | 199 | 159 | 14 |
| 2243 | 31.51 | 2.21 | GBIF | 10.96 | 73.55 | 24.13 | 26.07 | 143 | 20 | 45.73 | 139 | 390 | 13 |
| 2244 | 30.08 | -0.14 | GBIF | 10.98 | 88.58 | 22.90 | 22.92 | 119 | 26 | 44.87 | 159 | 287 | 10 |
| 2245 | 31.65 | 2.32 | GBIF | 11.14 | 69.64 | 23.77 | 25.97 | 145 | 19 | 45.57 | 136 | 400 | 7 |
| 2246 | 31.57 | 2.36 | GBIF | 11.34 | 67.51 | 23.60 | 25.97 | 145 | 20 | 45.74 | 140 | 399 | 11 |
| 2247 | 31.51 | 2.35 | GBIF | 11.29 | 66.81 | 23.72 | 26.13 | 136 | 21 | 45.46 | 131 | 380 | 8 |
| 2248 | 29.86 | -0.02 | GBIF | 11.77 | 87.16 | 22.63 | 22.90 | 145 | 39 | 42.59 | 291 | 326 | 8 |
| 2249 | 31.76 | 2.03 | GBIF | 10.72 | 78.80 | 23.37 | 24.93 | 149 | 25 | 43.89 | 150 | 364 | 1 |

| 2250 | 29.90 | -0.20 | GBIF | 10.00 | 90.09 | 22.95 | 22.88 | 118 | 25 | 44.95 | 161 | 289 | 10 |
| --- | --- | --- | --- | --- | --- | --- | --- | --- | --- | --- | --- | --- | --- |
| 2251 | 29.94 | -0.27 | GBIF | 10.89 | 87.84 | 22.53 | 22.43 | 117 | 26 | 44.67 | 165 | 302 | 8 |
| 2252 | 31.69 | 2.31 | GBIF | 11.12 | 70.36 | 23.37 | 25.55 | 147 | 21 | 45.34 | 137 | 409 | 11 |
| 2253 | 30.02 | -0.16 | GBIF | 10.81 | 88.59 | 22.92 | 22.90 | 113 | 23 | 45.51 | 238 | 282 | 8 |
| 2254 | 29.80 | -0.16 | GBIF | 9.46 | 91.83 | 23.03 | 22.87 | 109 | 26 | 44.30 | 231 | 157 |  |
| 2255 | 31.49 | 2.39 | GBIF | 11.05 | 64.62 | 23.58 | 26.10 | 126 | 19 | 45.66 | 116 | 362 | 10 |
| 2256 | 31.56 | 2.35 | GBIF | 11.34 | 67.91 | 23.67 | 26.02 | 143 | 20 | 45.52 | 138 | 394 | 15 |
| 2257 | 29.87 | -0.14 | GBIF | 10.68 | 89.03 | 23.13 | 23.10 | 110 | 23 | 46.04 | 228 | 268 | 15 |
| 2258 | 29.97 | -0.04 | GBIF | 11.01 | 86.68 | 22.53 | 22.57 | 128 | 30 | 43.56 | 267 | 304 | 4 |
| 2259 | 30.08 | -0.02 | GBIF | 11.81 | 86.83 | 23.17 | 23.28 | 123 | 28 | 45.03 | 153 | 282 | 4 |
| 2260 | 31.48 | 2.26 | GBIF | 10.89 | 70.27 | 24.05 | 26.20 | 139 | 20 | 45.83 | 135 | 384 | 9 |
| 2261 | 31.59 | 2.21 | GBIF | 10.87 | 71.96 | 23.65 | 25.68 | 144 | 21 | 45.23 | 138 | 397 | 16 |
| 2262 | 31.56 | 2.32 | GBIF | 11.22 | 70.10 | 23.88 | 26.07 | 150 | 20 | 46.37 | 147 | 408 | 9 |
| 2263 | 31.47 | 2.37 | GBIF | 10.69 | 64.80 | 23.72 | 26.18 | 123 | 18 | 46.32 | 201 | 355 |  |
| 2264 | 31.67 | 2.10 | GBIF | 10.80 | 76.06 | 23.95 | 25.72 | 145 | 26 | 43.47 | 150 | 400 | 1 |
| 2265 | 31.58 | 2.34 | GBIF | 11.27 | 68.33 | 23.67 | 25.95 | 146 | 20 | 45.90 | 138 | 398 | 11 |
| 2266 | 29.80 | -0.38 | GBIF | 10.31 | 90.42 | 22.87 | 22.68 | 109 | 25 | 42.75 | 160 | 150 |  |
| 2267 | 29.94 | -0.29 | GBIF | 10.64 | 88.68 | 22.45 | 22.33 | 117 | 25 | 44.91 | 168 | 303 | 4 |
| 2268 | 30.18 | -0.03 | GBIF | 10.11 | 89.45 | 23.07 | 23.00 | 121 | 27 | 44.80 | 246 | 280 | 5 |
| 2269 | 30.17 | -0.12 | GBIF | 11.00 | 87.30 | 22.87 | 22.87 | 128 | 27 | 44.88 | 166 | 289 | 7 |
| 2270 | 31.49 | 2.40 | GBIF | 11.09 | 64.49 | 23.60 | 26.13 | 126 | 19 | 46.06 | 114 | 362 | 8 |
| 2271 | 29.85 | -0.24 | GBIF | 8.78 | 90.55 | 23.03 | 22.90 | 111 | 25 | 44.79 | 152 | 122 |  |
| 2272 | 31.59 | 2.24 | GBIF | 10.75 | 72.15 | 23.92 | 25.95 | 145 | 20 | 44.98 | 139 | 394 | 9 |
| 2273 | 31.55 | 2.40 | GBIF | 11.38 | 65.42 | 23.53 | 26.02 | 139 | 19 | 46.56 | 127 | 388 | 7 |
| 2274 | 31.79 | 2.30 | GBIF | 11.29 | 71.92 | 23.42 | 25.53 | 148 | 21 | 45.29 | 139 | 419 | 0 |
| 2275 | 31.43 | 2.36 | GBIF | 10.82 | 65.61 | 23.67 | 26.10 | 129 | 20 | 46.04 | 121 | 375 | 19 |
| 2276 | 31.59 | 2.26 | GBIF | 10.62 | 71.73 | 23.97 | 26.00 | 146 | 20 | 45.66 | 141 | 395 | 8 |
| 2277 | 31.55 | 2.29 | GBIF | 11.06 | 69.99 | 24.08 | 26.27 | 145 | 21 | 45.49 | 145 | 394 | 10 |
| 2278 | 31.33 | 1.82 | GBIF | 8.22 | 82.17 | 26.07 | 26.15 | 148 | 20 | 45.44 | 135 | 295 |  |
| 2279 | 31.73 | 1.96 | GBIF | 10.46 | 80.45 | 22.82 | 24.32 | 147 | 26 | 43.15 | 153 | 364 | 3 |
| 2280 | 31.58 | 2.31 | GBIF | 11.02 | 70.17 | 23.98 | 26.15 | 149 | 19 | 46.10 | 143 | 399 | 15 |
| 2281 | 29.89 | 0.41 | GBIF | 8.98 | 80.86 | 3.52 | 3.15 | 241 | 96 | 28.48 | 457 | 368 | 7 |
| 2282 | 32.50 | 1.25 | GBIF | 11.31 | 81.35 | 22.98 | 23.23 | 168 | 27 | 44.21 | 164 | 261 | 10 |
| 2283 | 30.10 | 0.27 | GBIF | 10.96 | 85.61 | 21.70 | 22.22 | 136 | 40 | 38.67 | 186 | 327 | 19 |
| 2284 | 31.73 | 1.99 | GBIF | 10.54 | 78.09 | 22.05 | 23.73 | 144 | 25 | 43.41 | 150 | 374 | 4 |
| 2285 | 30.33 | 1.02 | GBIF | 11.24 | 84.52 | 24.78 | 24.75 | 157 | 33 | 37.96 | 176 | 332 | 7 |
| 2286 | 31.58 | 2.29 | GBIF | 10.91 | 70.38 | 24.02 | 26.17 | 146 | 20 | 45.57 | 146 | 396 | 11 |
| 2287 | 31.47 | 1.73 | GBIF | 10.20 | 79.69 | 22.05 | 23.62 | 156 | 27 | 43.85 | 160 | 360 | 14 |
| 2288 | 31.58 | 1.65 | GBIF | 11.21 | 80.64 | 22.33 | 23.95 | 170 | 31 | 43.59 | 181 | 379 | 22 |
| 2289 | 31.00 | 1.13 | GBIF | 10.38 | 81.12 | 22.35 | 23.35 | 167 | 34 | 42.24 | 185 | 332 | 14 |
| 2290 | 31.67 | 1.93 | GBIF | 10.37 | 80.99 | 23.02 | 24.52 | 150 | 25 | 43.49 | 152 | 357 | 3 |
| 2291 | 31.61 | 2.29 | GBIF | 10.86 | 70.51 | 24.05 | 26.18 | 145 | 19 | 45.78 | 141 | 393 | 7 |
| 2292 | 31.72 | 2.08 | GBIF | 10.75 | 77.34 | 23.83 | 25.48 | 147 | 25 | 43.67 | 150 | 401 | 5 |
| 2293 | 31.41 | 1.93 | GBIF | 9.96 | 80.96 | 25.18 | 26.30 | 139 | 20 | 44.41 | 134 | 302 | 14 |
| 2294 | 31.72 | 1.79 | GBIF | 11.37 | 80.05 | 22.47 | 24.13 | 155 | 29 | 42.38 | 170 | 372 | 19 |
| 2295 | 30.05 | -0.13 | GBIF | 11.13 | 88.36 | 23.07 | 23.07 | 112 | 23 | 45.82 | 147 | 274 | 15 |
| 2296 | 31.57 | 2.30 | GBIF | 11.07 | 70.09 | 23.95 | 26.13 | 150 | 20 | 45.95 | 147 | 404 | 15 |
| 2297 | 31.72 | 2.18 | GBIF | 10.92 | 74.26 | 23.65 | 25.52 | 148 | 23 | 44.54 | 145 | 411 | 2 |
| 2298 | 31.40 | 1.98 | GBIF | 9.50 | 80.51 | 25.02 | 26.05 | 137 | 20 | 44.99 | 130 | 344 |  |
| 2299 | 31.76 | 1.91 | GBIF | 10.75 | 80.83 | 22.85 | 24.33 | 149 | 26 | 43.15 | 156 | 363 | 4 |

| 2300 | 31.72 | 1.99 | GBIF | 10.51 | 79.01 | 22.65 | 24.25 | 146 | 26 | 43.10 | 152 | 367 | 7 |
| --- | --- | --- | --- | --- | --- | --- | --- | --- | --- | --- | --- | --- | --- |
| 2301 | 31.78 | 1.95 | GBIF | 10.68 | 80.33 | 22.78 | 24.30 | 147 | 26 | 42.84 | 155 | 366 | 4 |
| 2302 | 31.58 | 2.32 | GBIF | 11.17 | 69.36 | 23.82 | 26.05 | 147 | 19 | 46.06 | 137 | 396 | 15 |
| 2303 | 31.76 | 2.16 | GBIF | 10.87 | 75.46 | 23.42 | 25.20 | 147 | 24 | 44.23 | 147 | 413 | 0 |
| 2304 | 31.73 | 1.94 | GBIF | 10.49 | 80.71 | 22.73 | 24.22 | 150 | 26 | 43.47 | 153 | 366 | 3 |
| 2305 | 31.72 | 2.04 | GBIF | 10.61 | 78.00 | 23.58 | 25.18 | 147 | 26 | 43.35 | 152 | 359 | 18 |
| 2306 | 31.68 | 1.94 | GBIF | 10.44 | 80.94 | 22.87 | 24.38 | 149 | 26 | 43.53 | 153 | 359 | 3 |
| 2307 | 29.81 | -0.11 | GBIF | 11.31 | 87.66 | 23.12 | 23.08 | 128 | 29 | 45.06 | 258 | 287 | 11 |
| 2308 | 29.89 | -0.14 | GBIF | 10.87 | 88.35 | 23.13 | 23.13 | 111 | 23 | 45.83 | 230 | 271 | 12 |
| 2309 | 29.88 | -0.16 | GBIF | 10.27 | 90.06 | 23.15 | 23.07 | 109 | 23 | 45.79 | 145 | 271 |  |
| 2310 | 31.68 | 2.38 | GBIF | 11.20 | 67.47 | 23.12 | 25.47 | 147 | 20 | 46.27 | 130 | 411 | 8 |
| 2311 | 29.81 | -0.18 | GBIF | 9.29 | 91.09 | 23.03 | 22.90 | 111 | 26 | 44.44 | 234 | 273 |  |
| 2312 | 31.50 | 2.22 | GBIF | 10.78 | 72.86 | 24.45 | 26.07 | 138 | 20 | 45.19 | 133 | 375 | 14 |
| 2313 | 31.53 | 2.37 | GBIF | 11.36 | 66.04 | 23.65 | 26.12 | 141 | 22 | 45.47 | 137 | 392 | 7 |
| 2314 | 31.77 | 2.42 | GBIF | 11.71 | 69.28 | 22.80 | 25.12 | 153 | 19 | 47.42 | 128 | 438 | 2 |
| 2315 | 30.00 | -0.15 | GBIF | 10.90 | 88.62 | 23.05 | 23.05 | 109 | 23 | 45.76 | 148 | 274 | 9 |
| 2316 | 29.74 | -0.48 | GBIF | 11.27 | 89.42 | 22.92 | 22.85 | 110 | 29 | 39.71 | 169 | 154 | 5 |
| 2317 | 30.08 | -0.10 | GBIF | 11.04 | 88.33 | 23.13 | 23.10 | 111 | 24 | 45.16 | 150 | 274 | 9 |
| 2318 | 31.48 | 2.39 | GBIF | 10.93 | 64.26 | 23.68 | 26.23 | 122 | 19 | 45.67 | 112 | 353 |  |
| 2319 | 31.41 | 2.20 | GBIF | 10.06 | 71.34 | 24.18 | 26.10 | 126 | 20 | 44.43 | 125 | 350 | 8 |
| 2320 | 31.41 | 2.28 | GBIF | 10.66 | 68.76 | 23.83 | 26.03 | 133 | 21 | 45.50 | 129 | 380 | 16 |
| 2321 | 31.47 | 2.26 | GBIF | 10.82 | 70.70 | 24.12 | 26.17 | 135 | 19 | 45.71 | 128 | 370 | 13 |
| 2322 | 31.59 | 2.36 | GBIF | 11.34 | 67.91 | 23.63 | 25.98 | 148 | 21 | 45.68 | 146 | 406 | 15 |
| 2323 | 33.67 | 3.76 | GBIF | 13.16 | 78.32 | 21.87 | 24.42 | 126 | 8 | 56.48 | 89 | 325 | 5 |
| 2324 | 33.61 | 3.71 | GBIF | 11.93 | 75.00 | 20.57 | 23.18 | 148 | 9 | 60.79 | 91 | 375 | 3 |
| 2325 | 29.95 | -0.04 | GBIF | 10.95 | 86.90 | 21.83 | 21.95 | 134 | 33 | 41.75 | 292 | 339 | 5 |
| 2326 | 33.78 | 3.73 | GBIF | 12.44 | 76.33 | 20.98 | 23.68 | 128 | 7 | 59.59 | 83 | 349 | 3 |
| 2327 | 30.00 | -0.04 | GBIF | 11.20 | 87.50 | 22.82 | 22.82 | 126 | 29 | 43.99 | 256 | 290 | 6 |
| 2328 | 30.05 | -0.05 | GBIF | 11.72 | 87.44 | 23.10 | 23.17 | 127 | 27 | 45.15 | 254 | 283 | 4 |
| 2329 | 29.83 | -0.27 | GBIF | 8.93 | 92.01 | 23.03 | 22.85 | 108 | 25 | 43.77 | 155 | 152 |  |
| 2330 | 31.86 | 2.50 | GBIF | 11.88 | 71.11 | 22.80 | 25.13 | 154 | 19 | 47.95 | 130 | 450 | 11 |
| 2331 | 31.43 | 2.22 | GBIF | 10.05 | 71.28 | 24.10 | 26.05 | 127 | 18 | 45.40 | 118 | 352 | 8 |
| 2332 | 30.07 | -0.07 | GBIF | 11.72 | 86.79 | 23.15 | 23.23 | 117 | 25 | 44.98 | 153 | 281 | 3 |
| 2333 | 32.22 | 1.40 | GBIF | 11.38 | 81.25 | 23.40 | 23.68 | 157 | 28 | 42.95 | 166 | 324 | 11 |
| 2334 | 29.70 | -0.41 | GBIF | 9.21 | 92.08 | 22.98 | 22.82 | 107 | 28 | 39.09 | 166 | 139 |  |
| 2335 | 30.00 | -0.07 | GBIF | 11.38 | 87.50 | 22.90 | 22.95 | 120 | 25 | 45.18 | 247 | 286 | 5 |
| 2336 | 30.06 | 0.02 | GBIF | 10.48 | 89.53 | 23.00 | 22.90 | 122 | 29 | 44.33 | 244 | 278 |  |
| 2337 | 30.16 | -0.14 | GBIF | 10.98 | 86.48 | 22.77 | 22.75 | 128 | 26 | 44.34 | 175 | 297 | 7 |
| 2338 | 29.90 | -0.30 | GBIF | 10.49 | 89.67 | 22.32 | 22.22 | 117 | 25 | 43.93 | 171 | 153 | 12 |
| 2339 | 30.19 | -0.15 | GBIF | 11.01 | 86.68 | 22.63 | 22.57 | 124 | 26 | 44.17 | 175 | 299 | 7 |
| 2340 | 30.12 | -0.10 | GBIF | 10.82 | 88.66 | 23.07 | 23.03 | 118 | 25 | 45.38 | 156 | 281 |  |
| 2341 | 29.99 | -0.20 | GBIF | 10.69 | 88.36 | 22.80 | 22.77 | 113 | 24 | 44.36 | 162 | 288 | 8 |
| 2342 | 30.02 | -0.19 | GBIF | 10.93 | 87.40 | 22.82 | 22.75 | 116 | 24 | 45.25 | 161 | 289 | 9 |
| 2343 | 30.17 | -0.05 | GBIF | 10.42 | 89.80 | 23.13 | 23.10 | 121 | 26 | 45.22 | 155 | 279 | 6 |
| 2344 | 29.91 | -0.27 | GBIF | 10.64 | 89.43 | 22.55 | 22.50 | 115 | 25 | 44.29 | 166 | 297 | 6 |
| 2345 | 29.88 | -0.18 | GBIF | 9.98 | 89.86 | 23.12 | 23.02 | 110 | 23 | 45.40 | 233 | 271 |  |
| 2346 | 30.11 | -0.19 | GBIF | 11.00 | 86.61 | 22.37 | 22.37 | 121 | 25 | 44.95 | 171 | 308 | 9 |
| 2347 | 30.20 | -0.09 | GBIF | 10.34 | 88.39 | 22.97 | 22.93 | 120 | 25 | 45.23 | 157 | 282 |  |
| 2348 | 31.53 | 2.39 | GBIF | 11.41 | 65.57 | 23.60 | 26.08 | 141 | 21 | 46.16 | 133 | 394 | 7 |
| 2349 | 29.81 | -0.19 | GBIF | 9.03 | 92.18 | 23.05 | 22.87 | 109 | 26 | 44.03 | 233 | 125 |  |

| 2350 | 29.75 | -0.40 | GBIF | 9.89 | 91.59 | 22.97 | 22.82 | 107 | 27 | 41.31 | 163 | 147 |  |
| --- | --- | --- | --- | --- | --- | --- | --- | --- | --- | --- | --- | --- | --- |
| 2351 | 31.53 | 2.22 | GBIF | 10.88 | 72.08 | 23.95 | 25.95 | 142 | 20 | 45.53 | 137 | 389 | 13 |
| 2352 | 31.40 | 2.40 | GBIF | 11.37 | 63.86 | 23.47 | 26.08 | 120 | 17 | 47.18 | 102 | 347 | 8 |
| 2353 | 31.68 | 2.29 | GBIF | 10.83 | 71.74 | 23.70 | 25.73 | 146 | 21 | 44.65 | 144 | 403 | 8 |
| 2354 | 31.51 | 2.27 | GBIF | 10.86 | 70.51 | 24.10 | 26.20 | 138 | 20 | 45.48 | 137 | 381 | 11 |
| 2355 | 30.00 | -0.17 | GBIF | 10.88 | 88.48 | 22.90 | 22.88 | 111 | 24 | 45.25 | 154 | 282 |  |
| 2356 | 33.78 | 3.72 | GBIF | 12.62 | 76.93 | 21.03 | 23.70 | 128 | 7 | 59.81 | 83 | 350 | 4 |
| 2357 | 31.80 | 2.34 | GBIF | 11.39 | 71.20 | 23.50 | 25.67 | 148 | 22 | 45.15 | 142 | 420 | 7 |
| 2358 | 31.47 | 2.30 | GBIF | 11.13 | 68.25 | 23.80 | 26.08 | 134 | 21 | 44.61 | 134 | 379 | 1 |
| 2359 | 31.50 | 2.21 | GBIF | 10.99 | 73.77 | 24.10 | 26.02 | 140 | 20 | 45.65 | 132 | 382 | 13 |
| 2360 | 31.57 | 2.28 | GBIF | 10.72 | 71.03 | 24.08 | 26.17 | 142 | 20 | 45.23 | 141 | 387 | 11 |
| 2361 | 29.95 | -0.31 | GBIF | 10.41 | 89.73 | 22.48 | 22.33 | 116 | 24 | 44.78 | 166 | 152 | 4 |
| 2362 | 30.15 | -0.02 | GBIF | 10.71 | 89.24 | 23.18 | 23.15 | 124 | 28 | 44.73 | 157 | 280 |  |
| 2363 | 31.51 | 2.25 | GBIF | 10.86 | 71.44 | 24.17 | 26.25 | 135 | 19 | 45.21 | 130 | 371 | 13 |
| 2364 | 31.46 | 2.36 | GBIF | 10.53 | 65.02 | 23.67 | 26.08 | 124 | 18 | 46.48 | 111 | 356 | 17 |
| 2365 | 31.72 | 2.15 | GBIF | 10.91 | 75.23 | 23.67 | 25.48 | 146 | 24 | 44.00 | 148 | 404 | 3 |
| 2366 | 30.10 | -0.21 | GBIF | 10.93 | 87.40 | 22.13 | 22.15 | 126 | 25 | 44.59 | 182 | 320 | 15 |
| 2367 | 29.91 | -0.21 | GBIF | 10.44 | 90.01 | 22.90 | 22.88 | 121 | 28 | 43.07 | 168 | 292 | 5 |
| 2368 | 29.86 | -0.43 | GBIF | 10.58 | 89.62 | 22.53 | 22.38 | 119 | 24 | 43.93 | 170 | 155 | 12 |
| 2369 | 29.94 | -0.08 | GBIF | 11.13 | 87.60 | 22.25 | 22.32 | 134 | 30 | 43.80 | 283 | 325 | 6 |
| 2370 | 29.96 | -0.23 | GBIF | 11.14 | 87.73 | 22.75 | 22.72 | 126 | 26 | 44.97 | 171 | 301 | 12 |
| 2371 | 29.71 | -0.55 | GBIF | 11.41 | 89.83 | 22.85 | 22.85 | 112 | 31 | 38.17 | 173 | 293 | 6 |
| 2372 | 30.09 | -0.16 | GBIF | 11.09 | 87.34 | 22.75 | 22.73 | 124 | 26 | 45.36 | 166 | 295 | 9 |
| 2373 | 30.08 | -0.23 | GBIF | 10.86 | 87.57 | 22.48 | 22.43 | 120 | 24 | 45.60 | 167 | 301 | 7 |
| 2374 | 33.68 | 3.72 | GBIF | 12.88 | 77.56 | 21.42 | 23.97 | 132 | 8 | 58.05 | 88 | 363 | 18 |
| 2375 | 30.05 | -0.06 | GBIF | 11.77 | 87.16 | 23.13 | 23.20 | 120 | 26 | 45.40 | 152 | 282 | 4 |
| 2376 | 29.66 | -0.61 | GBIF | 11.63 | 89.49 | 22.85 | 22.93 | 113 | 33 | 35.45 | 182 | 296 | 8 |
| 2377 | 31.72 | 2.24 | GBIF | 10.88 | 72.99 | 23.52 | 25.48 | 147 | 22 | 44.93 | 141 | 410 | 1 |
| 2378 | 29.91 | -0.15 | GBIF | 10.81 | 88.59 | 23.12 | 23.10 | 114 | 23 | 46.44 | 235 | 275 | 18 |
| 2379 | 31.53 | 2.50 | GBIF | 10.92 | 61.33 | 23.07 | 25.82 | 130 | 16 | 48.06 | 104 | 369 | 18 |
| 2380 | 29.86 | -0.09 | GBIF | 11.63 | 86.82 | 23.00 | 23.12 | 117 | 26 | 45.04 | 240 | 288 | 6 |
| 2381 | 31.55 | 2.54 | GBIF | 11.01 | 61.50 | 22.62 | 25.40 | 139 | 16 | 48.58 | 110 | 395 | 7 |
| 2382 | 31.42 | 2.53 | GBIF | 11.61 | 59.84 | 22.80 | 25.75 | 137 | 17 | 48.54 | 205 | 392 | 17 |
| 2383 | 31.55 | 2.37 | GBIF | 11.38 | 66.91 | 23.62 | 26.05 | 145 | 21 | 45.59 | 141 | 403 | 7 |
| 2384 | 30.16 | -0.06 | GBIF | 10.67 | 88.89 | 23.22 | 23.20 | 117 | 25 | 45.51 | 153 | 276 | 4 |
| 2385 | 30.14 | -0.01 | GBIF | 10.47 | 88.77 | 23.12 | 23.07 | 118 | 28 | 44.53 | 151 | 274 |  |
| 2386 | 30.10 | -0.11 | GBIF | 10.73 | 88.71 | 23.02 | 23.02 | 118 | 25 | 45.67 | 155 | 280 |  |
| 2387 | 31.67 | 2.33 | GBIF | 11.17 | 69.36 | 23.62 | 25.83 | 146 | 20 | 45.49 | 136 | 402 | 8 |
| 2388 | 31.68 | 2.31 | GBIF | 11.13 | 70.46 | 23.42 | 25.58 | 147 | 21 | 45.36 | 137 | 407 | 4 |
| 2389 | 31.71 | 2.21 | GBIF | 10.86 | 73.37 | 23.52 | 25.45 | 147 | 23 | 44.55 | 144 | 410 | 2 |
| 2390 | 31.70 | 2.24 | GBIF | 10.69 | 72.73 | 23.38 | 25.37 | 147 | 22 | 44.65 | 140 | 411 | 5 |
| 2391 | 31.73 | 2.38 | GBIF | 11.52 | 69.38 | 23.17 | 25.47 | 150 | 20 | 46.32 | 131 | 422 | 11 |
| 2392 | 31.68 | 2.30 | GBIF | 11.02 | 71.08 | 23.50 | 25.63 | 147 | 21 | 45.23 | 138 | 406 | 8 |
| 2393 | 31.76 | 2.21 | GBIF | 10.96 | 74.04 | 23.52 | 25.43 | 146 | 23 | 44.73 | 143 | 412 | 7 |
| 2394 | 31.63 | 2.35 | GBIF | 11.34 | 67.91 | 23.60 | 25.92 | 145 | 19 | 46.11 | 131 | 398 | 15 |
| 2395 | 29.92 | -0.07 | GBIF | 11.14 | 87.04 | 22.12 | 22.20 | 133 | 31 | 42.89 | 284 | 330 | 6 |
| 2396 | 31.61 | 2.27 | GBIF | 10.59 | 72.05 | 24.17 | 26.17 | 139 | 19 | 45.07 | 136 | 382 | 0 |
| 2397 | 31.64 | 2.24 | GBIF | 10.62 | 73.22 | 24.02 | 25.93 | 142 | 20 | 45.11 | 137 | 392 | 1 |
| 2398 | 30.40 | 0.52 | GBIF | 10.42 | 86.81 | 19.73 | 20.07 | 202 | 48 | 46.74 | 333 | 220 | 6 |
| 2399 | 30.10 | -0.15 | GBIF | 11.17 | 87.93 | 22.78 | 22.75 | 126 | 27 | 45.01 | 167 | 295 | 8 |

| 2400 | 29.87 | -0.60 | GBIF | 10.82 | 90.97 | 21.95 | 21.83 | 129 | 28 | 41.65 | 190 | 156 | 7 |
| --- | --- | --- | --- | --- | --- | --- | --- | --- | --- | --- | --- | --- | --- |
| 2401 | 31.77 | 2.29 | GBIF | 11.25 | 72.12 | 23.47 | 25.57 | 148 | 22 | 45.13 | 140 | 416 | 7 |
| 2402 | 31.69 | 2.26 | GBIF | 10.67 | 72.62 | 23.68 | 25.63 | 146 | 21 | 45.04 | 140 | 404 | 2 |
| 2403 | 30.18 | -0.05 | GBIF | 9.95 | 89.64 | 23.07 | 22.98 | 117 | 26 | 45.04 | 155 | 278 |  |
| 2404 | 30.14 | 0.01 | GBIF | 10.24 | 89.06 | 23.03 | 22.93 | 122 | 29 | 44.55 | 152 | 278 | 9 |
| 2405 | 29.96 | -0.46 | GBIF | 10.56 | 91.02 | 21.75 | 21.68 | 134 | 27 | 43.08 | 199 | 348 | 6 |
| 2406 | 29.85 | -0.45 | GBIF | 10.54 | 90.10 | 22.45 | 22.30 | 119 | 26 | 42.77 | 172 | 157 | 4 |
| 2407 | 29.80 | -0.63 | GBIF | 11.25 | 90.00 | 22.45 | 22.33 | 119 | 30 | 39.22 | 182 | 160 | 10 |
| 2408 | 30.11 | -0.02 | GBIF | 11.72 | 86.85 | 23.22 | 23.32 | 123 | 27 | 45.36 | 154 | 281 | 5 |
| 2409 | 31.49 | 2.21 | GBIF | 10.78 | 72.86 | 24.50 | 26.10 | 135 | 19 | 45.65 | 127 | 370 | 14 |
| 2410 | 30.07 | -0.09 | GBIF | 11.45 | 88.08 | 23.08 | 23.15 | 116 | 25 | 44.99 | 153 | 282 | 5 |
| 2411 | 29.82 | -0.17 | GBIF | 9.57 | 91.11 | 23.05 | 22.92 | 111 | 25 | 45.12 | 232 | 272 |  |
| 2412 | 30.07 | -0.03 | GBIF | 11.88 | 86.68 | 23.17 | 23.30 | 126 | 27 | 45.65 | 154 | 282 | 4 |
| 2413 | 30.06 | -0.11 | GBIF | 11.24 | 88.52 | 23.08 | 23.10 | 116 | 24 | 45.44 | 151 | 277 | 14 |
| 2414 | 29.85 | -0.05 | GBIF | 11.72 | 86.79 | 22.73 | 23.00 | 146 | 39 | 42.89 | 292 | 322 | 6 |
| 2415 | 29.87 | -0.07 | GBIF | 11.52 | 86.65 | 22.70 | 22.80 | 130 | 31 | 43.44 | 268 | 310 | 6 |
| 2416 | 30.19 | -0.16 | GBIF | 11.04 | 86.26 | 22.62 | 22.52 | 124 | 25 | 44.75 | 172 | 298 | 14 |
| 2417 | 29.84 | -0.17 | GBIF | 9.86 | 90.44 | 23.05 | 22.90 | 109 | 24 | 44.82 | 229 | 271 | 10 |
| 2418 | 29.93 | -0.18 | GBIF | 11.02 | 88.13 | 23.08 | 23.08 | 114 | 24 | 45.62 | 153 | 279 | 7 |
| 2419 | 29.68 | -0.48 | GBIF | 11.09 | 90.92 | 22.95 | 22.82 | 109 | 31 | 37.75 | 168 | 153 | 12 |
| 2420 | 31.48 | 2.32 | GBIF | 11.23 | 68.03 | 23.75 | 26.05 | 135 | 21 | 44.42 | 136 | 380 | 2 |
| 2421 | 29.85 | -0.01 | GBIF | 11.58 | 87.09 | 22.52 | 22.82 | 139 | 40 | 41.64 | 283 | 326 | 17 |
| 2422 | 31.54 | 2.32 | GBIF | 11.33 | 69.11 | 23.85 | 26.15 | 145 | 22 | 44.98 | 146 | 394 | 8 |
| 2423 | 31.52 | 2.38 | GBIF | 11.40 | 65.52 | 23.65 | 26.15 | 138 | 21 | 45.55 | 134 | 388 | 7 |
| 2424 | 29.84 | -0.14 | GBIF | 10.74 | 88.05 | 23.07 | 22.98 | 120 | 25 | 45.77 | 243 | 277 | 19 |
| 2425 | 29.87 | -0.35 | GBIF | 10.19 | 91.00 | 22.80 | 22.62 | 111 | 25 | 43.44 | 158 | 156 | 12 |
| 2426 | 33.80 | -0.83 | GBIF | 8.34 | 84.26 | 22.90 | 21.88 | 190 | 30 | 58.85 | 270 | 106 |  |
| 2427 | 29.92 | -0.25 | GBIF | 10.89 | 88.55 | 22.63 | 22.63 | 116 | 25 | 44.94 | 160 | 296 | 6 |
| 2428 | 29.92 | -0.36 | GBIF | 10.34 | 90.72 | 22.23 | 22.08 | 121 | 25 | 43.56 | 175 | 165 | 4 |
| 2429 | 29.80 | -0.09 | GBIF | 11.48 | 86.93 | 23.05 | 23.05 | 121 | 29 | 44.21 | 249 | 291 | 12 |
| 2430 | 29.95 | -0.18 | GBIF | 10.86 | 88.28 | 23.12 | 23.08 | 112 | 22 | 46.02 | 152 | 279 | 8 |
| 2431 | 31.76 | 2.25 | GBIF | 11.10 | 72.55 | 23.72 | 25.72 | 147 | 22 | 44.92 | 142 | 410 | 0 |
| 2432 | 29.89 | -0.34 | GBIF | 10.31 | 90.42 | 22.30 | 22.18 | 118 | 25 | 43.27 | 172 | 160 | 5 |
| 2433 | 31.76 | 2.27 | GBIF | 11.13 | 72.77 | 23.63 | 25.67 | 147 | 22 | 44.95 | 142 | 412 | 7 |
| 2434 | 29.84 | -0.19 | GBIF | 9.35 | 91.67 | 23.07 | 22.88 | 109 | 24 | 45.15 | 232 | 121 |  |
| 2435 | 30.16 | -0.07 | GBIF | 10.74 | 88.77 | 23.20 | 23.17 | 116 | 25 | 45.18 | 153 | 274 | 8 |
| 2436 | 30.20 | -0.05 | GBIF | 9.72 | 89.14 | 23.00 | 22.88 | 116 | 26 | 44.55 | 240 | 276 |  |
| 2437 | 31.72 | 2.32 | GBIF | 11.29 | 71.02 | 23.47 | 25.62 | 148 | 21 | 45.49 | 137 | 415 | 8 |
| 2438 | 29.93 | -0.03 | GBIF | 11.02 | 86.75 | 21.58 | 21.70 | 140 | 36 | 40.52 | 298 | 350 | 6 |
| 2439 | 29.81 | -0.15 | GBIF | 9.83 | 90.97 | 23.05 | 22.87 | 112 | 26 | 44.69 | 234 | 128 |  |
| 2440 | 29.89 | -0.35 | GBIF | 10.31 | 90.42 | 22.28 | 22.17 | 119 | 25 | 43.06 | 175 | 159 | 5 |
| 2441 | 30.06 | -0.17 | GBIF | 10.95 | 87.60 | 22.78 | 22.75 | 114 | 24 | 45.18 | 158 | 287 | 16 |
| 2442 | 31.76 | 2.33 | GBIF | 11.37 | 71.04 | 23.60 | 25.75 | 147 | 21 | 45.34 | 137 | 415 | 7 |
| 2443 | 29.94 | -0.03 | GBIF | 10.97 | 86.35 | 21.37 | 21.47 | 141 | 36 | 40.63 | 219 | 358 | 5 |
| 2444 | 31.65 | 2.28 | GBIF | 10.70 | 71.81 | 23.98 | 25.98 | 143 | 20 | 45.04 | 136 | 391 | 0 |
| 2445 | 30.09 | -0.14 | GBIF | 11.00 | 88.00 | 22.90 | 22.90 | 119 | 26 | 44.47 | 161 | 288 | 9 |
| 2446 | 31.65 | 2.29 | GBIF | 10.90 | 71.24 | 23.85 | 25.92 | 144 | 20 | 45.13 | 137 | 395 | 0 |
| 2447 | 30.13 | -0.17 | GBIF | 11.10 | 86.72 | 22.60 | 22.60 | 119 | 25 | 45.02 | 166 | 299 | 8 |
| 2448 | 30.16 | -0.03 | GBIF | 10.56 | 88.73 | 23.17 | 23.13 | 122 | 28 | 44.47 | 157 | 281 | 2 |
| 2449 | 30.12 | -0.03 | GBIF | 11.61 | 87.28 | 23.20 | 23.30 | 123 | 26 | 45.66 | 155 | 280 | 4 |

| 2450 | 30.14 | -0.06 | GBIF | 11.20 | 88.19 | 23.22 | 23.23 | 117 | 25 | 45.40 | 154 | 278 | 4 |
| --- | --- | --- | --- | --- | --- | --- | --- | --- | --- | --- | --- | --- | --- |
| 2451 | 30.12 | -0.12 | GBIF | 10.76 | 88.18 | 22.95 | 22.93 | 121 | 28 | 44.02 | 162 | 286 | 7 |
| 2452 | 30.11 | -0.08 | GBIF | 11.38 | 88.18 | 23.15 | 23.20 | 117 | 24 | 45.55 | 153 | 279 | 5 |
| 2453 | 30.14 | -0.07 | GBIF | 11.18 | 88.06 | 23.22 | 23.23 | 115 | 25 | 45.39 | 153 | 278 | 5 |
| 2454 | 29.70 | -0.58 | GBIF | 11.56 | 89.60 | 22.82 | 22.83 | 112 | 30 | 37.92 | 175 | 293 | 10 |
| 2455 | 31.42 | 2.33 | GBIF | 10.63 | 66.46 | 23.80 | 26.13 | 122 | 18 | 46.11 | 112 | 351 |  |
| 2456 | 30.09 | -0.06 | GBIF | 11.69 | 87.25 | 23.13 | 23.22 | 119 | 26 | 45.02 | 155 | 283 | 2 |
| 2457 | 29.82 | -0.08 | GBIF | 11.63 | 86.82 | 23.00 | 23.05 | 116 | 29 | 43.53 | 243 | 291 | 11 |
| 2458 | 30.16 | -0.18 | GBIF | 10.82 | 86.60 | 22.37 | 22.37 | 126 | 26 | 44.27 | 182 | 313 | 9 |
| 2459 | 33.65 | 3.62 | GBIF | 10.90 | 74.66 | 18.92 | 21.42 | 167 | 9 | 66.51 | 89 | 416 | 4 |
| 2460 | 31.47 | 2.50 | GBIF | 11.10 | 60.33 | 23.07 | 25.87 | 129 | 17 | 48.08 | 198 | 371 | 23 |
| 2461 | 31.59 | 2.47 | GBIF | 11.13 | 63.21 | 23.10 | 25.77 | 139 | 17 | 47.48 | 115 | 389 | 0 |
| 2462 | 31.59 | 2.20 | GBIF | 10.88 | 72.08 | 23.68 | 25.72 | 145 | 22 | 44.59 | 141 | 398 | 9 |
| 2463 | 31.49 | 2.36 | GBIF | 10.98 | 65.72 | 23.75 | 26.20 | 126 | 18 | 46.00 | 113 | 356 | 11 |
| 2464 | 31.57 | 2.53 | GBIF | 11.07 | 62.52 | 22.73 | 25.43 | 140 | 17 | 48.11 | 112 | 395 | 1 |
| 2465 | 31.73 | 2.43 | GBIF | 11.59 | 68.19 | 22.85 | 25.23 | 150 | 19 | 47.13 | 127 | 426 | 11 |
| 2466 | 30.01 | -0.19 | GBIF | 10.85 | 88.21 | 22.78 | 22.77 | 115 | 25 | 44.14 | 166 | 289 | 13 |
| 2467 | 31.52 | 2.34 | GBIF | 11.35 | 67.96 | 23.68 | 26.03 | 142 | 22 | 44.99 | 141 | 391 | 11 |
| 2468 | 29.64 | -0.79 | GBIF | 11.39 | 90.41 | 21.95 | 21.82 | 120 | 33 | 34.01 | 198 | 160 | 7 |
| 2469 | 29.70 | -0.46 | GBIF | 10.77 | 91.24 | 22.92 | 22.77 | 107 | 29 | 38.79 | 165 | 151 | 5 |
| 2470 | 31.74 | 2.37 | GBIF | 11.57 | 69.68 | 23.15 | 25.42 | 150 | 20 | 46.23 | 132 | 422 | 15 |
| 2471 | 31.70 | 2.36 | GBIF | 11.27 | 69.17 | 23.38 | 25.65 | 147 | 20 | 45.94 | 131 | 409 | 8 |
| 2472 | 31.54 | 2.36 | GBIF | 11.37 | 67.66 | 23.72 | 26.10 | 141 | 21 | 45.05 | 142 | 393 | 8 |
| 2473 | 29.81 | -0.35 | GBIF | 9.21 | 91.17 | 22.97 | 22.80 | 107 | 26 | 42.44 | 160 | 148 |  |
| 2474 | 31.44 | 2.24 | GBIF | 10.38 | 70.58 | 24.15 | 26.13 | 127 | 19 | 44.77 | 122 | 354 | 6 |
| 2475 | 29.91 | -0.05 | GBIF | 11.16 | 87.17 | 22.10 | 22.20 | 136 | 34 | 42.12 | 289 | 333 | 6 |
| 2476 | 32.11 | 1.38 | GBIF | 11.40 | 81.43 | 23.52 | 23.78 | 155 | 29 | 42.52 | 170 | 320 | 7 |
| 2477 | 31.53 | 2.27 | GBIF | 10.76 | 70.78 | 24.10 | 26.22 | 136 | 19 | 45.22 | 132 | 372 | 8 |
| 2478 | 32.90 | 2.25 | GBIF | 12.97 | 77.64 | 22.45 | 24.42 | 181 | 20 | 51.40 | 138 | 440 | 25 |
| 2479 | 30.05 | -0.02 | GBIF | 11.67 | 87.06 | 23.63 | 23.20 | 144 | 33 | 44.99 | 173 | 293 | 5 |
| 2480 | 30.06 | -0.02 | GBIF | 11.78 | 86.64 | 23.15 | 23.25 | 132 | 28 | 45.54 | 161 | 285 | 5 |
| 2481 | 29.99 | -0.26 | GBIF | 10.87 | 87.63 | 22.57 | 22.47 | 118 | 25 | 45.04 | 169 | 302 | 4 |
| 2482 | 31.59 | 2.30 | GBIF | 10.88 | 70.62 | 23.97 | 26.08 | 148 | 20 | 45.51 | 147 | 398 | 9 |
| 2483 | 30.03 | -0.13 | GBIF | 11.22 | 88.32 | 23.03 | 23.02 | 114 | 23 | 46.03 | 150 | 277 | 11 |
| 2484 | 29.83 | -0.38 | GBIF | 10.41 | 90.51 | 22.80 | 22.62 | 111 | 24 | 43.21 | 160 | 149 | 4 |
| 2485 | 33.65 | 3.67 | GBIF | 12.05 | 76.27 | 20.68 | 23.17 | 147 | 9 | 61.10 | 90 | 372 | 11 |
| 2486 | 32.17 | 1.83 | GBIF | 11.49 | 79.80 | 22.02 | 23.70 | 149 | 25 | 42.95 | 151 | 370 | 4 |
| 2487 | 31.42 | 3.50 | GBIF | 12.12 | 69.64 | 24.42 | 27.57 | 168 | 10 | 56.24 | 98 | 477 | 7 |
| 2488 | 31.83 | 2.17 | GBIF | 11.14 | 74.78 | 23.20 | 25.05 | 147 | 24 | 44.44 | 147 | 421 | 0 |
| 2489 | 32.00 | 2.00 | GBIF | 11.23 | 79.61 | 22.00 | 23.58 | 151 | 24 | 43.77 | 150 | 394 | 6 |
| 2490 | 32.33 | 3.08 | GBIF | 12.73 | 76.20 | 22.55 | 24.93 | 188 | 13 | 56.10 | 106 | 497 | 6 |
| 2491 | 30.00 | -0.35 | GBIF | 10.18 | 90.12 | 22.27 | 22.15 | 122 | 25 | 44.59 | 174 | 166 | 2 |
| 2492 | 31.78 | 2.37 | GBIF | 11.57 | 70.53 | 23.28 | 25.52 | 149 | 20 | 46.29 | 133 | 425 | 7 |
| 2493 | 30.13 | -0.14 | GBIF | 10.98 | 87.17 | 22.83 | 22.82 | 121 | 25 | 44.63 | 166 | 290 | 7 |
| 2494 | 30.08 | -0.20 | GBIF | 11.00 | 86.61 | 22.63 | 22.57 | 115 | 24 | 44.99 | 160 | 294 | 12 |
| 2495 | 30.12 | -0.18 | GBIF | 11.12 | 86.85 | 22.50 | 22.50 | 119 | 25 | 44.80 | 169 | 305 | 8 |
| 2496 | 31.42 | 2.31 | GBIF | 10.43 | 66.45 | 23.77 | 26.07 | 121 | 17 | 46.24 | 107 | 342 | 18 |
| 2497 | 29.96 | -0.01 | GBIF | 11.09 | 86.65 | 21.98 | 22.05 | 133 | 36 | 40.11 | 200 | 335 | 10 |
| 2498 | 31.46 | 2.39 | GBIF | 10.79 | 64.24 | 23.52 | 26.05 | 134 | 20 | 47.48 | 118 | 389 | 18 |
| 2499 | 29.83 | -0.15 | GBIF | 10.37 | 89.37 | 23.02 | 22.90 | 110 | 25 | 44.25 | 230 | 274 | 16 |

| 2500 | 29.85 | -0.07 | GBIF | 11.68 | 86.48 | 22.78 | 22.92 | 119 | 29 | 43.34 | 249 | 301 | 6 |
| --- | --- | --- | --- | --- | --- | --- | --- | --- | --- | --- | --- | --- | --- |
| 2501 | 31.65 | 2.26 | GBIF | 10.58 | 72.99 | 24.10 | 26.07 | 140 | 20 | 44.85 | 138 | 385 | 0 |
| 2502 | 31.22 | 2.28 | GBIF | 11.56 | 70.48 | 22.23 | 24.38 | 149 | 22 | 47.13 | 241 | 427 | 14 |
| 2503 | 29.78 | -0.42 | GBIF | 10.84 | 90.35 | 22.83 | 22.75 | 109 | 27 | 41.04 | 164 | 148 | 4 |
| 2504 | 31.40 | 2.48 | GBIF | 11.63 | 61.18 | 22.88 | 25.77 | 128 | 17 | 48.22 | 187 | 369 | 15 |
| 2505 | 31.63 | 2.27 | GBIF | 10.60 | 72.11 | 24.13 | 26.15 | 138 | 20 | 44.72 | 137 | 380 | 0 |
| 2506 | 29.82 | -0.37 | GBIF | 10.09 | 90.92 | 22.88 | 22.70 | 109 | 26 | 42.57 | 161 | 150 |  |
| 2507 | 29.93 | -0.28 | GBIF | 10.72 | 88.57 | 22.48 | 22.40 | 116 | 26 | 44.65 | 166 | 301 | 6 |
| 2508 | 30.02 | -0.10 | GBIF | 11.53 | 88.04 | 23.02 | 23.07 | 119 | 24 | 45.76 | 243 | 282 | 9 |
| 2509 | 29.94 | -0.33 | GBIF | 10.38 | 89.44 | 22.43 | 22.28 | 117 | 24 | 44.66 | 166 | 160 | 4 |
| 2510 | 31.88 | 2.31 | GBIF | 11.54 | 72.14 | 22.98 | 25.12 | 149 | 22 | 45.95 | 139 | 433 | 0 |
| 2511 | 31.49 | 2.02 | GBIF | 10.64 | 77.68 | 24.90 | 26.22 | 139 | 22 | 43.68 | 140 | 365 | 12 |
| 2512 | 31.90 | 2.26 | GBIF | 11.50 | 72.78 | 22.73 | 24.78 | 152 | 22 | 46.14 | 140 | 438 | 0 |
| 2513 | 31.87 | 2.21 | GBIF | 11.53 | 72.94 | 22.90 | 24.90 | 150 | 23 | 45.24 | 145 | 433 | 0 |
| 2514 | 31.91 | 2.37 | GBIF | 11.43 | 71.86 | 23.12 | 25.25 | 149 | 21 | 46.35 | 139 | 437 | 7 |
| 2515 | 29.90 | -0.13 | GBIF | 11.07 | 87.90 | 23.02 | 23.05 | 122 | 26 | 45.47 | 248 | 284 | 9 |
| 2516 | 29.82 | -0.40 | GBIF | 10.52 | 90.66 | 22.82 | 22.63 | 112 | 25 | 43.17 | 161 | 152 | 4 |
| 2517 | 30.16 | 0.00 | GBIF | 10.25 | 89.13 | 23.08 | 23.00 | 120 | 28 | 44.64 | 151 | 276 |  |
| 2518 | 30.18 | -0.13 | GBIF | 10.99 | 86.55 | 22.72 | 22.68 | 132 | 27 | 44.65 | 175 | 297 | 13 |
| 2519 | 30.19 | -0.18 | GBIF | 10.94 | 86.84 | 22.40 | 22.37 | 135 | 27 | 44.61 | 186 | 314 | 8 |
| 2520 | 31.68 | 2.14 | GBIF | 10.93 | 74.83 | 23.80 | 25.62 | 145 | 25 | 43.47 | 150 | 404 | 1 |
| 2521 | 30.17 | -0.17 | GBIF | 10.95 | 86.90 | 22.50 | 22.50 | 125 | 26 | 44.63 | 269 | 309 | 9 |
| 2522 | 31.74 | 2.40 | GBIF | 11.59 | 69.41 | 23.00 | 25.30 | 151 | 20 | 46.57 | 130 | 425 | 11 |
| 2523 | 31.74 | 2.28 | GBIF | 11.14 | 72.35 | 23.55 | 25.60 | 148 | 22 | 44.78 | 143 | 412 | 7 |
| 2524 | 31.46 | 2.32 | GBIF | 11.02 | 67.59 | 23.75 | 26.08 | 130 | 20 | 45.38 | 124 | 366 | 19 |
| 2525 | 30.08 | -0.18 | GBIF | 11.00 | 87.30 | 22.72 | 22.70 | 117 | 24 | 45.74 | 158 | 289 | 16 |
| 2526 | 31.48 | 2.24 | GBIF | 10.73 | 71.56 | 24.23 | 26.27 | 133 | 19 | 44.82 | 129 | 363 | 14 |
| 2527 | 29.80 | -0.05 | GBIF | 11.69 | 85.97 | 23.00 | 23.05 | 126 | 31 | 43.10 | 256 | 300 | 16 |
| 2528 | 33.70 | 3.95 | GBIF | 13.88 | 79.33 | 22.75 | 25.35 | 109 | 7 | 53.62 | 84 | 280 | 8 |
| 2529 | 29.87 | -0.09 | GBIF | 11.54 | 86.78 | 22.82 | 22.90 | 121 | 28 | 44.15 | 250 | 297 | 6 |
| 2530 | 29.78 | -0.47 | GBIF | 11.14 | 89.13 | 22.82 | 22.78 | 112 | 27 | 41.23 | 164 | 150 | 9 |
| 2531 | 30.07 | -0.29 | GBIF | 10.13 | 88.89 | 19.87 | 19.87 | 159 | 32 | 40.00 | 266 | 416 | 15 |
| 2532 | 29.94 | -0.23 | GBIF | 11.07 | 88.60 | 22.78 | 22.83 | 121 | 26 | 44.43 | 168 | 296 | 6 |
| 2533 | 29.80 | -0.07 | GBIF | 11.62 | 86.05 | 23.07 | 23.10 | 123 | 30 | 43.77 | 254 | 294 | 15 |
| 2534 | 29.88 | -0.11 | GBIF | 11.53 | 86.65 | 22.87 | 22.97 | 121 | 28 | 45.05 | 251 | 295 | 6 |
| 2535 | 29.96 | -0.09 | GBIF | 11.23 | 88.39 | 22.82 | 22.87 | 121 | 25 | 45.39 | 247 | 289 | 13 |
| 2536 | 30.13 | -0.19 | GBIF | 10.94 | 86.84 | 22.22 | 22.23 | 125 | 25 | 44.61 | 180 | 319 | 9 |
| 2537 | 30.04 | 0.14 | GBIF | 11.06 | 86.39 | 21.67 | 22.13 | 144 | 42 | 38.69 | 207 | 345 | 8 |
| 2538 | 31.74 | 2.35 | GBIF | 11.43 | 70.58 | 23.37 | 25.55 | 149 | 20 | 46.00 | 134 | 419 | 11 |
| 2539 | 29.90 | -0.07 | GBIF | 11.28 | 86.79 | 22.52 | 22.60 | 132 | 31 | 43.59 | 275 | 316 | 6 |
| 2540 | 31.66 | 2.28 | GBIF | 10.73 | 71.98 | 24.07 | 26.08 | 142 | 20 | 44.79 | 136 | 388 | 4 |
| 2541 | 30.04 | 0.04 | GBIF | 10.94 | 88.24 | 23.05 | 23.35 | 121 | 28 | 43.63 | 148 | 276 | 9 |
| 2542 | 30.07 | 0.05 | GBIF | 10.64 | 89.43 | 22.98 | 23.27 | 123 | 29 | 43.61 | 245 | 275 |  |
| 2543 | 30.02 | -0.03 | GBIF | 11.53 | 86.72 | 23.57 | 23.08 | 142 | 32 | 45.30 | 280 | 294 | 9 |
| 2544 | 29.90 | -0.25 | GBIF | 10.52 | 89.12 | 22.67 | 22.62 | 116 | 26 | 44.42 | 164 | 292 | 5 |
| 2545 | 29.76 | -0.65 | GBIF | 11.42 | 90.61 | 22.37 | 22.23 | 120 | 32 | 37.93 | 188 | 166 | 9 |
| 2546 | 30.05 | -0.34 | GBIF | 10.07 | 90.77 | 21.38 | 21.32 | 142 | 28 | 42.48 | 216 | 367 | 6 |
| 2547 | 30.36 | -0.31 | GBIF | 11.03 | 86.81 | 18.32 | 18.22 | 155 | 35 | 38.98 | 252 | 180 | 7 |
| 2548 | 29.96 | -0.20 | GBIF | 10.75 | 88.84 | 22.88 | 22.90 | 115 | 24 | 45.09 | 160 | 289 | 6 |
| 2549 | 29.96 | -0.17 | GBIF | 11.01 | 88.07 | 23.07 | 23.07 | 106 | 21 | 46.03 | 146 | 273 | 9 |

| 2550 | 31.46 | 2.25 | GBIF | 10.69 | 70.81 | 24.13 | 26.17 | 131 | 19 | 45.56 | 123 | 361 | 13 |
| --- | --- | --- | --- | --- | --- | --- | --- | --- | --- | --- | --- | --- | --- |
| 2551 | 31.58 | 1.75 | GBIF | 10.19 | 81.53 | 22.37 | 23.87 | 160 | 28 | 43.94 | 168 | 372 | 6 |
| 2552 | 30.14 | -0.17 | GBIF | 10.99 | 86.55 | 22.50 | 22.47 | 119 | 25 | 44.59 | 256 | 306 | 8 |
| 2553 | 33.66 | 3.69 | GBIF | 12.40 | 77.02 | 20.85 | 23.30 | 143 | 8 | 60.83 | 89 | 362 | 11 |
| 2554 | 29.90 | -0.37 | GBIF | 10.32 | 90.57 | 22.15 | 22.02 | 125 | 24 | 44.32 | 179 | 160 | 4 |
| 2555 | 31.52 | 2.39 | GBIF | 11.42 | 64.91 | 23.60 | 26.15 | 138 | 22 | 45.92 | 131 | 389 | 7 |
| 2556 | 30.14 | 0.08 | GBIF | 10.54 | 88.59 | 22.78 | 23.05 | 130 | 31 | 43.39 | 257 | 144 | 13 |
| 2557 | 31.56 | 2.45 | GBIF | 11.27 | 63.34 | 23.30 | 25.98 | 135 | 18 | 46.71 | 117 | 378 | 12 |
| 2558 | 32.18 | 2.35 | GBIF | 11.68 | 76.36 | 22.27 | 24.20 | 168 | 20 | 48.67 | 136 | 449 | 0 |
| 2559 | 31.77 | 2.38 | GBIF | 11.59 | 70.25 | 23.10 | 25.35 | 150 | 20 | 46.51 | 132 | 427 | 8 |
| 2560 | 31.49 | 2.59 | GBIF | 10.96 | 58.60 | 22.85 | 25.82 | 131 | 15 | 48.84 | 186 | 371 | 0 |
| 2561 | 31.72 | 2.23 | GBIF | 10.87 | 72.93 | 23.38 | 25.35 | 147 | 23 | 44.58 | 142 | 412 | 1 |
| 2562 | 31.41 | 2.30 | GBIF | 10.50 | 67.31 | 23.82 | 26.05 | 123 | 18 | 45.72 | 115 | 352 | 13 |
| 2563 | 29.93 | -0.10 | GBIF | 11.29 | 88.22 | 22.53 | 22.60 | 133 | 30 | 44.21 | 275 | 312 | 9 |
| 2564 | 33.63 | 3.79 | GBIF | 12.92 | 76.48 | 21.75 | 24.42 | 131 | 9 | 57.17 | 90 | 336 | 4 |
| 2565 | 31.65 | 1.92 | GBIF | 10.29 | 81.04 | 23.12 | 24.57 | 149 | 26 | 43.54 | 153 | 355 | 3 |
| 2566 | 31.54 | 2.24 | GBIF | 10.83 | 71.74 | 23.97 | 26.03 | 142 | 20 | 45.16 | 139 | 388 | 13 |
| 2567 | 32.36 | 2.68 | GBIF | 12.50 | 78.62 | 22.03 | 24.17 | 194 | 17 | 53.76 | 123 | 493 | 4 |
| 2568 | 29.63 | -0.44 | GBIF | 9.09 | 92.77 | 23.03 | 22.87 | 106 | 31 | 37.13 | 233 | 144 |  |
| 2569 | 31.84 | 2.30 | GBIF | 11.44 | 71.51 | 22.92 | 25.08 | 149 | 22 | 45.46 | 140 | 432 | 7 |
| 2570 | 29.90 | -0.02 | GBIF | 11.25 | 86.54 | 22.18 | 22.30 | 132 | 34 | 41.47 | 273 | 325 | 7 |
| 2571 | 29.67 | -0.61 | GBIF | 11.66 | 89.68 | 22.83 | 22.92 | 122 | 36 | 35.63 | 186 | 298 | 8 |
| 2572 | 29.95 | -0.06 | GBIF | 11.03 | 86.88 | 21.83 | 21.95 | 135 | 33 | 42.36 | 295 | 339 | 5 |
| 2573 | 29.86 | -0.15 | GBIF | 10.49 | 89.67 | 23.08 | 23.02 | 108 | 23 | 45.43 | 224 | 269 |  |
| 2574 | 30.02 | -0.07 | GBIF | 11.58 | 87.09 | 22.95 | 23.02 | 122 | 26 | 45.54 | 247 | 283 | 13 |
| 2575 | 30.20 | -0.01 | GBIF | 9.49 | 90.40 | 22.97 | 22.77 | 118 | 27 | 44.74 | 242 | 124 |  |
| 2576 | 30.06 | -0.07 | GBIF | 11.73 | 86.91 | 23.17 | 23.22 | 117 | 25 | 45.02 | 153 | 280 | 4 |
| 2577 | 29.85 | -0.33 | GBIF | 9.86 | 90.44 | 22.85 | 22.67 | 108 | 25 | 42.91 | 159 | 151 |  |
| 2578 | 30.08 | 0.19 | GBIF | 11.20 | 87.50 | 22.52 | 22.95 | 133 | 34 | 40.50 | 262 | 294 | 22 |
| 2579 | 30.09 | -0.12 | GBIF | 10.89 | 88.55 | 23.10 | 23.03 | 113 | 24 | 45.55 | 150 | 275 |  |
| 2580 | 30.00 | 0.00 | GBIF | 11.23 | 87.70 | 22.98 | 23.32 | 126 | 29 | 44.16 | 254 | 289 | 13 |
| 2581 | 30.11 | -0.15 | GBIF | 11.09 | 87.34 | 22.80 | 22.78 | 127 | 26 | 45.05 | 169 | 294 | 8 |
| 2582 | 30.10 | 0.17 | GBIF | 11.24 | 87.14 | 23.17 | 23.07 | 151 | 36 | 42.36 | 288 | 301 | 26 |
| 2583 | 31.69 | 2.04 | GBIF | 10.64 | 77.68 | 24.10 | 25.72 | 148 | 25 | 43.33 | 149 | 348 | 2 |
| 2584 | 31.55 | 2.26 | GBIF | 10.67 | 71.11 | 24.02 | 26.08 | 142 | 20 | 45.28 | 137 | 385 | 18 |
| 2585 | 31.53 | 2.28 | GBIF | 10.85 | 70.45 | 24.07 | 26.20 | 140 | 20 | 45.19 | 140 | 383 | 11 |
| 2586 | 29.79 | -0.66 | GBIF | 11.31 | 91.20 | 22.40 | 22.32 | 120 | 30 | 38.42 | 185 | 157 | 9 |
| 2587 | 29.75 | -0.51 | GBIF | 11.30 | 88.98 | 22.92 | 22.92 | 115 | 29 | 40.22 | 170 | 295 | 7 |
| 2588 | 31.73 | 2.14 | GBIF | 10.85 | 75.35 | 23.70 | 25.43 | 145 | 24 | 43.99 | 148 | 404 | 2 |
| 2589 | 29.76 | -0.66 | GBIF | 11.39 | 91.13 | 22.10 | 21.98 | 123 | 32 | 37.63 | 194 | 164 | 9 |
| 2590 | 29.68 | -0.42 | GBIF | 9.17 | 91.75 | 23.02 | 22.80 | 106 | 29 | 39.03 | 166 | 138 |  |
| 2591 | 29.64 | -0.51 | GBIF | 9.91 | 92.60 | 23.02 | 22.90 | 110 | 35 | 35.33 | 181 | 158 | 8 |
| 2592 | 29.83 | -0.01 | GBIF | 11.65 | 87.59 | 22.65 | 22.88 | 144 | 40 | 41.84 | 289 | 325 | 13 |
| 2593 | 29.79 | -0.67 | GBIF | 11.28 | 90.27 | 22.28 | 22.15 | 121 | 28 | 38.80 | 186 | 155 | 15 |
| 2594 | 29.61 | -0.65 | GBIF | 11.68 | 91.21 | 22.62 | 22.63 | 130 | 43 | 31.95 | 209 | 312 | 9 |
| 2595 | 29.77 | -0.74 | GBIF | 11.35 | 90.80 | 20.87 | 20.73 | 142 | 36 | 36.35 | 230 | 178 | 15 |
| 2596 | 31.65 | 2.22 | GBIF | 10.72 | 72.90 | 23.55 | 25.50 | 146 | 23 | 44.45 | 142 | 403 | 1 |
| 2597 | 31.73 | 2.55 | GBIF | 11.63 | 67.64 | 22.40 | 24.95 | 156 | 18 | 49.05 | 120 | 448 | 13 |
| 2598 | 31.46 | 2.22 | GBIF | 10.39 | 71.67 | 24.52 | 26.17 | 130 | 18 | 45.57 | 121 | 358 | 21 |
| 2599 | 31.51 | 2.33 | GBIF | 11.34 | 67.91 | 23.72 | 26.07 | 140 | 21 | 45.34 | 134 | 386 | 15 |

| 2600 | 29.94 | -0.24 | GBIF | 11.14 | 87.73 | 22.77 | 22.80 | 126 | 27 | 44.83 | 172 | 299 | 12 |
| --- | --- | --- | --- | --- | --- | --- | --- | --- | --- | --- | --- | --- | --- |
| 2601 | 31.75 | 2.22 | GBIF | 10.97 | 73.11 | 23.37 | 25.33 | 147 | 23 | 44.49 | 144 | 413 | 0 |
| 2602 | 31.31 | 2.34 | GBIF | 11.43 | 66.86 | 22.85 | 25.33 | 140 | 22 | 46.39 | 128 | 400 | 14 |
| 2603 | 31.41 | 2.24 | GBIF | 10.26 | 69.78 | 24.05 | 26.08 | 125 | 18 | 45.44 | 116 | 349 | 6 |
| 2604 | 31.53 | 2.32 | GBIF | 11.33 | 69.11 | 23.73 | 26.03 | 144 | 22 | 44.86 | 145 | 395 | 11 |
| 2605 | 31.51 | 2.52 | GBIF | 10.93 | 60.74 | 22.87 | 25.70 | 130 | 16 | 47.98 | 104 | 371 | 6 |
| 2606 | 31.53 | 2.23 | GBIF | 10.86 | 71.91 | 24.03 | 26.07 | 141 | 21 | 44.86 | 140 | 388 | 14 |
| 2607 | 31.96 | 2.25 | GBIF | 11.45 | 73.87 | 22.50 | 24.48 | 150 | 23 | 46.04 | 142 | 445 | 0 |
| 2608 | 29.97 | -0.14 | GBIF | 11.21 | 88.25 | 23.00 | 23.03 | 111 | 23 | 45.71 | 149 | 278 | 5 |
| 2609 | 31.48 | 2.28 | GBIF | 11.01 | 69.67 | 23.90 | 26.10 | 136 | 19 | 45.77 | 127 | 373 | 7 |
| 2610 | 30.02 | -0.15 | GBIF | 10.91 | 88.69 | 23.02 | 23.03 | 112 | 23 | 45.77 | 149 | 276 |  |
| 2611 | 33.64 | 3.69 | GBIF | 12.02 | 76.05 | 20.68 | 23.25 | 145 | 9 | 60.67 | 90 | 395 | 5 |
| 2612 | 31.43 | 1.96 | GBIF | 10.27 | 80.84 | 24.80 | 26.23 | 139 | 22 | 43.80 | 141 | 307 | 8 |
| 2613 | 31.51 | 2.57 | GBIF | 10.98 | 60.68 | 22.57 | 25.47 | 136 | 16 | 48.89 | 106 | 388 | 0 |
| 2614 | 30.05 | -0.19 | GBIF | 11.01 | 87.37 | 22.67 | 22.62 | 115 | 23 | 45.52 | 158 | 289 | 9 |
| 2615 | 30.18 | -0.08 | GBIF | 10.44 | 88.49 | 23.08 | 23.02 | 122 | 26 | 45.59 | 159 | 281 | 9 |
| 2616 | 31.49 | 0.63 | GBIF | 10.34 | 81.43 | 21.38 | 22.28 | 151 | 43 | 42.48 | 194 | 286 | 6 |
| 2617 | 29.92 | -0.34 | GBIF | 10.36 | 90.07 | 22.25 | 22.13 | 120 | 25 | 43.40 | 174 | 162 | 4 |
| 2618 | 29.93 | 0.00 | GBIF | 11.17 | 86.56 | 22.10 | 22.20 | 137 | 39 | 40.40 | 289 | 331 | 11 |
| 2619 | 31.62 | 2.25 | GBIF | 10.64 | 72.39 | 23.90 | 25.88 | 144 | 20 | 44.99 | 138 | 394 | 1 |
| 2620 | 31.70 | 1.95 | GBIF | 10.45 | 81.01 | 22.93 | 24.38 | 151 | 25 | 43.83 | 152 | 360 | 7 |
| 2621 | 31.51 | 2.53 | GBIF | 11.10 | 60.66 | 22.80 | 25.70 | 131 | 16 | 48.31 | 104 | 373 | 3 |
| 2622 | 32.38 | 1.29 | GBIF | 11.17 | 81.51 | 23.00 | 23.27 | 156 | 27 | 43.22 | 159 | 257 | 5 |
| 2623 | 32.26 | 1.39 | GBIF | 11.39 | 81.37 | 23.33 | 23.65 | 162 | 28 | 43.57 | 168 | 328 | 15 |
| 2624 | 29.97 | -0.08 | GBIF | 11.10 | 87.40 | 22.67 | 22.70 | 115 | 26 | 44.25 | 244 | 293 | 5 |
| 2625 | 29.98 | -0.31 | GBIF | 10.32 | 90.50 | 22.42 | 22.27 | 119 | 24 | 45.13 | 253 | 161 | 4 |
| 2626 | 29.73 | -0.43 | GBIF | 10.16 | 91.52 | 22.95 | 22.78 | 107 | 28 | 40.24 | 166 | 147 |  |
| 2627 | 31.93 | 2.06 | GBIF | 11.01 | 78.63 | 22.43 | 24.05 | 154 | 24 | 44.76 | 148 | 388 | 0 |
| 2628 | 31.79 | 2.22 | GBIF | 11.20 | 73.20 | 23.37 | 25.33 | 148 | 23 | 44.76 | 144 | 420 | 0 |
| 2629 | 29.92 | -0.15 | GBIF | 11.02 | 88.20 | 22.90 | 22.93 | 113 | 23 | 45.48 | 151 | 282 | 9 |
| 2630 | 29.82 | -0.19 | GBIF | 9.17 | 91.67 | 23.05 | 22.88 | 111 | 25 | 44.99 | 234 | 122 |  |
| 2631 | 29.92 | -0.11 | GBIF | 11.41 | 87.76 | 22.77 | 22.82 | 127 | 28 | 45.35 | 261 | 300 | 10 |
| 2632 | 33.70 | 3.85 | GBIF | 13.35 | 78.53 | 22.28 | 24.87 | 117 | 7 | 56.63 | 82 | 299 | 3 |
| 2633 | 31.77 | 2.10 | GBIF | 10.78 | 77.02 | 23.92 | 25.58 | 149 | 24 | 44.10 | 148 | 399 | 0 |
| 2634 | 31.74 | 2.24 | GBIF | 11.02 | 72.96 | 23.62 | 25.62 | 147 | 22 | 44.70 | 142 | 410 | 0 |
| 2635 | 31.61 | 2.28 | GBIF | 10.69 | 71.76 | 24.07 | 26.12 | 143 | 20 | 44.90 | 142 | 391 | 0 |
| 2636 | 31.75 | 2.39 | GBIF | 11.61 | 69.51 | 22.97 | 25.25 | 151 | 20 | 46.57 | 131 | 427 | 15 |
| 2637 | 31.72 | 2.34 | GBIF | 11.29 | 69.70 | 23.25 | 25.48 | 149 | 20 | 45.96 | 133 | 417 | 15 |
| 2638 | 31.77 | 2.35 | GBIF | 11.47 | 71.22 | 23.42 | 25.58 | 147 | 21 | 45.94 | 135 | 419 | 7 |
| 2639 | 31.73 | 2.20 | GBIF | 10.90 | 73.65 | 23.62 | 25.52 | 147 | 23 | 44.67 | 144 | 410 | 1 |
| 2640 | 31.67 | 2.30 | GBIF | 11.01 | 70.57 | 23.60 | 25.73 | 146 | 20 | 45.41 | 136 | 402 | 4 |
| 2641 | 31.56 | 2.29 | GBIF | 10.85 | 70.45 | 24.18 | 26.32 | 141 | 20 | 45.37 | 141 | 386 | 9 |
| 2642 | 30.11 | -0.05 | GBIF | 11.67 | 87.06 | 23.15 | 23.23 | 121 | 26 | 45.15 | 156 | 283 | 2 |
| 2643 | 30.12 | 0.00 | GBIF | 11.13 | 88.36 | 23.15 | 23.17 | 123 | 27 | 45.66 | 152 | 279 | 6 |
| 2644 | 30.07 | -0.14 | GBIF | 11.01 | 88.78 | 22.90 | 22.92 | 113 | 24 | 44.90 | 154 | 282 | 11 |
| 2645 | 31.60 | 2.23 | GBIF | 10.77 | 72.26 | 23.85 | 25.85 | 145 | 21 | 44.47 | 141 | 395 | 10 |
| 2646 | 29.94 | 0.00 | GBIF | 11.09 | 86.65 | 21.88 | 21.95 | 135 | 37 | 40.07 | 203 | 339 | 10 |
| 2647 | 29.98 | -0.18 | GBIF | 10.83 | 88.80 | 22.90 | 22.92 | 111 | 23 | 45.46 | 156 | 283 |  |
| 2648 | 30.04 | -0.09 | GBIF | 11.55 | 87.50 | 23.03 | 23.08 | 118 | 25 | 45.46 | 242 | 282 | 9 |
| 2649 | 29.85 | -0.11 | GBIF | 11.62 | 86.69 | 23.05 | 23.13 | 115 | 25 | 44.95 | 238 | 284 | 8 |

| 2650 | 29.74 | -0.45 | GBIF | 10.90 | 90.08 | 22.92 | 22.77 | 108 | 28 | 39.95 | 166 | 149 | 4 |
| --- | --- | --- | --- | --- | --- | --- | --- | --- | --- | --- | --- | --- | --- |
| 2651 | 31.55 | 2.34 | GBIF | 11.33 | 68.27 | 23.65 | 25.97 | 146 | 21 | 45.27 | 143 | 401 | 15 |
| 2652 | 33.73 | 3.63 | GBIF | 12.93 | 76.53 | 21.08 | 23.70 | 136 | 8 | 59.52 | 90 | 349 | 11 |
| 2653 | 31.50 | 2.30 | GBIF | 11.22 | 69.24 | 23.77 | 26.03 | 142 | 20 | 45.78 | 136 | 390 | 9 |
| 2654 | 31.41 | 2.36 | GBIF | 10.94 | 65.52 | 23.65 | 26.07 | 129 | 20 | 46.33 | 117 | 375 | 22 |
| 2655 | 29.87 | -0.33 | GBIF | 10.13 | 90.40 | 22.75 | 22.60 | 112 | 25 | 43.14 | 160 | 154 | 8 |
| 2656 | 30.17 | -0.10 | GBIF | 10.89 | 87.84 | 22.97 | 22.93 | 123 | 27 | 44.16 | 164 | 286 | 8 |
| 2657 | 31.57 | 2.39 | GBIF | 11.40 | 65.52 | 23.67 | 26.18 | 134 | 18 | 46.19 | 123 | 376 | 9 |
| 2658 | 33.77 | 3.69 | GBIF | 12.77 | 76.91 | 21.08 | 23.72 | 130 | 7 | 60.24 | 82 | 329 | 7 |
| 2659 | 31.83 | 0.73 | GBIF | 10.38 | 81.76 | 21.33 | 21.98 | 151 | 34 | 43.17 | 174 | 204 | 11 |
| 2660 | 31.92 | 0.69 | GBIF | 10.49 | 81.97 | 22.13 | 22.30 | 150 | 36 | 40.75 | 188 | 209 | 7 |
| 2661 | 33.60 | 3.64 | GBIF | 11.35 | 75.17 | 20.28 | 22.87 | 154 | 8 | 64.64 | 84 | 390 | 4 |
| 2662 | 31.40 | 2.23 | GBIF | 9.68 | 70.17 | 24.02 | 25.97 | 124 | 18 | 45.48 | 115 | 347 | 7 |
| 2663 | 31.45 | 2.22 | GBIF | 10.42 | 71.84 | 24.50 | 26.15 | 130 | 19 | 44.94 | 125 | 358 | 10 |
| 2664 | 31.43 | 2.35 | GBIF | 10.79 | 65.80 | 23.70 | 26.10 | 126 | 20 | 45.25 | 122 | 367 | 18 |
| 2665 | 29.98 | -0.15 | GBIF | 11.13 | 88.36 | 22.97 | 22.98 | 113 | 23 | 45.63 | 150 | 281 | 7 |
| 2666 | 31.74 | 2.30 | GBIF | 11.22 | 71.90 | 23.78 | 25.85 | 147 | 22 | 44.52 | 147 | 412 | 0 |
| 2667 | 31.64 | 2.20 | GBIF | 10.79 | 72.92 | 23.62 | 25.58 | 147 | 23 | 44.41 | 144 | 402 | 2 |
| 2668 | 31.68 | 2.36 | GBIF | 11.18 | 68.56 | 23.50 | 25.77 | 146 | 20 | 45.65 | 133 | 404 | 1 |
| 2669 | 31.00 | 0.50 | GBIF | 10.33 | 82.67 | 20.83 | 21.58 | 158 | 43 | 43.43 | 191 | 292 | 14 |
| 2670 | 32.10 | 1.27 | GBIF | 11.21 | 81.81 | 23.20 | 23.45 | 169 | 31 | 43.43 | 180 | 328 | 14 |
| 2671 | 32.17 | 1.98 | GBIF | 12.19 | 78.66 | 22.07 | 23.80 | 154 | 24 | 44.33 | 151 | 398 | 22 |
| 2672 | 31.80 | 2.32 | GBIF | 11.32 | 71.62 | 23.53 | 25.65 | 145 | 22 | 45.12 | 139 | 415 | 0 |
| 2673 | 31.61 | 2.26 | GBIF | 10.59 | 72.55 | 24.10 | 26.07 | 141 | 19 | 45.36 | 135 | 384 | 0 |
| 2674 | 31.73 | 2.26 | GBIF | 10.97 | 73.11 | 23.67 | 25.63 | 146 | 22 | 44.71 | 142 | 408 | 1 |
| 2675 | 29.93 | -0.06 | GBIF | 11.06 | 87.07 | 22.08 | 22.13 | 132 | 31 | 43.03 | 283 | 331 | 5 |
| 2676 | 29.90 | -0.10 | GBIF | 11.41 | 87.09 | 22.72 | 22.77 | 133 | 29 | 45.09 | 272 | 311 | 6 |
| 2677 | 29.89 | -0.13 | GBIF | 11.08 | 88.67 | 23.08 | 23.13 | 112 | 24 | 45.30 | 232 | 275 | 8 |
| 2678 | 31.65 | 2.38 | GBIF | 11.31 | 66.91 | 23.38 | 25.77 | 145 | 19 | 46.33 | 129 | 400 | 4 |
| 2679 | 31.63 | 2.25 | GBIF | 10.61 | 72.66 | 23.92 | 25.90 | 144 | 20 | 45.06 | 138 | 395 | 1 |
| 2680 | 30.01 | -0.06 | GBIF | 11.36 | 87.37 | 22.92 | 22.93 | 119 | 25 | 44.91 | 244 | 284 | 8 |
| 2681 | 29.64 | -0.61 | GBIF | 11.59 | 89.86 | 22.93 | 23.02 | 117 | 35 | 35.20 | 186 | 298 | 9 |
| 2682 | 29.87 | -0.03 | GBIF | 11.59 | 87.16 | 22.58 | 22.87 | 146 | 38 | 42.76 | 292 | 322 | 7 |
| 2683 | 30.00 | -0.24 | GBIF | 11.16 | 87.17 | 22.60 | 22.58 | 120 | 25 | 44.55 | 173 | 299 | 4 |
| 2684 | 33.16 | -0.31 | GBIF | 7.73 | 83.06 | 22.42 | 21.47 | 260 | 58 | 48.76 | 406 | 221 |  |
| 2685 | 29.82 | -0.39 | GBIF | 10.39 | 91.15 | 22.83 | 22.67 | 110 | 24 | 43.31 | 160 | 150 |  |
| 2686 | 30.03 | -0.26 | GBIF | 10.72 | 87.84 | 22.58 | 22.45 | 118 | 24 | 45.17 | 163 | 161 | 5 |
| 2687 | 29.98 | -0.32 | GBIF | 10.24 | 90.63 | 22.37 | 22.22 | 121 | 24 | 45.06 | 257 | 165 | 4 |
| 2688 | 29.98 | -0.37 | GBIF | 10.13 | 90.48 | 22.22 | 22.07 | 123 | 25 | 44.45 | 177 | 167 | 3 |
| 2689 | 29.98 | -0.30 | GBIF | 10.30 | 89.57 | 22.43 | 22.30 | 118 | 23 | 45.56 | 168 | 159 | 4 |
| 2690 | 30.04 | -0.27 | GBIF | 10.51 | 88.31 | 22.52 | 22.40 | 118 | 24 | 45.31 | 251 | 164 | 5 |
| 2691 | 29.98 | -0.34 | GBIF | 10.24 | 90.63 | 22.33 | 22.22 | 121 | 25 | 44.99 | 172 | 167 | 4 |
| 2692 | 29.96 | -0.34 | GBIF | 10.31 | 90.42 | 22.40 | 22.27 | 119 | 25 | 44.83 | 253 | 163 | 4 |
| 2693 | 29.96 | -0.44 | GBIF | 10.36 | 90.86 | 21.87 | 21.77 | 132 | 26 | 43.86 | 191 | 166 | 6 |
| 2694 | 29.96 | -0.40 | GBIF | 10.24 | 90.63 | 22.18 | 22.05 | 126 | 25 | 44.35 | 178 | 175 | 4 |
| 2695 | 30.01 | -0.37 | GBIF | 10.07 | 90.69 | 21.92 | 21.82 | 129 | 27 | 43.90 | 187 | 177 | 2 |
| 2696 | 29.96 | -0.35 | GBIF | 10.27 | 90.86 | 22.37 | 22.22 | 120 | 25 | 44.21 | 172 | 162 | 4 |
| 2697 | 29.94 | -0.38 | GBIF | 10.32 | 90.50 | 22.23 | 22.07 | 123 | 25 | 44.63 | 175 | 164 | 4 |
| 2698 | 29.94 | -0.34 | GBIF | 10.38 | 90.22 | 22.35 | 22.18 | 119 | 24 | 44.00 | 169 | 159 | 4 |
| 2699 | 29.94 | -0.48 | GBIF | 10.46 | 90.16 | 21.90 | 21.77 | 131 | 27 | 43.04 | 190 | 168 | 6 |

| 2700 | 29.96 | -0.39 | GBIF | 10.22 | 90.41 | 22.22 | 22.08 | 125 | 25 | 44.71 | 175 | 170 | 3 |
| --- | --- | --- | --- | --- | --- | --- | --- | --- | --- | --- | --- | --- | --- |
| 2701 | 29.94 | -0.47 | GBIF | 10.38 | 91.08 | 21.87 | 21.75 | 130 | 26 | 43.08 | 190 | 165 | 6 |
| 2702 | 29.92 | -0.39 | GBIF | 10.33 | 90.64 | 22.33 | 22.17 | 121 | 24 | 44.81 | 174 | 162 | 4 |
| 2703 | 29.92 | -0.40 | GBIF | 10.31 | 90.42 | 22.10 | 21.97 | 126 | 25 | 43.58 | 183 | 165 | 4 |
| 2704 | 29.89 | -0.43 | GBIF | 10.32 | 90.50 | 22.28 | 22.15 | 123 | 26 | 43.88 | 176 | 161 | 4 |
| 2705 | 29.92 | -0.35 | GBIF | 10.37 | 90.14 | 22.25 | 22.13 | 120 | 24 | 43.52 | 172 | 160 | 4 |
| 2706 | 29.89 | -0.38 | GBIF | 10.34 | 90.72 | 22.22 | 22.08 | 123 | 26 | 43.34 | 178 | 164 | 4 |
| 2707 | 29.89 | -0.42 | GBIF | 10.33 | 90.57 | 22.28 | 22.15 | 122 | 25 | 44.22 | 176 | 162 | 4 |
| 2708 | 29.89 | -0.47 | GBIF | 10.28 | 91.00 | 22.03 | 21.88 | 126 | 26 | 42.27 | 185 | 160 | 2 |
| 2709 | 29.87 | -0.38 | GBIF | 10.40 | 90.43 | 22.70 | 22.52 | 119 | 24 | 44.13 | 163 | 161 | 12 |
| 2710 | 29.87 | -0.35 | GBIF | 10.28 | 90.20 | 22.67 | 22.53 | 115 | 24 | 43.88 | 161 | 158 | 12 |
| 2711 | 29.87 | -0.39 | GBIF | 10.43 | 89.94 | 22.70 | 22.52 | 119 | 24 | 44.33 | 165 | 159 | 12 |
| 2712 | 29.87 | -0.43 | GBIF | 10.50 | 89.74 | 22.52 | 22.37 | 119 | 24 | 43.92 | 170 | 156 | 6 |
| 2713 | 29.87 | -0.40 | GBIF | 10.47 | 90.23 | 22.62 | 22.48 | 120 | 24 | 44.74 | 165 | 154 | 12 |
| 2714 | 29.87 | -0.44 | GBIF | 10.48 | 90.30 | 22.42 | 22.28 | 119 | 24 | 43.67 | 170 | 157 | 4 |
| 2715 | 29.87 | -0.48 | GBIF | 10.39 | 91.15 | 22.20 | 22.07 | 124 | 26 | 42.73 | 181 | 159 | 1 |
| 2716 | 29.87 | -0.45 | GBIF | 10.44 | 90.80 | 22.30 | 22.17 | 121 | 25 | 42.85 | 175 | 154 | 3 |
| 2717 | 29.85 | -0.38 | GBIF | 10.48 | 91.09 | 22.72 | 22.57 | 113 | 24 | 43.61 | 161 | 155 | 5 |
| 2718 | 29.85 | -0.49 | GBIF | 10.50 | 91.30 | 22.23 | 22.10 | 122 | 27 | 41.90 | 181 | 160 | 3 |
| 2719 | 29.87 | -0.54 | GBIF | 10.33 | 91.45 | 22.03 | 21.87 | 126 | 26 | 41.60 | 185 | 155 | 3 |
| 2720 | 29.85 | -0.39 | GBIF | 10.47 | 90.23 | 22.72 | 22.55 | 114 | 24 | 43.89 | 163 | 154 | 5 |
| 2721 | 29.85 | -0.51 | GBIF | 10.49 | 90.45 | 22.22 | 22.08 | 122 | 27 | 41.39 | 181 | 161 | 4 |
| 2722 | 29.85 | -0.47 | GBIF | 10.54 | 90.88 | 22.40 | 22.23 | 118 | 26 | 42.31 | 175 | 158 | 4 |
| 2723 | 29.85 | -0.60 | GBIF | 10.91 | 91.67 | 22.07 | 21.97 | 126 | 29 | 40.62 | 189 | 156 | 8 |
| 2724 | 29.85 | -0.46 | GBIF | 10.51 | 90.59 | 22.42 | 22.22 | 119 | 26 | 42.46 | 174 | 160 | 4 |
| 2725 | 29.85 | -0.53 | GBIF | 10.41 | 91.30 | 21.95 | 21.80 | 127 | 28 | 40.78 | 189 | 163 | 4 |
| 2726 | 29.85 | -0.57 | GBIF | 10.60 | 90.60 | 21.83 | 21.67 | 129 | 28 | 40.84 | 191 | 156 | 6 |
| 2727 | 30.27 | -0.16 | GBIF | 10.39 | 88.06 | 22.17 | 22.07 | 130 | 27 | 44.61 | 181 | 317 | 6 |
| 2728 | 30.27 | -0.15 | GBIF | 10.51 | 87.57 | 22.53 | 22.45 | 127 | 26 | 45.18 | 263 | 297 | 6 |
| 2729 | 29.90 | -0.52 | GBIF | 10.30 | 90.35 | 22.05 | 21.85 | 126 | 25 | 42.80 | 184 | 160 | 1 |
| 2730 | 30.01 | -0.05 | GBIF | 11.45 | 87.40 | 22.93 | 23.00 | 120 | 25 | 45.16 | 245 | 283 | 13 |
| 2731 | 30.15 | -0.15 | GBIF | 11.01 | 86.68 | 22.72 | 22.68 | 128 | 26 | 44.36 | 177 | 299 | 7 |
| 2732 | 31.93 | 2.18 | GBIF | 11.22 | 75.28 | 22.57 | 24.40 | 154 | 23 | 45.68 | 144 | 439 | 0 |
| 2733 | 36.80 | 3.20 | GBIF | 12.50 | 81.17 | 28.43 | 26.83 | 43 | 0 | 87.01 | 48 | 11 | 5 |
| 2734 | 32.80 | 6.80 | GBIF | 13.38 | 70.77 | 25.98 | 28.42 | 147 | 6 | 71.86 | 112 | 417 | 3 |
| 2735 | 31.70 | 7.20 | GBIF | 13.45 | 69.33 | 26.25 | 28.35 | 160 | 1 | 83.89 | 81 | 433 | 4 |
| 2736 | 37.20 | 3.00 | GBIF | 11.84 | 78.42 | 27.93 | 26.00 | 53 | 0 | 97.03 | 47 | 7 | 2 |
| 2737 | 38.30 | 4.20 | GBIF | 12.32 | 72.08 | 21.00 | 19.00 | 145 | 9 | 84.58 | 117 | 35 | 11 |
| 2738 | 31.70 | 5.20 | GBIF | 13.65 | 76.26 | 25.90 | 28.38 | 145 | 5 | 69.08 | 157 | 399 | 9 |
| 2739 | 36.70 | 2.60 | GBIF | 10.72 | 82.44 | 27.85 | 26.12 | 57 | 1 | 84.95 | 92 | 20 |  |
| 2740 | 33.30 | 7.10 | GBIF | 12.80 | 68.82 | 25.70 | 27.30 | 158 | 7 | 71.35 | 110 | 441 | 3 |
| 2741 | 34.30 | 6.90 | GBIF | 14.22 | 71.80 | 24.85 | 26.02 | 172 | 16 | 57.50 | 176 | 459 | 6 |
| 2742 | 31.80 | 2.30 | GBIF | 11.33 | 71.73 | 23.27 | 25.40 | 149 | 22 | 45.27 | 139 | 424 | 0 |
| 2743 | 35.00 | 2.50 | GBIF | 11.92 | 84.52 | 23.27 | 24.33 | 79 | 9 | 55.08 | 139 | 180 | 5 |
| 2744 | 34.80 | 2.60 | GBIF | 11.19 | 81.10 | 21.07 | 22.58 | 105 | 10 | 56.84 | 89 | 244 | 12 |
| 2745 | 36.20 | 4.00 | GBIF | 11.03 | 81.13 | 29.47 | 28.80 | 54 | 2 | 79.11 | 64 | 21 |  |
| 2746 | 34.09 | -2.28 | GBIF | 11.88 | 78.18 | 22.80 | 22.02 | 143 | 6 | 62.74 | 173 | 58 | 8 |
| 2747 | 35.60 | -3.51 | GBIF | 11.16 | 71.07 | 21.53 | 18.43 | 150 | 0 | 91.45 | 357 | 5 | 8 |
| 2748 | 35.13 | -1.88 | GBIF | 12.17 | 79.52 | 20.17 | 18.47 | 157 | 22 | 52.55 | 372 | 94 | 4 |
| 2749 | 35.61 | -3.00 | GBIF | 8.46 | 64.57 | 12.25 | 9.37 | 247 | 7 | 76.64 | 518 | 34 | 4 |

| 2750 | 35.97 | -3.43 | GBIF | 11.82 | 72.10 | 23.45 | 20.42 | 153 | 0 | 94.72 | 318 | 6 | 5 |
| --- | --- | --- | --- | --- | --- | --- | --- | --- | --- | --- | --- | --- | --- |
| 2751 | 34.97 | -2.24 | GBIF | 11.95 | 77.60 | 20.97 | 18.82 | 134 | 18 | 56.42 | 304 | 74 | 6 |
| 2752 | 35.04 | -1.98 | GBIF | 12.02 | 79.64 | 20.47 | 18.60 | 155 | 20 | 54.01 | 373 | 87 | 6 |
| 2753 | 34.99 | -2.61 | GBIF | 11.95 | 75.63 | 19.97 | 17.42 | 129 | 9 | 69.66 | 321 | 39 | 8 |
| 2754 | 36.81 | -7.85 | GBIF | 7.49 | 60.91 | 22.27 | 19.47 | 319 | 4 | 90.05 | 513 | 49 | 4 |
| 2755 | 37.29 | -6.99 | GBIF | 10.42 | 66.35 | 26.02 | 22.48 | 226 | 7 | 82.38 | 364 | 34 | 9 |
| 2756 | 35.63 | -3.47 | GBIF | 11.43 | 70.96 | 21.87 | 18.80 | 171 | 1 | 90.43 | 373 | 12 | 10 |
| 2757 | 37.28 | -7.17 | GBIF | 9.84 | 64.32 | 25.32 | 21.63 | 270 | 5 | 86.30 | 380 | 34 | 9 |
| 2758 | 35.93 | -3.94 | GBIF | 10.90 | 71.24 | 23.08 | 20.00 | 145 | 1 | 90.31 | 336 | 10 | 6 |
| 2759 | 35.54 | -3.36 | GBIF | 10.75 | 68.91 | 20.18 | 17.85 | 224 | 5 | 90.51 | 450 | 27 | 15 |
| 2760 | 35.58 | -3.28 | GBIF | 9.57 | 65.08 | 18.42 | 15.93 | 253 | 8 | 87.49 | 500 | 40 | 8 |
| 2761 | 37.38 | -6.84 | GBIF | 10.41 | 66.30 | 26.37 | 22.83 | 181 | 7 | 78.16 | 363 | 30 | 6 |
| 2762 | 34.96 | -7.73 | GBIF | 11.63 | 67.59 | 23.67 | 20.73 | 133 | 0 | 106.23 | 155 | 0 | 6 |
| 2763 | 36.05 | -2.90 | GBIF | 12.81 | 74.04 | 25.07 | 21.80 | 94 | 0 | 95.19 | 161 | 0 | 6 |
| 2764 | 34.92 | -2.41 | GBIF | 11.99 | 76.38 | 21.15 | 18.87 | 131 | 16 | 60.01 | 302 | 66 | 4 |
| 2765 | 36.16 | -3.74 | GBIF | 11.25 | 70.31 | 22.23 | 18.97 | 170 | 1 | 93.82 | 363 | 9 | 4 |
| 2766 | 36.17 | -4.07 | GBIF | 11.42 | 71.86 | 22.48 | 19.40 | 141 | 1 | 90.70 | 238 | 8 | 7 |
| 2767 | 34.93 | -1.44 | GBIF | 12.57 | 81.60 | 20.53 | 18.98 | 176 | 34 | 40.95 | 305 | 156 | 5 |
| 2768 | 35.15 | -2.50 | GBIF | 11.94 | 76.06 | 19.77 | 17.17 | 122 | 11 | 68.88 | 311 | 39 | 5 |
| 2769 | 36.05 | -4.20 | GBIF | 11.61 | 71.22 | 23.03 | 20.00 | 138 | 1 | 90.06 | 241 | 10 | 8 |
| 2770 | 36.20 | -4.08 | GBIF | 11.40 | 71.70 | 22.45 | 19.42 | 152 | 1 | 92.36 | 238 | 8 | 8 |
| 2771 | 36.19 | -4.04 | GBIF | 11.44 | 71.51 | 22.42 | 19.35 | 154 | 1 | 92.82 | 274 | 8 | 8 |
| 2772 | 35.41 | -3.22 | GBIF | 9.73 | 66.67 | 14.83 | 12.60 | 201 | 3 | 81.38 | 312 | 20 | 3 |
| 2773 | 34.95 | -2.69 | GBIF | 11.89 | 75.26 | 20.07 | 17.47 | 131 | 8 | 71.32 | 324 | 35 | 7 |
| 2774 | 35.51 | -3.12 | GBIF | 10.08 | 67.67 | 17.58 | 14.83 | 194 | 5 | 83.93 | 434 | 22 | 4 |
| 2775 | 35.51 | -3.17 | GBIF | 12.10 | 70.35 | 19.72 | 16.85 | 149 | 3 | 84.34 | 369 | 17 | 4 |
| 2776 | 35.88 | -3.66 | GBIF | 10.40 | 67.53 | 23.53 | 19.93 | 169 | 3 | 88.38 | 363 | 18 | 6 |
| 2777 | 34.94 | -2.36 | GBIF | 12.14 | 77.34 | 21.12 | 18.93 | 128 | 16 | 58.33 | 300 | 69 | 6 |
| 2778 | 34.91 | -2.24 | GBIF | 12.03 | 77.63 | 21.45 | 19.38 | 134 | 19 | 54.90 | 343 | 80 | 5 |
| 2779 | 35.89 | -3.65 | GBIF | 11.13 | 69.15 | 23.58 | 20.17 | 152 | 2 | 90.48 | 330 | 12 | 6 |
| 2780 | 31.26 | -6.89 | GBIF | 12.32 | 68.05 | 25.80 | 23.75 | 199 | 0 | 96.96 | 133 | 1 | 3 |
| 2781 | 34.87 | -7.71 | GBIF | 11.84 | 68.06 | 23.70 | 20.67 | 135 | 0 | 106.98 | 157 | 0 | 12 |
| 2782 | 36.17 | -4.00 | GBIF | 11.38 | 71.59 | 22.37 | 19.28 | 151 | 1 | 92.32 | 276 | 8 | 7 |
| 2783 | 36.18 | -3.90 | GBIF | 11.18 | 71.23 | 22.15 | 19.05 | 162 | 1 | 93.99 | 272 | 7 | 7 |
| 2784 | 36.00 | -4.08 | GBIF | 11.22 | 70.99 | 22.67 | 19.57 | 138 | 1 | 91.39 | 339 | 8 | 7 |
| 2785 | 35.62 | -3.65 | GBIF | 10.29 | 68.16 | 19.83 | 16.70 | 195 | 1 | 91.35 | 426 | 11 | 6 |
| 2786 | 36.02 | -4.12 | GBIF | 11.32 | 71.17 | 22.52 | 19.48 | 145 | 0 | 92.95 | 249 | 7 | 7 |
| 2787 | 35.08 | -2.43 | GBIF | 11.93 | 76.01 | 20.27 | 17.78 | 129 | 13 | 65.11 | 310 | 50 | 4 |
| 2788 | 35.90 | -3.62 | GBIF | 11.03 | 69.39 | 23.52 | 20.12 | 148 | 1 | 89.88 | 324 | 11 | 7 |
| 2789 | 34.84 | -2.56 | GBIF | 12.02 | 75.10 | 20.55 | 18.17 | 132 | 12 | 64.31 | 320 | 52 | 7 |
| 2790 | 34.64 | -1.98 | GBIF | 11.88 | 80.24 | 21.33 | 19.93 | 162 | 20 | 49.11 | 302 | 105 | 4 |
| 2791 | 35.73 | -3.65 | GBIF | 8.96 | 66.36 | 20.82 | 17.43 | 196 | 1 | 94.90 | 410 | 11 | 7 |
| 2792 | 34.60 | -1.82 | GBIF | 11.52 | 80.54 | 20.87 | 19.53 | 184 | 24 | 47.08 | 319 | 124 | 6 |
| 2793 | 35.67 | -3.61 | GBIF | 9.79 | 67.53 | 19.65 | 16.55 | 208 | 1 | 93.10 | 442 | 10 | 13 |
| 2794 | 34.68 | -1.90 | GBIF | 11.66 | 79.85 | 20.72 | 19.27 | 176 | 23 | 48.67 | 318 | 114 | 6 |
| 2795 | 36.09 | -3.97 | GBIF | 11.46 | 70.73 | 23.00 | 19.80 | 144 | 1 | 91.68 | 331 | 8 | 8 |
| 2796 | 34.80 | -2.46 | GBIF | 12.13 | 76.79 | 21.05 | 18.92 | 131 | 14 | 60.53 | 310 | 62 | 13 |
| 2797 | 36.20 | -3.84 | GBIF | 11.42 | 71.35 | 22.12 | 19.03 | 163 | 1 | 93.47 | 273 | 8 | 6 |
| 2798 | 36.19 | -4.18 | GBIF | 11.58 | 71.95 | 22.63 | 19.70 | 137 | 1 | 88.54 | 249 | 10 | 7 |
| 2799 | 34.89 | -2.58 | GBIF | 11.98 | 75.37 | 20.32 | 17.88 | 130 | 11 | 66.58 | 320 | 47 | 7 |

| 2800 | 34.85 | -2.58 | GBIF | 11.99 | 75.42 | 20.43 | 18.03 | 134 | 12 | 64.82 | 326 | 51 | 7 |
| --- | --- | --- | --- | --- | --- | --- | --- | --- | --- | --- | --- | --- | --- |
| 2801 | 35.88 | -3.72 | GBIF | 11.46 | 69.87 | 23.58 | 20.23 | 155 | 2 | 89.23 | 340 | 14 | 15 |
| 2802 | 35.00 | -2.43 | GBIF | 11.88 | 76.67 | 20.57 | 18.18 | 125 | 14 | 62.43 | 310 | 57 | 4 |
| 2803 | 34.95 | -2.42 | GBIF | 11.91 | 76.34 | 20.93 | 18.58 | 131 | 15 | 61.48 | 305 | 62 | 4 |
| 2804 | 35.52 | -3.27 | GBIF | 7.95 | 60.69 | 15.07 | 12.68 | 266 | 14 | 76.45 | 512 | 69 | 4 |
| 2805 | 34.92 | -2.44 | GBIF | 12.07 | 76.42 | 20.80 | 18.53 | 130 | 15 | 61.69 | 311 | 61 | 4 |
| 2806 | 36.54 | -7.76 | GBIF | 8.70 | 65.41 | 18.22 | 15.63 | 247 | 6 | 85.75 | 459 | 45 | 4 |
| 2807 | 35.58 | -1.99 | GBIF | 11.65 | 78.72 | 16.13 | 13.88 | 192 | 18 | 67.77 | 430 | 60 | 4 |
| 2808 | 35.89 | -3.27 | GBIF | 11.07 | 70.04 | 21.33 | 18.33 | 186 | 2 | 94.18 | 273 | 10 | 6 |
| 2809 | 34.92 | -1.83 | GBIF | 11.94 | 80.15 | 20.02 | 18.48 | 172 | 25 | 48.78 | 318 | 118 | 7 |
| 2810 | 35.52 | -3.07 | GBIF | 9.89 | 68.69 | 18.78 | 15.67 | 166 | 1 | 90.83 | 396 | 5 | 4 |
| 2811 | 34.61 | -1.86 | GBIF | 11.50 | 79.86 | 21.08 | 19.73 | 182 | 22 | 48.53 | 312 | 118 | 6 |
| 2812 | 34.88 | -2.26 | GBIF | 12.15 | 77.88 | 21.50 | 19.42 | 135 | 20 | 53.90 | 345 | 84 | 5 |
| 2813 | 35.72 | -3.70 | GBIF | 9.16 | 66.85 | 21.48 | 18.05 | 173 | 2 | 91.50 | 375 | 15 | 14 |
| 2814 | 34.90 | -2.41 | GBIF | 11.98 | 76.27 | 21.15 | 18.87 | 131 | 16 | 59.62 | 303 | 66 | 5 |
| 2815 | 34.86 | -2.41 | GBIF | 11.91 | 76.34 | 21.18 | 18.97 | 133 | 16 | 58.86 | 306 | 69 | 9 |
| 2816 | 34.63 | -2.35 | GBIF | 12.14 | 78.84 | 22.10 | 20.37 | 131 | 14 | 56.69 | 294 | 72 | 6 |
| 2817 | 34.97 | -2.42 | GBIF | 11.84 | 76.40 | 20.70 | 18.33 | 131 | 15 | 62.11 | 309 | 60 | 4 |
| 2818 | 35.88 | -3.77 | GBIF | 11.89 | 71.64 | 23.62 | 20.48 | 154 | 2 | 87.73 | 341 | 14 | 11 |
| 2819 | 34.84 | -2.45 | GBIF | 12.18 | 76.09 | 21.08 | 18.87 | 133 | 14 | 60.94 | 307 | 63 | 7 |
| 2820 | 35.93 | -3.77 | GBIF | 11.55 | 71.30 | 23.35 | 20.20 | 154 | 2 | 88.92 | 341 | 13 | 9 |
| 2821 | 36.07 | -3.77 | GBIF | 11.48 | 70.88 | 22.95 | 19.73 | 158 | 1 | 92.13 | 341 | 10 | 6 |
| 2822 | 35.90 | -3.71 | GBIF | 11.93 | 70.61 | 23.67 | 20.32 | 146 | 1 | 90.99 | 321 | 11 | 11 |
| 2823 | 36.11 | -4.18 | GBIF | 11.67 | 71.14 | 22.58 | 19.62 | 143 | 1 | 91.66 | 247 | 8 | 7 |
| 2824 | 36.02 | -3.70 | GBIF | 11.96 | 70.76 | 23.37 | 20.13 | 155 | 1 | 93.06 | 333 | 9 | 7 |
| 2825 | 35.22 | -1.84 | GBIF | 12.18 | 80.15 | 19.63 | 17.58 | 164 | 21 | 56.75 | 385 | 84 | 14 |
| 2826 | 35.89 | -3.78 | GBIF | 11.63 | 70.93 | 23.50 | 20.32 | 156 | 2 | 87.26 | 346 | 15 | 21 |
| 2827 | 34.98 | -7.77 | GBIF | 11.18 | 67.73 | 23.38 | 20.50 | 135 | 0 | 105.93 | 158 | 0 | 5 |
| 2828 | 35.56 | -3.07 | GBIF | 8.88 | 65.74 | 16.32 | 13.60 | 213 | 3 | 86.62 | 451 | 15 | 4 |
| 2829 | 35.80 | -3.62 | GBIF | 9.52 | 66.15 | 23.45 | 19.80 | 130 | 1 | 88.85 | 307 | 10 | 0 |
| 2830 | 36.08 | -3.72 | GBIF | 11.72 | 71.01 | 22.73 | 19.48 | 163 | 1 | 93.04 | 351 | 10 | 5 |
| 2831 | 34.46 | -1.68 | GBIF | 11.51 | 81.04 | 21.83 | 20.57 | 189 | 26 | 46.28 | 306 | 133 | 7 |
| 2832 | 36.09 | -4.13 | GBIF | 11.52 | 71.14 | 22.85 | 19.88 | 139 | 1 | 90.91 | 237 | 9 | 7 |
| 2833 | 34.73 | -3.07 | GBIF | 11.73 | 73.33 | 20.55 | 18.13 | 152 | 3 | 82.29 | 232 | 15 | 7 |
| 2834 | 36.13 | -4.06 | GBIF | 11.27 | 70.91 | 22.58 | 19.38 | 145 | 1 | 91.74 | 345 | 8 | 7 |
| 2835 | 36.18 | -6.68 | GBIF | 10.83 | 68.13 | 22.70 | 19.48 | 135 | 0 | 109.33 | 287 | 1 | 5 |
| 2836 | 36.18 | -4.08 | GBIF | 11.51 | 71.93 | 22.57 | 19.48 | 149 | 1 | 92.13 | 275 | 7 | 7 |
| 2837 | 36.08 | -3.74 | GBIF | 11.57 | 70.96 | 22.93 | 19.73 | 159 | 1 | 92.54 | 342 | 9 | 5 |
| 2838 | 36.05 | -4.01 | GBIF | 11.46 | 71.61 | 22.52 | 19.43 | 142 | 1 | 91.18 | 340 | 8 | 9 |
| 2839 | 36.00 | -3.77 | GBIF | 11.61 | 71.22 | 23.08 | 20.00 | 152 | 1 | 90.97 | 335 | 10 | 8 |
| 2840 | 34.95 | -1.78 | GBIF | 12.19 | 80.74 | 20.05 | 18.58 | 170 | 24 | 48.36 | 315 | 115 | 6 |
| 2841 | 35.26 | -2.49 | GBIF | 11.98 | 76.27 | 19.32 | 16.65 | 121 | 9 | 72.67 | 340 | 31 | 6 |
| 2842 | 34.73 | -2.42 | GBIF | 11.56 | 77.06 | 21.08 | 19.03 | 134 | 15 | 58.94 | 312 | 67 | 5 |
| 2843 | 37.27 | -7.23 | GBIF | 9.32 | 62.17 | 25.00 | 21.15 | 281 | 4 | 87.44 | 395 | 33 | 14 |
| 2844 | 37.27 | -8.20 | GBIF | 7.57 | 57.32 | 26.37 | 23.45 | 294 | 8 | 90.11 | 478 | 35 | 6 |
| 2845 | 36.00 | -4.11 | GBIF | 11.19 | 70.83 | 22.60 | 19.45 | 141 | 1 | 91.22 | 345 | 9 | 7 |
| 2846 | 36.15 | -4.04 | GBIF | 11.39 | 71.65 | 22.55 | 19.38 | 147 | 1 | 92.37 | 274 | 8 | 7 |
| 2847 | 35.96 | -3.76 | GBIF | 11.75 | 71.21 | 23.30 | 20.17 | 162 | 2 | 88.48 | 355 | 13 | 8 |
| 2848 | 35.53 | -3.01 | GBIF | 10.18 | 68.81 | 19.63 | 16.95 | 161 | 2 | 87.81 | 368 | 9 | 4 |
| 2849 | 34.98 | -2.56 | GBIF | 12.05 | 75.79 | 20.18 | 17.68 | 127 | 11 | 68.00 | 317 | 43 | 7 |

| 2850 | 34.73 | -1.94 | GBIF | 11.74 | 79.34 | 21.08 | 19.58 | 163 | 22 | 47.75 | 305 | 111 | 6 |
| --- | --- | --- | --- | --- | --- | --- | --- | --- | --- | --- | --- | --- | --- |
| 2851 | 36.06 | -3.60 | GBIF | 12.07 | 71.88 | 23.35 | 20.13 | 151 | 1 | 91.86 | 234 | 10 | 11 |
| 2852 | 34.97 | -2.67 | GBIF | 11.71 | 74.58 | 19.88 | 17.23 | 132 | 8 | 71.73 | 327 | 36 | 7 |
| 2853 | 36.12 | -3.67 | GBIF | 11.86 | 71.44 | 23.07 | 19.83 | 153 | 1 | 91.56 | 332 | 10 | 5 |
| 2854 | 35.83 | -3.61 | GBIF | 9.29 | 65.90 | 23.42 | 19.73 | 133 | 1 | 88.48 | 309 | 10 | 0 |
| 2855 | 34.87 | -2.45 | GBIF | 12.08 | 76.00 | 20.82 | 18.60 | 131 | 14 | 61.62 | 314 | 60 | 13 |
| 2856 | 35.44 | -3.27 | GBIF | 8.32 | 66.60 | 11.48 | 8.67 | 229 | 8 | 76.26 | 421 | 36 | 3 |
| 2857 | 35.56 | -3.34 | GBIF | 10.81 | 69.28 | 20.22 | 17.85 | 219 | 5 | 90.09 | 439 | 27 | 6 |
| 2858 | 34.84 | -2.50 | GBIF | 12.21 | 76.30 | 20.83 | 18.53 | 128 | 13 | 62.61 | 311 | 56 | 5 |
| 2859 | 35.83 | -3.88 | GBIF | 11.88 | 71.54 | 23.67 | 20.48 | 136 | 1 | 89.29 | 320 | 12 | 16 |
| 2860 | 35.05 | -2.65 | GBIF | 11.93 | 75.05 | 19.70 | 17.02 | 129 | 8 | 72.37 | 346 | 33 | 8 |
| 2861 | 34.85 | -7.74 | GBIF | 11.77 | 67.62 | 23.70 | 20.72 | 138 | 0 | 106.30 | 161 | 0 | 6 |
| 2862 | 36.17 | -4.16 | GBIF | 11.63 | 71.76 | 22.65 | 19.70 | 143 | 1 | 88.03 | 257 | 10 | 7 |
| 2863 | 34.77 | -2.21 | GBIF | 12.26 | 78.08 | 21.82 | 20.00 | 141 | 19 | 52.49 | 292 | 88 | 7 |
| 2864 | 35.61 | -3.01 | GBIF | 8.51 | 64.95 | 12.18 | 9.33 | 247 | 7 | 77.34 | 519 | 33 | 10 |
| 2865 | 34.65 | -2.34 | GBIF | 12.18 | 78.60 | 22.20 | 20.43 | 130 | 15 | 55.93 | 292 | 74 | 7 |
| 2866 | 35.87 | -3.76 | GBIF | 11.91 | 71.31 | 23.67 | 20.52 | 150 | 2 | 88.31 | 333 | 13 | 15 |
| 2867 | 34.68 | -1.97 | GBIF | 11.77 | 79.50 | 21.30 | 19.83 | 164 | 21 | 49.13 | 303 | 106 | 6 |
| 2868 | 36.03 | -3.68 | GBIF | 12.02 | 71.10 | 23.40 | 20.13 | 153 | 1 | 91.49 | 327 | 10 | 6 |
| 2869 | 35.10 | -2.63 | GBIF | 12.22 | 75.41 | 19.50 | 16.82 | 129 | 8 | 73.30 | 347 | 31 | 9 |
| 2870 | 35.50 | -3.21 | GBIF | 10.63 | 69.05 | 19.42 | 16.50 | 170 | 4 | 84.06 | 398 | 21 | 4 |
| 2871 | 35.95 | -3.75 | GBIF | 11.88 | 71.16 | 23.43 | 20.25 | 160 | 3 | 87.11 | 357 | 15 | 8 |
| 2872 | 35.58 | -3.10 | GBIF | 9.60 | 67.13 | 17.80 | 14.92 | 218 | 4 | 87.72 | 454 | 20 | 4 |
| 2873 | 36.19 | -4.00 | GBIF | 11.38 | 71.54 | 22.35 | 19.25 | 155 | 1 | 93.99 | 274 | 7 | 7 |
| 2874 | 34.97 | -7.42 | GBIF | 11.79 | 67.38 | 22.93 | 19.98 | 113 | 0 | 110.56 | 150 | 0 | 5 |
| 2875 | 36.09 | -4.07 | GBIF | 11.51 | 71.48 | 22.82 | 19.73 | 141 | 1 | 91.77 | 234 | 9 | 8 |
| 2876 | 34.97 | -2.76 | GBIF | 12.19 | 74.80 | 19.98 | 17.22 | 132 | 6 | 74.91 | 326 | 28 | 7 |
| 2877 | 35.44 | -3.40 | GBIF | 10.10 | 70.63 | 20.22 | 17.28 | 168 | 0 | 90.57 | 415 | 5 | 7 |
| 2878 | 34.55 | -2.32 | GBIF | 12.52 | 78.77 | 22.33 | 20.75 | 139 | 13 | 56.58 | 290 | 74 | 7 |
| 2879 | 35.51 | -3.20 | GBIF | 11.34 | 70.01 | 19.65 | 16.77 | 157 | 3 | 84.96 | 378 | 18 | 4 |
| 2880 | 34.97 | -2.35 | GBIF | 12.15 | 77.39 | 21.20 | 18.98 | 121 | 17 | 57.50 | 298 | 68 | 5 |
| 2881 | 34.95 | -2.44 | GBIF | 12.01 | 76.49 | 20.80 | 18.47 | 125 | 15 | 61.29 | 307 | 60 | 4 |
| 2882 | 34.83 | -2.32 | GBIF | 12.02 | 77.03 | 21.80 | 19.70 | 133 | 19 | 54.61 | 294 | 82 | 5 |
| 2883 | 34.83 | -2.24 | GBIF | 12.26 | 78.08 | 21.70 | 19.77 | 135 | 19 | 53.08 | 292 | 85 | 4 |
| 2884 | 35.53 | -3.14 | GBIF | 11.17 | 69.36 | 19.40 | 16.55 | 165 | 3 | 85.41 | 385 | 17 | 4 |
| 2885 | 35.91 | -3.32 | GBIF | 11.92 | 72.22 | 23.73 | 20.62 | 145 | 1 | 90.02 | 218 | 9 | 11 |
| 2886 | 34.97 | -2.50 | GBIF | 12.16 | 76.47 | 20.47 | 18.05 | 130 | 14 | 63.13 | 320 | 55 | 5 |
| 2887 | 34.90 | -2.35 | GBIF | 12.23 | 77.43 | 21.45 | 19.25 | 124 | 18 | 56.06 | 297 | 74 | 5 |
| 2888 | 34.97 | -2.23 | GBIF | 11.95 | 77.60 | 20.92 | 18.77 | 134 | 18 | 55.64 | 347 | 76 | 6 |
| 2889 | 34.81 | -2.21 | GBIF | 12.25 | 78.53 | 21.68 | 19.80 | 138 | 20 | 51.93 | 293 | 89 | 4 |
| 2890 | 34.94 | -2.34 | GBIF | 12.23 | 77.37 | 21.38 | 19.20 | 124 | 18 | 56.32 | 295 | 74 | 6 |
| 2891 | 35.57 | -3.04 | GBIF | 8.72 | 65.05 | 15.93 | 13.22 | 225 | 2 | 87.02 | 467 | 14 | 10 |
| 2892 | 35.68 | -3.72 | GBIF | 8.62 | 64.30 | 18.37 | 15.67 | 254 | 5 | 92.04 | 507 | 26 | 6 |
| 2893 | 34.85 | -2.30 | GBIF | 12.14 | 77.34 | 21.78 | 19.67 | 129 | 19 | 54.63 | 292 | 81 | 5 |
| 2894 | 35.72 | -3.11 | GBIF | 9.75 | 68.18 | 14.40 | 11.77 | 223 | 9 | 78.74 | 465 | 38 | 4 |
| 2895 | 34.85 | -2.40 | GBIF | 11.93 | 76.50 | 21.23 | 19.03 | 132 | 16 | 57.76 | 305 | 72 | 6 |
| 2896 | 35.10 | -2.52 | GBIF | 11.94 | 76.06 | 19.85 | 17.28 | 124 | 10 | 68.37 | 313 | 41 | 6 |
| 2897 | 35.56 | -3.19 | GBIF | 11.57 | 70.58 | 19.73 | 16.88 | 163 | 2 | 87.55 | 380 | 13 | 4 |
| 2898 | 35.48 | -3.18 | GBIF | 10.84 | 68.62 | 16.83 | 14.12 | 201 | 6 | 79.89 | 435 | 31 | 8 |
| 2899 | 35.66 | -3.53 | GBIF | 10.75 | 69.35 | 21.05 | 17.93 | 188 | 1 | 93.27 | 407 | 9 | 13 |

| 2900 | 36.10 | -4.02 | GBIF | 11.67 | 71.19 | 22.92 | 19.75 | 142 | 1 | 91.81 | 334 | 8 | 8 |
| --- | --- | --- | --- | --- | --- | --- | --- | --- | --- | --- | --- | --- | --- |
| 2901 | 34.87 | -2.36 | GBIF | 12.20 | 77.22 | 21.65 | 19.48 | 128 | 18 | 55.91 | 295 | 76 | 5 |
| 2902 | 36.16 | -3.69 | GBIF | 11.84 | 71.77 | 22.88 | 19.67 | 156 | 1 | 92.38 | 338 | 10 | 5 |
| 2903 | 36.04 | -3.99 | GBIF | 11.32 | 71.62 | 22.42 | 19.33 | 144 | 1 | 91.53 | 344 | 8 | 9 |
| 2904 | 35.67 | -3.45 | GBIF | 11.27 | 70.47 | 21.58 | 18.53 | 191 | 2 | 92.97 | 398 | 13 | 12 |
| 2905 | 36.14 | -3.79 | GBIF | 11.16 | 70.62 | 22.28 | 19.08 | 166 | 1 | 93.77 | 361 | 8 | 4 |
| 2906 | 36.09 | -3.80 | GBIF | 11.34 | 70.89 | 22.83 | 19.67 | 152 | 1 | 90.99 | 338 | 10 | 6 |
| 2907 | 35.80 | -3.67 | GBIF | 9.92 | 67.00 | 23.52 | 19.83 | 134 | 2 | 87.96 | 314 | 12 | 6 |
| 2908 | 35.96 | -3.53 | GBIF | 11.66 | 71.52 | 23.53 | 20.47 | 152 | 1 | 91.43 | 231 | 11 | 4 |
| 2909 | 36.18 | -4.04 | GBIF | 11.48 | 71.72 | 22.38 | 19.32 | 149 | 1 | 91.95 | 277 | 7 | 8 |
| 2910 | 36.19 | -3.65 | GBIF | 12.10 | 72.02 | 22.87 | 19.68 | 155 | 1 | 92.51 | 243 | 9 | 5 |
| 2911 | 35.99 | -3.64 | GBIF | 11.90 | 70.83 | 23.47 | 20.30 | 150 | 1 | 91.24 | 323 | 11 | 11 |
| 2912 | 35.97 | -3.74 | GBIF | 11.88 | 71.11 | 23.35 | 20.18 | 173 | 3 | 87.72 | 372 | 16 | 8 |
| 2913 | 35.46 | -3.31 | GBIF | 7.49 | 59.93 | 13.63 | 10.73 | 240 | 9 | 77.80 | 507 | 41 | 3 |
| 2914 | 35.60 | -3.56 | GBIF | 10.87 | 70.11 | 20.87 | 17.75 | 167 | 1 | 91.93 | 385 | 7 | 11 |
| 2915 | 36.16 | -3.81 | GBIF | 11.25 | 71.20 | 21.93 | 18.83 | 167 | 1 | 93.59 | 271 | 8 | 4 |
| 2916 | 36.00 | -3.62 | GBIF | 11.75 | 70.78 | 23.43 | 20.25 | 148 | 1 | 90.62 | 232 | 11 | 14 |
| 2917 | 36.03 | -4.01 | GBIF | 11.24 | 71.60 | 22.52 | 19.43 | 144 | 1 | 91.03 | 345 | 8 | 9 |
| 2918 | 35.97 | -3.75 | GBIF | 11.79 | 71.46 | 23.28 | 20.15 | 160 | 2 | 88.36 | 352 | 13 | 8 |
| 2919 | 36.15 | -3.88 | GBIF | 10.98 | 70.81 | 21.78 | 18.67 | 163 | 1 | 93.05 | 366 | 8 | 6 |
| 2920 | 36.04 | -3.90 | GBIF | 11.37 | 71.49 | 22.68 | 19.58 | 151 | 1 | 93.94 | 342 | 7 | 8 |
| 2921 | 35.81 | -3.55 | GBIF | 9.63 | 66.90 | 23.42 | 19.85 | 122 | 1 | 89.35 | 290 | 8 | 0 |
| 2922 | 36.10 | -3.88 | GBIF | 11.09 | 70.65 | 22.52 | 19.35 | 160 | 1 | 94.46 | 353 | 7 | 8 |
| 2923 | 36.14 | -3.89 | GBIF | 10.97 | 70.75 | 21.87 | 18.75 | 159 | 1 | 92.86 | 360 | 7 | 6 |
| 2924 | 36.12 | -3.96 | GBIF | 11.26 | 70.81 | 22.30 | 19.10 | 154 | 1 | 93.40 | 354 | 7 | 7 |
| 2925 | 35.98 | -3.44 | GBIF | 11.59 | 72.00 | 22.80 | 19.82 | 175 | 0 | 96.51 | 359 | 6 | 5 |
| 2926 | 36.08 | -4.08 | GBIF | 11.51 | 71.48 | 22.92 | 19.85 | 145 | 1 | 92.37 | 232 | 10 | 8 |
| 2927 | 36.01 | -3.89 | GBIF | 10.74 | 71.14 | 22.28 | 19.18 | 156 | 1 | 93.40 | 357 | 7 | 8 |
| 2928 | 36.09 | -3.94 | GBIF | 11.33 | 70.83 | 22.87 | 19.68 | 146 | 1 | 92.57 | 336 | 8 | 8 |
| 2929 | 34.88 | -2.56 | GBIF | 12.05 | 75.79 | 20.43 | 18.05 | 130 | 11 | 64.86 | 319 | 50 | 7 |
| 2930 | 36.01 | -4.04 | GBIF | 11.23 | 71.50 | 22.53 | 19.53 | 142 | 1 | 92.12 | 344 | 8 | 8 |
| 2931 | 36.85 | -7.99 | GBIF | 7.99 | 57.08 | 26.05 | 23.15 | 357 | 8 | 92.47 | 478 | 48 | 19 |
| 2932 | 35.97 | -3.54 | GBIF | 11.73 | 71.49 | 23.52 | 20.42 | 158 | 1 | 93.18 | 330 | 11 | 4 |
| 2933 | 34.68 | -2.20 | GBIF | 12.28 | 78.24 | 21.95 | 20.30 | 143 | 17 | 52.92 | 293 | 87 | 5 |
| 2934 | 34.66 | -2.38 | GBIF | 11.90 | 78.29 | 22.00 | 20.13 | 132 | 15 | 57.57 | 296 | 71 | 6 |
| 2935 | 34.82 | -2.88 | GBIF | 12.23 | 74.54 | 20.25 | 17.67 | 144 | 5 | 77.25 | 347 | 22 | 6 |
| 2936 | 35.97 | -3.65 | GBIF | 12.03 | 70.78 | 23.55 | 20.40 | 146 | 1 | 90.06 | 320 | 11 | 21 |
| 2937 | 34.76 | -2.25 | GBIF | 12.22 | 78.31 | 21.98 | 20.20 | 136 | 18 | 52.97 | 289 | 85 | 6 |
| 2938 | 35.54 | -3.18 | GBIF | 11.72 | 70.58 | 19.75 | 16.88 | 147 | 2 | 88.31 | 362 | 11 | 4 |
| 2939 | 34.97 | -1.41 | GBIF | 12.64 | 82.09 | 20.27 | 18.65 | 171 | 34 | 40.54 | 308 | 154 | 8 |
| 2940 | 34.65 | -7.72 | GBIF | 12.12 | 66.94 | 23.32 | 20.30 | 143 | 0 | 107.22 | 165 | 0 | 8 |
| 2941 | 34.77 | -2.14 | GBIF | 12.22 | 79.33 | 21.65 | 19.92 | 151 | 20 | 52.12 | 296 | 92 | 4 |
| 2942 | 35.55 | -3.04 | GBIF | 9.15 | 66.30 | 17.60 | 14.88 | 209 | 1 | 90.04 | 449 | 9 | 10 |
| 2943 | 34.91 | -1.55 | GBIF | 12.47 | 81.48 | 21.25 | 19.70 | 167 | 29 | 43.20 | 297 | 136 | 8 |
| 2944 | 35.66 | -3.38 | GBIF | 11.35 | 69.63 | 20.52 | 18.22 | 207 | 2 | 92.93 | 292 | 16 | 17 |
| 2945 | 34.90 | -2.48 | GBIF | 12.12 | 76.69 | 20.75 | 18.47 | 129 | 13 | 62.54 | 315 | 57 | 12 |
| 2946 | 34.85 | -2.32 | GBIF | 12.14 | 77.34 | 21.77 | 19.65 | 126 | 19 | 54.40 | 292 | 81 | 5 |
| 2947 | 36.06 | -3.91 | GBIF | 11.34 | 71.33 | 22.78 | 19.67 | 149 | 1 | 92.41 | 339 | 8 | 8 |
| 2948 | 35.44 | -3.07 | GBIF | 12.37 | 74.50 | 21.82 | 18.85 | 92 | 1 | 88.21 | 259 | 5 | 5 |
| 2949 | 35.48 | -3.35 | GBIF | 8.89 | 63.97 | 17.25 | 14.80 | 259 | 8 | 84.03 | 529 | 42 | 6 |

| 2950 | 34.90 | -2.58 | GBIF | 11.98 | 75.37 | 20.22 | 17.77 | 131 | 10 | 67.19 | 324 | 45 | 7 |
| --- | --- | --- | --- | --- | --- | --- | --- | --- | --- | --- | --- | --- | --- |
| 2951 | 36.40 | -4.00 | GBIF | 11.32 | 71.17 | 20.67 | 17.45 | 150 | 1 | 93.84 | 292 | 6 | 4 |
| 2952 | 35.43 | -3.38 | GBIF | 9.75 | 69.15 | 19.30 | 16.42 | 176 | 0 | 90.48 | 440 | 6 | 6 |
| 2953 | 34.88 | -1.91 | GBIF | 11.63 | 79.62 | 20.68 | 19.08 | 159 | 23 | 48.51 | 305 | 109 | 6 |
| 2954 | 34.89 | -2.55 | GBIF | 12.07 | 76.37 | 20.57 | 18.18 | 129 | 12 | 64.18 | 317 | 52 | 7 |
| 2955 | 31.87 | -2.25 | GBIF | 8.27 | 81.05 | 22.08 | 21.58 | 196 | 4 | 64.13 | 349 | 36 |  |
| 2956 | 35.15 | -1.91 | GBIF | 12.07 | 78.92 | 20.10 | 18.00 | 163 | 22 | 54.97 | 378 | 87 | 4 |
| 2957 | 35.92 | -3.68 | GBIF | 11.99 | 70.54 | 23.63 | 20.33 | 145 | 1 | 91.32 | 321 | 10 | 11 |
| 2958 | 36.08 | -3.69 | GBIF | 11.78 | 71.41 | 22.80 | 19.55 | 170 | 2 | 91.50 | 361 | 11 | 5 |
| 2959 | 34.97 | -2.94 | GBIF | 12.39 | 74.20 | 20.23 | 17.25 | 138 | 4 | 81.40 | 347 | 17 | 6 |
| 2960 | 35.76 | -3.00 | GBIF | 11.90 | 72.56 | 15.67 | 12.97 | 187 | 2 | 83.37 | 416 | 14 | 6 |
| 2961 | 34.38 | -6.90 | GBIF | 12.03 | 68.37 | 22.97 | 20.07 | 133 | 0 | 113.09 | 165 | 0 | 3 |
| 2962 | 35.45 | -3.04 | GBIF | 12.43 | 74.45 | 22.33 | 19.40 | 89 | 1 | 87.75 | 254 | 5 | 4 |
| 2963 | 34.93 | -2.53 | GBIF | 12.06 | 75.84 | 20.50 | 18.07 | 130 | 13 | 64.35 | 320 | 52 | 6 |
| 2964 | 35.15 | -3.12 | GBIF | 12.67 | 74.12 | 20.05 | 17.03 | 150 | 1 | 89.50 | 383 | 6 | 4 |
| 2965 | 35.94 | -3.64 | GBIF | 11.95 | 70.71 | 23.62 | 20.38 | 156 | 2 | 88.62 | 339 | 14 | 8 |
| 2966 | 34.89 | -2.35 | GBIF | 12.22 | 77.32 | 21.55 | 19.37 | 125 | 18 | 55.73 | 297 | 76 | 5 |
| 2967 | 34.90 | -2.39 | GBIF | 12.05 | 76.75 | 21.33 | 19.08 | 132 | 17 | 59.03 | 298 | 69 | 5 |
| 2968 | 34.78 | -2.25 | GBIF | 12.13 | 78.23 | 21.88 | 20.02 | 133 | 19 | 52.11 | 289 | 87 | 13 |
| 2969 | 35.60 | -3.20 | GBIF | 10.74 | 69.75 | 19.65 | 16.73 | 175 | 1 | 90.03 | 393 | 10 | 3 |
| 2970 | 35.93 | -4.13 | GBIF | 11.48 | 71.33 | 22.62 | 19.68 | 146 | 1 | 90.80 | 254 | 11 | 7 |
| 2971 | 36.16 | -4.59 | GBIF | 11.68 | 69.54 | 22.77 | 19.95 | 126 | 1 | 91.75 | 261 | 9 | 5 |
| 2972 | 35.00 | -2.30 | GBIF | 12.21 | 77.76 | 21.07 | 18.88 | 130 | 18 | 56.70 | 301 | 73 | 6 |
| 2973 | 35.51 | -3.33 | GBIF | 9.03 | 64.07 | 17.67 | 15.17 | 284 | 10 | 86.47 | 552 | 49 | 5 |
| 2974 | 35.54 | -3.16 | GBIF | 11.64 | 70.13 | 19.58 | 16.70 | 163 | 3 | 86.00 | 379 | 17 | 4 |
| 2975 | 34.98 | -2.21 | GBIF | 12.03 | 78.14 | 20.75 | 18.63 | 141 | 18 | 56.56 | 306 | 75 | 6 |
| 2976 | 35.47 | -3.18 | GBIF | 10.37 | 67.76 | 15.82 | 13.18 | 202 | 4 | 81.47 | 431 | 23 | 12 |
| 2977 | 34.94 | -2.24 | GBIF | 11.93 | 78.00 | 20.95 | 18.85 | 135 | 18 | 55.59 | 306 | 77 | 6 |
| 2978 | 34.89 | -2.39 | GBIF | 12.00 | 76.92 | 21.25 | 19.05 | 130 | 16 | 58.72 | 302 | 69 | 5 |
| 2979 | 36.08 | -3.78 | GBIF | 11.45 | 70.68 | 22.92 | 19.75 | 155 | 1 | 91.89 | 338 | 10 | 6 |
| 2980 | 36.07 | -3.64 | GBIF | 12.02 | 71.53 | 23.33 | 20.08 | 152 | 1 | 91.24 | 328 | 10 | 6 |
| 2981 | 34.95 | -2.60 | GBIF | 11.98 | 75.37 | 20.22 | 17.70 | 129 | 9 | 68.06 | 319 | 42 | 7 |
| 2982 | 34.99 | -2.38 | GBIF | 11.93 | 76.99 | 20.53 | 18.25 | 134 | 15 | 61.73 | 310 | 60 | 5 |
| 2983 | 35.55 | -3.17 | GBIF | 11.81 | 70.71 | 19.62 | 16.78 | 160 | 3 | 85.60 | 376 | 16 | 4 |
| 2984 | 34.73 | -2.11 | GBIF | 12.26 | 79.60 | 21.83 | 20.15 | 153 | 20 | 51.53 | 296 | 94 | 4 |
| 2985 | 34.71 | -1.81 | GBIF | 11.71 | 80.19 | 20.50 | 19.03 | 185 | 26 | 46.55 | 324 | 128 | 5 |
| 2986 | 36.01 | -4.07 | GBIF | 11.29 | 71.47 | 22.58 | 19.55 | 142 | 1 | 92.20 | 343 | 8 | 8 |
| 2987 | 35.66 | -3.75 | GBIF | 9.40 | 66.67 | 19.63 | 16.85 | 215 | 3 | 91.49 | 445 | 22 | 7 |
| 2988 | 34.70 | -2.78 | GBIF | 11.47 | 73.98 | 20.05 | 17.75 | 144 | 7 | 72.74 | 346 | 32 | 6 |
| 2989 | 36.16 | -4.02 | GBIF | 11.45 | 71.12 | 22.53 | 19.38 | 147 | 1 | 92.99 | 272 | 7 | 7 |
| 2990 | 34.90 | -3.16 | GBIF | 12.25 | 73.80 | 20.18 | 17.43 | 158 | 2 | 87.81 | 387 | 10 | 5 |
| 2991 | 34.98 | -3.12 | GBIF | 12.30 | 73.65 | 20.22 | 17.37 | 151 | 2 | 87.35 | 355 | 9 | 3 |
| 2992 | 35.54 | -3.08 | GBIF | 9.43 | 67.32 | 17.00 | 14.30 | 203 | 3 | 86.37 | 439 | 16 | 4 |
| 2993 | 36.14 | -3.98 | GBIF | 11.27 | 70.91 | 22.53 | 19.33 | 151 | 1 | 92.76 | 346 | 8 | 7 |
| 2994 | 34.82 | -3.09 | GBIF | 11.99 | 73.12 | 20.07 | 17.43 | 156 | 3 | 84.49 | 239 | 14 | 6 |
| 2995 | 34.96 | -2.48 | GBIF | 12.09 | 76.53 | 20.57 | 18.17 | 130 | 14 | 61.89 | 316 | 58 | 5 |
| 2996 | 34.99 | -2.41 | GBIF | 11.85 | 76.45 | 20.55 | 18.20 | 130 | 15 | 61.76 | 309 | 59 | 4 |
| 2997 | 34.67 | -2.64 | GBIF | 11.74 | 74.31 | 20.18 | 18.15 | 139 | 9 | 67.30 | 334 | 43 | 7 |
| 2998 | 36.03 | -3.96 | GBIF | 11.23 | 71.55 | 22.52 | 19.42 | 145 | 1 | 91.74 | 344 | 8 | 9 |
| 2999 | 36.81 | -3.35 | GBIF | 10.14 | 66.29 | 20.15 | 17.30 | 295 | 16 | 93.04 | 269 | 70 | 9 |

| 3000 | 36.05 | -3.99 | GBIF | 11.45 | 71.56 | 22.52 | 19.42 | 143 | 1 | 91.50 | 341 | 7 | 9 |
| --- | --- | --- | --- | --- | --- | --- | --- | --- | --- | --- | --- | --- | --- |
| 3001 | 34.62 | -2.53 | GBIF | 11.81 | 76.18 | 21.35 | 19.48 | 132 | 11 | 62.94 | 310 | 53 | 6 |
| 3002 | 34.69 | -2.72 | GBIF | 11.63 | 74.57 | 20.05 | 17.77 | 143 | 7 | 70.61 | 343 | 35 | 7 |
| 3003 | 36.08 | -3.81 | GBIF | 11.41 | 70.86 | 22.92 | 19.75 | 151 | 1 | 91.32 | 337 | 10 | 6 |
| 3004 | 36.00 | -3.60 | GBIF | 11.76 | 70.83 | 23.37 | 20.22 | 151 | 1 | 90.89 | 233 | 11 | 9 |
| 3005 | 36.18 | -3.95 | GBIF | 11.19 | 71.74 | 22.17 | 19.08 | 165 | 1 | 94.87 | 276 | 7 | 6 |
| 3006 | 35.85 | -3.55 | GBIF | 9.21 | 65.77 | 23.37 | 19.70 | 148 | 2 | 88.23 | 324 | 15 | 6 |
| 3007 | 36.07 | -3.89 | GBIF | 11.30 | 71.07 | 23.00 | 19.83 | 146 | 1 | 91.50 | 333 | 9 | 8 |
| 3008 | 36.03 | -3.64 | GBIF | 12.02 | 71.53 | 23.38 | 20.15 | 156 | 2 | 89.93 | 336 | 13 | 7 |
| 3009 | 36.09 | -3.91 | GBIF | 11.25 | 70.75 | 22.83 | 19.63 | 148 | 1 | 91.56 | 338 | 9 | 8 |
| 3010 | 36.11 | -4.17 | GBIF | 11.59 | 71.11 | 22.57 | 19.65 | 140 | 1 | 91.49 | 246 | 8 | 7 |
| 3011 | 36.18 | -3.81 | GBIF | 11.43 | 71.01 | 22.20 | 19.07 | 165 | 1 | 93.64 | 266 | 8 | 4 |
| 3012 | 36.10 | -4.03 | GBIF | 11.53 | 71.58 | 22.68 | 19.53 | 146 | 1 | 92.08 | 342 | 8 | 8 |
| 3013 | 36.03 | -3.82 | GBIF | 11.47 | 71.22 | 22.65 | 19.53 | 147 | 1 | 91.31 | 338 | 7 | 8 |
| 3014 | 36.16 | -3.96 | GBIF | 11.23 | 71.50 | 22.50 | 19.37 | 154 | 1 | 92.76 | 351 | 8 | 6 |
| 3015 | 36.02 | -3.84 | GBIF | 11.27 | 71.36 | 22.62 | 19.52 | 150 | 1 | 92.75 | 341 | 7 | 8 |
| 3016 | 36.12 | -3.75 | GBIF | 11.32 | 70.73 | 22.68 | 19.43 | 158 | 1 | 91.95 | 344 | 10 | 4 |
| 3017 | 35.88 | -3.63 | GBIF | 10.31 | 67.82 | 23.45 | 19.92 | 163 | 3 | 87.11 | 354 | 16 | 6 |
| 3018 | 35.85 | -3.60 | GBIF | 9.40 | 66.20 | 23.40 | 19.75 | 143 | 1 | 90.02 | 321 | 11 | 6 |
| 3019 | 34.76 | -2.59 | GBIF | 11.51 | 75.71 | 19.68 | 17.47 | 144 | 10 | 67.44 | 344 | 45 | 7 |
| 3020 | 35.62 | -7.80 | GBIF | 10.81 | 67.98 | 20.57 | 17.75 | 141 | 0 | 108.75 | 171 | 1 | 4 |
| 3021 | 34.83 | -2.22 | GBIF | 12.27 | 78.63 | 21.67 | 19.75 | 135 | 19 | 52.17 | 343 | 88 | 4 |
| 3022 | 34.88 | -2.38 | GBIF | 12.06 | 76.80 | 21.20 | 19.02 | 134 | 17 | 58.84 | 303 | 71 | 5 |
| 3023 | 36.07 | -4.05 | GBIF | 11.54 | 71.69 | 22.97 | 19.85 | 141 | 1 | 90.76 | 334 | 10 | 8 |
| 3024 | 36.18 | -4.06 | GBIF | 11.49 | 71.82 | 22.48 | 19.42 | 147 | 1 | 91.42 | 236 | 8 | 7 |
| 3025 | 35.45 | -3.14 | GBIF | 10.77 | 69.91 | 18.20 | 15.48 | 162 | 1 | 87.64 | 407 | 8 | 13 |
| 3026 | 34.68 | -2.40 | GBIF | 11.81 | 77.69 | 21.90 | 19.98 | 128 | 14 | 57.30 | 298 | 70 | 5 |
| 3027 | 37.13 | -7.40 | GBIF | 8.62 | 60.68 | 24.60 | 21.22 | 285 | 5 | 87.28 | 434 | 36 | 8 |
| 3028 | 37.01 | -7.23 | GBIF | 8.95 | 62.15 | 24.73 | 21.43 | 250 | 8 | 84.67 | 406 | 40 | 5 |
| 3029 | 37.02 | -7.39 | GBIF | 9.49 | 61.24 | 25.57 | 22.25 | 276 | 8 | 86.30 | 436 | 38 | 22 |
| 3030 | 34.80 | -2.51 | GBIF | 12.09 | 76.05 | 20.83 | 18.62 | 130 | 13 | 62.58 | 314 | 55 | 7 |
| 3031 | 35.49 | -3.29 | GBIF | 7.49 | 59.93 | 13.23 | 10.95 | 277 | 16 | 75.47 | 398 | 65 | 3 |
| 3032 | 34.82 | -2.22 | GBIF | 12.27 | 78.18 | 21.77 | 19.88 | 133 | 19 | 51.34 | 291 | 89 | 4 |
| 3033 | 35.85 | -3.49 | GBIF | 9.55 | 66.32 | 23.40 | 19.83 | 137 | 2 | 87.73 | 305 | 12 | 0 |
| 3034 | 34.79 | -2.10 | GBIF | 12.23 | 79.44 | 21.77 | 20.08 | 149 | 20 | 51.48 | 291 | 93 | 4 |
| 3035 | 34.61 | -2.07 | GBIF | 12.39 | 79.95 | 21.93 | 20.43 | 154 | 18 | 51.05 | 296 | 96 | 6 |
| 3036 | 35.53 | -3.03 | GBIF | 9.81 | 68.11 | 19.25 | 16.53 | 174 | 1 | 90.82 | 393 | 6 | 4 |
| 3037 | 36.68 | -8.25 | GBIF | 7.44 | 54.32 | 25.87 | 22.85 | 379 | 6 | 98.71 | 478 | 41 | 4 |
| 3038 | 35.62 | -3.66 | GBIF | 10.17 | 67.33 | 18.87 | 16.38 | 213 | 2 | 91.30 | 452 | 13 | 6 |
| 3039 | 36.10 | -3.89 | GBIF | 11.18 | 70.73 | 22.63 | 19.43 | 157 | 1 | 93.39 | 348 | 9 | 8 |
| 3040 | 34.64 | -2.11 | GBIF | 12.31 | 79.92 | 22.17 | 20.60 | 151 | 17 | 52.34 | 289 | 92 | 8 |
| 3041 | 35.91 | -3.26 | GBIF | 11.34 | 70.45 | 23.10 | 19.98 | 139 | 1 | 92.49 | 210 | 8 | 7 |
| 3042 | 34.96 | -2.37 | GBIF | 12.03 | 77.14 | 20.87 | 18.63 | 128 | 16 | 59.38 | 304 | 65 | 5 |
| 3043 | 35.41 | -3.16 | GBIF | 9.97 | 69.70 | 16.48 | 14.02 | 188 | 2 | 83.62 | 433 | 14 | 5 |
| 3044 | 35.55 | -3.15 | GBIF | 11.38 | 70.22 | 19.50 | 16.65 | 165 | 3 | 86.31 | 381 | 16 | 10 |
| 3045 | 36.18 | -4.11 | GBIF | 11.55 | 71.74 | 22.53 | 19.55 | 142 | 1 | 91.66 | 240 | 7 | 7 |
| 3046 | 36.04 | -4.02 | GBIF | 11.32 | 71.62 | 22.42 | 19.38 | 141 | 0 | 91.48 | 342 | 7 | 9 |
| 3047 | 36.44 | -3.25 | GBIF | 9.23 | 64.96 | 16.38 | 13.13 | 274 | 5 | 91.61 | 340 | 25 | 5 |
| 3048 | 35.45 | -3.23 | GBIF | 8.94 | 66.23 | 13.90 | 11.47 | 212 | 8 | 76.99 | 461 | 36 | 11 |
| 3049 | 34.62 | -2.50 | GBIF | 11.55 | 75.99 | 21.18 | 19.32 | 132 | 12 | 61.36 | 312 | 57 | 5 |

| 3050 | 35.42 | -2.82 | GBIF | 12.16 | 73.69 | 19.45 | 16.47 | 123 | 3 | 81.67 | 303 | 13 | 4 |
| --- | --- | --- | --- | --- | --- | --- | --- | --- | --- | --- | --- | --- | --- |
| 3051 | 35.74 | -3.78 | GBIF | 11.29 | 69.27 | 23.47 | 20.05 | 122 | 1 | 87.62 | 299 | 11 | 10 |
| 3052 | 36.07 | -4.00 | GBIF | 11.54 | 71.24 | 22.90 | 19.75 | 139 | 1 | 91.09 | 332 | 8 | 8 |
| 3053 | 35.62 | -3.62 | GBIF | 10.41 | 68.93 | 20.17 | 17.05 | 180 | 1 | 91.66 | 406 | 8 | 6 |
| 3054 | 36.10 | -3.86 | GBIF | 11.14 | 70.97 | 22.62 | 19.43 | 162 | 1 | 93.58 | 354 | 9 | 7 |
| 3055 | 35.92 | -3.51 | GBIF | 11.17 | 69.79 | 23.53 | 20.27 | 145 | 1 | 90.79 | 312 | 11 | 4 |
| 3056 | 35.90 | -3.42 | GBIF | 11.01 | 69.67 | 23.38 | 20.12 | 146 | 1 | 92.91 | 216 | 9 | 6 |
| 3057 | 35.91 | -3.56 | GBIF | 11.39 | 69.89 | 23.55 | 20.28 | 162 | 2 | 88.83 | 342 | 15 | 11 |
| 3058 | 35.94 | -3.57 | GBIF | 11.62 | 70.83 | 23.60 | 20.42 | 153 | 1 | 92.68 | 322 | 11 | 5 |
| 3059 | 35.95 | -3.55 | GBIF | 11.60 | 70.73 | 23.62 | 20.45 | 153 | 1 | 92.02 | 323 | 11 | 4 |
| 3060 | 35.86 | -3.27 | GBIF | 10.91 | 69.04 | 19.95 | 17.63 | 211 | 4 | 92.66 | 289 | 20 | 6 |
| 3061 | 35.83 | -3.59 | GBIF | 9.24 | 66.01 | 23.40 | 19.75 | 136 | 2 | 88.16 | 312 | 12 | 0 |
| 3062 | 35.87 | -3.64 | GBIF | 9.92 | 67.00 | 23.43 | 19.82 | 164 | 2 | 89.11 | 353 | 15 | 6 |
| 3063 | 36.15 | -3.69 | GBIF | 11.81 | 71.57 | 22.77 | 19.55 | 160 | 2 | 92.09 | 344 | 11 | 5 |
| 3064 | 35.85 | -3.76 | GBIF | 11.99 | 71.38 | 23.65 | 20.48 | 140 | 1 | 89.79 | 319 | 10 | 11 |
| 3065 | 36.98 | -3.27 | GBIF | 10.57 | 66.09 | 20.78 | 17.92 | 336 | 21 | 97.63 | 242 | 110 | 7 |
| 3066 | 36.11 | -3.80 | GBIF | 11.23 | 71.04 | 22.58 | 19.42 | 161 | 1 | 92.96 | 351 | 9 | 5 |
| 3067 | 35.99 | -3.79 | GBIF | 11.54 | 71.24 | 23.18 | 20.10 | 150 | 1 | 91.73 | 330 | 9 | 8 |
| 3068 | 38.64 | -6.31 | GBIF | 9.23 | 68.40 | 27.28 | 24.97 | 202 | 22 | 60.92 | 289 | 90 | 5 |
| 3069 | 35.96 | -3.65 | GBIF | 12.07 | 70.98 | 23.58 | 20.40 | 147 | 1 | 91.08 | 322 | 10 | 21 |
| 3070 | 37.16 | -2.67 | GBIF | 13.18 | 74.02 | 22.82 | 20.13 | 140 | 4 | 82.77 | 174 | 20 | 4 |
| 3071 | 31.33 | -6.91 | GBIF | 12.75 | 67.46 | 25.73 | 23.80 | 197 | 0 | 97.02 | 132 | 1 | 3 |
| 3072 | 38.67 | -6.39 | GBIF | 9.28 | 68.77 | 27.28 | 24.95 | 193 | 20 | 62.17 | 278 | 79 | 10 |
| 3073 | 34.62 | -2.28 | GBIF | 12.24 | 78.98 | 22.32 | 20.67 | 138 | 15 | 55.54 | 290 | 77 | 11 |
| 3074 | 36.06 | -4.06 | GBIF | 11.50 | 71.88 | 22.88 | 19.83 | 137 | 1 | 90.58 | 236 | 9 | 8 |
| 3075 | 34.94 | -2.42 | GBIF | 11.95 | 76.60 | 20.93 | 18.65 | 129 | 15 | 61.01 | 305 | 62 | 4 |
| 3076 | 35.48 | -3.38 | GBIF | 10.25 | 68.79 | 19.48 | 17.18 | 210 | 2 | 89.51 | 457 | 16 | 9 |
| 3077 | 34.77 | -2.79 | GBIF | 12.07 | 74.49 | 20.28 | 17.82 | 142 | 7 | 72.80 | 339 | 31 | 7 |
| 3078 | 36.08 | -3.75 | GBIF | 11.46 | 70.73 | 22.87 | 19.68 | 158 | 1 | 91.98 | 342 | 10 | 5 |
| 3079 | 36.69 | -3.36 | GBIF | 10.86 | 68.29 | 20.18 | 17.43 | 240 | 7 | 91.58 | 281 | 32 | 44 |
| 3080 | 35.85 | -4.00 | GBIF | 10.34 | 71.32 | 21.48 | 18.53 | 163 | 0 | 91.68 | 384 | 8 | 7 |
| 3081 | 38.62 | -6.34 | GBIF | 9.27 | 68.64 | 26.57 | 24.25 | 205 | 20 | 63.40 | 287 | 79 | 5 |
| 3082 | 35.35 | -1.97 | GBIF | 11.53 | 78.46 | 17.48 | 15.28 | 179 | 18 | 64.07 | 422 | 65 | 5 |
| 3083 | 38.60 | -6.37 | GBIF | 9.26 | 68.58 | 26.38 | 24.03 | 214 | 20 | 64.32 | 301 | 82 | 6 |
| 3084 | 38.61 | -6.38 | GBIF | 9.28 | 68.77 | 26.60 | 24.25 | 217 | 22 | 63.73 | 302 | 87 | 19 |
| 3085 | 38.75 | -6.36 | GBIF | 9.13 | 68.61 | 27.63 | 25.33 | 211 | 24 | 60.79 | 295 | 96 | 5 |
| 3086 | 35.95 | -3.26 | GBIF | 11.72 | 71.44 | 23.68 | 20.62 | 139 | 2 | 89.39 | 216 | 9 | 9 |
| 3087 | 34.97 | -3.16 | GBIF | 12.25 | 73.80 | 20.17 | 17.30 | 156 | 2 | 87.88 | 385 | 9 | 4 |
| 3088 | 34.87 | -2.44 | GBIF | 12.04 | 76.21 | 20.98 | 18.75 | 130 | 14 | 60.98 | 312 | 62 | 13 |
| 3089 | 35.95 | -3.38 | GBIF | 12.13 | 72.17 | 23.52 | 20.47 | 154 | 0 | 94.59 | 215 | 7 | 6 |
| 3090 | 38.71 | -6.22 | GBIF | 8.99 | 67.61 | 27.72 | 25.38 | 176 | 23 | 55.65 | 262 | 96 | 14 |
| 3091 | 36.03 | -3.77 | GBIF | 11.59 | 70.68 | 23.10 | 19.95 | 151 | 1 | 91.36 | 332 | 10 | 8 |
| 3092 | 34.83 | -2.30 | GBIF | 12.13 | 77.72 | 21.93 | 19.87 | 129 | 20 | 53.47 | 291 | 84 | 6 |
| 3093 | 35.27 | -3.11 | GBIF | 12.90 | 74.14 | 20.53 | 17.37 | 126 | 2 | 88.06 | 334 | 7 | 5 |
| 3094 | 35.81 | -3.73 | GBIF | 10.92 | 69.09 | 23.55 | 20.08 | 145 | 2 | 86.46 | 331 | 15 | 6 |
| 3095 | 35.00 | -3.11 | GBIF | 12.32 | 73.80 | 20.25 | 17.38 | 149 | 2 | 86.92 | 352 | 9 | 3 |
| 3096 | 36.05 | -3.70 | GBIF | 11.97 | 71.66 | 23.20 | 19.97 | 156 | 1 | 92.19 | 336 | 10 | 6 |
| 3097 | 35.37 | -1.81 | GBIF | 11.92 | 79.44 | 18.40 | 16.18 | 175 | 21 | 59.05 | 408 | 81 | 6 |
| 3098 | 35.50 | -3.14 | GBIF | 10.93 | 69.20 | 18.78 | 15.95 | 170 | 3 | 85.15 | 405 | 16 | 5 |
| 3099 | 35.95 | -3.56 | GBIF | 11.73 | 71.11 | 23.57 | 20.45 | 150 | 1 | 91.24 | 321 | 11 | 5 |

| 3100 | 34.88 | -2.31 | GBIF | 12.21 | 77.27 | 21.67 | 19.55 | 126 | 18 | 54.60 | 294 | 79 | 5 |
| --- | --- | --- | --- | --- | --- | --- | --- | --- | --- | --- | --- | --- | --- |
| 3101 | 35.81 | -3.44 | GBIF | 10.73 | 69.25 | 23.45 | 20.18 | 120 | 1 | 91.96 | 275 | 8 | 7 |
| 3102 | 35.99 | -3.41 | GBIF | 11.83 | 72.15 | 23.17 | 20.15 | 158 | 0 | 95.54 | 227 | 6 | 6 |
| 3103 | 35.89 | -3.41 | GBIF | 10.92 | 69.53 | 23.38 | 20.12 | 141 | 1 | 92.57 | 300 | 8 | 7 |
| 3104 | 35.84 | -3.57 | GBIF | 9.23 | 65.95 | 23.40 | 19.73 | 139 | 2 | 88.40 | 314 | 12 | 6 |
| 3105 | 36.04 | -4.16 | GBIF | 11.46 | 71.17 | 23.00 | 19.93 | 139 | 1 | 90.02 | 246 | 10 | 7 |
| 3106 | 34.82 | -2.02 | GBIF | 11.88 | 80.29 | 21.65 | 19.88 | 152 | 21 | 49.90 | 292 | 100 | 5 |
| 3107 | 34.90 | -2.90 | GBIF | 12.48 | 73.43 | 20.27 | 17.48 | 140 | 4 | 79.38 | 352 | 19 | 7 |
| 3108 | 35.16 | -2.00 | GBIF | 11.94 | 79.08 | 19.62 | 17.68 | 160 | 18 | 60.34 | 383 | 71 | 4 |
| 3109 | 37.70 | -3.32 | GBIF | 11.50 | 69.28 | 23.13 | 20.42 | 190 | 8 | 93.76 | 184 | 32 | 10 |
| 3110 | 35.75 | -3.57 | GBIF | 10.03 | 68.25 | 23.13 | 19.80 | 137 | 1 | 90.64 | 305 | 10 | 7 |
| 3111 | 36.04 | -4.08 | GBIF | 11.45 | 71.56 | 22.57 | 19.52 | 142 | 1 | 92.35 | 246 | 7 | 8 |
| 3112 | 38.09 | -7.67 | GBIF | 8.42 | 66.80 | 28.13 | 24.92 | 206 | 6 | 81.25 | 403 | 26 | 5 |
| 3113 | 34.44 | -2.24 | GBIF | 12.02 | 79.58 | 22.57 | 21.05 | 139 | 12 | 56.67 | 286 | 72 | 14 |
| 3114 | 37.80 | -3.93 | GBIF | 10.86 | 69.16 | 24.25 | 20.40 | 124 | 7 | 76.01 | 242 | 28 | 5 |
| 3115 | 37.77 | -3.66 | GBIF | 11.01 | 69.67 | 24.38 | 21.42 | 104 | 6 | 77.58 | 176 | 22 | 7 |
| 3116 | 35.51 | -3.24 | GBIF | 8.38 | 62.10 | 15.27 | 12.90 | 218 | 8 | 76.90 | 450 | 46 | 14 |
| 3117 | 35.80 | -3.46 | GBIF | 10.50 | 69.08 | 23.33 | 20.07 | 129 | 1 | 90.53 | 287 | 9 | 1 |
| 3118 | 34.75 | -2.62 | GBIF | 11.47 | 75.44 | 20.72 | 18.47 | 133 | 10 | 67.19 | 322 | 44 | 11 |
| 3119 | 35.00 | -3.02 | GBIF | 12.18 | 73.79 | 20.42 | 17.40 | 141 | 3 | 84.48 | 336 | 13 | 5 |
| 3120 | 35.02 | -2.49 | GBIF | 12.13 | 75.83 | 20.40 | 17.93 | 125 | 13 | 65.15 | 313 | 49 | 5 |
| 3121 | 35.66 | -3.32 | GBIF | 10.87 | 67.92 | 20.03 | 17.60 | 245 | 4 | 92.84 | 477 | 25 | 7 |
| 3122 | 35.96 | -3.95 | GBIF | 10.87 | 71.96 | 22.72 | 19.70 | 142 | 1 | 90.21 | 339 | 9 | 6 |
| 3123 | 36.90 | -3.23 | GBIF | 10.26 | 66.18 | 19.67 | 16.83 | 282 | 15 | 92.16 | 237 | 84 | 8 |
| 3124 | 37.14 | -7.35 | GBIF | 9.46 | 60.63 | 25.40 | 21.28 | 285 | 7 | 86.60 | 430 | 37 | 15 |
| 3125 | 37.82 | -4.07 | GBIF | 10.88 | 68.40 | 24.70 | 20.93 | 118 | 10 | 66.52 | 241 | 47 | 20 |
| 3126 | 35.98 | -3.78 | GBIF | 11.59 | 71.11 | 23.22 | 20.12 | 151 | 1 | 91.04 | 331 | 10 | 8 |
| 3127 | 34.82 | -2.44 | GBIF | 12.13 | 76.26 | 21.15 | 18.97 | 133 | 15 | 60.26 | 307 | 65 | 9 |
| 3128 | 36.22 | -4.04 | GBIF | 11.28 | 71.87 | 22.23 | 19.17 | 152 | 1 | 91.46 | 280 | 9 | 7 |
| 3129 | 35.59 | -3.17 | GBIF | 11.54 | 71.24 | 19.65 | 16.88 | 169 | 2 | 88.68 | 386 | 12 | 4 |
| 3130 | 34.80 | -2.43 | GBIF | 12.08 | 76.96 | 21.13 | 19.00 | 132 | 15 | 59.15 | 308 | 67 | 6 |
| 3131 | 36.85 | -3.26 | GBIF | 9.37 | 63.72 | 18.43 | 15.42 | 307 | 16 | 93.34 | 266 | 77 | 8 |
| 3132 | 37.11 | -2.94 | GBIF | 10.57 | 65.63 | 16.42 | 13.50 | 276 | 6 | 91.82 | 305 | 26 | 7 |
| 3133 | 35.05 | -2.93 | GBIF | 12.64 | 74.36 | 20.25 | 17.22 | 133 | 4 | 81.97 | 326 | 17 | 8 |
| 3134 | 35.47 | -2.03 | GBIF | 12.10 | 79.61 | 18.02 | 15.82 | 166 | 13 | 71.15 | 400 | 44 | 3 |
| 3135 | 34.80 | -2.26 | GBIF | 12.07 | 78.35 | 21.78 | 19.82 | 134 | 19 | 53.04 | 293 | 86 | 13 |
| 3136 | 36.07 | -3.84 | GBIF | 11.39 | 71.20 | 22.95 | 19.78 | 150 | 1 | 91.90 | 335 | 9 | 8 |
| 3137 | 35.57 | -3.26 | GBIF | 8.52 | 62.62 | 16.53 | 14.10 | 239 | 7 | 82.16 | 482 | 42 | 18 |
| 3138 | 34.21 | -2.18 | GBIF | 11.86 | 80.12 | 22.88 | 21.95 | 143 | 9 | 58.64 | 283 | 66 | 14 |
| 3139 | 35.55 | -3.38 | GBIF | 11.27 | 69.98 | 20.68 | 18.32 | 195 | 4 | 87.93 | 409 | 24 | 16 |
| 3140 | 36.95 | -3.39 | GBIF | 11.61 | 66.71 | 22.22 | 19.43 | 254 | 12 | 99.84 | 201 | 53 | 16 |
| 3141 | 36.04 | -3.96 | GBIF | 11.28 | 71.41 | 22.40 | 19.32 | 144 | 1 | 91.47 | 343 | 7 | 9 |
| 3142 | 35.64 | -3.58 | GBIF | 10.57 | 69.57 | 20.70 | 17.60 | 182 | 1 | 92.70 | 400 | 8 | 6 |
| 3143 | 35.66 | -3.44 | GBIF | 11.38 | 70.70 | 21.77 | 18.70 | 189 | 2 | 92.87 | 393 | 13 | 13 |
| 3144 | 35.47 | -3.15 | GBIF | 10.44 | 68.70 | 16.58 | 13.98 | 191 | 3 | 84.17 | 425 | 17 | 12 |
| 3145 | 35.71 | -3.43 | GBIF | 10.93 | 69.59 | 20.80 | 18.42 | 207 | 1 | 95.69 | 416 | 13 | 16 |
| 3146 | 35.02 | -2.12 | GBIF | 12.22 | 78.82 | 20.70 | 18.72 | 147 | 19 | 56.80 | 361 | 77 | 5 |
| 3147 | 35.92 | -3.63 | GBIF | 11.82 | 70.34 | 23.60 | 20.32 | 151 | 1 | 90.26 | 327 | 11 | 7 |
| 3148 | 34.65 | -2.15 | GBIF | 12.27 | 78.69 | 21.98 | 20.38 | 150 | 17 | 52.31 | 293 | 90 | 4 |
| 3149 | 34.98 | -2.52 | GBIF | 12.15 | 75.94 | 20.33 | 17.88 | 127 | 11 | 66.26 | 316 | 46 | 6 |

| 3150 | 34.83 | -3.19 | GBIF | 11.68 | 72.57 | 20.30 | 17.53 | 156 | 2 | 86.79 | 388 | 10 | 6 |
| --- | --- | --- | --- | --- | --- | --- | --- | --- | --- | --- | --- | --- | --- |
| 3151 | 34.77 | -2.48 | GBIF | 11.93 | 76.44 | 20.83 | 18.73 | 133 | 13 | 61.61 | 316 | 59 | 6 |
| 3152 | 38.11 | -7.63 | GBIF | 8.77 | 66.92 | 28.25 | 25.02 | 227 | 7 | 79.15 | 406 | 34 | 5 |
| 3153 | 35.22 | -1.87 | GBIF | 12.23 | 79.96 | 19.83 | 17.78 | 158 | 21 | 55.77 | 376 | 84 | 13 |
| 3154 | 38.19 | -7.71 | GBIF | 8.09 | 65.79 | 28.00 | 24.80 | 199 | 5 | 80.76 | 381 | 26 | 6 |
| 3155 | 36.16 | -3.94 | GBIF | 11.16 | 71.07 | 22.43 | 19.28 | 158 | 1 | 93.92 | 272 | 7 | 7 |
| 3156 | 35.62 | -3.56 | GBIF | 10.83 | 70.35 | 20.83 | 17.77 | 173 | 1 | 92.30 | 389 | 8 | 8 |
| 3157 | 35.85 | -3.74 | GBIF | 11.48 | 70.40 | 23.63 | 20.30 | 153 | 2 | 88.40 | 340 | 14 | 7 |
| 3158 | 34.82 | -7.68 | GBIF | 12.02 | 67.18 | 23.45 | 20.42 | 133 | 0 | 108.20 | 156 | 0 | 6 |
| 3159 | 34.59 | -2.21 | GBIF | 12.35 | 78.66 | 22.28 | 20.73 | 142 | 15 | 53.99 | 289 | 82 | 6 |
| 3160 | 35.57 | -3.16 | GBIF | 11.43 | 70.96 | 19.55 | 16.73 | 165 | 2 | 87.19 | 380 | 13 | 4 |
| 3161 | 35.13 | -2.19 | GBIF | 12.20 | 79.22 | 20.45 | 18.18 | 138 | 16 | 61.39 | 352 | 61 | 4 |
| 3162 | 34.82 | -2.52 | GBIF | 12.13 | 75.31 | 20.78 | 18.48 | 129 | 12 | 63.00 | 313 | 54 | 14 |
| 3163 | 34.72 | -2.25 | GBIF | 12.19 | 78.15 | 22.03 | 20.28 | 137 | 19 | 52.77 | 288 | 86 | 6 |
| 3164 | 35.99 | -3.68 | GBIF | 12.02 | 70.69 | 23.50 | 20.32 | 151 | 1 | 91.51 | 327 | 10 | 7 |
| 3165 | 36.90 | -3.36 | GBIF | 11.06 | 66.62 | 20.93 | 18.13 | 293 | 19 | 93.68 | 246 | 82 | 9 |
| 3166 | 37.16 | -7.36 | GBIF | 8.90 | 60.54 | 25.07 | 21.47 | 281 | 5 | 87.11 | 432 | 34 | 11 |
| 3167 | 36.20 | -3.68 | GBIF | 11.87 | 71.92 | 22.63 | 19.47 | 157 | 1 | 92.22 | 340 | 9 | 4 |
| 3168 | 35.63 | -7.56 | GBIF | 10.32 | 68.38 | 21.67 | 19.12 | 129 | 0 | 109.33 | 150 | 1 | 5 |
| 3169 | 35.99 | -3.66 | GBIF | 12.00 | 70.59 | 23.52 | 20.30 | 148 | 1 | 90.91 | 322 | 10 | 8 |
| 3170 | 35.50 | -3.06 | GBIF | 10.76 | 70.78 | 20.43 | 17.40 | 112 | 1 | 89.58 | 300 | 5 | 4 |
| 3171 | 35.94 | -3.27 | GBIF | 11.77 | 71.80 | 23.70 | 20.67 | 140 | 1 | 92.38 | 208 | 8 | 8 |
| 3172 | 34.90 | -2.28 | GBIF | 12.20 | 77.71 | 21.68 | 19.57 | 130 | 19 | 54.71 | 293 | 80 | 5 |
| 3173 | 36.09 | -3.67 | GBIF | 11.78 | 71.85 | 22.73 | 19.52 | 158 | 1 | 90.96 | 346 | 10 | 5 |
| 3174 | 35.49 | -3.05 | GBIF | 11.19 | 71.74 | 21.40 | 18.37 | 102 | 1 | 88.24 | 237 | 5 | 4 |
| 3175 | 36.60 | -5.76 | GBIF | 12.30 | 69.49 | 22.02 | 18.57 | 115 | 1 | 95.46 | 271 | 7 | 5 |
| 3176 | 34.86 | -7.64 | GBIF | 11.89 | 67.57 | 23.72 | 20.72 | 130 | 0 | 107.75 | 153 | 0 | 13 |
| 3177 | 35.80 | -3.60 | GBIF | 9.52 | 66.09 | 23.45 | 19.80 | 131 | 1 | 89.38 | 305 | 10 | 0 |
| 3178 | 36.00 | -3.66 | GBIF | 11.96 | 70.76 | 23.45 | 20.27 | 151 | 1 | 91.37 | 323 | 10 | 7 |
| 3179 | 35.89 | -3.76 | GBIF | 11.77 | 71.31 | 23.57 | 20.43 | 163 | 2 | 87.00 | 356 | 15 | 11 |
| 3180 | 36.19 | -3.92 | GBIF | 11.16 | 71.53 | 21.92 | 18.82 | 157 | 1 | 92.79 | 277 | 8 | 6 |
| 3181 | 37.26 | -6.85 | GBIF | 10.18 | 65.70 | 26.48 | 22.93 | 190 | 7 | 80.92 | 370 | 32 | 7 |
| 3182 | 36.11 | -3.69 | GBIF | 11.78 | 71.41 | 22.83 | 19.58 | 162 | 1 | 92.75 | 345 | 10 | 5 |
| 3183 | 37.47 | -7.10 | GBIF | 9.47 | 65.34 | 24.30 | 20.60 | 255 | 5 | 84.50 | 394 | 22 | 12 |
| 3184 | 36.09 | -3.74 | GBIF | 11.44 | 70.63 | 22.83 | 19.58 | 161 | 1 | 92.85 | 344 | 9 | 5 |
| 3185 | 37.53 | -7.10 | GBIF | 9.37 | 67.39 | 21.17 | 17.80 | 296 | 9 | 80.84 | 496 | 34 | 7 |
| 3186 | 36.06 | -3.44 | GBIF | 9.85 | 68.40 | 20.72 | 17.53 | 212 | 1 | 97.78 | 432 | 9 | 5 |
| 3187 | 34.62 | -2.42 | GBIF | 11.53 | 76.83 | 21.43 | 19.58 | 135 | 13 | 58.89 | 307 | 66 | 4 |
| 3188 | 34.85 | -6.59 | GBIF | 11.56 | 67.99 | 23.35 | 20.10 | 126 | 0 | 113.76 | 154 | 0 | 3 |
| 3189 | 38.03 | -4.11 | GBIF | 10.40 | 67.97 | 24.22 | 20.82 | 126 | 4 | 79.96 | 245 | 21 | 14 |
| 3190 | 34.90 | -2.83 | GBIF | 12.37 | 74.05 | 20.25 | 17.53 | 136 | 6 | 76.46 | 333 | 24 | 7 |
| 3191 | 36.20 | -3.53 | GBIF | 11.98 | 70.91 | 22.62 | 19.40 | 156 | 1 | 93.38 | 246 | 8 | 6 |
| 3192 | 36.51 | -7.61 | GBIF | 9.24 | 63.74 | 22.92 | 20.57 | 239 | 2 | 94.19 | 410 | 21 | 4 |
| 3193 | 34.76 | -2.47 | GBIF | 11.79 | 76.57 | 21.10 | 18.97 | 129 | 14 | 60.04 | 312 | 62 | 6 |
| 3194 | 34.78 | -2.54 | GBIF | 12.07 | 75.89 | 20.73 | 18.52 | 132 | 12 | 63.76 | 319 | 53 | 8 |
| 3195 | 34.97 | -2.22 | GBIF | 11.93 | 78.00 | 20.73 | 18.58 | 138 | 18 | 56.84 | 306 | 74 | 6 |
| 3196 | 34.87 | -2.29 | GBIF | 12.21 | 77.76 | 21.77 | 19.67 | 125 | 20 | 53.59 | 294 | 83 | 5 |
| 3197 | 34.85 | -2.29 | GBIF | 12.18 | 78.10 | 21.83 | 19.75 | 126 | 20 | 53.21 | 291 | 84 | 5 |
| 3198 | 34.78 | -2.41 | GBIF | 12.02 | 76.54 | 21.08 | 18.93 | 138 | 16 | 59.60 | 310 | 68 | 5 |
| 3199 | 34.74 | -2.52 | GBIF | 12.01 | 76.98 | 20.95 | 18.85 | 130 | 12 | 62.45 | 315 | 54 | 7 |

| 3200 | 35.78 | -3.34 | GBIF | 11.09 | 69.32 | 21.15 | 18.72 | 198 | 2 | 93.24 | 264 | 17 | 15 |
| --- | --- | --- | --- | --- | --- | --- | --- | --- | --- | --- | --- | --- | --- |
| 3201 | 34.74 | -2.54 | GBIF | 12.07 | 76.42 | 20.90 | 18.75 | 132 | 12 | 63.56 | 317 | 52 | 7 |
| 3202 | 34.80 | -2.48 | GBIF | 12.06 | 76.32 | 20.95 | 18.77 | 131 | 13 | 61.66 | 312 | 59 | 6 |
| 3203 | 34.77 | -2.58 | GBIF | 11.76 | 75.86 | 20.48 | 18.23 | 137 | 11 | 65.68 | 326 | 48 | 9 |
| 3204 | 34.77 | -2.40 | GBIF | 11.79 | 77.07 | 20.82 | 18.72 | 139 | 15 | 59.64 | 316 | 67 | 5 |
| 3205 | 35.76 | -3.63 | GBIF | 9.68 | 66.72 | 23.27 | 19.70 | 112 | 1 | 91.46 | 281 | 7 | 6 |
| 3206 | 34.69 | -2.60 | GBIF | 11.79 | 76.08 | 20.63 | 18.55 | 136 | 10 | 65.88 | 326 | 47 | 11 |
| 3207 | 34.60 | -2.59 | GBIF | 11.21 | 75.22 | 19.50 | 17.60 | 155 | 10 | 66.07 | 353 | 48 | 6 |
| 3208 | 34.85 | -2.23 | GBIF | 12.20 | 78.21 | 21.45 | 19.47 | 137 | 19 | 53.34 | 299 | 85 | 4 |
| 3209 | 34.61 | -2.46 | GBIF | 11.47 | 75.94 | 21.17 | 19.28 | 141 | 13 | 60.25 | 312 | 63 | 4 |
| 3210 | 34.74 | -2.44 | GBIF | 11.61 | 76.88 | 21.38 | 19.28 | 129 | 15 | 58.96 | 304 | 66 | 5 |
| 3211 | 34.74 | -2.58 | GBIF | 11.62 | 76.43 | 20.23 | 18.08 | 137 | 10 | 65.81 | 333 | 46 | 7 |
| 3212 | 34.87 | -2.33 | GBIF | 12.17 | 77.49 | 21.60 | 19.50 | 130 | 18 | 55.83 | 297 | 78 | 5 |
| 3213 | 34.83 | -2.54 | GBIF | 12.09 | 75.10 | 20.68 | 18.33 | 130 | 12 | 63.51 | 316 | 54 | 10 |
| 3214 | 34.69 | -2.58 | GBIF | 11.89 | 76.23 | 20.98 | 18.90 | 133 | 10 | 65.27 | 317 | 49 | 11 |
| 3215 | 34.66 | -2.46 | GBIF | 11.28 | 76.24 | 20.32 | 18.35 | 141 | 13 | 60.41 | 332 | 62 | 4 |
| 3216 | 34.91 | -2.50 | GBIF | 12.13 | 76.74 | 20.55 | 18.22 | 134 | 14 | 62.14 | 326 | 59 | 7 |
| 3217 | 34.74 | -2.55 | GBIF | 11.93 | 76.50 | 20.92 | 18.72 | 141 | 12 | 62.42 | 325 | 58 | 8 |
| 3218 | 36.04 | -3.93 | GBIF | 11.20 | 71.34 | 22.33 | 19.23 | 146 | 1 | 92.29 | 344 | 7 | 9 |
| 3219 | 34.81 | -7.61 | GBIF | 11.83 | 67.23 | 23.72 | 20.65 | 131 | 0 | 108.16 | 154 | 0 | 6 |
| 3220 | 34.85 | -7.47 | GBIF | 11.98 | 66.90 | 23.02 | 20.03 | 121 | 0 | 109.53 | 155 | 0 | 4 |
| 3221 | 34.75 | -2.58 | GBIF | 11.56 | 76.04 | 20.08 | 17.90 | 140 | 10 | 65.85 | 337 | 46 | 7 |
| 3222 | 34.80 | -7.78 | GBIF | 11.88 | 67.09 | 23.47 | 20.50 | 142 | 0 | 106.22 | 163 | 0 | 5 |
| 3223 | 36.07 | -4.18 | GBIF | 11.58 | 71.06 | 22.97 | 19.95 | 141 | 1 | 91.09 | 242 | 10 | 7 |
| 3224 | 34.89 | -2.52 | GBIF | 12.12 | 76.69 | 20.62 | 18.30 | 129 | 13 | 62.84 | 318 | 55 | 6 |
| 3225 | 35.00 | -2.36 | GBIF | 12.13 | 77.23 | 21.05 | 18.75 | 125 | 16 | 59.07 | 300 | 64 | 5 |
| 3226 | 36.07 | -4.10 | GBIF | 11.52 | 71.14 | 22.98 | 19.95 | 137 | 1 | 90.54 | 234 | 10 | 7 |
| 3227 | 34.99 | -2.21 | GBIF | 12.00 | 77.92 | 20.88 | 18.73 | 136 | 18 | 56.99 | 302 | 73 | 6 |
| 3228 | 35.86 | -3.61 | GBIF | 9.55 | 66.78 | 23.35 | 19.75 | 150 | 2 | 88.00 | 334 | 14 | 6 |
| 3229 | 35.83 | -3.66 | GBIF | 9.72 | 66.55 | 23.52 | 19.78 | 138 | 2 | 86.61 | 317 | 14 | 0 |
| 3230 | 36.08 | -4.13 | GBIF | 11.54 | 70.81 | 22.95 | 19.90 | 140 | 1 | 90.81 | 236 | 9 | 7 |
| 3231 | 34.96 | -2.51 | GBIF | 12.12 | 76.21 | 20.35 | 17.92 | 127 | 12 | 64.72 | 317 | 49 | 6 |
| 3232 | 35.43 | -2.86 | GBIF | 13.36 | 75.05 | 21.43 | 18.30 | 84 | 4 | 79.69 | 235 | 14 | 4 |
| 3233 | 35.90 | -3.80 | GBIF | 11.41 | 70.42 | 23.42 | 20.20 | 152 | 2 | 87.42 | 342 | 15 | 8 |
| 3234 | 38.08 | -7.61 | GBIF | 8.99 | 67.10 | 28.30 | 25.07 | 232 | 8 | 78.97 | 410 | 35 | 5 |
| 3235 | 35.41 | -3.32 | GBIF | 10.02 | 69.62 | 18.82 | 15.72 | 184 | 0 | 91.83 | 454 | 4 | 4 |
| 3236 | 34.65 | -7.77 | GBIF | 12.06 | 67.36 | 23.25 | 20.32 | 147 | 0 | 106.46 | 167 | 0 | 7 |
| 3237 | 36.19 | -3.63 | GBIF | 12.10 | 72.02 | 22.83 | 19.68 | 155 | 1 | 92.87 | 245 | 9 | 6 |
| 3238 | 35.76 | -3.80 | GBIF | 11.72 | 70.21 | 23.50 | 20.17 | 135 | 2 | 87.22 | 318 | 13 | 7 |
| 3239 | 36.15 | -3.95 | GBIF | 11.15 | 71.02 | 22.45 | 19.27 | 157 | 1 | 94.47 | 354 | 7 | 6 |
| 3240 | 38.12 | -7.80 | GBIF | 8.31 | 65.42 | 27.72 | 24.52 | 205 | 6 | 79.79 | 404 | 30 | 6 |
| 3241 | 36.19 | -4.06 | GBIF | 11.38 | 71.59 | 22.43 | 19.33 | 149 | 1 | 91.88 | 277 | 8 | 8 |
| 3242 | 35.97 | -3.27 | GBIF | 11.94 | 71.94 | 23.50 | 20.47 | 137 | 1 | 92.83 | 208 | 7 | 11 |
| 3243 | 35.89 | -3.54 | GBIF | 10.18 | 67.83 | 23.45 | 19.98 | 168 | 3 | 86.58 | 357 | 19 | 5 |
| 3244 | 37.06 | -7.28 | GBIF | 9.45 | 61.76 | 25.57 | 22.08 | 271 | 10 | 84.16 | 410 | 45 | 6 |
| 3245 | 35.98 | -3.39 | GBIF | 12.05 | 72.16 | 23.35 | 20.33 | 158 | 0 | 95.46 | 223 | 6 | 6 |
| 3246 | 35.94 | -3.58 | GBIF | 11.68 | 70.81 | 23.57 | 20.38 | 156 | 1 | 92.10 | 327 | 11 | 11 |
| 3247 | 35.98 | -3.53 | GBIF | 11.68 | 71.24 | 23.45 | 20.40 | 151 | 1 | 92.50 | 231 | 9 | 4 |
| 3248 | 35.99 | -3.25 | GBIF | 12.24 | 72.87 | 23.95 | 20.92 | 128 | 1 | 92.38 | 197 | 6 | 8 |
| 3249 | 35.87 | -3.41 | GBIF | 10.36 | 68.60 | 23.32 | 19.97 | 137 | 1 | 91.63 | 295 | 9 | 6 |

| 3250 | 35.67 | -3.80 | GBIF | 10.28 | 68.56 | 20.53 | 17.27 | 198 | 2 | 91.35 | 422 | 17 | 15 |
| --- | --- | --- | --- | --- | --- | --- | --- | --- | --- | --- | --- | --- | --- |
| 3251 | 35.87 | -3.71 | GBIF | 10.91 | 68.61 | 23.48 | 20.05 | 149 | 2 | 88.81 | 328 | 13 | 7 |
| 3252 | 35.84 | -3.73 | GBIF | 11.37 | 70.16 | 23.57 | 20.25 | 165 | 3 | 86.55 | 362 | 18 | 6 |
| 3253 | 35.41 | -3.02 | GBIF | 13.42 | 76.23 | 22.23 | 19.13 | 88 | 2 | 85.62 | 249 | 8 | 8 |
| 3254 | 35.83 | -3.70 | GBIF | 10.00 | 66.67 | 23.52 | 19.83 | 150 | 2 | 88.39 | 333 | 14 | 6 |
| 3255 | 35.40 | -3.01 | GBIF | 13.35 | 75.85 | 22.13 | 19.00 | 87 | 3 | 84.53 | 247 | 9 | 6 |
| 3256 | 35.81 | -3.71 | GBIF | 10.33 | 67.93 | 23.55 | 19.92 | 141 | 2 | 86.92 | 323 | 14 | 6 |
| 3257 | 35.42 | -3.39 | GBIF | 10.09 | 71.57 | 20.50 | 17.22 | 147 | 0 | 92.67 | 385 | 2 | 6 |
| 3258 | 35.83 | -3.76 | GBIF | 11.88 | 71.16 | 23.60 | 20.38 | 154 | 2 | 87.44 | 347 | 14 | 6 |
| 3259 | 36.11 | -3.78 | GBIF | 11.23 | 71.10 | 22.58 | 19.42 | 163 | 1 | 93.24 | 352 | 10 | 4 |
| 3260 | 35.55 | -3.27 | GBIF | 8.39 | 62.16 | 15.65 | 13.25 | 287 | 14 | 80.01 | 540 | 67 | 18 |
| 3261 | 35.83 | -3.40 | GBIF | 10.87 | 69.66 | 23.35 | 20.12 | 141 | 1 | 93.14 | 295 | 9 | 8 |
| 3262 | 35.95 | -3.27 | GBIF | 11.85 | 71.82 | 23.68 | 20.62 | 137 | 1 | 92.80 | 208 | 7 | 11 |
| 3263 | 34.98 | -2.26 | GBIF | 12.02 | 77.58 | 21.32 | 19.15 | 127 | 19 | 55.44 | 295 | 77 | 7 |
| 3264 | 35.45 | -3.11 | GBIF | 11.63 | 72.20 | 20.42 | 17.33 | 113 | 1 | 90.10 | 316 | 4 | 9 |
| 3265 | 35.46 | -3.18 | GBIF | 10.48 | 68.46 | 16.65 | 13.95 | 189 | 2 | 84.40 | 427 | 15 | 8 |
| 3266 | 34.74 | -2.79 | GBIF | 11.87 | 74.63 | 20.28 | 17.88 | 142 | 7 | 72.89 | 340 | 31 | 7 |
| 3267 | 36.13 | -3.62 | GBIF | 12.07 | 71.40 | 23.13 | 19.95 | 150 | 1 | 91.92 | 236 | 9 | 5 |
| 3268 | 35.51 | -3.01 | GBIF | 11.05 | 71.29 | 21.28 | 18.25 | 115 | 2 | 85.94 | 285 | 8 | 4 |
| 3269 | 36.03 | -3.67 | GBIF | 12.04 | 71.25 | 23.38 | 20.13 | 153 | 1 | 91.99 | 328 | 9 | 6 |
| 3270 | 35.83 | -3.84 | GBIF | 12.30 | 72.35 | 23.82 | 20.62 | 135 | 1 | 91.19 | 317 | 9 | 11 |
| 3271 | 35.02 | -2.05 | GBIF | 12.11 | 78.63 | 20.73 | 18.87 | 150 | 19 | 55.60 | 365 | 81 | 5 |
| 3272 | 36.03 | -3.91 | GBIF | 11.11 | 71.21 | 22.30 | 19.22 | 153 | 1 | 92.95 | 351 | 7 | 8 |
| 3273 | 35.75 | -3.46 | GBIF | 10.51 | 69.59 | 21.83 | 18.75 | 177 | 1 | 93.93 | 372 | 9 | 8 |
| 3274 | 35.76 | -3.65 | GBIF | 9.82 | 67.24 | 23.33 | 19.72 | 111 | 1 | 91.92 | 280 | 6 | 6 |
| 3275 | 35.67 | -3.96 | GBIF | 10.35 | 69.46 | 22.97 | 19.75 | 150 | 2 | 86.36 | 352 | 21 | 9 |
| 3276 | 36.19 | -4.22 | GBIF | 11.61 | 72.10 | 22.75 | 19.83 | 133 | 1 | 91.62 | 239 | 7 | 8 |
| 3277 | 35.72 | -3.62 | GBIF | 8.74 | 65.24 | 18.68 | 16.08 | 231 | 3 | 92.87 | 473 | 16 | 6 |
| 3278 | 35.99 | -4.10 | GBIF | 11.22 | 70.55 | 22.72 | 19.60 | 140 | 1 | 91.09 | 344 | 10 | 7 |
| 3279 | 35.85 | -3.96 | GBIF | 11.08 | 71.05 | 23.52 | 20.43 | 140 | 1 | 88.84 | 331 | 13 | 7 |
| 3280 | 36.05 | -3.94 | GBIF | 11.32 | 70.78 | 22.63 | 19.50 | 141 | 1 | 91.57 | 335 | 7 | 9 |
| 3281 | 34.13 | -2.18 | GBIF | 11.86 | 79.06 | 22.87 | 22.03 | 149 | 8 | 60.50 | 180 | 64 | 10 |
| 3282 | 34.89 | -2.59 | GBIF | 11.95 | 75.63 | 20.28 | 17.87 | 131 | 10 | 66.93 | 322 | 46 | 7 |
| 3283 | 37.92 | -4.05 | GBIF | 10.52 | 67.90 | 25.05 | 21.33 | 121 | 8 | 72.17 | 241 | 38 | 5 |
| 3284 | 34.81 | -2.26 | GBIF | 12.12 | 77.67 | 21.77 | 19.80 | 134 | 19 | 53.24 | 294 | 85 | 12 |
| 3285 | 35.92 | -3.61 | GBIF | 11.71 | 70.53 | 23.58 | 20.35 | 150 | 1 | 91.17 | 322 | 11 | 6 |
| 3286 | 36.02 | -3.61 | GBIF | 11.86 | 71.44 | 23.30 | 20.15 | 152 | 1 | 91.41 | 233 | 10 | 11 |
| 3287 | 35.96 | -3.33 | GBIF | 12.08 | 71.92 | 23.32 | 20.27 | 152 | 1 | 94.72 | 218 | 8 | 7 |
| 3288 | 37.84 | -4.07 | GBIF | 10.95 | 68.44 | 23.95 | 21.13 | 121 | 10 | 66.90 | 243 | 46 | 10 |
| 3289 | 34.85 | -2.38 | GBIF | 12.06 | 76.80 | 21.30 | 19.12 | 132 | 17 | 57.52 | 304 | 73 | 5 |
| 3290 | 35.43 | -3.01 | GBIF | 13.17 | 75.67 | 22.43 | 19.40 | 88 | 2 | 86.26 | 252 | 6 | 9 |
| 3291 | 35.90 | -3.89 | GBIF | 10.09 | 67.73 | 23.33 | 19.90 | 114 | 1 | 91.05 | 290 | 8 | 7 |
| 3292 | 36.18 | -4.19 | GBIF | 11.73 | 71.93 | 22.68 | 19.80 | 136 | 1 | 88.79 | 246 | 9 | 8 |
| 3293 | 36.06 | -4.07 | GBIF | 11.52 | 71.53 | 22.93 | 19.85 | 140 | 1 | 91.64 | 235 | 8 | 8 |
| 3294 | 36.13 | -3.72 | GBIF | 11.67 | 71.14 | 22.87 | 19.58 | 162 | 1 | 92.39 | 345 | 10 | 5 |
| 3295 | 34.93 | -2.49 | GBIF | 12.15 | 76.90 | 20.63 | 18.32 | 130 | 14 | 61.21 | 320 | 60 | 5 |
| 3296 | 34.94 | -2.21 | GBIF | 11.96 | 78.16 | 20.82 | 18.73 | 140 | 18 | 56.04 | 357 | 77 | 6 |
| 3297 | 35.51 | -3.07 | GBIF | 10.11 | 69.24 | 19.23 | 16.13 | 154 | 1 | 90.80 | 378 | 5 | 4 |
| 3298 | 35.48 | -3.09 | GBIF | 10.83 | 70.35 | 19.63 | 16.57 | 137 | 1 | 91.04 | 360 | 4 | 4 |
| 3299 | 36.02 | -4.15 | GBIF | 11.23 | 71.10 | 22.57 | 19.53 | 141 | 0 | 90.63 | 252 | 9 | 7 |

| 3300 | 35.87 | -3.52 | GBIF | 9.32 | 66.08 | 23.35 | 19.75 | 152 | 2 | 88.42 | 328 | 14 | 0 |
| --- | --- | --- | --- | --- | --- | --- | --- | --- | --- | --- | --- | --- | --- |
| 3301 | 35.01 | -2.28 | GBIF | 12.10 | 77.56 | 20.83 | 18.63 | 129 | 17 | 57.92 | 302 | 69 | 6 |
| 3302 | 35.17 | -2.46 | GBIF | 11.98 | 76.27 | 19.73 | 17.22 | 125 | 11 | 68.94 | 347 | 40 | 5 |
| 3303 | 34.78 | -2.03 | GBIF | 12.01 | 79.53 | 21.83 | 20.13 | 154 | 20 | 50.63 | 292 | 100 | 5 |
| 3304 | 35.46 | -3.13 | GBIF | 10.62 | 69.39 | 18.40 | 15.68 | 157 | 1 | 88.23 | 399 | 7 | 8 |
| 3305 | 34.92 | -2.40 | GBIF | 12.00 | 76.92 | 21.15 | 18.92 | 130 | 17 | 59.05 | 301 | 68 | 5 |
| 3306 | 37.37 | -7.11 | GBIF | 9.36 | 65.90 | 23.98 | 20.48 | 242 | 3 | 86.79 | 392 | 18 | 11 |
| 3307 | 34.61 | -2.00 | GBIF | 11.77 | 80.65 | 21.63 | 20.05 | 167 | 19 | 50.44 | 304 | 103 | 4 |
| 3308 | 35.47 | -3.22 | GBIF | 8.82 | 64.83 | 14.37 | 11.77 | 217 | 10 | 75.90 | 458 | 44 | 11 |
| 3309 | 35.77 | -3.05 | GBIF | 10.67 | 71.59 | 10.83 | 8.27 | 236 | 10 | 73.94 | 515 | 42 | 5 |
| 3310 | 35.99 | -3.22 | GBIF | 12.02 | 72.83 | 23.65 | 20.63 | 130 | 1 | 93.42 | 202 | 5 | 7 |
| 3311 | 35.70 | -3.75 | GBIF | 10.47 | 67.97 | 23.08 | 19.60 | 154 | 3 | 87.67 | 342 | 20 | 10 |
| 3312 | 35.85 | -3.47 | GBIF | 9.60 | 66.67 | 23.35 | 19.82 | 142 | 2 | 88.35 | 309 | 13 | 0 |
| 3313 | 35.54 | -3.27 | GBIF | 8.38 | 62.04 | 16.22 | 13.77 | 271 | 13 | 80.34 | 525 | 61 | 11 |
| 3314 | 35.43 | -3.09 | GBIF | 12.31 | 74.15 | 21.27 | 18.23 | 95 | 1 | 88.47 | 265 | 5 | 5 |
| 3315 | 34.89 | -7.65 | GBIF | 11.99 | 67.75 | 23.47 | 20.42 | 129 | 0 | 108.74 | 153 | 0 | 9 |
| 3316 | 35.44 | -3.22 | GBIF | 9.34 | 67.21 | 14.03 | 11.63 | 212 | 8 | 77.28 | 319 | 36 | 3 |
| 3317 | 34.84 | -2.60 | GBIF | 12.05 | 74.84 | 20.53 | 18.12 | 132 | 10 | 66.93 | 321 | 46 | 8 |
| 3318 | 35.60 | -3.14 | GBIF | 10.98 | 70.86 | 19.40 | 16.62 | 172 | 2 | 88.93 | 390 | 12 | 4 |
| 3319 | 34.61 | -1.90 | GBIF | 11.57 | 80.32 | 21.05 | 19.67 | 178 | 22 | 48.76 | 314 | 113 | 14 |
| 3320 | 34.97 | -7.61 | GBIF | 12.22 | 67.92 | 24.15 | 21.12 | 125 | 0 | 107.71 | 149 | 0 | 4 |
| 3321 | 34.03 | -7.20 | GBIF | 12.23 | 68.30 | 22.28 | 19.85 | 147 | 0 | 111.92 | 191 | 0 | 3 |
| 3322 | 37.54 | -7.17 | GBIF | 9.03 | 65.46 | 23.92 | 20.23 | 307 | 9 | 81.97 | 448 | 37 | 8 |
| 3323 | 34.85 | -7.62 | GBIF | 12.08 | 67.88 | 23.68 | 20.63 | 130 | 0 | 107.87 | 153 | 0 | 6 |
| 3324 | 34.89 | -7.62 | GBIF | 12.19 | 67.73 | 23.88 | 20.83 | 128 | 0 | 108.05 | 152 | 0 | 9 |
| 3325 | 37.10 | -7.36 | GBIF | 9.43 | 60.81 | 25.40 | 21.37 | 289 | 7 | 86.83 | 431 | 37 | 15 |
| 3326 | 34.98 | -7.62 | GBIF | 12.21 | 67.82 | 24.23 | 21.18 | 126 | 0 | 107.29 | 150 | 0 | 4 |
| 3327 | 35.60 | -3.44 | GBIF | 11.45 | 71.12 | 21.62 | 18.58 | 186 | 2 | 91.00 | 396 | 14 | 13 |
| 3328 | 38.03 | -7.77 | GBIF | 8.23 | 64.76 | 27.87 | 24.52 | 203 | 7 | 80.71 | 404 | 33 | 5 |
| 3329 | 36.15 | -3.83 | GBIF | 11.07 | 70.49 | 21.75 | 18.63 | 171 | 1 | 95.09 | 374 | 7 | 5 |
| 3330 | 36.08 | -3.70 | GBIF | 11.74 | 71.60 | 22.75 | 19.55 | 164 | 1 | 92.19 | 352 | 10 | 5 |
| 3331 | 34.89 | -2.44 | GBIF | 12.08 | 76.48 | 20.90 | 18.63 | 131 | 14 | 61.60 | 312 | 61 | 5 |
| 3332 | 35.82 | -3.64 | GBIF | 9.55 | 66.32 | 23.48 | 19.78 | 133 | 2 | 87.89 | 311 | 12 | 0 |
| 3333 | 35.47 | -3.11 | GBIF | 10.67 | 69.72 | 19.07 | 16.35 | 147 | 1 | 90.21 | 382 | 5 | 5 |
| 3334 | 35.08 | -1.86 | GBIF | 12.15 | 79.41 | 20.15 | 18.22 | 161 | 23 | 51.80 | 383 | 98 | 5 |
| 3335 | 34.82 | -2.39 | GBIF | 11.94 | 77.04 | 21.42 | 19.30 | 129 | 17 | 57.00 | 300 | 73 | 13 |
| 3336 | 34.91 | -7.76 | GBIF | 11.57 | 68.04 | 23.13 | 20.18 | 135 | 0 | 106.55 | 162 | 0 | 4 |
| 3337 | 38.08 | -3.91 | GBIF | 10.71 | 68.64 | 24.78 | 20.93 | 115 | 7 | 75.72 | 214 | 29 | 6 |
| 3338 | 36.11 | -4.05 | GBIF | 11.46 | 71.61 | 22.58 | 19.47 | 146 | 1 | 92.88 | 344 | 8 | 7 |
| 3339 | 35.70 | -3.11 | GBIF | 9.76 | 67.77 | 14.62 | 11.98 | 220 | 8 | 79.34 | 459 | 36 | 4 |
| 3340 | 34.89 | -2.30 | GBIF | 12.22 | 77.81 | 21.60 | 19.48 | 128 | 19 | 55.25 | 294 | 79 | 5 |
| 3341 | 35.15 | -2.99 | GBIF | 13.13 | 75.43 | 20.67 | 17.62 | 122 | 3 | 85.45 | 304 | 12 | 5 |
| 3342 | 36.03 | -4.09 | GBIF | 11.38 | 71.15 | 22.45 | 19.43 | 147 | 1 | 92.36 | 249 | 8 | 8 |
| 3343 | 35.45 | -3.30 | GBIF | 8.06 | 62.96 | 14.70 | 11.98 | 220 | 3 | 82.79 | 473 | 20 | 3 |
| 3344 | 35.67 | -3.52 | GBIF | 10.89 | 69.37 | 21.35 | 18.20 | 171 | 1 | 92.51 | 377 | 9 | 13 |
| 3345 | 34.82 | -2.19 | GBIF | 12.22 | 78.82 | 21.62 | 19.78 | 140 | 20 | 51.86 | 349 | 90 | 4 |
| 3346 | 34.91 | -2.47 | GBIF | 12.13 | 76.74 | 20.73 | 18.45 | 132 | 14 | 61.84 | 315 | 60 | 6 |
| 3347 | 35.55 | -3.29 | GBIF | 9.61 | 65.81 | 18.87 | 16.37 | 265 | 7 | 90.14 | 515 | 37 | 4 |
| 3348 | 35.58 | -3.14 | GBIF | 10.93 | 70.54 | 19.38 | 16.60 | 171 | 2 | 88.39 | 387 | 13 | 4 |
| 3349 | 35.42 | -3.20 | GBIF | 9.66 | 67.54 | 14.22 | 12.00 | 210 | 4 | 80.51 | 324 | 23 | 4 |

| 3350 | 34.71 | -2.38 | GBIF | 11.88 | 77.61 | 21.68 | 19.75 | 129 | 16 | 56.56 | 301 | 74 | 5 |
| --- | --- | --- | --- | --- | --- | --- | --- | --- | --- | --- | --- | --- | --- |
| 3351 | 34.87 | -7.55 | GBIF | 11.71 | 66.90 | 23.35 | 20.23 | 125 | 0 | 109.46 | 154 | 0 | 5 |
| 3352 | 35.90 | -3.74 | GBIF | 11.67 | 70.71 | 23.57 | 20.35 | 150 | 2 | 88.36 | 337 | 14 | 11 |
| 3353 | 35.99 | -3.75 | GBIF | 11.74 | 71.16 | 23.20 | 20.05 | 157 | 1 | 90.01 | 345 | 10 | 8 |
| 3354 | 35.89 | -3.69 | GBIF | 11.22 | 69.29 | 23.60 | 20.15 | 157 | 2 | 89.78 | 342 | 14 | 10 |
| 3355 | 34.87 | -2.53 | GBIF | 12.13 | 76.31 | 20.68 | 18.37 | 129 | 13 | 63.76 | 315 | 55 | 5 |
| 3356 | 36.07 | -4.03 | GBIF | 11.57 | 71.89 | 22.90 | 19.82 | 142 | 1 | 90.70 | 234 | 10 | 8 |
| 3357 | 34.85 | -2.42 | GBIF | 12.02 | 76.59 | 21.13 | 18.95 | 130 | 16 | 59.01 | 305 | 68 | 9 |
| 3358 | 34.62 | -2.29 | GBIF | 12.27 | 78.63 | 22.23 | 20.58 | 138 | 15 | 56.11 | 293 | 76 | 8 |
| 3359 | 35.52 | -3.09 | GBIF | 9.73 | 67.59 | 17.60 | 14.93 | 187 | 1 | 89.03 | 425 | 8 | 4 |
| 3360 | 36.10 | -4.00 | GBIF | 11.57 | 71.45 | 22.90 | 19.72 | 143 | 1 | 91.15 | 264 | 8 | 8 |
| 3361 | 34.86 | -2.25 | GBIF | 12.23 | 78.37 | 21.58 | 19.57 | 134 | 20 | 53.63 | 294 | 84 | 4 |
| 3362 | 34.85 | -2.49 | GBIF | 12.21 | 76.78 | 20.87 | 18.63 | 128 | 13 | 61.81 | 309 | 57 | 5 |
| 3363 | 34.65 | -2.78 | GBIF | 10.92 | 72.30 | 18.50 | 16.32 | 157 | 7 | 72.46 | 379 | 33 | 6 |
| 3364 | 37.15 | -2.61 | GBIF | 12.93 | 74.33 | 22.78 | 20.12 | 130 | 2 | 83.33 | 257 | 15 | 3 |
| 3365 | 35.50 | -3.00 | GBIF | 11.54 | 72.59 | 21.90 | 18.92 | 107 | 1 | 86.99 | 272 | 5 | 4 |
| 3366 | 36.07 | -2.54 | GBIF | 10.36 | 71.44 | 24.00 | 20.88 | 116 | 1 | 89.52 | 181 | 5 | 5 |
| 3367 | 36.14 | -4.04 | GBIF | 11.32 | 71.17 | 22.57 | 19.33 | 145 | 1 | 92.61 | 340 | 8 | 7 |
| 3368 | 36.02 | -4.11 | GBIF | 11.32 | 70.78 | 22.72 | 19.60 | 143 | 1 | 92.56 | 245 | 8 | 7 |
| 3369 | 36.99 | -3.30 | GBIF | 10.70 | 65.64 | 21.50 | 18.62 | 312 | 20 | 97.51 | 234 | 85 | 7 |
| 3370 | 36.10 | -4.05 | GBIF | 11.55 | 71.74 | 22.78 | 19.67 | 141 | 1 | 90.77 | 334 | 9 | 8 |
| 3371 | 36.07 | -3.95 | GBIF | 11.38 | 71.15 | 22.95 | 19.80 | 143 | 1 | 92.23 | 333 | 8 | 8 |
| 3372 | 34.71 | -2.42 | GBIF | 11.63 | 77.04 | 21.62 | 19.58 | 128 | 15 | 57.66 | 301 | 69 | 5 |
| 3373 | 37.02 | -7.21 | GBIF | 8.99 | 62.88 | 24.73 | 21.47 | 254 | 7 | 86.25 | 398 | 36 | 5 |
| 3374 | 38.03 | -7.60 | GBIF | 9.02 | 66.85 | 28.28 | 25.07 | 235 | 8 | 78.89 | 415 | 38 | 4 |
| 3375 | 35.97 | -4.06 | GBIF | 10.96 | 70.70 | 22.32 | 19.33 | 145 | 0 | 92.31 | 351 | 7 | 7 |
| 3376 | 36.98 | -6.83 | GBIF | 10.11 | 65.22 | 25.63 | 22.63 | 199 | 9 | 81.69 | 346 | 38 | 20 |
| 3377 | 33.00 | -2.05 | GBIF | 10.23 | 80.58 | 22.18 | 21.35 | 211 | 24 | 57.40 | 310 | 88 |  |
| 3378 | 35.00 | -6.00 | GBIF | 12.45 | 69.94 | 25.42 | 21.80 | 129 | 0 | 109.89 | 292 | 0 | 6 |
| 3379 | 35.03 | -2.65 | GBIF | 11.79 | 75.11 | 19.68 | 17.00 | 130 | 8 | 72.10 | 324 | 33 | 7 |
| 3380 | 35.75 | -3.68 | GBIF | 9.80 | 67.12 | 23.22 | 19.63 | 129 | 1 | 92.18 | 299 | 8 | 6 |
| 3381 | 34.86 | -2.51 | GBIF | 12.16 | 76.47 | 20.77 | 18.50 | 128 | 13 | 62.57 | 313 | 55 | 5 |
| 3382 | 35.03 | -2.57 | GBIF | 12.06 | 75.84 | 20.02 | 17.47 | 126 | 10 | 68.55 | 317 | 41 | 8 |
| 3383 | 35.17 | -2.60 | GBIF | 12.43 | 75.35 | 19.70 | 16.98 | 121 | 9 | 73.16 | 310 | 31 | 9 |
| 3384 | 34.92 | -2.51 | GBIF | 12.09 | 76.53 | 20.47 | 18.12 | 133 | 14 | 61.77 | 327 | 60 | 6 |
| 3385 | 36.12 | -4.16 | GBIF | 11.68 | 71.24 | 22.60 | 19.63 | 141 | 1 | 91.94 | 242 | 8 | 7 |
| 3386 | 35.96 | -3.69 | GBIF | 12.09 | 70.71 | 23.62 | 20.40 | 144 | 1 | 90.22 | 319 | 11 | 8 |
| 3387 | 35.83 | -3.52 | GBIF | 9.35 | 66.31 | 23.42 | 19.80 | 136 | 1 | 88.14 | 306 | 11 | 0 |
| 3388 | 38.14 | -7.65 | GBIF | 8.70 | 66.92 | 28.20 | 24.98 | 218 | 7 | 78.48 | 401 | 32 | 6 |
| 3389 | 35.95 | -3.66 | GBIF | 12.12 | 70.86 | 23.58 | 20.42 | 146 | 1 | 90.17 | 321 | 11 | 15 |
| 3390 | 35.54 | -3.06 | GBIF | 9.53 | 67.61 | 18.25 | 15.55 | 182 | 1 | 91.13 | 415 | 6 | 4 |
| 3391 | 35.75 | -3.41 | GBIF | 10.85 | 69.55 | 22.15 | 19.03 | 175 | 1 | 93.79 | 363 | 11 | 7 |
| 3392 | 34.61 | -2.65 | GBIF | 11.42 | 74.13 | 19.83 | 17.85 | 144 | 9 | 67.20 | 345 | 43 | 6 |
| 3393 | 36.05 | -4.31 | GBIF | 11.50 | 70.99 | 23.07 | 20.05 | 129 | 1 | 91.05 | 243 | 9 | 7 |
| 3394 | 36.08 | -4.20 | GBIF | 11.56 | 71.35 | 22.90 | 19.93 | 139 | 1 | 91.62 | 242 | 8 | 7 |
| 3395 | 37.10 | -7.24 | GBIF | 9.68 | 62.02 | 25.83 | 22.38 | 276 | 8 | 85.33 | 404 | 41 | 5 |
| 3396 | 35.88 | -3.26 | GBIF | 10.88 | 69.27 | 20.50 | 17.52 | 203 | 3 | 93.11 | 294 | 15 | 7 |
| 3397 | 35.69 | -3.42 | GBIF | 11.13 | 69.97 | 21.28 | 18.23 | 203 | 2 | 93.67 | 415 | 15 | 12 |
| 3398 | 35.94 | -3.21 | GBIF | 11.03 | 70.28 | 23.37 | 20.22 | 122 | 1 | 92.04 | 197 | 6 | 8 |
| 3399 | 34.72 | -2.71 | GBIF | 11.92 | 74.48 | 20.38 | 18.05 | 139 | 8 | 71.10 | 333 | 36 | 7 |

| 3400 | 35.77 | -3.59 | GBIF | 9.98 | 67.45 | 23.37 | 19.90 | 111 | 1 | 91.36 | 274 | 7 | 0 |
| --- | --- | --- | --- | --- | --- | --- | --- | --- | --- | --- | --- | --- | --- |
| 3401 | 35.45 | -3.32 | GBIF | 7.79 | 61.84 | 13.80 | 11.00 | 240 | 9 | 77.56 | 509 | 41 | 4 |
| 3402 | 35.64 | -3.47 | GBIF | 11.36 | 70.55 | 21.70 | 18.60 | 178 | 1 | 92.50 | 382 | 10 | 16 |
| 3403 | 35.50 | -3.11 | GBIF | 10.26 | 68.39 | 18.33 | 15.60 | 168 | 1 | 89.12 | 406 | 8 | 4 |
| 3404 | 35.60 | -3.13 | GBIF | 10.40 | 69.33 | 18.67 | 15.87 | 185 | 3 | 88.07 | 411 | 15 | 4 |
| 3405 | 35.64 | -3.41 | GBIF | 11.44 | 70.19 | 20.83 | 18.53 | 197 | 2 | 91.49 | 406 | 18 | 13 |
| 3406 | 35.94 | -3.37 | GBIF | 11.99 | 72.24 | 23.53 | 20.42 | 150 | 0 | 93.64 | 215 | 7 | 6 |
| 3407 | 34.63 | -2.52 | GBIF | 11.83 | 76.34 | 21.15 | 19.25 | 132 | 12 | 61.79 | 314 | 56 | 5 |
| 3408 | 34.61 | -2.55 | GBIF | 11.70 | 75.97 | 21.13 | 19.28 | 138 | 11 | 64.29 | 315 | 52 | 6 |
| 3409 | 35.85 | -3.62 | GBIF | 9.47 | 66.20 | 23.40 | 19.73 | 143 | 1 | 90.13 | 321 | 11 | 6 |
| 3410 | 34.62 | -2.54 | GBIF | 11.92 | 76.39 | 21.33 | 19.48 | 132 | 11 | 62.77 | 310 | 53 | 6 |
| 3411 | 34.79 | -1.97 | GBIF | 11.81 | 80.33 | 21.13 | 19.68 | 157 | 22 | 48.02 | 300 | 109 | 6 |
| 3412 | 35.49 | -3.02 | GBIF | 11.51 | 72.38 | 21.97 | 18.95 | 104 | 1 | 87.40 | 270 | 5 | 4 |
| 3413 | 35.50 | -3.08 | GBIF | 10.62 | 70.31 | 19.97 | 16.87 | 128 | 1 | 90.61 | 334 | 4 | 4 |
| 3414 | 35.70 | -3.60 | GBIF | 9.27 | 66.67 | 19.07 | 16.52 | 227 | 2 | 93.48 | 465 | 13 | 13 |
| 3415 | 34.81 | -2.22 | GBIF | 12.26 | 78.08 | 21.75 | 19.88 | 134 | 19 | 51.35 | 292 | 89 | 4 |
| 3416 | 36.18 | -3.72 | GBIF | 11.48 | 71.27 | 22.48 | 19.25 | 166 | 1 | 93.19 | 355 | 10 | 4 |
| 3417 | 35.59 | -3.01 | GBIF | 8.25 | 63.46 | 13.42 | 10.53 | 258 | 6 | 80.29 | 519 | 30 | 10 |
| 3418 | 34.90 | -2.79 | GBIF | 12.24 | 75.10 | 20.15 | 17.50 | 135 | 7 | 74.31 | 330 | 28 | 8 |
| 3419 | 34.93 | -2.44 | GBIF | 12.05 | 76.75 | 20.98 | 18.68 | 129 | 15 | 61.27 | 307 | 61 | 4 |
| 3420 | 34.82 | -2.76 | GBIF | 12.27 | 74.85 | 20.45 | 18.02 | 136 | 7 | 71.97 | 329 | 32 | 7 |
| 3421 | 35.89 | -3.64 | GBIF | 10.71 | 68.64 | 23.52 | 20.03 | 165 | 3 | 86.69 | 354 | 18 | 6 |
| 3422 | 35.93 | -3.72 | GBIF | 11.97 | 71.23 | 23.53 | 20.32 | 146 | 1 | 90.14 | 325 | 11 | 11 |
| 3423 | 36.02 | -3.88 | GBIF | 10.91 | 71.30 | 22.37 | 19.27 | 153 | 1 | 92.41 | 349 | 8 | 8 |
| 3424 | 35.50 | -3.35 | GBIF | 9.35 | 65.38 | 18.15 | 15.72 | 274 | 9 | 87.06 | 547 | 44 | 7 |
| 3425 | 36.08 | -3.63 | GBIF | 12.01 | 71.48 | 23.20 | 19.97 | 155 | 1 | 92.40 | 240 | 9 | 6 |
| 3426 | 36.12 | -3.77 | GBIF | 11.21 | 70.49 | 22.68 | 19.47 | 156 | 1 | 92.56 | 343 | 9 | 4 |
| 3427 | 35.45 | -3.00 | GBIF | 12.81 | 74.90 | 22.38 | 19.38 | 89 | 2 | 86.13 | 253 | 7 | 5 |
| 3428 | 35.87 | -3.70 | GBIF | 10.56 | 67.68 | 23.48 | 19.92 | 168 | 3 | 87.07 | 365 | 18 | 6 |
| 3429 | 36.14 | -4.01 | GBIF | 11.38 | 71.09 | 22.53 | 19.32 | 150 | 1 | 92.76 | 346 | 8 | 7 |
| 3430 | 36.06 | -4.02 | GBIF | 11.51 | 71.48 | 22.70 | 19.62 | 144 | 1 | 91.47 | 341 | 8 | 8 |
| 3431 | 36.19 | -4.16 | GBIF | 11.44 | 71.51 | 22.57 | 19.58 | 139 | 1 | 91.57 | 241 | 8 | 7 |
| 3432 | 37.93 | -9.21 | GBIF | 8.49 | 63.37 | 25.78 | 22.67 | 190 | 1 | 98.33 | 392 | 7 | 3 |
| 3433 | 36.04 | -4.09 | GBIF | 11.41 | 71.30 | 22.47 | 19.45 | 144 | 1 | 92.01 | 249 | 8 | 8 |
| 3434 | 35.42 | -3.21 | GBIF | 9.78 | 67.94 | 13.50 | 10.92 | 211 | 5 | 79.07 | 339 | 26 | 3 |
| 3435 | 34.94 | -2.55 | GBIF | 12.03 | 76.16 | 20.32 | 17.87 | 128 | 11 | 66.66 | 317 | 46 | 7 |
| 3436 | 34.38 | -2.34 | GBIF | 12.26 | 77.58 | 22.25 | 20.87 | 143 | 10 | 59.55 | 167 | 64 | 6 |
| 3437 | 35.59 | -3.04 | GBIF | 8.40 | 63.64 | 14.75 | 11.70 | 240 | 3 | 84.62 | 486 | 18 | 10 |
| 3438 | 35.08 | -3.03 | GBIF | 12.58 | 74.02 | 20.40 | 17.37 | 136 | 2 | 85.62 | 330 | 11 | 6 |
| 3439 | 35.18 | -3.19 | GBIF | 12.13 | 72.22 | 19.55 | 16.43 | 167 | 0 | 92.03 | 384 | 4 | 4 |
| 3440 | 36.17 | -4.13 | GBIF | 11.58 | 71.95 | 22.65 | 19.67 | 141 | 1 | 91.91 | 239 | 7 | 7 |
| 3441 | 34.91 | -2.38 | GBIF | 12.07 | 76.91 | 21.27 | 19.03 | 133 | 17 | 59.21 | 300 | 69 | 5 |
| 3442 | 34.46 | -3.39 | GBIF | 11.38 | 71.15 | 21.92 | 20.25 | 152 | 2 | 87.37 | 109 | 8 | 7 |
| 3443 | 36.03 | -3.94 | GBIF | 11.13 | 71.31 | 22.23 | 19.15 | 149 | 0 | 92.21 | 351 | 7 | 9 |
| 3444 | 37.12 | -2.73 | GBIF | 13.02 | 73.54 | 22.65 | 20.00 | 145 | 4 | 82.55 | 176 | 21 | 4 |
| 3445 | 35.93 | -3.62 | GBIF | 11.85 | 70.96 | 23.63 | 20.38 | 153 | 1 | 91.38 | 327 | 11 | 6 |
| 3446 | 37.30 | -7.09 | GBIF | 9.91 | 64.76 | 25.17 | 21.58 | 249 | 4 | 85.81 | 375 | 29 | 5 |
| 3447 | 34.89 | -2.10 | GBIF | 12.26 | 79.60 | 21.52 | 19.72 | 147 | 20 | 52.26 | 295 | 91 | 5 |
| 3448 | 36.06 | -4.16 | GBIF | 11.55 | 70.86 | 23.05 | 20.02 | 143 | 1 | 90.10 | 244 | 10 | 7 |
| 3449 | 37.25 | -7.19 | GBIF | 9.73 | 63.56 | 25.28 | 21.55 | 270 | 5 | 86.27 | 387 | 32 | 6 |

| 3450 | 38.35 | -7.61 | GBIF | 8.54 | 67.26 | 27.93 | 24.83 | 214 | 8 | 76.23 | 363 | 36 | 5 |
| --- | --- | --- | --- | --- | --- | --- | --- | --- | --- | --- | --- | --- | --- |
| 3451 | 36.01 | -3.73 | GBIF | 11.81 | 70.71 | 23.22 | 20.05 | 150 | 1 | 91.48 | 330 | 10 | 8 |
| 3452 | 36.18 | -3.64 | GBIF | 12.11 | 72.07 | 22.97 | 19.78 | 151 | 1 | 91.86 | 242 | 9 | 5 |
| 3453 | 35.94 | -3.60 | GBIF | 11.82 | 70.76 | 23.60 | 20.40 | 156 | 1 | 92.21 | 326 | 11 | 6 |
| 3454 | 36.11 | -3.78 | GBIF | 11.26 | 70.36 | 22.70 | 19.48 | 157 | 1 | 92.50 | 345 | 10 | 4 |
| 3455 | 35.42 | -3.18 | GBIF | 9.60 | 68.57 | 14.88 | 12.60 | 204 | 3 | 80.92 | 447 | 21 | 4 |
| 3456 | 36.81 | -7.84 | GBIF | 7.59 | 61.72 | 21.62 | 18.85 | 309 | 4 | 89.05 | 525 | 46 | 4 |
| 3457 | 36.86 | -7.82 | GBIF | 7.49 | 61.91 | 21.58 | 18.82 | 320 | 7 | 86.59 | 511 | 60 | 4 |
| 3458 | 36.84 | -7.84 | GBIF | 7.42 | 62.39 | 21.17 | 18.43 | 308 | 6 | 86.79 | 527 | 54 | 4 |
| 3459 | 36.97 | -7.74 | GBIF | 8.06 | 58.82 | 26.33 | 23.33 | 351 | 11 | 88.25 | 463 | 56 | 9 |
| 3460 | 37.16 | -7.34 | GBIF | 9.30 | 60.78 | 25.33 | 21.20 | 280 | 6 | 86.86 | 424 | 35 | 14 |
| 3461 | 37.01 | -7.21 | GBIF | 9.01 | 63.00 | 24.57 | 21.37 | 251 | 7 | 86.28 | 398 | 36 | 5 |
| 3462 | 37.17 | -7.31 | GBIF | 9.26 | 61.31 | 25.35 | 21.80 | 271 | 6 | 84.76 | 418 | 37 | 10 |
| 3463 | 37.08 | -7.33 | GBIF | 9.78 | 61.53 | 25.68 | 22.25 | 284 | 8 | 85.88 | 422 | 41 | 7 |
| 3464 | 37.02 | -7.33 | GBIF | 9.47 | 61.47 | 25.30 | 21.97 | 276 | 7 | 86.75 | 423 | 39 | 6 |
| 3465 | 35.44 | -3.37 | GBIF | 9.25 | 66.55 | 18.18 | 15.23 | 218 | 3 | 86.01 | 484 | 20 | 7 |
| 3466 | 34.92 | -2.32 | GBIF | 12.26 | 77.58 | 21.58 | 19.45 | 125 | 19 | 54.63 | 293 | 79 | 6 |
| 3467 | 36.30 | -4.40 | GBIF | 11.74 | 71.16 | 23.00 | 19.97 | 120 | 1 | 91.57 | 232 | 7 | 6 |
| 3468 | 36.02 | -4.08 | GBIF | 11.32 | 71.17 | 22.43 | 19.42 | 142 | 0 | 92.64 | 247 | 7 | 8 |
| 3469 | 36.44 | -4.37 | GBIF | 11.28 | 70.91 | 22.33 | 19.17 | 127 | 1 | 92.93 | 228 | 6 | 3 |
| 3470 | 35.71 | -3.72 | GBIF | 9.57 | 67.37 | 21.65 | 18.20 | 180 | 2 | 90.87 | 386 | 16 | 21 |
| 3471 | 36.13 | -4.03 | GBIF | 11.38 | 71.09 | 22.55 | 19.33 | 147 | 1 | 92.82 | 344 | 8 | 7 |
| 3472 | 36.16 | -4.12 | GBIF | 11.58 | 71.50 | 22.65 | 19.63 | 145 | 1 | 92.24 | 237 | 8 | 7 |
| 3473 | 36.03 | -3.83 | GBIF | 11.39 | 71.20 | 22.62 | 19.52 | 148 | 1 | 92.51 | 339 | 7 | 8 |
| 3474 | 35.72 | -3.45 | GBIF | 10.84 | 69.50 | 20.90 | 18.50 | 196 | 1 | 94.08 | 401 | 12 | 9 |
| 3475 | 36.15 | -4.14 | GBIF | 11.65 | 71.47 | 22.70 | 19.72 | 140 | 1 | 91.49 | 236 | 8 | 7 |
| 3476 | 35.95 | -3.79 | GBIF | 11.43 | 70.58 | 23.20 | 20.12 | 149 | 1 | 91.00 | 329 | 10 | 8 |
| 3477 | 35.79 | -3.38 | GBIF | 11.19 | 70.39 | 22.33 | 19.25 | 174 | 1 | 94.39 | 357 | 9 | 7 |
| 3478 | 36.75 | -2.81 | GBIF | 11.92 | 72.22 | 22.77 | 19.33 | 121 | 0 | 89.02 | 188 | 4 | 7 |
| 3479 | 35.93 | -3.65 | GBIF | 11.89 | 70.36 | 23.57 | 20.32 | 148 | 1 | 91.27 | 322 | 11 | 14 |
| 3480 | 35.94 | -3.30 | GBIF | 11.94 | 71.94 | 23.77 | 20.67 | 153 | 2 | 89.08 | 225 | 12 | 15 |
| 3481 | 36.11 | -3.64 | GBIF | 11.90 | 71.69 | 22.73 | 19.55 | 162 | 1 | 92.37 | 347 | 10 | 5 |
| 3482 | 35.87 | -3.36 | GBIF | 10.84 | 69.95 | 23.40 | 20.17 | 146 | 1 | 91.41 | 306 | 10 | 10 |
| 3483 | 36.16 | -3.67 | GBIF | 11.93 | 71.46 | 22.98 | 19.77 | 154 | 1 | 92.31 | 332 | 9 | 5 |
| 3484 | 35.54 | -3.17 | GBIF | 11.99 | 70.96 | 19.70 | 16.87 | 155 | 3 | 85.99 | 370 | 15 | 4 |
| 3485 | 35.89 | -3.58 | GBIF | 10.40 | 68.42 | 23.43 | 20.00 | 165 | 2 | 89.60 | 351 | 15 | 5 |
| 3486 | 35.88 | -3.74 | GBIF | 11.58 | 70.63 | 23.55 | 20.32 | 165 | 3 | 86.95 | 362 | 18 | 15 |
| 3487 | 35.85 | -3.40 | GBIF | 10.48 | 68.97 | 23.33 | 20.02 | 137 | 1 | 91.99 | 292 | 10 | 13 |
| 3488 | 35.48 | -3.01 | GBIF | 12.03 | 73.77 | 22.18 | 19.25 | 97 | 2 | 86.33 | 260 | 6 | 4 |
| 3489 | 35.55 | -3.10 | GBIF | 9.50 | 66.90 | 16.70 | 13.98 | 225 | 5 | 85.10 | 462 | 24 | 4 |
| 3490 | 36.03 | -3.66 | GBIF | 12.03 | 71.20 | 23.38 | 20.13 | 152 | 1 | 91.53 | 326 | 10 | 6 |
| 3491 | 35.88 | -3.43 | GBIF | 10.51 | 68.68 | 23.43 | 20.07 | 144 | 1 | 92.79 | 220 | 9 | 6 |
| 3492 | 34.94 | -2.38 | GBIF | 12.08 | 76.91 | 21.12 | 18.87 | 129 | 16 | 59.12 | 302 | 67 | 5 |
| 3493 | 35.88 | -3.47 | GBIF | 9.93 | 67.52 | 23.37 | 19.90 | 154 | 2 | 88.61 | 330 | 14 | 0 |
| 3494 | 35.40 | -3.19 | GBIF | 9.60 | 68.09 | 13.92 | 11.33 | 206 | 4 | 79.29 | 331 | 24 | 4 |
| 3495 | 35.54 | -3.32 | GBIF | 9.98 | 67.00 | 19.17 | 16.73 | 256 | 6 | 89.89 | 503 | 34 | 5 |
| 3496 | 36.11 | -4.01 | GBIF | 11.55 | 71.30 | 22.72 | 19.53 | 144 | 1 | 92.15 | 339 | 7 | 7 |
| 3497 | 34.95 | -7.64 | GBIF | 12.24 | 68.01 | 24.08 | 21.07 | 128 | 0 | 107.73 | 152 | 0 | 4 |
| 3498 | 34.98 | -7.69 | GBIF | 11.89 | 67.95 | 23.93 | 20.98 | 130 | 0 | 106.32 | 154 | 0 | 4 |
| 3499 | 35.74 | -3.51 | GBIF | 10.42 | 68.98 | 21.62 | 18.42 | 180 | 1 | 94.18 | 381 | 10 | 13 |

| 3500 | 34.86 | -2.48 | GBIF | 12.17 | 76.52 | 20.87 | 18.63 | 128 | 13 | 61.56 | 309 | 58 | 8 |
| --- | --- | --- | --- | --- | --- | --- | --- | --- | --- | --- | --- | --- | --- |
| 3501 | 34.97 | -7.66 | GBIF | 12.18 | 68.06 | 24.35 | 21.33 | 131 | 0 | 106.91 | 153 | 0 | 4 |
| 3502 | 37.18 | -7.34 | GBIF | 8.93 | 60.77 | 24.90 | 20.87 | 269 | 5 | 85.80 | 422 | 32 | 10 |
| 3503 | 37.14 | -7.33 | GBIF | 9.57 | 61.38 | 25.48 | 21.37 | 285 | 7 | 86.91 | 421 | 37 | 9 |
| 3504 | 35.63 | -3.05 | GBIF | 8.72 | 64.63 | 12.83 | 9.80 | 258 | 9 | 78.86 | 526 | 36 | 10 |
| 3505 | 37.10 | -7.34 | GBIF | 9.76 | 61.37 | 25.62 | 22.20 | 286 | 8 | 86.42 | 424 | 40 | 10 |
| 3506 | 36.12 | -3.81 | GBIF | 11.15 | 70.57 | 22.47 | 19.30 | 165 | 1 | 91.67 | 361 | 10 | 5 |
| 3507 | 36.13 | -3.97 | GBIF | 11.27 | 71.31 | 22.47 | 19.30 | 151 | 1 | 92.95 | 346 | 8 | 7 |
| 3508 | 36.20 | -3.89 | GBIF | 11.27 | 71.82 | 21.88 | 18.82 | 160 | 1 | 93.54 | 278 | 7 | 7 |
| 3509 | 34.84 | -2.42 | GBIF | 12.01 | 76.49 | 21.18 | 18.98 | 132 | 15 | 59.52 | 304 | 67 | 6 |
| 3510 | 35.69 | -3.69 | GBIF | 8.49 | 63.85 | 18.33 | 15.62 | 249 | 4 | 92.88 | 498 | 22 | 6 |
| 3511 | 34.19 | -2.17 | GBIF | 11.92 | 79.98 | 22.93 | 22.05 | 144 | 9 | 58.50 | 177 | 68 | 8 |
| 3512 | 36.84 | -3.26 | GBIF | 9.32 | 63.81 | 18.08 | 15.10 | 298 | 14 | 92.43 | 276 | 66 | 8 |
| 3513 | 34.94 | -2.32 | GBIF | 12.31 | 77.90 | 21.45 | 19.32 | 124 | 18 | 55.80 | 294 | 75 | 6 |
| 3514 | 36.01 | -4.00 | GBIF | 11.11 | 71.67 | 22.47 | 19.45 | 146 | 1 | 91.51 | 346 | 8 | 8 |
| 3515 | 34.72 | -2.51 | GBIF | 11.91 | 76.34 | 20.97 | 18.87 | 132 | 12 | 62.17 | 316 | 55 | 7 |
| 3516 | 35.41 | -3.23 | GBIF | 9.64 | 66.49 | 14.70 | 11.98 | 198 | 3 | 81.47 | 316 | 18 | 3 |
| 3517 | 36.63 | -2.91 | GBIF | 11.84 | 71.77 | 21.87 | 18.67 | 140 | 0 | 93.40 | 205 | 6 | 6 |
| 3518 | 36.18 | -4.18 | GBIF | 11.67 | 72.02 | 22.67 | 19.75 | 137 | 1 | 88.51 | 248 | 10 | 7 |
| 3519 | 34.70 | -2.36 | GBIF | 12.08 | 77.96 | 21.98 | 20.08 | 133 | 16 | 56.35 | 296 | 75 | 5 |
| 3520 | 35.90 | -3.46 | GBIF | 10.83 | 68.95 | 23.48 | 20.17 | 166 | 2 | 89.99 | 241 | 14 | 4 |
| 3521 | 35.92 | -3.74 | GBIF | 11.80 | 71.08 | 23.52 | 20.33 | 149 | 2 | 88.72 | 335 | 12 | 15 |
| 3522 | 36.13 | -4.11 | GBIF | 11.63 | 71.37 | 22.57 | 19.58 | 143 | 1 | 91.53 | 241 | 8 | 7 |
| 3523 | 35.60 | -3.05 | GBIF | 8.33 | 63.13 | 14.30 | 11.25 | 243 | 4 | 84.35 | 493 | 20 | 10 |
| 3524 | 36.14 | -4.09 | GBIF | 11.47 | 71.22 | 22.62 | 19.52 | 139 | 1 | 90.98 | 238 | 8 | 7 |
| 3525 | 36.20 | -3.96 | GBIF | 11.18 | 71.69 | 22.03 | 18.92 | 155 | 1 | 92.90 | 280 | 7 | 7 |
| 3526 | 34.86 | -2.47 | GBIF | 12.18 | 76.57 | 20.85 | 18.62 | 128 | 13 | 61.34 | 310 | 59 | 12 |
| 3527 | 36.00 | -3.74 | GBIF | 11.75 | 71.21 | 23.18 | 20.05 | 158 | 2 | 89.31 | 346 | 13 | 8 |
| 3528 | 35.90 | -3.72 | GBIF | 11.66 | 70.23 | 23.62 | 20.30 | 150 | 1 | 90.82 | 329 | 12 | 11 |
| 3529 | 35.82 | -3.47 | GBIF | 10.21 | 68.06 | 23.45 | 20.02 | 118 | 1 | 92.98 | 275 | 6 | 1 |
| 3530 | 36.04 | -4.04 | GBIF | 11.37 | 71.94 | 22.55 | 19.47 | 141 | 1 | 91.88 | 342 | 8 | 9 |
| 3531 | 36.13 | -3.65 | GBIF | 11.88 | 71.16 | 23.08 | 19.85 | 157 | 1 | 92.69 | 334 | 10 | 5 |
| 3532 | 36.13 | -3.93 | GBIF | 11.07 | 70.94 | 22.05 | 18.88 | 160 | 1 | 94.49 | 361 | 7 | 7 |
| 3533 | 36.11 | -3.75 | GBIF | 11.32 | 70.73 | 22.70 | 19.48 | 155 | 1 | 91.74 | 340 | 9 | 4 |
| 3534 | 36.00 | -4.15 | GBIF | 11.14 | 70.97 | 22.47 | 19.40 | 144 | 0 | 91.82 | 252 | 8 | 8 |
| 3535 | 35.96 | -3.78 | GBIF | 11.61 | 71.22 | 23.20 | 20.12 | 151 | 1 | 90.31 | 334 | 10 | 8 |
| 3536 | 36.14 | -4.63 | GBIF | 11.88 | 69.90 | 23.13 | 20.27 | 124 | 1 | 92.70 | 255 | 8 | 6 |
| 3537 | 36.19 | -3.62 | GBIF | 12.12 | 71.27 | 22.92 | 19.77 | 153 | 1 | 93.61 | 240 | 8 | 14 |
| 3538 | 34.91 | -7.78 | GBIF | 11.39 | 68.21 | 23.42 | 20.53 | 137 | 0 | 105.39 | 161 | 0 | 4 |
| 3539 | 34.88 | -2.20 | GBIF | 12.07 | 78.41 | 21.38 | 19.42 | 141 | 20 | 53.49 | 300 | 86 | 5 |
| 3540 | 36.90 | -3.21 | GBIF | 10.57 | 66.93 | 19.72 | 16.88 | 283 | 17 | 88.53 | 256 | 85 | 15 |
| 3541 | 34.97 | -2.31 | GBIF | 12.27 | 77.64 | 21.22 | 19.05 | 130 | 18 | 56.91 | 300 | 74 | 6 |
| 3542 | 35.80 | -3.33 | GBIF | 11.23 | 70.16 | 21.28 | 18.88 | 192 | 2 | 93.33 | 257 | 16 | 7 |
| 3543 | 38.14 | -4.10 | GBIF | 10.18 | 67.44 | 23.70 | 20.27 | 128 | 5 | 78.26 | 239 | 24 | 6 |
| 3544 | 35.76 | -3.10 | GBIF | 9.52 | 68.04 | 12.47 | 9.88 | 249 | 9 | 76.54 | 527 | 42 | 5 |
| 3545 | 36.15 | -4.05 | GBIF | 11.37 | 71.49 | 22.55 | 19.38 | 146 | 1 | 92.02 | 274 | 8 | 7 |
| 3546 | 34.90 | -2.20 | GBIF | 12.02 | 78.54 | 21.17 | 19.15 | 139 | 20 | 53.69 | 352 | 83 | 5 |
| 3547 | 35.94 | -3.42 | GBIF | 11.88 | 71.97 | 23.60 | 20.52 | 146 | 0 | 94.08 | 307 | 7 | 9 |
| 3548 | 37.17 | -7.38 | GBIF | 8.33 | 60.39 | 24.53 | 20.98 | 292 | 5 | 87.55 | 431 | 36 | 8 |
| 3549 | 35.88 | -3.34 | GBIF | 11.18 | 70.73 | 23.52 | 20.33 | 135 | 1 | 91.00 | 287 | 9 | 7 |

| 3550 | 37.20 | -7.26 | GBIF | 9.41 | 62.31 | 25.35 | 21.43 | 276 | 6 | 85.51 | 402 | 37 | 14 |
| --- | --- | --- | --- | --- | --- | --- | --- | --- | --- | --- | --- | --- | --- |
| 3551 | 35.97 | -3.22 | GBIF | 11.49 | 71.82 | 23.63 | 20.55 | 122 | 0 | 92.83 | 198 | 4 | 7 |
| 3552 | 37.14 | -7.25 | GBIF | 9.77 | 62.26 | 25.80 | 21.85 | 279 | 8 | 85.64 | 404 | 40 | 6 |
| 3553 | 35.38 | -2.57 | GBIF | 11.98 | 74.90 | 18.93 | 16.12 | 132 | 7 | 77.20 | 351 | 24 | 5 |
| 3554 | 35.83 | -3.46 | GBIF | 10.16 | 68.18 | 23.45 | 19.98 | 130 | 1 | 91.64 | 290 | 8 | 0 |
| 3555 | 36.02 | -3.64 | GBIF | 11.95 | 71.13 | 23.37 | 20.17 | 152 | 1 | 91.07 | 327 | 10 | 7 |
| 3556 | 36.06 | -3.93 | GBIF | 11.32 | 70.73 | 22.85 | 19.67 | 143 | 1 | 91.62 | 333 | 7 | 8 |
| 3557 | 36.00 | -3.71 | GBIF | 11.96 | 70.76 | 23.37 | 20.18 | 152 | 1 | 92.10 | 330 | 9 | 8 |
| 3558 | 35.90 | -3.65 | GBIF | 11.51 | 69.75 | 23.60 | 20.22 | 147 | 2 | 90.07 | 322 | 12 | 7 |
| 3559 | 36.18 | -3.62 | GBIF | 12.11 | 71.65 | 22.90 | 19.77 | 154 | 1 | 93.05 | 243 | 9 | 8 |
| 3560 | 36.19 | -3.70 | GBIF | 11.71 | 71.83 | 22.63 | 19.43 | 159 | 2 | 92.11 | 344 | 11 | 4 |
| 3561 | 36.05 | -3.93 | GBIF | 11.33 | 71.28 | 22.57 | 19.45 | 146 | 1 | 91.91 | 340 | 7 | 9 |
| 3562 | 35.48 | -3.13 | GBIF | 10.24 | 68.28 | 17.13 | 14.50 | 186 | 2 | 85.58 | 422 | 14 | 8 |
| 3563 | 35.17 | -2.80 | GBIF | 13.19 | 75.38 | 20.17 | 17.30 | 120 | 5 | 78.56 | 305 | 20 | 10 |
| 3564 | 34.86 | -2.45 | GBIF | 12.10 | 76.10 | 20.85 | 18.63 | 132 | 14 | 61.65 | 312 | 61 | 9 |
| 3565 | 35.74 | -3.45 | GBIF | 10.59 | 69.68 | 21.78 | 18.70 | 182 | 1 | 93.84 | 381 | 10 | 8 |
| 3566 | 34.57 | -2.24 | GBIF | 12.33 | 79.06 | 22.38 | 20.83 | 142 | 14 | 55.52 | 288 | 79 | 7 |
| 3567 | 35.00 | -2.44 | GBIF | 11.95 | 76.11 | 20.57 | 18.15 | 125 | 14 | 62.93 | 310 | 55 | 4 |
| 3568 | 34.96 | -2.52 | GBIF | 12.09 | 76.05 | 20.28 | 17.85 | 128 | 11 | 65.92 | 318 | 46 | 6 |
| 3569 | 35.69 | -3.17 | GBIF | 8.88 | 64.78 | 14.30 | 12.03 | 231 | 12 | 76.32 | 468 | 52 | 3 |
| 3570 | 37.14 | -3.12 | GBIF | 9.21 | 65.31 | 17.15 | 14.33 | 313 | 10 | 90.15 | 333 | 50 | 7 |
| 3571 | 36.01 | -4.28 | GBIF | 11.86 | 72.31 | 22.83 | 19.88 | 133 | 1 | 91.15 | 258 | 9 | 8 |
| 3572 | 36.91 | -3.38 | GBIF | 11.24 | 66.52 | 21.40 | 18.62 | 281 | 17 | 92.73 | 247 | 70 | 16 |
| 3573 | 35.53 | -3.36 | GBIF | 10.56 | 68.56 | 20.00 | 17.65 | 234 | 5 | 90.48 | 468 | 28 | 11 |
| 3574 | 36.95 | -3.34 | GBIF | 10.78 | 66.56 | 20.33 | 17.55 | 336 | 21 | 96.29 | 252 | 107 | 7 |
| 3575 | 35.40 | -3.36 | GBIF | 10.15 | 71.48 | 19.53 | 16.37 | 166 | 0 | 92.43 | 429 | 3 | 6 |
| 3576 | 35.76 | -3.55 | GBIF | 10.18 | 68.34 | 23.33 | 19.95 | 128 | 1 | 91.29 | 290 | 8 | 7 |
| 3577 | 37.65 | -8.70 | GBIF | 8.08 | 61.70 | 25.55 | 22.57 | 233 | 2 | 94.55 | 449 | 14 | 3 |
| 3578 | 34.93 | -2.30 | GBIF | 12.22 | 77.37 | 21.52 | 19.40 | 126 | 19 | 54.74 | 293 | 79 | 6 |
| 3579 | 35.65 | -3.43 | GBIF | 11.47 | 70.78 | 21.07 | 18.67 | 194 | 2 | 91.59 | 403 | 15 | 10 |
| 3580 | 34.79 | -1.70 | GBIF | 11.85 | 80.07 | 20.45 | 18.95 | 187 | 28 | 45.69 | 323 | 136 | 11 |
| 3581 | 34.81 | -2.46 | GBIF | 12.14 | 76.36 | 21.02 | 18.82 | 129 | 14 | 60.64 | 311 | 62 | 13 |
| 3582 | 36.12 | -4.09 | GBIF | 11.52 | 71.53 | 22.57 | 19.50 | 141 | 1 | 91.42 | 240 | 8 | 7 |
| 3583 | 35.52 | -3.11 | GBIF | 9.99 | 67.51 | 17.50 | 14.78 | 203 | 5 | 84.74 | 441 | 23 | 4 |
| 3584 | 35.77 | -3.91 | GBIF | 11.58 | 71.50 | 23.67 | 20.52 | 138 | 2 | 87.06 | 325 | 16 | 13 |
| 3585 | 34.73 | -2.43 | GBIF | 11.58 | 76.71 | 21.08 | 19.00 | 135 | 15 | 59.20 | 312 | 66 | 5 |
| 3586 | 36.14 | -4.00 | GBIF | 11.35 | 71.38 | 22.53 | 19.33 | 147 | 1 | 93.11 | 343 | 8 | 7 |
| 3587 | 36.08 | -4.18 | GBIF | 11.58 | 71.06 | 22.88 | 19.93 | 141 | 1 | 91.46 | 242 | 9 | 7 |
| 3588 | 34.88 | -2.23 | GBIF | 12.12 | 77.67 | 21.28 | 19.28 | 139 | 19 | 54.19 | 303 | 82 | 5 |
| 3589 | 36.06 | -4.18 | GBIF | 11.58 | 71.06 | 23.08 | 20.05 | 140 | 1 | 90.43 | 240 | 10 | 7 |
| 3590 | 35.78 | -3.83 | GBIF | 11.91 | 71.31 | 23.63 | 20.40 | 134 | 1 | 88.66 | 314 | 12 | 7 |
| 3591 | 36.05 | -4.15 | GBIF | 11.53 | 71.19 | 23.02 | 19.97 | 138 | 1 | 91.40 | 237 | 10 | 6 |
| 3592 | 35.83 | -3.95 | GBIF | 11.53 | 71.64 | 23.72 | 20.63 | 143 | 2 | 87.96 | 331 | 15 | 12 |
| 3593 | 35.92 | -4.10 | GBIF | 11.25 | 70.75 | 22.57 | 19.53 | 141 | 1 | 90.86 | 347 | 10 | 13 |
| 3594 | 34.65 | -1.90 | GBIF | 11.59 | 79.39 | 20.57 | 19.10 | 179 | 23 | 48.73 | 322 | 114 | 8 |
| 3595 | 35.71 | -3.50 | GBIF | 10.82 | 69.39 | 21.53 | 18.38 | 175 | 1 | 93.11 | 378 | 10 | 13 |
| 3596 | 34.74 | -1.85 | GBIF | 11.72 | 79.71 | 20.38 | 18.90 | 179 | 25 | 47.41 | 324 | 120 | 5 |
| 3597 | 35.89 | -3.95 | GBIF | 10.84 | 70.86 | 23.25 | 20.15 | 148 | 1 | 90.43 | 338 | 12 | 7 |
| 3598 | 35.43 | -3.02 | GBIF | 13.17 | 75.67 | 22.45 | 19.43 | 89 | 2 | 86.86 | 252 | 6 | 13 |
| 3599 | 35.48 | -3.27 | GBIF | 7.57 | 60.60 | 13.30 | 10.48 | 244 | 13 | 74.72 | 508 | 53 | 3 |

| 3600 | 35.12 | -3.15 | GBIF | 12.57 | 73.92 | 19.98 | 16.95 | 159 | 1 | 89.19 | 371 | 6 | 3 |
| --- | --- | --- | --- | --- | --- | --- | --- | --- | --- | --- | --- | --- | --- |
| 3601 | 35.41 | -3.31 | GBIF | 10.14 | 69.94 | 19.17 | 16.07 | 172 | 0 | 91.20 | 437 | 3 | 3 |
| 3602 | 35.18 | -3.07 | GBIF | 13.13 | 75.00 | 20.40 | 17.38 | 133 | 2 | 87.83 | 323 | 9 | 5 |
| 3603 | 35.58 | -3.25 | GBIF | 8.88 | 64.31 | 18.07 | 15.60 | 201 | 3 | 87.17 | 433 | 20 | 14 |
| 3604 | 35.51 | -3.09 | GBIF | 9.92 | 68.39 | 18.15 | 15.50 | 177 | 1 | 89.73 | 415 | 7 | 4 |
| 3605 | 35.77 | -3.44 | GBIF | 10.60 | 69.74 | 22.27 | 19.20 | 161 | 1 | 92.42 | 344 | 9 | 7 |
| 3606 | 36.11 | -3.94 | GBIF | 11.20 | 70.44 | 22.48 | 19.28 | 152 | 1 | 92.89 | 347 | 8 | 8 |
| 3607 | 34.96 | -6.34 | GBIF | 11.23 | 67.22 | 23.12 | 19.73 | 130 | 0 | 112.60 | 284 | 0 | 4 |
| 3608 | 36.93 | -2.92 | GBIF | 12.23 | 71.96 | 22.10 | 19.28 | 135 | 1 | 87.28 | 174 | 8 | 6 |
| 3609 | 36.02 | -3.93 | GBIF | 10.98 | 71.27 | 22.15 | 19.08 | 149 | 1 | 91.92 | 351 | 7 | 8 |
| 3610 | 36.13 | -3.79 | GBIF | 11.13 | 70.41 | 22.57 | 19.35 | 162 | 1 | 93.12 | 352 | 9 | 4 |
| 3611 | 36.19 | -3.79 | GBIF | 11.44 | 71.07 | 22.20 | 19.05 | 171 | 1 | 94.85 | 267 | 8 | 4 |
| 3612 | 35.62 | -3.48 | GBIF | 11.34 | 71.33 | 21.68 | 18.62 | 165 | 1 | 90.89 | 370 | 9 | 10 |
| 3613 | 35.48 | -3.30 | GBIF | 7.51 | 60.07 | 13.48 | 11.13 | 267 | 13 | 77.53 | 536 | 52 | 3 |
| 3614 | 35.44 | -3.34 | GBIF | 8.26 | 64.02 | 15.37 | 12.62 | 234 | 8 | 79.71 | 493 | 36 | 5 |
| 3615 | 34.95 | -2.78 | GBIF | 12.19 | 74.80 | 20.07 | 17.32 | 133 | 6 | 74.57 | 326 | 28 | 8 |
| 3616 | 35.95 | -3.48 | GBIF | 11.66 | 71.52 | 23.58 | 20.50 | 153 | 0 | 93.51 | 320 | 8 | 6 |
| 3617 | 35.67 | -3.56 | GBIF | 10.42 | 69.04 | 21.18 | 18.03 | 171 | 1 | 91.95 | 380 | 9 | 6 |
| 3618 | 35.60 | -3.46 | GBIF | 11.47 | 71.22 | 22.02 | 18.92 | 162 | 1 | 89.52 | 362 | 12 | 16 |
| 3619 | 37.03 | -7.38 | GBIF | 9.53 | 61.06 | 25.48 | 21.58 | 272 | 8 | 85.66 | 432 | 38 | 14 |
| 3620 | 34.82 | -2.49 | GBIF | 12.21 | 76.30 | 20.95 | 18.72 | 129 | 13 | 62.14 | 310 | 57 | 13 |
| 3621 | 34.80 | -7.73 | GBIF | 11.96 | 67.56 | 23.37 | 20.32 | 139 | 0 | 107.45 | 159 | 0 | 6 |
| 3622 | 35.97 | -3.67 | GBIF | 12.08 | 70.66 | 23.58 | 20.40 | 146 | 1 | 90.90 | 321 | 10 | 10 |
| 3623 | 34.78 | -2.39 | GBIF | 11.82 | 77.23 | 21.02 | 18.95 | 137 | 16 | 58.14 | 311 | 71 | 5 |
| 3624 | 34.85 | -7.78 | GBIF | 11.66 | 67.39 | 23.63 | 20.72 | 141 | 0 | 106.05 | 162 | 0 | 4 |
| 3625 | 35.07 | -3.01 | GBIF | 12.60 | 74.12 | 20.40 | 17.38 | 136 | 3 | 84.66 | 326 | 13 | 7 |
| 3626 | 34.83 | -2.44 | GBIF | 12.13 | 76.26 | 21.13 | 18.95 | 133 | 15 | 60.13 | 307 | 65 | 7 |
| 3627 | 36.04 | -3.79 | GBIF | 11.58 | 71.06 | 23.10 | 19.97 | 145 | 1 | 90.88 | 326 | 9 | 8 |
| 3628 | 36.13 | -3.91 | GBIF | 11.03 | 70.67 | 22.03 | 18.85 | 167 | 1 | 94.43 | 367 | 7 | 7 |
| 3629 | 34.96 | -2.44 | GBIF | 11.93 | 76.50 | 20.83 | 18.48 | 126 | 15 | 62.03 | 307 | 59 | 4 |
| 3630 | 35.59 | -3.38 | GBIF | 11.42 | 70.04 | 20.70 | 18.35 | 199 | 3 | 89.85 | 411 | 21 | 16 |
| 3631 | 36.15 | -3.77 | GBIF | 11.21 | 70.49 | 22.40 | 19.15 | 170 | 1 | 94.04 | 363 | 9 | 4 |
| 3632 | 35.96 | -3.51 | GBIF | 11.69 | 71.29 | 23.60 | 20.48 | 149 | 1 | 92.93 | 319 | 9 | 4 |
| 3633 | 35.66 | -3.48 | GBIF | 11.26 | 70.36 | 21.53 | 18.43 | 179 | 1 | 93.09 | 387 | 9 | 16 |
| 3634 | 36.04 | -3.69 | GBIF | 11.98 | 71.71 | 23.30 | 20.05 | 157 | 1 | 92.45 | 335 | 10 | 7 |
| 3635 | 37.77 | -3.99 | GBIF | 10.62 | 67.62 | 21.95 | 17.93 | 156 | 7 | 77.04 | 310 | 30 | 6 |
| 3636 | 36.83 | -7.84 | GBIF | 7.55 | 62.40 | 20.97 | 18.23 | 305 | 4 | 88.59 | 528 | 49 | 4 |
| 3637 | 35.49 | -3.04 | GBIF | 11.24 | 71.60 | 21.52 | 18.50 | 101 | 1 | 87.46 | 270 | 5 | 4 |
| 3638 | 34.99 | -2.46 | GBIF | 12.00 | 76.43 | 20.57 | 18.13 | 125 | 14 | 63.53 | 343 | 54 | 4 |
| 3639 | 34.86 | -2.58 | GBIF | 11.97 | 75.31 | 20.42 | 18.00 | 132 | 11 | 65.76 | 324 | 49 | 7 |
| 3640 | 36.98 | -3.20 | GBIF | 11.27 | 67.51 | 20.22 | 17.48 | 300 | 15 | 95.64 | 223 | 106 | 8 |
| 3641 | 34.91 | -2.56 | GBIF | 12.02 | 75.58 | 20.42 | 17.95 | 129 | 11 | 65.83 | 318 | 47 | 7 |
| 3642 | 35.45 | -3.08 | GBIF | 12.23 | 74.09 | 21.48 | 18.50 | 92 | 1 | 87.86 | 260 | 5 | 5 |
| 3643 | 34.68 | -2.27 | GBIF | 12.03 | 78.65 | 22.18 | 20.42 | 135 | 17 | 53.92 | 288 | 82 | 9 |
| 3644 | 34.88 | -7.73 | GBIF | 11.78 | 68.51 | 24.00 | 21.02 | 136 | 0 | 106.27 | 158 | 0 | 12 |
| 3645 | 35.79 | -3.57 | GBIF | 9.98 | 67.86 | 23.43 | 19.93 | 109 | 1 | 91.76 | 273 | 6 | 6 |
| 3646 | 38.06 | -7.64 | GBIF | 8.86 | 67.62 | 28.22 | 25.07 | 227 | 7 | 79.60 | 415 | 33 | 4 |
| 3647 | 34.88 | -3.05 | GBIF | 12.45 | 73.67 | 20.30 | 17.68 | 149 | 3 | 84.04 | 351 | 13 | 6 |
| 3648 | 35.87 | -3.84 | GBIF | 11.79 | 71.90 | 23.67 | 20.50 | 118 | 1 | 90.27 | 295 | 8 | 15 |
| 3649 | 37.10 | -7.13 | GBIF | 10.10 | 63.52 | 26.00 | 22.80 | 265 | 10 | 85.39 | 390 | 41 | 5 |

| 3650 | 34.81 | -2.86 | GBIF | 12.21 | 74.44 | 20.27 | 17.68 | 143 | 6 | 75.84 | 344 | 25 | 6 |
| --- | --- | --- | --- | --- | --- | --- | --- | --- | --- | --- | --- | --- | --- |
| 3651 | 37.03 | -7.25 | GBIF | 8.82 | 61.71 | 24.85 | 21.43 | 265 | 7 | 85.35 | 407 | 40 | 6 |
| 3652 | 34.83 | -2.57 | GBIF | 11.98 | 74.90 | 20.47 | 18.10 | 132 | 11 | 65.28 | 321 | 50 | 8 |
| 3653 | 34.83 | -2.70 | GBIF | 12.28 | 74.90 | 20.60 | 18.17 | 133 | 8 | 70.45 | 324 | 37 | 7 |
| 3654 | 35.07 | -2.16 | GBIF | 12.21 | 78.76 | 20.42 | 18.32 | 142 | 17 | 59.49 | 358 | 67 | 4 |
| 3655 | 34.45 | -2.33 | GBIF | 12.17 | 78.49 | 22.12 | 20.67 | 143 | 11 | 58.70 | 297 | 67 | 6 |
| 3656 | 35.95 | -3.43 | GBIF | 11.92 | 71.84 | 23.62 | 20.57 | 146 | 1 | 92.88 | 220 | 8 | 9 |
| 3657 | 35.08 | -2.05 | GBIF | 12.14 | 78.84 | 20.50 | 18.52 | 148 | 18 | 57.08 | 362 | 75 | 4 |
| 3658 | 36.02 | -3.58 | GBIF | 11.84 | 71.34 | 23.35 | 20.22 | 151 | 1 | 91.73 | 230 | 10 | 6 |
| 3659 | 35.81 | -3.69 | GBIF | 10.02 | 66.78 | 23.53 | 19.83 | 138 | 2 | 87.10 | 319 | 13 | 6 |
| 3660 | 34.88 | -2.99 | GBIF | 12.49 | 73.92 | 20.33 | 17.62 | 146 | 4 | 81.44 | 347 | 17 | 6 |
| 3661 | 37.16 | -7.28 | GBIF | 9.48 | 61.98 | 25.53 | 21.55 | 275 | 7 | 85.30 | 411 | 38 | 7 |
| 3662 | 35.84 | -4.02 | GBIF | 10.58 | 71.51 | 22.30 | 19.30 | 153 | 1 | 90.65 | 366 | 10 | 7 |
| 3663 | 35.83 | -4.07 | GBIF | 10.86 | 72.39 | 21.75 | 18.88 | 144 | 0 | 91.91 | 366 | 7 | 8 |
| 3664 | 34.85 | -3.14 | GBIF | 12.01 | 73.22 | 20.10 | 17.40 | 157 | 2 | 86.01 | 389 | 11 | 5 |
| 3665 | 35.84 | -4.15 | GBIF | 11.35 | 72.29 | 22.52 | 19.53 | 130 | 0 | 91.38 | 265 | 7 | 9 |
| 3666 | 35.71 | -3.55 | GBIF | 10.07 | 68.48 | 21.83 | 18.62 | 177 | 1 | 94.07 | 375 | 8 | 7 |
| 3667 | 35.94 | -3.51 | GBIF | 11.41 | 70.42 | 23.57 | 20.37 | 151 | 1 | 92.29 | 318 | 11 | 4 |
| 3668 | 35.97 | -3.60 | GBIF | 11.87 | 71.06 | 23.53 | 20.38 | 154 | 1 | 91.14 | 325 | 11 | 11 |
| 3669 | 34.81 | -3.10 | GBIF | 11.83 | 73.05 | 20.02 | 17.42 | 157 | 3 | 84.17 | 239 | 14 | 6 |
| 3670 | 34.98 | -2.91 | GBIF | 12.34 | 74.35 | 20.13 | 17.22 | 138 | 4 | 81.40 | 332 | 17 | 8 |
| 3671 | 35.96 | -3.63 | GBIF | 12.06 | 70.93 | 23.63 | 20.45 | 154 | 2 | 89.61 | 329 | 13 | 8 |
| 3672 | 34.21 | -2.36 | GBIF | 12.42 | 79.09 | 22.60 | 21.75 | 146 | 7 | 63.56 | 168 | 55 | 7 |
| 3673 | 34.85 | -2.65 | GBIF | 12.15 | 75.47 | 20.40 | 18.00 | 131 | 9 | 68.77 | 322 | 41 | 11 |
| 3674 | 35.95 | -3.72 | GBIF | 12.03 | 70.78 | 23.53 | 20.30 | 163 | 2 | 88.98 | 357 | 15 | 8 |
| 3675 | 35.68 | -3.67 | GBIF | 8.93 | 65.21 | 18.57 | 15.88 | 235 | 4 | 92.07 | 479 | 21 | 6 |
| 3676 | 35.82 | -3.56 | GBIF | 9.23 | 65.95 | 23.42 | 19.77 | 135 | 1 | 88.27 | 308 | 11 | 0 |
| 3677 | 35.81 | -3.92 | GBIF | 11.73 | 71.49 | 23.72 | 20.55 | 135 | 2 | 86.37 | 321 | 16 | 16 |
| 3678 | 35.87 | -4.00 | GBIF | 10.18 | 70.72 | 22.33 | 19.28 | 152 | 1 | 90.72 | 359 | 11 | 7 |
| 3679 | 35.91 | -4.16 | GBIF | 11.81 | 72.00 | 22.70 | 19.72 | 134 | 0 | 91.25 | 261 | 8 | 8 |
| 3680 | 35.97 | -4.10 | GBIF | 11.23 | 70.60 | 22.67 | 19.63 | 142 | 1 | 91.75 | 244 | 9 | 7 |
| 3681 | 35.95 | -4.12 | GBIF | 11.36 | 70.99 | 22.45 | 19.52 | 144 | 1 | 90.93 | 256 | 9 | 7 |
| 3682 | 35.91 | -4.02 | GBIF | 10.42 | 70.38 | 22.58 | 19.48 | 140 | 1 | 89.39 | 341 | 10 | 6 |
| 3683 | 35.96 | -4.17 | GBIF | 11.58 | 71.50 | 22.87 | 19.87 | 139 | 1 | 89.61 | 254 | 11 | 11 |
| 3684 | 35.87 | -4.11 | GBIF | 11.27 | 71.36 | 22.75 | 19.70 | 142 | 0 | 91.20 | 350 | 9 | 8 |
| 3685 | 35.91 | -4.07 | GBIF | 10.84 | 70.86 | 22.62 | 19.55 | 141 | 1 | 90.01 | 346 | 11 | 7 |
| 3686 | 35.44 | -3.16 | GBIF | 10.42 | 69.44 | 16.88 | 14.27 | 185 | 2 | 84.35 | 425 | 14 | 5 |
| 3687 | 35.42 | -3.06 | GBIF | 13.31 | 76.05 | 22.33 | 19.30 | 91 | 1 | 88.03 | 251 | 5 | 6 |
| 3688 | 35.58 | -3.18 | GBIF | 11.21 | 70.49 | 19.67 | 16.80 | 176 | 2 | 89.26 | 394 | 12 | 4 |
| 3689 | 35.75 | -3.30 | GBIF | 10.78 | 67.82 | 19.92 | 17.55 | 229 | 4 | 94.06 | 295 | 22 | 10 |
| 3690 | 35.92 | -3.80 | GBIF | 11.29 | 70.57 | 23.35 | 20.15 | 156 | 2 | 87.59 | 346 | 15 | 13 |
| 3691 | 35.56 | -3.24 | GBIF | 9.94 | 67.17 | 19.45 | 16.43 | 180 | 2 | 88.33 | 401 | 14 | 3 |
| 3692 | 37.88 | -3.88 | GBIF | 10.93 | 68.33 | 24.52 | 20.58 | 122 | 7 | 76.56 | 232 | 28 | 7 |
| 3693 | 35.97 | -3.85 | GBIF | 11.05 | 70.38 | 23.00 | 19.87 | 152 | 1 | 91.27 | 339 | 10 | 7 |
| 3694 | 34.93 | -2.46 | GBIF | 12.12 | 76.69 | 20.83 | 18.52 | 134 | 15 | 60.91 | 315 | 63 | 4 |
| 3695 | 35.47 | -3.23 | GBIF | 8.53 | 63.15 | 14.23 | 11.58 | 213 | 10 | 75.54 | 454 | 43 | 7 |
| 3696 | 35.81 | -4.17 | GBIF | 11.07 | 72.33 | 21.93 | 18.98 | 132 | 0 | 92.96 | 339 | 6 | 8 |
| 3697 | 38.08 | -7.63 | GBIF | 8.84 | 66.98 | 28.32 | 25.07 | 227 | 7 | 79.86 | 408 | 32 | 5 |
| 3698 | 35.81 | -4.03 | GBIF | 11.27 | 72.22 | 23.00 | 20.08 | 146 | 1 | 89.65 | 350 | 11 | 10 |
| 3699 | 35.92 | -4.15 | GBIF | 11.72 | 71.93 | 22.70 | 19.73 | 141 | 1 | 90.43 | 264 | 10 | 8 |

| 3700 | 34.84 | -2.90 | GBIF | 12.38 | 74.10 | 20.20 | 17.57 | 143 | 5 | 78.58 | 344 | 20 | 7 |
| --- | --- | --- | --- | --- | --- | --- | --- | --- | --- | --- | --- | --- | --- |
| 3701 | 35.97 | -4.03 | GBIF | 10.87 | 71.49 | 22.15 | 19.20 | 146 | 0 | 91.46 | 352 | 7 | 7 |
| 3702 | 35.96 | -4.13 | GBIF | 11.41 | 71.30 | 22.72 | 19.78 | 140 | 1 | 89.77 | 249 | 11 | 7 |
| 3703 | 35.82 | -4.07 | GBIF | 11.03 | 72.59 | 22.70 | 19.82 | 137 | 0 | 91.72 | 347 | 7 | 8 |
| 3704 | 35.87 | -4.05 | GBIF | 10.35 | 70.89 | 22.15 | 19.07 | 145 | 1 | 90.39 | 358 | 11 | 7 |
| 3705 | 35.92 | -4.18 | GBIF | 11.82 | 71.67 | 22.65 | 19.62 | 137 | 0 | 91.05 | 265 | 8 | 8 |
| 3706 | 35.96 | -4.19 | GBIF | 11.77 | 71.80 | 22.95 | 19.93 | 145 | 1 | 87.66 | 268 | 13 | 12 |
| 3707 | 35.93 | -4.08 | GBIF | 10.98 | 70.81 | 22.48 | 19.50 | 143 | 1 | 91.48 | 350 | 9 | 7 |
| 3708 | 35.80 | -4.19 | GBIF | 10.94 | 71.98 | 21.25 | 18.30 | 138 | 0 | 92.90 | 351 | 5 | 8 |
| 3709 | 35.87 | -4.10 | GBIF | 11.03 | 71.18 | 22.65 | 19.58 | 145 | 1 | 90.82 | 353 | 11 | 8 |
| 3710 | 37.00 | -3.24 | GBIF | 11.00 | 66.67 | 20.52 | 17.77 | 355 | 20 | 99.73 | 237 | 124 | 7 |
| 3711 | 35.69 | -3.56 | GBIF | 9.94 | 68.56 | 20.90 | 17.77 | 185 | 1 | 93.50 | 398 | 9 | 6 |
| 3712 | 35.82 | -3.78 | GBIF | 12.15 | 71.89 | 23.67 | 20.50 | 147 | 2 | 88.48 | 336 | 13 | 7 |
| 3713 | 35.94 | -3.78 | GBIF | 11.52 | 70.71 | 23.32 | 20.18 | 161 | 2 | 88.02 | 355 | 15 | 8 |
| 3714 | 35.61 | -3.03 | GBIF | 8.63 | 64.43 | 12.87 | 9.92 | 244 | 6 | 79.35 | 509 | 27 | 10 |
| 3715 | 35.78 | -3.08 | GBIF | 9.55 | 67.73 | 12.32 | 9.28 | 248 | 9 | 76.10 | 523 | 42 | 4 |
| 3716 | 36.14 | -3.75 | GBIF | 11.25 | 70.31 | 22.57 | 19.28 | 166 | 1 | 93.45 | 354 | 10 | 4 |
| 3717 | 35.79 | -3.56 | GBIF | 9.77 | 67.41 | 23.42 | 19.88 | 111 | 1 | 91.47 | 275 | 7 | 0 |
| 3718 | 37.15 | -7.38 | GBIF | 8.54 | 60.58 | 24.57 | 21.07 | 288 | 5 | 88.05 | 434 | 34 | 7 |
| 3719 | 34.72 | -2.55 | GBIF | 11.95 | 77.10 | 20.92 | 18.80 | 134 | 11 | 63.81 | 320 | 51 | 7 |
| 3720 | 34.82 | -2.41 | GBIF | 11.98 | 76.82 | 21.37 | 19.20 | 131 | 17 | 58.14 | 302 | 70 | 7 |
| 3721 | 35.89 | -4.10 | GBIF | 11.28 | 71.41 | 22.73 | 19.70 | 148 | 1 | 91.04 | 357 | 10 | 13 |
| 3722 | 35.83 | -3.64 | GBIF | 9.51 | 66.49 | 23.45 | 19.77 | 135 | 1 | 88.50 | 312 | 11 | 0 |
| 3723 | 36.00 | -4.18 | GBIF | 11.29 | 71.47 | 22.57 | 19.53 | 140 | 1 | 90.15 | 260 | 9 | 9 |
| 3724 | 35.88 | -4.13 | GBIF | 11.46 | 71.61 | 22.82 | 19.77 | 135 | 0 | 90.37 | 257 | 8 | 9 |
| 3725 | 35.91 | -3.76 | GBIF | 11.57 | 71.01 | 23.48 | 20.32 | 166 | 3 | 87.39 | 361 | 17 | 15 |
| 3726 | 36.11 | -3.66 | GBIF | 11.85 | 71.39 | 22.93 | 19.72 | 156 | 1 | 91.63 | 337 | 10 | 5 |
| 3727 | 35.79 | -2.88 | GBIF | 9.80 | 71.01 | 11.77 | 9.22 | 235 | 10 | 75.70 | 500 | 40 | 4 |
| 3728 | 36.88 | -3.36 | GBIF | 10.84 | 66.51 | 21.07 | 18.22 | 289 | 18 | 93.74 | 247 | 74 | 19 |
| 3729 | 35.71 | -3.58 | GBIF | 9.38 | 67.02 | 19.90 | 16.75 | 218 | 2 | 94.25 | 451 | 11 | 6 |
| 3730 | 34.13 | -1.81 | GBIF | 11.55 | 81.91 | 21.63 | 20.58 | 178 | 17 | 52.22 | 297 | 93 | 10 |
| 3731 | 35.24 | -1.98 | GBIF | 11.82 | 78.78 | 19.15 | 16.97 | 157 | 18 | 60.79 | 386 | 68 | 5 |
| 3732 | 34.86 | -2.44 | GBIF | 12.08 | 76.48 | 21.00 | 18.77 | 132 | 14 | 60.93 | 310 | 63 | 13 |
| 3733 | 35.10 | -2.13 | GBIF | 12.27 | 79.65 | 20.42 | 18.32 | 139 | 16 | 59.25 | 354 | 67 | 4 |
| 3734 | 34.81 | -2.33 | GBIF | 12.02 | 77.58 | 21.55 | 19.52 | 135 | 18 | 55.38 | 299 | 79 | 4 |
| 3735 | 35.48 | -3.25 | GBIF | 7.68 | 60.43 | 13.23 | 10.98 | 237 | 13 | 73.75 | 488 | 56 | 4 |
| 3736 | 34.67 | -2.58 | GBIF | 11.85 | 76.45 | 21.02 | 19.00 | 135 | 11 | 65.50 | 316 | 49 | 8 |
| 3737 | 34.21 | -2.33 | GBIF | 12.42 | 78.59 | 22.70 | 21.83 | 143 | 8 | 61.93 | 169 | 59 | 7 |
| 3738 | 35.00 | -2.50 | GBIF | 12.17 | 76.04 | 20.42 | 17.97 | 126 | 12 | 64.75 | 314 | 50 | 5 |
| 3739 | 34.03 | -2.34 | GBIF | 12.11 | 78.63 | 22.72 | 21.82 | 144 | 6 | 63.98 | 176 | 55 | 8 |
| 3740 | 34.43 | -2.21 | GBIF | 12.20 | 80.79 | 22.67 | 21.17 | 145 | 12 | 56.97 | 288 | 72 | 5 |
| 3741 | 34.21 | -2.14 | GBIF | 12.10 | 80.67 | 22.95 | 22.05 | 146 | 9 | 58.31 | 283 | 68 | 6 |
| 3742 | 34.75 | -2.37 | GBIF | 11.67 | 77.26 | 21.02 | 19.00 | 141 | 16 | 58.46 | 313 | 70 | 5 |
| 3743 | 34.65 | -2.32 | GBIF | 12.30 | 78.34 | 22.25 | 20.52 | 129 | 15 | 54.52 | 291 | 77 | 7 |
| 3744 | 33.87 | -2.20 | GBIF | 11.72 | 80.25 | 23.22 | 22.42 | 143 | 5 | 64.05 | 179 | 58 | 16 |
| 3745 | 35.74 | -3.00 | GBIF | 11.69 | 71.73 | 15.50 | 12.75 | 192 | 3 | 82.66 | 422 | 15 | 6 |
| 3746 | 34.80 | -2.29 | GBIF | 12.12 | 77.67 | 21.88 | 19.90 | 130 | 18 | 53.54 | 292 | 83 | 5 |
| 3747 | 35.59 | -3.10 | GBIF | 9.90 | 67.81 | 18.57 | 15.68 | 200 | 3 | 87.65 | 429 | 18 | 10 |
| 3748 | 36.09 | -3.83 | GBIF | 11.27 | 70.86 | 22.75 | 19.58 | 156 | 1 | 92.47 | 342 | 9 | 6 |
| 3749 | 36.03 | -3.48 | GBIF | 11.37 | 71.49 | 22.93 | 19.82 | 184 | 2 | 92.11 | 260 | 13 | 4 |

| 3750 | 35.83 | -4.17 | GBIF | 11.13 | 72.24 | 22.23 | 19.30 | 130 | 0 | 91.73 | 273 | 6 | 9 |
| --- | --- | --- | --- | --- | --- | --- | --- | --- | --- | --- | --- | --- | --- |
| 3751 | 35.07 | -1.82 | GBIF | 12.21 | 80.32 | 19.90 | 18.40 | 166 | 24 | 51.79 | 387 | 102 | 5 |
| 3752 | 35.87 | -4.19 | GBIF | 11.22 | 71.44 | 22.42 | 19.40 | 136 | 0 | 91.45 | 269 | 8 | 8 |
| 3753 | 35.94 | -4.11 | GBIF | 11.38 | 71.09 | 22.43 | 19.52 | 141 | 0 | 91.09 | 255 | 8 | 7 |
| 3754 | 37.17 | -7.22 | GBIF | 9.73 | 63.20 | 25.62 | 22.30 | 266 | 6 | 85.87 | 393 | 37 | 5 |
| 3755 | 35.82 | -4.13 | GBIF | 11.06 | 72.75 | 22.08 | 19.20 | 134 | 0 | 91.98 | 351 | 6 | 9 |
| 3756 | 35.90 | -4.02 | GBIF | 10.39 | 70.69 | 22.75 | 19.62 | 141 | 1 | 89.28 | 339 | 11 | 7 |
| 3757 | 35.98 | -4.17 | GBIF | 11.40 | 71.25 | 22.70 | 19.70 | 140 | 1 | 90.40 | 252 | 10 | 15 |
| 3758 | 35.96 | -4.02 | GBIF | 10.76 | 71.25 | 22.30 | 19.35 | 147 | 0 | 91.23 | 352 | 8 | 6 |
| 3759 | 35.90 | -4.09 | GBIF | 11.24 | 70.70 | 22.67 | 19.62 | 143 | 1 | 90.31 | 350 | 11 | 8 |
| 3760 | 36.18 | -3.85 | GBIF | 11.38 | 71.54 | 22.08 | 19.00 | 165 | 1 | 94.06 | 272 | 8 | 6 |
| 3761 | 35.64 | -3.04 | GBIF | 8.98 | 66.05 | 11.83 | 8.88 | 245 | 9 | 76.62 | 520 | 38 | 5 |
| 3762 | 36.08 | -3.86 | GBIF | 11.27 | 70.91 | 22.85 | 19.70 | 152 | 1 | 92.50 | 340 | 9 | 8 |
| 3763 | 38.77 | -6.11 | GBIF | 9.34 | 68.69 | 27.90 | 25.72 | 167 | 26 | 54.90 | 242 | 99 | 5 |
| 3764 | 35.84 | -4.11 | GBIF | 11.16 | 71.99 | 22.50 | 19.57 | 134 | 0 | 91.06 | 346 | 8 | 9 |
| 3765 | 35.87 | -4.14 | GBIF | 11.48 | 71.77 | 22.75 | 19.72 | 133 | 0 | 90.73 | 261 | 8 | 9 |
| 3766 | 35.96 | -4.10 | GBIF | 11.24 | 70.70 | 22.53 | 19.55 | 144 | 1 | 92.08 | 249 | 8 | 7 |
| 3767 | 35.93 | -4.19 | GBIF | 11.85 | 71.82 | 22.65 | 19.62 | 135 | 0 | 90.80 | 266 | 8 | 10 |
| 3768 | 36.09 | -4.17 | GBIF | 11.57 | 71.01 | 22.68 | 19.75 | 140 | 1 | 91.44 | 244 | 8 | 7 |
| 3769 | 35.86 | -3.51 | GBIF | 9.22 | 65.83 | 23.35 | 19.70 | 143 | 2 | 87.07 | 318 | 13 | 0 |
| 3770 | 36.12 | -4.19 | GBIF | 11.73 | 71.54 | 22.83 | 19.83 | 139 | 1 | 91.39 | 241 | 8 | 7 |
| 3771 | 36.11 | -4.11 | GBIF | 11.55 | 71.74 | 22.65 | 19.65 | 142 | 1 | 91.52 | 239 | 9 | 7 |
| 3772 | 36.16 | -4.10 | GBIF | 11.60 | 71.60 | 22.63 | 19.62 | 142 | 1 | 92.22 | 239 | 7 | 7 |
| 3773 | 36.13 | -4.19 | GBIF | 11.77 | 71.31 | 22.82 | 19.82 | 140 | 1 | 91.28 | 238 | 8 | 7 |
| 3774 | 36.20 | -3.81 | GBIF | 11.43 | 71.41 | 22.13 | 19.03 | 167 | 1 | 94.24 | 270 | 8 | 5 |
| 3775 | 35.82 | -3.79 | GBIF | 12.27 | 71.73 | 23.72 | 20.53 | 134 | 1 | 90.20 | 313 | 9 | 7 |
| 3776 | 35.94 | -3.68 | GBIF | 12.04 | 70.83 | 23.52 | 20.28 | 148 | 1 | 91.26 | 326 | 10 | 11 |
| 3777 | 35.00 | -1.53 | GBIF | 12.52 | 81.28 | 20.63 | 19.18 | 170 | 29 | 44.01 | 300 | 136 | 20 |
| 3778 | 35.91 | -3.51 | GBIF | 10.77 | 69.02 | 23.47 | 20.10 | 166 | 2 | 89.10 | 349 | 15 | 4 |
| 3779 | 35.87 | -3.45 | GBIF | 9.81 | 67.18 | 23.35 | 19.83 | 145 | 2 | 88.25 | 318 | 13 | 5 |
| 3780 | 34.89 | -2.41 | GBIF | 11.93 | 76.44 | 20.98 | 18.75 | 135 | 15 | 60.15 | 308 | 66 | 6 |
| 3781 | 34.48 | -2.26 | GBIF | 12.38 | 79.89 | 22.47 | 21.02 | 138 | 12 | 56.62 | 287 | 73 | 7 |
| 3782 | 34.65 | -2.41 | GBIF | 11.54 | 77.46 | 21.32 | 19.37 | 137 | 14 | 58.56 | 312 | 68 | 5 |
| 3783 | 34.98 | -2.55 | GBIF | 12.10 | 76.10 | 20.17 | 17.68 | 128 | 11 | 67.35 | 320 | 44 | 7 |
| 3784 | 34.75 | -1.97 | GBIF | 11.76 | 79.45 | 21.55 | 19.77 | 160 | 21 | 48.84 | 300 | 106 | 7 |
| 3785 | 37.36 | -7.27 | GBIF | 8.22 | 61.38 | 25.03 | 21.57 | 307 | 8 | 83.86 | 438 | 52 | 8 |
| 3786 | 34.81 | -6.24 | GBIF | 12.06 | 68.13 | 23.32 | 20.48 | 133 | 0 | 111.25 | 291 | 1 | 7 |
| 3787 | 34.40 | -8.35 | GBIF | 12.55 | 68.21 | 23.23 | 19.97 | 153 | 0 | 111.65 | 202 | 0 | 6 |
| 3788 | 37.09 | -7.42 | GBIF | 8.40 | 60.43 | 24.48 | 21.13 | 276 | 5 | 85.98 | 441 | 38 | 7 |
| 3789 | 34.91 | -2.51 | GBIF | 12.10 | 76.58 | 20.52 | 18.20 | 134 | 14 | 61.93 | 327 | 59 | 6 |
| 3790 | 34.68 | -2.55 | GBIF | 12.01 | 76.49 | 21.18 | 19.17 | 139 | 13 | 61.59 | 320 | 60 | 7 |
| 3791 | 35.52 | -3.16 | GBIF | 12.03 | 70.37 | 19.68 | 16.82 | 155 | 3 | 85.87 | 372 | 15 | 4 |
| 3792 | 35.48 | -3.07 | GBIF | 11.29 | 71.92 | 21.05 | 18.02 | 96 | 1 | 88.50 | 273 | 5 | 4 |
| 3793 | 35.92 | -3.55 | GBIF | 11.43 | 70.09 | 23.55 | 20.32 | 158 | 2 | 90.15 | 331 | 14 | 4 |
| 3794 | 35.99 | -3.50 | GBIF | 11.73 | 71.93 | 23.42 | 20.40 | 166 | 1 | 93.24 | 244 | 10 | 8 |
| 3795 | 34.89 | -6.25 | GBIF | 11.82 | 68.35 | 23.37 | 20.52 | 130 | 0 | 111.82 | 286 | 1 | 3 |
| 3796 | 34.82 | -7.66 | GBIF | 12.02 | 67.18 | 23.45 | 20.45 | 132 | 0 | 108.04 | 155 | 0 | 6 |
| 3797 | 35.86 | -3.42 | GBIF | 10.24 | 68.28 | 23.32 | 19.92 | 130 | 1 | 91.41 | 286 | 8 | 6 |
| 3798 | 35.64 | -3.55 | GBIF | 10.76 | 69.86 | 20.73 | 17.67 | 173 | 1 | 92.46 | 391 | 7 | 6 |
| 3799 | 34.94 | -2.22 | GBIF | 11.89 | 77.72 | 20.95 | 18.87 | 138 | 19 | 55.27 | 305 | 79 | 6 |

| 3800 | 35.55 | -3.19 | GBIF | 11.61 | 70.35 | 19.78 | 16.90 | 150 | 2 | 87.92 | 366 | 11 | 4 |
| --- | --- | --- | --- | --- | --- | --- | --- | --- | --- | --- | --- | --- | --- |
| 3801 | 35.88 | -4.17 | GBIF | 11.58 | 71.95 | 22.62 | 19.60 | 133 | 0 | 90.85 | 264 | 8 | 8 |
| 3802 | 37.18 | -7.41 | GBIF | 8.03 | 60.40 | 24.22 | 20.72 | 306 | 6 | 86.16 | 437 | 43 | 10 |
| 3803 | 35.44 | -3.19 | GBIF | 10.13 | 68.47 | 16.35 | 13.73 | 193 | 2 | 84.09 | 431 | 15 | 5 |
| 3804 | 35.22 | -3.11 | GBIF | 12.80 | 73.99 | 20.32 | 17.22 | 135 | 1 | 88.38 | 329 | 7 | 4 |
| 3805 | 34.96 | -2.56 | GBIF | 12.04 | 75.73 | 20.33 | 17.83 | 129 | 11 | 67.32 | 317 | 45 | 7 |
| 3806 | 37.17 | -7.28 | GBIF | 9.40 | 61.84 | 25.45 | 21.47 | 278 | 7 | 85.59 | 412 | 38 | 8 |
| 3807 | 34.72 | -2.03 | GBIF | 12.15 | 79.93 | 21.92 | 20.23 | 154 | 19 | 50.48 | 292 | 98 | 5 |
| 3808 | 34.73 | -2.39 | GBIF | 11.73 | 77.14 | 21.40 | 19.40 | 133 | 16 | 57.15 | 306 | 71 | 5 |
| 3809 | 36.15 | -3.81 | GBIF | 11.08 | 71.05 | 22.13 | 18.98 | 166 | 1 | 94.00 | 267 | 8 | 4 |
| 3810 | 35.88 | -3.53 | GBIF | 9.73 | 67.13 | 23.38 | 19.85 | 157 | 2 | 87.13 | 341 | 16 | 0 |
| 3811 | 35.43 | -3.04 | GBIF | 13.17 | 75.67 | 22.50 | 19.45 | 89 | 2 | 86.89 | 249 | 6 | 7 |
| 3812 | 34.74 | -1.88 | GBIF | 11.73 | 79.22 | 20.43 | 18.95 | 175 | 24 | 48.03 | 320 | 115 | 6 |
| 3813 | 35.91 | -3.75 | GBIF | 11.66 | 71.09 | 23.52 | 20.35 | 154 | 2 | 89.88 | 341 | 12 | 15 |
| 3814 | 35.00 | -2.29 | GBIF | 12.16 | 77.44 | 21.17 | 19.00 | 128 | 18 | 56.94 | 297 | 72 | 6 |
| 3815 | 35.72 | -3.51 | GBIF | 10.42 | 68.53 | 21.18 | 18.02 | 190 | 1 | 94.60 | 399 | 10 | 13 |
| 3816 | 35.64 | -3.54 | GBIF | 10.94 | 69.69 | 21.05 | 17.93 | 167 | 0 | 93.77 | 380 | 5 | 13 |
| 3817 | 35.19 | -2.47 | GBIF | 11.98 | 76.76 | 19.67 | 17.15 | 122 | 10 | 69.86 | 311 | 37 | 5 |
| 3818 | 34.96 | -2.54 | GBIF | 12.09 | 76.05 | 20.28 | 17.78 | 128 | 11 | 67.01 | 318 | 45 | 7 |
| 3819 | 35.81 | -4.01 | GBIF | 11.35 | 71.84 | 23.42 | 20.43 | 142 | 1 | 88.50 | 341 | 12 | 9 |
| 3820 | 36.20 | -3.51 | GBIF | 11.95 | 71.56 | 22.38 | 19.17 | 162 | 1 | 93.64 | 252 | 7 | 6 |
| 3821 | 34.97 | -2.56 | GBIF | 12.04 | 75.73 | 20.23 | 17.73 | 128 | 11 | 67.69 | 318 | 44 | 7 |
| 3822 | 33.72 | -7.92 | GBIF | 11.54 | 67.10 | 19.58 | 16.28 | 200 | 0 | 115.26 | 248 | 0 | 7 |
| 3823 | 37.07 | -7.27 | GBIF | 9.44 | 61.71 | 25.58 | 22.13 | 271 | 9 | 84.16 | 409 | 44 | 6 |
| 3824 | 36.93 | -3.31 | GBIF | 9.85 | 65.23 | 19.22 | 16.32 | 337 | 21 | 95.36 | 258 | 106 | 8 |
| 3825 | 35.82 | -4.08 | GBIF | 11.06 | 72.75 | 22.67 | 19.77 | 134 | 0 | 91.46 | 345 | 7 | 8 |
| 3826 | 36.31 | -4.28 | GBIF | 11.05 | 70.83 | 21.97 | 18.95 | 133 | 1 | 92.68 | 245 | 6 | 6 |
| 3827 | 34.97 | -2.40 | GBIF | 11.88 | 76.67 | 20.88 | 18.58 | 128 | 15 | 60.75 | 304 | 61 | 4 |
| 3828 | 34.88 | -2.60 | GBIF | 11.98 | 75.84 | 20.35 | 17.93 | 130 | 10 | 67.16 | 320 | 45 | 7 |
| 3829 | 35.67 | -3.56 | GBIF | 10.30 | 69.59 | 21.03 | 17.92 | 174 | 1 | 92.33 | 384 | 9 | 6 |
| 3830 | 34.73 | -1.99 | GBIF | 11.88 | 80.29 | 21.75 | 20.00 | 153 | 20 | 48.98 | 296 | 103 | 6 |
| 3831 | 35.55 | -3.05 | GBIF | 9.25 | 66.55 | 17.62 | 14.90 | 194 | 1 | 90.15 | 432 | 8 | 4 |
| 3832 | 34.94 | -2.69 | GBIF | 11.88 | 75.21 | 20.07 | 17.47 | 132 | 8 | 71.25 | 326 | 36 | 7 |
| 3833 | 35.55 | -3.00 | GBIF | 9.25 | 66.55 | 17.47 | 14.80 | 233 | 3 | 88.30 | 473 | 15 | 10 |
| 3834 | 34.63 | -2.72 | GBIF | 11.29 | 73.80 | 20.38 | 18.30 | 140 | 8 | 69.77 | 335 | 37 | 6 |
| 3835 | 35.29 | -3.01 | GBIF | 13.31 | 75.62 | 21.35 | 18.20 | 96 | 3 | 85.80 | 257 | 11 | 13 |
| 3836 | 38.20 | -7.75 | GBIF | 7.71 | 64.24 | 27.85 | 24.60 | 205 | 6 | 81.14 | 424 | 26 | 7 |
| 3837 | 38.12 | -7.69 | GBIF | 8.04 | 65.38 | 28.08 | 24.82 | 200 | 6 | 80.54 | 390 | 27 | 6 |
| 3838 | 38.18 | -7.72 | GBIF | 7.88 | 64.55 | 27.97 | 24.73 | 192 | 5 | 81.09 | 410 | 23 | 7 |
| 3839 | 35.80 | -3.44 | GBIF | 10.73 | 69.64 | 23.30 | 20.07 | 129 | 1 | 91.06 | 286 | 9 | 7 |
| 3840 | 38.19 | -7.72 | GBIF | 7.93 | 65.03 | 27.92 | 24.72 | 190 | 5 | 81.24 | 408 | 23 | 7 |
| 3841 | 34.89 | -7.74 | GBIF | 11.75 | 68.31 | 23.87 | 20.90 | 136 | 0 | 106.11 | 160 | 0 | 8 |
| 3842 | 35.85 | -4.13 | GBIF | 11.22 | 72.37 | 22.45 | 19.48 | 139 | 0 | 91.75 | 352 | 7 | 9 |
| 3843 | 35.46 | -3.39 | GBIF | 10.00 | 68.49 | 20.05 | 17.05 | 198 | 2 | 88.95 | 444 | 14 | 7 |
| 3844 | 35.90 | -3.79 | GBIF | 11.63 | 70.88 | 23.50 | 20.32 | 149 | 2 | 89.05 | 333 | 13 | 11 |
| 3845 | 31.16 | -6.81 | GBIF | 12.70 | 67.91 | 25.98 | 23.70 | 202 | 0 | 96.67 | 340 | 1 | 3 |
| 3846 | 35.48 | -3.32 | GBIF | 7.65 | 60.24 | 14.27 | 11.92 | 268 | 13 | 78.07 | 529 | 54 | 3 |
| 3847 | 34.80 | -2.20 | GBIF | 12.26 | 78.58 | 21.68 | 19.85 | 140 | 20 | 51.65 | 294 | 91 | 4 |
| 3848 | 35.72 | -3.46 | GBIF | 10.82 | 69.39 | 21.67 | 18.55 | 187 | 1 | 93.29 | 390 | 11 | 8 |
| 3849 | 34.83 | -2.20 | GBIF | 12.24 | 78.98 | 21.58 | 19.70 | 139 | 20 | 52.09 | 349 | 88 | 4 |

| 3850 | 38.07 | -7.75 | GBIF | 8.08 | 64.15 | 27.90 | 24.47 | 201 | 6 | 80.51 | 401 | 28 | 5 |
| --- | --- | --- | --- | --- | --- | --- | --- | --- | --- | --- | --- | --- | --- |
| 3851 | 38.11 | -7.78 | GBIF | 8.16 | 64.75 | 27.73 | 24.45 | 213 | 7 | 79.81 | 402 | 34 | 6 |
| 3852 | 38.08 | -7.73 | GBIF | 7.97 | 63.80 | 27.92 | 24.50 | 205 | 6 | 80.46 | 403 | 30 | 5 |
| 3853 | 35.18 | -7.83 | GBIF | 10.30 | 66.88 | 22.68 | 19.78 | 136 | 0 | 104.63 | 165 | 1 | 4 |
| 3854 | 31.03 | -6.81 | GBIF | 12.38 | 68.37 | 25.93 | 24.27 | 204 | 0 | 97.78 | 141 | 0 | 3 |
| 3855 | 35.86 | -3.29 | GBIF | 10.84 | 69.95 | 21.18 | 18.20 | 193 | 2 | 92.64 | 278 | 13 | 6 |
| 3856 | 35.95 | -3.61 | GBIF | 11.95 | 70.71 | 23.62 | 20.42 | 156 | 1 | 92.40 | 232 | 11 | 12 |
| 3857 | 35.12 | -3.05 | GBIF | 12.83 | 74.56 | 20.33 | 17.33 | 137 | 2 | 86.54 | 331 | 10 | 5 |
| 3858 | 35.88 | -4.09 | GBIF | 11.08 | 71.05 | 22.70 | 19.63 | 146 | 1 | 90.64 | 354 | 11 | 8 |
| 3859 | 35.95 | -4.16 | GBIF | 11.63 | 71.76 | 22.70 | 19.73 | 142 | 1 | 89.45 | 262 | 11 | 8 |
| 3860 | 35.89 | -4.00 | GBIF | 10.38 | 70.63 | 22.78 | 19.70 | 144 | 1 | 90.29 | 344 | 10 | 7 |
| 3861 | 35.86 | -3.58 | GBIF | 9.29 | 65.90 | 23.35 | 19.70 | 144 | 2 | 87.42 | 322 | 14 | 6 |
| 3862 | 35.68 | -3.54 | GBIF | 10.26 | 68.85 | 21.03 | 17.88 | 186 | 1 | 93.29 | 399 | 9 | 13 |
| 3863 | 34.89 | -2.34 | GBIF | 12.23 | 77.43 | 21.65 | 19.48 | 125 | 18 | 55.34 | 294 | 77 | 5 |
| 3864 | 35.89 | -4.07 | GBIF | 10.74 | 71.14 | 22.53 | 19.48 | 145 | 1 | 90.64 | 352 | 11 | 7 |
| 3865 | 34.92 | -2.34 | GBIF | 12.22 | 77.32 | 21.32 | 19.13 | 125 | 18 | 56.44 | 298 | 73 | 6 |
| 3866 | 34.98 | -2.30 | GBIF | 12.20 | 77.71 | 21.23 | 19.07 | 128 | 18 | 56.66 | 298 | 74 | 6 |
| 3867 | 35.95 | -4.05 | GBIF | 10.84 | 70.86 | 22.25 | 19.30 | 145 | 0 | 92.18 | 352 | 8 | 6 |
| 3868 | 34.80 | -2.68 | GBIF | 12.23 | 75.00 | 20.65 | 18.28 | 134 | 9 | 69.24 | 323 | 40 | 8 |
| 3869 | 34.81 | -2.72 | GBIF | 12.21 | 74.90 | 20.57 | 18.17 | 135 | 8 | 71.45 | 327 | 35 | 7 |
| 3870 | 38.36 | -4.59 | GBIF | 9.75 | 64.57 | 18.67 | 15.83 | 158 | 15 | 66.97 | 276 | 52 | 14 |
| 3871 | 35.83 | -3.44 | GBIF | 10.53 | 69.30 | 23.43 | 20.12 | 114 | 1 | 92.40 | 268 | 6 | 6 |
| 3872 | 36.17 | -4.08 | GBIF | 11.48 | 71.72 | 22.57 | 19.48 | 146 | 1 | 91.99 | 275 | 8 | 7 |
| 3873 | 35.90 | -3.53 | GBIF | 10.41 | 68.03 | 23.47 | 20.00 | 171 | 3 | 88.02 | 356 | 17 | 5 |
| 3874 | 36.01 | -3.79 | GBIF | 11.53 | 71.14 | 22.98 | 19.90 | 148 | 1 | 90.82 | 331 | 9 | 8 |
| 3875 | 35.92 | -3.43 | GBIF | 11.28 | 70.08 | 23.43 | 20.20 | 145 | 1 | 92.72 | 220 | 8 | 9 |
| 3876 | 34.75 | -2.22 | GBIF | 12.25 | 78.03 | 21.85 | 20.08 | 141 | 19 | 52.74 | 291 | 87 | 9 |
| 3877 | 34.77 | -2.23 | GBIF | 12.24 | 78.47 | 21.88 | 20.07 | 139 | 19 | 52.50 | 290 | 88 | 13 |
| 3878 | 34.75 | -2.34 | GBIF | 11.86 | 78.02 | 21.05 | 19.12 | 139 | 16 | 56.84 | 312 | 74 | 13 |
| 3879 | 34.71 | -2.28 | GBIF | 12.08 | 78.46 | 21.98 | 20.17 | 134 | 18 | 53.68 | 292 | 83 | 8 |
| 3880 | 35.79 | -3.50 | GBIF | 10.06 | 67.96 | 23.37 | 19.97 | 121 | 1 | 91.29 | 282 | 7 | 7 |
| 3881 | 35.81 | -3.85 | GBIF | 12.16 | 71.94 | 23.77 | 20.57 | 132 | 2 | 88.19 | 316 | 13 | 9 |
| 3882 | 35.57 | -3.05 | GBIF | 8.79 | 65.12 | 16.13 | 13.40 | 217 | 2 | 86.94 | 456 | 14 | 10 |
| 3883 | 34.56 | -2.25 | GBIF | 12.29 | 79.30 | 22.37 | 20.83 | 138 | 14 | 55.03 | 287 | 79 | 7 |
| 3884 | 34.74 | -2.24 | GBIF | 12.23 | 78.37 | 21.95 | 20.18 | 137 | 18 | 52.41 | 289 | 87 | 5 |
| 3885 | 35.81 | -3.98 | GBIF | 11.49 | 72.27 | 23.70 | 20.67 | 145 | 2 | 87.84 | 339 | 16 | 16 |
| 3886 | 35.84 | -3.95 | GBIF | 11.32 | 71.23 | 23.65 | 20.50 | 142 | 1 | 88.92 | 327 | 13 | 8 |
| 3887 | 36.01 | -3.67 | GBIF | 12.00 | 70.59 | 23.43 | 20.23 | 150 | 1 | 91.10 | 324 | 10 | 7 |
| 3888 | 35.44 | -3.35 | GBIF | 8.52 | 65.58 | 15.60 | 12.93 | 230 | 6 | 82.09 | 491 | 29 | 5 |
| 3889 | 35.10 | -3.02 | GBIF | 12.99 | 75.10 | 20.65 | 17.65 | 128 | 3 | 85.17 | 313 | 13 | 5 |
| 3890 | 34.61 | -2.72 | GBIF | 11.27 | 73.16 | 20.48 | 18.42 | 140 | 8 | 69.61 | 334 | 37 | 6 |
| 3891 | 35.14 | -3.00 | GBIF | 13.08 | 75.19 | 20.57 | 17.53 | 127 | 3 | 85.99 | 312 | 12 | 5 |
| 3892 | 34.83 | -3.13 | GBIF | 11.92 | 73.11 | 20.07 | 17.40 | 157 | 2 | 85.76 | 238 | 11 | 5 |
| 3893 | 35.87 | -3.50 | GBIF | 9.27 | 66.19 | 23.30 | 19.70 | 143 | 2 | 86.75 | 316 | 14 | 0 |
| 3894 | 36.06 | -3.63 | GBIF | 12.07 | 71.83 | 23.38 | 20.13 | 154 | 1 | 92.22 | 327 | 10 | 6 |
| 3895 | 34.96 | -3.09 | GBIF | 12.30 | 73.65 | 20.33 | 17.48 | 147 | 2 | 85.56 | 350 | 11 | 5 |
| 3896 | 35.97 | -3.30 | GBIF | 12.01 | 71.91 | 23.42 | 20.38 | 144 | 1 | 93.38 | 213 | 8 | 8 |
| 3897 | 35.16 | -3.12 | GBIF | 12.68 | 74.12 | 20.03 | 17.02 | 150 | 1 | 89.48 | 380 | 6 | 4 |
| 3898 | 36.02 | -3.75 | GBIF | 11.62 | 71.27 | 23.03 | 19.88 | 154 | 1 | 90.65 | 339 | 10 | 8 |
| 3899 | 34.64 | -2.49 | GBIF | 11.50 | 76.16 | 21.02 | 19.12 | 134 | 12 | 61.18 | 316 | 59 | 5 |

| 3900 | 36.13 | -3.67 | GBIF | 11.88 | 71.54 | 23.02 | 19.80 | 154 | 1 | 91.85 | 242 | 10 | 5 |
| --- | --- | --- | --- | --- | --- | --- | --- | --- | --- | --- | --- | --- | --- |
| 3901 | 36.01 | -3.97 | GBIF | 10.93 | 72.35 | 22.13 | 19.17 | 146 | 1 | 91.41 | 352 | 8 | 8 |
| 3902 | 36.07 | -3.72 | GBIF | 11.74 | 71.16 | 23.05 | 19.82 | 158 | 1 | 92.67 | 341 | 10 | 5 |
| 3903 | 36.17 | -4.04 | GBIF | 11.43 | 71.46 | 22.37 | 19.25 | 149 | 1 | 91.69 | 278 | 8 | 7 |
| 3904 | 36.17 | -4.38 | GBIF | 11.23 | 70.65 | 23.07 | 20.07 | 125 | 1 | 91.94 | 240 | 8 | 7 |
| 3905 | 36.18 | -4.01 | GBIF | 11.44 | 71.51 | 22.30 | 19.25 | 151 | 1 | 93.02 | 277 | 7 | 8 |
| 3906 | 34.72 | -2.30 | GBIF | 12.14 | 78.33 | 21.93 | 20.12 | 134 | 18 | 53.52 | 293 | 83 | 10 |
| 3907 | 35.68 | -3.41 | GBIF | 11.23 | 69.72 | 20.62 | 18.25 | 200 | 2 | 92.10 | 289 | 17 | 16 |
| 3908 | 36.04 | -2.14 | GBIF | 11.13 | 73.19 | 26.27 | 23.15 | 106 | 2 | 82.88 | 170 | 11 | 6 |
| 3909 | 34.91 | -2.49 | GBIF | 12.13 | 76.79 | 20.63 | 18.35 | 128 | 13 | 62.49 | 317 | 56 | 7 |
| 3910 | 34.99 | -2.43 | GBIF | 11.88 | 76.12 | 20.60 | 18.22 | 125 | 14 | 62.47 | 312 | 57 | 4 |
| 3911 | 35.93 | -3.86 | GBIF | 10.24 | 68.28 | 23.23 | 19.87 | 143 | 1 | 90.93 | 325 | 10 | 7 |
| 3912 | 35.84 | -3.98 | GBIF | 11.11 | 71.67 | 23.40 | 20.35 | 146 | 1 | 88.68 | 341 | 13 | 8 |
| 3913 | 36.87 | -7.56 | GBIF | 8.41 | 61.83 | 23.43 | 20.60 | 288 | 4 | 88.29 | 450 | 46 | 7 |
| 3914 | 35.92 | -3.62 | GBIF | 11.73 | 70.68 | 23.58 | 20.32 | 147 | 1 | 90.53 | 321 | 11 | 6 |
| 3915 | 35.99 | -3.90 | GBIF | 10.77 | 70.89 | 22.57 | 19.47 | 154 | 1 | 92.07 | 352 | 9 | 7 |
| 3916 | 35.90 | -3.83 | GBIF | 10.43 | 68.59 | 23.37 | 19.97 | 152 | 2 | 87.49 | 344 | 14 | 7 |
| 3917 | 34.89 | -2.22 | GBIF | 12.03 | 78.08 | 21.30 | 19.30 | 140 | 19 | 54.58 | 301 | 82 | 5 |
| 3918 | 34.96 | -2.21 | GBIF | 12.02 | 78.03 | 20.75 | 18.67 | 140 | 18 | 55.96 | 307 | 76 | 6 |
| 3919 | 35.09 | -2.36 | GBIF | 12.04 | 77.19 | 20.35 | 17.95 | 130 | 15 | 62.67 | 308 | 56 | 5 |
| 3920 | 35.05 | -2.53 | GBIF | 12.13 | 76.31 | 20.12 | 17.58 | 125 | 11 | 67.60 | 315 | 42 | 6 |
| 3921 | 34.89 | -2.24 | GBIF | 12.08 | 77.96 | 21.33 | 19.25 | 135 | 19 | 54.57 | 298 | 81 | 5 |
| 3922 | 35.98 | -3.63 | GBIF | 11.91 | 70.88 | 23.52 | 20.33 | 150 | 1 | 90.82 | 323 | 11 | 15 |
| 3923 | 34.83 | -2.39 | GBIF | 12.03 | 76.65 | 21.40 | 19.25 | 130 | 17 | 57.11 | 301 | 75 | 9 |
| 3924 | 35.16 | -2.56 | GBIF | 11.99 | 75.42 | 19.72 | 17.03 | 121 | 9 | 71.17 | 331 | 33 | 8 |
| 3925 | 36.05 | -4.39 | GBIF | 11.46 | 69.87 | 23.13 | 20.12 | 134 | 2 | 89.56 | 255 | 12 | 6 |
| 3926 | 35.13 | -2.47 | GBIF | 11.97 | 76.71 | 19.78 | 17.28 | 130 | 12 | 67.18 | 320 | 46 | 4 |
| 3927 | 34.85 | -2.21 | GBIF | 12.16 | 78.44 | 21.43 | 19.52 | 138 | 20 | 52.57 | 299 | 87 | 4 |
| 3928 | 35.45 | -3.17 | GBIF | 10.58 | 69.17 | 17.28 | 14.60 | 178 | 2 | 84.88 | 420 | 12 | 5 |
| 3929 | 38.33 | -7.74 | GBIF | 8.18 | 66.46 | 27.72 | 24.63 | 220 | 6 | 82.07 | 409 | 27 | 6 |
| 3930 | 35.91 | -3.86 | GBIF | 10.00 | 67.57 | 23.35 | 19.83 | 125 | 1 | 90.10 | 305 | 8 | 7 |
| 3931 | 34.95 | -2.49 | GBIF | 12.14 | 76.36 | 20.58 | 18.22 | 134 | 15 | 61.41 | 321 | 60 | 5 |
| 3932 | 36.07 | -3.62 | GBIF | 12.08 | 71.50 | 23.32 | 20.12 | 153 | 1 | 91.99 | 238 | 10 | 6 |
| 3933 | 35.99 | -3.45 | GBIF | 11.53 | 72.54 | 22.48 | 19.53 | 183 | 0 | 97.17 | 377 | 5 | 5 |
| 3934 | 35.78 | -3.55 | GBIF | 10.14 | 68.06 | 23.42 | 20.00 | 115 | 1 | 91.76 | 278 | 7 | 7 |
| 3935 | 36.11 | -3.52 | GBIF | 11.77 | 71.36 | 23.00 | 19.82 | 171 | 1 | 91.67 | 252 | 11 | 12 |
| 3936 | 34.92 | -2.90 | GBIF | 12.42 | 73.91 | 20.25 | 17.38 | 139 | 4 | 79.57 | 350 | 19 | 7 |
| 3937 | 35.82 | -3.74 | GBIF | 11.29 | 70.13 | 23.57 | 20.20 | 156 | 2 | 86.69 | 349 | 16 | 6 |
| 3938 | 36.08 | -3.49 | GBIF | 11.11 | 70.75 | 22.78 | 19.57 | 177 | 2 | 92.36 | 365 | 12 | 4 |
| 3939 | 37.02 | -7.36 | GBIF | 9.57 | 61.38 | 25.48 | 22.22 | 281 | 8 | 85.61 | 433 | 40 | 15 |
| 3940 | 35.68 | -3.18 | GBIF | 8.85 | 64.60 | 14.80 | 12.52 | 227 | 9 | 79.40 | 459 | 42 | 3 |
| 3941 | 35.85 | -3.98 | GBIF | 10.58 | 71.03 | 22.57 | 19.55 | 154 | 1 | 90.56 | 360 | 11 | 7 |
| 3942 | 35.06 | -2.91 | GBIF | 12.73 | 74.42 | 20.23 | 17.23 | 135 | 4 | 80.37 | 329 | 18 | 8 |
| 3943 | 37.08 | -7.46 | GBIF | 8.19 | 58.93 | 25.52 | 21.97 | 316 | 11 | 84.81 | 449 | 54 | 8 |
| 3944 | 37.08 | -7.54 | GBIF | 8.51 | 59.50 | 26.08 | 22.73 | 337 | 12 | 85.97 | 454 | 58 | 8 |
| 3945 | 37.19 | -7.53 | GBIF | 8.18 | 58.87 | 26.47 | 23.02 | 335 | 13 | 83.76 | 451 | 59 | 7 |
| 3946 | 34.99 | -7.65 | GBIF | 12.09 | 68.31 | 24.33 | 21.32 | 129 | 0 | 106.61 | 153 | 0 | 4 |
| 3947 | 35.90 | -3.49 | GBIF | 10.77 | 69.02 | 23.48 | 20.17 | 165 | 2 | 90.13 | 242 | 14 | 4 |
| 3948 | 36.08 | -4.12 | GBIF | 11.53 | 71.19 | 22.97 | 19.98 | 140 | 1 | 90.85 | 235 | 9 | 7 |
| 3949 | 36.04 | -4.13 | GBIF | 11.51 | 71.04 | 22.97 | 19.93 | 139 | 1 | 91.30 | 239 | 10 | 6 |

| 3950 | 35.52 | -3.14 | GBIF | 11.16 | 69.31 | 19.40 | 16.55 | 159 | 3 | 84.73 | 381 | 17 | 4 |
| --- | --- | --- | --- | --- | --- | --- | --- | --- | --- | --- | --- | --- | --- |
| 3951 | 34.95 | -2.26 | GBIF | 12.03 | 77.63 | 21.30 | 19.12 | 130 | 18 | 55.88 | 298 | 76 | 6 |
| 3952 | 35.94 | -3.75 | GBIF | 11.72 | 71.01 | 23.42 | 20.28 | 143 | 1 | 89.00 | 322 | 11 | 9 |
| 3953 | 34.69 | -2.22 | GBIF | 12.27 | 78.18 | 22.02 | 20.33 | 144 | 17 | 53.39 | 291 | 86 | 5 |
| 3954 | 34.84 | -2.77 | GBIF | 12.34 | 74.80 | 20.40 | 17.90 | 135 | 7 | 72.94 | 328 | 30 | 7 |
| 3955 | 34.92 | -2.36 | GBIF | 12.23 | 77.43 | 21.25 | 19.05 | 125 | 17 | 57.99 | 300 | 70 | 5 |
| 3956 | 36.00 | -3.95 | GBIF | 10.77 | 71.83 | 22.08 | 19.07 | 153 | 0 | 93.05 | 359 | 6 | 8 |
| 3957 | 35.47 | -3.17 | GBIF | 10.88 | 68.83 | 16.90 | 14.22 | 188 | 4 | 82.93 | 342 | 19 | 8 |
| 3958 | 35.92 | -3.66 | GBIF | 11.90 | 70.41 | 23.62 | 20.33 | 146 | 1 | 90.60 | 321 | 11 | 15 |
| 3959 | 35.99 | -3.99 | GBIF | 10.86 | 71.91 | 22.13 | 19.18 | 148 | 0 | 91.85 | 354 | 7 | 7 |
| 3960 | 35.93 | -3.89 | GBIF | 10.13 | 68.47 | 23.15 | 19.80 | 139 | 1 | 88.79 | 324 | 12 | 7 |
| 3961 | 35.92 | -3.91 | GBIF | 10.30 | 69.13 | 23.17 | 19.90 | 138 | 1 | 89.75 | 322 | 11 | 6 |
| 3962 | 35.57 | -3.14 | GBIF | 11.01 | 70.12 | 19.42 | 16.58 | 169 | 3 | 87.52 | 386 | 14 | 4 |
| 3963 | 35.59 | -3.20 | GBIF | 11.05 | 69.94 | 19.70 | 16.82 | 175 | 2 | 89.47 | 393 | 11 | 3 |
| 3964 | 34.40 | -2.02 | GBIF | 11.76 | 81.66 | 22.40 | 20.98 | 163 | 15 | 53.41 | 289 | 90 | 8 |
| 3965 | 35.83 | -3.81 | GBIF | 12.31 | 72.40 | 23.78 | 20.65 | 137 | 1 | 91.30 | 316 | 9 | 8 |
| 3966 | 35.52 | -3.22 | GBIF | 10.53 | 68.85 | 19.45 | 16.53 | 172 | 3 | 85.14 | 397 | 20 | 3 |
| 3967 | 38.16 | -4.08 | GBIF | 10.32 | 67.43 | 24.13 | 20.68 | 124 | 6 | 76.42 | 233 | 28 | 6 |
| 3968 | 35.96 | -3.41 | GBIF | 12.05 | 72.16 | 23.65 | 20.60 | 148 | 0 | 93.84 | 308 | 7 | 7 |
| 3969 | 34.92 | -2.11 | GBIF | 12.20 | 79.22 | 21.22 | 19.32 | 144 | 20 | 52.67 | 299 | 88 | 5 |
| 3970 | 35.45 | -3.09 | GBIF | 12.07 | 73.58 | 21.17 | 18.15 | 95 | 1 | 88.37 | 267 | 5 | 6 |
| 3971 | 35.89 | -3.98 | GBIF | 10.48 | 70.78 | 23.05 | 19.97 | 151 | 1 | 91.15 | 344 | 11 | 7 |
| 3972 | 34.80 | -2.33 | GBIF | 11.96 | 77.65 | 21.73 | 19.65 | 133 | 18 | 55.18 | 295 | 80 | 4 |
| 3973 | 35.55 | -3.02 | GBIF | 9.32 | 66.55 | 17.90 | 15.22 | 213 | 2 | 89.33 | 452 | 10 | 4 |
| 3974 | 34.75 | -2.21 | GBIF | 12.24 | 78.47 | 21.85 | 20.03 | 140 | 19 | 52.17 | 291 | 89 | 13 |
| 3975 | 34.90 | -2.50 | GBIF | 12.17 | 76.52 | 20.70 | 18.40 | 129 | 14 | 61.24 | 317 | 60 | 8 |
| 3976 | 35.57 | -3.02 | GBIF | 8.54 | 64.22 | 15.22 | 12.20 | 246 | 4 | 85.01 | 493 | 20 | 10 |
| 3977 | 34.85 | -2.22 | GBIF | 12.22 | 78.31 | 21.53 | 19.60 | 137 | 20 | 52.79 | 297 | 87 | 4 |
| 3978 | 37.18 | -7.42 | GBIF | 7.97 | 59.07 | 24.57 | 20.93 | 306 | 8 | 84.99 | 438 | 48 | 9 |
| 3979 | 35.97 | -3.80 | GBIF | 11.39 | 70.76 | 23.15 | 20.03 | 147 | 1 | 91.02 | 329 | 10 | 8 |
| 3980 | 34.92 | -2.28 | GBIF | 12.18 | 78.04 | 21.55 | 19.42 | 129 | 19 | 54.52 | 336 | 80 | 6 |
| 3981 | 35.41 | -3.05 | GBIF | 13.43 | 75.85 | 22.30 | 19.20 | 90 | 2 | 86.87 | 250 | 7 | 14 |
| 3982 | 34.91 | -2.29 | GBIF | 12.23 | 77.37 | 21.68 | 19.53 | 125 | 19 | 54.49 | 292 | 80 | 6 |
| 3983 | 34.84 | -6.32 | GBIF | 11.59 | 68.19 | 23.23 | 20.48 | 130 | 0 | 111.84 | 157 | 1 | 3 |
| 3984 | 35.84 | -3.99 | GBIF | 10.82 | 71.22 | 23.03 | 19.98 | 149 | 1 | 90.00 | 349 | 12 | 7 |
| 3985 | 34.99 | -2.76 | GBIF | 12.26 | 74.75 | 19.93 | 17.13 | 130 | 6 | 74.52 | 344 | 27 | 7 |
| 3986 | 35.46 | -3.12 | GBIF | 10.68 | 69.83 | 19.07 | 16.37 | 145 | 1 | 89.90 | 382 | 5 | 8 |
| 3987 | 35.53 | -3.00 | GBIF | 10.38 | 69.22 | 19.98 | 17.28 | 148 | 2 | 86.38 | 344 | 10 | 4 |
| 3988 | 35.46 | -3.09 | GBIF | 11.28 | 71.41 | 20.43 | 17.37 | 108 | 1 | 89.79 | 305 | 3 | 4 |
| 3989 | 37.07 | -7.47 | GBIF | 8.23 | 59.23 | 25.87 | 22.38 | 317 | 12 | 84.73 | 449 | 55 | 10 |
| 3990 | 35.52 | -3.34 | GBIF | 9.61 | 66.26 | 18.77 | 16.30 | 271 | 8 | 89.13 | 536 | 39 | 5 |
| 3991 | 35.50 | -3.30 | GBIF | 7.53 | 59.79 | 13.92 | 11.55 | 291 | 17 | 76.62 | 388 | 71 | 3 |
| 3992 | 35.41 | -3.27 | GBIF | 9.68 | 66.78 | 15.93 | 13.10 | 203 | 2 | 84.85 | 304 | 12 | 3 |
| 3993 | 35.85 | -3.88 | GBIF | 11.44 | 70.63 | 23.55 | 20.30 | 134 | 1 | 89.48 | 319 | 10 | 11 |
| 3994 | 35.95 | -3.91 | GBIF | 10.76 | 70.78 | 23.03 | 19.90 | 149 | 1 | 90.74 | 339 | 11 | 6 |
| 3995 | 35.96 | -3.92 | GBIF | 10.92 | 71.82 | 22.78 | 19.75 | 145 | 1 | 90.61 | 339 | 9 | 7 |
| 3996 | 35.81 | -3.87 | GBIF | 12.04 | 71.68 | 23.77 | 20.58 | 138 | 1 | 89.58 | 322 | 12 | 12 |
| 3997 | 34.04 | -2.05 | GBIF | 11.84 | 80.01 | 23.13 | 22.42 | 151 | 9 | 59.85 | 186 | 69 | 7 |
| 3998 | 34.62 | -1.99 | GBIF | 11.79 | 80.76 | 21.60 | 19.98 | 165 | 19 | 49.93 | 305 | 103 | 4 |
| 3999 | 35.98 | -3.82 | GBIF | 11.27 | 70.86 | 23.10 | 20.00 | 151 | 1 | 91.35 | 333 | 10 | 8 |

| 4000 | 35.57 | -3.13 | GBIF | 10.76 | 69.41 | 19.28 | 16.40 | 171 | 3 | 87.15 | 389 | 15 | 4 |
| --- | --- | --- | --- | --- | --- | --- | --- | --- | --- | --- | --- | --- | --- |
| 4001 | 34.49 | -8.78 | GBIF | 12.17 | 70.33 | 22.12 | 19.05 | 178 | 0 | 111.53 | 237 | 0 | 10 |
| 4002 | 38.14 | -7.79 | GBIF | 8.23 | 65.34 | 27.77 | 24.48 | 225 | 8 | 77.30 | 420 | 37 | 7 |
| 4003 | 34.80 | -2.32 | GBIF | 12.11 | 77.62 | 21.78 | 19.78 | 135 | 18 | 55.10 | 296 | 81 | 4 |
| 4004 | 36.02 | -4.19 | GBIF | 11.48 | 71.33 | 23.03 | 20.00 | 138 | 1 | 89.22 | 249 | 11 | 8 |
| 4005 | 36.08 | -4.16 | GBIF | 11.57 | 71.01 | 22.90 | 19.93 | 139 | 1 | 91.41 | 240 | 9 | 7 |
| 4006 | 34.75 | -2.28 | GBIF | 12.25 | 78.03 | 21.93 | 20.12 | 133 | 19 | 53.82 | 291 | 83 | 5 |
| 4007 | 34.82 | -2.58 | GBIF | 11.95 | 75.16 | 20.50 | 18.13 | 132 | 11 | 65.94 | 321 | 48 | 7 |
| 4008 | 35.83 | -3.41 | GBIF | 10.81 | 69.28 | 23.45 | 20.18 | 140 | 1 | 91.91 | 299 | 10 | 7 |
| 4009 | 35.44 | -3.15 | GBIF | 10.58 | 69.63 | 17.20 | 14.57 | 178 | 2 | 85.24 | 417 | 13 | 13 |
| 4010 | 35.52 | -3.24 | GBIF | 9.67 | 66.21 | 19.02 | 15.93 | 179 | 3 | 84.71 | 410 | 21 | 14 |
| 4011 | 37.13 | -7.37 | GBIF | 9.11 | 60.72 | 25.12 | 21.60 | 285 | 6 | 86.77 | 432 | 36 | 8 |
| 4012 | 35.43 | -3.36 | GBIF | 9.21 | 68.21 | 17.33 | 14.62 | 202 | 2 | 86.35 | 465 | 13 | 6 |
| 4013 | 35.59 | -3.23 | GBIF | 9.68 | 66.78 | 19.47 | 16.40 | 189 | 1 | 90.71 | 408 | 11 | 10 |
| 4014 | 35.58 | -3.32 | GBIF | 10.52 | 67.85 | 19.65 | 17.25 | 230 | 4 | 91.71 | 452 | 25 | 6 |
| 4015 | 34.85 | -2.12 | GBIF | 12.22 | 79.33 | 21.53 | 19.77 | 148 | 20 | 52.28 | 294 | 91 | 4 |
| 4016 | 37.04 | -7.35 | GBIF | 9.73 | 61.22 | 25.50 | 22.22 | 276 | 7 | 86.13 | 429 | 38 | 10 |
| 4017 | 36.07 | -3.97 | GBIF | 11.50 | 70.99 | 22.90 | 19.73 | 142 | 1 | 90.85 | 332 | 9 | 8 |
| 4018 | 34.92 | -2.45 | GBIF | 12.10 | 76.58 | 20.85 | 18.55 | 129 | 14 | 61.83 | 310 | 59 | 4 |
| 4019 | 35.74 | -3.08 | GBIF | 9.82 | 68.71 | 13.13 | 10.20 | 243 | 7 | 80.88 | 506 | 29 | 5 |
| 4020 | 35.85 | -3.82 | GBIF | 12.27 | 72.21 | 23.77 | 20.63 | 133 | 1 | 90.88 | 313 | 9 | 11 |
| 4021 | 34.85 | -2.34 | GBIF | 12.18 | 77.11 | 21.65 | 19.50 | 131 | 18 | 55.88 | 298 | 78 | 4 |
| 4022 | 36.91 | -3.36 | GBIF | 11.13 | 66.62 | 21.03 | 18.25 | 296 | 20 | 93.39 | 249 | 82 | 10 |
| 4023 | 34.61 | -2.48 | GBIF | 11.46 | 75.88 | 20.97 | 19.10 | 133 | 12 | 60.66 | 317 | 59 | 5 |
| 4024 | 34.78 | -2.60 | GBIF | 11.83 | 75.37 | 20.55 | 18.25 | 133 | 10 | 66.39 | 324 | 45 | 15 |
| 4025 | 35.86 | -3.94 | GBIF | 11.10 | 70.70 | 23.53 | 20.35 | 139 | 1 | 88.38 | 326 | 12 | 7 |
| 4026 | 35.92 | -3.41 | GBIF | 11.41 | 70.42 | 23.47 | 20.28 | 145 | 1 | 92.87 | 216 | 8 | 13 |
| 4027 | 36.12 | -3.69 | GBIF | 11.84 | 71.34 | 23.00 | 19.75 | 160 | 1 | 92.71 | 340 | 10 | 5 |
| 4028 | 34.81 | -2.35 | GBIF | 11.97 | 77.76 | 21.63 | 19.55 | 133 | 18 | 55.74 | 297 | 78 | 4 |
| 4029 | 35.76 | -3.11 | GBIF | 9.33 | 67.15 | 13.32 | 10.62 | 244 | 10 | 76.60 | 508 | 45 | 4 |
| 4030 | 35.76 | -3.07 | GBIF | 9.98 | 69.81 | 11.20 | 8.42 | 253 | 11 | 72.78 | 539 | 50 | 5 |
| 4031 | 35.54 | -3.12 | GBIF | 10.69 | 68.98 | 19.28 | 16.40 | 175 | 3 | 87.17 | 392 | 16 | 4 |
| 4032 | 35.60 | -3.33 | GBIF | 10.84 | 68.19 | 19.82 | 17.43 | 229 | 4 | 91.90 | 450 | 25 | 10 |
| 4033 | 35.55 | -3.14 | GBIF | 11.09 | 69.76 | 19.33 | 16.47 | 172 | 3 | 86.86 | 392 | 17 | 4 |
| 4034 | 34.93 | -2.41 | GBIF | 11.93 | 76.44 | 21.08 | 18.80 | 132 | 16 | 59.98 | 301 | 66 | 4 |
| 4035 | 37.07 | -7.49 | GBIF | 8.31 | 59.35 | 25.57 | 22.12 | 317 | 11 | 84.89 | 452 | 53 | 11 |
| 4036 | 37.93 | -4.17 | GBIF | 10.26 | 66.61 | 21.45 | 18.48 | 133 | 16 | 63.24 | 269 | 63 | 13 |
| 4037 | 34.85 | -2.25 | GBIF | 12.23 | 78.37 | 21.68 | 19.67 | 134 | 20 | 53.42 | 294 | 84 | 5 |
| 4038 | 35.75 | -3.36 | GBIF | 11.02 | 69.34 | 21.10 | 18.68 | 202 | 2 | 93.45 | 271 | 17 | 7 |
| 4039 | 36.05 | -3.61 | GBIF | 12.07 | 71.83 | 23.35 | 20.15 | 151 | 1 | 91.86 | 234 | 10 | 11 |
| 4040 | 36.00 | -3.35 | GBIF | 12.08 | 71.50 | 23.50 | 20.42 | 154 | 1 | 94.99 | 215 | 7 | 7 |
| 4041 | 37.02 | -7.25 | GBIF | 8.90 | 62.24 | 24.45 | 21.15 | 252 | 6 | 85.38 | 410 | 37 | 5 |
| 4042 | 34.80 | -7.62 | GBIF | 11.92 | 67.33 | 23.63 | 20.60 | 132 | 0 | 107.79 | 155 | 0 | 6 |
| 4043 | 36.09 | -3.68 | GBIF | 11.76 | 71.26 | 22.70 | 19.45 | 158 | 1 | 91.69 | 345 | 10 | 5 |
| 4044 | 35.00 | -2.39 | GBIF | 11.81 | 76.18 | 20.57 | 18.23 | 128 | 15 | 61.41 | 308 | 59 | 4 |
| 4045 | 34.34 | -2.06 | GBIF | 12.07 | 81.53 | 22.85 | 21.57 | 151 | 13 | 54.90 | 282 | 81 | 6 |
| 4046 | 34.88 | -1.66 | GBIF | 11.94 | 80.69 | 20.57 | 18.95 | 176 | 27 | 45.03 | 312 | 135 | 5 |
| 4047 | 35.96 | -3.86 | GBIF | 11.02 | 70.67 | 23.03 | 19.90 | 149 | 1 | 90.49 | 336 | 11 | 7 |
| 4048 | 35.96 | -3.82 | GBIF | 11.28 | 70.52 | 23.17 | 20.03 | 150 | 1 | 91.16 | 333 | 10 | 8 |
| 4049 | 35.92 | -3.87 | GBIF | 9.98 | 67.45 | 23.23 | 19.78 | 132 | 1 | 89.33 | 312 | 11 | 13 |

| 4050 | 35.44 | -3.12 | GBIF | 11.34 | 71.78 | 19.73 | 17.02 | 131 | 1 | 89.39 | 363 | 5 | 7 |
| --- | --- | --- | --- | --- | --- | --- | --- | --- | --- | --- | --- | --- | --- |
| 4051 | 35.52 | -3.38 | GBIF | 11.05 | 69.94 | 20.47 | 18.15 | 199 | 4 | 88.36 | 419 | 23 | 10 |
| 4052 | 35.43 | -3.29 | GBIF | 8.75 | 66.79 | 13.50 | 10.75 | 220 | 4 | 82.18 | 348 | 20 | 3 |
| 4053 | 35.57 | -3.40 | GBIF | 11.50 | 70.55 | 21.02 | 18.68 | 188 | 3 | 88.74 | 283 | 21 | 16 |
| 4054 | 35.56 | -3.01 | GBIF | 8.84 | 65.49 | 16.15 | 13.52 | 242 | 3 | 85.61 | 486 | 18 | 10 |
| 4055 | 35.50 | -3.26 | GBIF | 7.55 | 59.92 | 13.68 | 11.37 | 245 | 16 | 73.11 | 490 | 65 | 4 |
| 4056 | 35.49 | -3.23 | GBIF | 8.74 | 64.28 | 14.43 | 12.25 | 223 | 10 | 75.48 | 456 | 50 | 5 |
| 4057 | 35.54 | -3.26 | GBIF | 7.94 | 61.09 | 14.60 | 12.28 | 267 | 13 | 76.84 | 511 | 66 | 11 |
| 4058 | 35.00 | -2.34 | GBIF | 12.15 | 77.39 | 20.95 | 18.72 | 129 | 16 | 58.94 | 302 | 66 | 5 |
| 4059 | 35.00 | -2.32 | GBIF | 12.23 | 77.37 | 21.05 | 18.83 | 130 | 17 | 58.37 | 301 | 69 | 5 |
| 4060 | 35.59 | -3.35 | GBIF | 11.19 | 69.51 | 20.32 | 17.97 | 211 | 4 | 89.73 | 428 | 24 | 9 |
| 4061 | 35.44 | -3.28 | GBIF | 8.58 | 68.12 | 11.97 | 9.23 | 222 | 7 | 77.75 | 377 | 30 | 3 |
| 4062 | 34.81 | -2.28 | GBIF | 12.13 | 77.78 | 21.78 | 19.80 | 133 | 19 | 53.71 | 294 | 83 | 5 |
| 4063 | 35.53 | -3.33 | GBIF | 9.94 | 66.72 | 19.20 | 16.78 | 254 | 6 | 90.23 | 500 | 32 | 12 |
| 4064 | 35.93 | -3.85 | GBIF | 10.29 | 68.16 | 23.23 | 19.85 | 141 | 1 | 89.46 | 323 | 11 | 7 |
| 4065 | 35.93 | -3.95 | GBIF | 10.85 | 70.92 | 23.02 | 19.97 | 144 | 1 | 90.31 | 337 | 10 | 6 |
| 4066 | 34.59 | -7.83 | GBIF | 12.24 | 67.63 | 23.18 | 20.32 | 148 | 0 | 106.17 | 172 | 0 | 5 |
| 4067 | 34.52 | -7.99 | GBIF | 11.82 | 68.30 | 23.00 | 20.22 | 150 | 0 | 106.57 | 183 | 0 | 5 |
| 4068 | 36.12 | -4.04 | GBIF | 11.42 | 71.35 | 22.55 | 19.37 | 147 | 1 | 92.35 | 344 | 8 | 7 |
| 4069 | 36.08 | -3.90 | GBIF | 11.27 | 70.86 | 22.88 | 19.68 | 147 | 1 | 92.07 | 337 | 8 | 8 |
| 4070 | 35.14 | -1.78 | GBIF | 12.19 | 80.74 | 19.98 | 18.33 | 175 | 24 | 53.58 | 395 | 102 | 5 |
| 4071 | 35.85 | -3.64 | GBIF | 9.57 | 65.98 | 23.43 | 19.73 | 144 | 1 | 89.54 | 323 | 12 | 6 |
| 4072 | 35.08 | -2.09 | GBIF | 12.21 | 79.27 | 20.37 | 18.33 | 148 | 18 | 58.04 | 364 | 73 | 4 |
| 4073 | 34.41 | -7.85 | GBIF | 12.03 | 67.23 | 22.53 | 19.70 | 145 | 0 | 109.50 | 185 | 0 | 6 |
| 4074 | 34.56 | -7.84 | GBIF | 12.35 | 67.86 | 23.18 | 20.37 | 150 | 0 | 106.41 | 175 | 0 | 5 |
| 4075 | 34.58 | -8.00 | GBIF | 11.57 | 68.04 | 22.32 | 19.42 | 145 | 0 | 107.01 | 191 | 0 | 5 |
| 4076 | 34.43 | -7.87 | GBIF | 12.07 | 67.41 | 22.67 | 19.78 | 145 | 0 | 108.33 | 184 | 0 | 7 |
| 4077 | 36.10 | -4.19 | GBIF | 11.65 | 71.91 | 22.68 | 19.75 | 140 | 1 | 91.57 | 245 | 8 | 7 |
| 4078 | 35.88 | -3.81 | GBIF | 11.75 | 71.21 | 23.65 | 20.43 | 133 | 1 | 90.26 | 314 | 9 | 11 |
| 4079 | 35.94 | -3.83 | GBIF | 10.82 | 69.39 | 23.23 | 19.97 | 148 | 1 | 89.81 | 329 | 12 | 7 |
| 4080 | 34.65 | -2.60 | GBIF | 11.73 | 75.21 | 19.92 | 17.93 | 145 | 10 | 66.20 | 339 | 46 | 7 |
| 4081 | 34.69 | -2.63 | GBIF | 11.43 | 74.67 | 19.67 | 17.58 | 143 | 9 | 67.24 | 345 | 44 | 7 |
| 4082 | 35.77 | -3.15 | GBIF | 8.71 | 64.03 | 14.47 | 12.15 | 244 | 13 | 77.52 | 484 | 55 | 10 |
| 4083 | 36.10 | -3.91 | GBIF | 11.23 | 70.65 | 22.68 | 19.47 | 153 | 1 | 92.97 | 345 | 8 | 8 |
| 4084 | 36.94 | -3.31 | GBIF | 10.02 | 65.52 | 19.73 | 16.88 | 332 | 21 | 95.38 | 249 | 114 | 7 |
| 4085 | 36.17 | -3.93 | GBIF | 11.15 | 71.02 | 22.32 | 19.18 | 158 | 1 | 93.73 | 356 | 7 | 6 |
| 4086 | 34.83 | -7.58 | GBIF | 11.76 | 67.19 | 23.50 | 20.40 | 128 | 0 | 108.92 | 154 | 0 | 7 |
| 4087 | 34.91 | -7.27 | GBIF | 11.63 | 67.20 | 23.30 | 19.98 | 115 | 0 | 110.89 | 147 | 0 | 5 |
| 4088 | 34.98 | -7.24 | GBIF | 11.81 | 68.26 | 23.68 | 20.38 | 111 | 0 | 111.56 | 145 | 0 | 4 |
| 4089 | 34.86 | -2.35 | GBIF | 12.22 | 77.32 | 21.65 | 19.48 | 128 | 18 | 55.60 | 297 | 78 | 5 |
| 4090 | 35.52 | -3.13 | GBIF | 10.86 | 69.16 | 19.28 | 16.43 | 165 | 3 | 85.35 | 388 | 17 | 4 |
| 4091 | 35.49 | -3.17 | GBIF | 11.53 | 69.48 | 18.52 | 15.65 | 176 | 4 | 83.93 | 415 | 20 | 8 |
| 4092 | 34.91 | -6.87 | GBIF | 11.35 | 67.16 | 23.50 | 20.23 | 119 | 0 | 112.95 | 151 | 0 | 4 |
| 4093 | 34.88 | -7.70 | GBIF | 11.88 | 67.86 | 23.88 | 20.85 | 134 | 0 | 107.58 | 155 | 0 | 8 |
| 4094 | 36.18 | -4.16 | GBIF | 11.54 | 71.69 | 22.62 | 19.65 | 143 | 1 | 88.85 | 255 | 10 | 7 |
| 4095 | 35.76 | -3.56 | GBIF | 10.18 | 68.34 | 23.33 | 19.95 | 128 | 1 | 91.51 | 292 | 8 | 7 |
| 4096 | 35.86 | -3.28 | GBIF | 10.89 | 69.82 | 21.10 | 18.10 | 196 | 2 | 92.99 | 278 | 14 | 6 |
| 4097 | 35.65 | -3.44 | GBIF | 11.47 | 70.78 | 21.23 | 18.82 | 189 | 2 | 91.81 | 393 | 16 | 10 |
| 4098 | 35.78 | -3.28 | GBIF | 10.64 | 68.22 | 20.12 | 17.65 | 213 | 4 | 92.60 | 285 | 21 | 6 |
| 4099 | 35.11 | -2.79 | GBIF | 12.64 | 74.80 | 19.73 | 16.88 | 129 | 6 | 77.70 | 325 | 22 | 9 |

| 4100 | 35.57 | -3.21 | GBIF | 11.20 | 70.00 | 19.78 | 16.85 | 168 | 2 | 86.97 | 387 | 14 | 3 |
| --- | --- | --- | --- | --- | --- | --- | --- | --- | --- | --- | --- | --- | --- |
| 4101 | 35.99 | -3.96 | GBIF | 10.84 | 72.28 | 22.18 | 19.18 | 144 | 1 | 91.36 | 348 | 7 | 8 |
| 4102 | 35.52 | -3.31 | GBIF | 8.77 | 63.53 | 17.20 | 14.72 | 295 | 11 | 85.01 | 569 | 55 | 4 |
| 4103 | 36.14 | -4.19 | GBIF | 11.81 | 71.57 | 22.80 | 19.78 | 137 | 1 | 91.46 | 237 | 8 | 7 |
| 4104 | 36.04 | -3.75 | GBIF | 11.59 | 71.11 | 23.02 | 19.83 | 150 | 1 | 91.59 | 331 | 9 | 7 |
| 4105 | 36.04 | -3.65 | GBIF | 12.06 | 71.78 | 23.33 | 20.10 | 159 | 2 | 90.81 | 338 | 12 | 6 |
| 4106 | 36.34 | -3.94 | GBIF | 11.26 | 70.81 | 21.02 | 17.77 | 156 | 1 | 93.48 | 288 | 8 | 6 |
| 4107 | 36.10 | -4.11 | GBIF | 11.52 | 71.09 | 22.72 | 19.68 | 139 | 1 | 91.41 | 238 | 9 | 7 |
| 4108 | 36.29 | -3.86 | GBIF | 11.27 | 71.31 | 21.50 | 18.37 | 161 | 1 | 93.32 | 282 | 7 | 6 |
| 4109 | 35.95 | -3.97 | GBIF | 10.78 | 71.89 | 22.63 | 19.63 | 146 | 1 | 91.80 | 344 | 8 | 6 |
| 4110 | 35.43 | -3.24 | GBIF | 9.26 | 67.09 | 13.90 | 11.10 | 208 | 5 | 79.17 | 335 | 26 | 3 |
| 4111 | 35.00 | -2.18 | GBIF | 12.26 | 79.09 | 20.73 | 18.65 | 143 | 19 | 55.83 | 359 | 78 | 6 |
| 4112 | 34.61 | -2.79 | GBIF | 11.43 | 72.82 | 20.30 | 18.27 | 146 | 7 | 71.88 | 245 | 33 | 6 |
| 4113 | 34.66 | -2.62 | GBIF | 11.74 | 74.79 | 19.95 | 17.95 | 141 | 9 | 67.39 | 338 | 43 | 7 |
| 4114 | 36.02 | -4.18 | GBIF | 11.42 | 71.35 | 23.00 | 19.95 | 136 | 1 | 89.06 | 247 | 11 | 7 |
| 4115 | 36.16 | -3.97 | GBIF | 11.25 | 71.66 | 22.50 | 19.35 | 152 | 1 | 92.34 | 349 | 9 | 6 |
| 4116 | 36.12 | -3.99 | GBIF | 11.38 | 71.09 | 22.37 | 19.20 | 146 | 1 | 91.90 | 344 | 8 | 7 |
| 4117 | 35.74 | -3.57 | GBIF | 9.90 | 68.28 | 23.10 | 19.78 | 137 | 1 | 91.37 | 307 | 9 | 7 |
| 4118 | 35.15 | -1.93 | GBIF | 12.03 | 79.69 | 20.08 | 18.07 | 160 | 21 | 55.37 | 375 | 85 | 4 |
| 4119 | 34.52 | -2.27 | GBIF | 12.44 | 79.75 | 22.43 | 20.97 | 138 | 13 | 56.15 | 288 | 75 | 11 |
| 4120 | 36.07 | -3.82 | GBIF | 11.43 | 71.01 | 22.97 | 19.80 | 142 | 1 | 91.12 | 325 | 8 | 6 |
| 4121 | 36.12 | -4.23 | GBIF | 11.67 | 72.02 | 22.83 | 19.88 | 138 | 1 | 92.38 | 240 | 8 | 8 |
| 4122 | 35.59 | -3.09 | GBIF | 9.06 | 65.64 | 15.98 | 13.77 | 228 | 5 | 84.98 | 466 | 25 | 4 |
| 4123 | 35.96 | -3.46 | GBIF | 11.74 | 72.03 | 23.55 | 20.50 | 156 | 0 | 94.49 | 326 | 8 | 8 |
| 4124 | 35.96 | -3.58 | GBIF | 11.78 | 70.98 | 23.57 | 20.43 | 152 | 1 | 91.16 | 322 | 11 | 5 |
| 4125 | 36.10 | -3.83 | GBIF | 11.19 | 70.83 | 22.63 | 19.47 | 160 | 1 | 92.87 | 348 | 9 | 6 |
| 4126 | 34.90 | -1.73 | GBIF | 11.88 | 80.78 | 19.70 | 18.15 | 177 | 26 | 46.79 | 326 | 124 | 7 |
| 4127 | 34.98 | -1.79 | GBIF | 12.27 | 80.76 | 20.15 | 18.67 | 170 | 24 | 49.39 | 311 | 112 | 6 |
| 4128 | 34.82 | -1.42 | GBIF | 11.99 | 81.02 | 20.78 | 19.28 | 184 | 38 | 39.82 | 313 | 169 | 8 |
| 4129 | 34.91 | -2.42 | GBIF | 11.98 | 76.82 | 21.05 | 18.83 | 132 | 15 | 60.41 | 306 | 65 | 4 |
| 4130 | 35.81 | -3.76 | GBIF | 11.82 | 70.81 | 23.60 | 20.33 | 160 | 2 | 87.16 | 357 | 15 | 6 |
| 4131 | 36.18 | -3.87 | GBIF | 11.29 | 71.47 | 22.20 | 19.12 | 160 | 1 | 93.34 | 359 | 8 | 7 |
| 4132 | 34.99 | -2.17 | GBIF | 12.23 | 78.87 | 20.70 | 18.63 | 143 | 19 | 56.01 | 358 | 78 | 6 |
| 4133 | 35.60 | -3.35 | GBIF | 11.21 | 69.19 | 20.22 | 17.88 | 215 | 4 | 90.95 | 430 | 24 | 14 |
| 4134 | 34.99 | -2.22 | GBIF | 11.95 | 77.60 | 20.90 | 18.70 | 137 | 18 | 57.21 | 300 | 73 | 6 |
| 4135 | 35.84 | -3.48 | GBIF | 9.77 | 67.36 | 23.42 | 19.90 | 136 | 2 | 88.05 | 302 | 12 | 0 |
| 4136 | 35.86 | -3.81 | GBIF | 12.23 | 72.82 | 23.68 | 20.57 | 135 | 1 | 90.91 | 315 | 9 | 11 |
| 4137 | 34.66 | -2.43 | GBIF | 11.38 | 76.91 | 20.93 | 18.97 | 138 | 13 | 59.82 | 319 | 63 | 4 |
| 4138 | 34.81 | -2.40 | GBIF | 11.98 | 76.76 | 21.30 | 19.15 | 132 | 17 | 58.50 | 302 | 70 | 7 |
| 4139 | 34.99 | -2.51 | GBIF | 12.18 | 76.09 | 20.43 | 17.98 | 134 | 13 | 63.93 | 319 | 53 | 6 |
| 4140 | 34.93 | -2.56 | GBIF | 12.02 | 75.63 | 20.42 | 17.95 | 128 | 11 | 66.22 | 316 | 47 | 7 |
| 4141 | 36.16 | -4.19 | GBIF | 11.77 | 71.75 | 22.77 | 19.80 | 136 | 1 | 90.37 | 242 | 9 | 7 |
| 4142 | 35.59 | -3.39 | GBIF | 11.57 | 70.58 | 20.98 | 18.63 | 194 | 3 | 90.01 | 402 | 20 | 12 |
| 4143 | 35.90 | -3.56 | GBIF | 11.12 | 69.48 | 23.53 | 20.22 | 171 | 2 | 88.50 | 359 | 17 | 5 |
| 4144 | 35.49 | -3.33 | GBIF | 8.51 | 63.02 | 16.68 | 14.22 | 267 | 10 | 82.51 | 535 | 49 | 4 |
| 4145 | 35.58 | -3.30 | GBIF | 9.87 | 65.78 | 18.87 | 16.38 | 253 | 7 | 90.22 | 494 | 34 | 9 |
| 4146 | 34.94 | -3.17 | GBIF | 12.21 | 73.99 | 20.20 | 17.37 | 157 | 2 | 88.18 | 386 | 9 | 4 |
| 4147 | 35.32 | -3.06 | GBIF | 13.22 | 75.57 | 21.27 | 18.12 | 99 | 2 | 86.93 | 261 | 9 | 5 |
| 4148 | 35.26 | -3.00 | GBIF | 13.38 | 75.56 | 21.15 | 18.00 | 102 | 3 | 84.88 | 268 | 12 | 6 |
| 4149 | 35.40 | -3.09 | GBIF | 12.70 | 74.71 | 21.03 | 17.98 | 102 | 2 | 87.90 | 282 | 6 | 13 |

| 4150 | 35.03 | -2.74 | GBIF | 12.13 | 74.44 | 19.78 | 17.02 | 129 | 7 | 74.77 | 325 | 28 | 7 |
| --- | --- | --- | --- | --- | --- | --- | --- | --- | --- | --- | --- | --- | --- |
| 4151 | 35.17 | -2.58 | GBIF | 12.20 | 74.85 | 19.78 | 17.03 | 120 | 9 | 72.91 | 308 | 31 | 9 |
| 4152 | 37.11 | -7.22 | GBIF | 9.73 | 62.39 | 25.85 | 22.50 | 270 | 9 | 84.89 | 402 | 39 | 5 |
| 4153 | 37.13 | -7.20 | GBIF | 9.94 | 62.92 | 25.78 | 21.93 | 267 | 7 | 85.31 | 396 | 37 | 5 |
| 4154 | 37.07 | -7.23 | GBIF | 9.22 | 61.44 | 25.50 | 22.05 | 266 | 8 | 85.07 | 400 | 41 | 13 |
| 4155 | 37.05 | -7.39 | GBIF | 9.39 | 60.98 | 25.40 | 22.05 | 282 | 7 | 86.18 | 435 | 38 | 7 |
| 4156 | 37.04 | -7.21 | GBIF | 9.07 | 62.53 | 25.08 | 21.72 | 258 | 8 | 86.21 | 398 | 37 | 5 |
| 4157 | 37.08 | -7.28 | GBIF | 9.67 | 61.97 | 25.75 | 22.28 | 277 | 9 | 84.74 | 412 | 44 | 6 |
| 4158 | 37.16 | -7.35 | GBIF | 9.23 | 60.69 | 25.27 | 21.15 | 276 | 6 | 86.60 | 428 | 34 | 15 |
| 4159 | 37.12 | -7.23 | GBIF | 9.87 | 62.45 | 25.80 | 21.90 | 274 | 8 | 85.10 | 405 | 40 | 5 |
| 4160 | 37.04 | -7.36 | GBIF | 9.63 | 60.92 | 25.50 | 22.18 | 283 | 8 | 86.35 | 434 | 38 | 14 |
| 4161 | 36.27 | -4.21 | GBIF | 11.16 | 71.53 | 21.93 | 18.95 | 132 | 0 | 92.47 | 253 | 5 | 6 |
| 4162 | 37.18 | -7.33 | GBIF | 8.99 | 60.75 | 25.07 | 21.02 | 270 | 5 | 85.26 | 421 | 33 | 10 |
| 4163 | 36.89 | -2.94 | GBIF | 12.64 | 72.65 | 22.92 | 19.42 | 130 | 0 | 89.08 | 179 | 4 | 8 |
| 4164 | 36.01 | -3.84 | GBIF | 11.14 | 70.97 | 22.70 | 19.58 | 152 | 1 | 92.85 | 342 | 8 | 8 |
| 4165 | 34.47 | -2.87 | GBIF | 11.61 | 73.01 | 20.95 | 19.17 | 144 | 5 | 75.35 | 143 | 23 | 6 |
| 4166 | 34.61 | -2.13 | GBIF | 12.23 | 79.44 | 22.25 | 20.68 | 147 | 17 | 52.26 | 291 | 89 | 4 |
| 4167 | 31.39 | -6.94 | GBIF | 12.28 | 67.12 | 25.50 | 23.35 | 197 | 0 | 97.75 | 128 | 1 | 5 |
| 4168 | 34.83 | -7.79 | GBIF | 11.65 | 67.34 | 23.58 | 20.60 | 141 | 0 | 106.20 | 162 | 0 | 4 |
| 4169 | 37.84 | -7.82 | GBIF | 8.18 | 63.87 | 27.40 | 24.10 | 229 | 7 | 83.99 | 443 | 34 | 5 |
| 4170 | 37.87 | -7.63 | GBIF | 8.67 | 65.66 | 27.67 | 24.30 | 275 | 13 | 79.97 | 441 | 52 | 4 |
| 4171 | 37.98 | -7.62 | GBIF | 8.82 | 66.29 | 28.22 | 24.92 | 241 | 9 | 79.18 | 425 | 40 | 6 |
| 4172 | 38.00 | -7.73 | GBIF | 8.49 | 65.83 | 27.95 | 24.62 | 207 | 7 | 80.91 | 416 | 33 | 5 |
| 4173 | 33.75 | -3.90 | GBIF | 12.79 | 71.86 | 23.97 | 22.72 | 151 | 1 | 88.50 | 123 | 4 | 16 |
| 4174 | 35.00 | -3.67 | GBIF | 10.07 | 68.95 | 23.48 | 19.88 | 119 | 0 | 95.08 | 273 | 3 | 6 |
| 4175 | 35.00 | -2.42 | GBIF | 11.84 | 76.40 | 20.53 | 18.17 | 127 | 14 | 62.12 | 309 | 58 | 4 |
| 4176 | 35.14 | -2.02 | GBIF | 12.03 | 78.65 | 19.93 | 18.00 | 151 | 18 | 58.15 | 371 | 73 | 4 |
| 4177 | 35.49 | -3.21 | GBIF | 10.14 | 67.61 | 17.92 | 15.02 | 192 | 5 | 81.19 | 436 | 30 | 4 |
| 4178 | 34.96 | -2.17 | GBIF | 12.15 | 78.90 | 20.93 | 18.92 | 142 | 20 | 54.91 | 356 | 81 | 6 |
| 4179 | 34.97 | -7.39 | GBIF | 11.74 | 67.10 | 22.92 | 19.95 | 112 | 0 | 111.21 | 147 | 0 | 5 |
| 4180 | 35.83 | -3.82 | GBIF | 12.32 | 72.45 | 23.80 | 20.63 | 131 | 1 | 90.44 | 311 | 9 | 9 |
| 4181 | 37.63 | -7.58 | GBIF | 7.73 | 61.38 | 25.47 | 21.85 | 342 | 16 | 82.26 | 479 | 64 | 8 |
| 4182 | 34.82 | -3.16 | GBIF | 11.71 | 72.72 | 20.08 | 17.42 | 159 | 2 | 86.16 | 236 | 11 | 5 |
| 4183 | 34.96 | -2.58 | GBIF | 11.99 | 75.42 | 20.20 | 17.67 | 128 | 10 | 68.42 | 318 | 42 | 7 |
| 4184 | 35.15 | -1.66 | GBIF | 12.22 | 81.44 | 20.42 | 18.63 | 154 | 23 | 49.19 | 365 | 106 | 6 |
| 4185 | 35.51 | -3.04 | GBIF | 10.25 | 69.73 | 20.23 | 17.15 | 128 | 2 | 88.55 | 316 | 6 | 4 |
| 4186 | 36.11 | -4.13 | GBIF | 11.59 | 71.55 | 22.58 | 19.62 | 140 | 1 | 91.91 | 244 | 8 | 7 |
| 4187 | 36.07 | -3.98 | GBIF | 11.53 | 71.14 | 22.90 | 19.72 | 142 | 1 | 90.77 | 333 | 8 | 8 |
| 4188 | 37.18 | -7.25 | GBIF | 9.55 | 62.42 | 25.60 | 21.70 | 275 | 7 | 84.91 | 404 | 39 | 9 |
| 4189 | 36.44 | -3.31 | GBIF | 11.70 | 69.23 | 21.15 | 17.72 | 194 | 3 | 91.04 | 294 | 14 | 7 |
| 4190 | 36.53 | -3.21 | GBIF | 10.36 | 68.60 | 19.93 | 16.35 | 170 | 0 | 99.86 | 282 | 1 | 8 |
| 4191 | 34.79 | -2.12 | GBIF | 12.25 | 79.55 | 21.72 | 20.00 | 150 | 20 | 51.63 | 295 | 93 | 4 |
| 4192 | 34.82 | -7.41 | GBIF | 11.83 | 66.48 | 22.83 | 19.73 | 119 | 0 | 110.54 | 154 | 0 | 4 |
| 4193 | 37.54 | -6.92 | GBIF | 10.22 | 65.07 | 25.73 | 22.02 | 229 | 11 | 76.33 | 365 | 45 | 20 |
| 4194 | 35.06 | -7.45 | GBIF | 11.48 | 67.11 | 23.27 | 20.20 | 110 | 0 | 110.42 | 142 | 0 | 4 |
| 4195 | 35.12 | -7.50 | GBIF | 11.52 | 67.01 | 24.48 | 21.45 | 111 | 0 | 108.30 | 138 | 0 | 4 |
| 4196 | 37.61 | -7.42 | GBIF | 8.97 | 64.51 | 28.10 | 24.87 | 312 | 19 | 79.44 | 471 | 68 | 13 |
| 4197 | 37.86 | -7.75 | GBIF | 8.37 | 64.36 | 26.72 | 23.35 | 262 | 8 | 83.09 | 448 | 37 | 4 |
| 4198 | 35.18 | -2.38 | GBIF | 12.02 | 77.53 | 19.88 | 17.45 | 132 | 12 | 66.52 | 351 | 46 | 4 |
| 4199 | 35.01 | -7.58 | GBIF | 12.27 | 68.15 | 24.42 | 21.42 | 123 | 0 | 107.36 | 148 | 0 | 4 |

| 4200 | 36.01 | -3.92 | GBIF | 10.88 | 71.55 | 22.02 | 18.98 | 151 | 0 | 92.72 | 356 | 6 | 8 |
| --- | --- | --- | --- | --- | --- | --- | --- | --- | --- | --- | --- | --- | --- |
| 4201 | 36.52 | -7.51 | GBIF | 9.63 | 62.96 | 25.58 | 23.10 | 237 | 3 | 96.05 | 397 | 20 | 10 |
| 4202 | 36.48 | -7.42 | GBIF | 10.06 | 64.89 | 25.73 | 23.32 | 221 | 4 | 95.64 | 387 | 17 | 9 |
| 4203 | 36.66 | -7.42 | GBIF | 8.96 | 64.45 | 23.07 | 20.48 | 228 | 2 | 91.73 | 403 | 25 | 7 |
| 4204 | 36.51 | -3.27 | GBIF | 9.76 | 65.49 | 17.15 | 14.53 | 261 | 8 | 91.02 | 314 | 35 | 8 |
| 4205 | 34.77 | -2.56 | GBIF | 11.92 | 75.90 | 20.73 | 18.50 | 134 | 12 | 62.97 | 324 | 54 | 9 |
| 4206 | 34.31 | -7.58 | GBIF | 12.20 | 66.67 | 21.80 | 18.98 | 143 | 0 | 109.77 | 190 | 0 | 7 |
| 4207 | 34.79 | -7.66 | GBIF | 12.24 | 67.63 | 23.65 | 20.65 | 134 | 0 | 107.01 | 156 | 0 | 6 |
| 4208 | 34.63 | -7.60 | GBIF | 11.80 | 65.92 | 21.98 | 18.82 | 133 | 0 | 110.74 | 174 | 0 | 6 |
| 4209 | 34.91 | -7.79 | GBIF | 11.32 | 67.81 | 23.43 | 20.57 | 137 | 0 | 105.47 | 161 | 0 | 4 |
| 4210 | 36.84 | -2.97 | GBIF | 12.74 | 73.23 | 22.88 | 19.35 | 127 | 0 | 91.17 | 183 | 3 | 10 |
| 4211 | 35.05 | -1.68 | GBIF | 12.24 | 81.07 | 20.50 | 19.17 | 162 | 24 | 48.63 | 298 | 111 | 5 |
| 4212 | 35.60 | -3.16 | GBIF | 11.07 | 70.99 | 19.38 | 16.65 | 174 | 2 | 89.43 | 393 | 11 | 4 |
| 4213 | 36.06 | -3.96 | GBIF | 11.45 | 71.12 | 22.80 | 19.67 | 140 | 1 | 90.55 | 332 | 8 | 9 |
| 4214 | 34.33 | -2.40 | GBIF | 12.07 | 77.40 | 22.23 | 20.95 | 139 | 9 | 61.57 | 163 | 58 | 7 |
| 4215 | 35.04 | -2.29 | GBIF | 12.13 | 77.72 | 20.68 | 18.45 | 133 | 17 | 59.45 | 303 | 66 | 6 |
| 4216 | 35.02 | -2.10 | GBIF | 12.19 | 78.66 | 20.88 | 18.92 | 144 | 19 | 55.18 | 356 | 80 | 5 |
| 4217 | 35.98 | -3.91 | GBIF | 10.76 | 71.25 | 22.42 | 19.37 | 151 | 1 | 92.07 | 350 | 8 | 7 |
| 4218 | 35.52 | -3.05 | GBIF | 10.04 | 68.78 | 19.50 | 16.37 | 148 | 1 | 90.90 | 360 | 4 | 4 |
| 4219 | 35.98 | -3.86 | GBIF | 10.98 | 70.41 | 22.87 | 19.73 | 149 | 1 | 90.75 | 337 | 10 | 7 |
| 4220 | 36.08 | -3.93 | GBIF | 11.30 | 70.63 | 22.98 | 19.80 | 142 | 1 | 91.24 | 329 | 9 | 8 |
| 4221 | 35.51 | -3.08 | GBIF | 10.06 | 68.89 | 18.92 | 15.80 | 160 | 1 | 90.63 | 390 | 5 | 4 |
| 4222 | 36.18 | -4.15 | GBIF | 11.50 | 71.43 | 22.58 | 19.62 | 141 | 1 | 91.99 | 238 | 8 | 7 |
| 4223 | 36.05 | -3.86 | GBIF | 11.50 | 71.43 | 22.88 | 19.75 | 146 | 1 | 92.17 | 333 | 7 | 8 |
| 4224 | 38.06 | -6.29 | GBIF | 9.64 | 69.36 | 25.53 | 22.92 | 194 | 14 | 67.47 | 320 | 51 | 4 |
| 4225 | 34.69 | -2.42 | GBIF | 11.67 | 77.26 | 21.72 | 19.73 | 128 | 15 | 57.24 | 302 | 70 | 5 |
| 4226 | 34.83 | -2.37 | GBIF | 12.11 | 77.12 | 21.47 | 19.33 | 132 | 17 | 56.81 | 301 | 75 | 7 |
| 4227 | 36.87 | -3.34 | GBIF | 10.57 | 66.04 | 20.62 | 17.73 | 297 | 19 | 93.81 | 253 | 78 | 23 |
| 4228 | 30.53 | -1.49 | GBIF | 11.36 | 83.52 | 20.75 | 20.82 | 152 | 5 | 57.38 | 189 | 116 | 5 |
| 4229 | 30.58 | -1.48 | GBIF | 11.21 | 84.27 | 20.58 | 20.67 | 151 | 6 | 56.91 | 185 | 284 | 5 |
| 4230 | 30.67 | -1.88 | GBIF | 11.48 | 82.61 | 20.83 | 20.82 | 160 | 5 | 60.96 | 163 | 112 | 8 |
| 4231 | 30.75 | -1.88 | GBIF | 10.16 | 82.59 | 20.97 | 20.80 | 145 | 4 | 61.21 | 151 | 98 | 6 |
| 4232 | 30.79 | -1.94 | GBIF | 9.74 | 83.26 | 21.00 | 20.75 | 162 | 4 | 61.87 | 159 | 106 | 9 |
| 4233 | 30.72 | -1.83 | GBIF | 11.07 | 83.21 | 19.87 | 19.95 | 142 | 6 | 59.30 | 155 | 274 | 14 |
| 4234 | 30.74 | -1.91 | GBIF | 10.12 | 82.92 | 21.08 | 20.93 | 149 | 4 | 61.40 | 152 | 96 | 6 |
| 4235 | 30.78 | -1.55 | GBIF | 10.25 | 87.61 | 20.00 | 19.73 | 153 | 5 | 59.14 | 174 | 109 | 8 |
| 4236 | 30.71 | -1.74 | GBIF | 11.02 | 83.46 | 19.85 | 19.92 | 148 | 6 | 59.55 | 160 | 267 | 7 |
| 4237 | 30.63 | -1.90 | GBIF | 11.50 | 82.14 | 20.87 | 20.85 | 160 | 6 | 60.06 | 164 | 122 | 8 |
| 4238 | 30.63 | -1.83 | GBIF | 11.30 | 83.09 | 20.97 | 20.93 | 156 | 5 | 59.76 | 165 | 118 | 7 |
| 4239 | 30.60 | -1.40 | GBIF | 11.24 | 85.81 | 20.67 | 20.62 | 144 | 6 | 55.37 | 189 | 128 | 9 |
| 4240 | 30.71 | -1.68 | GBIF | 10.77 | 82.82 | 21.00 | 21.02 | 149 | 5 | 59.77 | 166 | 265 | 7 |
| 4241 | 30.47 | -1.56 | GBIF | 11.57 | 82.68 | 20.53 | 20.63 | 153 | 6 | 56.74 | 190 | 289 | 6 |
| 4242 | 30.68 | -1.87 | GBIF | 11.43 | 82.85 | 20.97 | 20.92 | 156 | 5 | 60.56 | 161 | 108 | 7 |
| 4243 | 30.66 | -1.77 | GBIF | 11.18 | 83.46 | 20.67 | 20.68 | 155 | 6 | 59.44 | 169 | 296 | 8 |
| 4244 | 30.67 | -1.85 | GBIF | 11.41 | 82.67 | 20.97 | 20.92 | 159 | 5 | 60.63 | 164 | 113 | 7 |
| 4245 | 30.69 | -1.82 | GBIF | 11.28 | 83.58 | 20.32 | 20.33 | 150 | 6 | 59.60 | 162 | 297 | 7 |
| 4246 | 30.78 | -1.97 | GBIF | 10.24 | 82.59 | 21.07 | 20.88 | 153 | 4 | 62.27 | 151 | 99 | 5 |
| 4247 | 30.61 | -1.87 | GBIF | 11.35 | 82.85 | 20.63 | 20.65 | 161 | 7 | 59.72 | 165 | 127 | 8 |
| 4248 | 30.71 | -1.95 | GBIF | 11.02 | 82.83 | 20.85 | 20.80 | 155 | 5 | 61.29 | 152 | 107 | 12 |
| 4249 | 30.78 | -1.93 | GBIF | 9.52 | 84.22 | 20.95 | 20.68 | 169 | 4 | 62.35 | 165 | 105 | 7 |

| 4250 | 30.72 | -1.99 | GBIF | 11.03 | 82.34 | 21.12 | 21.05 | 157 | 4 | 62.46 | 150 | 103 | 7 |
| --- | --- | --- | --- | --- | --- | --- | --- | --- | --- | --- | --- | --- | --- |
| 4251 | 30.64 | -1.41 | GBIF | 10.68 | 87.57 | 20.90 | 20.65 | 142 | 5 | 55.53 | 190 | 116 | 7 |
| 4252 | 30.66 | -1.99 | GBIF | 11.27 | 82.24 | 20.40 | 20.40 | 156 | 6 | 59.95 | 158 | 120 | 9 |
| 4253 | 30.52 | -1.41 | GBIF | 11.57 | 85.05 | 20.82 | 20.90 | 143 | 6 | 55.52 | 190 | 280 | 7 |
| 4254 | 30.65 | -1.87 | GBIF | 11.43 | 82.19 | 20.82 | 20.78 | 158 | 5 | 60.04 | 165 | 116 | 7 |
| 4255 | 30.61 | -1.64 | GBIF | 11.28 | 83.58 | 20.40 | 20.45 | 165 | 7 | 57.62 | 194 | 301 | 5 |
| 4256 | 30.78 | -1.90 | GBIF | 9.25 | 84.09 | 20.93 | 20.67 | 156 | 4 | 61.55 | 157 | 102 | 5 |
| 4257 | 30.67 | -1.57 | GBIF | 11.13 | 83.02 | 19.93 | 20.08 | 155 | 6 | 57.96 | 177 | 286 | 5 |
| 4258 | 30.79 | -1.74 | GBIF | 10.16 | 83.95 | 20.85 | 20.68 | 158 | 5 | 61.27 | 166 | 103 | 6 |
| 4259 | 30.69 | -1.91 | GBIF | 11.25 | 82.72 | 20.42 | 20.37 | 153 | 6 | 60.72 | 155 | 278 | 11 |
| 4260 | 30.78 | -1.68 | GBIF | 10.08 | 85.45 | 20.75 | 20.50 | 156 | 5 | 60.82 | 166 | 108 | 5 |
| 4261 | 30.73 | -1.71 | GBIF | 10.41 | 83.27 | 20.93 | 20.92 | 146 | 5 | 60.00 | 161 | 100 | 7 |
| 4262 | 30.71 | -1.67 | GBIF | 10.94 | 82.89 | 20.92 | 20.92 | 146 | 5 | 58.91 | 169 | 107 | 6 |
| 4263 | 30.77 | -1.62 | GBIF | 10.66 | 86.65 | 20.77 | 20.42 | 163 | 5 | 60.76 | 173 | 113 | 5 |
| 4264 | 30.65 | -1.41 | GBIF | 10.53 | 87.05 | 20.85 | 20.58 | 141 | 5 | 55.88 | 188 | 113 | 7 |
| 4265 | 30.76 | -1.46 | GBIF | 10.68 | 85.47 | 20.77 | 20.53 | 151 | 4 | 58.39 | 182 | 114 | 7 |
| 4266 | 30.79 | -1.41 | GBIF | 11.00 | 84.62 | 20.77 | 20.62 | 158 | 5 | 56.95 | 205 | 128 | 6 |
| 4267 | 30.64 | -1.90 | GBIF | 11.57 | 82.03 | 20.70 | 20.72 | 157 | 6 | 60.04 | 162 | 120 | 8 |
| 4268 | 30.63 | -1.95 | GBIF | 11.43 | 82.85 | 20.10 | 20.12 | 159 | 8 | 58.85 | 165 | 122 | 10 |
| 4269 | 30.77 | -1.93 | GBIF | 9.57 | 83.92 | 20.85 | 20.60 | 164 | 5 | 61.63 | 163 | 106 | 5 |
| 4270 | 30.61 | -1.56 | GBIF | 11.07 | 84.54 | 19.93 | 20.10 | 161 | 7 | 56.10 | 197 | 292 | 5 |
| 4271 | 30.61 | -1.65 | GBIF | 11.32 | 83.83 | 20.80 | 20.87 | 158 | 6 | 58.06 | 182 | 264 | 5 |
| 4272 | 30.70 | -1.44 | GBIF | 10.25 | 86.13 | 20.78 | 20.60 | 150 | 5 | 57.23 | 189 | 113 | 6 |
| 4273 | 30.66 | -1.78 | GBIF | 11.18 | 83.46 | 20.57 | 20.58 | 155 | 6 | 59.61 | 167 | 295 | 11 |
| 4274 | 30.76 | -1.76 | GBIF | 10.80 | 82.44 | 20.80 | 20.70 | 153 | 5 | 60.98 | 160 | 103 | 7 |
| 4275 | 30.63 | -1.51 | GBIF | 11.07 | 83.84 | 20.68 | 20.77 | 156 | 6 | 57.26 | 180 | 283 | 9 |
| 4276 | 30.61 | -1.53 | GBIF | 11.04 | 84.94 | 19.72 | 19.92 | 155 | 7 | 56.52 | 97 | 282 | 5 |
| 4277 | 30.76 | -1.82 | GBIF | 10.49 | 82.61 | 20.98 | 20.85 | 151 | 5 | 61.32 | 155 | 99 | 5 |
| 4278 | 30.55 | -1.35 | GBIF | 11.28 | 85.48 | 20.87 | 20.87 | 141 | 6 | 55.08 | 190 | 130 | 7 |
| 4279 | 30.64 | -1.78 | GBIF | 11.14 | 83.15 | 20.42 | 20.48 | 153 | 6 | 58.98 | 167 | 295 | 8 |
| 4280 | 30.75 | -1.60 | GBIF | 10.74 | 85.93 | 20.73 | 20.45 | 155 | 5 | 59.71 | 171 | 112 | 5 |
| 4281 | 30.75 | -1.79 | GBIF | 11.00 | 82.09 | 20.92 | 20.83 | 150 | 4 | 61.31 | 156 | 278 | 8 |
| 4282 | 30.70 | -1.70 | GBIF | 10.98 | 83.14 | 20.77 | 20.80 | 157 | 5 | 60.06 | 167 | 266 | 14 |
| 4283 | 30.75 | -1.99 | GBIF | 11.02 | 82.28 | 21.08 | 20.98 | 158 | 5 | 61.89 | 155 | 107 | 7 |
| 4284 | 30.69 | -1.93 | GBIF | 11.25 | 82.72 | 20.20 | 20.20 | 153 | 6 | 60.46 | 155 | 108 | 9 |
| 4285 | 29.75 | -0.32 | GBIF | 8.81 | 91.75 | 23.03 | 22.87 | 109 | 27 | 42.12 | 236 | 129 |  |
| 4286 | 29.75 | 0.32 | GBIF | 9.88 | 85.87 | 20.78 | 21.55 | 153 | 79 | 22.55 | 348 | 379 | 8 |
| 4287 | 30.72 | -1.92 | GBIF | 10.50 | 83.33 | 21.08 | 20.98 | 148 | 4 | 61.31 | 152 | 94 | 8 |
| 4288 | 30.65 | -1.89 | GBIF | 11.50 | 82.14 | 20.87 | 20.87 | 158 | 6 | 60.09 | 163 | 118 | 8 |
| 4289 | 30.73 | -1.69 | GBIF | 10.24 | 83.95 | 20.95 | 20.90 | 145 | 5 | 59.56 | 163 | 100 | 7 |
| 4290 | 30.74 | -1.69 | GBIF | 9.65 | 84.65 | 20.82 | 20.67 | 144 | 5 | 59.64 | 164 | 98 | 5 |
| 4291 | 30.64 | -1.69 | GBIF | 11.27 | 83.52 | 20.88 | 20.93 | 163 | 6 | 59.08 | 178 | 119 | 6 |
| 4292 | 30.62 | -1.92 | GBIF | 11.53 | 82.38 | 20.88 | 20.88 | 158 | 6 | 59.61 | 164 | 126 | 9 |
| 4293 | 30.73 | -1.96 | GBIF | 10.32 | 83.20 | 21.03 | 20.90 | 150 | 4 | 61.52 | 152 | 101 | 7 |
| 4294 | 30.80 | -1.76 | GBIF | 10.52 | 83.47 | 20.85 | 20.68 | 161 | 5 | 61.72 | 164 | 107 | 5 |
| 4295 | 41.70 | 1.10 | GBIF | 12.18 | 70.83 | 29.83 | 26.78 | 113 | 5 | 93.66 | 148 | 38 | 6 |
| 4296 | 41.22 | 1.16 | GBIF | 11.31 | 70.68 | 28.62 | 26.92 | 105 | 6 | 98.38 | 140 | 26 | 8 |
| 4297 | 41.93 | 1.12 | GBIF | 12.27 | 70.91 | 29.70 | 30.25 | 115 | 4 | 91.51 | 148 | 50 | 10 |
| 4298 | 41.52 | 1.31 | GBIF | 11.49 | 70.50 | 28.52 | 26.73 | 107 | 5 | 96.85 | 141 | 32 | 11 |

| Thresholds | Scenarios | Loss  km^2^ | remain suitable  km^2^ | Gain  Km^2^ | %  loss | % gain | Range change (%) | Current range size Km^2^ | Future range size Km^2^ |
| --- | --- | --- | --- | --- | --- | --- | --- | --- | --- |
| WM | 2050_4.5 | 389293.35 | 497653.94 | 35921.58 | 43.89 | 4.05 | -39.84 | 886947.29 | 533575.52 |
|  | 2050_8.5 | 478993.33 | 407921.63 | 39750.98 | 54.01 | 4.48 | -49.52 | 886914.95 | 447672.60 |
|  | 2050_4.5 | 481640.73 | 405276.19 | 35438.85 | 54.31 | 4.00 | -50.31 | 886916.92 | 440715.04 |
|  | 2050_8.5 | 632510.28 | 254424.58 | 28571.03 | 71.32 | 3.22 | -68.09 | 886934.86 | 282995.61 |
| 10 percentiles | 2070_4.5 | 336712.70 | 417891.54 | 39611.36 | 44.62 | 5.25 | -39.37 | 754604.24 | 457502.90 |
|  | 2070_8.5 | 418531.61 | 336022.92 | 38626.41 | 55.47 | 5.12 | -50.35 | 754554.53 | 374649.33 |
|  | 2070_4.5 | 417660.06 | 336913.44 | 31577.88 | 55.35 | 4.18 | -51.17 | 754573.50 | 368491.32 |
|  | 2070_8.5 | 551066.37 | 203545.75 | 27086.29 | 73.03 | 3.59 | -69.44 | 754612.12 | 230632.04 |

**TABLE S2** Projected area (km²) of suitable habitat, including gains and losses, for L. africana under future climate scenarios (2050 and 2070) using weighted mean and 10% omission rate thresholds.

*WM = weighted mean, 10% = 10 percentile

**FIGURE S1** Predicted change in habitat suitability of L. africana by 2050s and by 2070s. Green, pixels that are predicted to be suitable under both current and future climates; blue, pixels that are not currently predicted to be suitable but forecasted to be suitable in the future; red, currently suitable but not in the future; and grey, unsuitable both under current and future climates. Maps were generated using two thresholds (weighted mean and 10% (10 percentile omission rate, applied from right to left, respectively) values to evaluate the area change in each scenario.

**FIGURE S2** Mean of weighted mean and 10% (10 percentile omission rate threshold suitable areas size of *L*. *africana* under current and each future climate emission scenarios (2050s and 2070s). An ensemble model outputs for the weighted mean (right) and the 10% omission rate (left).

**TABLE S3** Current range of IUCN, suitable area predicted by Weighted mean (WM) and 10 percentiles, and its average value.

| Countries | Size (km^2^) of IUCN Range for *L*. *africana* per countries | Suitable Area_WM | Suitable Area 10% | Average suitable habitat (km2) |
| --- | --- | --- | --- | --- |
| South Sudan | 316154.60 | 2853.18 | 1480.52 | 2166.85 |
| Eritrea | 5331.50 | 3019.95 | 1803.17 | 2411.56 |
| Ethiopia | 22278.11 | 206182.72 | 158773.29 | 182478.00 |
| Kenya | 130855.57 | 206602.56 | 192136.04 | 199369.30 |
| Somalia | 4500.61 | 9028.94 | 7851.54 | 8440.24 |
| Uganda | 17265.60 | 109120.31 | 96075.58 | 102597.94 |
| United Republic of Tanzania | 391659.73 | 329857.93 | 277147.59 | 303502.76 |
| Rwanda | 1103.27 | 23984.36 | 22565.04 | 23274.70 |
| Total | 889149.01 | 890649.94 | 757832.76 | 824241.35 |

**FIGURE S3**. Current IUCN Range of L. africana across the east African countries (Source: Gobush et al., 2022; the range is summed from Extant (Resident), possibly extant (resident) and extant and reintroduced (resident).
